# Neural control of body-plan axis in regenerating planaria

**DOI:** 10.1371/journal.pcbi.1006904

**Published:** 2019-04-16

**Authors:** Alexis Pietak, Johanna Bischof, Joshua LaPalme, Junji Morokuma, Michael Levin

**Affiliations:** 1 Allen Discovery Center, Tufts University, Medford, Massachusetts, United States of America; 2 Department of Biology, Tufts University, Medford, Massachusetts, United States of America; Purdue University, UNITED STATES

## Abstract

Control of axial polarity during regeneration is a crucial open question. We developed a quantitative model of regenerating planaria, which elucidates self-assembly mechanisms of morphogen gradients required for robust body-plan control. The computational model has been developed to predict the fraction of heteromorphoses expected in a population of regenerating planaria fragments subjected to different treatments, and for fragments originating from different regions along the anterior-posterior and medio-lateral axis. This allows for a direct comparison between computational and experimental regeneration outcomes. Vector transport of morphogens was identified as a fundamental requirement to account for virtually scale-free self-assembly of the morphogen gradients observed in planarian homeostasis and regeneration. The model correctly describes altered body-plans following many known experimental manipulations, and accurately predicts outcomes of novel cutting scenarios, which we tested. We show that the vector transport field coincides with the alignment of nerve axons distributed throughout the planarian tissue, and demonstrate that the head-tail axis is controlled by the net polarity of neurons in a regenerating fragment. This model provides a comprehensive framework for mechanistically understanding fundamental aspects of body-plan regulation, and sheds new light on the role of the nervous system in directing growth and form.

## Author summary

Understanding how large-scale anatomy emerges from the activity of cellular pathways is a key goal of evolutionary developmental biology. Elucidating the rules of body-wide morphogenesis is especially essential for transitioning molecular signaling data at the cellular level into advances in regenerative biomedicine. We constructed and analyzed a comprehensive, multiscale computational model to explain the determination of axial polarity during planarian regeneration. Uniquely, our model explains the various head-tail patterning outcomes of a wide range of molecular and physiological manipulations. Testing the novel predictions of this model revealed the nervous system as an instructive regulator of axial patterning.

## Introduction

Humanity’s inability to regenerate significant portions of anatomy (e.g. lost limbs, severed spinal cord, damaged organs) has prompted decades of intensive study into organisms that can. The planarian flatworm is one such model organism, as it can completely regrow from a piece as small as ~1/250^th^ of the original worm [1, 2]. Crucially, this regenerating organism exhibits complex behaviors and properties functioning well beyond the level of a single cell. It can maintain body-plan homeostasis under normal circumstances, including body-wide allometric remodeling during growth and shrinkage depending on availability of food, while also detecting injury to precisely reproduce missing features, and stopping regeneration once the correct body structure (the *target morphology*) has been restored [3, 4]. Therefore, a deep and functional understanding of regeneration requires, not only an account of single cell activities such as gene expression [5], but also of the regenerating organism as an intricate system exhibiting complex dynamics and coordination over multiple levels of scale. How do patterning control systems scale to enable correct regeneration of fragments of vastly different sizes and shapes? What tissue-level properties underlie long-range coordination of anatomical outcomes? What is the role of the nervous system in regeneration? All of these open questions have major implications, not only for evolutionary and developmental biology, but also for implementing rational repair strategies in the regenerative medicine of complex organs.

**Fig 1:**
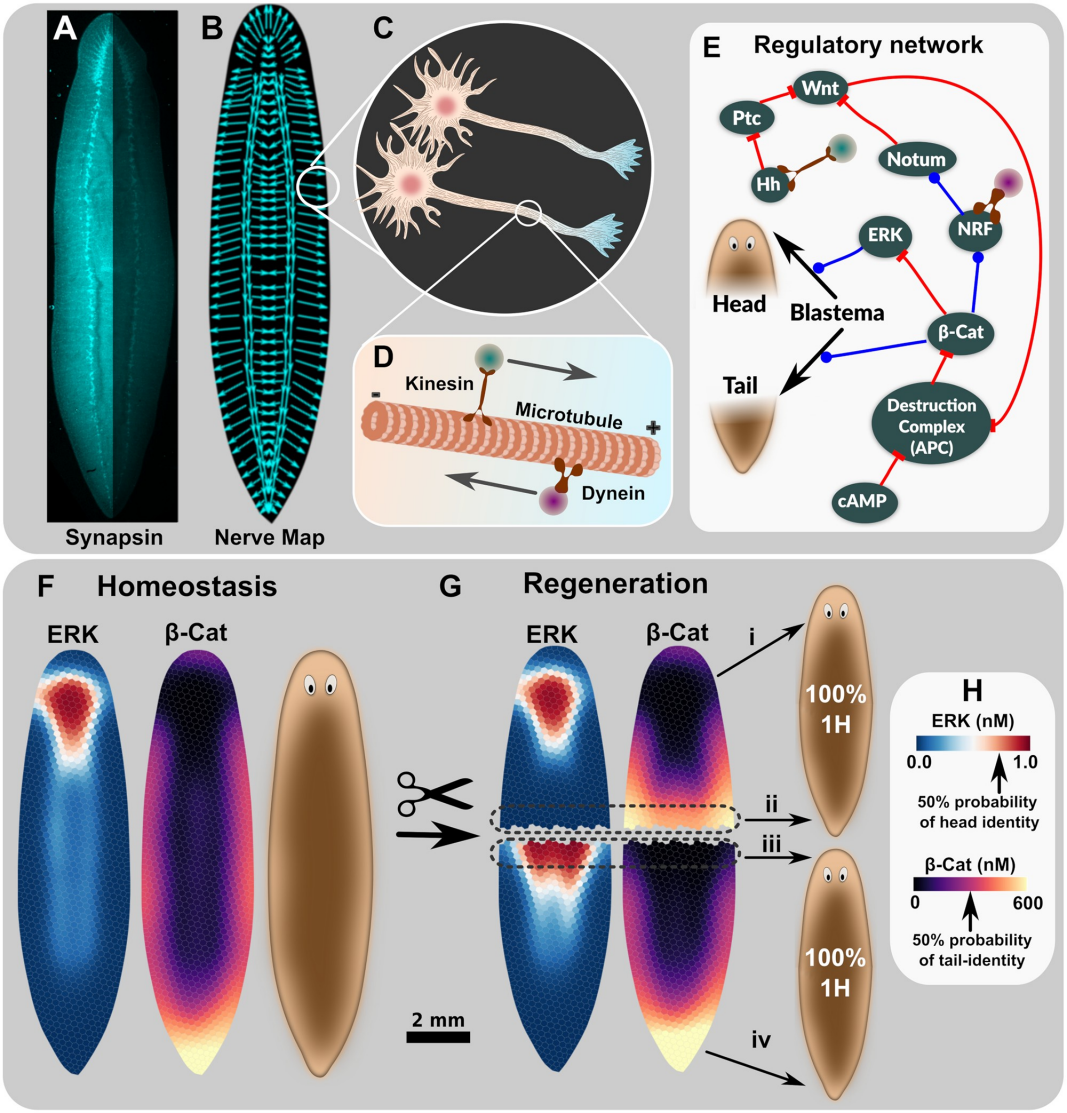
A conceptual summary of our model of anterior-posterior axis control in planaria regeneration. We hypothesize morphogens to be transported on a vector field coincident with neuronal axons (A), with hypothesized transport polarity as shown in (B). We further hypothesize that vector transport of morphogens occurs on aligned microtubule arrays in nerve axons (C, D). A regulatory network (E) integrates molecular-genetic interactions with vector transport in nerves to produce stable, self-assembling morphogen gradients consistent with planarian body-plan in homeostasis (F), and capable of responding to cutting event perturbations to establish new gradients (G). Head and tail regeneration are assumed to be related to concentrations of ERK and *β*-Cat, respectively (F, G, H). The percentage of regenerated heteromorphoses in a population was estimated from steady-state ERK and *β*-Cat gradients after cutting (G), where average morphogen concentration at wound edges (G *ii*, and *iii*) were used to define the probability of the blastema developing into a head or tail (G, H) using a Markov Chain Model for Regeneration (see text and Fig 3 for details). Head (G, transition ‘*i*’) and tail (G, transition ‘*iv*’) regions existing on a fragment prior to cutting were assumed to be terminally differentiated, and to therefore remain as head or tail in the regenerated fragment. In the regulatory network of E, blue lines with circular endpoints represent activating interaction, whereas red lines with flat-line ends represent an inhibitory relationship. Kinesin symbol on Hedgehog node, and dynein symbol on NRF, signify proposed transport of the respective factors. Hh—Hedgehog, Ptc—Patched, Wnt—Wingless/integrated (Wnt1 and Wnt11 combined), *β*-Cat—*β*-Catenin, ERK—extracellular-signal regulated kinase, cAMP—cyclic adenosine monophophate, NRF—Notum Regulating Factor.

Body-plan consists of precisely-organized collectives of different cell types, where cells receive cues to proliferate and differentiate by responding to morphogens, including molecular-genetic [6–8], mechanical [9–11], and bioelectrical [12–14] signals. Therefore, spatial patterns of morphogens can serve as instructive *pre-patterns* to induce changes to single cell identity and ultimately specify aspects of the final body-plan [6, 15–18] (Fig 1). Planaria maintain a population of pluripotent adult stem cells (*neoblasts*), which migrate to wounds to form a blastema, where subsequent proliferation and differentiation ultimately regenerates lost portions of head, trunk, and tail [19–22]. Neoblasts respond to morphogenetic gene products [19, 20, 22–24] and chemical messengers [25–27] to produce the needed cell types to transition the blastema at the former anterior portion of a wound into a new head, while creating tails at wound sites facing the original posterior.

**Fig 2:**
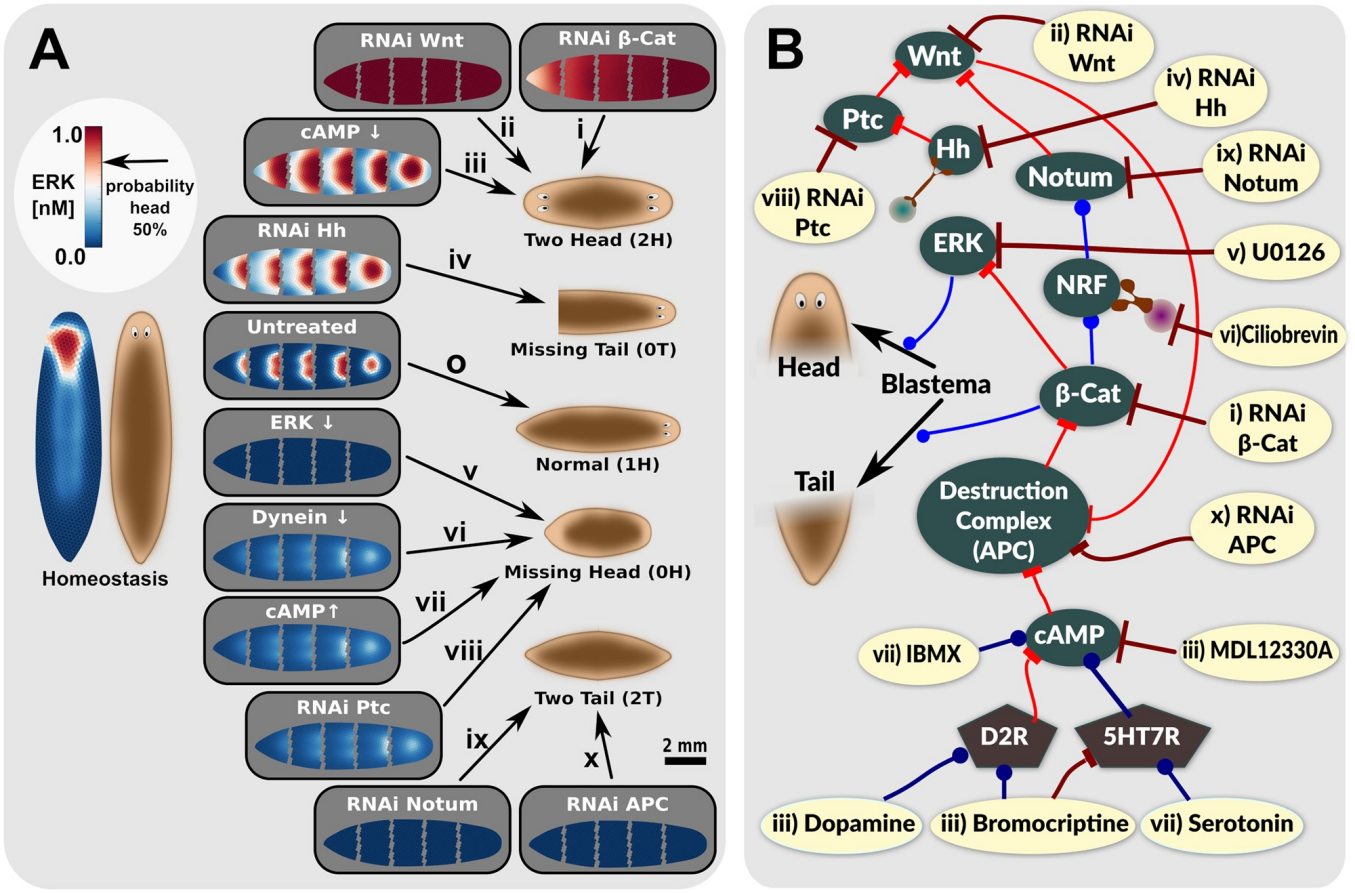
Planarian body-plan can be altered by a variety of treatments, which are also consistent with various steady-state outcomes of our computational model. Heteromorphoses resulting from 10 different RNAi/pharmacological manipulations (A ‘*i*’ through ‘*x*’) are predicted by experimentally-consistent perturbations to our regulatory network model (A ‘*i*’ through ‘*x*’ and B). Experimentally-consistent model-predicted morphogen gradients are found for homeostasis (A, leftmost panel), normal wild-type regeneration (A, transition ‘o’), and interventions associated with abnormal posterior regeneration (head on the posterior to regenerate 2H, A, transition ‘*i*’ through ‘*iii*’), abnormal posterior regeneration (e.g. loss of tail, A ‘*iv*’), abnormal anterior regeneration (loss of head, A transitions ‘*v*’ to ‘*viii*’), or duplication of tail on the anterior (A, transition ‘*ix*’ and ‘*x*’). Specific sites of the regulatory network model where treatments ‘*i*’ through ‘*x*’ act as perturbations, are shown as yellow ellipses in (B). References to intervention outcomes are summarized in Table 1. In the regulatory network of B, blue lines with circular endpoints represent activating interaction, whereas red lines with flat-line ends represent an inhibitory relationship. Dark blue lines represent a proposed activation interaction induced by a pharmacological agent, whereas dark red arrows represent a proposed inhibition interaction induced by a pharmacological agent or RNAi. Dopamine 2 receptor—D2R, Serotonin 7 receptor—5HT7R.

For whole planaria in morphological homeostasis, factors such as *β*-Catenin (*β*-Cat), Wingless/Integrated (Wnt), extracellular-regulated receptor kinase (ERK), Notum, as well as other position control genes (PCGs), are found as concentration gradients with characteristic polarities with respect to the anterior-posterior axis of the worm [18, 24, 28–35]. Remarkably, during early regeneration (from 15 to 72 hours after injury), these same morphogens spontaneously reform their original polarity in each fragment of a worm cut into multiple pieces [24, 28–34]. Consistent with the concept of positional information regulating the body-plan, various heteromorphoses can be generated through experimentally-applied genetic and pharmacological interventions, which act by influencing levels of morphogens involved in anterior-posterior axis control [32, 36, 37] (Fig 2, Table 1).

**Table 1:** A summary of gene products and chemical messengers that are known to alter regenerated planarian body-plan when inhibited by RNAi or pharmacological agents.

Genetically-Expressed Factors	Exhibits gradient?	Blocking agent	Effect of blocking	References
Wnt1 & Wnt11 (Wnt)	Yes, posterior max	RNAi	Head duplication (2H)	[33, 37, 38]
Hedgehog (Hh)	No	RNAi	Loss of tail (0T)	[39, 40]
Notum	Yes, anterior max	RNAi	Loss of head (0H)	[32]
Extracellular receptor kinase (ERK)	Yes, anterior max	RNAi or U0126	Loss of head (0H)	[24, 30, 31]
*β*-Catenin (*β*-Cat)	Yes, posterior max	RNAi	Head duplication (2H)	[36, 41]
Adenomatous polyposis coli (APC)	*Unknown*		Loss of head (0H) & tail duplication (2T)	[34]
**Chemical Messengers**				
Cyclic adenosine monophosphate (cAMP)	*Unknown*	MDL12330A	Head duplication (2H)	This work
Serotonin	*Unknown*	Bromocriptine (via 5HT7R-like)	Head duplication (2H)	[25, 42]

A major knowledge gap exists in determining how wound-induced repolarization of morphogens occurs in each fragment of a cut planaria. Reaction-diffusion mechanisms (e.g. diffusing proteinaceous transcription products or other signaling factors that regulate each other’s expression and activity levels), have long been proposed to account for self-assembly of molecular pre-patterns in a variety of biological systems [6, 15, 17, 43, 44], including the gradients observed in planaria homeostasis and regeneration [45–47]. However, the dependence of reaction-diffusion mechanisms on the size of a biological system—which is an intrinsic feature of most reaction-diffusion models—may limit their ability to account for chemical gradients in planaria fragments of diverse size [48, 49]. Specifically, for a system of interacting molecules with fixed rates of diffusion and other physically-constrained factors, a gradient may spontaneously develop for a system at a certain size, but this pattern will drastically change (e.g. a categorical change in pattern type from smooth gradient to stripes or spots) when the system size increases [48, 49]. This scale-dependence of reaction-diffusion schemes can be mitigated by incorporating directional transport, here termed *vector transport*, of substances into the reaction-diffusion scheme, which has been shown to robustly account for self-assembly of concentration gradients in a manner essentially independent of size scale [48, 50, 51]. The complex nature of emergent outcomes driven by such processes underscore the essential need to simulate models of regeneration in a spatialized framework to fully determine their large-scale patterning properties, extract testable predictions, and ultimately develop strategies for rational modulation of outcomes in biomedical and synthetic biology settings.

Here we present, and quantitatively analyze, an inclusive model of the molecular regulation underlying planarian regeneration, synthesizing much published work on the results of physiological and molecular-genetic manipulations (Tables 1 and 2). Our model combines a molecular signaling network at the cellular level with vector transport of morphogens (e.g. the directional transport of morphogens by a vector field), and correctly predicts regenerative patterning outcomes in a variety of settings, including editing of regenerative pattern without use of molecular or genetic manipulations. Our model also explains several outstanding puzzles in planarian regeneration, such as how self-assembling, self-scaling morphogen gradients can form in pieces of different size and location across the worm; the mechanistic origin of various heteromorphoses (e.g. one-headed, two-headed, missing-head or missing-tail outcomes) under a range of pharmacological and RNAi interventions; and the instructive role of the nervous system in regeneration. We also tested a number of unique predictions of this model experimentally (Table 2). Hypothesizing that vector transport can take place on the aligned microtubules of nerves enabled the experimental discovery of the crucial role for nerve directionality in determining the AP polarity of fragments, including the re-setting of pre-existing polarity. We show how cooperation of biophysical and molecular-genetic processes can functionally link single cell physiology and long-range anatomical patterning. Importantly, while this work explores mechanisms enabling body-plan regeneration and target morphology in the context of planarian regeneration (Fig 1), the underlying concepts reveal a powerful, multi-scale patterning system that is applicable to a wide range of biological systems and contexts, such as embryogenesis, regeneration, cancer, and the bioengineering of organoids [52, 53].

**Table 2:** Summary of model predictions and their state of experimental validation.

	Model hypothesis/prediction	Experimental Support
1	Vector transport of morphogens occurs relative to planaria nervous system axon alignment.	Previously hypothesized in [30, 31, 39] for VNC; tested herein, see Figs 5, 6 and 7.
2	Vector transport of morphogens is mediated by kinesin/dynein motor proteins.	Tested herein, dynein inhibition inhibited head formation, consistent with model prediction.
3	Vector transport of morphogens requires microtubules.	Previously observed axis disruption with microtubule destabilizers (see S5 Fig) [54, 55].
4	Hh is subjected to vector transport to the posterior via kinesin.	Hh postulated to be transported in nerves [30, 31, 39]; indirectly tested herein, see Fig 4. To be tested further in future work when materials/technology become available.
5	NRF and Hh are expressed in nerves.	Hh detected in neurons [30, 39]. To be tested further in future work when materials/technology become available.
6	Two-headed worms have a bipolar nervous system and bipolar transport map.	Previously reported via cilia flow assay [56]; re-tested herein, see Fig 7. To be tested further in future work when materials/technology become available.
7	Axoplasmic transport network of planaria nervous system corresponds to form of vector maps detailed here.	Planaria neural transport network previously assessed, but reportedly difficult to trace [57]; to be tested further in future work when materials/technology become available.
8	Full RNAi of *β*-Cat leads to complete loss of AP axis.	Previously reported [29].
9	Partial RNAi of *β*-Cat leads to more 2H regenerates closer to the head.	Tested herein, see Fig 4.
10	Interventions decreasing cAMP leading to more 2H regenerates closer to tail.	Tested herein, see Table 3 and Fig 4.
11	Hh RNAi leading to loss of tail regeneration (0T).	Previously reported [39, 58].
12	Blocking ERK signaling leads to loss of head regeneration (0H).	Previously reported [30, 31]; tested herein see Table 3.
13	Increasing serotonin leads to headless outcomes via cAMP decrease (0H).	Previously reported [25, 42]; tested herein see Table 3.
14	Inhibition of Notum leads to 2T and 0H outcomes.	Previously reported [32].
15	Inhibition of *β*-Cat or Wnt1 and Wnt11 leads to 2H with loss of Notum.	Previously reported [32].
16	Appearance of Notum gradient at anterior facing wounds with Wnt and *β*-Cat at posterior-facing wounds.	Previously reported [32].
17	RNAi of APC leads to 2T and 0H outcomes with Notum polarity maintained.	Previously reported [34].
18	RNAi of Ptc leads to 0H and 2T outcomes.	Previously reported [39, 40].
19	NRF is subjected to vector transport to the anterior by dynein.	Indirectly tested herein as dynein inhibition inhibits head formation, consistent with inhibition of NRF transport and correlated loss of Notum expression. To be tested further in future work when materials/technology become available.

## Methods

### Planaria computational model

Our model is hierarchical and multifactorial in nature, connecting several major concepts. Details of our theory, model, and software are described in S1 Text.

The planaria models presented herein were formally implemented and explored using the **Pl**anarian **I**nterface for **M**odeling **B**ody **O**rganization (PLIMBO), a 1D and 2D finite volume method simulator written in Python3 with open-source tools utilized from Scipy, Numpy, Matplotlib, Scikit-learn [59, 60], and BETSE [61, 62]. PLIMBO was developed specifically to study all aspects of the planaria regeneration modeling reported in this manuscript. PLIMBO allows quantitative testing of the behavior of the regulatory network model reported on herein, in both 1D and 2D contexts, and under a range of experimental perturbations, with vector (convective) transport of morphogens on imported nerve polarity vector fields, as well as the extraction of novel testable predictions. PLIMBO also has the capacity to run parameter searches to automatically iterate parameters to assist model parameterization, to perform sensitivity analyses, and to perform scaling analysis of the model (where a body-shape is progressively scaled); these tools all assist in the development and exploration of complex biological models. PLIMBO is freely available from: https://gitlab.com/betse/plimbo.

Our planarian regeneration model requires the biophysical mechanism of vector transport to create virtually scale-free self-assembly of morphogen gradients instructive for planarian body-plan homeostasis and regeneration, a premise which extends conventional reaction-diffusion mechanisms by adding a convective transport term, the fundamental properties of which have been explored elsewhere [48, 50, 51]. Here we further propose transport of morphogens on polarized microtubules of nerve axons distributed throughout the planarian tissue (i.e. not just the ventral nerve cords, VNC) as the specific vector transport mechanism (Fig 1B, 1C and 1D). Vector transport of Hh on the VNC has been previously hypothesized and qualitatively reported, but not subjected to a formal analysis to determine feasibility and predictions [30, 31, 39].

Experimental investigations into neuronal polarity and axoplasmic transport networks of planaria [63] are reportedly very difficult undertakings, and have produced results that are not useful for the scope required by this present work. Therefore, we estimated maps of nerve axon polarities (Fig 1B), which were derived by manually tracing synapsin stains to determine approximate axon angle at various locations in the planaria tissue cross-section (Fig 1A). The manual traces were assumed to be vector fields supporting directional transport of morphogens (Fig 1B). A summary of all nerve map models is shown in S1 Fig.

#### Creation of planaria models and neural transport and production fields

Two-dimensional planaria models were created by tracing synapsin stains (S1 Fig columns A and E) to obtain realistic estimates of body shapes, proportions, and axon angles throughout the tissue. Experimentally-derived synapsin stains were straightened along the central midline using an ImageJ feature (’straighten’). In PLIMBO, a clipped and optimized Voronoi grid (1500–2000 grid elements per model) was generated within the domain prescribed by the desired planaria body shape, thereby creating planaria models with a modeled length from 0.5 to 2.5 cm (all models shown in main figures measured 1.20 cm in simulated length, which is consistent with average worm size used in experiments). A finite volume technique was used to model transport of substances within the body (as described in detail elsewhere [61]), where the edge of the planaria body or wound was treated as a closed boundary. Three replicates each of 1H and 2H worm heteromorphoses were generated (S1 Fig). A production gradient, *G*(*x*, *y*), intended to represent the distribution of nerve bodies in the planaria, was used to modulate production of morphogens subjected to axoplasmic transport. The production field was estimated by taking the divergence of the vector transport field, $\vec{u}\left(x,y\right)$, defining the neural traces (S1 Fig columns C and G). The production gradient *G*(*x*, *y*) was normalized to a maximum of 1.0 and used as a multiplier of chemical dynamics equations. The normalized vector transport field, $\vec{u}\left(x,y\right)$, in combination with its associated production gradient *G*(*x*, *y*), and the model’s regulatory network (Fig 1E, with details in S1 Fig), produced self-assembling morphogen gradients consistent with observed regeneration outcomes (e.g. Figs 1F, 1G and 2).

#### Regulatory network model

A regulatory network at the core of the model included major molecular signals known to be involved in regulation of anterior-posterior axis regeneration in planaria (Fig 1E and Tables 1 and 2). Importantly, the nodes and relationships shown in Fig 1E are consistent with existing experiments described in the literature (see Tables 1 and 2), and represents the simplest model that can account for changes to planarian body-plan, which occur following major known RNAi and pharmacological intervention experiments. The morphogen Hh is assumed to be produced in neurons, and to be moved away from neural cell bodies by kinesin [64, 65], while a presently unidentified substance, Notum Regulating Factor (NRF), is moved back towards neural bodies by dynein [66]. Factors ERK, Wnt, adenomatous polyposis coli (APC), cyclic adenosine monophosphate (cAMP), and Notum are assumed to have the capacity to be expressed homogeneously in all cells. Substances of the regulatory network have production/decay conditions that are often modulated by other substances of the regulatory network, as shown schematically in Fig 1E, with details in S1 Text. The outer edges of the planaria shape (or wound) are considered a closed (i.e. zero-flux) boundary condition. The initial conditions for all molecular factors of the model were set at zero values, except for cAMP which was set at 1.0 for all cells of a simulation not involving a cAMP-based intervention.

Each substance of the regulatory network could contain terms of the following form (shown in Eq 2 for a hypothetical concentration *C*_*i*_ regulated by substances *C*_*j*_ and *C*_*k*_), where specific equations and free parameters for each substance of the regulatory network are listed in S1 Text:

$$\begin{array}{c} \underset{\text{Mass}\;\text{flux}}{\underset{︸}{\phi_{i}}}=\underset{\text{Diffusion}}{\underset{︸}{-D_{i}\;\nabla C_{i}}}+\underset{\text{Motor}\;\text{protein}\;\text{convection}}{\underset{︸}{\alpha_{i}\;\vec{u}C_{i}}} \end{array}$$ (1)

$$\begin{array}{c} \underset{\text{Rate}\;\text{of}\;\text{Change}}{\underset{︸}{\frac{dC_{i}}{dt}}}=r_{i}^{max}\underset{\text{Growth}\;\text{Inhibitor}}{\underset{︸}{\left(\frac{1}{1+{\left(\frac{C_{j}}{K_{j}}\right)}^{n_{j}}}\right)}}\underset{\text{Growth}\;\text{Activator}}{\underset{︸}{\left(\frac{{\left(\frac{C_{k}}{K_{k}}\right)}^{n_{k}}}{1+{\left(\frac{C_{k}}{K_{k}}\right)}^{n_{k}}}\right)}}-\delta_{i}\;C_{i}\underset{\text{Decay}\;\text{Inhibitor}}{\underset{︸}{\left(\frac{1}{1+{\left(\frac{C_{k}}{K_{k}}\right)}^{n_{k}}}\right)}}\;\underset{\text{Decay}\;\text{Activator}}{\underset{︸}{\left(\frac{{\left(\frac{C_{j}}{K_{j}}\right)}^{n_{j}}}{1+{\left(\frac{C_{j}}{K_{j}}\right)}^{n_{j}}}\right)}}-\underset{\text{Divergence}\;\text{of}\;\text{flux}}{\underset{︸}{\nabla \cdot \phi_{i}}} \end{array}$$ (2)

Concentrations were updated in time using the explicit Euler method. Model stability with time-step (Δt) and minimum grid resolution (Δx) were rigorously tested and results were found to be stable and invariant for time-steps smaller than $△t<\frac{△x^{2}}{3\;D_{max}}$, where *D*_*max*_ represents the largest diffusion constant in the network (*D*_*max*_ = 1.5*x*10^−11^ *m*^2^/*s*). All simulations used time-steps half the size of the stability limit to ensure results were produced in a stable and invariant range.

#### Model parameterization and sensitivity analysis

Detailed information about model parameterization can be found in the S1 Text. Biologically realistic values were chosen for all parameters, based on thorough literature searches (see S1 Text for details). The planaria model was first parameterized in 1D, which features rapid simulation times that allow for high-throughput model testing. Notably, while using a simple 1D transport field, the 1D model shows similar results to that of the 2D model (see Supporting Figures and S1–S4 Videos). After achieving adequate results in 1D, the model was transitioned to the full 2D version using a body shape, production gradient *G*(*x*, *y*) and neural transport map $\vec{u}\left(x,y\right)$ for one of the models shown in the S1 Fig. A local sensitivity analysis was performed on both 1D and 2D models to identify parameters with the highest impact on model outcome, and to screen out parameters with the lowest impact (see S1 Text for details). The sensitivity analysis also showed the model results to depend strongly on the majority of parameters of the model (see S1 Text for details). Note that units stated on modeled concentrations are model-specific/arbitrary and are intended to be relative to the biological reality, rather than an absolute measure. While concentration magnitudes are relative, they were largely sourced from reports of canonical Wnt/*β*-Cat signaling pathways [67, 68] and other studies [69, 70].

#### Simulation of amputation, RNAi experiments, and pharmacological applications

Cutting of planaria was dynamically simulated by directly removing material from models at steady-state and re-establishing closed boundary conditions at the wounded boundaries. Models were further perturbed by knocking out or altering levels of regulatory network nodes to simulate a variety of RNAi or pharmacological interventions (see Fig 2). To examine the effect of an intervention (e.g. RNAi experiment or pharmacological agent), models were first initialized for 4.5 days of simulated time with original parameter values to obtain steady-state morphogen patterns, at which time the model was cut into fragments, with the introduction of the intervention to the model. All intervention models were examined 4.5 simulated days after cutting, when morphogen patterns were re-established. RNAi simulations (RNAi to Notum, Wnt, Hh, Ptc, and APC) were modeled by setting the growth rate of the inhibited substances to zero (specifically by multiplying their growth constant by 0.0).

Note that due to the long decay time of *β*-Cat in the system, for a ‘full’ *β*-Cat RNAi, as shown in Fig 2Ai, inhibition of *β*-Cat signaling was applied to the whole worm after the initial 7 day model initialization, and a ‘re-initialization’ phase consisting of a further 7 days of simulation of the whole worm with inhibited *β*-Cat growth was conducted. The re-initialization reduces levels of *β*-Cat further by allowing them time to decay in the whole worm, and is consistent with experimental technique for RNAi to *β*-Cat. The model was then cut into fragments and simulated for 4.5 days after the re-initialization phase, with *β*-Cat expression continued to be inhibited. For ‘partial’ RNAi block of *β*-Cat, the intervention was introduced at the time of cutting, and was applied with a shorter re-initialization phase of only 48 hours. Pharmacological intervention simulations involved modeling changes to cAMP levels (representing application of pharmacological agents such as IBMX [71], MDL12330A [72], neurotransmitter signaling enhancers such as bromocriptine [42, 73], or direct application of serotonin and dopamine [25, 42]), and block of ERK signaling via U0126, were modeled by introducing the intervention to a 7-day initialized and cut model at the time of cutting, and applying the intervention for 4.5 days of simulated time after cutting. A decrease in cAMP (and application of dopamine, a factor known to reduce cAMP via D2R receptor activation [74]) was modeled as a 0.25x decrease in the growth rate of cAMP, whereas an increase in cAMP (and application of serotonin, a factor known to increase cAMP via 5HTR7 activation [25, 42]) was modeled as a 5x increase in the growth rate of cAMP. ERK was inhibited by multiplying the growth rate of ERK by 0.0. Morphogen patterns were reported 4.5 days after cutting with natural decay rates of all substances preserved.

The resulting model produces stable, self-assembling morphogen gradients as outputs, which examine the functional relationships between morphogen patterns, cell/tissue patterns, and body-plan in homeostasis (e.g. Fig 1F) and with perturbation of the model by cutting events (e.g. Fig 1G). Supporting S3 and S4 Videos show the dynamics of key morphogen gradients during model initialization and after a cutting event in 2D, highlighting the ability for the system to maintain a steady-state, and yet to adapt to a perturbation such as cutting to acquire a new steady-state capable of directing regeneration. Note that the PLIMBO software includes configuration files and information to run all planaria models and associated analyses.

#### Markov Chain Model of Regeneration

**Fig 3:**
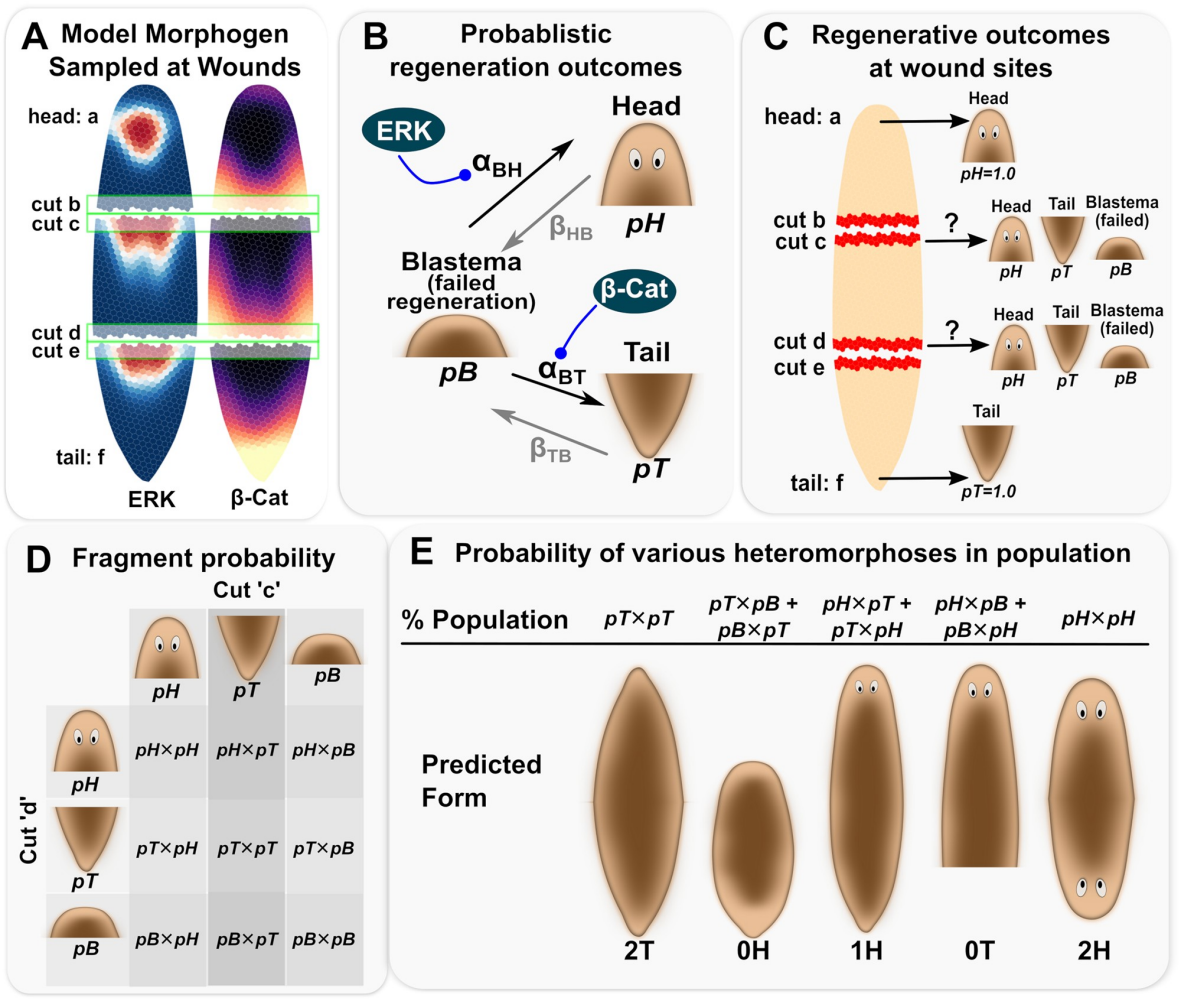
Key concepts underlying the Markov Chain Model of Regeneration used to predict regeneration outcomes from modeled steady-state morphogen concentrations averaged at the wound sites of the model. Concentrations of key instructive morphogens (ERK and *β*-Cat) were sampled and averaged at each model cut line (A). The base Markov Chain assumed possible transitions of the blastema at the cutting site into a potential head or tail, with the additional case that failure to transition to head or tail represents a failed regeneration (B). Head, tail, or blastema (failed) probabilities were represented by *pH*, *pT*, and *pB*, respectively (B, C). Transition rates from blastema to head (*α*_*BH*_) were assumed to be increased by ERK concentrations at the wound (B, Eq 3), while transitions from blastema to tail (*α*_*BT*_) were assumed to be increased by *β*-Cat concentrations at the wound (B, Eq 4). Regeneration outcomes at cut-lines were calculated probabilistically, where existing head and tail remained unchanged on top and bottommost fragments (C). To determine the frequencies of heteromorphoses in a particular fragment appearing in a population of regenerates, the probabilities of each possible outcome at each cut line of the fragment were multiplied (D). The respective predicted fraction of heteromorphoses appearing in the population were inferred from the combinations shown in (D), where the possible heteromorphoses and their net probabilities are shown in (E).

The final component of our model works from the fundamental hypothesis that molecular morphogen gradients ultimately specify the anterior-posterior head/tail decision by changing blastema identity (via morphogen influence on neoblast differentiation). Conceptually, neoblasts located at the blastema of a wounded region were assumed to probabilistically differentiate towards anterior or posterior lineages, thereby shaping the identity of the blastema towards the regeneration of a head or tail at the wound (Fig 1G). Consistent with experimental reports, ERK concentrations were assumed to promote development of an anterior/head identity [24, 30, 31], while *β*-Cat was assumed to promote establishment of posterior/tail identity [36, 37]. A Markov Chain Model of Regeneration was developed to predict regeneration outcomes in a population of planaria under the influence of steady-state morphogen concentration levels at wound sites of the model. The premise of the Markov Model is summarized in Fig 3. The transition probabilities from blastema to head (*α*_*BH*_) and from blastema to tail (*α*_*BT*_) were estimated in terms of averaged concentrations of ERK and *β*-Cat (respectively) at wound edges (Fig 3A and 3B):

$$\begin{array}{c} \alpha_{BH}=\frac{1}{1+exp(-(\frac{C_{ERK}-C_{1}}{\kappa_{1}}))} \end{array}$$ (3)

$$\begin{array}{c} \alpha_{BT}=\frac{1}{1+exp(-(\frac{C_{\beta Cat}-C_{2}}{\kappa_{2}}))} \end{array}$$ (4)

Where *C*_1_ = 0.75 nM, *C*_2_ = 300 nM, *κ*_1_ = 0.05 nM, and *κ*_2_ = 40.0 nM. Values for the reverse transitions were set as constants, where *β*_*HB*_ = *β*_*TB*_ = 5.0*x*10^−3^. These parameter values were chosen based on inspection of ERK and *β*-Cat morphogen gradients produced by model output under the normal regeneration condition (i.e. without an applied intervention) and choosing values that appropriately prescribe head and tail regions at anterior and posterior wound sites.

At the site of a wound, the probability of a regenerated head (*p*_*H*_), tail (*p*_*T*_), or failure to regenerate either head or tail (*p*_*B*_), were computed in terms of transition probabilities between states under the constraint that all probabilities must sum to 1.0, where the probabilities of a blastema proceeding to different outcomes were dynamically expressed as:

$$\begin{array}{c} \frac{dp_{H}}{dt}=(\alpha_{BH}-T\;\alpha_{BH}-H\;\left(\alpha_{BH}+\beta_{BH}\right))\;C_{wound} \end{array}$$ (5)

$$\begin{array}{c} \frac{dp_{T}}{dt}=(\alpha_{BT}-H\;\alpha_{BT}-T\;\left(\alpha_{BT}+\beta_{BT}\right))\;C_{wound} \end{array}$$ (6)

$$\begin{array}{c} p_{B}=1-p_{H}-p_{T} \end{array}$$ (7)

$$\begin{array}{c} C_{wound}=1-\frac{1}{1+exp(-g\left(t-t_{f}\right))} \end{array}$$ (8)

Equations describing anatomical outcome probabilities were solved dynamically (i.e. at each time-step of the iterated model) to yield regeneration probabilities as a function of time, which were sampled for cells at a particular cut line to determine the probability of head/tail at the site of a wound (Fig 3C).

Note that regeneration was assumed to be a time-dependent process induced by wounding and proceeding at the site of the wound. Fixing a time-dependence to regeneration was necessary to describe RNAi interventions such as partial RNAi to *β*-Cat, where morphogen levels continue to decrease to zero over time. Regeneration time-dependence was modeled by modulating probability for head and tail developments by a molecular wound signal factor (*C*_*wound*_), which is proposed to be a molecular substance transiently produced by cells at the time of wounding, and to modulate the ability for head or tail probabilities to change.*C*_*wound*_ was described by a simple pulse function (Eq 8), with *t* representing time after wounding and *g* = 12 hrs and *t*_*f*_ = 90 hrs. The biological identity of the *C*_*wound*_ substance may be a factor such as reactive oxygen species (ROS), which is strongly produced by wounding and has been previously found to be required for planaria regeneration and remodeling to proceed [75, 76]. These parameter values were chosen based on experimental observations that pharmacological interventions applied 72 hrs after amputation had no effect on regeneration.

The probability of morphological regenerates of a particular fragment were then computed in terms of the probabilities of head, tail or regenerative failure at each cut line of the fragment (Fig 3D). An existing, non-wounded head or tail on a fragment was assumed to be terminally differentiated tissue that would not change during the regeneration process, and these head or tail endpoints of the original body were assigned probabilities of 100% head and 100% tail outcomes, respectively (Fig 3C). The above-described Markov Model was used to estimate frequencies of heteromorphoses expected in a particular fragment appearing in a population of regenerates by multiplying the probabilities of each possible outcome at each cut line of a fragment (Fig 3D). The respective predicted fraction of heteromorphoses appearing in the population were inferred from the probabilities of combinations at both cut-lines of a fragment, where the possible heteromorphoses and their probabilities are shown in (Fig 3E). The Markov Model, including identification of fragments after cutting, identification of cells at the wound, calculation of probabilities at the wound, and calculation of heteromorphoses probabilities in each fragments, was performed in an automated fashion in PLIMBO.

### Planaria colony care

Planaria of the species *Dugesia japonica* were used in all experiments. Planaria were maintained as described in [77]. Animals were kept at 13 °C in Poland Spring water with weekly feedings of calf liver paste and twice-weekly cleanings. Animals were starved for at least 7 days before experiments were performed.

### Two-headed planaria generation

Planaria were cut as described in Nogi et al [78], using a scalpel on moist and cooled layers of filter paper. 2H worms were generated via 3 day incubation of cut fragments in 127 *μ*M octanol as described in [79, 80]. A 2H worm was characterized by at least one eye on each end of the worm [79].

### Amputation and regeneration experiments

A variety of cutting/amputation scenarios were tested, each of which were designed to cut the proposed neural transport map in key geometries to influence outcomes according to the main hypothesis.

In order to explore the role of proposed VNC cord polarity in directing regeneration outcomes, a nerve-deviation experiment inspired by the work of Kiortsis in Mediterranean Fanworm (*Spirographis spallanzanii*) [81], was designed. Trunk fragments of planaria were excised and immediately further cut using upwards or inverted L-shaped incisions. Specifically, fragments were cut perpendicular to the head-tail axis halfway from margin to midline of the worm and further cut along the midline towards either the head (upwards) or tail (inverted), causing tissue movement exposing the VNC in a forward- or reverse- polarity, with N = 43 and N = 46 replicates in upwards and inverted L-cut groups, respectively. The cuts were re-enforced every day for 7 days while worms regenerated at 20 °C, to prevent simple wound healing. All worms were monitored once a day for 14 days, with specimens reserved from each group for synapsin staining every other day for 11 days. Computational modeling using the same upwards and inverted L-shaped incisions on planaria models was performed to obtain predictions for these cutting scenarios. Control L-cuts were made similar to the L-cuts above, but not cutting so far from the margin to the midpoint as to including the VNC, thereby creating an exposed side area free of VNC but attached at the base to the main worm, with N = 30.

To explore the role of net nerve alignment in fragments, rectangular fragments with the ~1 mm long-edge corresponding to the original anterior-posterior worm axis (thereby allowing for identification of axis-polarity in the regenerates) were cut to include the ventral nerve cord (VNC-containing) or from the side margin of the worm, excluding the VNC (VNC-free), with N = 94 and N = 103 replicates in the VNC-free and VNC-containing groups, respectively. The fragments were cut from the margin of 1H worms, with the worm placed ventral side up, which allowed the VNC to be seen and to thereby be avoided in the VNC-free fragments. The fragments were checked 1 day after cutting to ensure that all 4 sides were cut. Fragments that did not fulfill this condition were discarded. All fragments were monitored once a day for 14 days, with additional specimens from each group reserved for fixation and synapsin staining at days 1 through 7, 10 and 14. The net orientation of the synapsin signal was determined using the Directionality plugin in ImageJ. Specifically confocal stacks of the whole fragments with a z-resolution of 2 *μ*m were z-stacked using the average intensity projection in ImageJ and oriented so that the long edge of the fragment was vertical in the images. Fragments in which no long edge could be determined were not analysed. The Directionality plugin was run using the Fourier component method, 90 bins and a histogram start at 0. Resulting data was averaged for all fragments, binned into 10° sections and plotted using Excel. Brightfield images and respective synapsin stains taken over multiple days and showing further replicates, can be found in S1 and S2 Datasets.

To explore regeneration outcomes for different cutting scenarios in two-headed worms, 2H worms were generated as described above, and had completed regeneration at least 1 month prior to subsequent amputation. The 2H worms were cut using either decapitation, or cuts through the central symmetry line of the worm, cuts along the diagonal of the worm [80], or a combination of transverse cuts producing fragments. The number of replicates varied in each cutting scenario, with N = 401 used for short fragments containing a head, N = 90 for medium fragments with head and second head amputation cut not crossing the central symmetry line, N = 140 for fragments with one head and second amputation crossing the central symmetry line, N = 90 for dual head amputation containing the central symmetry line, and N = 41 for fragments with dual head amputation and not containing the central symmetry line. Diagonal cuts of 2H worms were performed through the midpoint of the worm (as estimated by the pharyngial opening) and the base of the two heads (N = 97). Cut fragments were allowed to regenerate for 1 week at 20°C and 1 week at 13°C (to prevent fissioning) before scoring regenerative outcomes.

### Fixation and immunohistochemistry

Worms were fixed in Carnoy’s fixative as described in [78], were bleached in methanol, and were subsequently stained using the mouse-anti-synapsin primary antibody 3C11 at 1:50 dilution. 3C11 (anti SYNORF1) was deposited to the DSHB by [82]. A standard immunohistochemistry protocol was used as outlined in [78], using a goat-anti-mouse-Alexa488 secondary antibody (Invitrogen) at 1:250. Samples were mounted using VetraShield (Vector Laboratories) and imaged on a Nikon AZ100 Multizoom Macroscope or on a Leica SP8.

### *β*-Catenin Interference RNA

The dsRNA synthesis was performed as described in [83]. The template used was *Dugesia japonica* mRNA for *β*-catenin-B, partial cds (GenBank: AB499795). Each dsRNA injection was performed in the pre-pharyngeal area of 1~1.5 cm long worms, consisting of three pulses of 32.2 nL each, for three consecutive days. In order to achieve only a partial block of *β*-Cat, which was required to test the model prediction, the amputation was performed on the third day of dsRNA injection, at least 6 hours after the injection. Regeneration was at 13°C. As a control, VenusGFP dsRNA was injected, and displayed no phenotype different from uninjected worms. N = 20 replicates were used for experimental and control groups, respectively.

### Pharmacological treatments

Dynein transport function was inhibited by pre-soaking intact animals in 3 *μ*M Ciliobrevin D (Sigma, stock 25 mM in ethanol) for 3 days before cutting and placing trunk, pharynx or pre-tail fragments into fresh 3 *μ*M Ciliobrevin D solution for 3 days before removing the inhibitor and replacing with Poland Spring water. Regeneration was at 20°C and outcomes were scored after 14 days. N = 47 replicates were performed as well as N = 50 controls with matching concentration of ethanol.

For all other pharmacological treatments, excised pre-tail fragments were treated immediately after cutting for the first three days of regeneration, after which the regenerating fragments were washed in Poland Spring Water and allowed to regenerate for 10 days at 20 °C before scoring regenerative outcomes. All pharmacological stock solutions were aliquoted and stored at -20 °C. Bromocriptine (Tocris 0427) 50 mM stock solution in DMSO was used at a final concentration of 2 *μ*M, MDL12330A (Tocris 1436) 50 mM DMSO stock solution was diluted to 20 *μ*M, U0126 (Tocris 1144) was used at a concentration of 18 *μ*M, and 3-isobutyl-1-methylxanthine (IBMX) (Sigma I5879) was dissolved in DMSO and used at a concentration of 200 *μ*M. A referenced summary of projected downstream targets for these pharmacological agents is given in Table 3. Bromocriptine treatment was also used on N = 52 worms cut into 5 equal fragments each.

**Table 3:** Pharmacological agents explored in this work, including their dose, targets, replicates, relevant references, and effects on regenerated planarian morphology.

Agent	Dose	Targets	Replicates	References	Regenerates (%)		
					0H	1H	2H
MDL12330A	20 *μ*M	adenyl cyclase inhibitor (IC50 6 *μ*M)	33	[72]	0	84	16
U0126	18 *μ*M	ERK inhibitor (IC50 60-70 nM)	102	[84]	99	1	0
Bromocriptine	2 *μ*M	5HT7R-like inhibition (IC50 1.8 *μ*M)	81	[42]	0	10	90
		D2R agonist (EC50 2.6 nM)		[73]			
IBMX	200 *μ*M	increases cAMP and cGMP (IC50 50 μM)	30	[71]	20	80	0

### Planaria cilia flow visualization and analysis

To detect the pattern of cilia driven flow, an experimental procedure similar to [56] was used. Planaria were placed ventral side up in a small dish so that they attached to the surface of the water and a small amount of Carmine powder (Sigma) was sprinkled onto their ventral side. The movement of the powder particles was recorded on a Nikon AZ 100M microscope with an Andor DL-604M camera at 100 ms frame rate for 2 mins at a time. Slime and powder build-up was periodically removed. The functionality of the method was controlled by applying the same assay to worms treated with 3% ethanol to block cilia function [85] and worms cooled to 4°C, both of which showed a complete lack of cilia-driven powder movement. To analyze the flow pattern, recordings of 1.5 to 2 s were selected in which the worm showed no muscle-driven movement and analyzed using the PIVlab application [86, 87] in MATLAB (MathWorks). Particle image velocimetry (PIV) was calculated in an interrogation area of 64 x 48 x 32 pixels for subsequent passes for all frames, data was smoothed, and all frames were averaged to result in the shown flow pattern.

Ciliary beat was used as an indicator of cellular polarity, as the establishment of long-range order by physiological mechanisms is a key implication of our model. As a polarized microtubule array within individual cells is a required prerequisite for establishment of planar cell polarity [88–92], and cilia beat direction is in turn controlled by planar cell polarity [93], the beat direction of planaria cilia are read-outs of a net microtubule array alignment in individual cells, and ciliary beat direction is widely used as a readout of planar polarization in tissues [40, 94–99].

## Results

### Patterning outcomes are explained by spatialized, quantitative modeling of molecular regulatory network dynamics

It is crucial to develop tissue simulations of proposed signaling networks to fully test quantitative models of regeneration, ensuring that they produce the correct outcomes for known functional data in the field. Additionally, an existing gap in understanding planarian regeneration is how to describe self-regenerating morphogen gradients independent of size-scale, while maintaining adherence to realistic time-scales and conditions. Using realistic values for biological parameters (see S1 Text) and known regulatory interactions (e.g. the canonical Wnt/*β*-Cat pathway), we developed a model to address these knowledge gaps, and tested its predictive abilities by simulating diverse experimental conditions and comparing the outcomes, either against published results, or new experimental data, in a quantitatively rigorous manner. The main predictions of the full model, and their state of validation by previously-reported or presently-reported experimental evidence, are summarized in Table 2.

The core of the model is a regulatory network including major molecular signals known to be involved in regulation of anterior-posterior axis regeneration in planaria, namely Wnt, *β*-Cat, ERK, Notum, and Hh (see Table 1). Predicted morphogen signaling gradients were found to be consistent with experimentally-observed planaria body-plan outcomes for homeostasis (Fig 2A) and normal wild-type regeneration (Fig 2A‘*o*’).

We first tested the model’s ability to correctly predict outcomes of experimental manipulations known to alter regenerative outcomes in planaria by simulating 10 different RNAi and pharmacological interventions as perturbations to key components of the regulatory network (Fig 2). Specifically, we modeled RNAi against Wnt, Hh, *β*-Cat, APC, and Notum, as well as chemical manipulations of ERK, cAMP, dopamine, and serotonin (Fig 2 and Tables 1 and 3). Of these 10 different interventions, cAMP inhibition by MDL12330A and cAMP enhancement by the phosphodiesterase inhibitor IBMX have not been previously reported. Overall, the model generated stable morphogen concentration patterns, which were predictive of body-plan outcomes observed in experiments (Fig 2) for interventions associated with both abnormal posterior regeneration (e.g. head on the posterior to regenerate 2H, Fig 2A*i* to 2A*iii*), abnormal anterior regeneration (e.g. loss of head or tail on the anterior, Fig 2A*v* to 2A*x*), or loss of tail (Fig 2A*iv*). These model predictions, where regenerated body-plan is interpreted in terms of stable morphogen gradients after cutting the model, matched corresponding experimental outcomes, which we compiled from published work from a number of different labs, as well as from new experiments (Table 1 and 3). Details of predicted morphogen gradients and morphological outcomes for all interventions considered in the model are summarized in S3 Fig.

**Fig 4:**
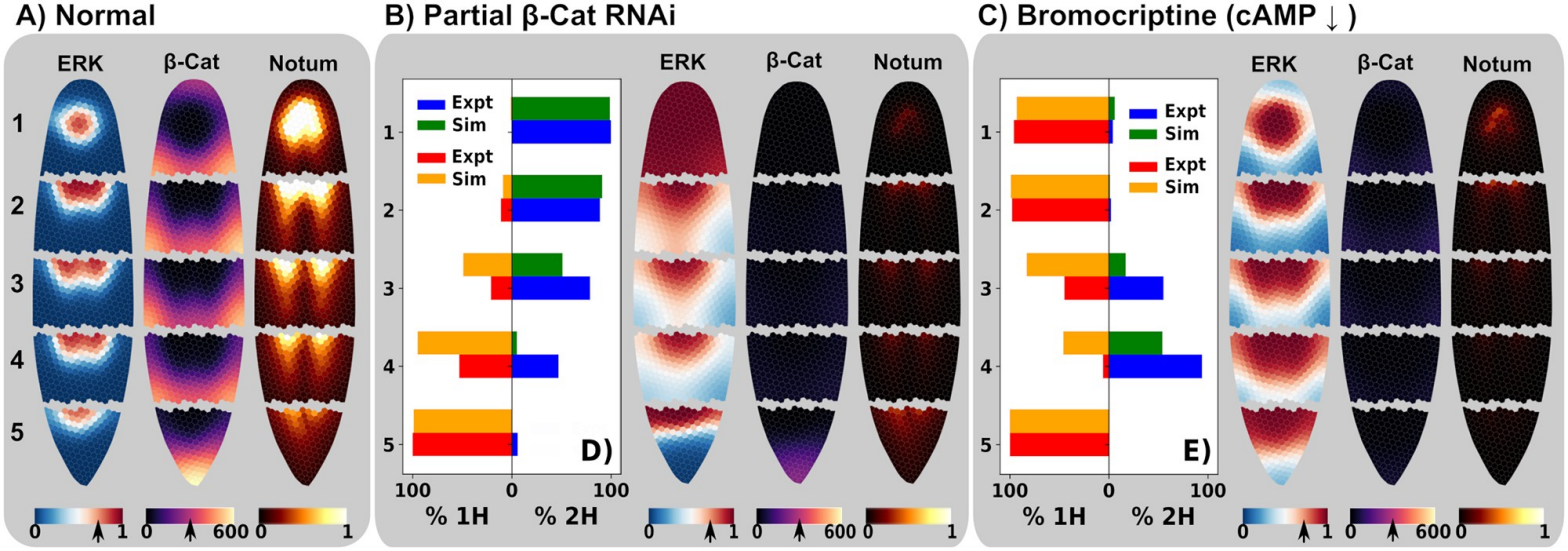
Model predictions and experimental regenerative outcomes show matching trends for different fragments along the anterior-posterior axis under different cases. For normal wild-type regeneration, the model predicts polarized gradients of ERK, *β*-Cat and Notum for each of the 5 cut fragments (A), which are predicted and found to regenerate as 100% 1H in each of the 5 cut fragments (0% 2H heteromorphoses). Partial (i.e. incompletely blocked) RNAi of *β*-Cat is predicted to show more morphogen gradient polarity disruption, and therefore more 2H regenerates, in fragments progressively closer to the head (B), a prediction that parallels experimental observations (D). In contrast, model perturbations inhibiting *β*-Cat stability (e.g. via decreased cAMP with bromocriptine treatment) are predicted to show more morphogen polarity disruption (C), and therefore, a higher frequency of 2H regenerates, in fragments cut closer to the tail (E). These modeling prediction trends are corroborated by experiment (D, E). Note that experimentally, the tail piece was only cut on one side and can consequently only regenerate as a 1H, as it only has one blastema.

The model also explains puzzling observations that have been reported experimentally with regards to Notum [32, 34]. In the model, Notum is transcribed at anterior-facing wounds (S4 Fig) and therefore appears as an anterior-polarized gradient under normal regeneration conditions, as is observed experimentally [28, 29, 32] (Fig 4A). Consistent with this anterior localization, experimental RNAi data shows that Notum is required for head induction [32], which our model reflects as a loss of anterior signals following simulated RNAi against Notum (Fig 2Aix). Paradoxically, the same experimental data [32] shows loss of Notum signal in 2H heteromorphoses, which is also recapitulated in our model (Fig 4B and 4C). Conversely, treatments leading to loss of anterior development (e.g. APC RNAi, S3L Fig) are predicted by the model to show very high Notum levels in spite of producing 0H regenerates—this is further consistent with the previously reported data [34].

The model provides insight into these otherwise paradoxical observations with respect to Notum, showing how they occur due to a confluence of four factors (Fig 1E): (1) expression of Notum is induced by *β*-Cat (indirectly via *β*-Cat’s induction of expression of a predicted Notum Regulating Factor (NRF), where NRF induces Notum expression); (2) NRF is subjected to vector transport by dynein (towards the anterior of fragments, and in the opposite direction to transport of Hh); (3) Notum inactivates Wnt at the anterior of regenerating fragments; and (4) *β*-Cat is ultimately what inhibits ERK signaling and therefore anterior regeneration. Taken together, while Notum is required to decrease levels of Wnt [32, 100, 101], and thereby induce anterior regeneration under normal circumstances, interventions that are downstream of Wnt signaling will show non-intuitive outcomes with respect to Notum. Manipulations leading to enhanced destruction of *β*-Cat (including decreases in Wnt and cAMP levels), decrease *β*-Cat, and therefore increase ERK and anterior regeneration signaling, while reducing levels of Notum. In contrast, as APC (and other elements of the *β*-Cat destruction complex) signal downstream of Wnt and Notum, RNAi to APC leads to significant increases in *β*-Cat levels and therefore to a strong inhibition of head formation with concurrent strong increases in Notum levels due to stimulated expression by increased *β*-Cat. Finally, the model correctly predicts that Notum works in conjunction with Patched (Ptc) to inhibit *β*-Cat signaling, such that inhibition of Notum or Ptc both lead to 0H or 2T heteromorphoses [39, 40] (Fig 2A*viii* and 2A*ix*). Please see S3 Fig, which presents modeling results illustrating these trends.

### Position along the head-tail axis determines regenerative outcome following molecular interventions

Due to the spatialized nature of our model and the ability to flexibly simulate different physical perturbations, the model can predict regenerative outcomes for fragments of different sizes, shapes and locations within the original animal. This enabled us to extract predictions of differential regenerative outcomes for fragments along the anterior-posterior axis (Fig 4). In the standard case, the model predicts polarized gradients of ERK, *β*-Cat, and Notum consistent with normal regeneration of single-headed worms in each fragment (Fig 4A). For a partial RNAi block of *β*-Cat, such that *β*-Cat is no longer being synthesized but levels have not fully decayed in the system, the model predicts higher levels of morphogen gradient polarity disruption for more anterior fragments (Fig 4B and 4D), which translates to a prediction of higher numbers of 2H regenerates closer to the head. This prediction fundamentally depends on the premise that Hh is synthesized and transported in neural tissue. To test this prediction, we performed a novel investigation of the effects of partial RNAi against *β*-Cat in changing the incidence of regenerative outcomes of worms cut into 5 fragments along the head-tail axis immediately after 3 consecutive days of RNAi injections. Our experimental observations show the same trend as the prediction of the model, with 100% 2H organisms regenerating from head fragments, and progressively lower incidence of 2H heteromorphoses forming in fragments cut closer to the tail (N = 20), see Fig 4B and 4D.

In contrast to the observation of high 2H regeneration frequencies in more anterior fragments for a partial *β*-Cat RNAi, the model predicts the reverse pattern for perturbations inhibiting cAMP (or any perturbation enhancing degradation of *β*-Cat in the canonical Wnt/*β*-Cat signaling pathway) where a higher level of morphogen polarity disruption, and therefore more 2H incidence in a population of regenerates, is predicted in fragments cut closer to the tail (Fig 4C and 4E). We tested this prediction by chemically inhibiting cAMP signaling through the application of Bromocriptine [25, 42], and did indeed observe the highest number of 2H in the pre-tail fragment, with very low 2H numbers in the pre-pharynx and head fragments (Fig 4C and 4E), with the experimental results showing the same trend as model predictions.

Note that these contrasting trends are not limited to a small sub-set of parameters, but appear to be a natural component of the planaria model, which can also easily be seen in the simpler 1D simulations (see S2C and S2D Fig).

In summary, these results indicate that our proposed model is capable of recapturing and accurately reflecting past and novel experimental data, as well as offering explanations for previously puzzling observations. At the same time, the model can make new, non-obvious predictions for the outcomes of genetic and chemical manipulations, especially in combination with complex physical manipulations, some of which we validated here (see Table 2).

### Target morphology is encoded by vector transport fields related to neural architecture

In addition to the signaling network within the individual cell, our model relies on vector transport of certain signaling factors, specifically Hh and NRF, which were identified as necessary in order to create a model capable of describing outcomes of RNAi experiments and the variety of new experiments reported herein (see Table 2). While the vector transport aspect of the model is compatible with several mechanisms of directional transport involving an array of potential cell types, including transcytosis [102], cytonemes [103], and electrophoretic transfer of small, charged morphogens via gap junctions [48, 104], experimental data presented here suggests a role for the nervous system anatomy of planaria in directing regeneration outcomes. We therefore proposed that the directional transport of morphogenic factors occurs with respect to aligned microtubules of the neuronal axons [105–107], where vesicles of Hh are hypothesized to be transported by the motor protein kinesin, while the here-proposed Notum Regulating Factor (NRF) is hypothesized to be moved by dynein. Neuronal transport or expression of these factors has previously been shown for Hh [30, 31, 39]. To test the importance of cellular motor protein activity for the patterning of the AP axis, we inhibited dynein-based transport through the application of low doses of Ciliobrevin D to regenerating fragments, which did not impact ciliary motion as assessed by gliding behavior. Blocking dynein-based transport led to inhibition of head formation during regeneration (N = 23/47 0H), consistent with the phenotype observed with Notum RNAi (see S3I, S3K and S5 Figs). Cell division was not disrupted by Ciliobrevin D treatment, as the tail blastema regenerated at a normal rate. Although we cannot rule out the possibility that other morphogen-regulating factors are also being transported by dynein, the consistency between the dynein inhibition and Notum RNAi supports the hypothesis that NRF is being transported via dynein towards the anterior blastema where we predict it is responsible for inducing Notum expression and subsequent head formation.

**Fig 5:**
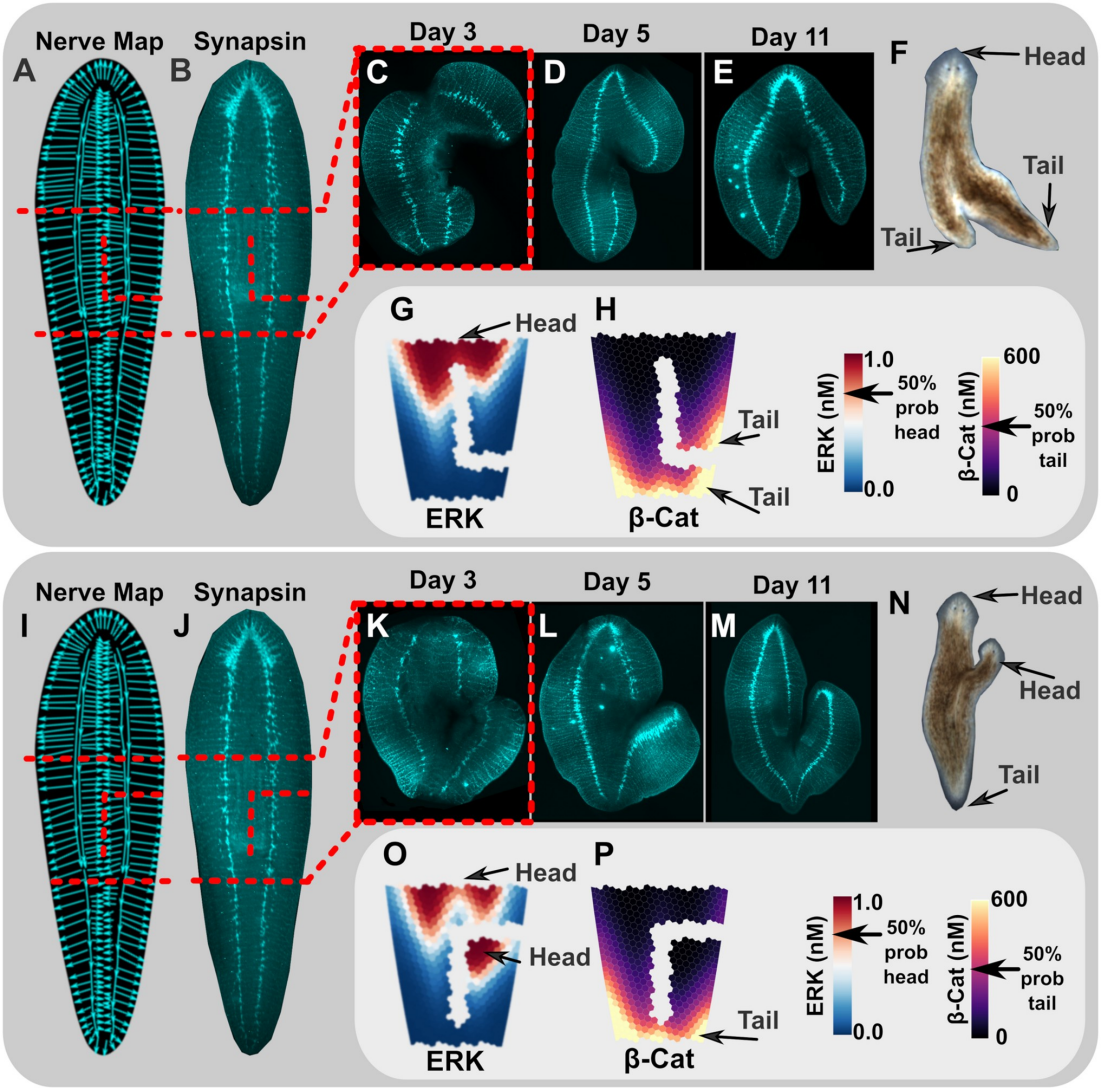
Unique cutting arrangements of 1H worms highlight the key role ventral nerve cords (VNC) play in directing regeneration outcomes. Excised trunk fragments of planaria were cut with an additional incision to generate a long segment containing a VNC with backwards polarity (cuts shown as red dotted line on traced nerve map in A, and on synapsin stain in B), or with an inverted incision of the same pattern to generate a long, free segment containing a VNC with forwards polarity (red dotted line on traced nerve map in I, and on synapsin stain in J). Modeling-predicted morphogen gradients were consistent with the formation of a tail on the long, free segment for the backward-polarity VNC incision pattern (G, H), and head formation on the long, free segment for the forwards-polarity VNC cutting pattern (O, P). These predictions were a direct match to actual regeneration outcomes (N = 80/80). The backwards-polarity VNC (synapsin stains in C, D, E, and final body-plan in F) showed regeneration of a tail on the long, free segment, as predicted. The forwards-polarity VNC (synapsin stains in K, L, M, and final body-plan in N), showed regeneration of a head on the long, free segment, as predicted.

**Fig 6:**
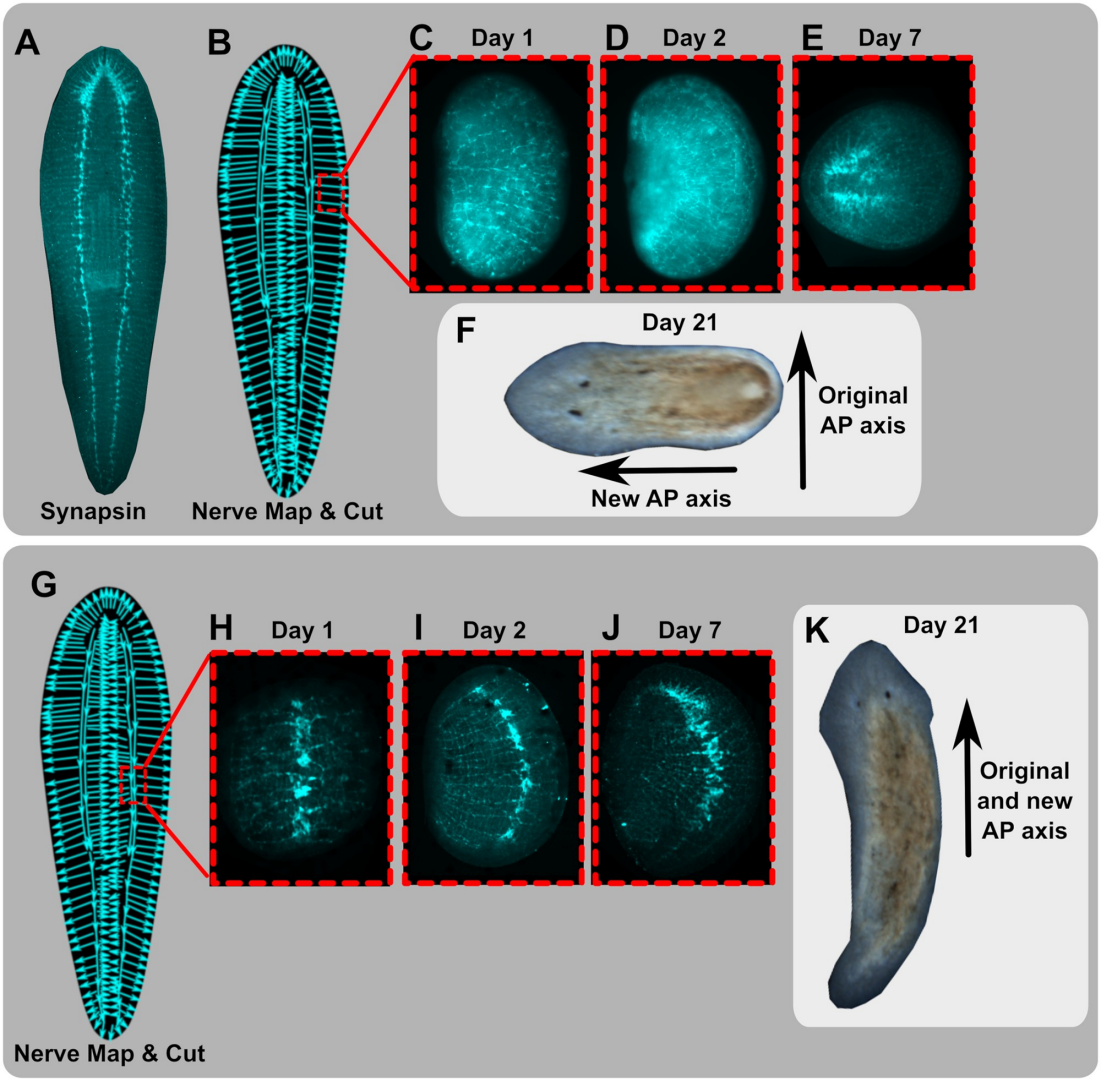
The net nerve alignment in a fragment determines regenerated anterior-posterior axis, where fragments with initial axon orientation perpendicular to the original axis show 90° rotation of the regenerated anterior-posterior axis. Synapsin stains of whole 1H worms (A) and hypothesized nerve polarity map traces with location of rectangular fragment cut regions are shown (B, G). The aspect ratio of all fragments was cut such that the longest side of the rectangular fragment corresponded with the original anterior-posterior axis. Rectangular fragments cut from the side margin of worms (B, with time-course of synapsin stains of fragments shown in C, D, and E) regenerated with anterior-posterior axis oriented perpendicular to the original axis (F), consistent with the fragment’s proposed net nerve polarity (B and C), an outcome which was observed in N = 94/94 replicates. In contrast, fragments of the same size and shape cut to include a portion of the ventral nerve cord (G, with time-course shown in H, I and J) regenerated as 1H worms with new axis corresponding to that of the original worm (K), an outcome observed in N = 103/103 replicates.

**Fig 7:**
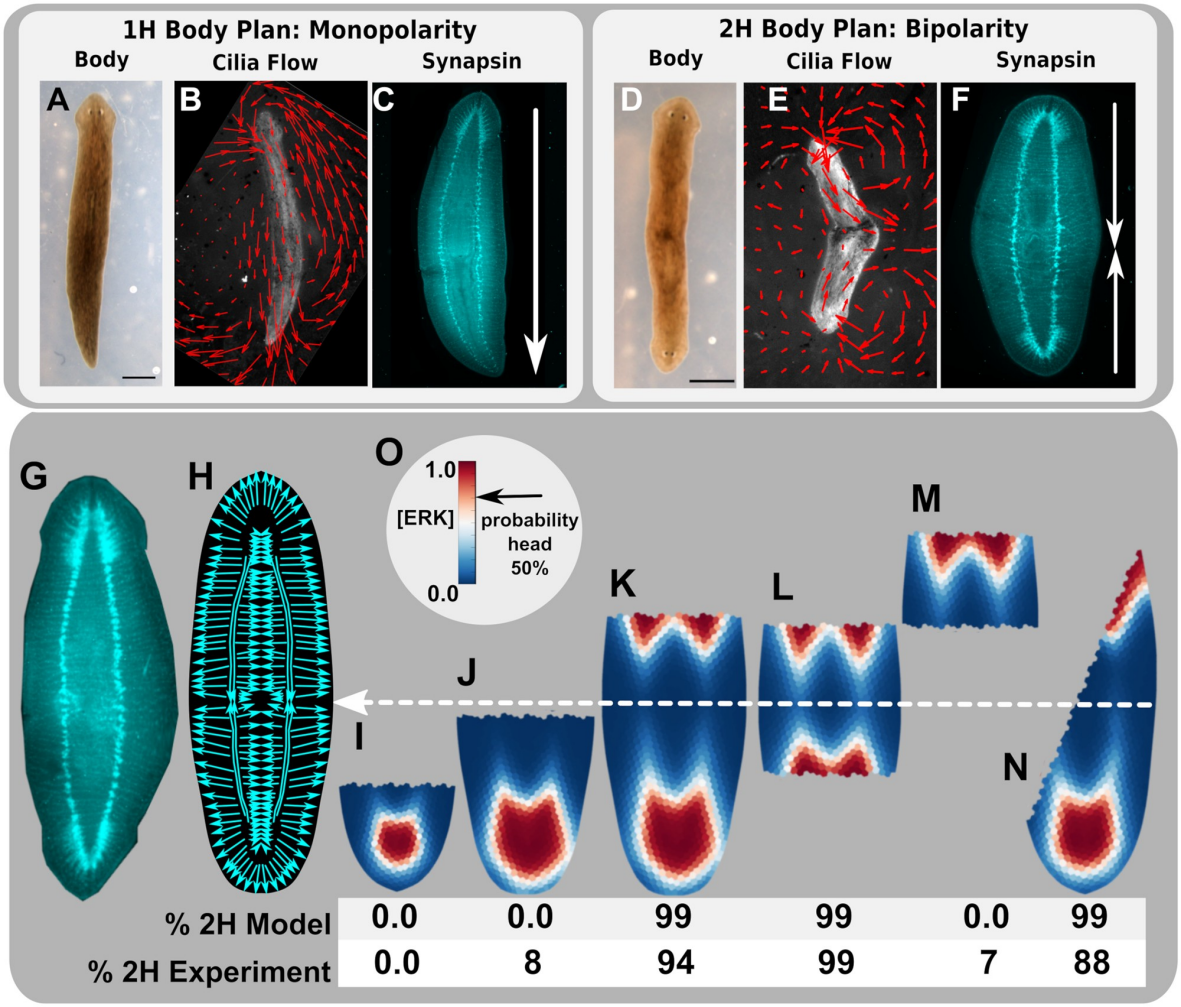
Model-predicted morphogen gradients are consistent with experimentally-observed regeneration outcomes for a variety of cutting scenarios of 2H worms, supporting the vector transport hypothesis. Planaria heteromophoses show organism-level polarities consistent with head number. In 1H worms (A), cilia flow vector fields (B) occur as monopolar gradients with flow directed away from the single head (B, C). In 2H worms (D) bipolar cilia flow fields were observed (E), which exhibit coordinated beat movement away from each of the two heads (F). From the bipolar cilia beat in 2H worms, we hypothesize a bipolar nervous system, where example synapsin stains of a whole 2H worm (F, G) and nerve polarity map trace (H) are shown. The white dotted line shows location of proposed line of symmetry at the midpoint between the two pharnyx for the bipolar 2H transport gradient (H), which is consistent with the observed location of cilia beat flow pattern symmetry in 2H worms. Panels I, J, K, L, M, and N show model-predicted steady-state ERK concentrations, where higher ERK levels are probabilistically associated with head outcomes (as shown in O). Cutting 2H worms showed experimental regeneration outcomes consistent with model predictions for a variety of cutting scenarios (I, J, K, L, M, N). A Chi^2^ test was used to compare model and experimental frequencies which showed corroboration between experiment and the model with p < 0.05.

As vector transport of morphogens is proposed to be determined by nerve axon polarity, and morphogen concentration is instructive for regenerated body-plan, we predicted that the average (net) polarity of nerves contained in a cut fragment would drive the patterning of the primary axis in head/tail regeneration. We sought to test this prediction by analyzing regeneration outcomes in a variety of different cutting scenarios involving the monopolar and bipolar nerve polarity maps of 1H and 2H heteromorphoses (Figs 5, 6 and 7).

The high nerve axon density and strong anterior-posterior alignment of axons in the VNC [57] are predicted to lead to VNC dominance of the transport direction in fragments containing the VNC (Fig 5). Therefore, when a 1H worm is cut perpendicular to its head-tail axis, transport along the VNC is predicted to lead to re-establishment of morphogen gradients with their original anterior-posterior polarity in each cut fragment (Fig 1G and 1F), a prediction that has been previously observed in experiments [29, 34]. To further test the involvement of VNC, L-shaped cuts were made into the margins of 1H worm trunk fragments, such that the VNC was diverted towards the induced third blastema via cutting, which induced a side-outgrowth containing either a forward-facing or a backward-facing VNC (Fig 5). Dependent on this directionality, the side outgrowths formed either a head when the forward-facing VNC was diverted (N = 46/46), or a tail when the backward-facing VNC was diverted outwards (N = 43/43), see Fig 5. When the L-shaped cuts were performed so that only tissue outside of the VNC was diverted towards a side outgrowth, no head regeneration was observed (N = 25/25), see S6 Fig. Taken together this data indicates that the VNC is important in generating head or tail regenerative outcomes, depending on cut orientation, and that other tissues, such as muscle, which are known to play an important role in patterning in planaria [18, 20], are not alone sufficient to determine new head/tail identity in tissues. These data further indicate that directional transport on the VNC has the ability to determine head/tail identity, as predicted by the steady-state morphogen gradients of ERK and *β*-Cat produced by the model for these same two cutting scenarios (Fig 5G, 5H, 5O and 5P).

To be able to functionally test the hypothesis that transport along the vector field formed by the net distribution of nerve axons distributed throughout the planarian tissue is the driver of subsequent patterning events, we sought a test case involving a cut fragment where the average nerve direction was not aligned with the prior head-tail axis and the gradient of morphogens that existed before the injury. Specifically we tested small fragments cut from the margins of the worm between the VNC and the body edge, where synapsin stains indicated a net nerve polarity oriented 90° to the anterior-posterior axis and thereby perpendicular to the original morphogen gradient (Fig 6B and 6C). The regeneration of rectangular fragments cut from this margin area (with cuts on all 4 sides so as to not bias blastema formation, and the long edge of the rectangle corresponding to the original anterior-posterior axis), was tracked over time, as well as observed in detail via a time course of synapsin staining. This revealed that the newly forming head in these fragments developed perpendicular to the original body axis and morphogen gradient and in alignment with the nerve fiber direction (Fig 6C–6F), exactly as predicted by the model (N = 94/94 regenerated 1H). These side pieces very likely contain nerve bodies in addition to the axons oriented 90° to the anterior-posterior axis, as seen by PC2 staining [63] and persistent synapsin staining over multiple days. These nerve bodies are hypothesized, by our model, to be the site of production of Hh and NRF, which are transported along the axons to the long edge of the fragments, perpendicular to the previous axis. In contrast, control fragments of the same size and shape, but containing a piece of the VNC, regenerated following the orientation of the VNC and in accordance with the head-tail orientation of the original tissue (N = 103/103 regenerated 1H), see Fig 6G–6K. Further depictions and quantification of nerve directionality in regenerating fragments for the VNC-free and VNC-containing fragments are shown in S7 Fig, as well as the S1 Dataset. This confirms a highly non-intuitive prediction of our model, given that it is, to our knowledge, the first case where regenerative polarity of a fragment is fundamentally different from that of the original tissue without any applied treatment. This indicates that the morphogen gradient expressed in planaria tissue for various position control genes (PCGs) is not deterministic for regenerative outcomes but rather is set up by the underlying neuronal polarity which determines patterning.

The 2H worms feature a non-trivial nerve polarity map (Fig 7G and 7H), which is proposed to exhibit bipolarity associated with each head and the location of the midpoint of the animal (Fig 7, where white arrow shows midpoint). This bipolarity was directly observable using cilia flow assay (Fig 7A–7F and S5 and S6 Videos). Cutting 2H worms with respect to the nerve polarity map lead to experimental regeneration outcomes consistent with modeled morphogen gradients resulting from vector transport on the field derived from nerve polarity for a variety of cutting scenarios (Fig 7I–7N). Experiments showed 0% 2H (0/401) for short fragments containing the head (Fig 7I), 8% 2H (11/140) for longer fragments with head but posterior cut not crossing the symmetry line (Fig 7J), 94% 2H (85/90) for cuts containing the head but posterior cut passing the symmetry line (Fig 7K), 99% 2H (89/90) for amputations of both heads in fragments containing the symmetry line (Fig 7L), and 7% 2H (3/41) for amputation of both heads in fragments that did not contain the symmetry line (Fig 7M). Cutting 2H worms diagonally at angles greater than 45° from the symmetry line regenerated 88% 2H (85/97) in fragments that showed regeneration (Fig 7N). Experimental regeneration outcomes showed statistically significant corroboration with model-predicted outcomes (based on Markov-model body-plan inference from predicted morphogen gradients) for the same fragments (Chi^2^ test, p < 0.05). This modeling and experimental data in 2H worms further indicates that transport of morphogens occurs with respect to nervous system polarity, and demonstrates further correct predictions of a wide range of non-intuitive regeneration outcomes.

### Morphogen gradient scaling

Models were run with planarian body shapes ranging from 0.3 cm to 2.4 cm in length, and were found to produce morphogen gradients with similar pattern form and of similar magnitude in models of very different size (S8 Fig). Moreover, cutting these models into 3 fragments showed that all models, except for the smallest, were able to fully re-polarize morphogen gradients (S8 Fig). Small fragments (below approximately 1 mm in length) begin failing to regenerate normal gradient polarities, with 0T and 2H heteromorphoses predicted as fragments become smaller (S8 Fig), an outcome which is experimentally observed for small or thin fragments [108–110].

Taken together, the results of the above-described analyses and new experiments show that our model correctly predicts the outcomes of a range of diverse amputations that probe the relationship between positional information in the intact worm and mechanistic transport processes that set the axial polarity of the resulting fragments. We conclude that regenerated anterior-posterior axial polarity depends on the average polarity of nerve axons contained in a regenerating fragment, and that these are dominant drivers in cases where their direction diverges from the pre-existing directionality of the prior AP axis.

## Discussion

Despite major advances in understanding cellular and molecular mechanisms required for regeneration and patterning, achieving a deep understanding of underlying large-scale pattern control during development and regeneration remains an outstanding challenge, crucial to achieving the goals of biomedical regeneration and the mitigation of birth defects. Here we utilize a tight integration between computational modeling and experiment to develop a context for understanding fundamental anterior-posterior body-plan control mechanisms in regenerating planaria, where instructive cues are ultimately described in terms of molecular positional information patterns. We present a comprehensive, spatialized model that correctly predicts and explains existing data, and also makes a number of surprising, novel predictions that were confirmed by experiments.

This work provides evidence that augmenting reaction-diffusion mechanisms with vector transport on hypothesized, though realistic, nerve polarity maps improves on limitations of reaction-diffusion models in a manner that is consistent with a variety of experimental observations for planaria. While reaction-diffusion mechanisms can elegantly generate various pre-patterns of molecular information to account for self-assembly of morphogens in a variety of systems [6, 17, 111], their patterns are often dependent on the size-scale of a tissue, and are therefore not sufficient for use in regenerating planaria [48, 49]. While scale-free models have been developed to address the limitations of traditional reaction-diffusion schemes [49], they have been found to have several limitations for planaria, including [48]: (1) having steady-state concentration magnitudes highly dependent on the size of the model, which presents difficulties for downstream biological signaling where consistent concentrations are required in fragments of very different sizes; (2) requiring many weeks to form a gradient for a model sized on the order of an average planaria length (~1 cm), whereas in reality, morphogen gradients reach a stable state at 72 hours or less [28, 30]; (3) being unable to describe gradients for both 1H and 2H worms with the same model [48, 49]; and (4) as gradients of the scale-free model form with respect to the longest length of a fragment, the model may produce non-realistic gradients in cut fragments [48, 49]. Therefore, after extensive assessment of a variety of reaction diffusion models, we concluded that reaction-diffusion in isolation appears unable to appropriately account for self-assembly of accurately instructive morphogen gradients in planaria fragments of a large variety of sizes spanning several orders of magnitude (e.g. 0.05 to 5.0 cm) and contexts (e.g. 2H worm regeneration).

In contrast, vector transport schemes described herein create self-assembling morphogen gradients that are virtually independent of scale, form with respect to the polarity of the transport field rather than with respect to the longest dimension of a fragment, and have relatively consistent concentration magnitudes independent of size [48, 50, 51, 62]. Previous reports have proposed vector transport of neurally-produced Hh on the VNC in an anterior to posterior direction as a key mechanism for maintaining axial polarity in regeneration [30, 39], however, this hypothesis has not been formally explored nor modeled. Our model expands on this original concept, predicting that in addition to Hh, a Notum-inducing substance such as NRF must be subjected to vector transport, and also, that the transport field includes all nerves, not only the VNC.

A foundational hypothesis of our model is that certain morphogens (NRF and Hh) are subjected to vector transport, and that this transport mechanism is crucial to account for the scale-free self-assembly of morphogen gradients directing the body-plan in regeneration (Fig 1). We herein show experiments, both of regenerative outcomes following various cuts, as well as chemical inhibition of dynein transport, that strongly support the concept that vector transport occurs with respect to the planaria nervous system. It is important to note that the vector transport aspect of the model is compatible with several mechanisms of directional transport involving an array of potential cell types, including transcytosis [102], cytonemes (which have been observed to transport both Wnt and Hh) [103, 112–114], and electrophoretic transfer of small, charged morphogens via gap junctions [48, 104]. However, given our experiments have provided multiple lines of evidence for a role of planarian nerve orientation in directing regeneration outcomes, we have highlighted axoplasmic transport, a well-known phenomenon mediated by motor proteins operating on the aligned microtubules of neuronal axons [115], as the most promising candidate for vector transport mechanism underlying planarian regenerative axial control. Importantly, evidence exists that morphogens such as FGF [116] and *β*-Cat [117] are subjected to axoplasmic transport in rat neurons, where morphogens are transported from source to target cells via synapses [118]. For consistency with published experimental observations involving Notum, it was necessary to introduce NRF as a *β*-Cat induced, neurally-transported factor, which in turn induces Notum expression. While the molecular identity of NRF will be explored in future work, one possibility that corresponds with all existing experimental information to date is that NRF is Notum mRNA transported in the nerves, where axoplasmic transport of mRNA is a fairly well-established phenomenon [65, 119–121].

The model is able to account for regeneration outcomes resulting from 10 different, major perturbations to the underlying molecular regulatory network (Fig 2A), and furthermore, makes accurate predictions regarding the expected incidence of heteromorphoses for different fragments cut along the anterior-posterior axis for different manipulations (Fig 4). The model is capable of predicting steady-state morphogen gradients consistent with regeneration outcomes of, not only cuts perpendicular to the anterior-posterior axis of 1H worms and complex cutting scenarios (Fig 5), but also the non-obvious regenerative outcomes of various localized sections of 2H worms (Fig 7). Furthermore, the incidence of 2H outcomes with cAMP inhibition and 0H outcomes with cAMP enhancement (Table 3), corroborates the previously suggested role for cAMP acting downstream of G-protein coupled receptor (GPCR) signaling through an abundantly-expressed 5HT7-like serotonin receptor [42]. This further highlights the role that neurotransmitters such as dopamine and serotonin, signaling through GPCRs, may play in specifying regenerated body-plan axis [25, 42], and suggests a potential additional signaling role for neural activity in the control of regeneration.

Importantly, the model also suggested a test to cleanly distinguish between two hypotheses: control of regenerative polarity by pre-existing morphogen gradients versus by the directionality of net innervation of a fragment. Our new experimental data confirmed the remarkable prediction of 90° rotation of the regenerated anterior-posterior axis in small VNC-free fragments, indicating that average neural axon polarity in a fragment is the dominant driver of anatomical polarity via the directional transport of morphogens with respect to nerve polarity (Fig 6). This fact is not apparent from fragments containing VNC as typically used in most studies, because the large nerves of the VNC dominate transport, thereby leading to morphogen transport and stable gradients aligned with the original anterior-posterior axis. However, in fragments lacking VNC, their axial polarity is dominated by the commisural nerves and lateral branches, which are directed at right angles to the anterior-posterior axis, and their regenerative anatomy corresponds to the neural directionality, not to that of the original anatomical polarity. This is, to our knowledge, the first case were anterior-posterior polarity is predictably changed during regeneration without molecular or chemical manipulations, and therefore presents a strong confirmation of our model and a new avenue in which the re-establishment of polarity can be studied in a new context in planaria.

An additional important current knowledge gap concerns mechanistic explanations for permanent rewriting of target morphology: planaria exposed to brief modulators of physiological state produce 2-headed (bipolar) regenerates, which continue to re-create two-headed anatomy upon future rounds of regeneration, with no further manipulation [79, 80]. The ability to permanently alter the pattern to which the animal regenerates, without genomic editing, is a novel example of large-scale epigenetics and has not been addressed by existing molecular pathway models. Here we show that the existence of a bipolar nervous system determines subsequent regeneration outcomes dependent on the portion of nervous system map cut from the whole: fragments containing significant bipolarity regenerate as 2H, whereas fragments cutting a monopolar portion out of the neural network regenerate with 1H morphology (Fig 7). Thus, we provide further evidence that target morphology outcomes may be encoded by the net polarity of nerves in a regenerating fragment.

These data have implications beyond planarian regeneration. It has long been known that the nervous system is required for proper appendage regeneration [122, 123]. However, a mechanistic explanation for this dependence has not been available; nor was it known whether the influence of the neurons is permissive or instructive for patterning, although experiments in tadpole tails [124], developing frog embryos [125], and non-planarian worms [81] have provided tantalizing suggestions to this effect. Our model proposes a specific mechanism by which neural growth patterns can control large-scale whole body patterning. This work in bilaterian regeneration is consistent with observations in nematode development [126], which suggest that neural control of Wnt signaling profiles is a highly conserved mechanism for body pattern control, conserved among taxa with widely divergent body-plans, developmental modes, and regenerative abilities. More broadly, these data suggest new hypotheses, to be tested in future work, about the evolutionary dynamics of nervous systems, ectodermal polarity pathways, and Hh and Wnt signaling pathways [40].

Ultimately, our model provides a context for understanding target morphology in terms of both an embedded vector transport field (augmenting the positional information concept with that of a directional information flow map) and the complex system established by interacting molecular entities. Within this context, the regenerating organism utilizes the inherent system dynamics to maintain body-plan homeostasis under normal circumstances by maintaining steady-state concentration profiles of various substances, yet naturally detects injury when the cell cluster is damaged as vector transport redistributes morphogens with respect to the new boundaries introduced by the specific injury. After injury, the morphogens achieve a new, similar steady-state to that preceding injury, which instructs the regeneration of missing features. Therefore, the model assists in providing a deeper understanding of mechanisms required to specify single cell activities such as gene expression changes and differentiation which lead to homeostasis and coordination over multiple levels of scale.

### Conclusions

Planaria are an important model for a very fundamental phenomenon: pattern homeostasis, which allows regulative regeneration to restore complex anatomical structures. It is widely acknowledged that the discovery of molecular signaling relationships has far outpaced our understanding of the origin of order and the ability of complex systems to remodel to correct anatomical patterns from diverse starting conditions. Fully quantitative models are an essential step for the field of developmental biology, and are an important emerging tool for rational discovery of interventions in regenerative medicine. The framework presented here is a powerful environment for general use, within which future findings in planarian regeneration and other systems can be predicted and interpreted. The model and simulation environment facilitate the addition of new data in this field to explore self-organizing dynamics that cannot be inferred from arrow diagrams alone, and make novel predictions about the large-scale behavior of molecular pathways. Our model contains a regulatory network which recapitulates a majority of published genetic and pharmacological manipulations, and makes new predictions for regenerative outcomes for a wide variety of experimental manipulations, both genetic, chemical and physical, which we successfully validated here. Furthermore, the modeling of neuronally-mediated vector transport of morphogens was validated in new experiments indicating the importance of dynein-based transport in morphogen localization as well as showing the reorientation of head-tail polarity in fragments, according to their nerve polarity, without any treatment. Even more importantly, our model and its analysis are proposed as a proof-of-principle effort, applicable potentially to a wide range of patterning contexts, as a first step towards an integrated modeling system within which complex, emergent dynamics can be explored.

## Supporting information

S1 Text Supporting information for modeling neural control of body-plan axis specification in regenerating planaria. This document describes additional theoretical and mathematical aspects of the computational model and of the regulatory networks component of the planaria model.
(PDF)

S1 Fig A summary of model results on different planaria body shapes and transport fields developed from experimentally-derived synapsin stains for one- and two-headed planaria. Experimentally-derived synapsin stains (columns A and E) were manually traced (columns B and F) to produce predictions of nerve axon polarity throughout the planarian tissue (i.e. including, but not limited to, the VNC), which were coincident with the morphogen vector transport field, *u*(*x*, *y*). Three replicates were traced for each of 1H and 2H worms, respectively. The divergence of the interpolated vector fields was calculated, normalized, and used as a production gradient *G*(*x*, *y*) (columns C and G) for specific morphogens of the model (Hh and NRF). Columns D and H show model predictions of ERK concentration for a simulated 72 hours after cutting the model. Note that only half of a synapsin stain (non-shaded regions in columns A and E) were traced, with trace reflected about the midline to create a symmetrical model.
(TIF)

S2 Fig A summary of output from initialization, simulation and key model perturbations from the 1D model. (TIF)

S3 Fig A summary of model-predicted steady-state morphogen gradients with predictions of probabilistic regeneration outcomes for a variety of treatment interventions. Panel A shows an untreated worm model cut into 5 pieces, and panels B through L show the results of 11 different interventions tested by the model. All panels show model-predicted steady-state concentrations of ERK, *β*-Cat, and Notum for an initially 1-headed worm cut into 5 pieces, 4.5 simulated days after cutting. The table to the left of each of the 2D morphogen maps summarizes the probabilistic regeneration outcomes predicted for each cut fragment, which were calculated from steady-state model ERK and *β*-Cat concentrations using the Markov Chain Model for Regeneration, described in the manuscript and in Fig 3. Here ‘2T’ = ‘two tailed’, ‘0H’ = ‘headless’, ‘1H’ = ‘one headed, one tailed’, ‘0T’ = ‘tailless’, ‘2H’ = ‘two headed’, and ‘00’ represents no head or tail).
(TIF)

S4 Fig Depiction of typical morphogen gradients arising in untreated 1H worms during initialization and simulation phases, showing all six major morphogens. Note that while factors such as NRF are induced by *β*-Cat, the localization of the NRF does not correspond to the pattern of the inducing agent due to the effect of axoplasmic transport. Therefore, while *β*-Cat is highest at the posterior, NRF appears in a polarized manner at anterior wound edges after cutting. As NRF induces Notum, Notum expression and protein levels are predicted to also appear localized at anterior wound edges, which has been observed experimentally [32].
(TIF)

S5 Fig Inhibition of dynein-driven transport leads to regeneration of headless worms. Brightfield image (A) and synapsin stain (B) of a headless worm regenerated from a trunk fragment following Ciliobrevin D treatment. N = 23/47 regenerated as headless and the remaining as 1H wildtype. Scale bar 300 *μ*m.
(TIF)

S6 Fig Lateral cut of the margin without injury of VNC does not induce head formation to the same extend as similar cut including the VNC. Synapsin stain of a worm after 7 days of regeneration from a fragment with a margin cut without injuring the VNC is shown in (A). At this time, regeneration of the side-piece is not comparable to the case including the VNC (Fig 5). Inset shows magnification of the side-piece showing beginning of putative brain formation, not towards the original head direction, but inwards towards the main animal, consistent with data in Fig 6. Brightfield image of a worm 7 days after regeneration from a fragment with a margin cut without injuring the VNC is shown in (B). Schematic of cut with respect to neural anatomy shown in (C). Outcomes were consistent for N = 25/25. Scale bar shows 500 *μ*m, inset shows 100 *μ*m.
(TIF)

S7 Fig Orientation of nerve fibers determines anterior-posterior axis in regenerating small fragments. Brightfield images of small fragments not containing the VNC as regeneration proceeds on day 1, 3, 5, 7 after cutting are shown in (A). Brightfield images of small fragments containing the VNC as it regenerates are shown in (B). For corresponding synapsin stains see Fig 6. Synapsin stains for later regenerative time points at day 10 and day 14 after cutting of a VNC-free fragments show gradual development of a full brain (C). Synapsin stain for later regenerative time points at day 10 and day 14 after cutting of VNC-containing fragments show gradual development of a full brain (D). Scheme showing orientation of synapsin stain signal as measured by the Directionality plugin in ImageJ, with fibers perpendicular to the long axis of the fragments (non-VNC neurons) having an orientation of 0 and180°, while signal in parallel with the long axis (VNC) has an orientation of 90° (E). The orientation of synapsin signal in fragments at day 1 after cutting for both VNC-free (red) and VNC-containg (blue) fragments, shows peak orientation at 0/180 and 90 degrees, respectively (F). N = 5 samples were analysed for directionality, where error bars show standard deviation. A total of N = 94/94 VNC-free fragments and N = 103/103 VNC-containing fragments regenerated consistently with descriptions. Scale bars show 250 *μ*m.
(TIF)

S8 Fig Modeled morphogen gradients show a high-degree of scale-invariance and remain suitable for axis specification over a wide range of size scales and fragment sizes. Here the simulated ERK concentration is shown in both whole worms (A), and in worms cut into three fragments (B), for planarian body models ranging from 2.5 to 0.5 cm in length. Morphogen gradients develop more detail as the body size increases, but remain able to specify head/tail identity at wounds. As body size decreases, the model predicts that small fragments (below ~1 mm in length) will fail to effectively re-polarize morphogen gradients, and that therefore axis disruptions and heteromorphoses should appear in regenerates from very small fragments, which is a well-known phenomenon [108–110].
(TIF)

S1 Video Animation of 1D Model Output Showing Simulated Dynamics of ERK, *β*-Cat and Notum Concentrations in a whole worm. This video shows an example planaria simulation which self-assembles stable morphogen gradients of ERK, *β*-Cat and Notum in a whole worm for the 1D model.
(MP4)

S2 Video Animation of 1D Model Output Showing Simulated Dynamics of ERK, *β*-Cat and Notum Concentrations after Cutting a Worm. This video shows an example planaria simulation which re-assembles stable morphogen gradients of ERK, *β*-Cat and Notum in each fragment of a whole worm cut into five pieces for the 1D model.
(MP4)

S3 Video Animation of 2D Model Output Showing Simulated Dynamics of ERK, *β*-Cat and Notum Concentrations in a whole worm. This video shows an example planaria simulation which self-assembles stable morphogen gradients of ERK, *β*-Cat and Notum in a whole worm for the 2D model.
(MP4)

S4 Video Animation of 2D Model Output Showing Simulated Dynamics of ERK, *β*-Cat and Notum Concentrations after Cutting a Worm. This video shows an example planaria simulation which re-assembles stable morphogen gradients of ERK, *β*-Cat and Notum in each fragment of a whole worm cut into five pieces for the 2D model.
(MP4)

S5 Video Visualizing cilia beat direction using carmine particles in a 1H worm. This video shows an example cilia flow video of a 1H worm, demonstrating monopolarity of the cilia system. Video is sped up from an original clip of 12 seconds of real-time at a 117 ms frame rate.
(MP4)

S6 Video Visualizing cilia beat direction using carmine particles in a 2H worm. This video shows an example cilia flow video of a 2H worm, demonstrating bipolarity of the cilia system. Video is sped up from an original clip of 10 seconds of real-time at a 80 ms frame rate.
(MP4)

S1 Dataset Zipped Folder of Experimental Images Showing Replicates of small fragment cutting scenarios. This dataset contains brightfield image and corresponding synapsin stains for the VNC-free and VNC-containing small fragment cutting scenarios shown in Fig 6. Each image is labeled in the format “x_dpc_Sample_y_tn.jpg”, where “x” represents the number of days post cutting and “y” the replicate number.
(ZIP)

S2 Dataset Zipped Folder of Experimental Images for whole worms, L-Cut Scenarios, and dynein inhibition. This dataset contains raw-images of synapsin stains of uncut one- and two- headed worms, synapsin stains and brightfield images of the upwards and inverted L-cut scenarios, and synapsin stains and brightfield images showing the effects of the dynein inhibitor Ciliobrevin D on planaria regeneration. A Word document contained in the zip folder provides detailed description of the different cases.
(ZIP)

S3 Dataset Spreadsheet of raw heteromorphoses counts for experimental data appearing in Figs 4 and 7. (XLSX)
