# Supplementary material for: Neural control of body-plan axis in regenerating planaria
Source: PLoS Comput Biol. 2019 Apr 16;15(4):e1006904. doi: 10.1371/journal.pcbi.1006904 (PMC6485777; doi:10.1371/journal.pcbi.1006904)
Supplement: S1 Text — This document describes additional theoretical and mathematical aspects of the computational model and of the regulatory networks component of the planaria model. (PDF) [file pcbi.1006904.s001.pdf]

Supporting Information for Modeling Neural Control of Body-plan Axis Specification in Regenerating Planaria

1 Modeling Platform

Planaria models were implemented and explored using the **P**lanarian **I**nterface for **M**odeling **B**ody **O**rganization (PLIMBO), a 1D and 2D finite volume method simulator written in Python3 with open-source tools utilized from Scipy, Numpy, Matplotlib, Scikit-learn [1, 2], and BETSE [3, 4]. PLIMBO allows quantitative testing of the behavior of the regulatory network model reported on herein, in both 1D and 2D contexts, and under a range of experimental perturbations, with vector transport of morphogens on imported nerve polarity vector fields estimated from real worm synapsin stains, as well as the extraction of novel testable predictions. PLIMBO also has the capacity to run parameter searches to automatically iterate parameters to assist model parameterization, to perform sensitivity analyses, and to perform scaling analysis of the model (where a body-shape is progressively scaled); these tools all assist in the development and exploration of complex biological models. PLIMBO is freely available from:

https://gitlab.com/betse/plimbo

2 Planaria Model Morphogen Equations

A representation of the regulatory network underlying the planaria modeling is shown in S1-Figure 1. Initial conditions for all molecular factors were set at zero values, and all substances except Hedgehog (Hh) and Notum Regulating Factor (NRF) have the potential to be produced in any cells (i.e. there was no restriction placed on growth patterns of molecules). The proposed nerve-transported factors, Hh and NRF, had growth rates modulated by the nerve density map, $G(x,y)$, of the planaria model (see Supporting Figure S2).

β-Catenin (β-Cat)

β-Cat levels, modulated by the canonical Wnt/β-Cat signaling pathway, are known to play a major role in planaria regeneration, serving to inhibit formation of the head and to participate in definition of the posterior, where RNAi to β-Cat is well known to induce 2H heteromorphoses [5–7]. In our model, consistent with the canonical Wnt/β-Cat signaling pathway [8, 9], β-Cat is degraded by the β-Cat Destruction Complex (which includes APC as a major contender, as described below [10]). The

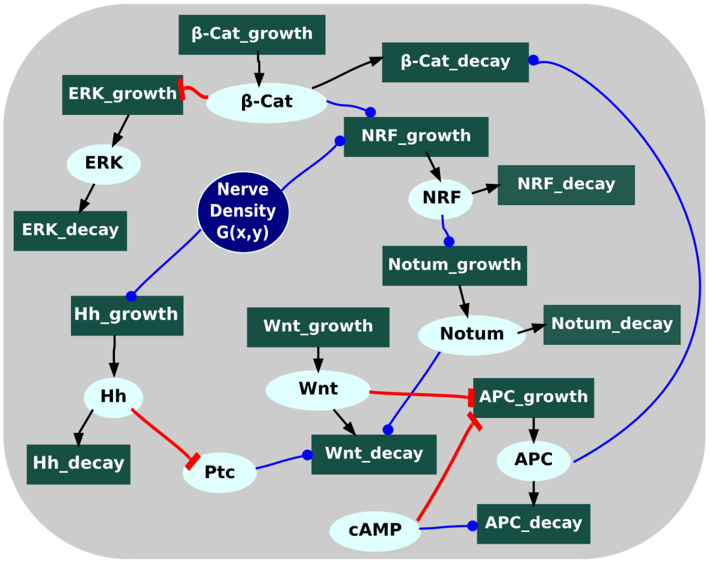

S1-Figure 1: A depiction of the complete gene regulatory network model used to model morphogen concentration in planaria homeostasis and regeneration. Black arrows show directions of mass transformation, blue lines with circular endpoints describe activation interactions, and red lines with flat endpoints describe inhibitor interactions. Substances of the model are shown as light blue ellipsoids. Rectangular boxes represent reactions (including production/decay of a substance). Mathematical handling of substance growth/decay, activation/inhibition relations, and vector transport are described in the following text.

core parameters for β-Cat dynamics are summarized in S1-Table 1. In our regulatory network model, the dynamics of β-Cat concentration (C_{bc}) are described by:

$$\frac{dC_{bc}}{dt} = r_{bc} - \left(d_{bc} + d_{bc}^{apc-deg} \left(\frac{(C_{apc}/K_{bc}^{apc})^{n_{bc}^{apc}}}{1 + (C_{apc}/K_{bc}^{apc})^{n_{bc}^{apc}}} \right) \right) C_{bc} + D_{bc} \nabla^2 C_{bc} \quad (1)$$

Adenomatous Polyposis Coli (APC)

Consistent with the canonical Wnt/β-Cat signaling pathway [8, 9], APC serves a crucial role in the β-Cat Destruction Complex, and is known to be inhibited by Wnts [10]. RNAi inhibition of APC is also known to generate 2T outcomes in planaria [11]. The APC/β-Cat Destruction Complex is assumed to be inactivated by cAMP [12]. The core parameters for APC dynamics are summarized in S1-Table 1. APC was not subjected to intercellular transport. In our regulatory network model, the dynamics of APC activity (C_{apc}) with respect to activation/deactivation of the β-Cat Destruction Complex are assumed to be fast compared to genetic expression, and are therefore described by a steady-state formulae to decrease the number of free parameters of the model. This steady-state approximation assumes equal rates of activation and deactivation of the APC signaling complex (leading to an activity level between 0 and 1 units).

The dynamic equation for C_{apc} :

$$\frac{dC_{apc}}{dt} = r_{apc} \left(\frac{1}{1 + \left(\frac{C_{Wnt}}{K_{apc}^{wnt}} \right)^{n_{apc}^{wnt}}} \right) \left(\frac{1}{1 + (C_{camp}/K_{apc}^{camp})^{n_{apc}^{camp}}} \right) - d_{apc} C_{apc} \left(\frac{(C_{camp}/K_{apc}^{camp})^{n_{apc}^{camp}}}{1 + (C_{camp}/K_{apc}^{camp})^{n_{apc}^{camp}}} \right) \quad (2)$$

was solved at steady state ($\frac{dC_{apc}}{dt} = 0$) to yield an expression for C_{apc} concentration at steady-state (assuming $r_{apc} = d_{apc}$):

$$C_{apc} = \left(\frac{\left(\frac{1}{1+\left(\frac{C_{Wnt}}{K_{apc}^{wnt}}\right)^{n_{apc}^{wnt}}} \right) \left(\frac{1}{1+(C_{camp}/K_{apc}^{camp})^{n_{apc}^{camp}}} \right)}{\left(\frac{(C_{camp}/K_{apc}^{camp})^{n_{apc}^{camp}}}{1+(C_{camp}/K_{apc}^{camp})^{n_{apc}^{camp}}} \right)} \right) \tag{3}$$

Extracellular Receptor Kinase (ERK)

ERK signaling has been shown to be associated with anterior development, where ERK is inhibited by β-Cat and inhibition of ERK leads to 0H heteromorphoses [13, 14]. Transport and production characteristics for ERK in our model are listed in S1-Table 1. ERK was not subject to intercellular transport. In our model, the rate of change of ERK signaling (C_{erk}) is assumed to be rapid compared with gene expression, and was therefore described by a steady-state equation assuming equal rates of activation/deactivation of ERK signaling (leading to an activity level between 0 and 1 units). The steady-state description of ERK also reduces the number of free parameters in the model.

The dynamic equation for C_{erk} :

$$\frac{dC_{erk}}{dt} = r_{erk} \left(\frac{1}{1+\left(\frac{C_{bc}}{K_{erk}^{bc}}\right)^{n_{erk}^{bc}}} \right) - d_{erk}\, C_{erk} \tag{4}$$

was solved at steady state ($\frac{dC_{erk}}{dt} = 0$) to yield an expression for C_{erk} concentration at steady-state (assuming $r_{erk} = d_{erk}$):

$$C_{erk} = \left(\frac{1}{1+\left(\frac{C_{bc}}{K_{erk}^{bc}}\right)^{n_{erk}^{bc}}} \right) \tag{5}$$

Hedgehog (Hh) and Patched (Ptc)

The Hedgehog-induced canonical Wnt/β-Cat pathway has been well demonstrated to play a role in planaria regeneration [6, 7, 15]. Experimental work on the Hh pathway in planaria indicates Ptc degrades Wnt11, while Hh inhibits the activity of Ptc [15,16]. RNAi inhibition of Hh leads to characteristic loss of tail (0T heteromorphoses), while RNAi inhibition of Ptc leads to 2T outcomes [6, 7, 15]. Key transport and production characteristics of Hh are listed in S1-Table 1. In our model, Hh is transported

via kinesin on the vector field described by $\vec{u}(x,y)$, and is produced on the nerve density field $G(x,y)$. Patched was modeled as a degradation term acting to enhance degradation of Wnt (see Eqs 7), and was assumed to be potentially active everywhere in the tissue. In our model, the dynamics of Hh concentration (C_{hh}) were described by:

$$\frac{dC_{hh}}{dt} = r_{hh} - d_{hh}\,C_{hh} - \nabla\cdot\left(-D_{hh}\,\nabla C_{hh} + u_{hh}\,\vec{u}(x,y)\,C_{hh}\right) \tag{6}$$

Wnt1 and Wnt11 (Wnt)

The canonical Wnt/β-Cat pathway has been well demonstrated to play a role in planaria regeneration [5,6], where polarized gradients of Wnt1 have been observed at the posterior edges of wounds [17,18] and Hh signaling enhances Wnt11 expression by blocking Patched (Ptc) [15]. Notum is further known to inhibit Wnts by inducing their degradation [19,20]. Inhibition of Wnt1 or Wnt11 independently does not induce heteromorphoses, but inhibition of both Wnt1 and Wnt11 induces 2H outcomes [15,16]. Furthermore, inhibition of either Notum (RNAi Notum) or Ptc (RNAi Ptc) are known to induce 0H and 2T outcomes [6,15–17]. On account of these observations, we included Wnt in our model as the representation of the combined activity of Wnt1 and Wnt11 (in order to reduce the number of free parameters in the model). Key transport and production characteristics of Wnt are listed in S1-Table 1. Wnts were subjected to regular diffusive transport only. The dynamics of Wnt concentration (C_{wnt}) were described by:

$$\frac{dC_{wnt}}{dt} = D_{wnt}\,\nabla^2 C_{wnt} + r_{wnt} - \left(d_{wnt} + d_{wnt}^{deg-notum}\left(\frac{\left(\frac{C_{notum}}{K_{wnt}^{notum}}\right)^{n_{wnt}^{notum}}}{1+\left(\frac{C_{notum}}{K_{wnt}^{notum}}\right)^{n_{wnt}^{notum}}}\right) + d_{wnt}^{deg-ptc}\left(\frac{1}{1+\left(\frac{C_{hh}}{K_{wnt}^{hh}}\right)^{n_{wnt}^{hh}}}\right)\right) C_{wnt} \tag{7}$$

Notum

Notum enzyme inhibits Wnt signaling [19,20], and is known to be required for anterior development, where RNAi to Notum is known to induce 2T outcomes [17]. Polarized gradients of Notum have been observed at the anterior edge of wounds [6,17]. Notum is known to be upregulated by β-Cat [21], although to correspond with the observations of increased Notum *transcription* at anterior wound edges [17], our model's logic indicates that the relationship between Notum and β-Cat must be indirect, and is proposed to occur via an intermediate factor (Notum Regulating Factor, see below). Characteristics and constants applying to Notum are listed in S1-Table 1. In the model, the dynamics of Notum concentration (C_{notum}) are described by:

$$\frac{dC_{notum}}{dt} = r_{notum}\left(\frac{\left(\frac{C_{nrf}}{K_{notum}^{nrf}}\right)^{n_{notum}^{nrf}}}{1+\left(\frac{C_{nrf}}{K_{notum}^{nrf}}\right)^{n_{notum}^{nrf}}}\right) - d_{notum}\,C_{notum} + D_{notum}\,\nabla^2 C_{notum} \tag{8}$$

Notum Regulating Factor (NRF)

While Notum is known to be upregulated by β-Cat [21], in order to match previous experimental observations of increased Notum *transcription* at anterior wound edges [17], the relationship between Notum and β-Cat must be indirect, and is proposed to occur via an intermediate and presently unidentified factor which we call Notum Regulating Factor (NRF). A fundamental hypothesis of our model is that NRF is upregulated by β-Cat, transported along nerve fibers in a direction from the axon to the nerve body by dynein, and in turn serves to upregulate Notum expression. NRF parameters are listed in S1-Table 1. In the model, the dynamics of NRF concentration (C_{nrf}) are described by:

$$\frac{dC_{nrf}}{dt} = r_{nrf} \left(\frac{\left(\frac{C_{bc}}{K_{nrf}^{bc}}\right)^{n_{nrf}^{bc}}}{1 + \left(\frac{C_{bc}}{K_{nrf}^{bc}}\right)^{n_{nrf}^{bc}}} \right) - d_{nrf}\, C_{nrf} - \nabla \cdot \left(-D_{nrf}\, \nabla C_{nrf} + u_{nrf}\, \vec{u}(x,y)\, C_{nrf} \right) \tag{9}$$

3',5' Cyclic Adenosine Monophosphate (cAMP)

Cyclic AMP is an important secondary messenger that has been found to support β-Cat stability [12], and to therefore be involved in promoting canonical Wnt/β-Cat stability. In planaria, cAMP levels are furthermore known to be increased by serotonin acting via a 5HT7R-like receptor, leading to 0H outcomes [22]. In planaria, cAMP may also be decreased by dopamine acting via D2R, leading to 2H outcomes [23–25]. In our model, the fundamental transport and production characteristics of cAMP are summarized in S1-Table 1. Given the fast rate of cAMP signaling dynamics, cAMP was considered only as a steady-state variable (C_{camp}).

3 Model Parameterization and Sensitivity Analysis

All parameters for the finalized model, including their values, description, units, and any supporting source references used to guide their specification, are summarized in S1-Table 1. During the parameterization process, values for all substances were initially set to diffusion constants of $1.5x10^{-11}$ m/s (which is in the medium range for known protein diffusion rates of ~50-70 kDa proteins [31, 34–36]), axoplasmic transport rates were set to $5.0x10^{-8}$ m/s (which is in the medium range for known axoplasmic transport rates [28–30, 33, 37]), production/transcription rates were set to $5.0x10^{-3}$ nM/s (which is in the range of known transcription rates for molecules of the canonical β-Cat/Wnt pathway [26, 27]), and decay and interaction rates were initially set by human inspection of model results (except for β-Cat decay rates which were initially set at that reported in [26]).

The planaria model was first parameterized by working in 1D, with an x-axis component of the axoplasmic transport $\overrightarrow{u}(x)$ and production gradient $G(x)$ described by the same mathematical function:

$$u(x) = \frac{1}{1 + \sqrt{(2\,x/x_{mid})}} \tag{10}$$

Index	Parameter	Description	Source	Base value	Units	% ΔErk	% Δβ-Cat
1	r_{bc}	β-Cat max production rate	Estimated; [26,27]	$2.5x10^{-3}$	nM/s	-9.5	6.3
2	d_{bc}	Base β-Cat decay constant	Estimated; [26]	$1x10^{-6}$	$1/s$	4.0	-1.2
3	$d_{bc}^{apc-deg}$	APC-induced β-Cat degradation constant	Estimated	$5.0x10^{-3}$	$1/s$	3.9	-3.5
4	K_{bc}^{apc}	$K_{1/2}$ constant for APC interaction with β-Cat	Estimated	0.5	nM	-7.2	6.4
5	n_{bc}^{apc}	Hill coefficient for APC interaction with β-Cat	Estimated	2		-17.7	11.0
6	D_{bc}	β-Cat diffusion constant	Estimated	$1x10^{-12}$	m^2/s	2.2	-0.2
7	K_{erk}^{bc}	$K_{1/2}$ constant for β-Cat interaction with ERK	Estimated	30	nM	23.4	0.0
8	n_{erk}^{bc}	Hill exponent for β-Cat interaction with ERK	Estimated	2		-31.8	0.0
9	K_{apc}^{camp}	$K_{1/2}$ constant for cAMP interaction with APC	Estimated	1	μM	16.7	-10.5
10	n_{apc}^{camp}	Hill exponent for cAMP interaction with APC	Estimated	2		0.0	0.0
11	K_{apc}^{wnt}	$K_{1/2}$ constant for Wnt interaction with APC	Estimated	30	nM	16.1	-8.9
12	n_{apc}^{wnt}	Hill coefficient for Wnt interaction with APC	Estimated	2		-20.3	13.5
13	r_{hh}	Hh max production rate	Estimated	0.005	nM/s	-17.5	18.4
14	d_{hh}	Hh decay constant	Estimated	$5x10^{-6}$	$1/s$	19.0	-8.0
15	D_{hh}	Hh diffusion constant	Estimated	$1.5x10^{-11}$	m^2/s	-9.3	13.2
16	u_{hh}	Max Hh axoplasmic transport rate	Estimated; [28–30]	$3x10^{-8}$	m/s	14.7	-9.2
17	r_{wnt}	Wnt max production rate	Estimated	0.01	nM/s	-13.2	11.0
18	d_{wnt}	Wnt decay constant	Estimated	$1x10^{-5}$	$1/s$	2.0	-0.9
19	K_{wnt}^{notum}	$K_{1/2}$ constant for Notum interaction with Wnt	Estimated	0.5	nM	-8.9	8.6
20	n_{wnt}^{notum}	Hill coefficient for Notum interaction with Wnt	Estimated	2.5		-13.8	11.5
21	D_{wnt}	Wnt diffusion constant	Estimated; [31,32]	$5x10^{-12}$	m^2/s	0.6	0.8
22	$d_{wnt}^{deg-notum}$	Notum-induced Wnt degradation constant	Estimated	$1.0x10^{-4}$	$1/s$	5.1	-4.2
23	$d_{wnt}^{deg-ptc}$	Ptc-induced Wnt degradation constant	Estimated	$7.5x10^{-5}$	$1/s$	7.2	-4.6
24	K_{wnt}^{hh}	$K_{1/2}$ constant for Hh interaction with Wnt	Estimated	300	nM	20.8	-9.4
25	n_{wnt}^{hh}	Hill coefficient for Hh interaction with Wnt	Estimated	2.5		-16.9	13.6
26	r_{nrf}	NRF max production rate	Estimated	$7.5x10^{-3}$	nM/s	22.6	-13.8
27	d_{nrf}	NRF decay constant	Estimated	$7.5x10^{-6}$	$1/s$	-18.9	21.8
28	K_{nrf}^{bc}	$K_{1/2}$ constant for BC interaction with NRF	Estimated	150	nM	-9.8	9.1
29	n_{nrf}^{bc}	Hill coefficient for BC interaction with NRF	Estimated	2		-2.8	2.7
30	D_{nrf}	NRF diffusion constant	Estimated; [31,32]	$1.5x10^{-11}$	m^2/s	0.1	2.1
31	u_{nrf}	Max NRF axoplasmic transport rate	Estimated; [28,30,33]	$-6.0x10^{-8}$	m/s	-0.3	-2.3
32	r_{notum}	Notum max production rate	Estimated	$2.5x10^{-3}$	nM/s	9.7	-7.2
33	d_{notum}	Notum decay constant	Estimated	$2.5x10^{-3}$	$1/s$	-8.9	8.6
34	K_{notum}^{nrf}	$K_{1/2}$ constant for NRF interaction with Notum	Estimated	250	nM	-18.1	18.9
35	n_{notum}^{nrf}	Hill coefficient for NRF interaction with Notum	Estimated	2		-14.7	12.0
36	D_{notum}	Notum diffusion constant	Estimated; [31,32]	$1.5x10^{-11}$	m^2/s	0.0	0.0

S1-Table 1: Parameter definition, base values, references, and sensitivities of ERK and β-Cat gradients to a 15% increase of each parameter in a local sensitivity analysis.

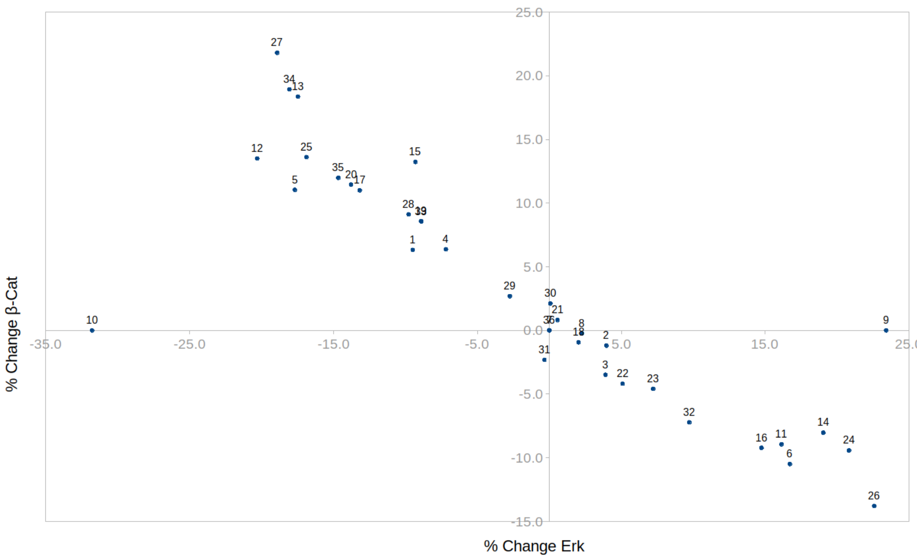

S1-Figure 2: Sensitivity analysis for planaria model, showing average percent change in ERK morphogen concentration against percent change in β-Cat morphogen gradient for individual perturbations of 15% increase to each parameter, where the parameter index (see S1-Table 1) labels the data point. The sensitivity analysis shows that the model's key morphogen gradients, ERK and β-Cat (both of which were assessed at 108 hrs simulated time in a 1H worm model cut into 5 pieces) depend significantly on a large number of model parameters, and that the model is stable for moderate perturbations to all parameter values.

These 1D descriptions for $\vec{u}(x)$ was chosen due to its qualitatively close correspondence to the 2D map of $G(x,y)$ and $\vec{u}(x,y)$ derived from planarian models (see Supplementary Figure S2_Fig.tif). Here, x_{mid} is the midpoint of the x-axis ($x_{mid} = 0.5$).

The 1D model features rapid simulation times, which allowed for a high-throughput in model testing and iterations, and shows similar results to that of the 2D model. After achieving adequate results in 1D, the model was transitioned to the full 2D version using the neural production and transport maps $G(x,y)$ and $\vec{u}(x,y)$, respectively, shown in the Supporting Figure S2_Figure.tif.

A local sensitivity analysis [38–40] was performed on the 2D model to identify parameters with the highest impact on model outcome, and to screen out parameters with the lowest impact, where sensitivity results for the final 2D model are summarized in S1-Table 1. The sensitivity analysis was conducted in PLIMBO by increasing the value of a parameter by 15%, while leaving all other factors constant, and by tracking the average percent change in the model's output concentrations of ERK or β-Cat after 96 hours of simulated time proceeding cutting a 1H worm model into 5 pieces. The sensitivity also showed the model results to depend strongly on the majority of parameters of the model (S1-Figure 2, S1-Table 1).

To obtain a model with general conformance to the complex dataset consisting of repolarization of ERK and β-Cat gradients in fragments of various sizes, as well as the heteromorphoses expected from the set of 10 interventions (which simulate RNAi or pharmacological treatments conducted in experiments), model parameters were progressively altered in PLIMBO by increasing each parameter above and below its initial value, while keeping all other parameters constant, thereby producing a set of model variations (S1-Figure 3). Output from model variations were visually inspected and the best variant was selected based on its ability to produce and re-polarize instructive ERK and β-Cat gradients after cutting, and its ability to produce the expected heteromorphoses with the set

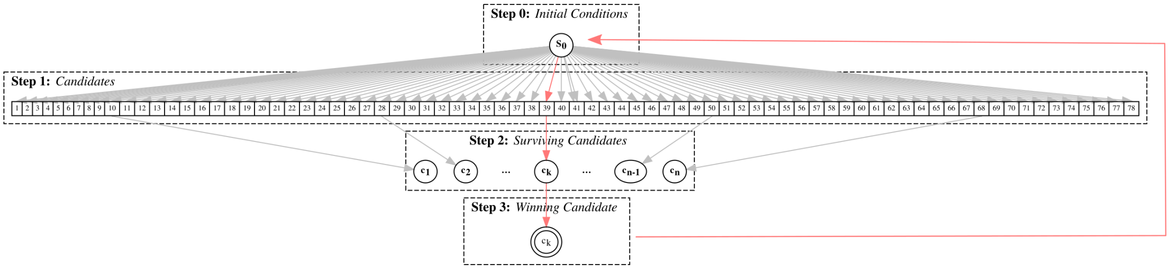

S1-Figure 3: Model parameterization was assisted by automated iteration of parameters in PLIMBO. A model with an initial set of parameter values (Step 0) was used to generate a number of model variations (Step 1) by progressively decreasing or increasing the value of each parameter value by multiplying or dividing by a fixed factor (0.5 to 0.8). Model variations were visually inspected and surviving candidates meeting all selection criteria were preserved (Step 2), with a wining candidate selected from the set of surviving candidates based on its qualitative ability to meet requirements (Step 3). The process was repeated with the wining candidate until further (qualitatively-assessed) improvements were no longer observed.

of 10 RNAi and pharmacological interventions (S1-Figure 3). The automated parameter search was repeated until no further qualitative improvements were obtained in the set of variants (S1-Figure 3).

References

[1] Millman KJ, Aivazis M. Python for Scientists and Engineers. Computing in Science & Engineering. 2011 Mar;13(2):9–12.

[2] Oliphant TE. Python for Scientific Computing. Computing in Science & Engineering. 2007 May;9(3):10–20.

[3] Pietak A, Levin M. Exploring Instructive Physiological Signaling with the Bioelectric Tissue Simulation Engine. Bioinformatics and Computational Biology. 2016;p. 55.

[4] Pietak A, Levin M. Bioelectric Gene and Reaction Networks: Computational Modelling of Genetic, Biochemical and Bioelectrical Dynamics in Pattern Regulation. Journal of the Royal Society, Interface. 2017 Sep;14(134).

[5] Gurley KA, Rink JC, Alvarado AS. -Catenin Defines Head Versus Tail Identity During Planarian Regeneration and Homeostasis. Science. 2008 Jan;319(5861):323–327.

[6] Owlarn S, Bartscherer K. Go Ahead, Grow a Head! A Planarian's Guide to Anterior Regeneration: Planarian Anterior Regeneration. Regeneration. 2016 Jun;3(3):139–155.

[7] Gurley KA, Elliott SA, Simakov O, Schmidt HA, Holstein TW, Alvarado AS. Expression of Secreted Wnt Pathway Components Reveals Unexpected Complexity of the Planarian Amputation Response. Developmental Biology. 2010 Nov;347(1):24–39.

[8] Caspi M, Zilberberg A, Eldar-Finkelman H, Rosin-Arbesfeld R. Nuclear GSK-3beta Inhibits the Canonical Wnt Signalling Pathway in a Beta-Catenin Phosphorylation-Independent Manner. Oncogene. 2008 Jun;27(25):3546–3555.

[9] Robertis EMD. Wnt Signaling in Axial Patterning and Regeneration: Lessons from Planaria. Science Signaling. 2010 Jun;3(127):pe21–pe21.

[10] Aoki K, Taketo MM. Adenomatous Polyposis Coli (APC): A Multi-Functional Tumor Suppressor Gene. Journal of Cell Science. 2007 Oct;120(19):3327–3335.

[11] Stückemann T, Cleland JP, Werner S, Thi-Kim Vu H, Bayersdorf R, Liu SY, et al. Antagonistic Self-Organizing Patterning Systems Control Maintenance and Regeneration of the Anteroposterior Axis in Planarians. Developmental Cell. 2017 Feb;40(3):248–263.e4.

[12] Hino Si, Tanji C, Nakayama KI, Kikuchi A. Phosphorylation of Beta-Catenin by Cyclic AMP-Dependent Protein Kinase Stabilizes Beta-Catenin through Inhibition of Its Ubiquitination. Molecular and Cellular Biology. 2005 Oct;25(20):9063–9072.

[13] Umesono Y, Tasaki J, Nishimura Y, Hrouda M, Kawaguchi E, Yazawa S, et al. The Molecular Logic for Planarian Regeneration along the Anterior–Posterior Axis. Nature. 2013 Aug;500(7460):73–76.

[14] Agata K, Tasaki J, Nakajima E, Umesono Y. Recent Identification of an ERK Signal Gradient Governing Planarian Regeneration. Zoology. 2014 Jun;117(3):161–162.

[15] Yazawa S, Umesono Y, Hayashi T, Tarui H, Agata K. Planarian Hedgehog/Patched Establishes Anterior–Posterior Polarity by Regulating Wnt Signaling. Proceedings of the National Academy of Sciences. 2009 Dec;106(52):22329–22334.

[16] Rink JC, Gurley KA, Elliott SA, Sanchez Alvarado A. Planarian Hh Signaling Regulates Regeneration Polarity and Links Hh Pathway Evolution to Cilia. Science. 2009;326(5958):1406–10.

[17] Petersen CP, Reddien PW. Polarized Notum Activation at Wounds Inhibits Wnt Function to Promote Planarian Head Regeneration. Science. 2011 May;332(6031):852–855.

[18] Petersen CP, Reddien PW. A Wound-Induced Wnt Expression Program Controls Planarian Regeneration Polarity. Proceedings of the National Academy of Sciences of the United States of America. 2009 Oct;106(40):17061–17066.

[19] Zhang X, Cheong SM, Amado NG, Reis AH, MacDonald BT, Zebisch M, et al. Notum Is Required for Neural and Head Induction via Wnt Deacylation, Oxidation, and Inactivation. Developmental Cell. 2015 Mar;32(6):719–730.

[20] Kakugawa S, Langton PF, Zebisch M, Howell SA, Chang TH, Liu Y, et al. Notum Deacylates Wnt Proteins to Suppress Signalling Activity. Nature. 2015 Feb;519(7542):187–192.

[21] Torisu Y, Watanabe A, Nonaka A, Midorikawa Y, Makuuchi M, Shimamura T, et al. Human Homolog of NOTUM, Overexpressed in Hepatocellular Carcinoma, Is Regulated Transcriptionally by Beta-Catenin/TCF. Cancer Science. 2008 Jun;99(6):1139–1146.

[22] Chan JD, Grab T, Marchant JS. Kinetic Profiling an Abundantly Expressed Planarian Serotonergic GPCR Identifies Bromocriptine as a Perdurant Antagonist. International Journal for Parasitology: Drugs and Drug Resistance. 2016 Dec;6(3):356–363.

[23] Min C, Cho DI, Kwon KJ, Kim KS, Shin CY, Kim KM. Novel Regulatory Mechanism of Canonical Wnt Signaling by Dopamine D2 Receptor through Direct Interaction with β-Catenin. Molecular Pharmacology. 2011 Jul;80(1):68–78.

[24] Stonehouse AH, Jones FS. Bromocriptine and Clozapine Regulate Dopamine 2 Receptor Gene Expression in the Mouse Striatum. Journal of molecular neuroscience: MN. 2005;25(1):29–36.

[25] Chan JD, Agbedanu PN, Zamanian M, Gruba SM, Haynes CL, Day TA, et al. ‘Death and Axes’: Unexpected Ca2+ Entry Phenologs Predict New Anti-Schistosomal Agents. PLoS Pathogens. 2014 Feb;10(2):e1003942.

[26] Benary U, Kofahl B, Hecht A, Wolf J. Modeling Wnt/β-Catenin Target Gene Expression in APC and Wnt Gradients Under Wild Type and Mutant Conditions. Frontiers in Physiology. 2013;4:21.

[27] Gasior K, Hauck M, Wilson A, Bhattacharya S. A Theoretical Model of the Wnt Signaling Pathway in the Epithelial Mesenchymal Transition. Theoretical Biology and Medical Modelling. 2017 Oct;14(1):19.

[28] Tang Y, Scott D, Das U, Gitler D, Ganguly A, Roy S. Fast Vesicle Transport Is Required for the Slow Axonal Transport of Synapsin. Journal of Neuroscience. 2013 Sep;33(39):15362–15375.

[29] Brown A. Axonal Transport of Membranous and Nonmembranous Cargoes. The Journal of Cell Biology. 2003 Mar;160(6):817–821.

[30] Maday S, Twelvetrees AE, Moughamian AJ, Holzbaur ELF. AXONAL TRANSPORT: CARGO-SPECIFIC MECHANISMS OF MOTILITY AND REGULATION. Neuron. 2014 Oct;84(2):292–309.

[31] Nenninger A, Mastroianni G, Mullineaux CW. Size Dependence of Protein Diffusion in the Cytoplasm of Escherichia Coli. Journal of Bacteriology. 2010 Sep;192(18):4535–4540.

[32] Young ME, Carroad PA, Bell RL. Estimation of Diffusion Coefficients of Proteins. Biotechnology and Bioengineering. 1980 May;22(5):947–955.

[33] Cosker KE, Courchesne SL, Segal RA. Action in the Axon: Generation and Transport of Signaling Endosomes. Current Opinion in Neurobiology. 2008 Jun;18(3):270–275.

[34] Annunziata O, Buzatu D, Albright JG. Protein Diffusion Coefficients Determined by Macroscopic-Gradient Rayleigh Interferometry and Dynamic Light Scattering. Langmuir. 2005 Dec;21(26):12085–12089.

[35] Kühn T, Ihalainen TO, Hyväluoma J, Dross N, Willman SF, Langowski J, et al. Protein Diffusion in Mammalian Cell Cytoplasm. PLOS ONE. 19-Aug-2011;6(8):e22962.

[36] Brune D, Kim S. Predicting Protein Diffusion Coefficients. Proceedings of the National Academy of Sciences. 1993 May;90(9):3835–3839.

[37] Stenoien DL, Brady ST. Discovery and Conceptual Development of Fast and Slow Axonal Transport. Basic Neurochemistry: Molecular, Cellular and Medical Aspects 6th edition. 1999;.

[38] Sher AA, Wang K, Wathen A, Maybank PJ, Mirams GR, Abramson D, et al. A Local Sensitivity Analysis Method for Developing Biological Models with Identifiable Parameters: Application to Cardiac Ionic Channel Modelling. Future Generation Computer Systems. 2013 Feb;29(2):591–598.

[39] Zhou X, Lin H. Local Sensitivity Analysis. In: Shekhar S, Xiong H, Zhou X, editors. Encyclopedia of GIS. Cham: Springer International Publishing; 2017. p. 1130–1131.

[40] Morio J. Global and Local Sensitivity Analysis Methods for a Physical System. European Journal of Physics. 2011;32(6):1577.
