# Supplementary figures and images for: Neural control of body-plan axis in regenerating planaria (part 1 of 4)
Source: PLoS Comput Biol. 2019 Apr 16;15(4):e1006904. doi: 10.1371/journal.pcbi.1006904 (PMC6485777; doi:10.1371/journal.pcbi.1006904)

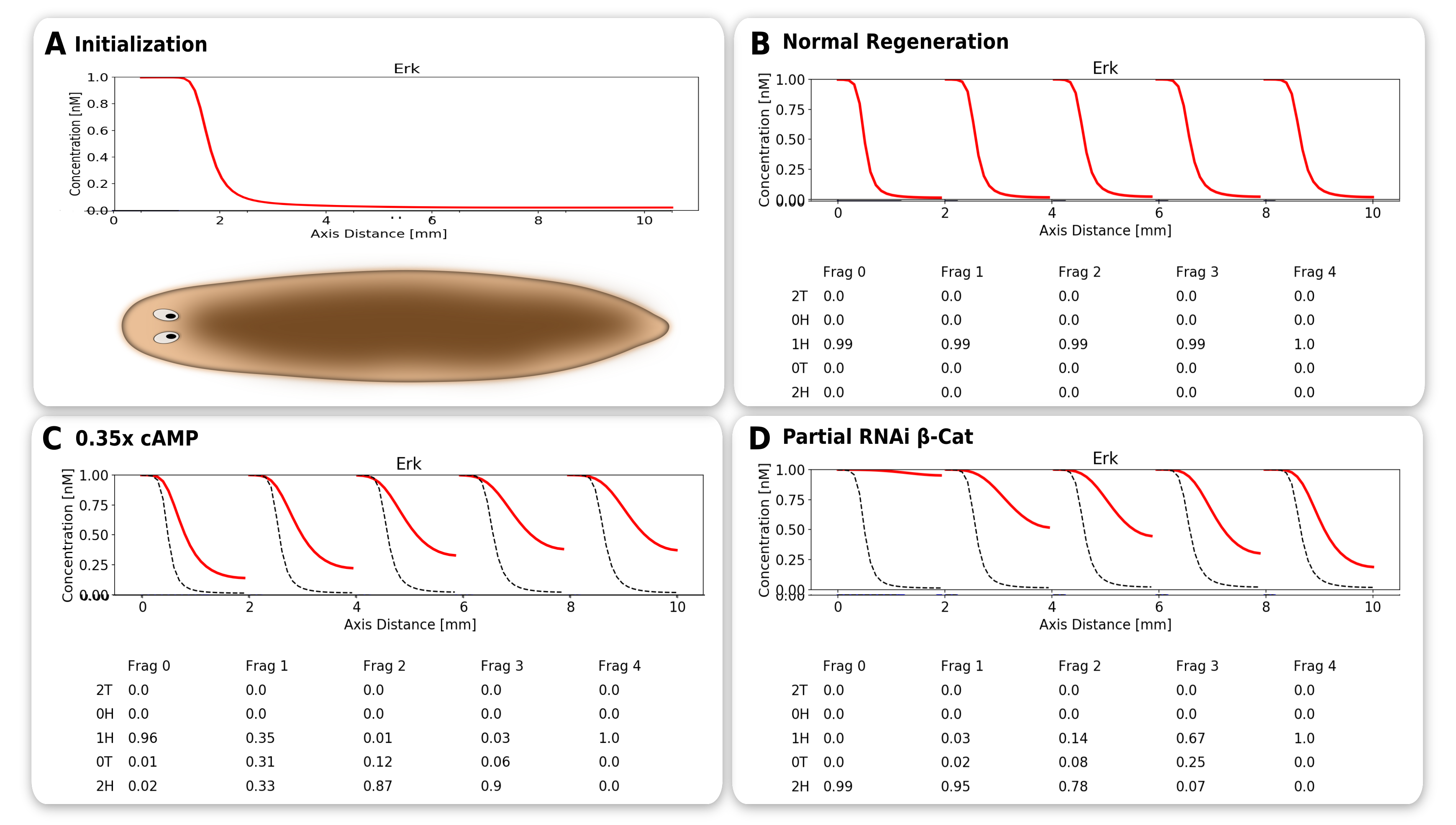

Supplement: S2 Fig — (TIF) [file pcbi.1006904.s003.tif]

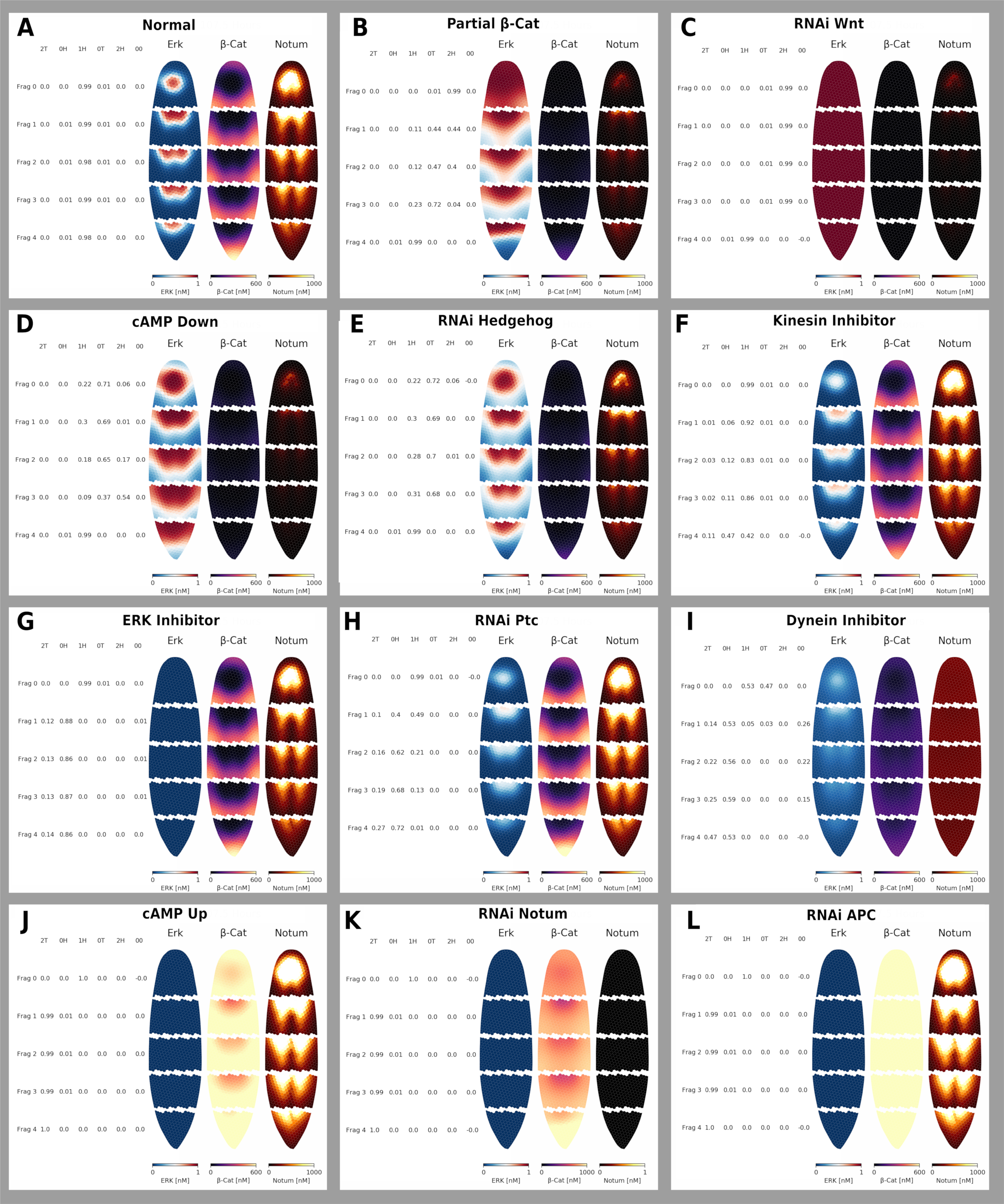

Supplement: S3 Fig — Panel A shows an untreated worm model cut into 5 pieces, and panels B through L show the results of 11 different interventions tested by the model. All panels show model-predicted steady-state concentrations of ERK, β-Cat, and Notum for an initially 1-headed worm cut into 5 pieces, 4.5 simulated days after cutting. The table to the left of each of the 2D morphogen maps summarizes the probabilistic regeneration outcomes predicted for each cut fragment, which were calculated from steady-state model ERK and β-Cat concentrations using the Markov Chain Model for Regeneration, described in the manuscript and in Fig 3. Here ‘2T’ = ‘two tailed’, ‘0H’ = ‘headless’, ‘1H’ = ‘one headed, one tailed’, ‘0T’ = ‘tailless’, ‘2H’ = ‘two headed’, and ‘00’ represents no head or tail). (TIF) [file pcbi.1006904.s004.tif]

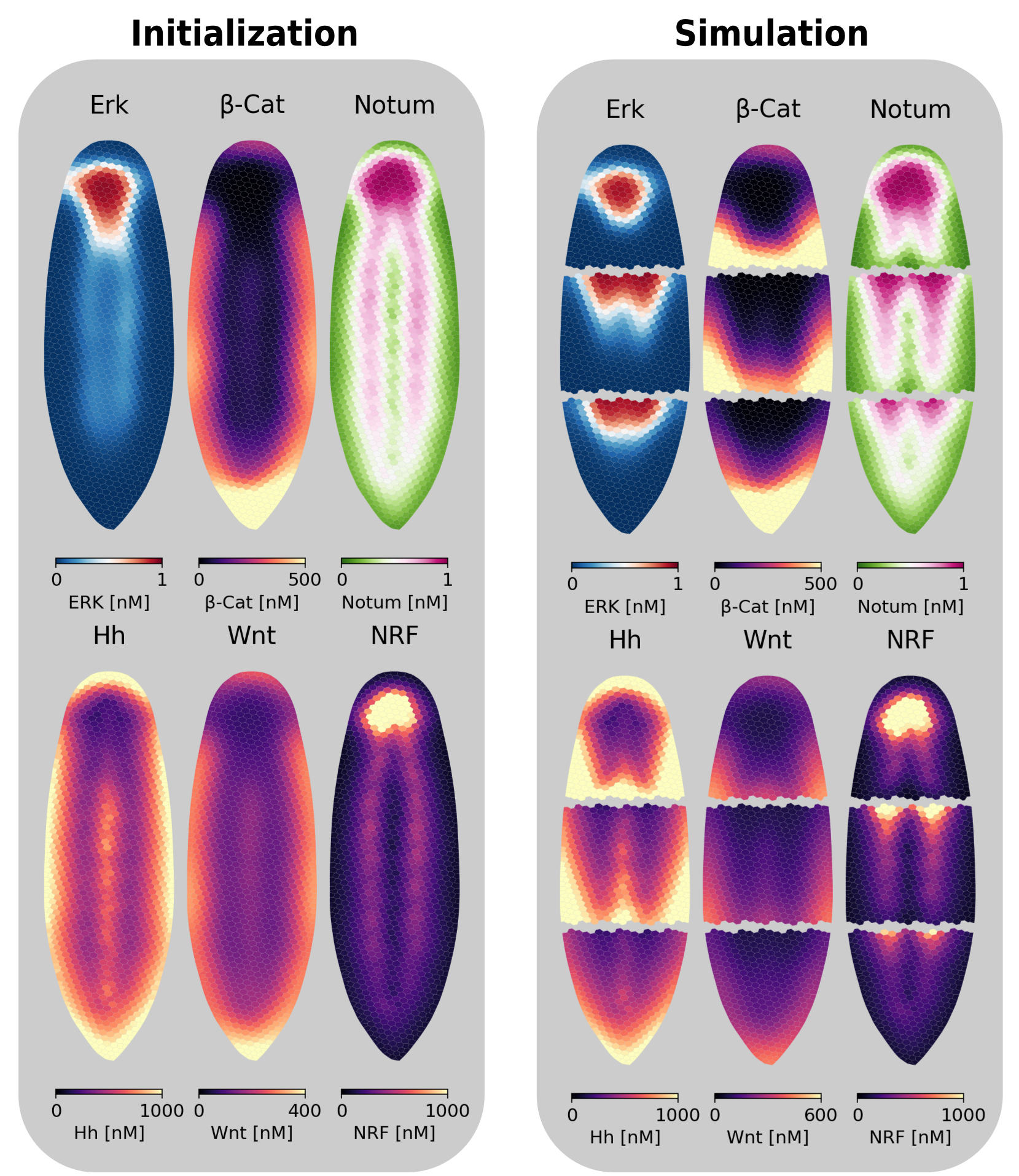

Supplement: S4 Fig — Note that while factors such as NRF are induced by β-Cat, the localization of the NRF does not correspond to the pattern of the inducing agent due to the effect of axoplasmic transport. Therefore, while β-Cat is highest at the posterior, NRF appears in a polarized manner at anterior wound edges after cutting. As NRF induces Notum, Notum expression and protein levels are predicted to also appear localized at anterior wound edges, which has been observed experimentally [32]. (TIF) [file pcbi.1006904.s005.tif]

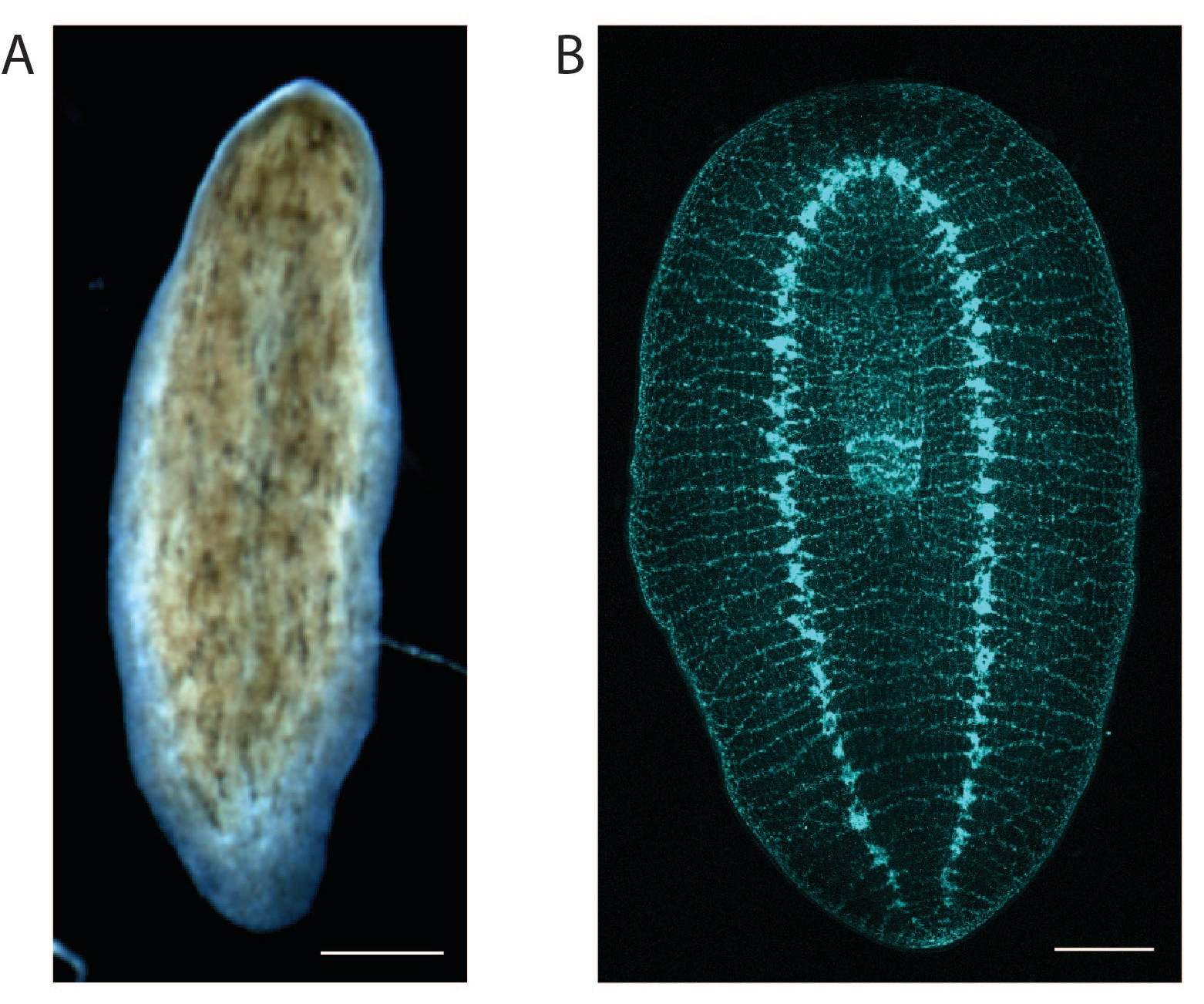

Supplement: S5 Fig — Brightfield image (A) and synapsin stain (B) of a headless worm regenerated from a trunk fragment following Ciliobrevin D treatment. N = 23/47 regenerated as headless and the remaining as 1H wildtype. Scale bar 300 μm. (TIF) [file pcbi.1006904.s006.tif]

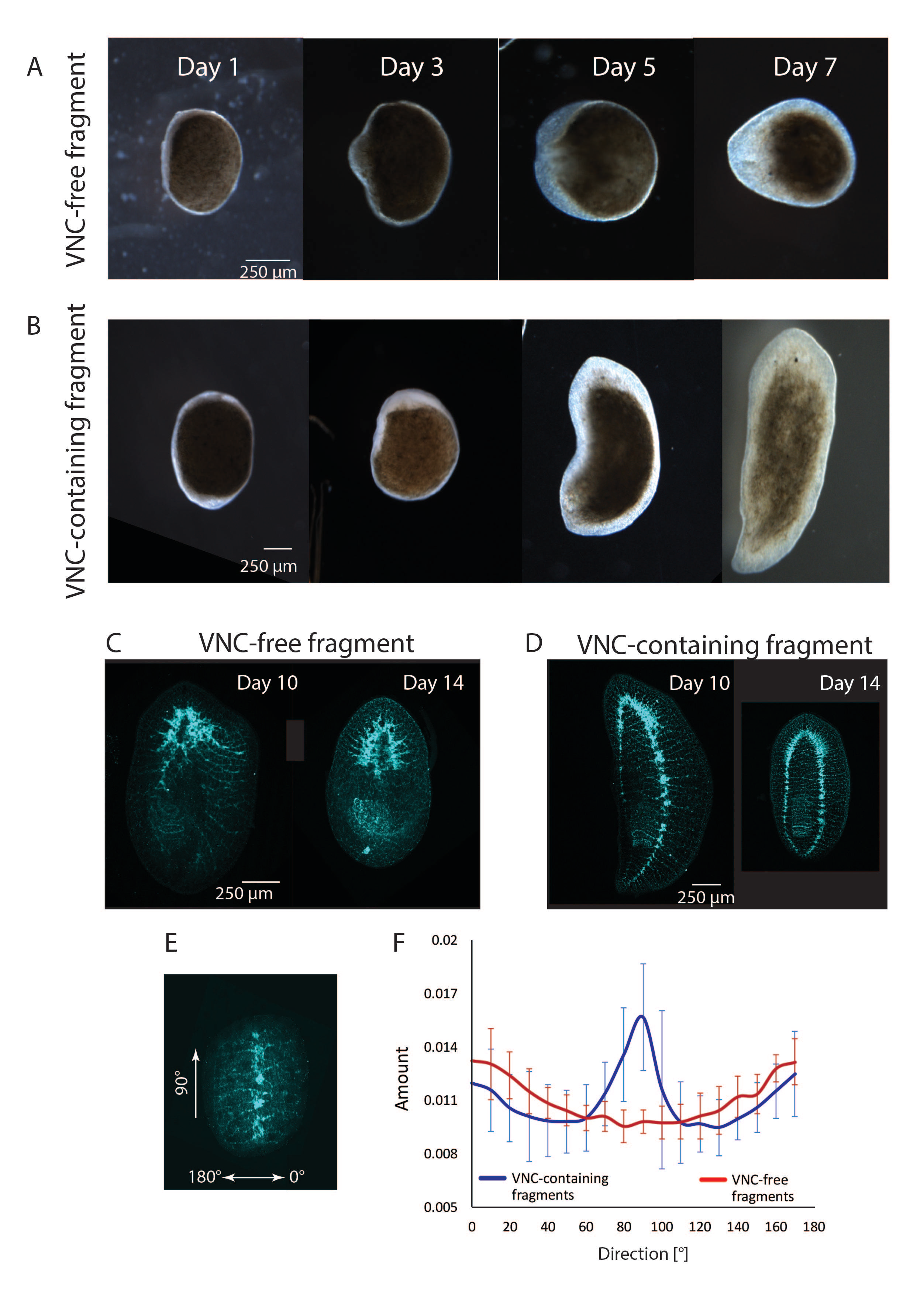

Supplement: S7 Fig — Brightfield images of small fragments not containing the VNC as regeneration proceeds on day 1, 3, 5, 7 after cutting are shown in (A). Brightfield images of small fragments containing the VNC as it regenerates are shown in (B). For corresponding synapsin stains see Fig 6. Synapsin stains for later regenerative time points at day 10 and day 14 after cutting of a VNC-free fragments show gradual development of a full brain (C). Synapsin stain for later regenerative time points at day 10 and day 14 after cutting of VNC-containing fragments show gradual development of a full brain (D). Scheme showing orientation of synapsin stain signal as measured by the Directionality plugin in ImageJ, with fibers perpendicular to the long axis of the fragments (non-VNC neurons) having an orientation of 0 and180°, while signal in parallel with the long axis (VNC) has an orientation of 90° (E). The orientation of synapsin signal in fragments at day 1 after cutting for both VNC-free (red) and VNC-containg (blue) fragments, shows peak orientation at 0/180 and 90 degrees, respectively (F). N = 5 samples were analysed for directionality, where error bars show standard deviation. A total of N = 94/94 VNC-free fragments and N = 103/103 VNC-containing fragments regenerated consistently with descriptions. Scale bars show 250 μm. (TIF) [file pcbi.1006904.s008.tif]

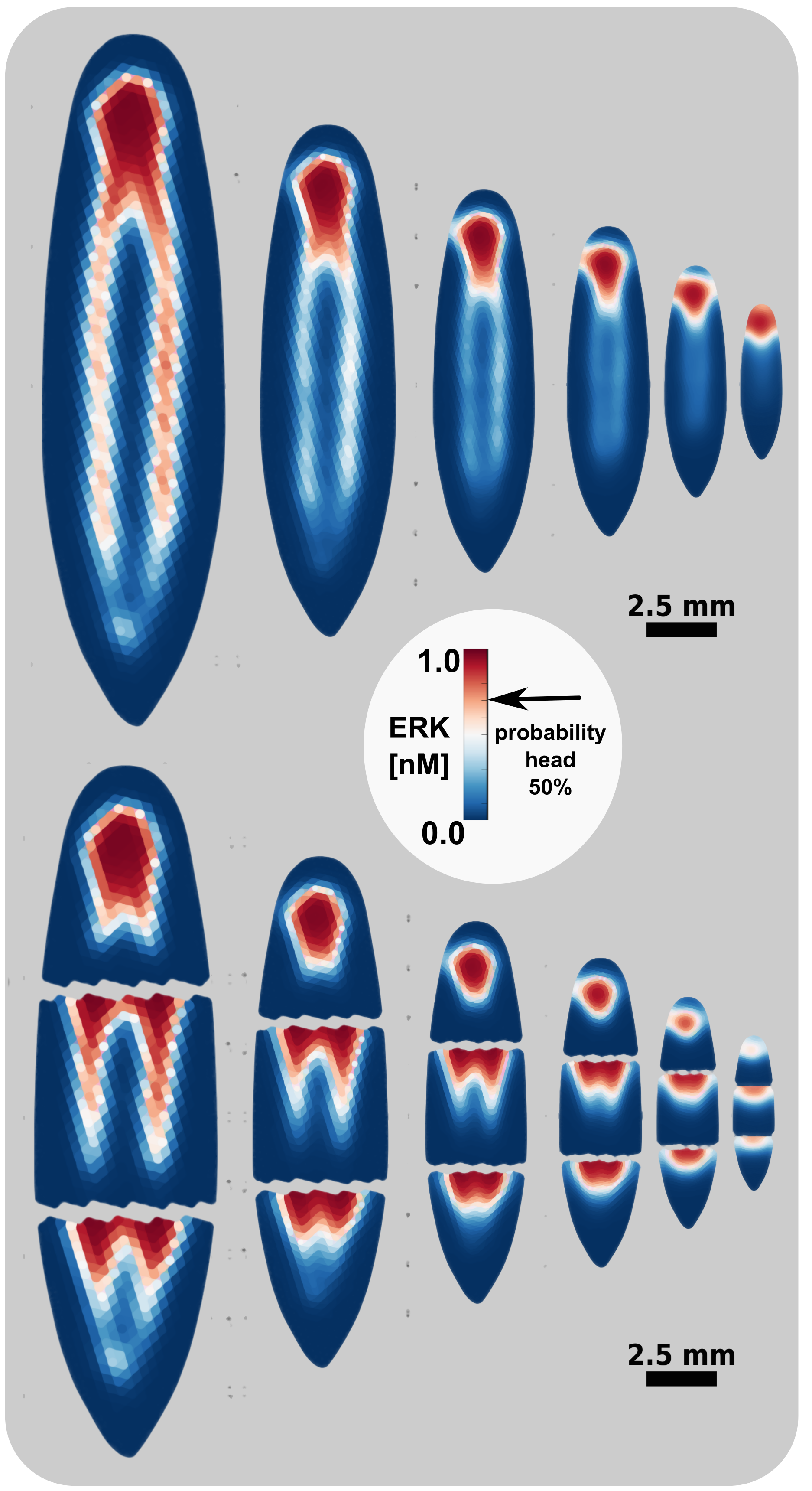

Supplement: S8 Fig — Here the simulated ERK concentration is shown in both whole worms (A), and in worms cut into three fragments (B), for planarian body models ranging from 2.5 to 0.5 cm in length. Morphogen gradients develop more detail as the body size increases, but remain able to specify head/tail identity at wounds. As body size decreases, the model predicts that small fragments (below ~1 mm in length) will fail to effectively re-polarize morphogen gradients, and that therefore axis disruptions and heteromorphoses should appear in regenerates from very small fragments, which is a well-known phenomenon [108–110]. (TIF) [file pcbi.1006904.s009.tif]

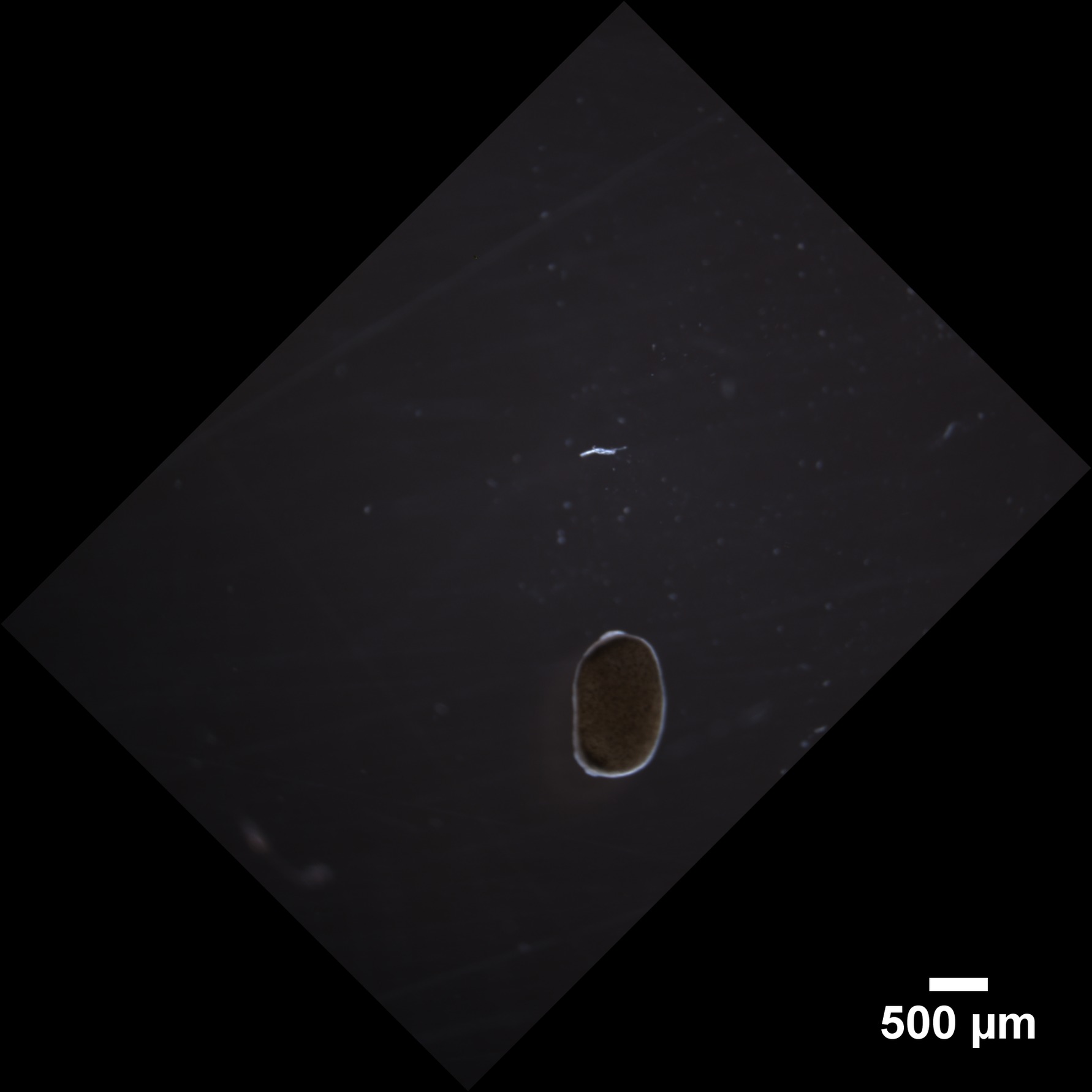

Supplement: S1 Dataset — This dataset contains brightfield image and corresponding synapsin stains for the VNC-free and VNC-containing small fragment cutting scenarios shown in Fig 6. Each image is labeled in the format “x_dpc_Sample_y_tn.jpg”, where “x” represents the number of days post cutting and “y” the replicate number. (ZIP) [file pcbi.1006904.s016.zip › smallfragments/VNC-containing/Brightfield_images/1 dpc_Sample 1_tn.jpg]

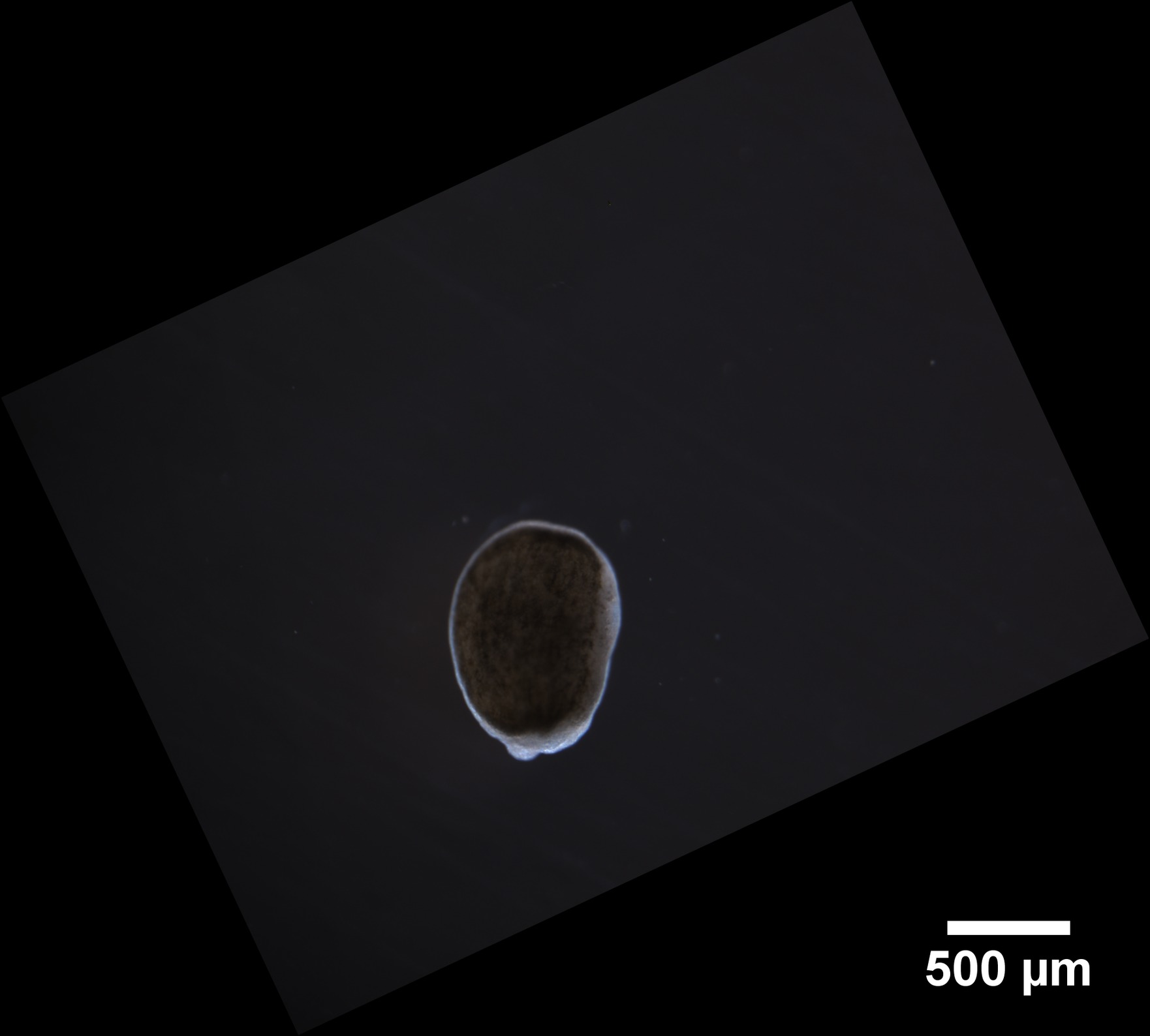

Supplement: S1 Dataset — This dataset contains brightfield image and corresponding synapsin stains for the VNC-free and VNC-containing small fragment cutting scenarios shown in Fig 6. Each image is labeled in the format “x_dpc_Sample_y_tn.jpg”, where “x” represents the number of days post cutting and “y” the replicate number. (ZIP) [file pcbi.1006904.s016.zip › smallfragments/VNC-containing/Brightfield_images/1 dpc_Sample 2_tn.jpg]

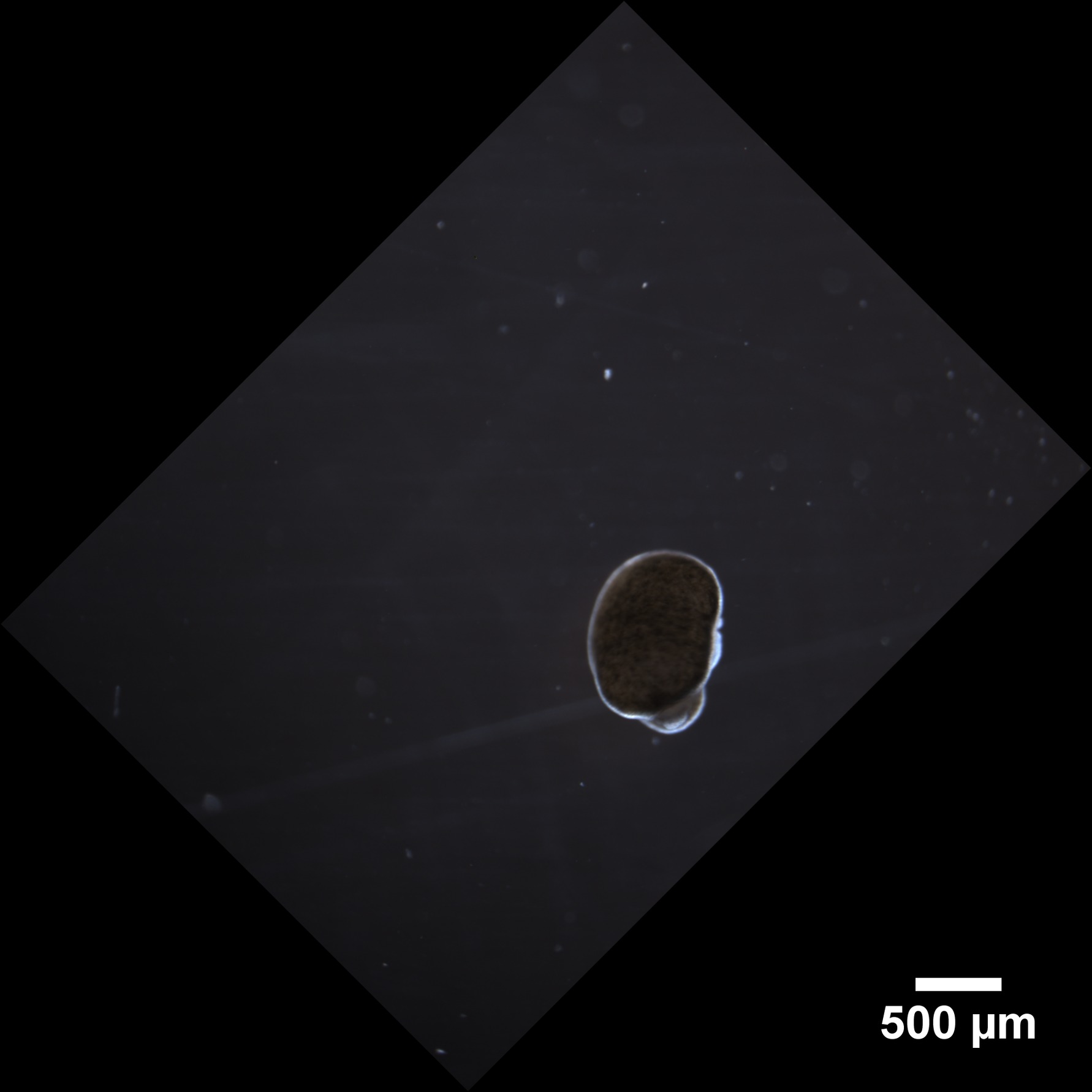

Supplement: S1 Dataset — This dataset contains brightfield image and corresponding synapsin stains for the VNC-free and VNC-containing small fragment cutting scenarios shown in Fig 6. Each image is labeled in the format “x_dpc_Sample_y_tn.jpg”, where “x” represents the number of days post cutting and “y” the replicate number. (ZIP) [file pcbi.1006904.s016.zip › smallfragments/VNC-containing/Brightfield_images/1 dpc_Sample 3_tn.jpg]

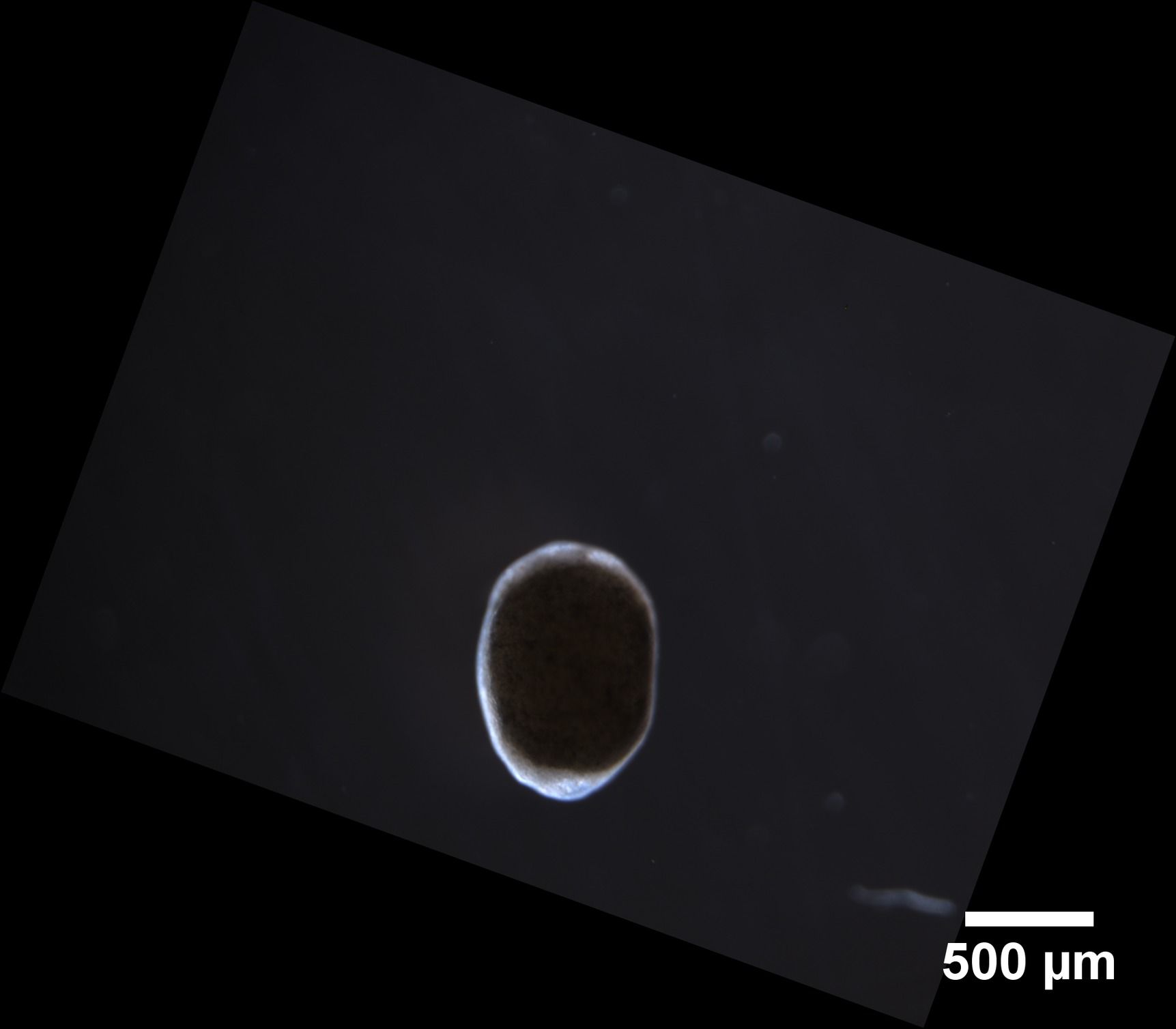

Supplement: S1 Dataset — This dataset contains brightfield image and corresponding synapsin stains for the VNC-free and VNC-containing small fragment cutting scenarios shown in Fig 6. Each image is labeled in the format “x_dpc_Sample_y_tn.jpg”, where “x” represents the number of days post cutting and “y” the replicate number. (ZIP) [file pcbi.1006904.s016.zip › smallfragments/VNC-containing/Brightfield_images/1 dpc_Sample 4_tn.jpg]

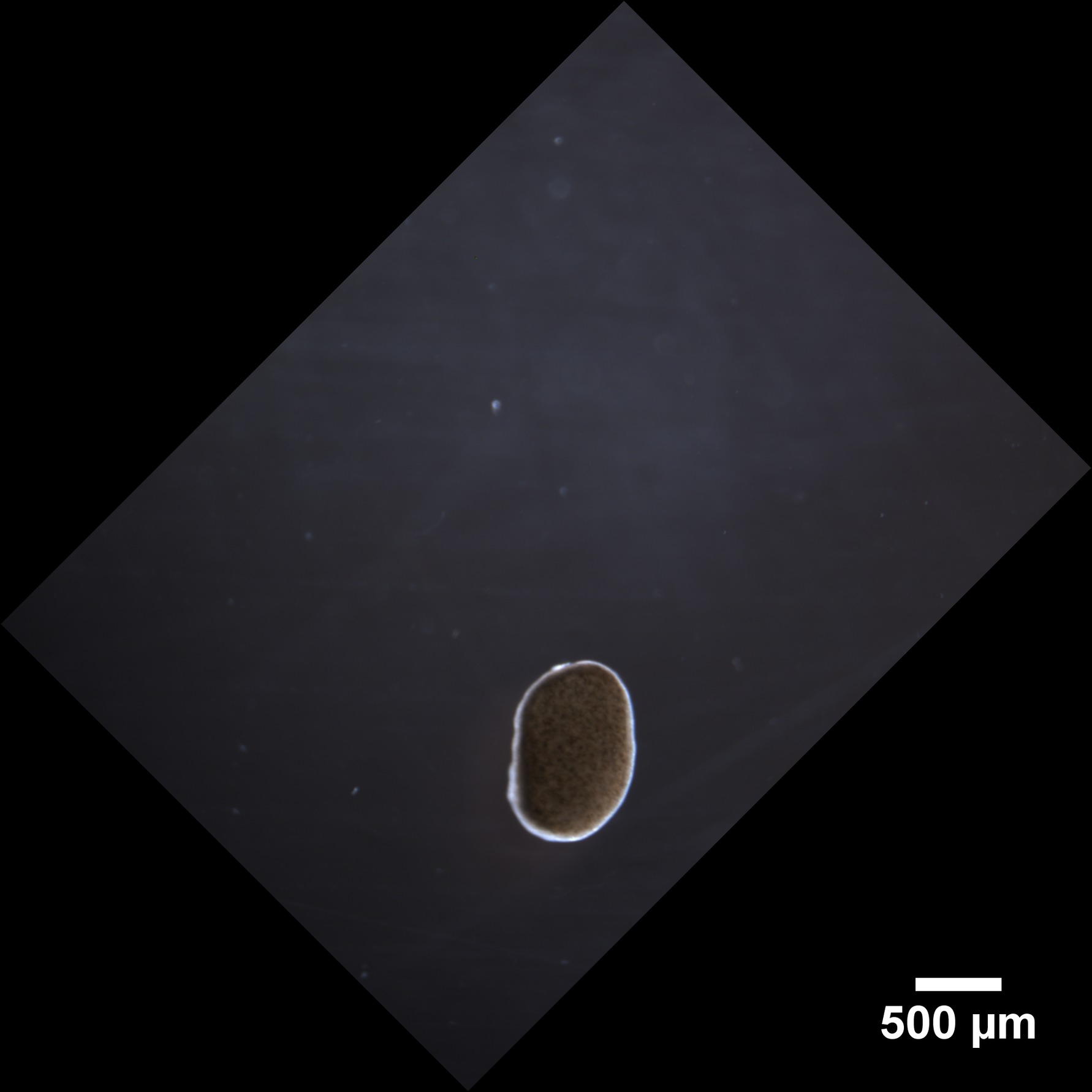

Supplement: S1 Dataset — This dataset contains brightfield image and corresponding synapsin stains for the VNC-free and VNC-containing small fragment cutting scenarios shown in Fig 6. Each image is labeled in the format “x_dpc_Sample_y_tn.jpg”, where “x” represents the number of days post cutting and “y” the replicate number. (ZIP) [file pcbi.1006904.s016.zip › smallfragments/VNC-containing/Brightfield_images/1 dpc_Sample 5_tn.jpg]

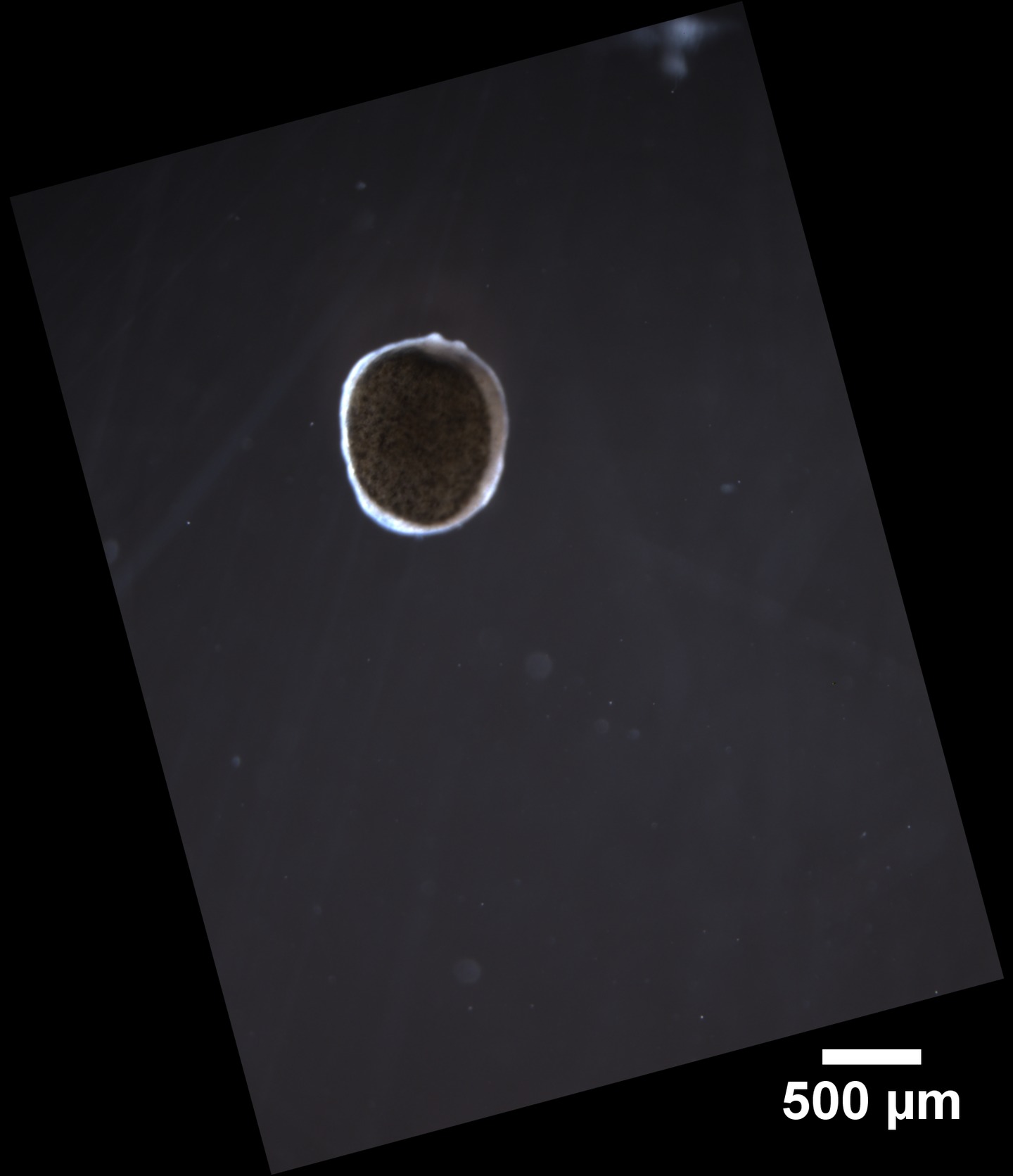

Supplement: S1 Dataset — This dataset contains brightfield image and corresponding synapsin stains for the VNC-free and VNC-containing small fragment cutting scenarios shown in Fig 6. Each image is labeled in the format “x_dpc_Sample_y_tn.jpg”, where “x” represents the number of days post cutting and “y” the replicate number. (ZIP) [file pcbi.1006904.s016.zip › smallfragments/VNC-containing/Brightfield_images/1 dpc_Sample 6_tn.jpg]

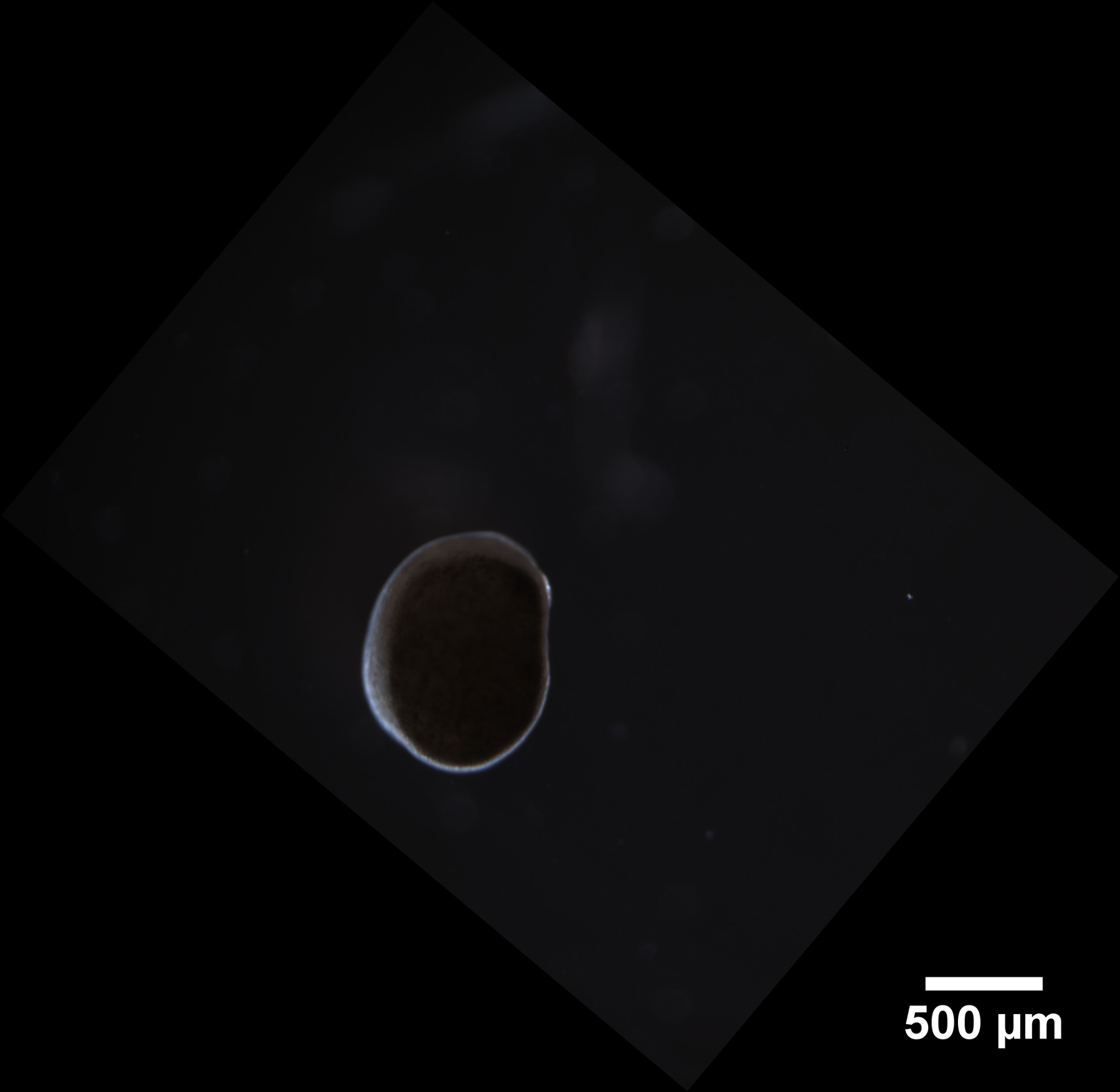

Supplement: S1 Dataset — This dataset contains brightfield image and corresponding synapsin stains for the VNC-free and VNC-containing small fragment cutting scenarios shown in Fig 6. Each image is labeled in the format “x_dpc_Sample_y_tn.jpg”, where “x” represents the number of days post cutting and “y” the replicate number. (ZIP) [file pcbi.1006904.s016.zip › smallfragments/VNC-containing/Brightfield_images/2 dpc_Sample 1_tn.jpg]

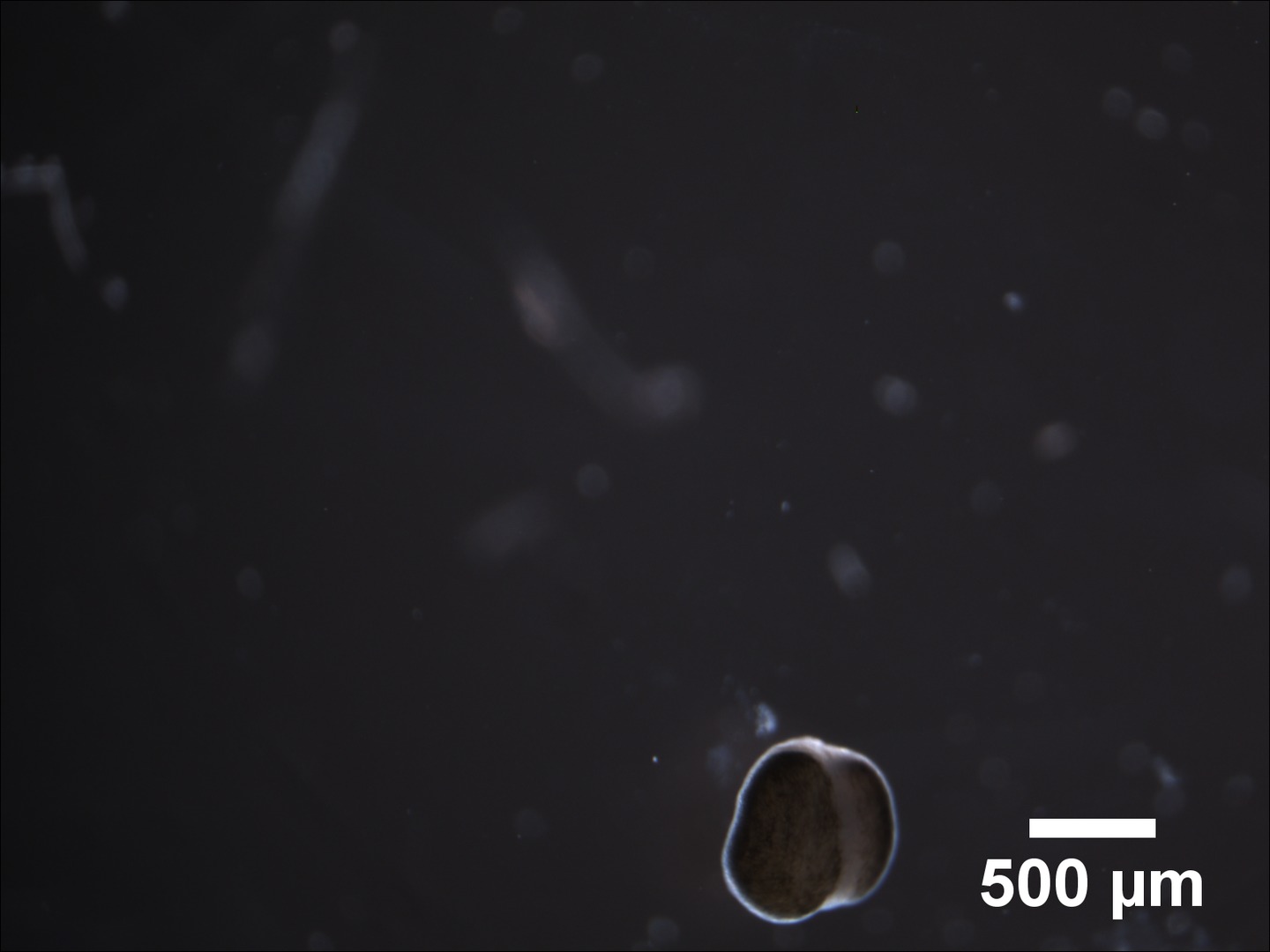

Supplement: S1 Dataset — This dataset contains brightfield image and corresponding synapsin stains for the VNC-free and VNC-containing small fragment cutting scenarios shown in Fig 6. Each image is labeled in the format “x_dpc_Sample_y_tn.jpg”, where “x” represents the number of days post cutting and “y” the replicate number. (ZIP) [file pcbi.1006904.s016.zip › smallfragments/VNC-containing/Brightfield_images/2 dpc_Sample 2_tn.jpg]

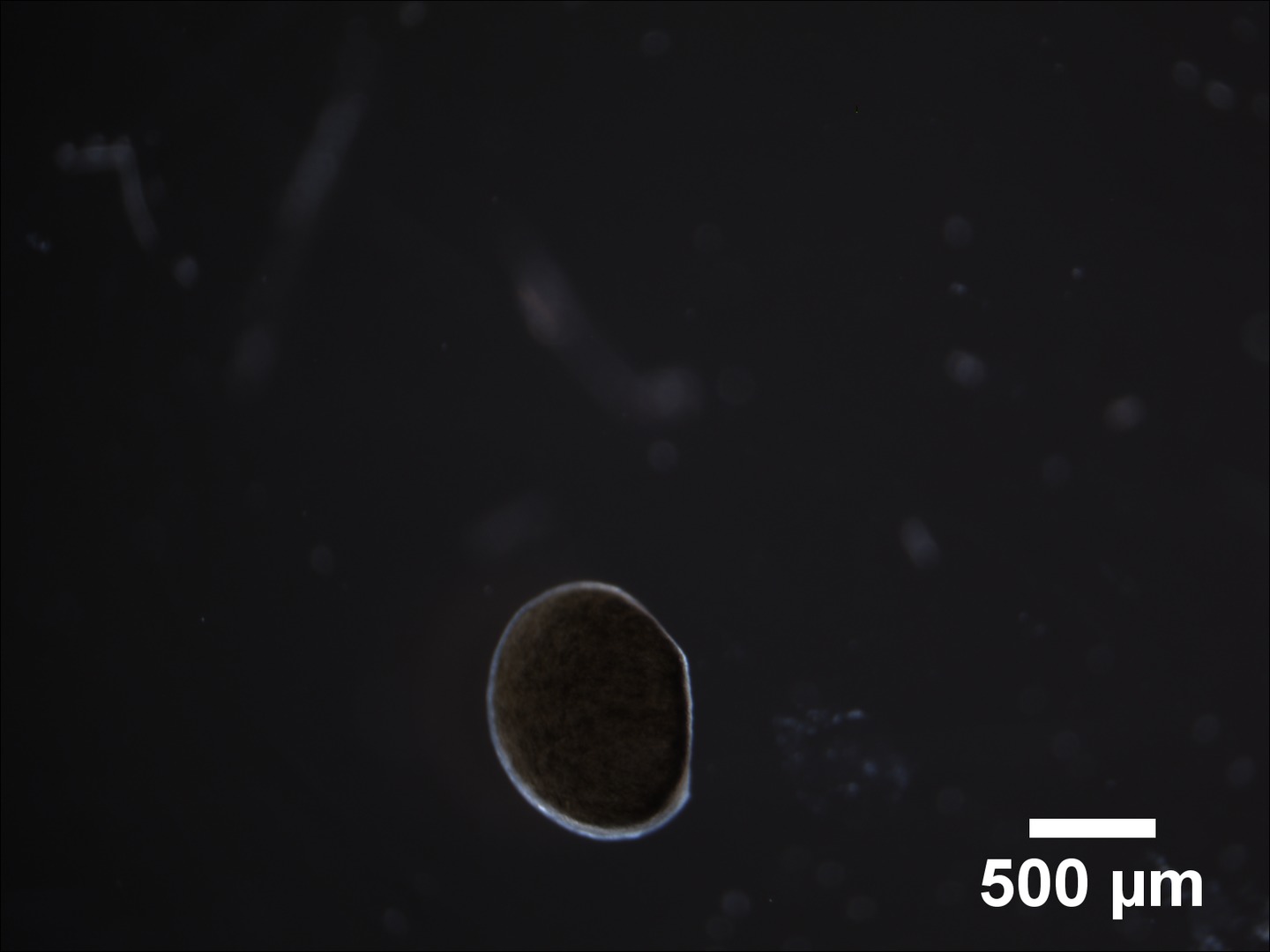

Supplement: S1 Dataset — This dataset contains brightfield image and corresponding synapsin stains for the VNC-free and VNC-containing small fragment cutting scenarios shown in Fig 6. Each image is labeled in the format “x_dpc_Sample_y_tn.jpg”, where “x” represents the number of days post cutting and “y” the replicate number. (ZIP) [file pcbi.1006904.s016.zip › smallfragments/VNC-containing/Brightfield_images/2 dpc_Sample 3_tn.jpg]

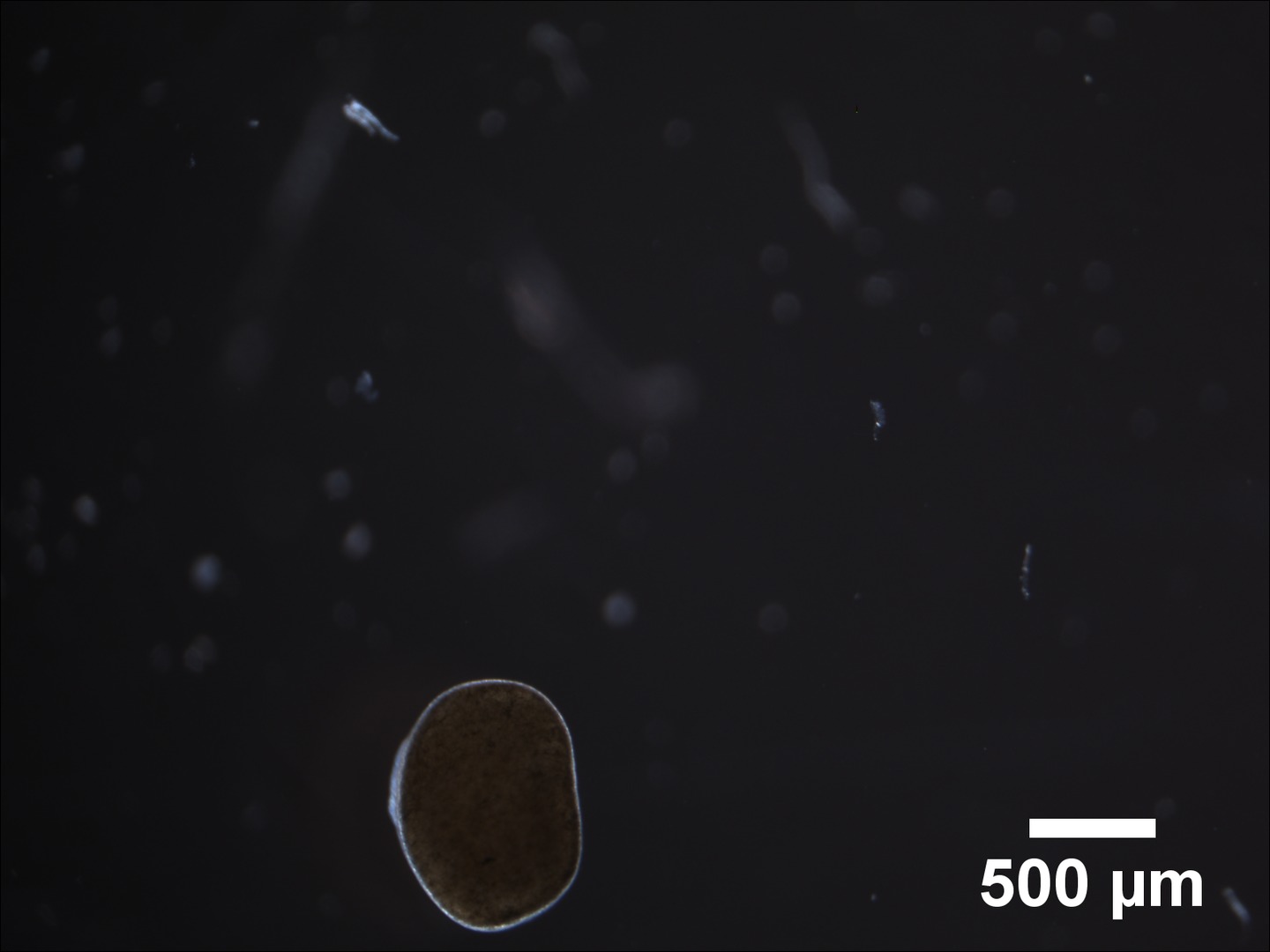

Supplement: S1 Dataset — This dataset contains brightfield image and corresponding synapsin stains for the VNC-free and VNC-containing small fragment cutting scenarios shown in Fig 6. Each image is labeled in the format “x_dpc_Sample_y_tn.jpg”, where “x” represents the number of days post cutting and “y” the replicate number. (ZIP) [file pcbi.1006904.s016.zip › smallfragments/VNC-containing/Brightfield_images/2 dpc_Sample 4_tn.jpg]

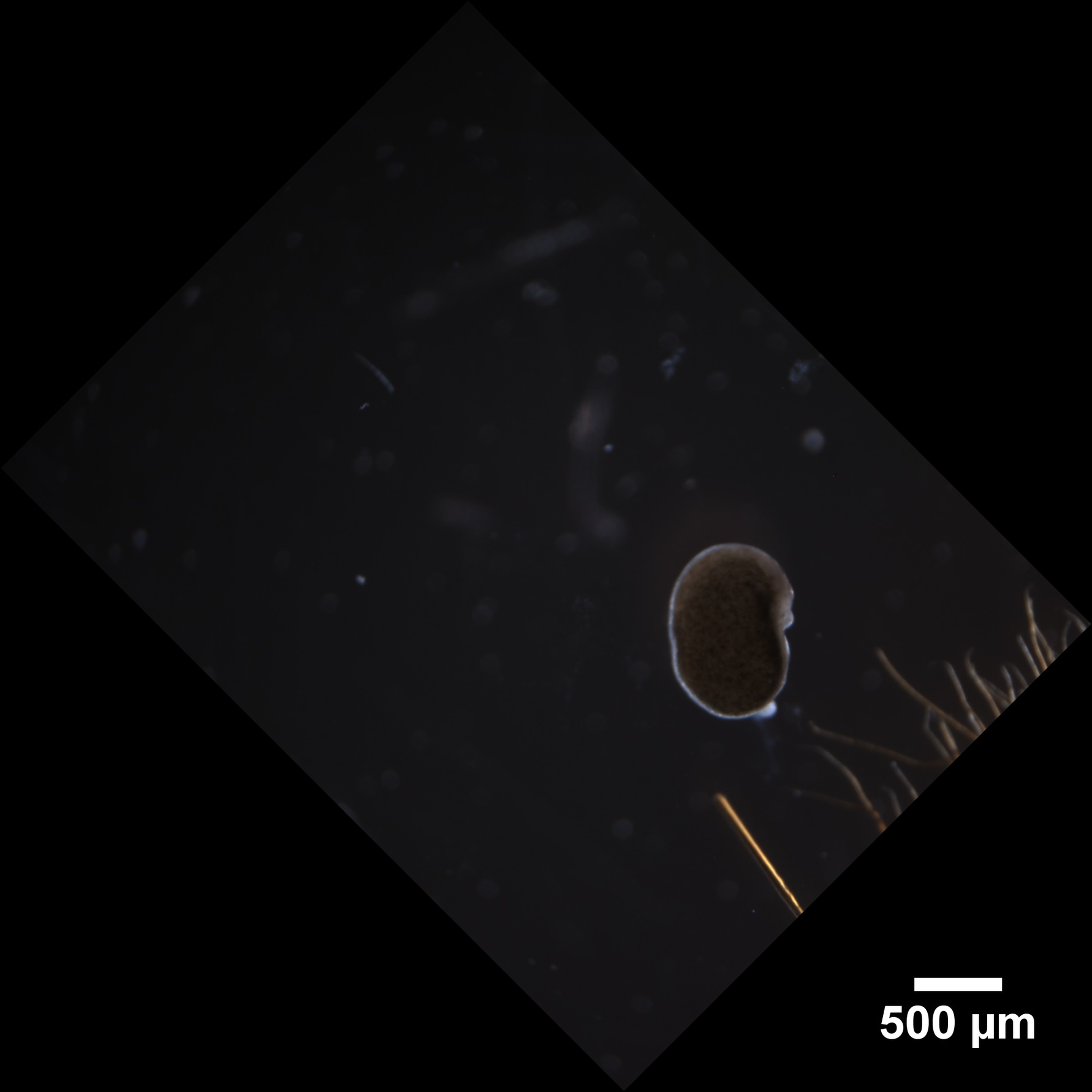

Supplement: S1 Dataset — This dataset contains brightfield image and corresponding synapsin stains for the VNC-free and VNC-containing small fragment cutting scenarios shown in Fig 6. Each image is labeled in the format “x_dpc_Sample_y_tn.jpg”, where “x” represents the number of days post cutting and “y” the replicate number. (ZIP) [file pcbi.1006904.s016.zip › smallfragments/VNC-containing/Brightfield_images/2 dpc_Sample 5_tn.jpg]

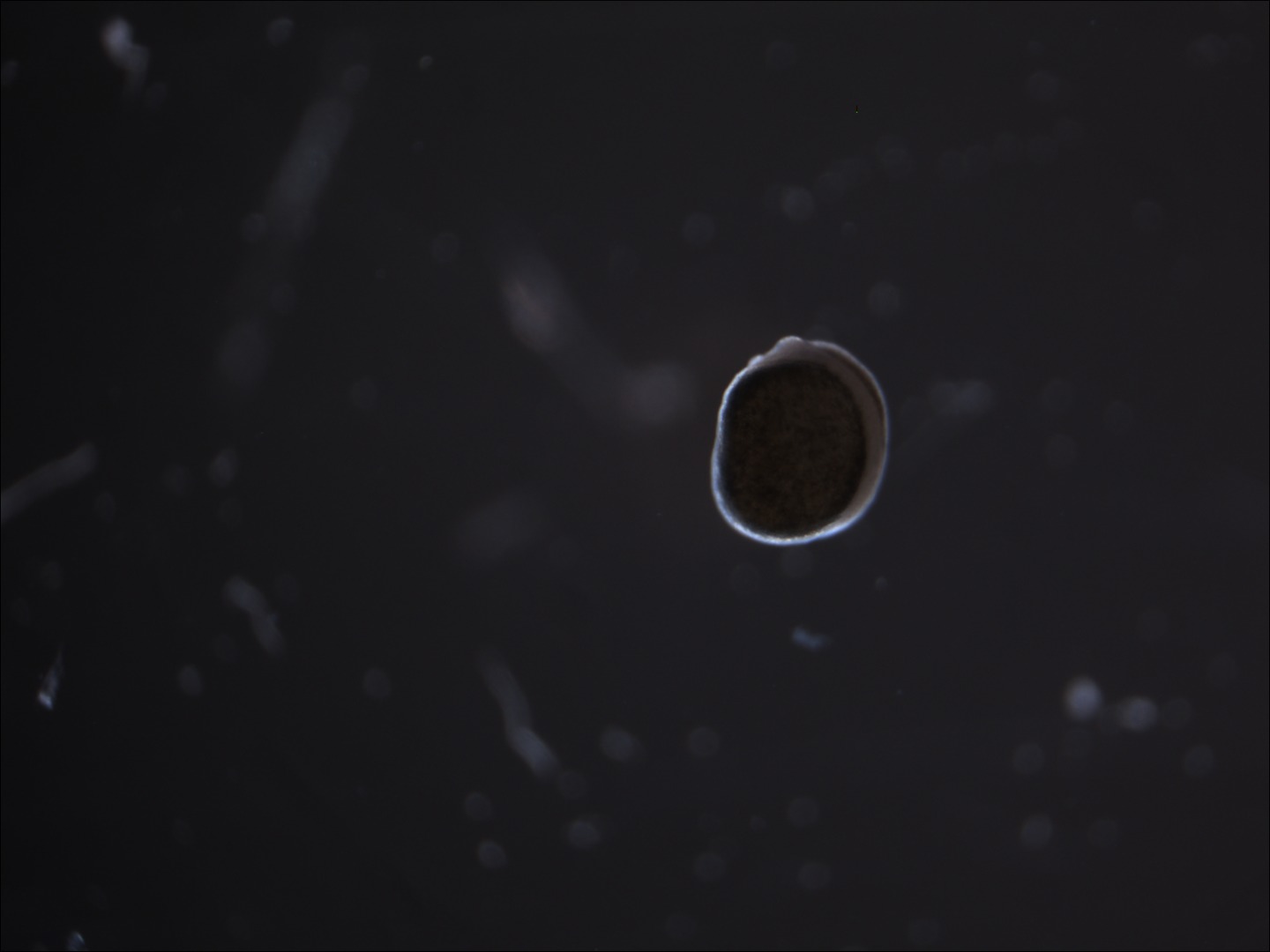

Supplement: S1 Dataset — This dataset contains brightfield image and corresponding synapsin stains for the VNC-free and VNC-containing small fragment cutting scenarios shown in Fig 6. Each image is labeled in the format “x_dpc_Sample_y_tn.jpg”, where “x” represents the number of days post cutting and “y” the replicate number. (ZIP) [file pcbi.1006904.s016.zip › smallfragments/VNC-containing/Brightfield_images/2 dpc_Sample 6_tn.jpg]

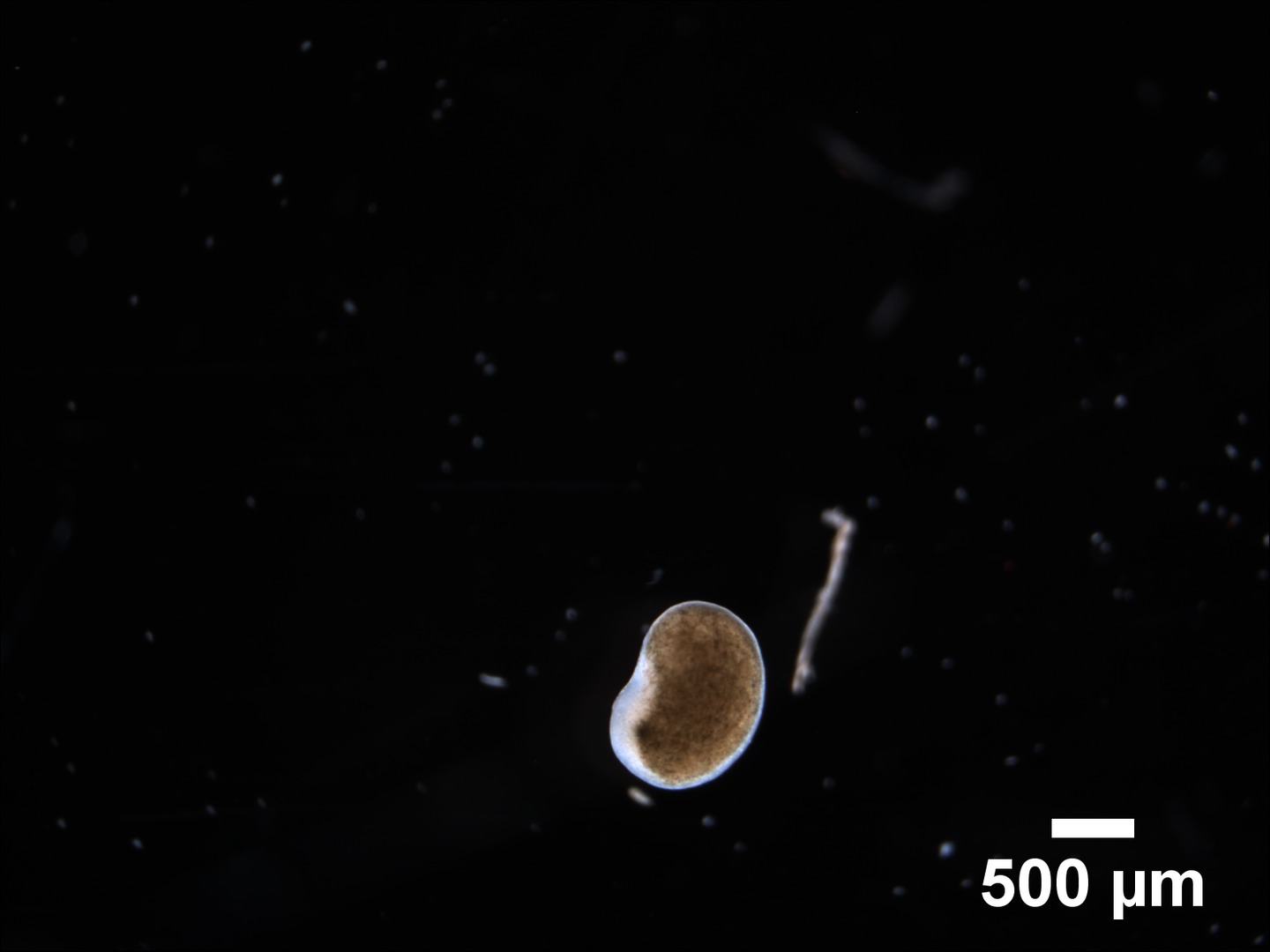

Supplement: S1 Dataset — This dataset contains brightfield image and corresponding synapsin stains for the VNC-free and VNC-containing small fragment cutting scenarios shown in Fig 6. Each image is labeled in the format “x_dpc_Sample_y_tn.jpg”, where “x” represents the number of days post cutting and “y” the replicate number. (ZIP) [file pcbi.1006904.s016.zip › smallfragments/VNC-containing/Brightfield_images/3 dpc_Sample 1_tn.jpg]

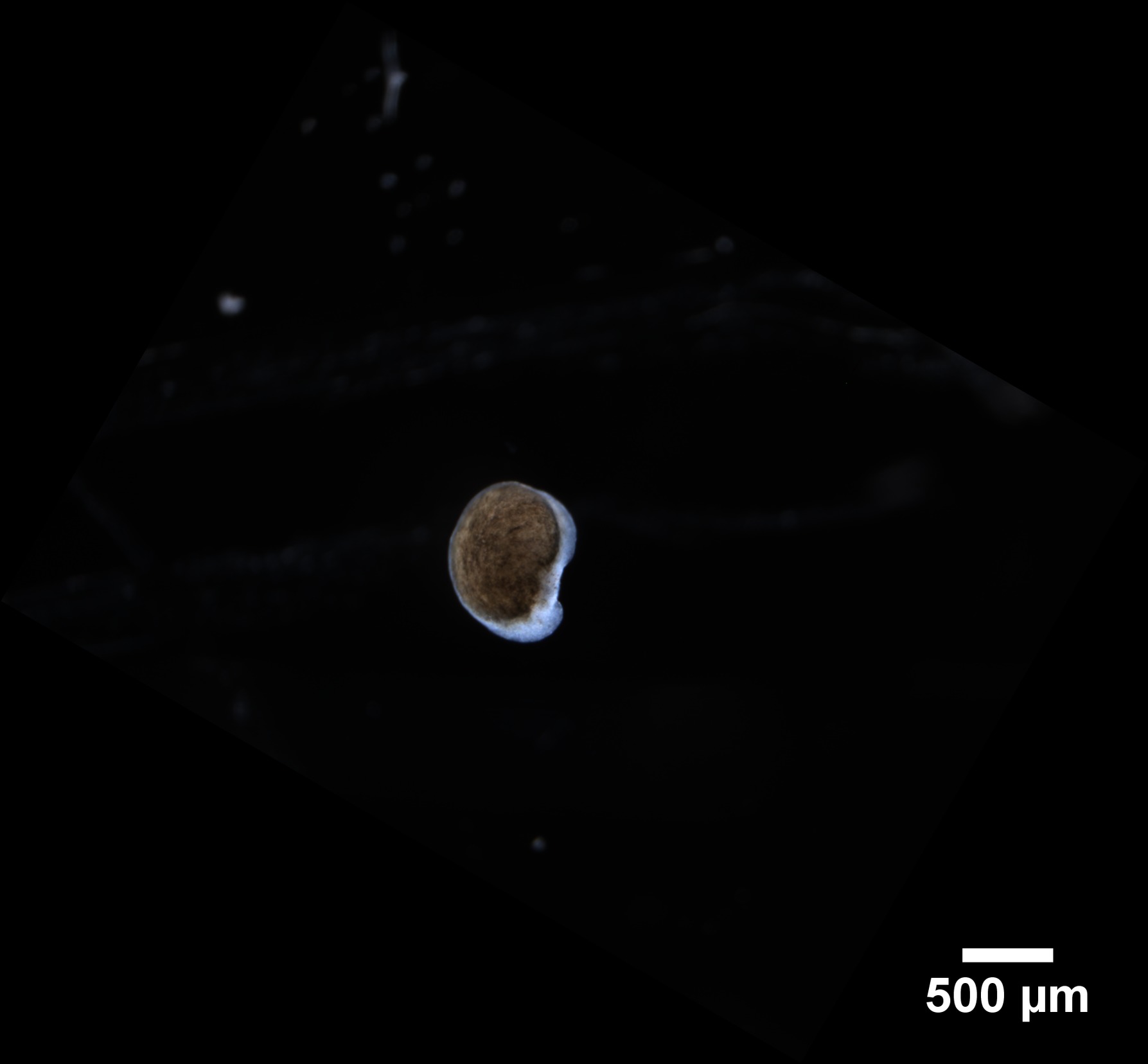

Supplement: S1 Dataset — This dataset contains brightfield image and corresponding synapsin stains for the VNC-free and VNC-containing small fragment cutting scenarios shown in Fig 6. Each image is labeled in the format “x_dpc_Sample_y_tn.jpg”, where “x” represents the number of days post cutting and “y” the replicate number. (ZIP) [file pcbi.1006904.s016.zip › smallfragments/VNC-containing/Brightfield_images/3 dpc_Sample 2_tn.jpg]

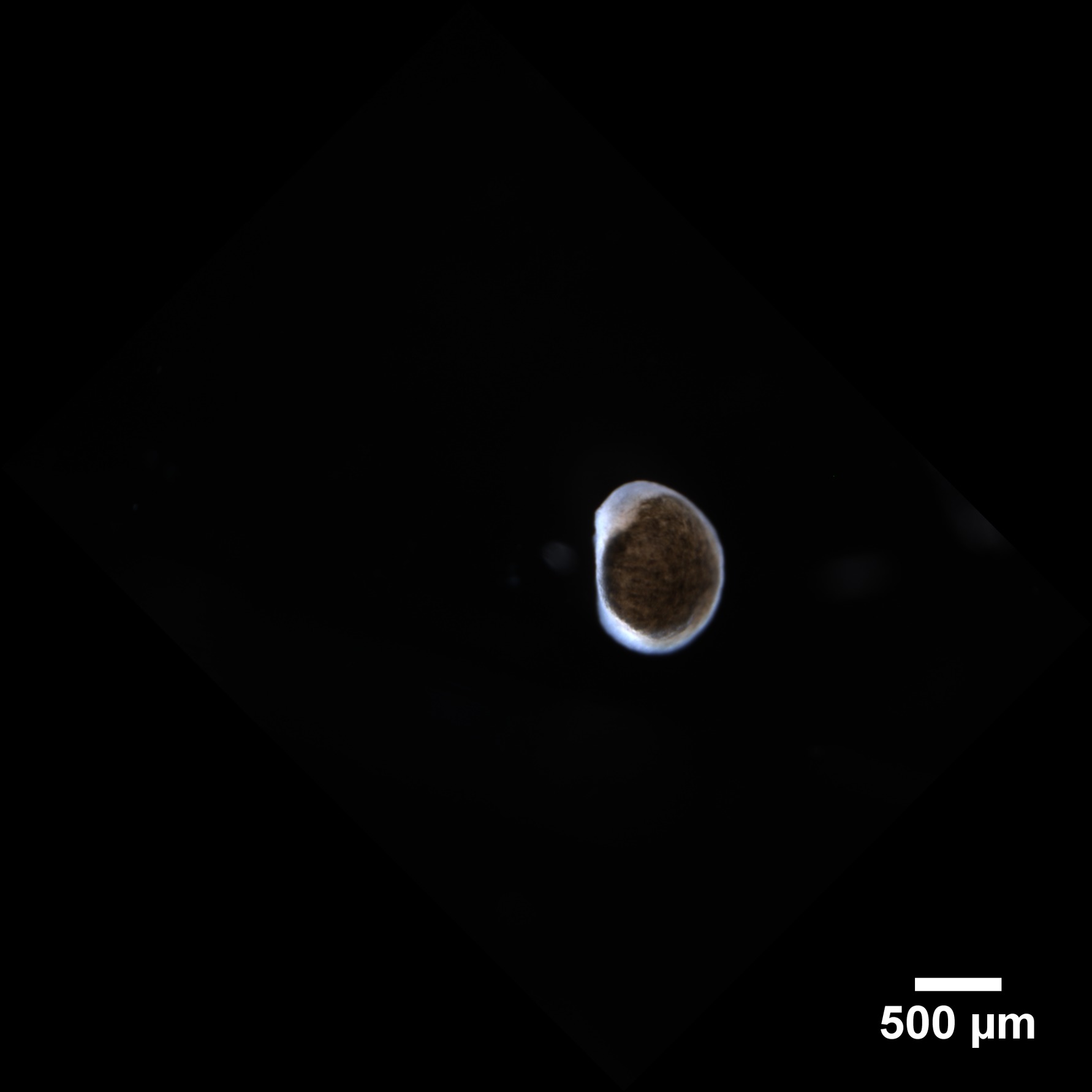

Supplement: S1 Dataset — This dataset contains brightfield image and corresponding synapsin stains for the VNC-free and VNC-containing small fragment cutting scenarios shown in Fig 6. Each image is labeled in the format “x_dpc_Sample_y_tn.jpg”, where “x” represents the number of days post cutting and “y” the replicate number. (ZIP) [file pcbi.1006904.s016.zip › smallfragments/VNC-containing/Brightfield_images/3 dpc_Sample 3_tn.jpg]

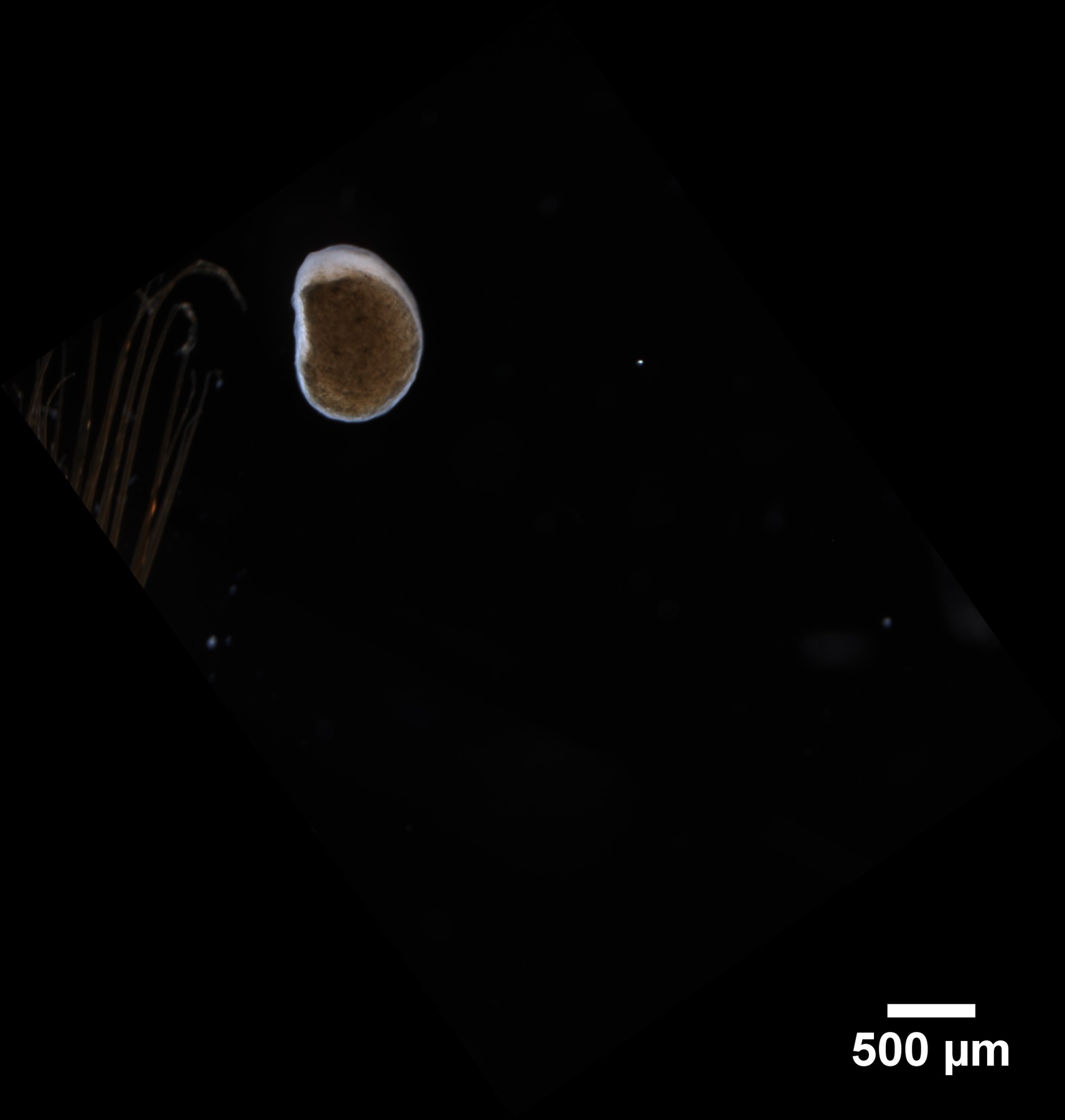

Supplement: S1 Dataset — This dataset contains brightfield image and corresponding synapsin stains for the VNC-free and VNC-containing small fragment cutting scenarios shown in Fig 6. Each image is labeled in the format “x_dpc_Sample_y_tn.jpg”, where “x” represents the number of days post cutting and “y” the replicate number. (ZIP) [file pcbi.1006904.s016.zip › smallfragments/VNC-containing/Brightfield_images/3 dpc_Sample 4_tn.jpg]

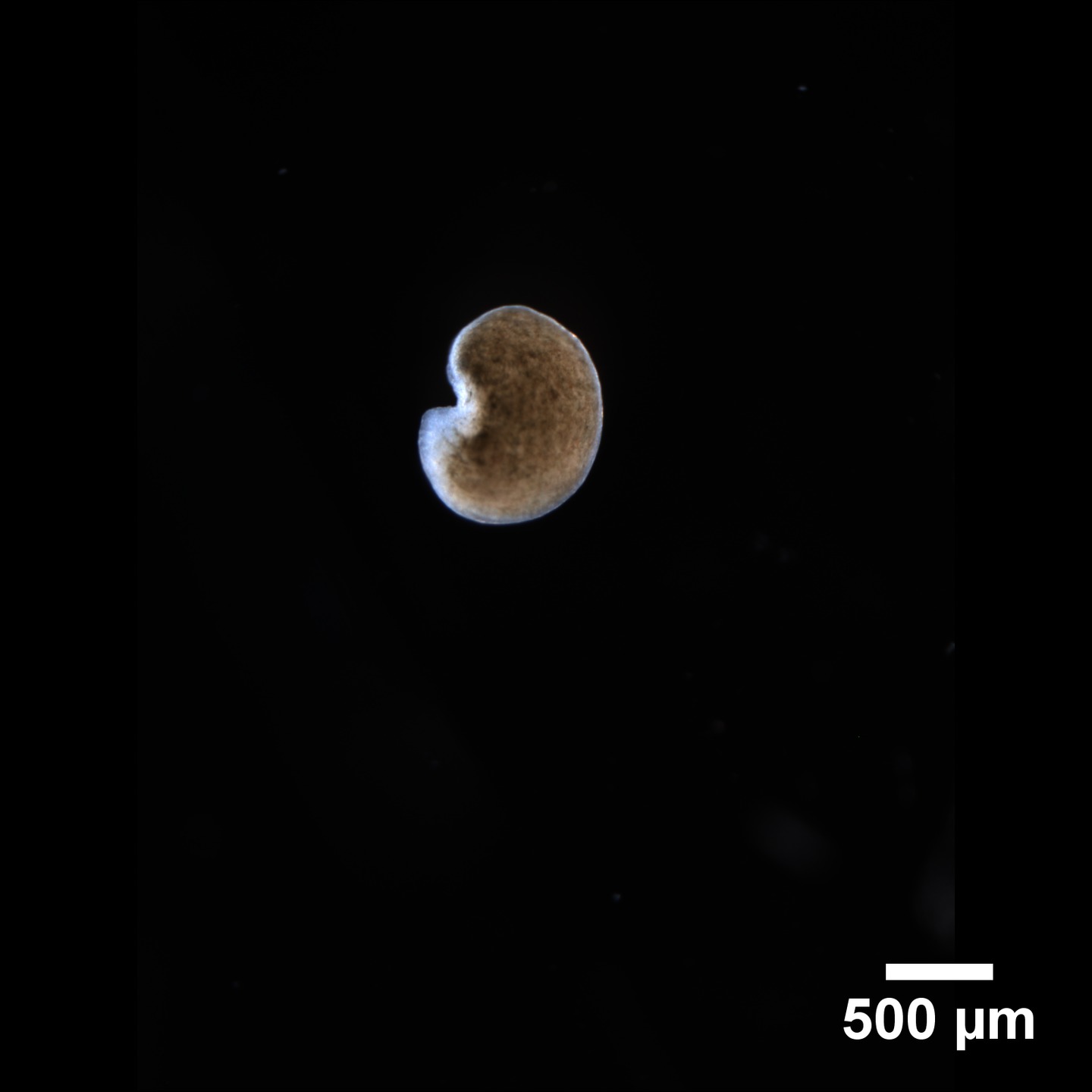

Supplement: S1 Dataset — This dataset contains brightfield image and corresponding synapsin stains for the VNC-free and VNC-containing small fragment cutting scenarios shown in Fig 6. Each image is labeled in the format “x_dpc_Sample_y_tn.jpg”, where “x” represents the number of days post cutting and “y” the replicate number. (ZIP) [file pcbi.1006904.s016.zip › smallfragments/VNC-containing/Brightfield_images/3 dpc_Sample 5_tn.jpg]

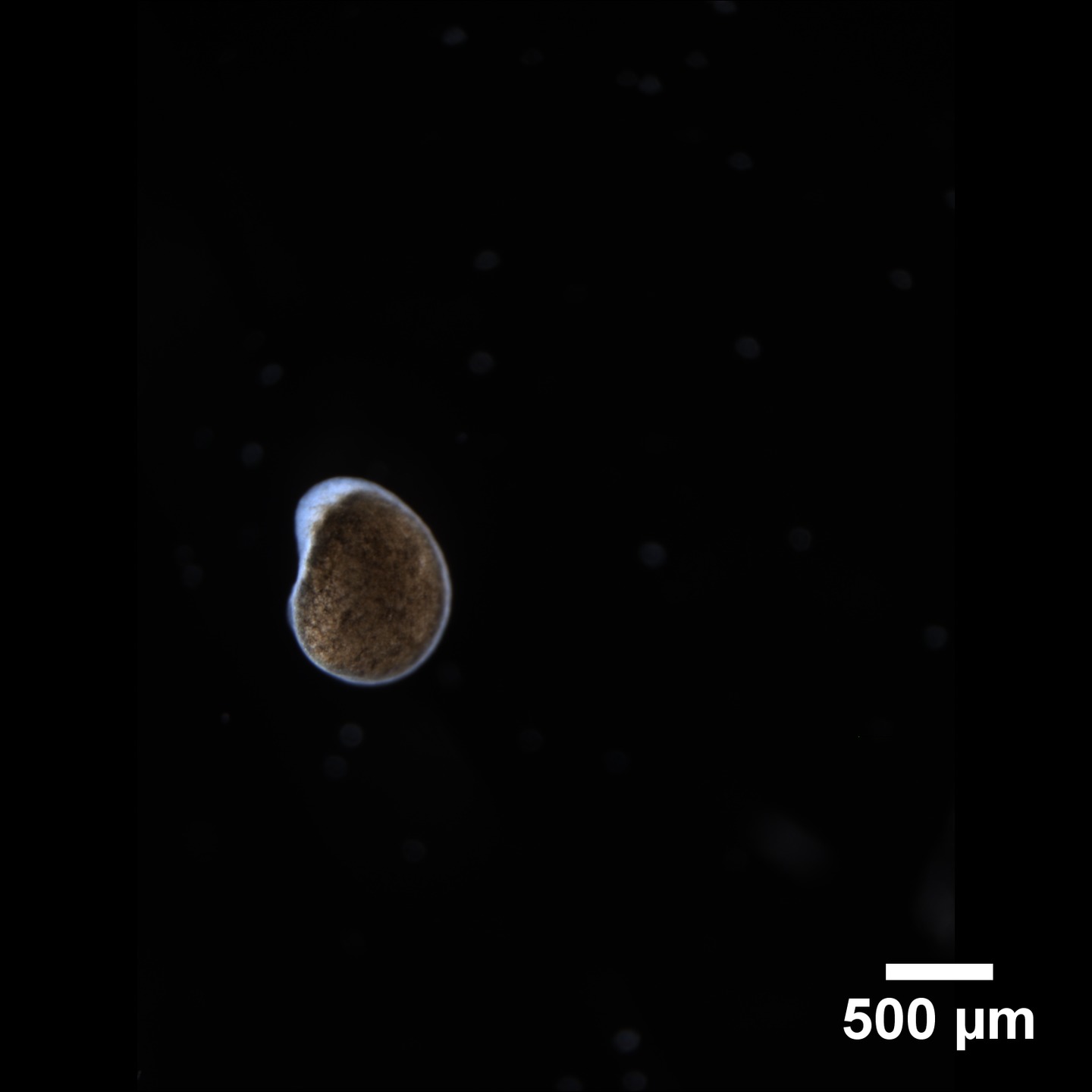

Supplement: S1 Dataset — This dataset contains brightfield image and corresponding synapsin stains for the VNC-free and VNC-containing small fragment cutting scenarios shown in Fig 6. Each image is labeled in the format “x_dpc_Sample_y_tn.jpg”, where “x” represents the number of days post cutting and “y” the replicate number. (ZIP) [file pcbi.1006904.s016.zip › smallfragments/VNC-containing/Brightfield_images/3 dpc_Sample 6_tn.jpg]

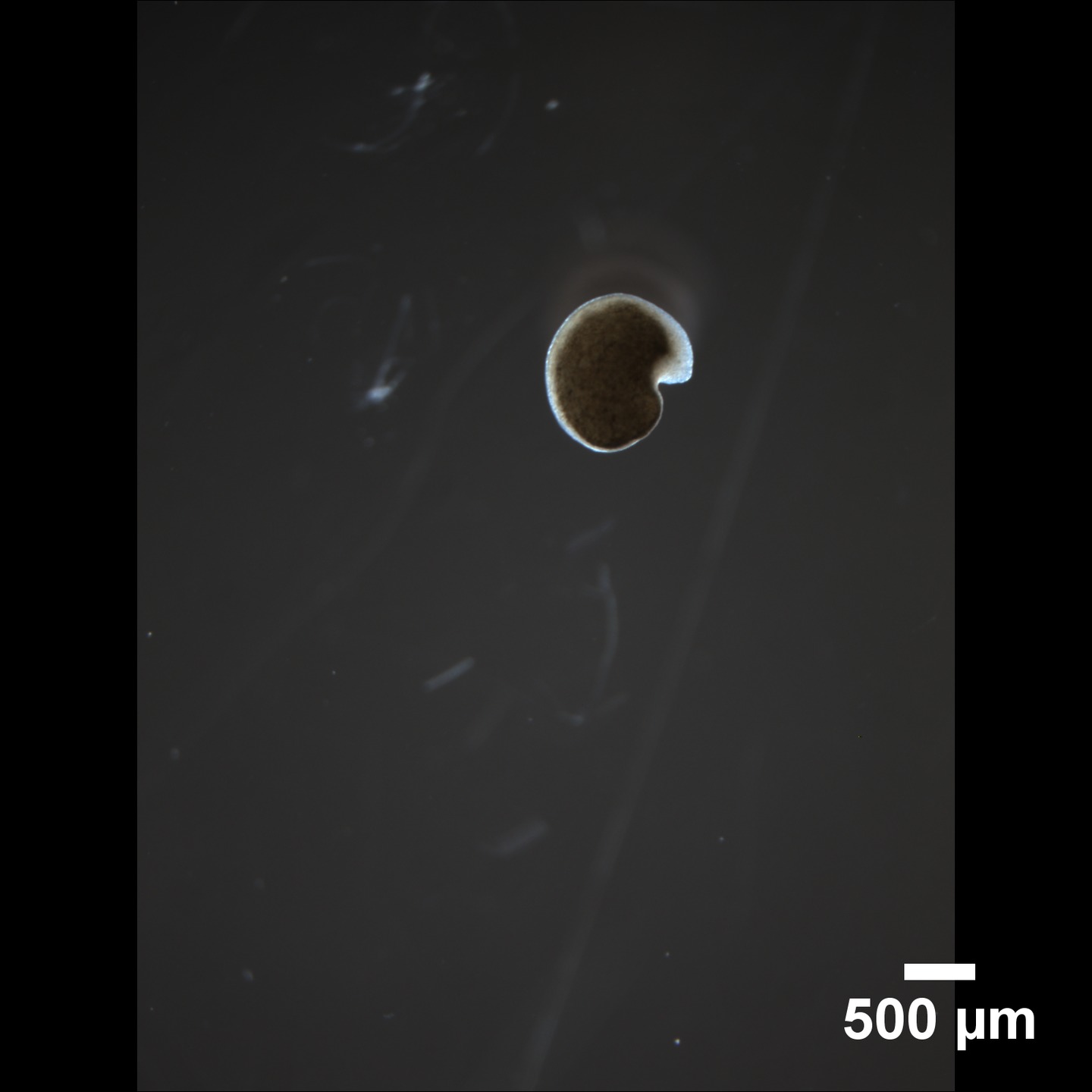

Supplement: S1 Dataset — This dataset contains brightfield image and corresponding synapsin stains for the VNC-free and VNC-containing small fragment cutting scenarios shown in Fig 6. Each image is labeled in the format “x_dpc_Sample_y_tn.jpg”, where “x” represents the number of days post cutting and “y” the replicate number. (ZIP) [file pcbi.1006904.s016.zip › smallfragments/VNC-containing/Brightfield_images/4 dpc_Sample 1_tn.jpg]

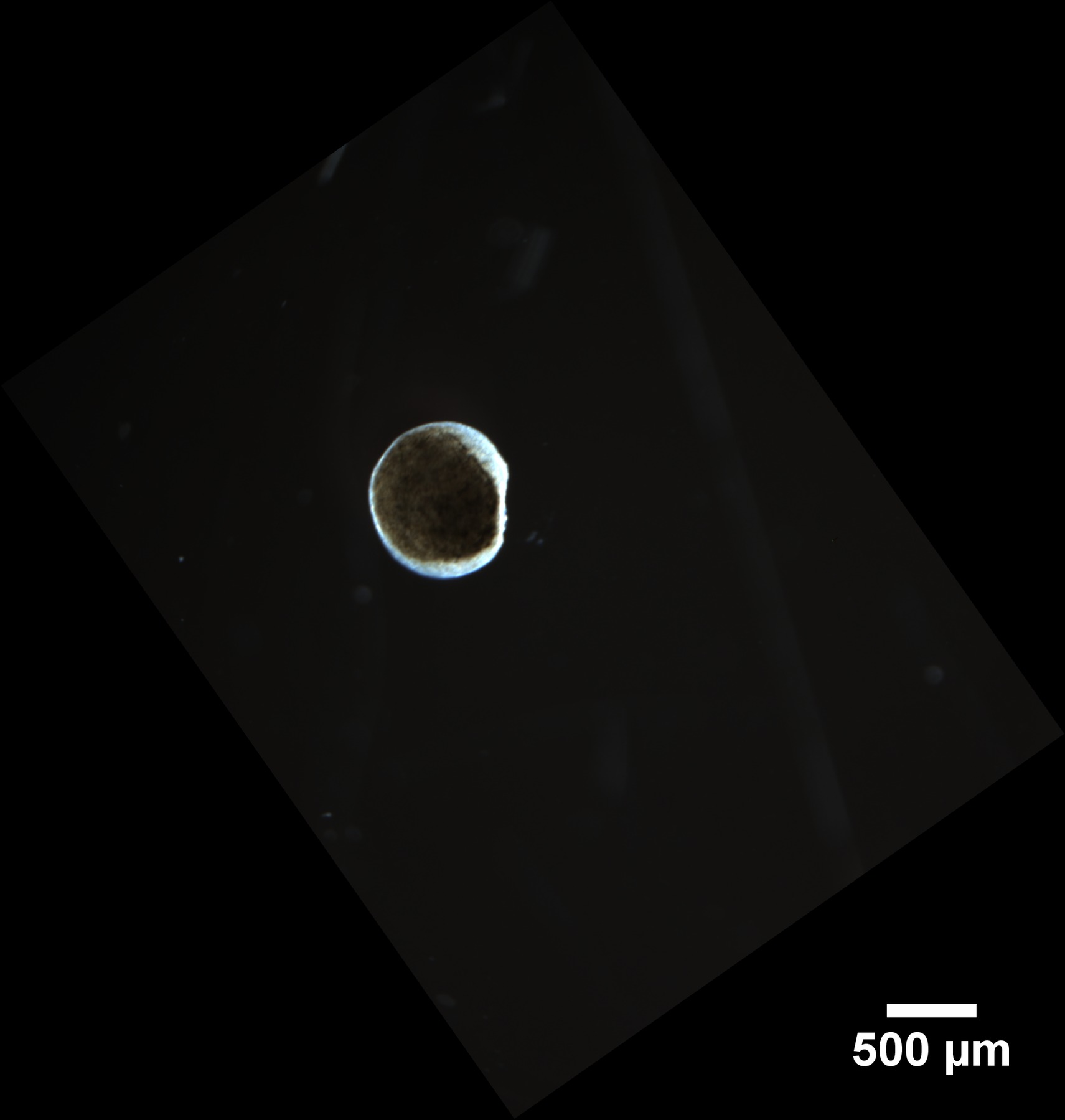

Supplement: S1 Dataset — This dataset contains brightfield image and corresponding synapsin stains for the VNC-free and VNC-containing small fragment cutting scenarios shown in Fig 6. Each image is labeled in the format “x_dpc_Sample_y_tn.jpg”, where “x” represents the number of days post cutting and “y” the replicate number. (ZIP) [file pcbi.1006904.s016.zip › smallfragments/VNC-containing/Brightfield_images/4 dpc_Sample 2_tn.jpg]

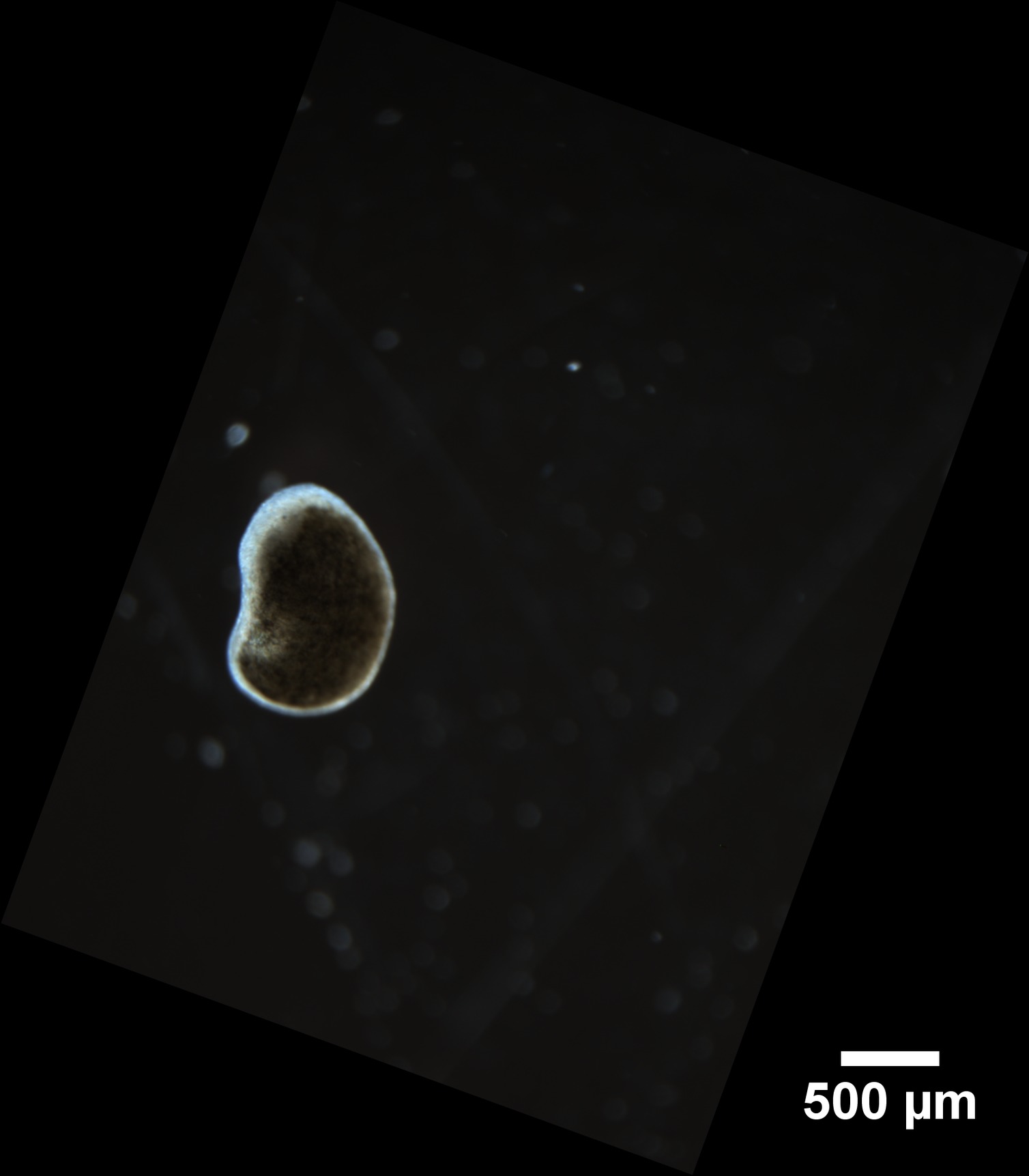

Supplement: S1 Dataset — This dataset contains brightfield image and corresponding synapsin stains for the VNC-free and VNC-containing small fragment cutting scenarios shown in Fig 6. Each image is labeled in the format “x_dpc_Sample_y_tn.jpg”, where “x” represents the number of days post cutting and “y” the replicate number. (ZIP) [file pcbi.1006904.s016.zip › smallfragments/VNC-containing/Brightfield_images/4 dpc_Sample 3_tn.jpg]

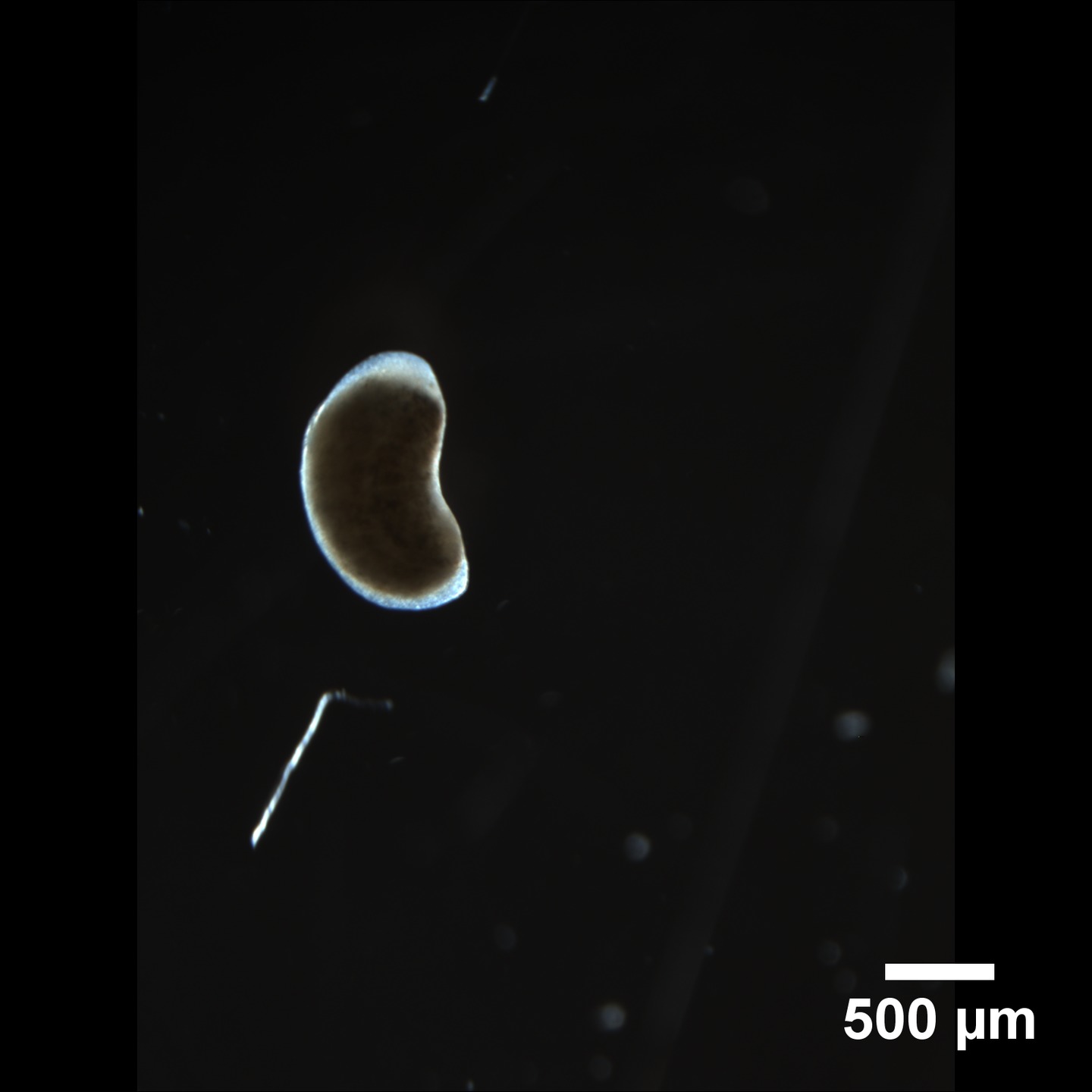

Supplement: S1 Dataset — This dataset contains brightfield image and corresponding synapsin stains for the VNC-free and VNC-containing small fragment cutting scenarios shown in Fig 6. Each image is labeled in the format “x_dpc_Sample_y_tn.jpg”, where “x” represents the number of days post cutting and “y” the replicate number. (ZIP) [file pcbi.1006904.s016.zip › smallfragments/VNC-containing/Brightfield_images/4 dpc_Sample 4_tn.jpg]

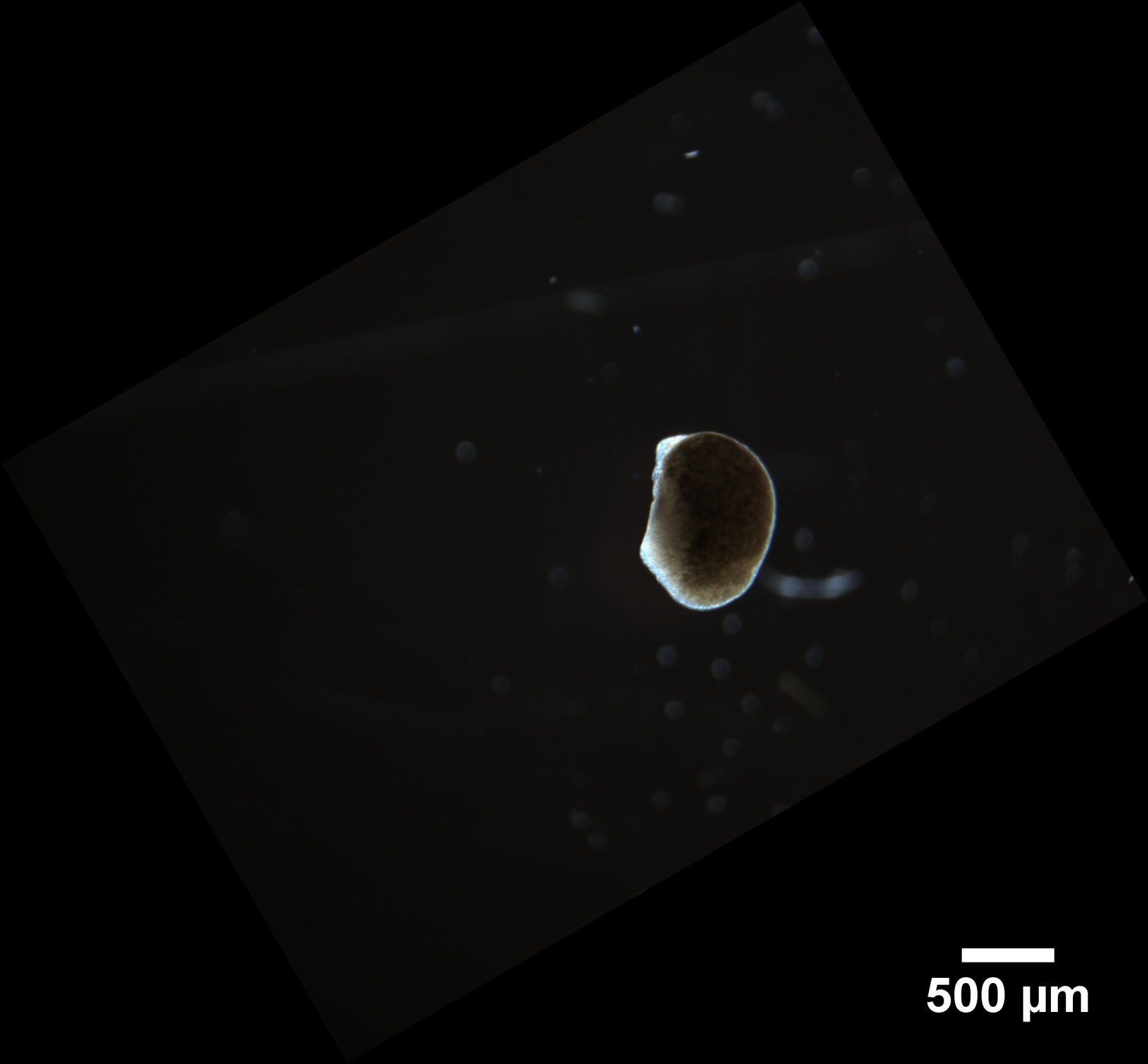

Supplement: S1 Dataset — This dataset contains brightfield image and corresponding synapsin stains for the VNC-free and VNC-containing small fragment cutting scenarios shown in Fig 6. Each image is labeled in the format “x_dpc_Sample_y_tn.jpg”, where “x” represents the number of days post cutting and “y” the replicate number. (ZIP) [file pcbi.1006904.s016.zip › smallfragments/VNC-containing/Brightfield_images/4 dpc_Sample 5_tn.jpg]

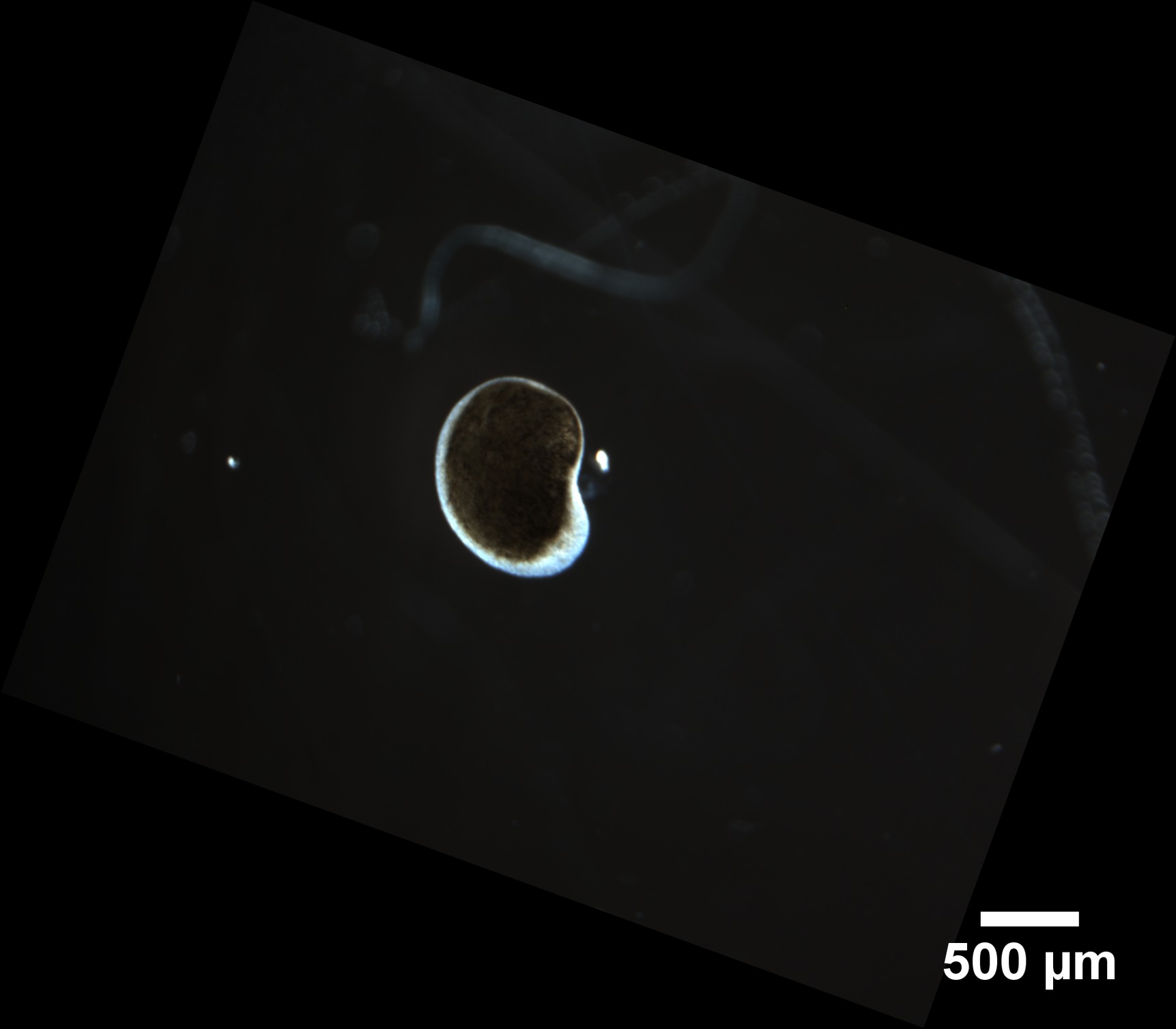

Supplement: S1 Dataset — This dataset contains brightfield image and corresponding synapsin stains for the VNC-free and VNC-containing small fragment cutting scenarios shown in Fig 6. Each image is labeled in the format “x_dpc_Sample_y_tn.jpg”, where “x” represents the number of days post cutting and “y” the replicate number. (ZIP) [file pcbi.1006904.s016.zip › smallfragments/VNC-containing/Brightfield_images/4 dpc_Sample 6_tn.jpg]

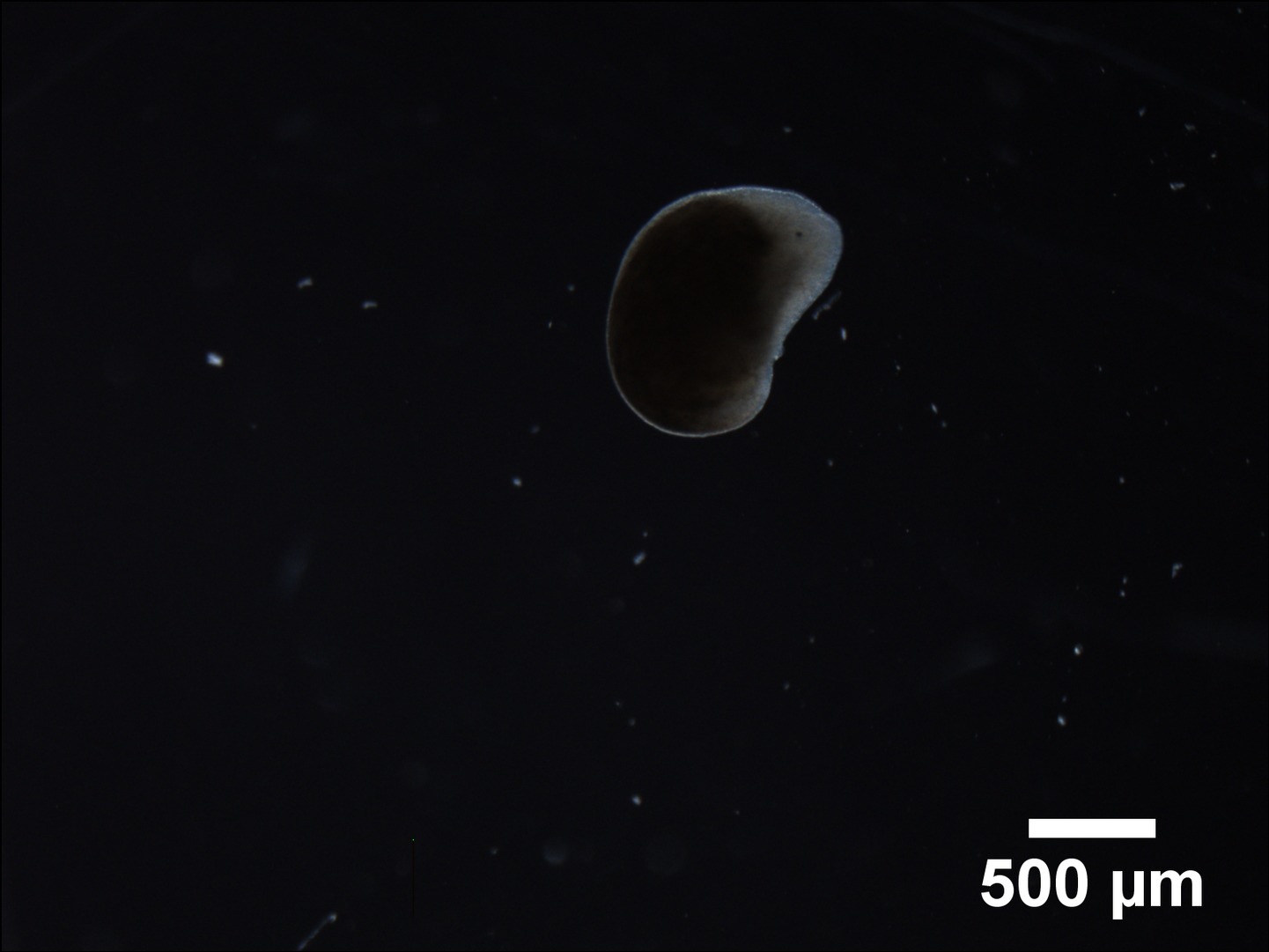

Supplement: S1 Dataset — This dataset contains brightfield image and corresponding synapsin stains for the VNC-free and VNC-containing small fragment cutting scenarios shown in Fig 6. Each image is labeled in the format “x_dpc_Sample_y_tn.jpg”, where “x” represents the number of days post cutting and “y” the replicate number. (ZIP) [file pcbi.1006904.s016.zip › smallfragments/VNC-containing/Brightfield_images/5 dpc_Sample 1_tn.jpg]

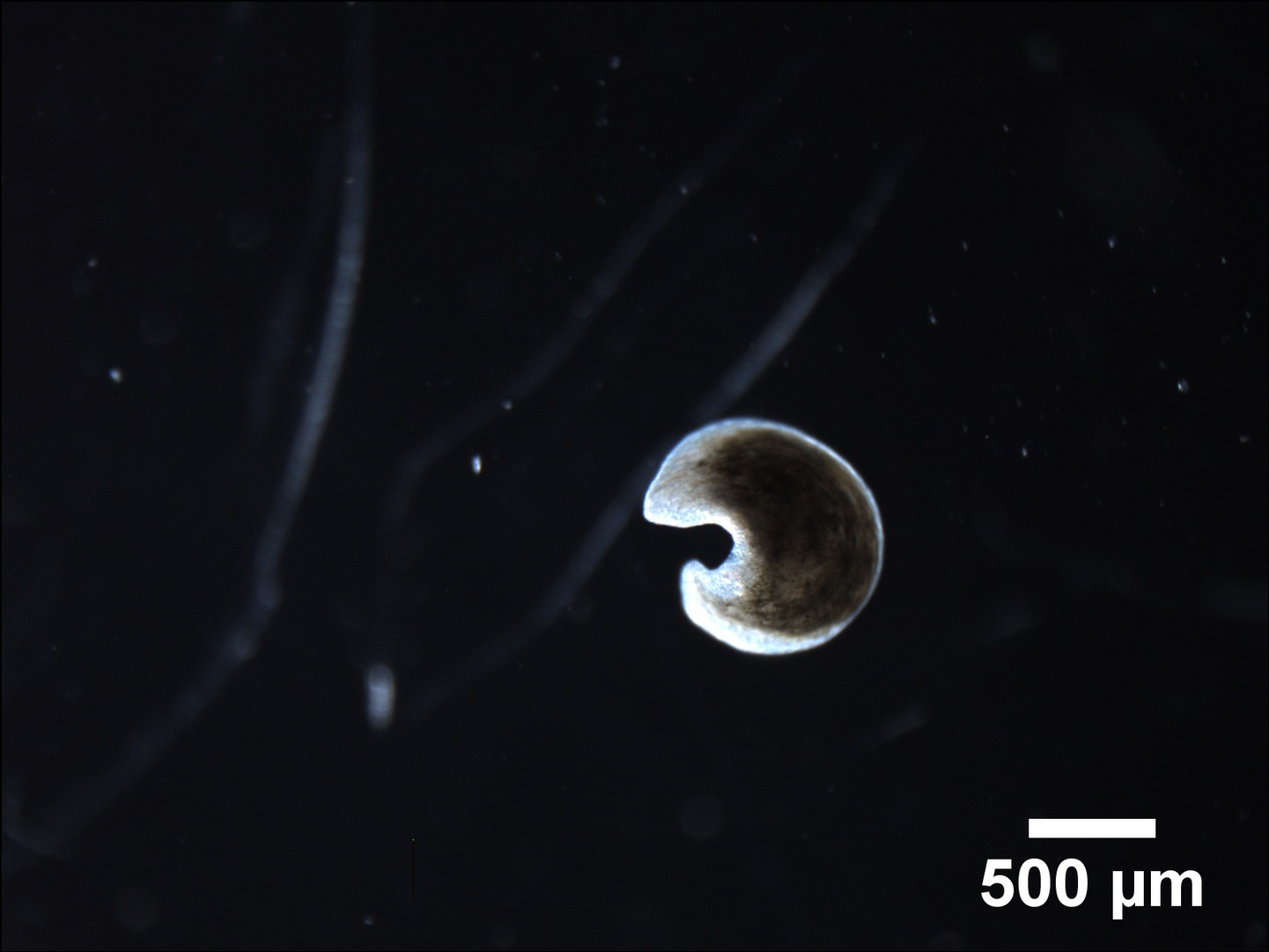

Supplement: S1 Dataset — This dataset contains brightfield image and corresponding synapsin stains for the VNC-free and VNC-containing small fragment cutting scenarios shown in Fig 6. Each image is labeled in the format “x_dpc_Sample_y_tn.jpg”, where “x” represents the number of days post cutting and “y” the replicate number. (ZIP) [file pcbi.1006904.s016.zip › smallfragments/VNC-containing/Brightfield_images/5 dpc_Sample 2_tn.jpg]

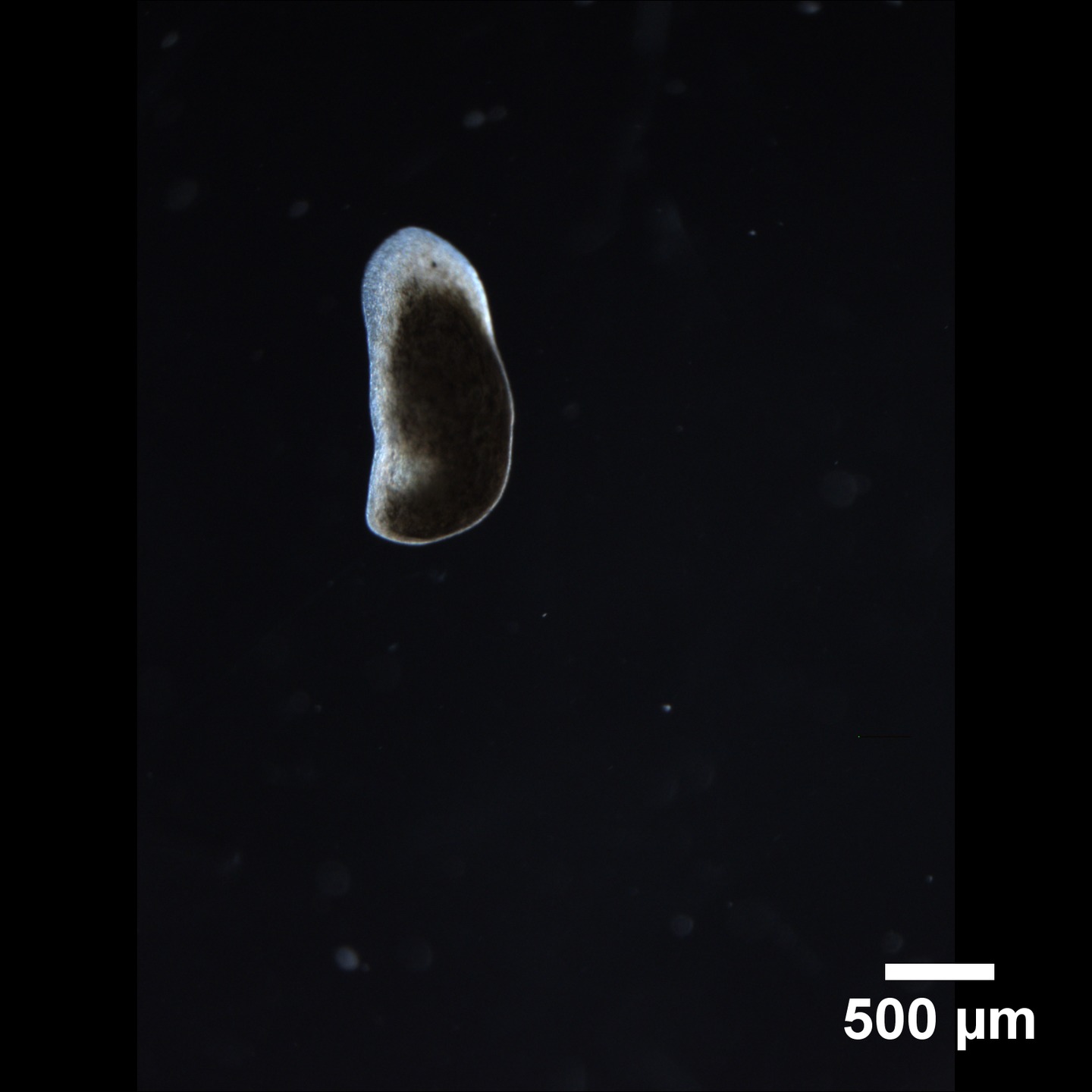

Supplement: S1 Dataset — This dataset contains brightfield image and corresponding synapsin stains for the VNC-free and VNC-containing small fragment cutting scenarios shown in Fig 6. Each image is labeled in the format “x_dpc_Sample_y_tn.jpg”, where “x” represents the number of days post cutting and “y” the replicate number. (ZIP) [file pcbi.1006904.s016.zip › smallfragments/VNC-containing/Brightfield_images/5 dpc_Sample 3_tn.jpg]

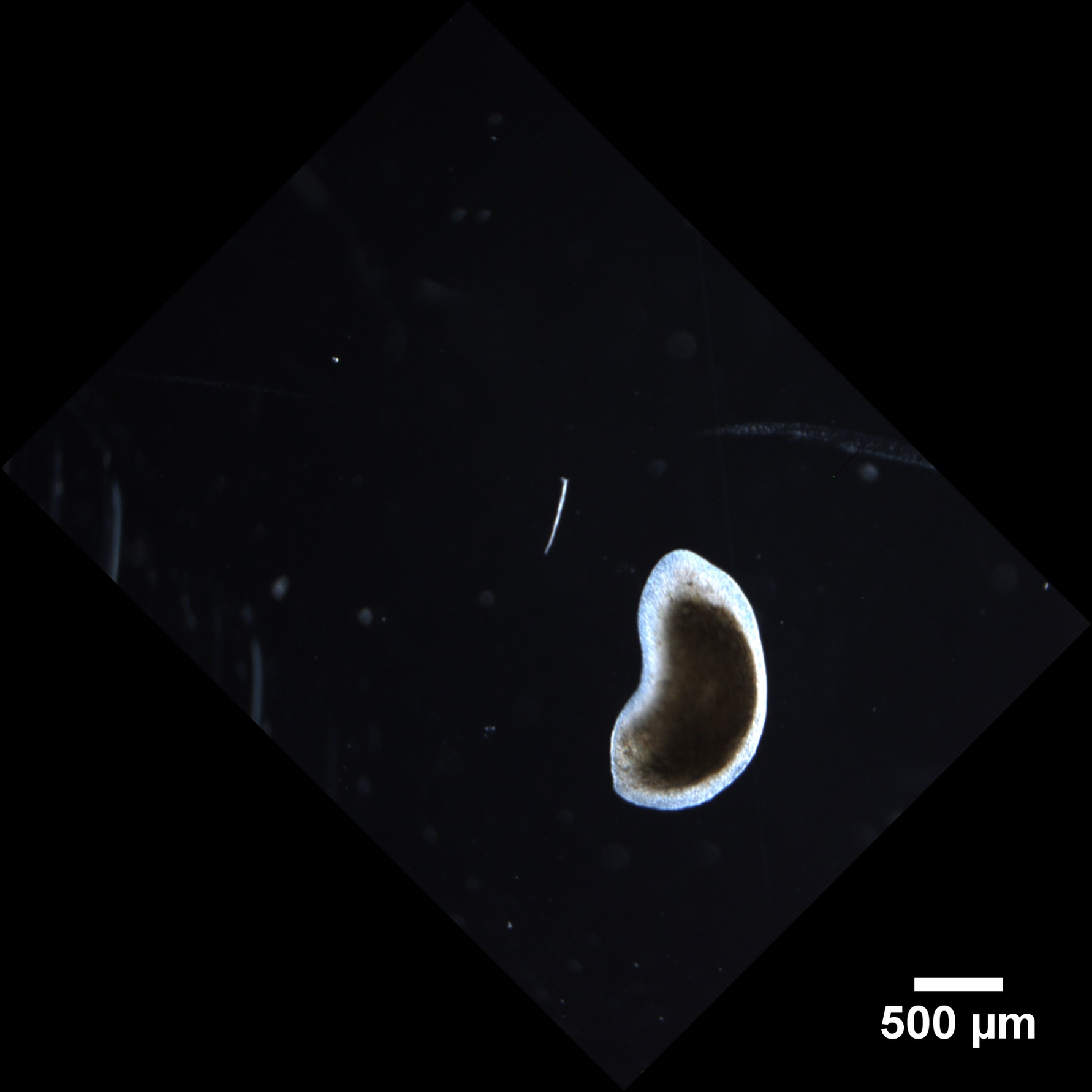

Supplement: S1 Dataset — This dataset contains brightfield image and corresponding synapsin stains for the VNC-free and VNC-containing small fragment cutting scenarios shown in Fig 6. Each image is labeled in the format “x_dpc_Sample_y_tn.jpg”, where “x” represents the number of days post cutting and “y” the replicate number. (ZIP) [file pcbi.1006904.s016.zip › smallfragments/VNC-containing/Brightfield_images/5 dpc_Sample 4_tn.jpg]

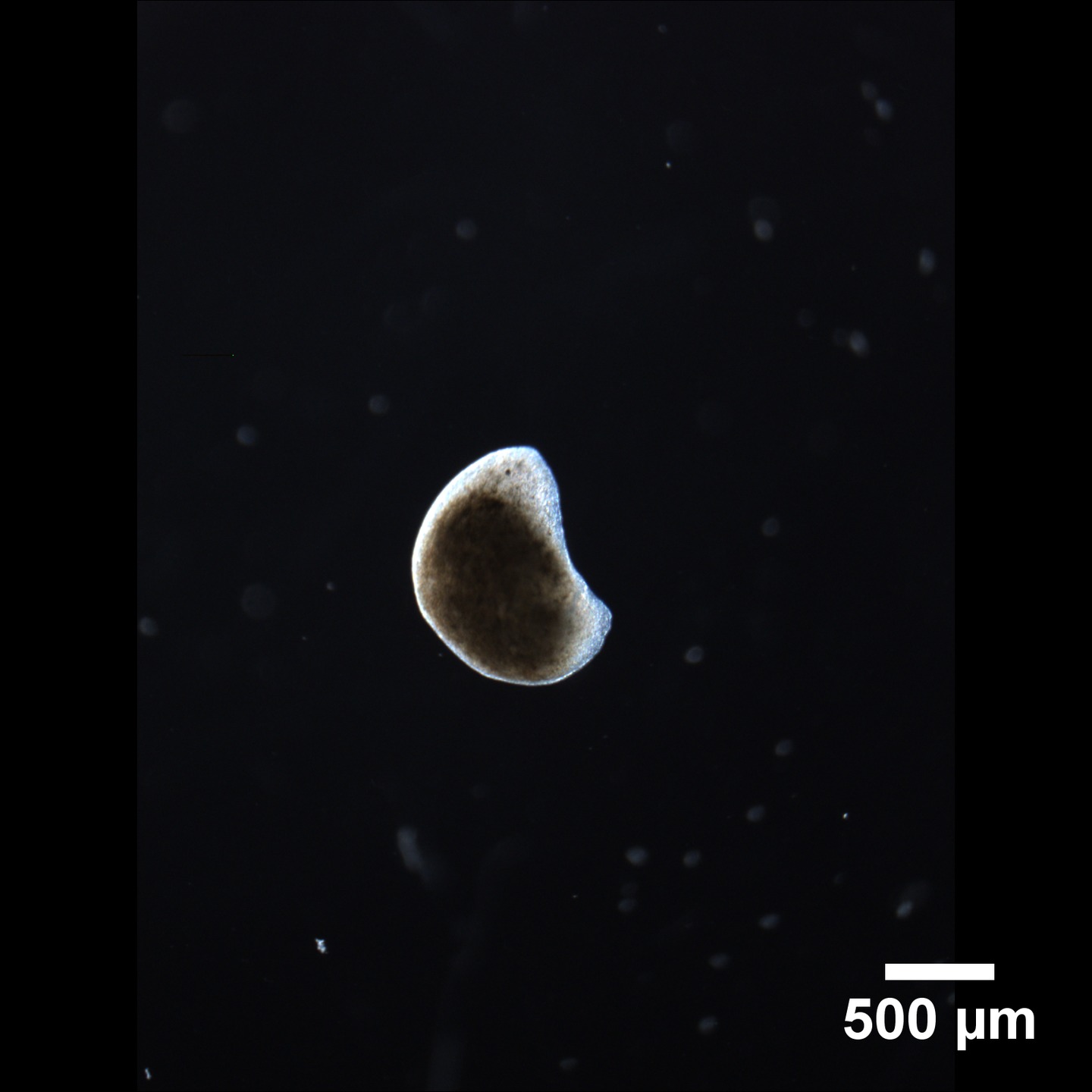

Supplement: S1 Dataset — This dataset contains brightfield image and corresponding synapsin stains for the VNC-free and VNC-containing small fragment cutting scenarios shown in Fig 6. Each image is labeled in the format “x_dpc_Sample_y_tn.jpg”, where “x” represents the number of days post cutting and “y” the replicate number. (ZIP) [file pcbi.1006904.s016.zip › smallfragments/VNC-containing/Brightfield_images/5 dpc_Sample 5_tn.jpg]

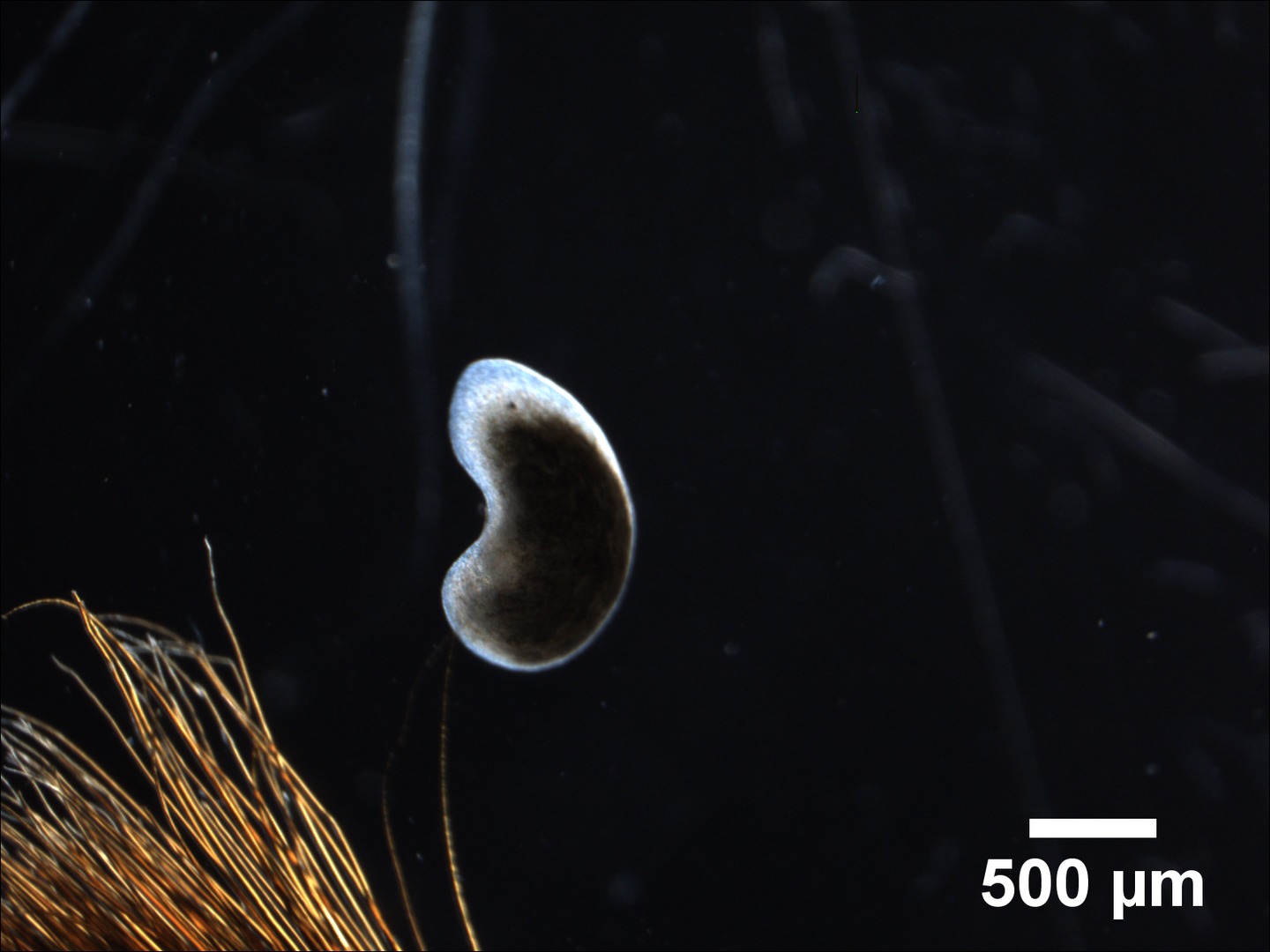

Supplement: S1 Dataset — This dataset contains brightfield image and corresponding synapsin stains for the VNC-free and VNC-containing small fragment cutting scenarios shown in Fig 6. Each image is labeled in the format “x_dpc_Sample_y_tn.jpg”, where “x” represents the number of days post cutting and “y” the replicate number. (ZIP) [file pcbi.1006904.s016.zip › smallfragments/VNC-containing/Brightfield_images/5 dpc_Sample 6_tn.jpg]

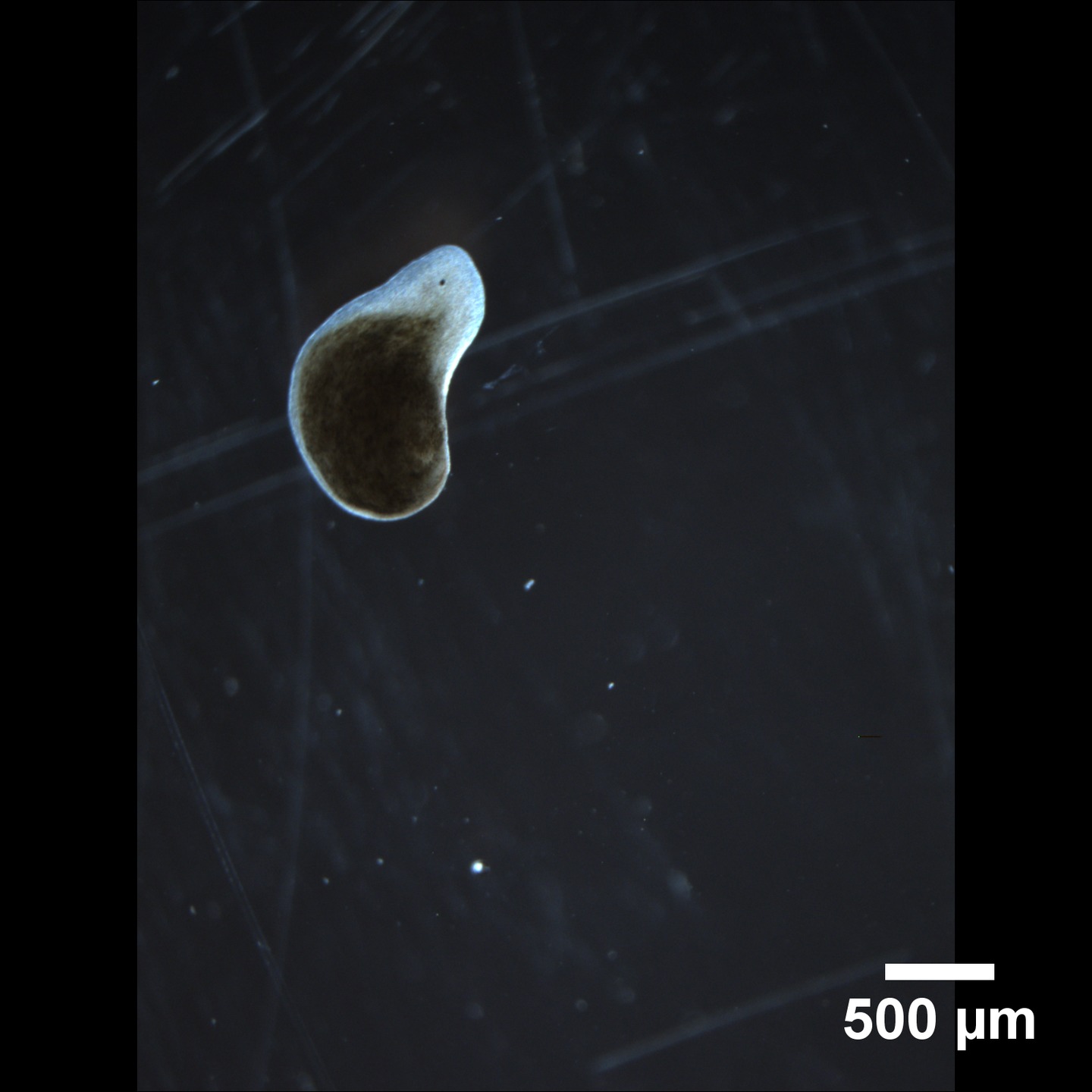

Supplement: S1 Dataset — This dataset contains brightfield image and corresponding synapsin stains for the VNC-free and VNC-containing small fragment cutting scenarios shown in Fig 6. Each image is labeled in the format “x_dpc_Sample_y_tn.jpg”, where “x” represents the number of days post cutting and “y” the replicate number. (ZIP) [file pcbi.1006904.s016.zip › smallfragments/VNC-containing/Brightfield_images/6 dpc_Sample 1_tn.jpg]

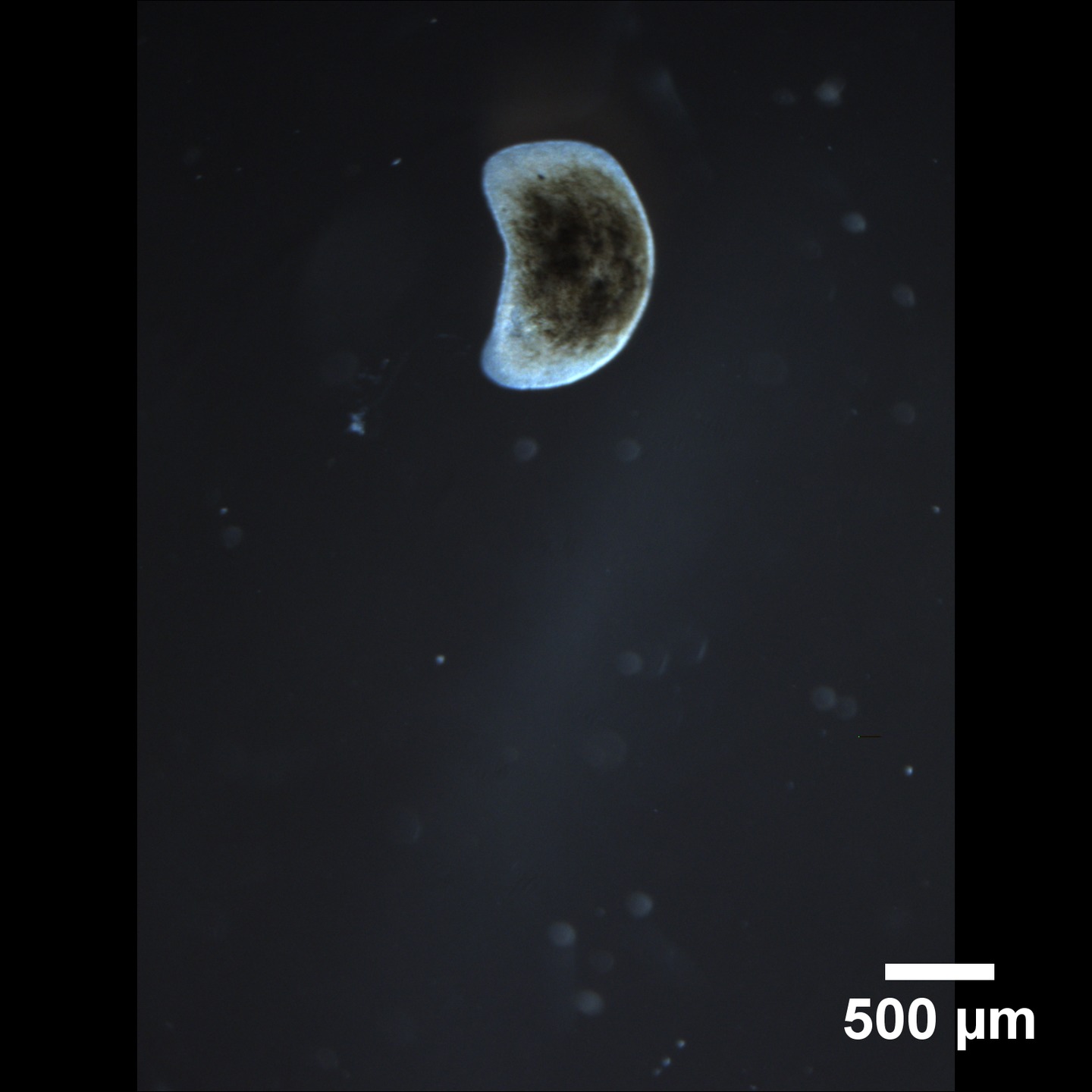

Supplement: S1 Dataset — This dataset contains brightfield image and corresponding synapsin stains for the VNC-free and VNC-containing small fragment cutting scenarios shown in Fig 6. Each image is labeled in the format “x_dpc_Sample_y_tn.jpg”, where “x” represents the number of days post cutting and “y” the replicate number. (ZIP) [file pcbi.1006904.s016.zip › smallfragments/VNC-containing/Brightfield_images/6 dpc_Sample 2_tn.jpg]

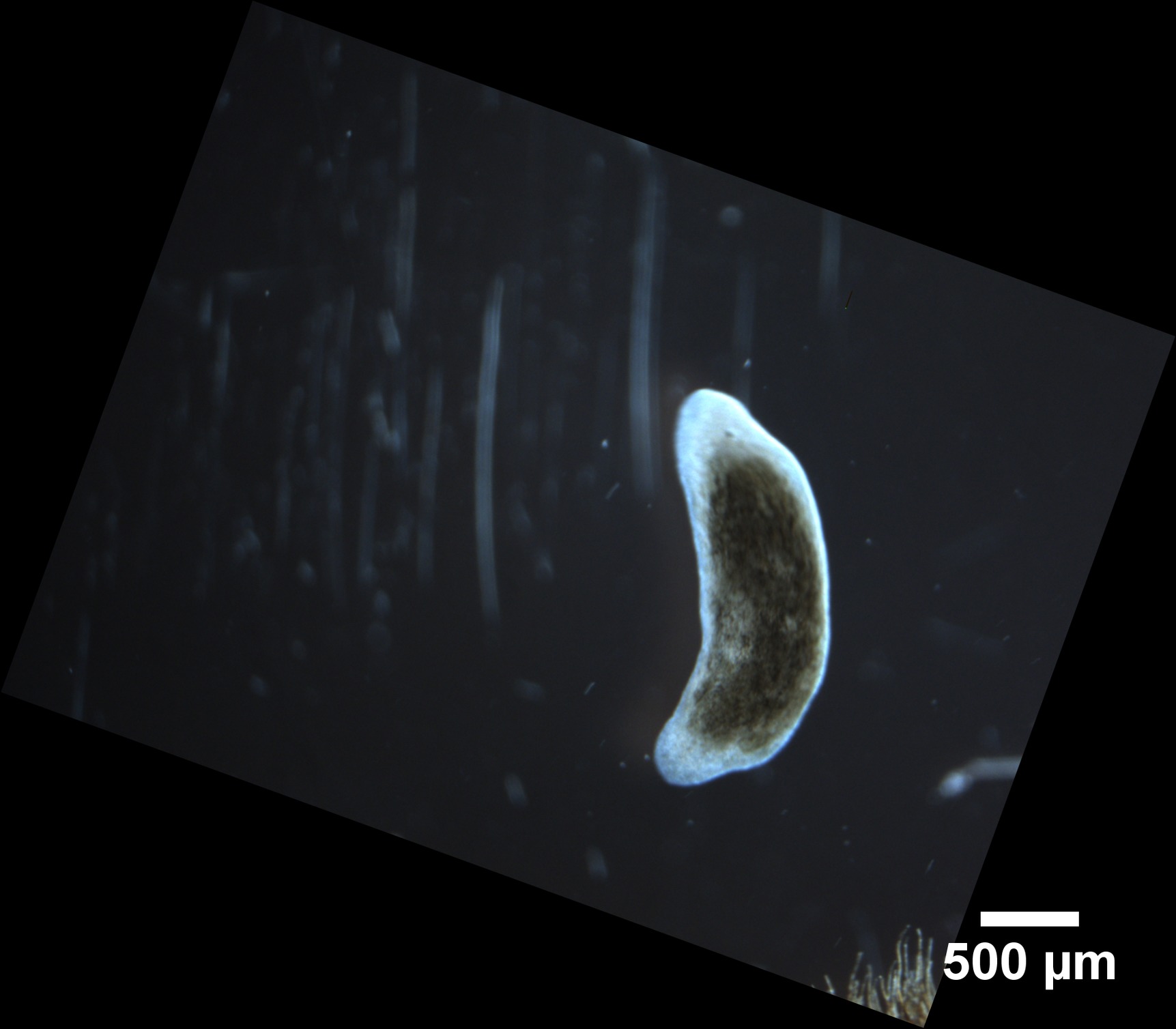

Supplement: S1 Dataset — This dataset contains brightfield image and corresponding synapsin stains for the VNC-free and VNC-containing small fragment cutting scenarios shown in Fig 6. Each image is labeled in the format “x_dpc_Sample_y_tn.jpg”, where “x” represents the number of days post cutting and “y” the replicate number. (ZIP) [file pcbi.1006904.s016.zip › smallfragments/VNC-containing/Brightfield_images/6 dpc_Sample 3_tn.jpg]

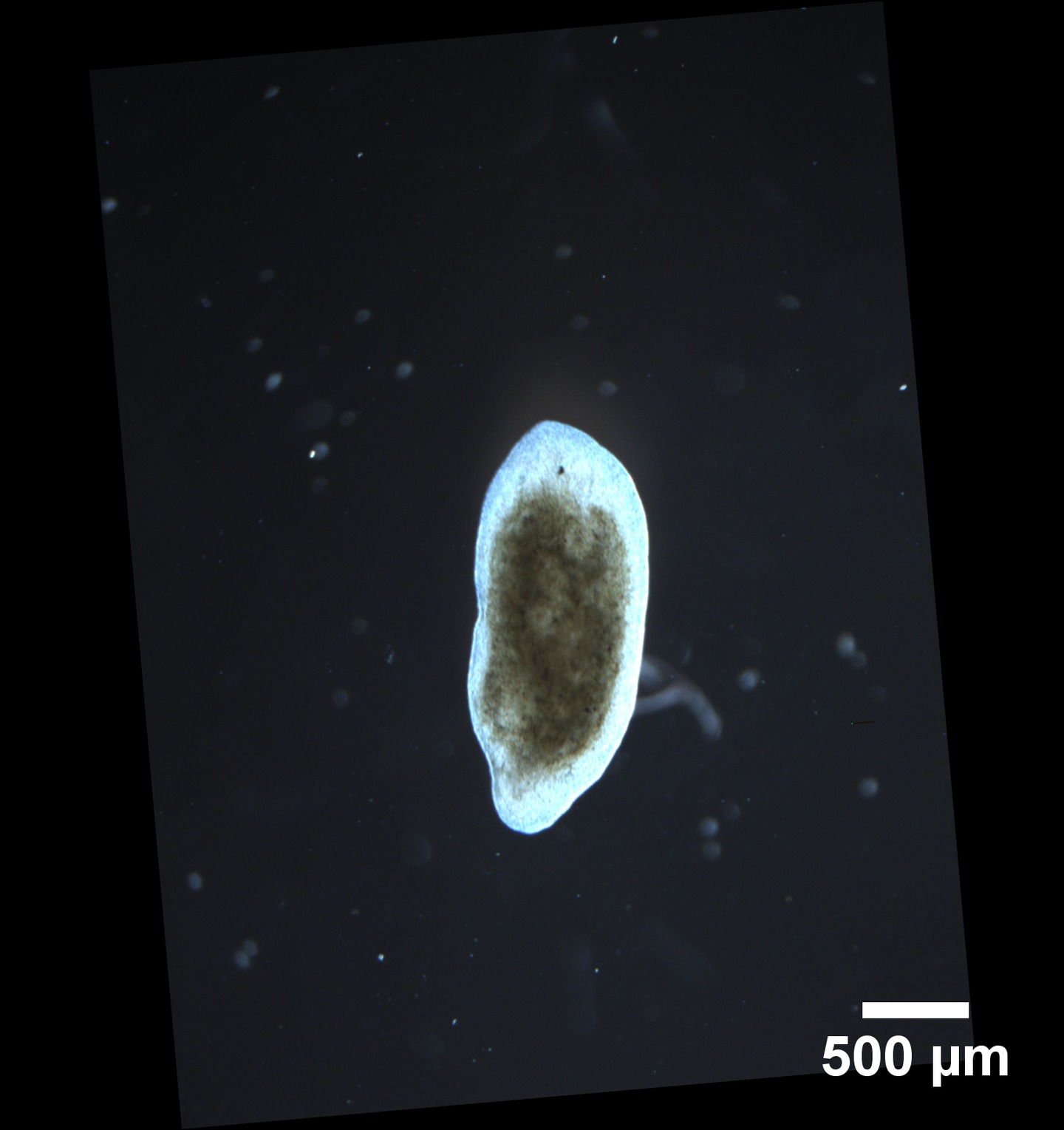

Supplement: S1 Dataset — This dataset contains brightfield image and corresponding synapsin stains for the VNC-free and VNC-containing small fragment cutting scenarios shown in Fig 6. Each image is labeled in the format “x_dpc_Sample_y_tn.jpg”, where “x” represents the number of days post cutting and “y” the replicate number. (ZIP) [file pcbi.1006904.s016.zip › smallfragments/VNC-containing/Brightfield_images/6 dpc_Sample 4_tn.jpg]

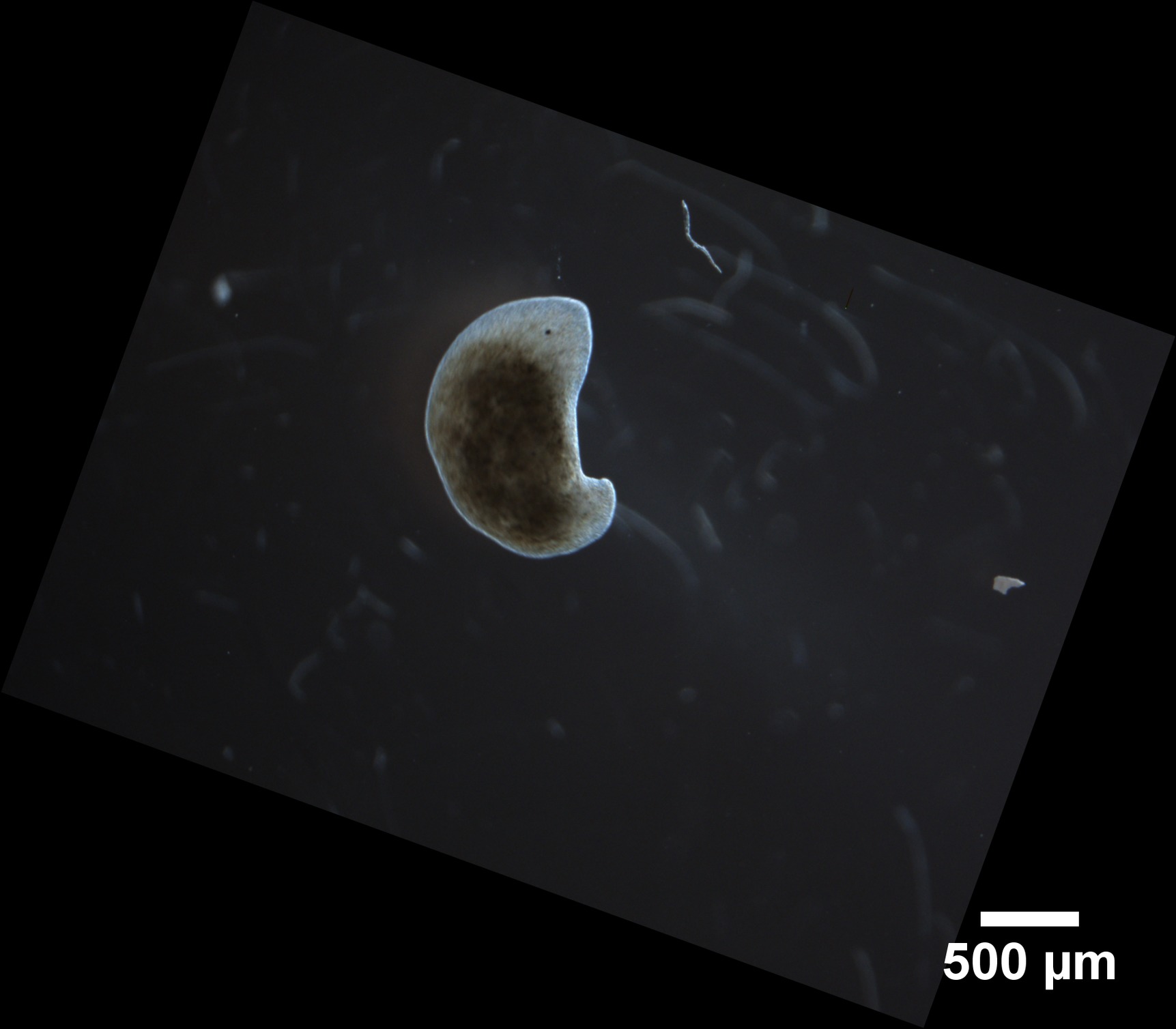

Supplement: S1 Dataset — This dataset contains brightfield image and corresponding synapsin stains for the VNC-free and VNC-containing small fragment cutting scenarios shown in Fig 6. Each image is labeled in the format “x_dpc_Sample_y_tn.jpg”, where “x” represents the number of days post cutting and “y” the replicate number. (ZIP) [file pcbi.1006904.s016.zip › smallfragments/VNC-containing/Brightfield_images/6 dpc_Sample 5_tn.jpg]

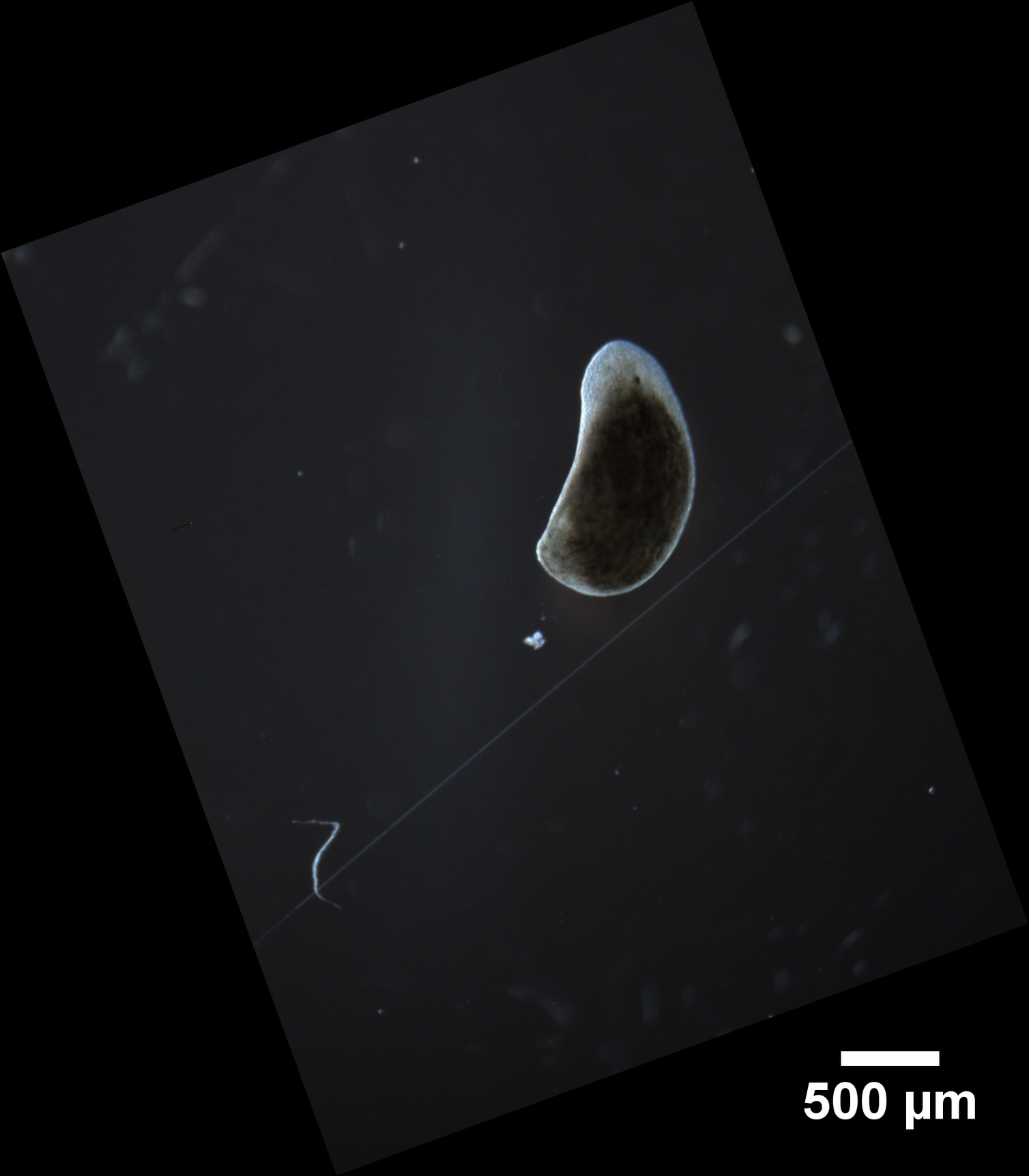

Supplement: S1 Dataset — This dataset contains brightfield image and corresponding synapsin stains for the VNC-free and VNC-containing small fragment cutting scenarios shown in Fig 6. Each image is labeled in the format “x_dpc_Sample_y_tn.jpg”, where “x” represents the number of days post cutting and “y” the replicate number. (ZIP) [file pcbi.1006904.s016.zip › smallfragments/VNC-containing/Brightfield_images/6 dpc_Sample 6_tn.jpg]

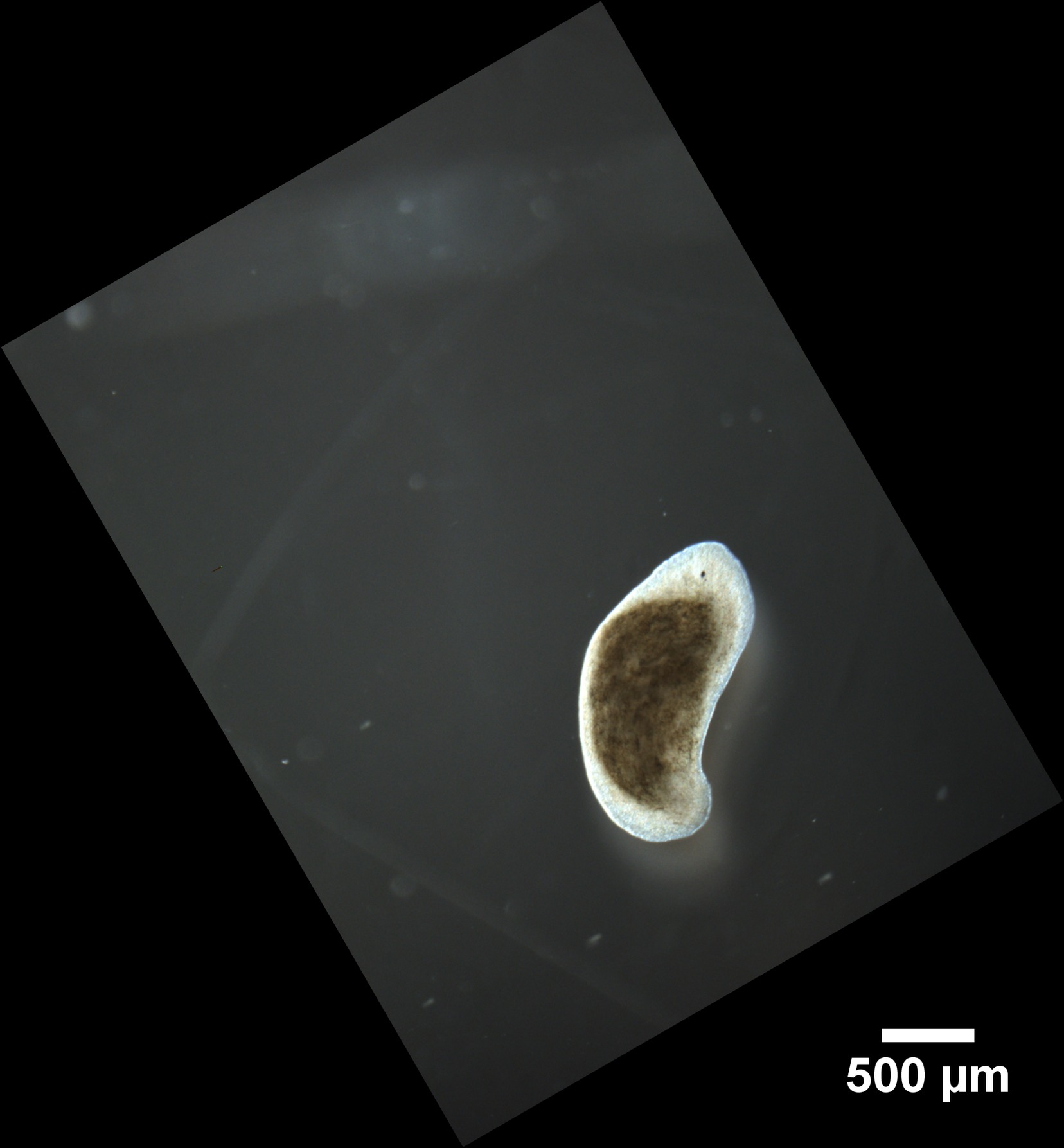

Supplement: S1 Dataset — This dataset contains brightfield image and corresponding synapsin stains for the VNC-free and VNC-containing small fragment cutting scenarios shown in Fig 6. Each image is labeled in the format “x_dpc_Sample_y_tn.jpg”, where “x” represents the number of days post cutting and “y” the replicate number. (ZIP) [file pcbi.1006904.s016.zip › smallfragments/VNC-containing/Brightfield_images/7 dpc_Sample 1_tn.jpg]

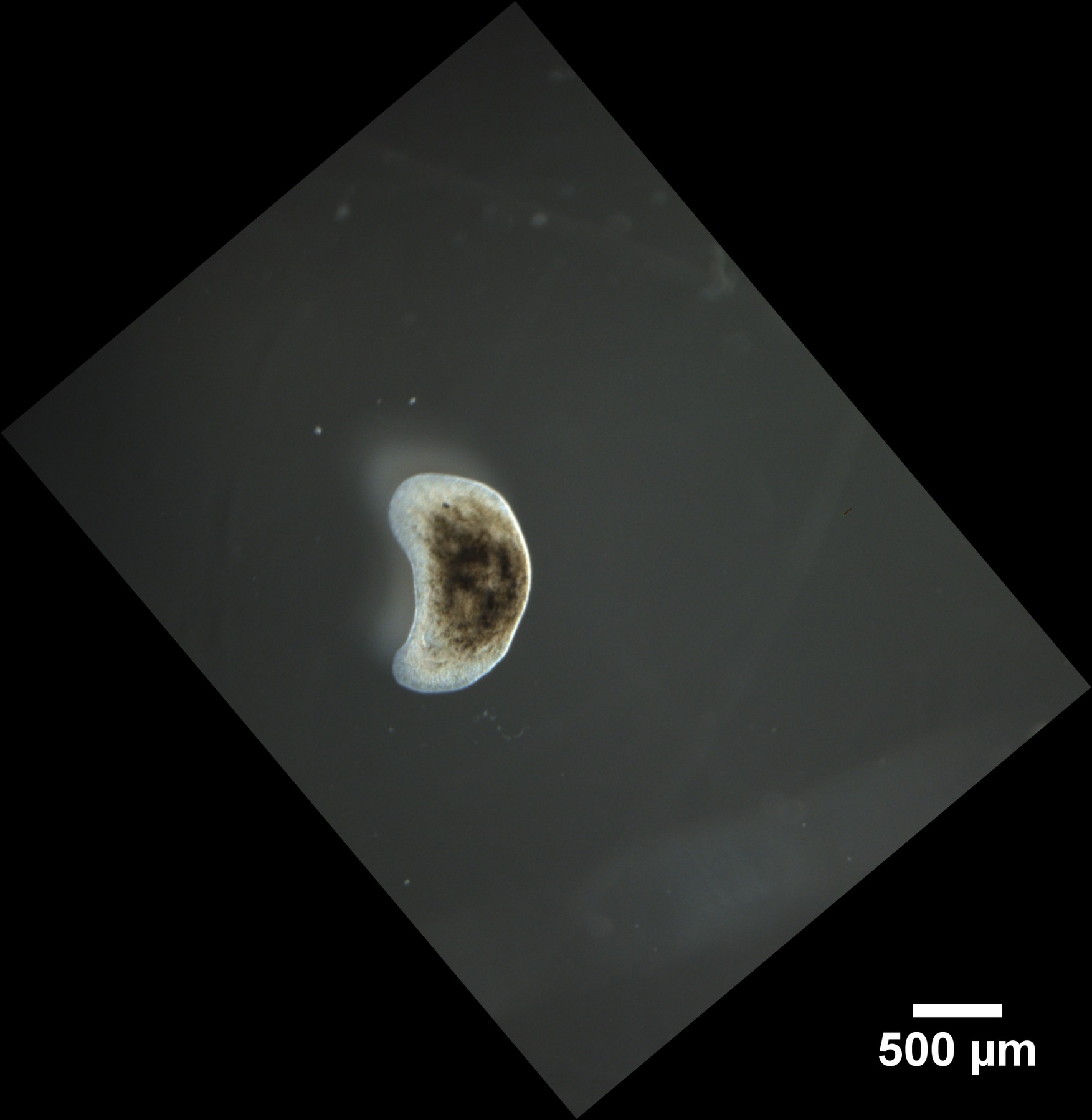

Supplement: S1 Dataset — This dataset contains brightfield image and corresponding synapsin stains for the VNC-free and VNC-containing small fragment cutting scenarios shown in Fig 6. Each image is labeled in the format “x_dpc_Sample_y_tn.jpg”, where “x” represents the number of days post cutting and “y” the replicate number. (ZIP) [file pcbi.1006904.s016.zip › smallfragments/VNC-containing/Brightfield_images/7 dpc_Sample 2_tn.jpg]

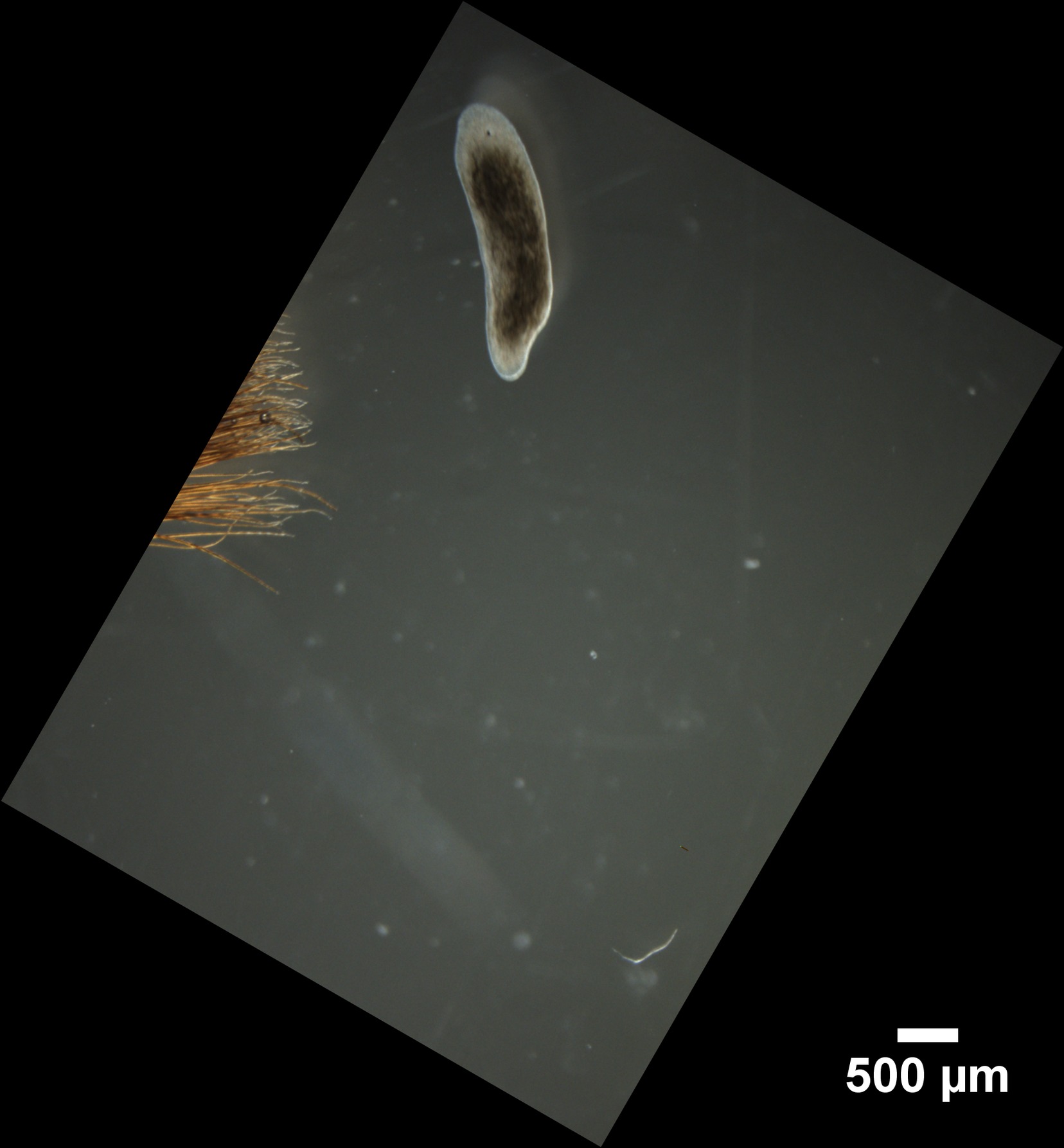

Supplement: S1 Dataset — This dataset contains brightfield image and corresponding synapsin stains for the VNC-free and VNC-containing small fragment cutting scenarios shown in Fig 6. Each image is labeled in the format “x_dpc_Sample_y_tn.jpg”, where “x” represents the number of days post cutting and “y” the replicate number. (ZIP) [file pcbi.1006904.s016.zip › smallfragments/VNC-containing/Brightfield_images/7 dpc_Sample 3_tn.jpg]

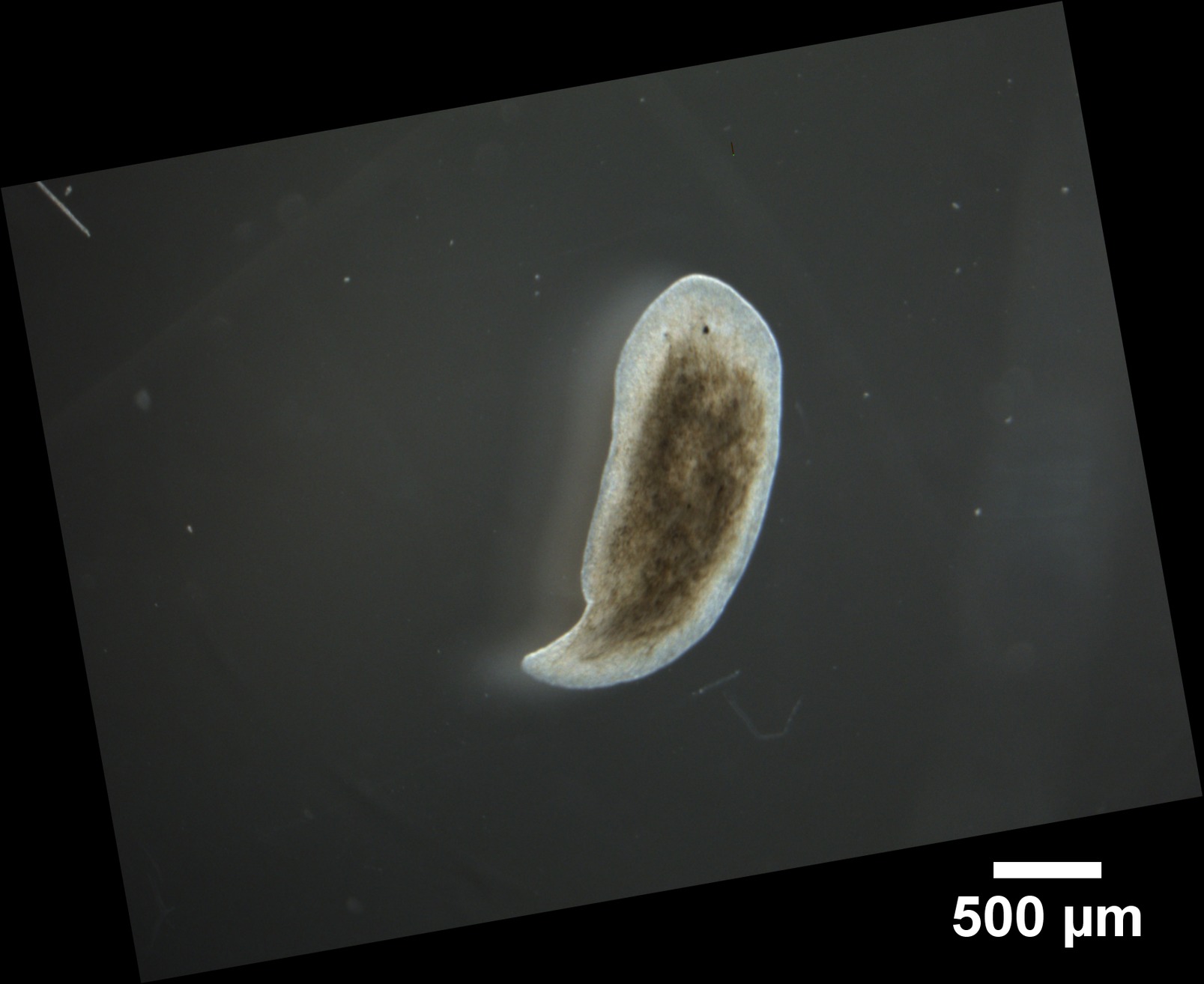

Supplement: S1 Dataset — This dataset contains brightfield image and corresponding synapsin stains for the VNC-free and VNC-containing small fragment cutting scenarios shown in Fig 6. Each image is labeled in the format “x_dpc_Sample_y_tn.jpg”, where “x” represents the number of days post cutting and “y” the replicate number. (ZIP) [file pcbi.1006904.s016.zip › smallfragments/VNC-containing/Brightfield_images/7 dpc_Sample 4_tn.jpg]

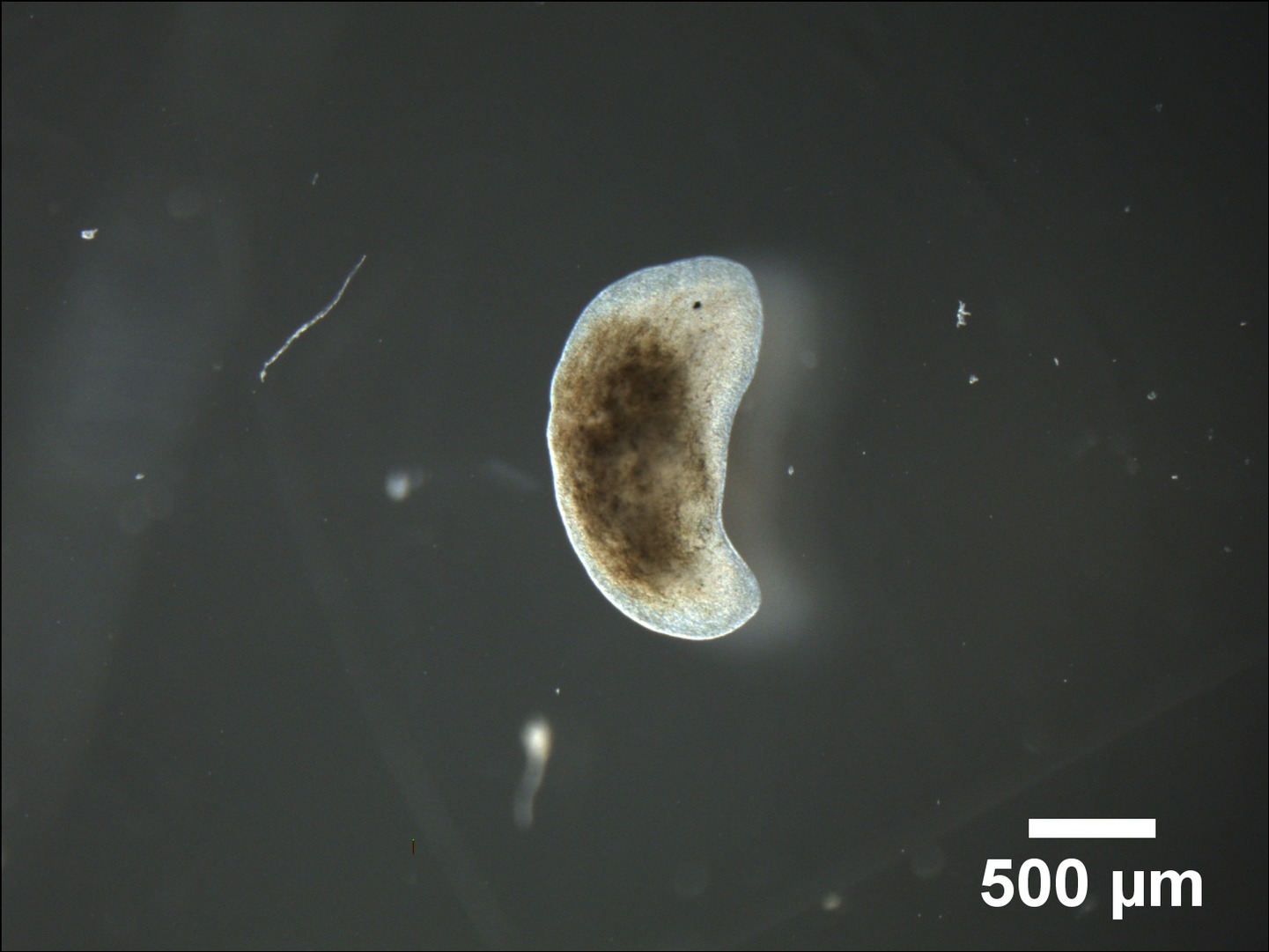

Supplement: S1 Dataset — This dataset contains brightfield image and corresponding synapsin stains for the VNC-free and VNC-containing small fragment cutting scenarios shown in Fig 6. Each image is labeled in the format “x_dpc_Sample_y_tn.jpg”, where “x” represents the number of days post cutting and “y” the replicate number. (ZIP) [file pcbi.1006904.s016.zip › smallfragments/VNC-containing/Brightfield_images/7 dpc_Sample 5_tn.jpg]

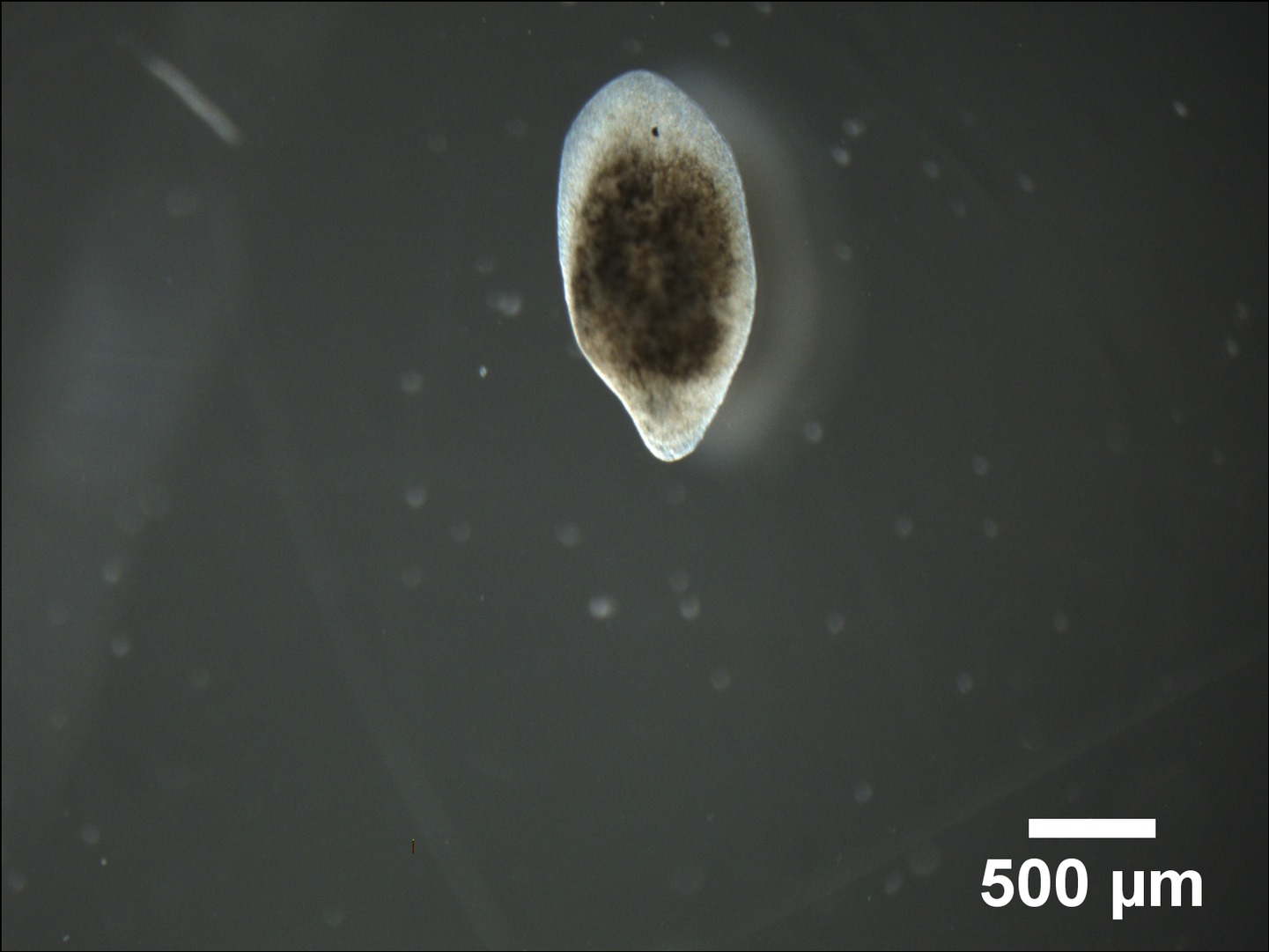

Supplement: S1 Dataset — This dataset contains brightfield image and corresponding synapsin stains for the VNC-free and VNC-containing small fragment cutting scenarios shown in Fig 6. Each image is labeled in the format “x_dpc_Sample_y_tn.jpg”, where “x” represents the number of days post cutting and “y” the replicate number. (ZIP) [file pcbi.1006904.s016.zip › smallfragments/VNC-containing/Brightfield_images/7 dpc_Sample 6_tn.jpg]

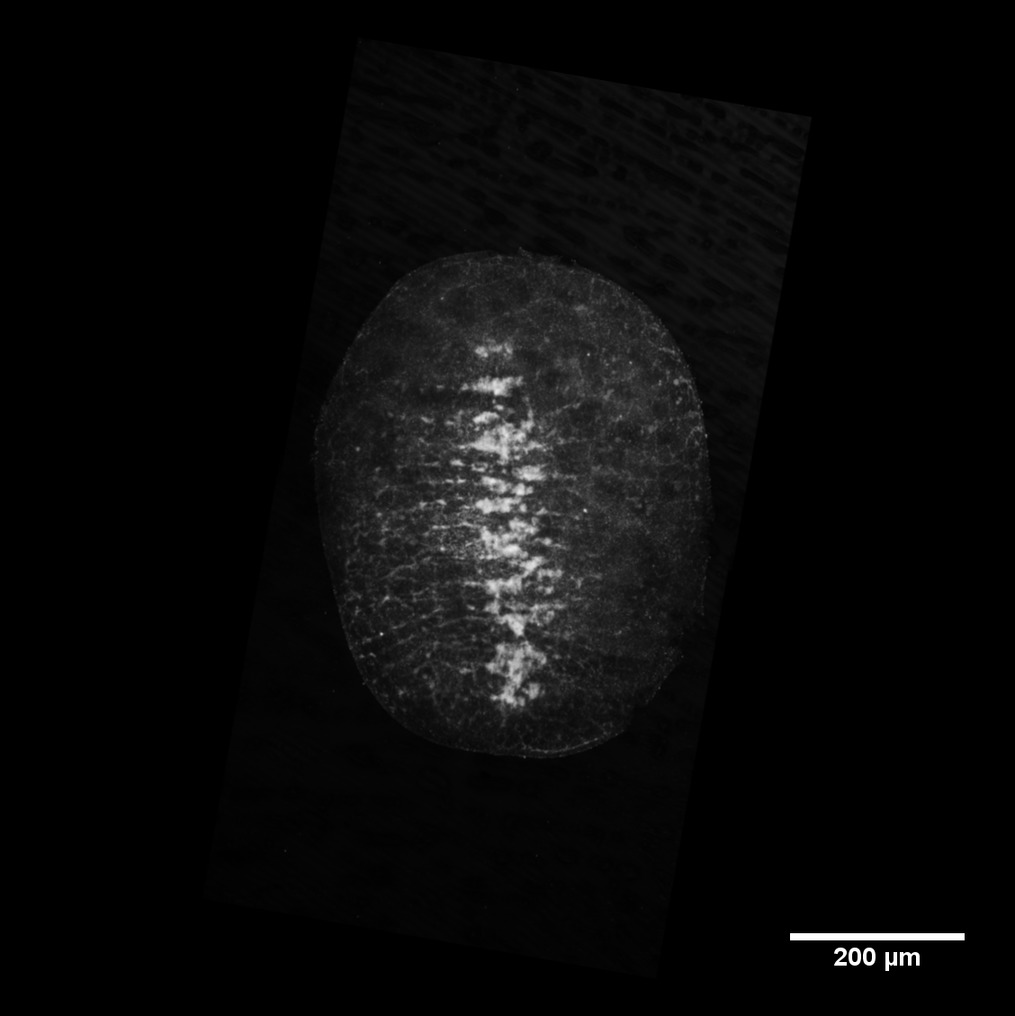

Supplement: S1 Dataset — This dataset contains brightfield image and corresponding synapsin stains for the VNC-free and VNC-containing small fragment cutting scenarios shown in Fig 6. Each image is labeled in the format “x_dpc_Sample_y_tn.jpg”, where “x” represents the number of days post cutting and “y” the replicate number. (ZIP) [file pcbi.1006904.s016.zip › smallfragments/VNC-containing/synapsin_stains/1 dpc_Sample 1_tn.jpg]

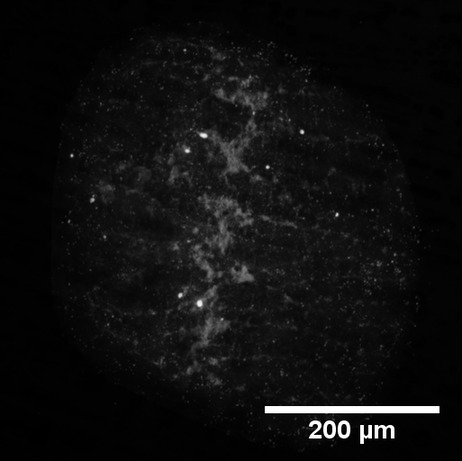

Supplement: S1 Dataset — This dataset contains brightfield image and corresponding synapsin stains for the VNC-free and VNC-containing small fragment cutting scenarios shown in Fig 6. Each image is labeled in the format “x_dpc_Sample_y_tn.jpg”, where “x” represents the number of days post cutting and “y” the replicate number. (ZIP) [file pcbi.1006904.s016.zip › smallfragments/VNC-containing/synapsin_stains/1 dpc_Sample 2_tn.jpg]

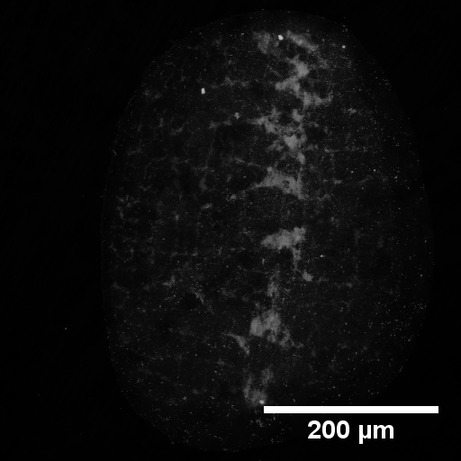

Supplement: S1 Dataset — This dataset contains brightfield image and corresponding synapsin stains for the VNC-free and VNC-containing small fragment cutting scenarios shown in Fig 6. Each image is labeled in the format “x_dpc_Sample_y_tn.jpg”, where “x” represents the number of days post cutting and “y” the replicate number. (ZIP) [file pcbi.1006904.s016.zip › smallfragments/VNC-containing/synapsin_stains/1 dpc_Sample 3_tn.jpg]

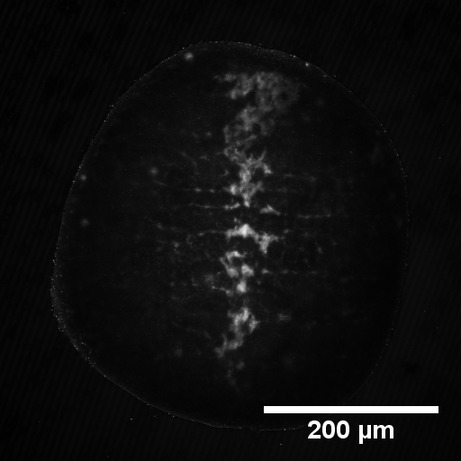

Supplement: S1 Dataset — This dataset contains brightfield image and corresponding synapsin stains for the VNC-free and VNC-containing small fragment cutting scenarios shown in Fig 6. Each image is labeled in the format “x_dpc_Sample_y_tn.jpg”, where “x” represents the number of days post cutting and “y” the replicate number. (ZIP) [file pcbi.1006904.s016.zip › smallfragments/VNC-containing/synapsin_stains/1 dpc_Sample 4_tn.jpg]

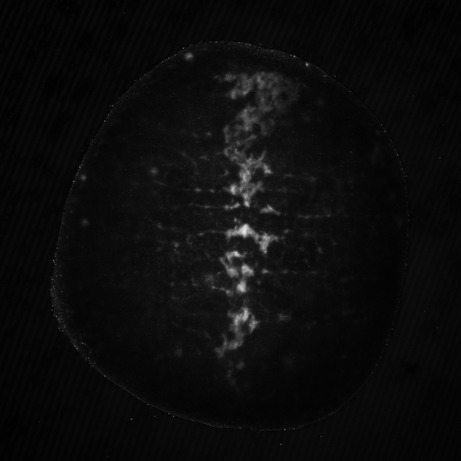

Supplement: S1 Dataset — This dataset contains brightfield image and corresponding synapsin stains for the VNC-free and VNC-containing small fragment cutting scenarios shown in Fig 6. Each image is labeled in the format “x_dpc_Sample_y_tn.jpg”, where “x” represents the number of days post cutting and “y” the replicate number. (ZIP) [file pcbi.1006904.s016.zip › smallfragments/VNC-containing/synapsin_stains/1 dpc_Sample 5_tn.jpg]

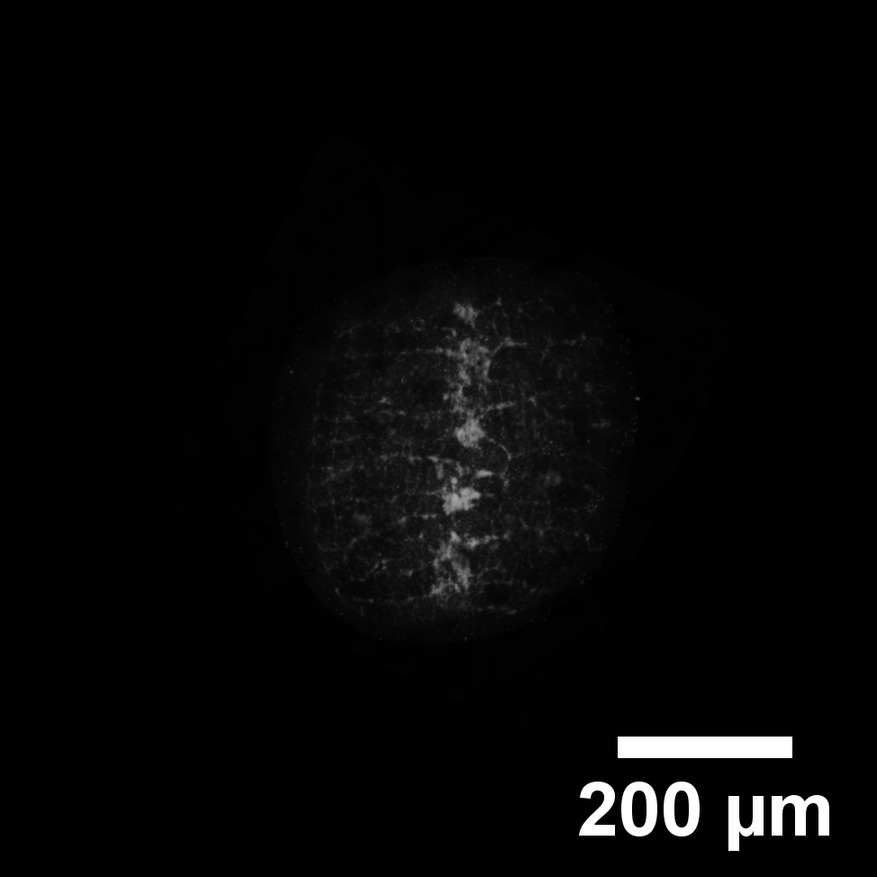

Supplement: S1 Dataset — This dataset contains brightfield image and corresponding synapsin stains for the VNC-free and VNC-containing small fragment cutting scenarios shown in Fig 6. Each image is labeled in the format “x_dpc_Sample_y_tn.jpg”, where “x” represents the number of days post cutting and “y” the replicate number. (ZIP) [file pcbi.1006904.s016.zip › smallfragments/VNC-containing/synapsin_stains/1 dpc_Sample 6_tn.jpg]

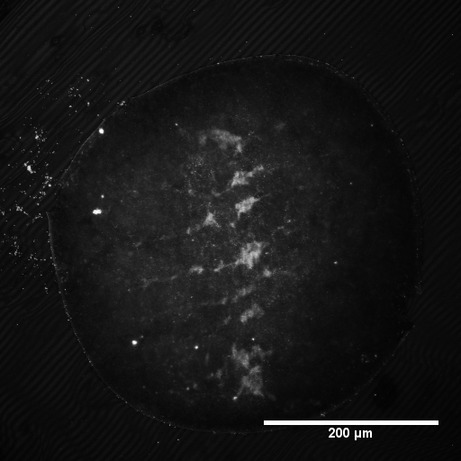

Supplement: S1 Dataset — This dataset contains brightfield image and corresponding synapsin stains for the VNC-free and VNC-containing small fragment cutting scenarios shown in Fig 6. Each image is labeled in the format “x_dpc_Sample_y_tn.jpg”, where “x” represents the number of days post cutting and “y” the replicate number. (ZIP) [file pcbi.1006904.s016.zip › smallfragments/VNC-containing/synapsin_stains/1 dpc_Sample 7_tn.jpg]

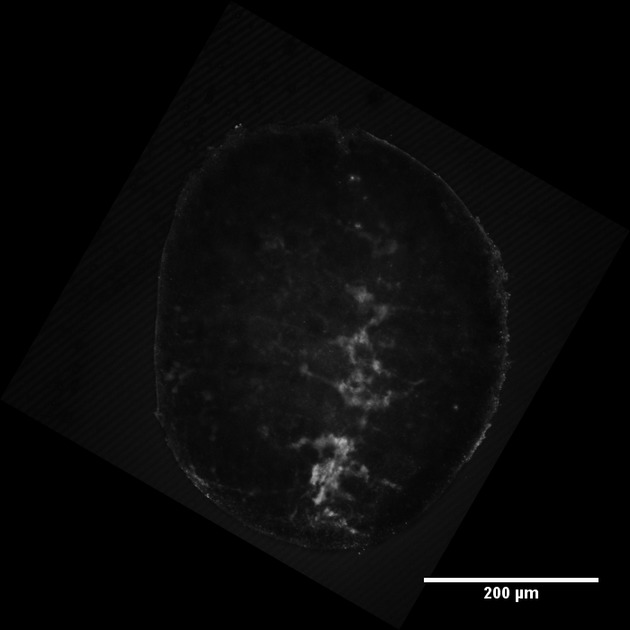

Supplement: S1 Dataset — This dataset contains brightfield image and corresponding synapsin stains for the VNC-free and VNC-containing small fragment cutting scenarios shown in Fig 6. Each image is labeled in the format “x_dpc_Sample_y_tn.jpg”, where “x” represents the number of days post cutting and “y” the replicate number. (ZIP) [file pcbi.1006904.s016.zip › smallfragments/VNC-containing/synapsin_stains/1 dpc_Sample 8_tn.jpg]

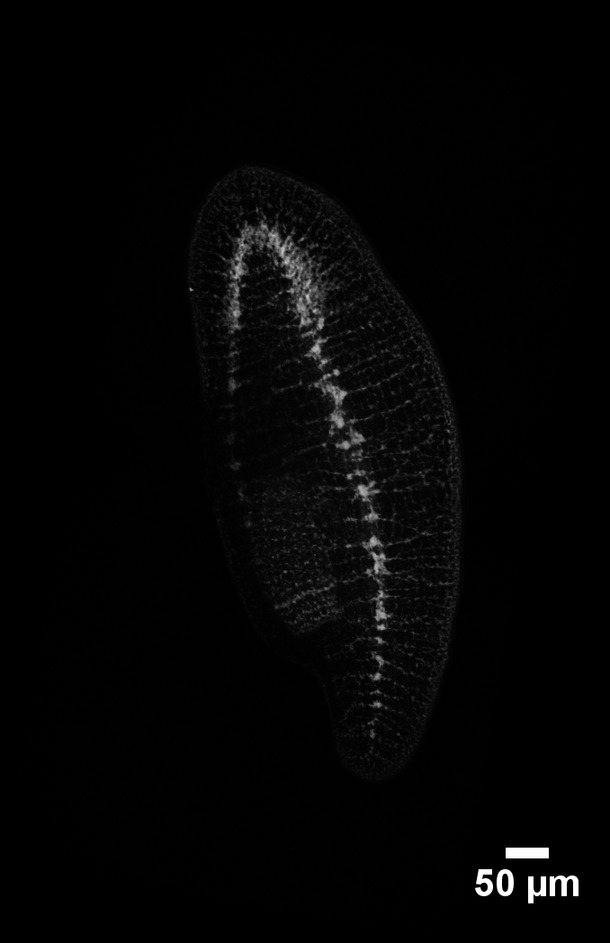

Supplement: S1 Dataset — This dataset contains brightfield image and corresponding synapsin stains for the VNC-free and VNC-containing small fragment cutting scenarios shown in Fig 6. Each image is labeled in the format “x_dpc_Sample_y_tn.jpg”, where “x” represents the number of days post cutting and “y” the replicate number. (ZIP) [file pcbi.1006904.s016.zip › smallfragments/VNC-containing/synapsin_stains/10 dpc_Sample 10_tn.jpg]

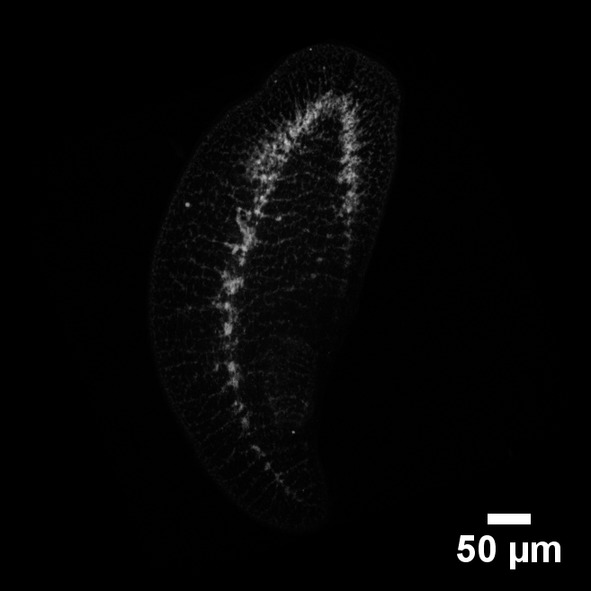

Supplement: S1 Dataset — This dataset contains brightfield image and corresponding synapsin stains for the VNC-free and VNC-containing small fragment cutting scenarios shown in Fig 6. Each image is labeled in the format “x_dpc_Sample_y_tn.jpg”, where “x” represents the number of days post cutting and “y” the replicate number. (ZIP) [file pcbi.1006904.s016.zip › smallfragments/VNC-containing/synapsin_stains/10 dpc_Sample 1_tn.jpg]

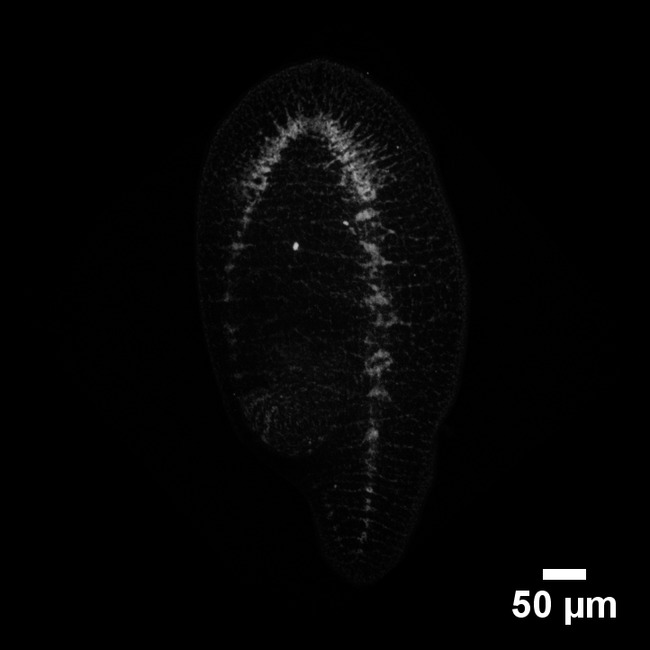

Supplement: S1 Dataset — This dataset contains brightfield image and corresponding synapsin stains for the VNC-free and VNC-containing small fragment cutting scenarios shown in Fig 6. Each image is labeled in the format “x_dpc_Sample_y_tn.jpg”, where “x” represents the number of days post cutting and “y” the replicate number. (ZIP) [file pcbi.1006904.s016.zip › smallfragments/VNC-containing/synapsin_stains/10 dpc_Sample 2_tn.jpg]

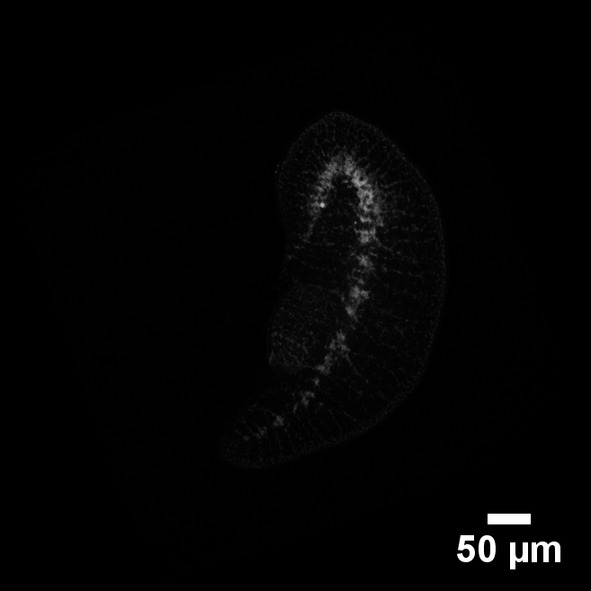

Supplement: S1 Dataset — This dataset contains brightfield image and corresponding synapsin stains for the VNC-free and VNC-containing small fragment cutting scenarios shown in Fig 6. Each image is labeled in the format “x_dpc_Sample_y_tn.jpg”, where “x” represents the number of days post cutting and “y” the replicate number. (ZIP) [file pcbi.1006904.s016.zip › smallfragments/VNC-containing/synapsin_stains/10 dpc_Sample 3_tn.jpg]

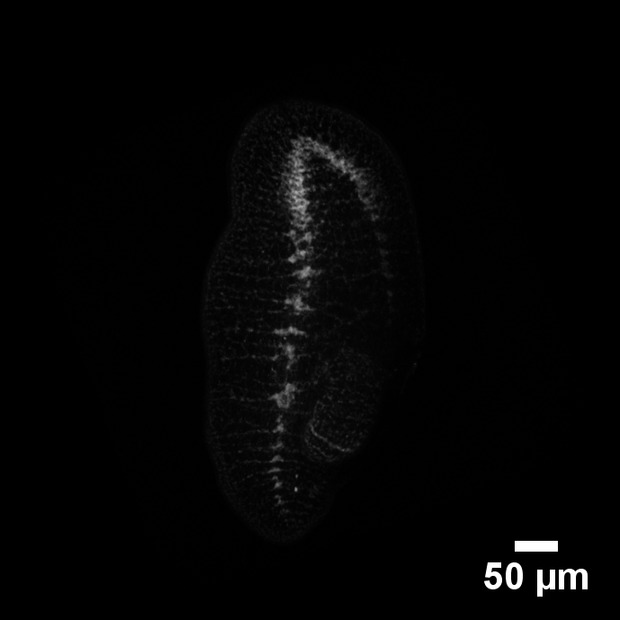

Supplement: S1 Dataset — This dataset contains brightfield image and corresponding synapsin stains for the VNC-free and VNC-containing small fragment cutting scenarios shown in Fig 6. Each image is labeled in the format “x_dpc_Sample_y_tn.jpg”, where “x” represents the number of days post cutting and “y” the replicate number. (ZIP) [file pcbi.1006904.s016.zip › smallfragments/VNC-containing/synapsin_stains/10 dpc_Sample 4_tn.jpg]

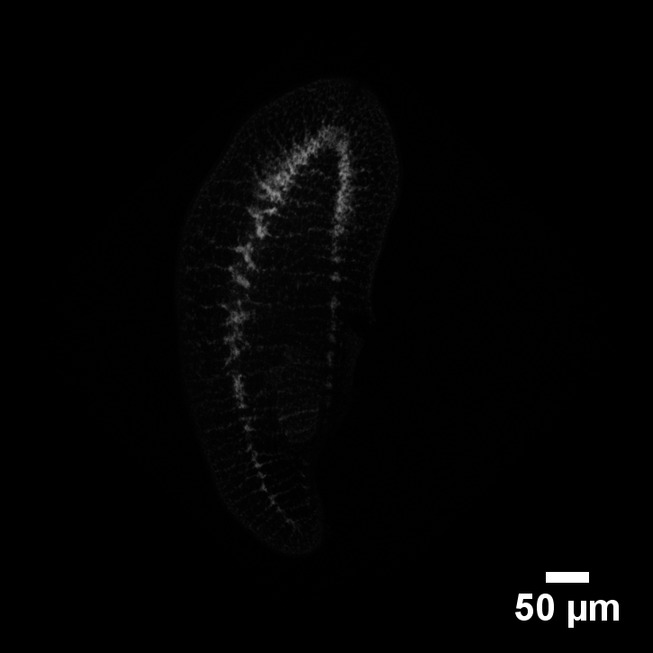

Supplement: S1 Dataset — This dataset contains brightfield image and corresponding synapsin stains for the VNC-free and VNC-containing small fragment cutting scenarios shown in Fig 6. Each image is labeled in the format “x_dpc_Sample_y_tn.jpg”, where “x” represents the number of days post cutting and “y” the replicate number. (ZIP) [file pcbi.1006904.s016.zip › smallfragments/VNC-containing/synapsin_stains/10 dpc_Sample 5_tn.jpg]

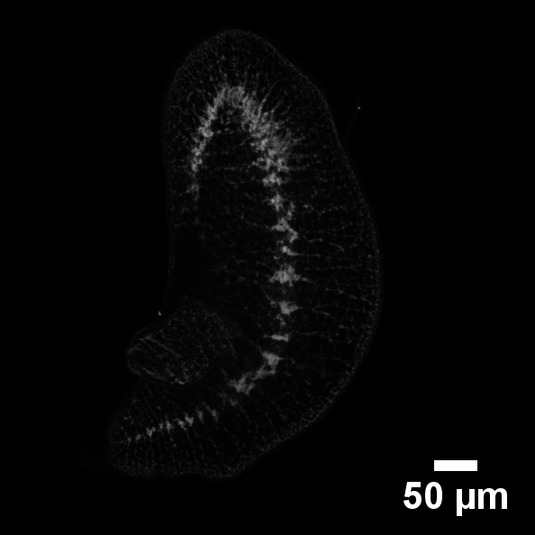

Supplement: S1 Dataset — This dataset contains brightfield image and corresponding synapsin stains for the VNC-free and VNC-containing small fragment cutting scenarios shown in Fig 6. Each image is labeled in the format “x_dpc_Sample_y_tn.jpg”, where “x” represents the number of days post cutting and “y” the replicate number. (ZIP) [file pcbi.1006904.s016.zip › smallfragments/VNC-containing/synapsin_stains/10 dpc_Sample 6_tn.jpg]

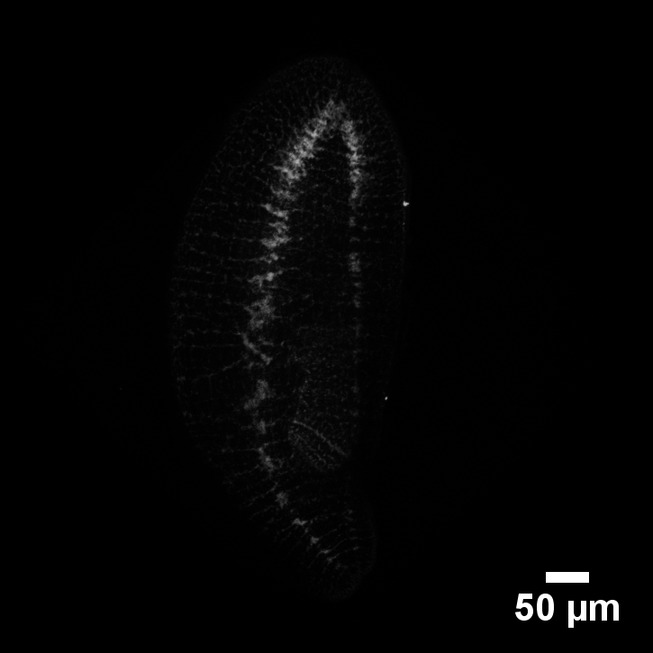

Supplement: S1 Dataset — This dataset contains brightfield image and corresponding synapsin stains for the VNC-free and VNC-containing small fragment cutting scenarios shown in Fig 6. Each image is labeled in the format “x_dpc_Sample_y_tn.jpg”, where “x” represents the number of days post cutting and “y” the replicate number. (ZIP) [file pcbi.1006904.s016.zip › smallfragments/VNC-containing/synapsin_stains/10 dpc_Sample 7_tn.jpg]

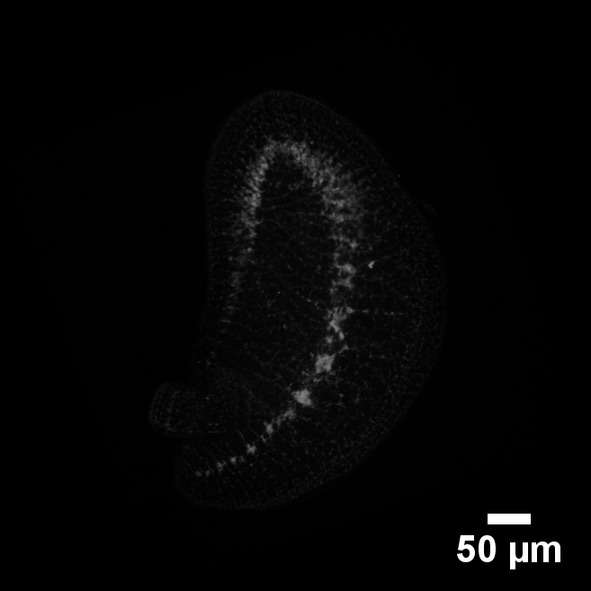

Supplement: S1 Dataset — This dataset contains brightfield image and corresponding synapsin stains for the VNC-free and VNC-containing small fragment cutting scenarios shown in Fig 6. Each image is labeled in the format “x_dpc_Sample_y_tn.jpg”, where “x” represents the number of days post cutting and “y” the replicate number. (ZIP) [file pcbi.1006904.s016.zip › smallfragments/VNC-containing/synapsin_stains/10 dpc_Sample 8_tn.jpg]

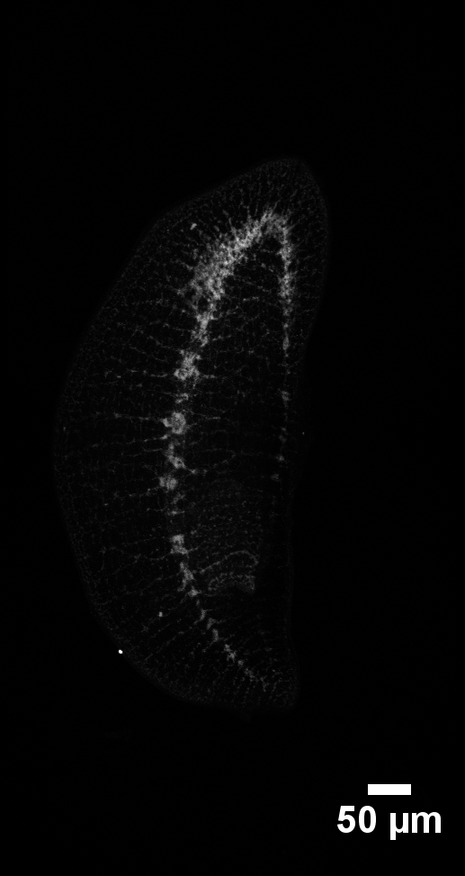

Supplement: S1 Dataset — This dataset contains brightfield image and corresponding synapsin stains for the VNC-free and VNC-containing small fragment cutting scenarios shown in Fig 6. Each image is labeled in the format “x_dpc_Sample_y_tn.jpg”, where “x” represents the number of days post cutting and “y” the replicate number. (ZIP) [file pcbi.1006904.s016.zip › smallfragments/VNC-containing/synapsin_stains/10 dpc_Sample 9_tn.jpg]

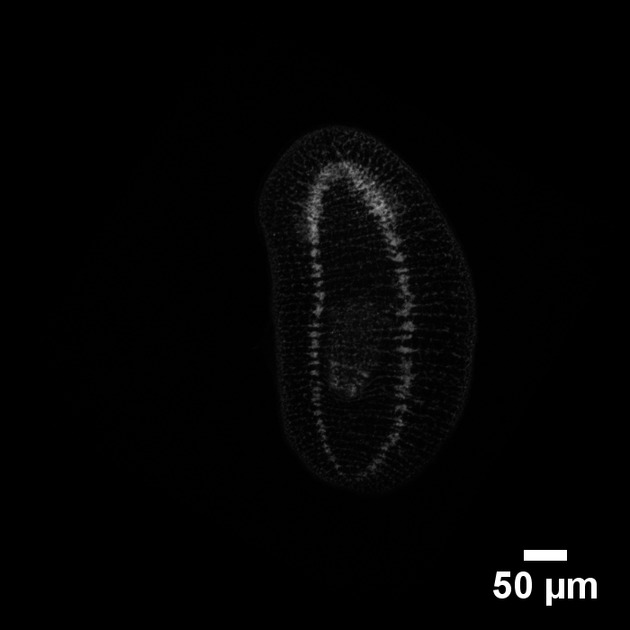

Supplement: S1 Dataset — This dataset contains brightfield image and corresponding synapsin stains for the VNC-free and VNC-containing small fragment cutting scenarios shown in Fig 6. Each image is labeled in the format “x_dpc_Sample_y_tn.jpg”, where “x” represents the number of days post cutting and “y” the replicate number. (ZIP) [file pcbi.1006904.s016.zip › smallfragments/VNC-containing/synapsin_stains/14 dpc_Sample 10_tn.jpg]

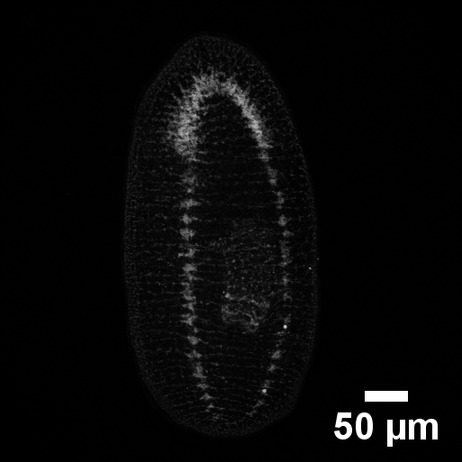

Supplement: S1 Dataset — This dataset contains brightfield image and corresponding synapsin stains for the VNC-free and VNC-containing small fragment cutting scenarios shown in Fig 6. Each image is labeled in the format “x_dpc_Sample_y_tn.jpg”, where “x” represents the number of days post cutting and “y” the replicate number. (ZIP) [file pcbi.1006904.s016.zip › smallfragments/VNC-containing/synapsin_stains/14 dpc_Sample 11_tn.jpg]

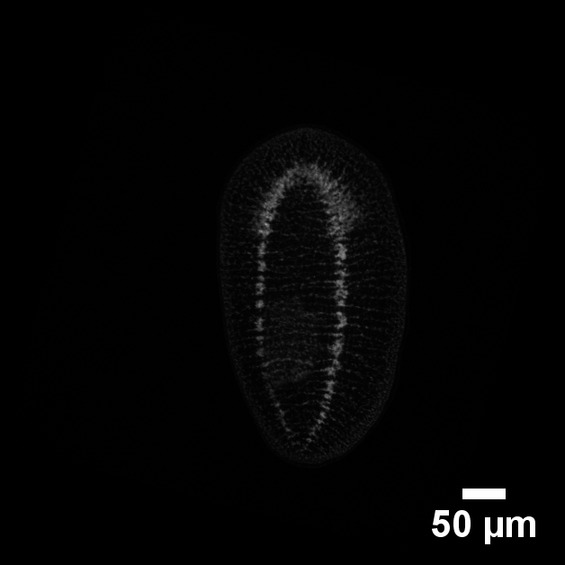

Supplement: S1 Dataset — This dataset contains brightfield image and corresponding synapsin stains for the VNC-free and VNC-containing small fragment cutting scenarios shown in Fig 6. Each image is labeled in the format “x_dpc_Sample_y_tn.jpg”, where “x” represents the number of days post cutting and “y” the replicate number. (ZIP) [file pcbi.1006904.s016.zip › smallfragments/VNC-containing/synapsin_stains/14 dpc_Sample 12_tn.jpg]

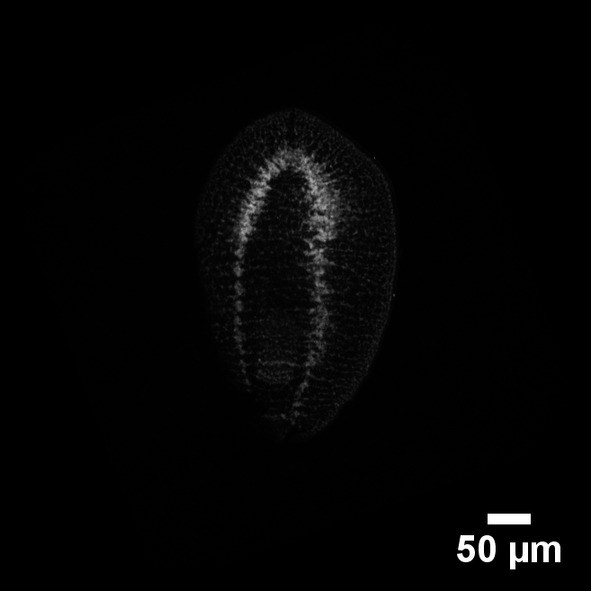

Supplement: S1 Dataset — This dataset contains brightfield image and corresponding synapsin stains for the VNC-free and VNC-containing small fragment cutting scenarios shown in Fig 6. Each image is labeled in the format “x_dpc_Sample_y_tn.jpg”, where “x” represents the number of days post cutting and “y” the replicate number. (ZIP) [file pcbi.1006904.s016.zip › smallfragments/VNC-containing/synapsin_stains/14 dpc_Sample 1_tn.jpg]

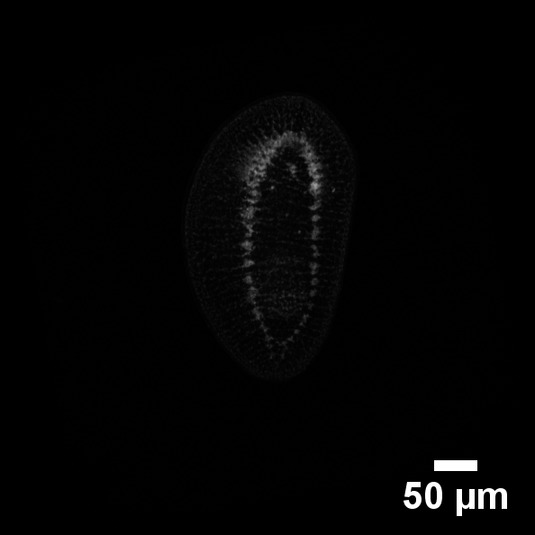

Supplement: S1 Dataset — This dataset contains brightfield image and corresponding synapsin stains for the VNC-free and VNC-containing small fragment cutting scenarios shown in Fig 6. Each image is labeled in the format “x_dpc_Sample_y_tn.jpg”, where “x” represents the number of days post cutting and “y” the replicate number. (ZIP) [file pcbi.1006904.s016.zip › smallfragments/VNC-containing/synapsin_stains/14 dpc_Sample 2_tn.jpg]

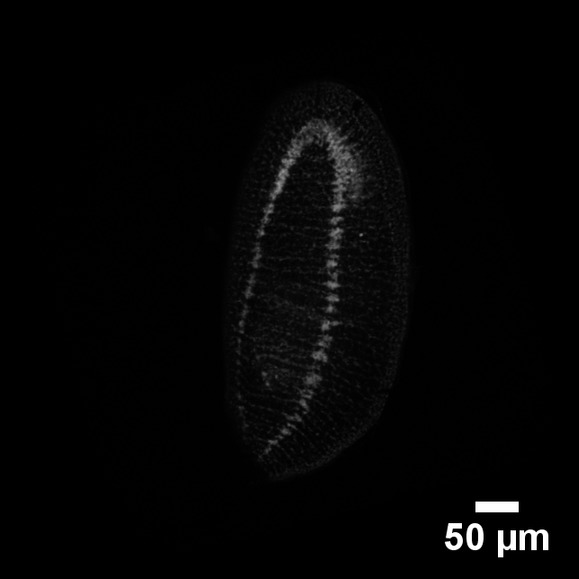

Supplement: S1 Dataset — This dataset contains brightfield image and corresponding synapsin stains for the VNC-free and VNC-containing small fragment cutting scenarios shown in Fig 6. Each image is labeled in the format “x_dpc_Sample_y_tn.jpg”, where “x” represents the number of days post cutting and “y” the replicate number. (ZIP) [file pcbi.1006904.s016.zip › smallfragments/VNC-containing/synapsin_stains/14 dpc_Sample 3_tn.jpg]

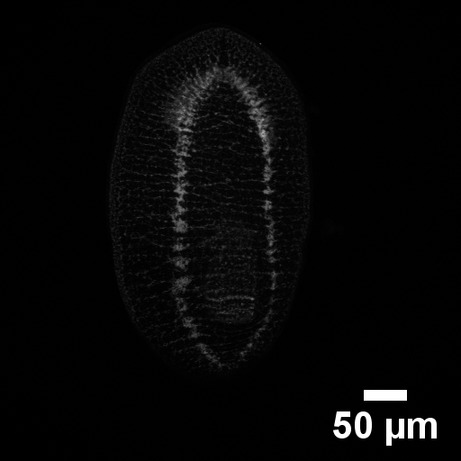

Supplement: S1 Dataset — This dataset contains brightfield image and corresponding synapsin stains for the VNC-free and VNC-containing small fragment cutting scenarios shown in Fig 6. Each image is labeled in the format “x_dpc_Sample_y_tn.jpg”, where “x” represents the number of days post cutting and “y” the replicate number. (ZIP) [file pcbi.1006904.s016.zip › smallfragments/VNC-containing/synapsin_stains/14 dpc_Sample 4_tn.jpg]

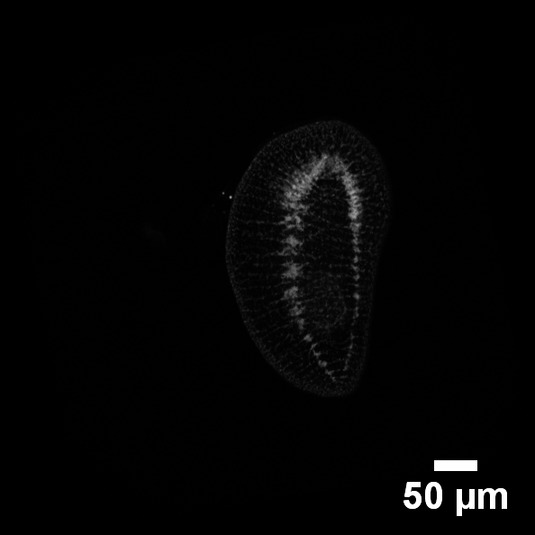

Supplement: S1 Dataset — This dataset contains brightfield image and corresponding synapsin stains for the VNC-free and VNC-containing small fragment cutting scenarios shown in Fig 6. Each image is labeled in the format “x_dpc_Sample_y_tn.jpg”, where “x” represents the number of days post cutting and “y” the replicate number. (ZIP) [file pcbi.1006904.s016.zip › smallfragments/VNC-containing/synapsin_stains/14 dpc_Sample 5_tn.jpg]

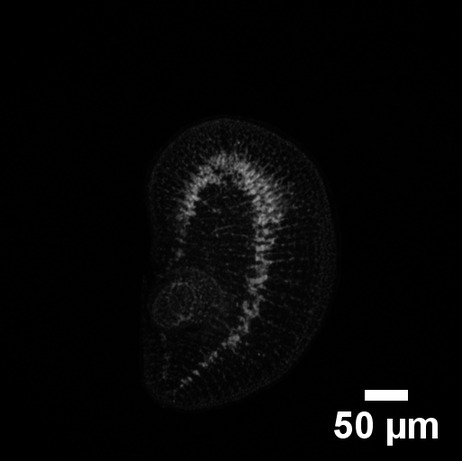

Supplement: S1 Dataset — This dataset contains brightfield image and corresponding synapsin stains for the VNC-free and VNC-containing small fragment cutting scenarios shown in Fig 6. Each image is labeled in the format “x_dpc_Sample_y_tn.jpg”, where “x” represents the number of days post cutting and “y” the replicate number. (ZIP) [file pcbi.1006904.s016.zip › smallfragments/VNC-containing/synapsin_stains/14 dpc_Sample 6_tn.jpg]

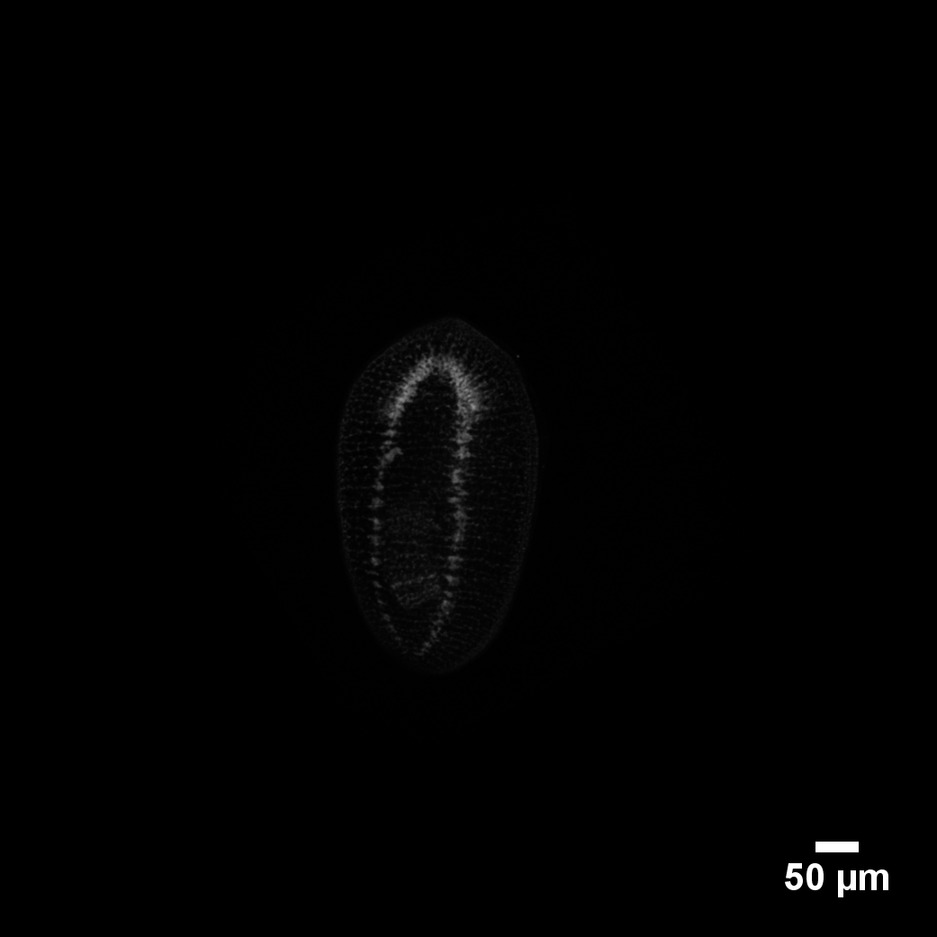

Supplement: S1 Dataset — This dataset contains brightfield image and corresponding synapsin stains for the VNC-free and VNC-containing small fragment cutting scenarios shown in Fig 6. Each image is labeled in the format “x_dpc_Sample_y_tn.jpg”, where “x” represents the number of days post cutting and “y” the replicate number. (ZIP) [file pcbi.1006904.s016.zip › smallfragments/VNC-containing/synapsin_stains/14 dpc_Sample 7_tn.jpg]

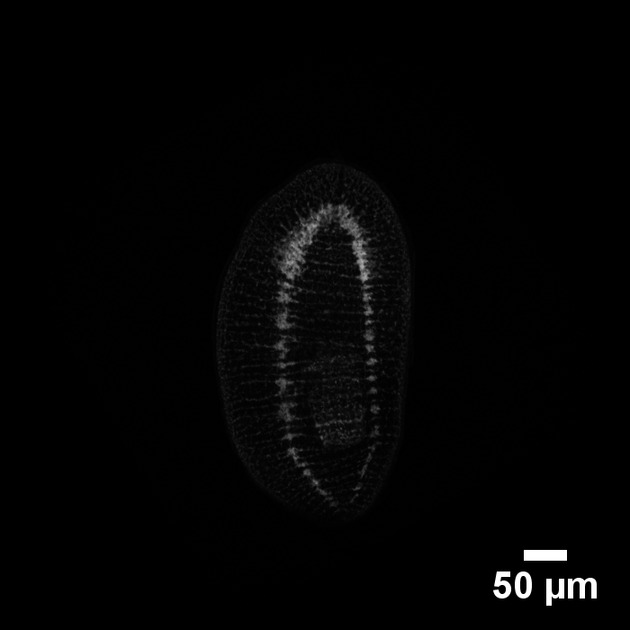

Supplement: S1 Dataset — This dataset contains brightfield image and corresponding synapsin stains for the VNC-free and VNC-containing small fragment cutting scenarios shown in Fig 6. Each image is labeled in the format “x_dpc_Sample_y_tn.jpg”, where “x” represents the number of days post cutting and “y” the replicate number. (ZIP) [file pcbi.1006904.s016.zip › smallfragments/VNC-containing/synapsin_stains/14 dpc_Sample 8_tn.jpg]

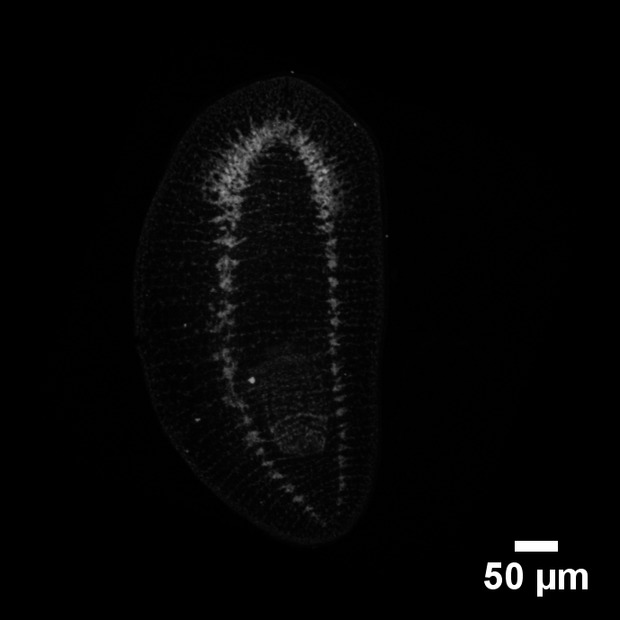

Supplement: S1 Dataset — This dataset contains brightfield image and corresponding synapsin stains for the VNC-free and VNC-containing small fragment cutting scenarios shown in Fig 6. Each image is labeled in the format “x_dpc_Sample_y_tn.jpg”, where “x” represents the number of days post cutting and “y” the replicate number. (ZIP) [file pcbi.1006904.s016.zip › smallfragments/VNC-containing/synapsin_stains/14 dpc_Sample 9_tn.jpg]

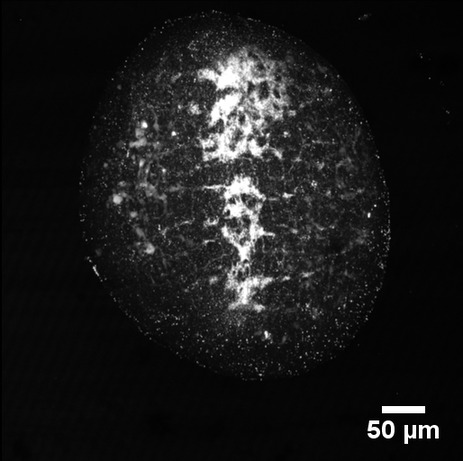

Supplement: S1 Dataset — This dataset contains brightfield image and corresponding synapsin stains for the VNC-free and VNC-containing small fragment cutting scenarios shown in Fig 6. Each image is labeled in the format “x_dpc_Sample_y_tn.jpg”, where “x” represents the number of days post cutting and “y” the replicate number. (ZIP) [file pcbi.1006904.s016.zip › smallfragments/VNC-containing/synapsin_stains/2 dpc_Sample 1_tn.jpg]

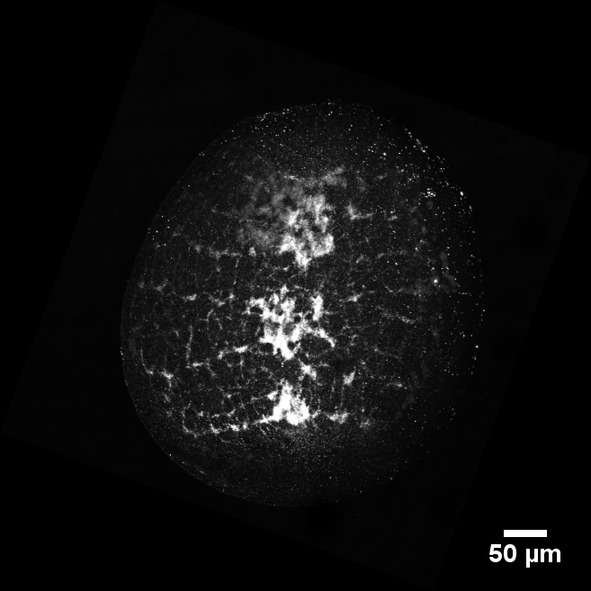

Supplement: S1 Dataset — This dataset contains brightfield image and corresponding synapsin stains for the VNC-free and VNC-containing small fragment cutting scenarios shown in Fig 6. Each image is labeled in the format “x_dpc_Sample_y_tn.jpg”, where “x” represents the number of days post cutting and “y” the replicate number. (ZIP) [file pcbi.1006904.s016.zip › smallfragments/VNC-containing/synapsin_stains/2 dpc_Sample 2_tn.jpg]

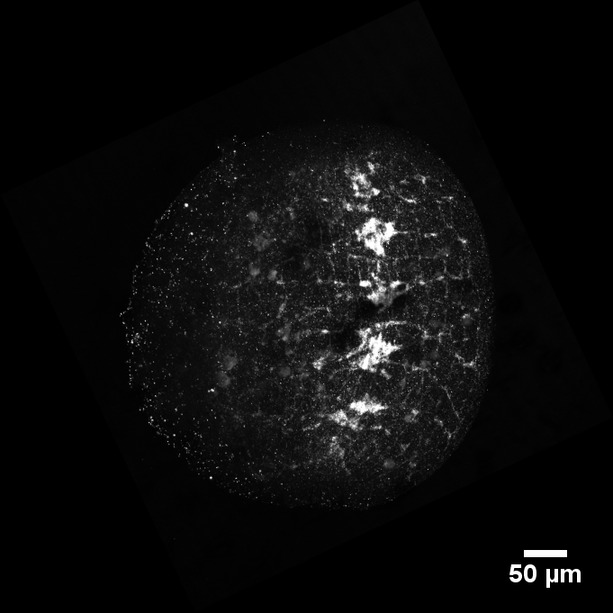

Supplement: S1 Dataset — This dataset contains brightfield image and corresponding synapsin stains for the VNC-free and VNC-containing small fragment cutting scenarios shown in Fig 6. Each image is labeled in the format “x_dpc_Sample_y_tn.jpg”, where “x” represents the number of days post cutting and “y” the replicate number. (ZIP) [file pcbi.1006904.s016.zip › smallfragments/VNC-containing/synapsin_stains/2 dpc_Sample 3_tn.jpg]

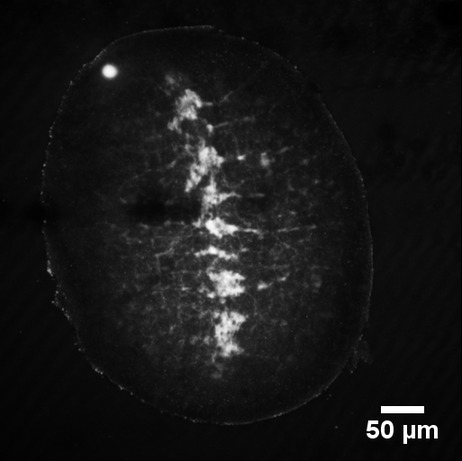

Supplement: S1 Dataset — This dataset contains brightfield image and corresponding synapsin stains for the VNC-free and VNC-containing small fragment cutting scenarios shown in Fig 6. Each image is labeled in the format “x_dpc_Sample_y_tn.jpg”, where “x” represents the number of days post cutting and “y” the replicate number. (ZIP) [file pcbi.1006904.s016.zip › smallfragments/VNC-containing/synapsin_stains/2 dpc_Sample 4_tn.jpg]

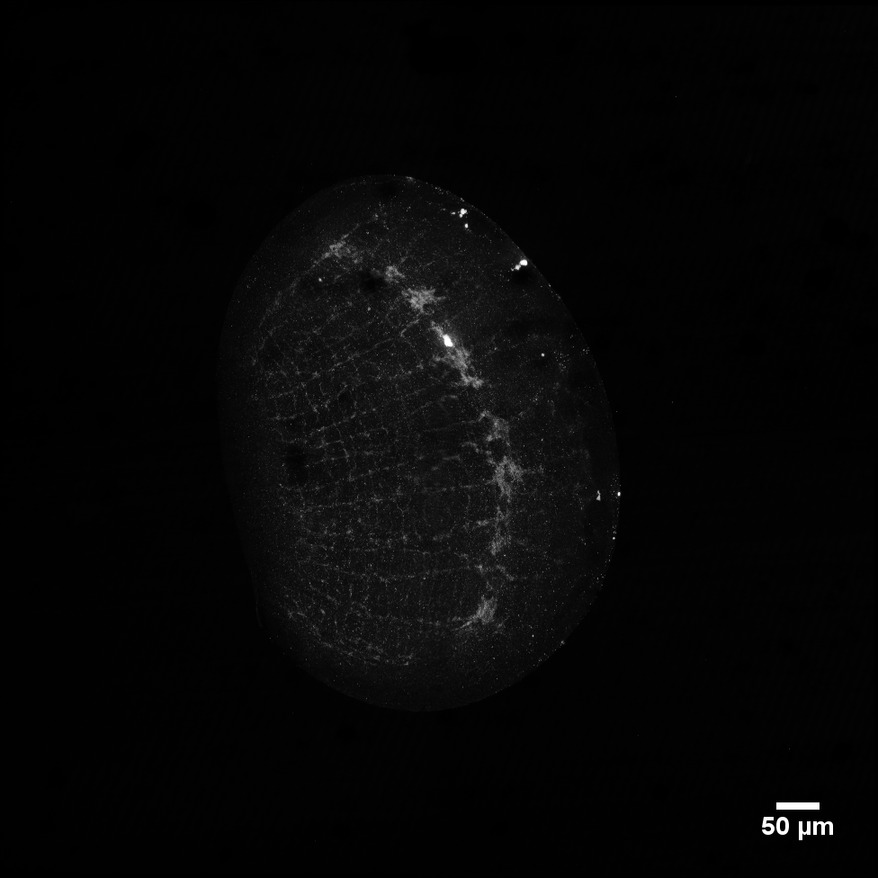

Supplement: S1 Dataset — This dataset contains brightfield image and corresponding synapsin stains for the VNC-free and VNC-containing small fragment cutting scenarios shown in Fig 6. Each image is labeled in the format “x_dpc_Sample_y_tn.jpg”, where “x” represents the number of days post cutting and “y” the replicate number. (ZIP) [file pcbi.1006904.s016.zip › smallfragments/VNC-containing/synapsin_stains/2 dpc_Sample 5_tn.jpg]

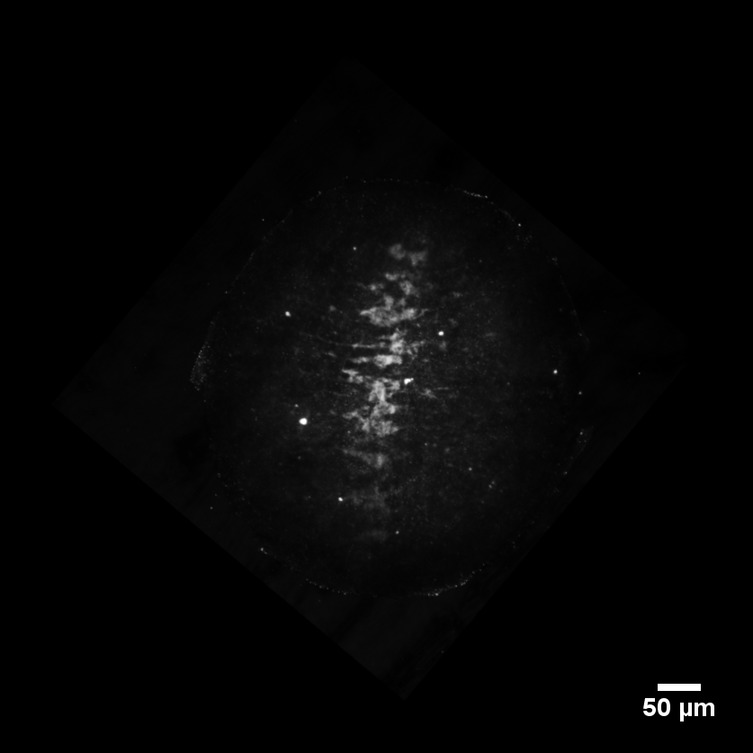

Supplement: S1 Dataset — This dataset contains brightfield image and corresponding synapsin stains for the VNC-free and VNC-containing small fragment cutting scenarios shown in Fig 6. Each image is labeled in the format “x_dpc_Sample_y_tn.jpg”, where “x” represents the number of days post cutting and “y” the replicate number. (ZIP) [file pcbi.1006904.s016.zip › smallfragments/VNC-containing/synapsin_stains/2 dpc_Sample 6_tn.jpg]

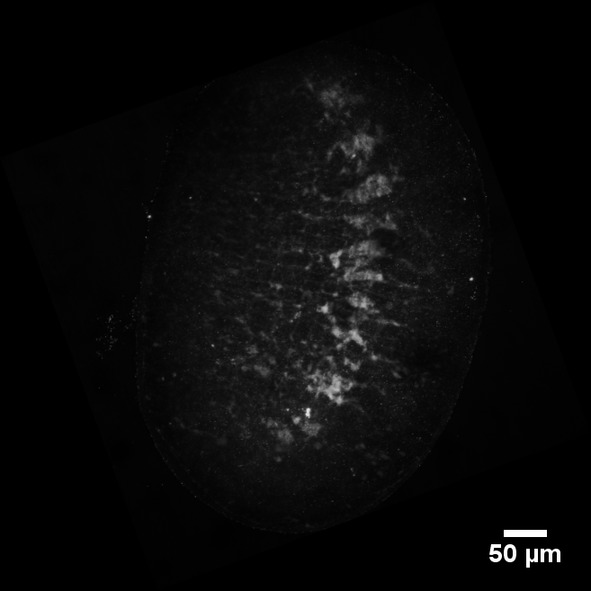

Supplement: S1 Dataset — This dataset contains brightfield image and corresponding synapsin stains for the VNC-free and VNC-containing small fragment cutting scenarios shown in Fig 6. Each image is labeled in the format “x_dpc_Sample_y_tn.jpg”, where “x” represents the number of days post cutting and “y” the replicate number. (ZIP) [file pcbi.1006904.s016.zip › smallfragments/VNC-containing/synapsin_stains/2 dpc_Sample 7_tn.jpg]

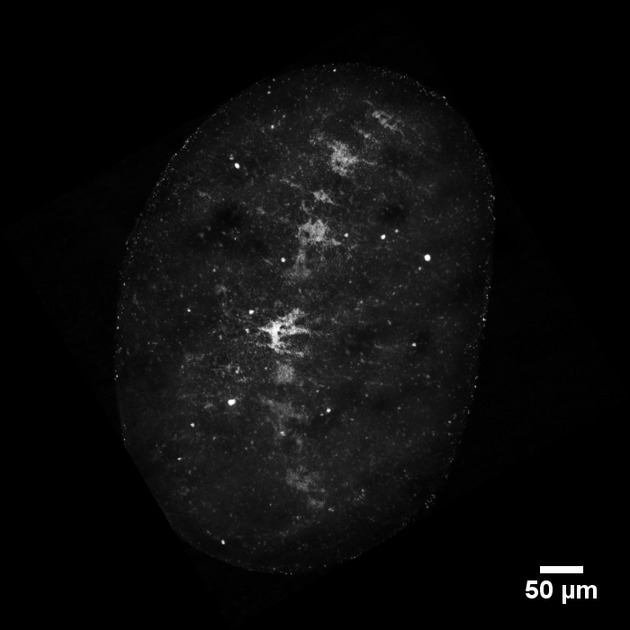

Supplement: S1 Dataset — This dataset contains brightfield image and corresponding synapsin stains for the VNC-free and VNC-containing small fragment cutting scenarios shown in Fig 6. Each image is labeled in the format “x_dpc_Sample_y_tn.jpg”, where “x” represents the number of days post cutting and “y” the replicate number. (ZIP) [file pcbi.1006904.s016.zip › smallfragments/VNC-containing/synapsin_stains/2 dpc_Sample 8_tn.jpg]

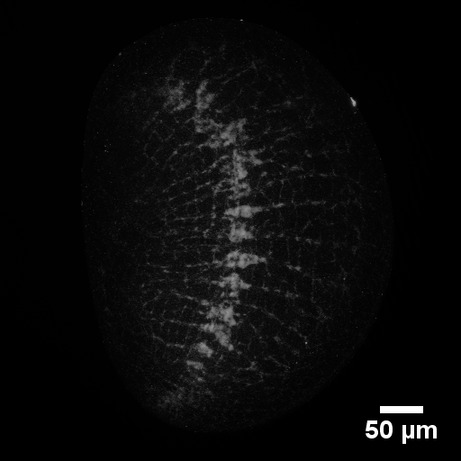

Supplement: S1 Dataset — This dataset contains brightfield image and corresponding synapsin stains for the VNC-free and VNC-containing small fragment cutting scenarios shown in Fig 6. Each image is labeled in the format “x_dpc_Sample_y_tn.jpg”, where “x” represents the number of days post cutting and “y” the replicate number. (ZIP) [file pcbi.1006904.s016.zip › smallfragments/VNC-containing/synapsin_stains/3 dpc_Sample 1_tn.jpg]

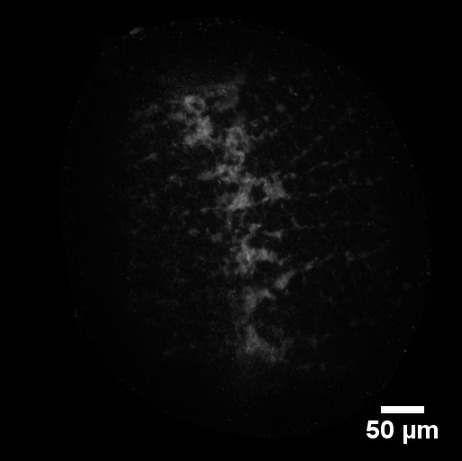

Supplement: S1 Dataset — This dataset contains brightfield image and corresponding synapsin stains for the VNC-free and VNC-containing small fragment cutting scenarios shown in Fig 6. Each image is labeled in the format “x_dpc_Sample_y_tn.jpg”, where “x” represents the number of days post cutting and “y” the replicate number. (ZIP) [file pcbi.1006904.s016.zip › smallfragments/VNC-containing/synapsin_stains/3 dpc_Sample 2_tn.jpg]

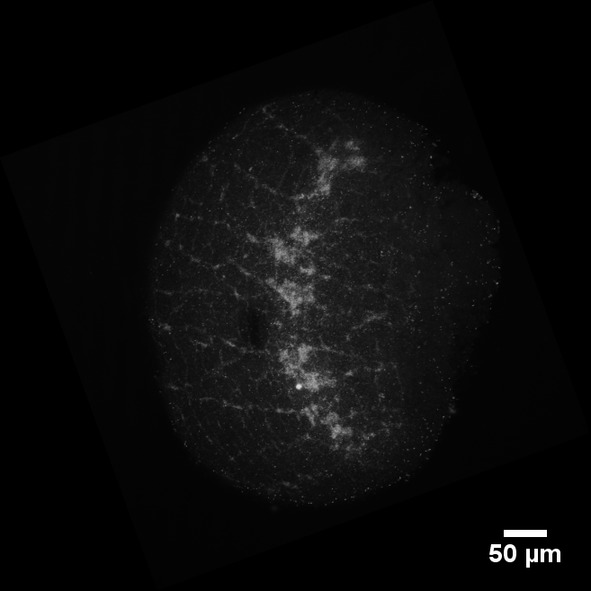

Supplement: S1 Dataset — This dataset contains brightfield image and corresponding synapsin stains for the VNC-free and VNC-containing small fragment cutting scenarios shown in Fig 6. Each image is labeled in the format “x_dpc_Sample_y_tn.jpg”, where “x” represents the number of days post cutting and “y” the replicate number. (ZIP) [file pcbi.1006904.s016.zip › smallfragments/VNC-containing/synapsin_stains/3 dpc_Sample 3_tn.jpg]

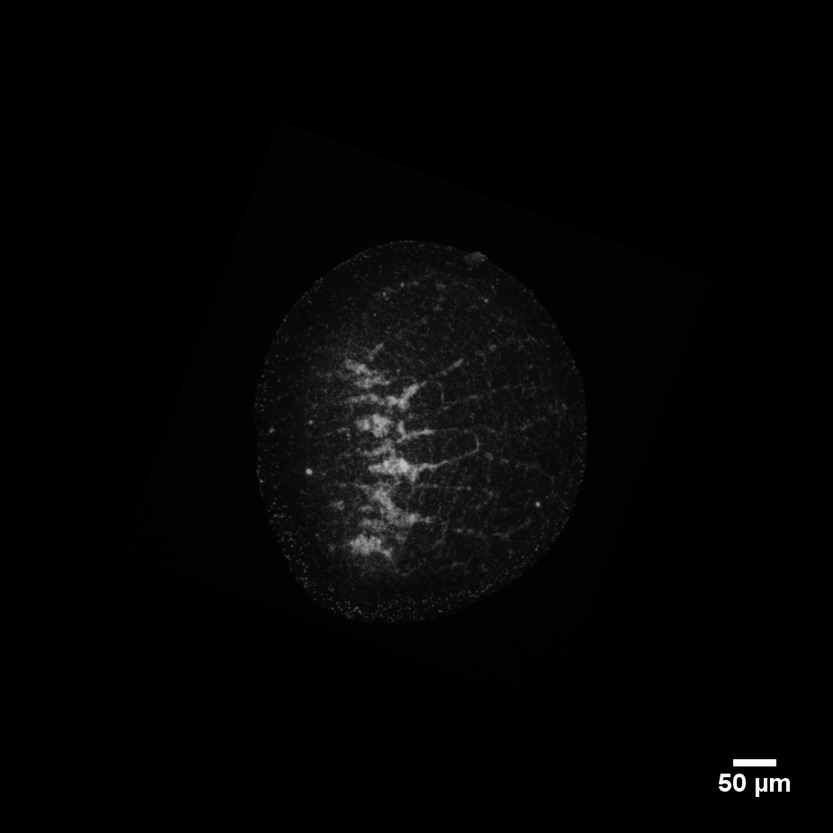

Supplement: S1 Dataset — This dataset contains brightfield image and corresponding synapsin stains for the VNC-free and VNC-containing small fragment cutting scenarios shown in Fig 6. Each image is labeled in the format “x_dpc_Sample_y_tn.jpg”, where “x” represents the number of days post cutting and “y” the replicate number. (ZIP) [file pcbi.1006904.s016.zip › smallfragments/VNC-containing/synapsin_stains/3 dpc_Sample 4_tn.jpg]

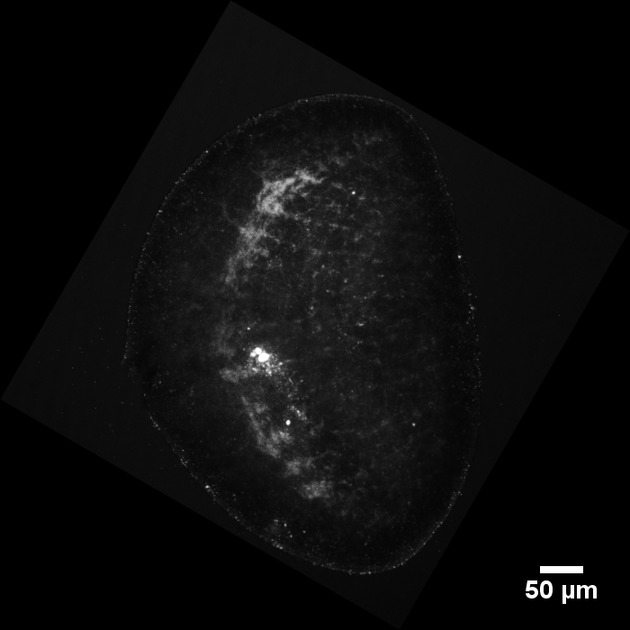

Supplement: S1 Dataset — This dataset contains brightfield image and corresponding synapsin stains for the VNC-free and VNC-containing small fragment cutting scenarios shown in Fig 6. Each image is labeled in the format “x_dpc_Sample_y_tn.jpg”, where “x” represents the number of days post cutting and “y” the replicate number. (ZIP) [file pcbi.1006904.s016.zip › smallfragments/VNC-containing/synapsin_stains/4 dpc_Sample 1_tn.jpg]

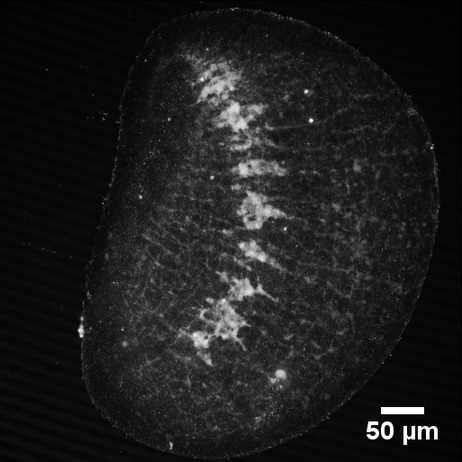

Supplement: S1 Dataset — This dataset contains brightfield image and corresponding synapsin stains for the VNC-free and VNC-containing small fragment cutting scenarios shown in Fig 6. Each image is labeled in the format “x_dpc_Sample_y_tn.jpg”, where “x” represents the number of days post cutting and “y” the replicate number. (ZIP) [file pcbi.1006904.s016.zip › smallfragments/VNC-containing/synapsin_stains/4 dpc_Sample 2_tn.jpg]

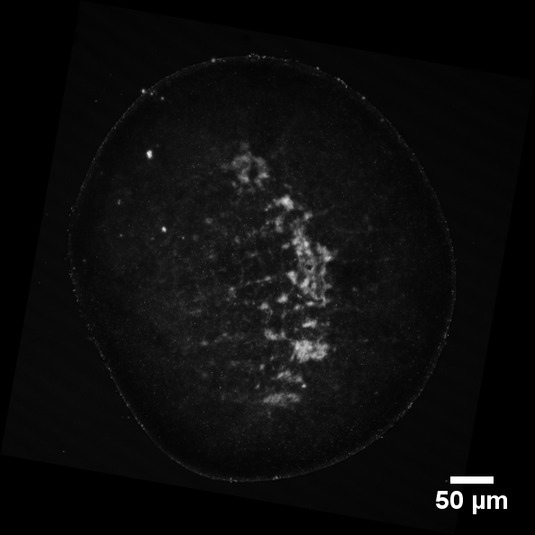

Supplement: S1 Dataset — This dataset contains brightfield image and corresponding synapsin stains for the VNC-free and VNC-containing small fragment cutting scenarios shown in Fig 6. Each image is labeled in the format “x_dpc_Sample_y_tn.jpg”, where “x” represents the number of days post cutting and “y” the replicate number. (ZIP) [file pcbi.1006904.s016.zip › smallfragments/VNC-containing/synapsin_stains/4 dpc_Sample 3_tn.jpg]

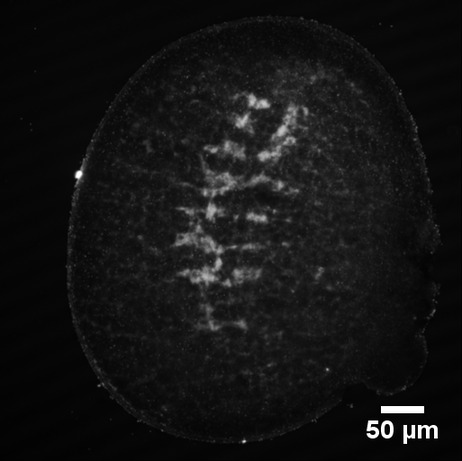

Supplement: S1 Dataset — This dataset contains brightfield image and corresponding synapsin stains for the VNC-free and VNC-containing small fragment cutting scenarios shown in Fig 6. Each image is labeled in the format “x_dpc_Sample_y_tn.jpg”, where “x” represents the number of days post cutting and “y” the replicate number. (ZIP) [file pcbi.1006904.s016.zip › smallfragments/VNC-containing/synapsin_stains/4 dpc_Sample 4_tn.jpg]

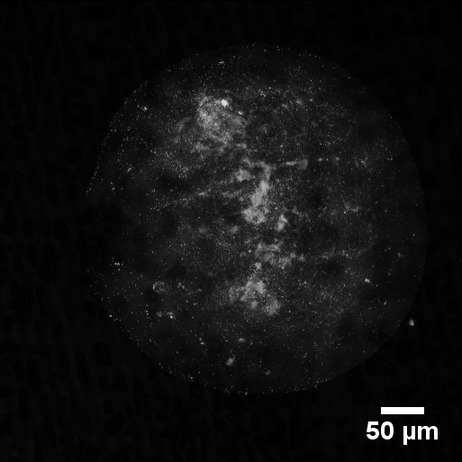

Supplement: S1 Dataset — This dataset contains brightfield image and corresponding synapsin stains for the VNC-free and VNC-containing small fragment cutting scenarios shown in Fig 6. Each image is labeled in the format “x_dpc_Sample_y_tn.jpg”, where “x” represents the number of days post cutting and “y” the replicate number. (ZIP) [file pcbi.1006904.s016.zip › smallfragments/VNC-containing/synapsin_stains/5 dpc_Sample 1_tn.jpg]

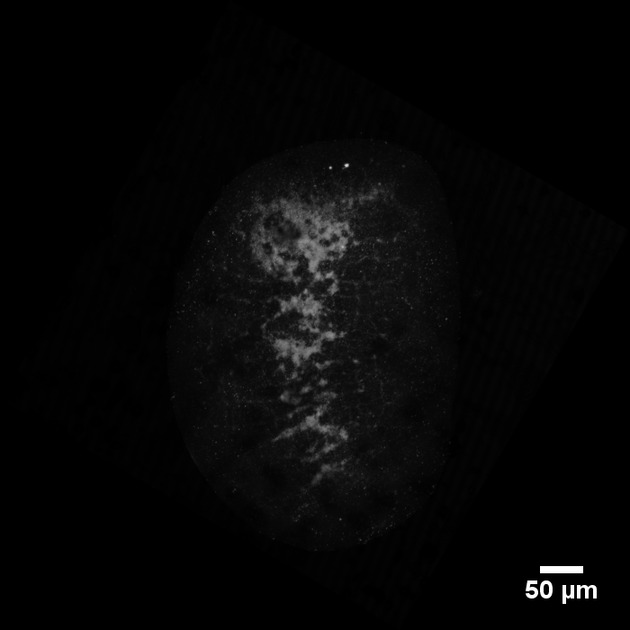

Supplement: S1 Dataset — This dataset contains brightfield image and corresponding synapsin stains for the VNC-free and VNC-containing small fragment cutting scenarios shown in Fig 6. Each image is labeled in the format “x_dpc_Sample_y_tn.jpg”, where “x” represents the number of days post cutting and “y” the replicate number. (ZIP) [file pcbi.1006904.s016.zip › smallfragments/VNC-containing/synapsin_stains/5 dpc_Sample 2_tn.jpg]

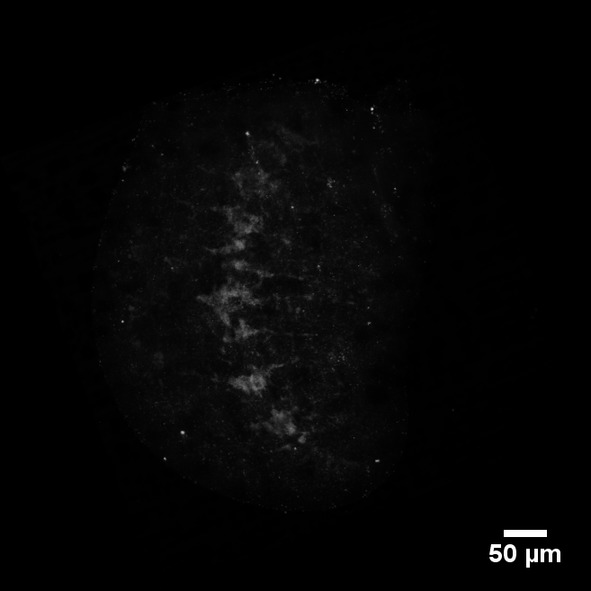

Supplement: S1 Dataset — This dataset contains brightfield image and corresponding synapsin stains for the VNC-free and VNC-containing small fragment cutting scenarios shown in Fig 6. Each image is labeled in the format “x_dpc_Sample_y_tn.jpg”, where “x” represents the number of days post cutting and “y” the replicate number. (ZIP) [file pcbi.1006904.s016.zip › smallfragments/VNC-containing/synapsin_stains/5 dpc_Sample 3_tn.jpg]

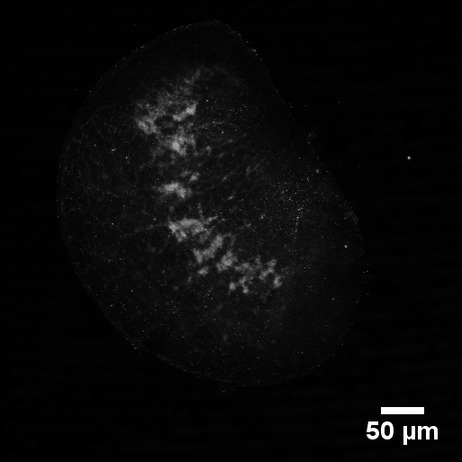

Supplement: S1 Dataset — This dataset contains brightfield image and corresponding synapsin stains for the VNC-free and VNC-containing small fragment cutting scenarios shown in Fig 6. Each image is labeled in the format “x_dpc_Sample_y_tn.jpg”, where “x” represents the number of days post cutting and “y” the replicate number. (ZIP) [file pcbi.1006904.s016.zip › smallfragments/VNC-containing/synapsin_stains/5 dpc_Sample 4_tn.jpg]

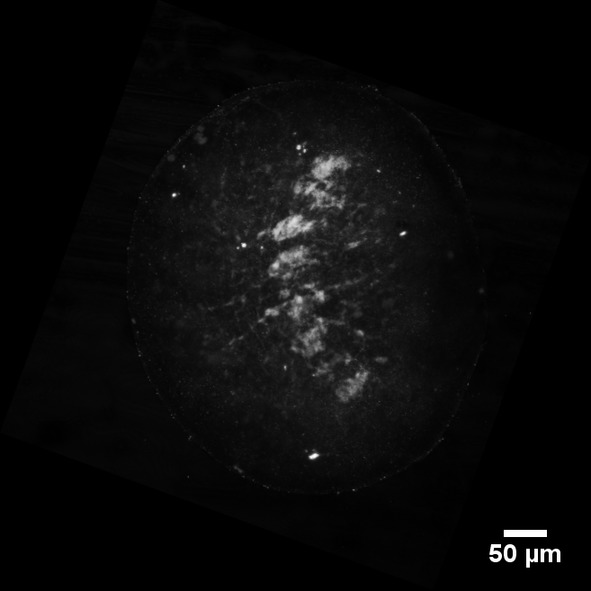

Supplement: S1 Dataset — This dataset contains brightfield image and corresponding synapsin stains for the VNC-free and VNC-containing small fragment cutting scenarios shown in Fig 6. Each image is labeled in the format “x_dpc_Sample_y_tn.jpg”, where “x” represents the number of days post cutting and “y” the replicate number. (ZIP) [file pcbi.1006904.s016.zip › smallfragments/VNC-containing/synapsin_stains/5 dpc_Sample 5_tn.jpg]

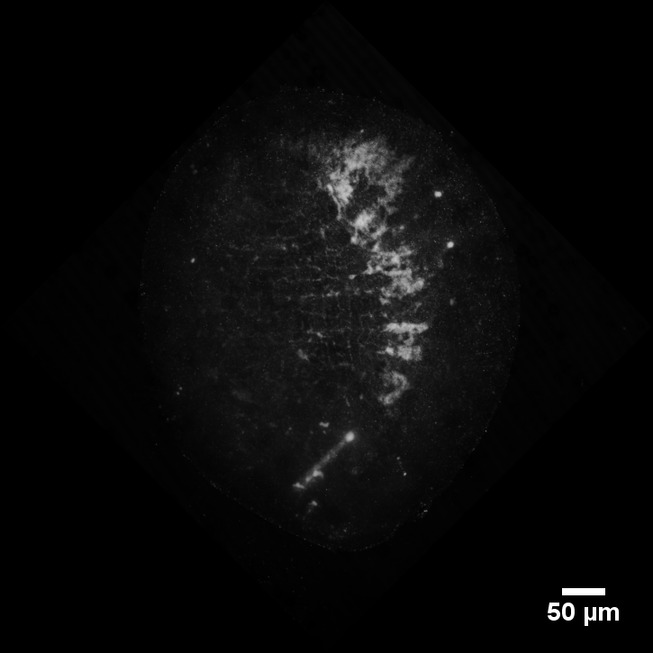

Supplement: S1 Dataset — This dataset contains brightfield image and corresponding synapsin stains for the VNC-free and VNC-containing small fragment cutting scenarios shown in Fig 6. Each image is labeled in the format “x_dpc_Sample_y_tn.jpg”, where “x” represents the number of days post cutting and “y” the replicate number. (ZIP) [file pcbi.1006904.s016.zip › smallfragments/VNC-containing/synapsin_stains/5 dpc_Sample 6_tn.jpg]
