# Supplementary figures and images for: Neural control of body-plan axis in regenerating planaria (part 3 of 4)
Source: PLoS Comput Biol. 2019 Apr 16;15(4):e1006904. doi: 10.1371/journal.pcbi.1006904 (PMC6485777; doi:10.1371/journal.pcbi.1006904)

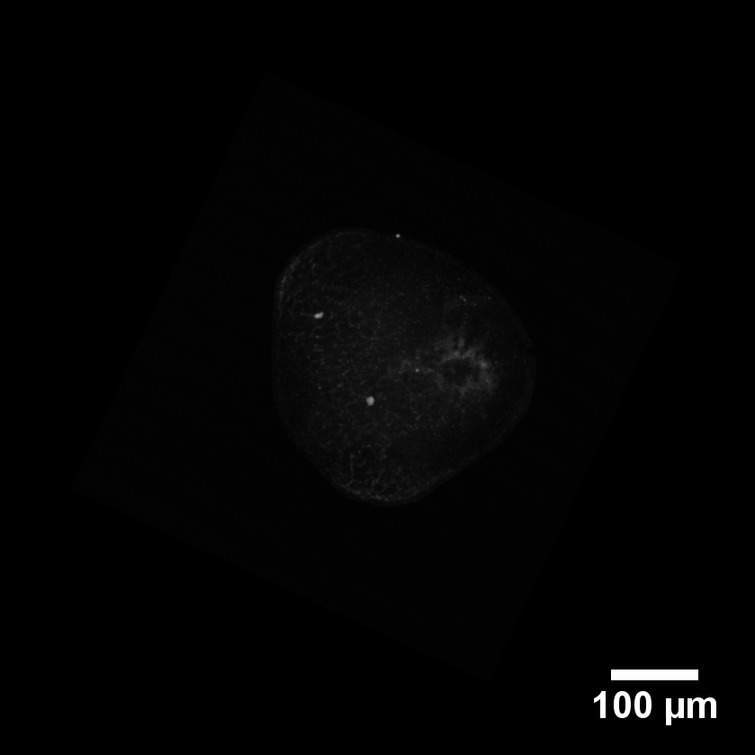

Supplement: S1 Dataset — This dataset contains brightfield image and corresponding synapsin stains for the VNC-free and VNC-containing small fragment cutting scenarios shown in Fig 6. Each image is labeled in the format “x_dpc_Sample_y_tn.jpg”, where “x” represents the number of days post cutting and “y” the replicate number. (ZIP) [file pcbi.1006904.s016.zip › smallfragments/VNC-free/synapsin_stains/7 dpc_Sample 5_tn.jpg]

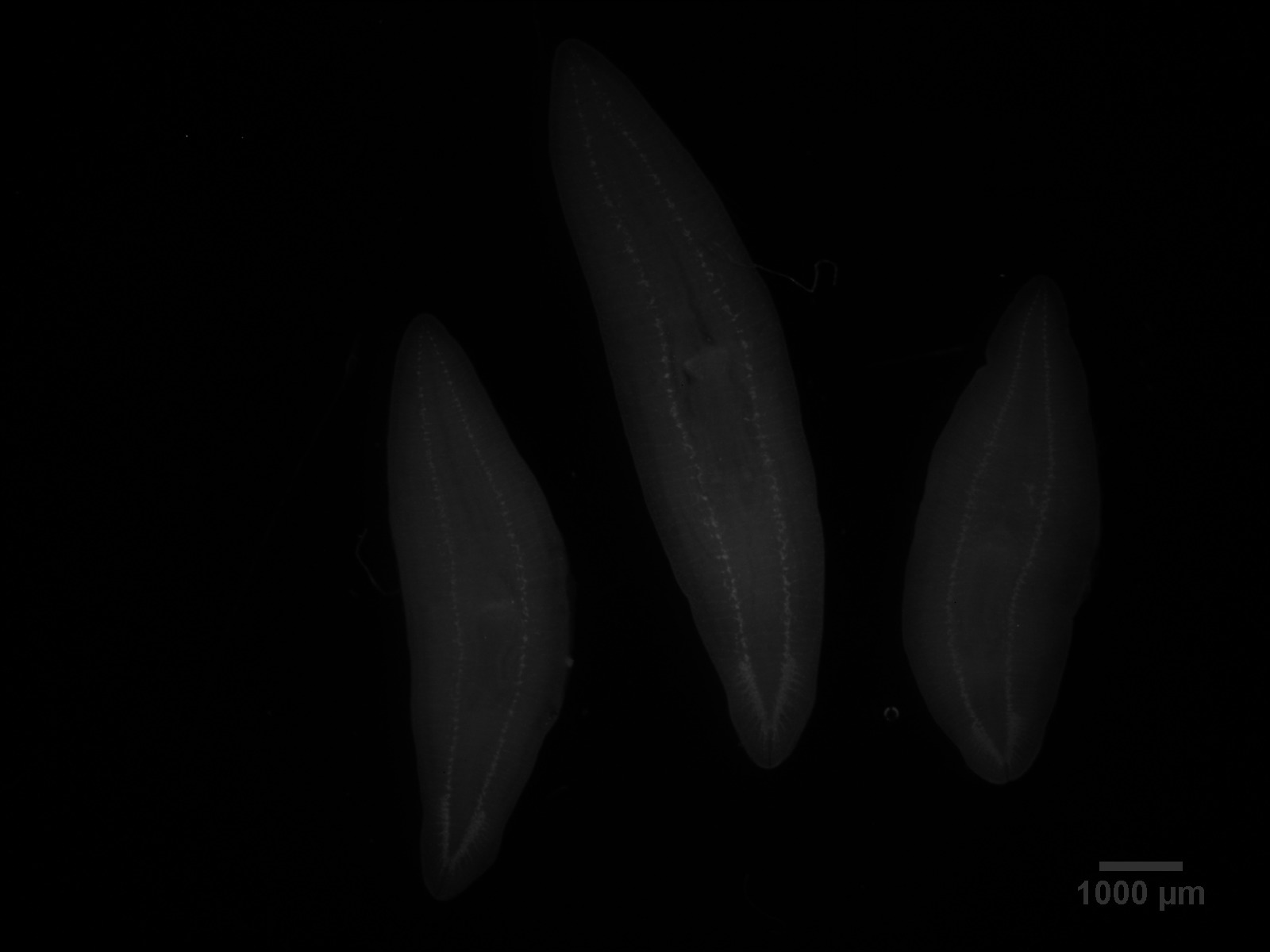

Supplement: S2 Dataset — This dataset contains raw-images of synapsin stains of uncut one- and two- headed worms, synapsin stains and brightfield images of the upwards and inverted L-cut scenarios, and synapsin stains and brightfield images showing the effects of the dynein inhibitor Ciliobrevin D on planaria regeneration. A Word document contained in the zip folder provides detailed description of the different cases. (ZIP) [file pcbi.1006904.s017.zip › DatasetS9i/1synapsinstain1H/Sample 10, 11, 12.jpg]

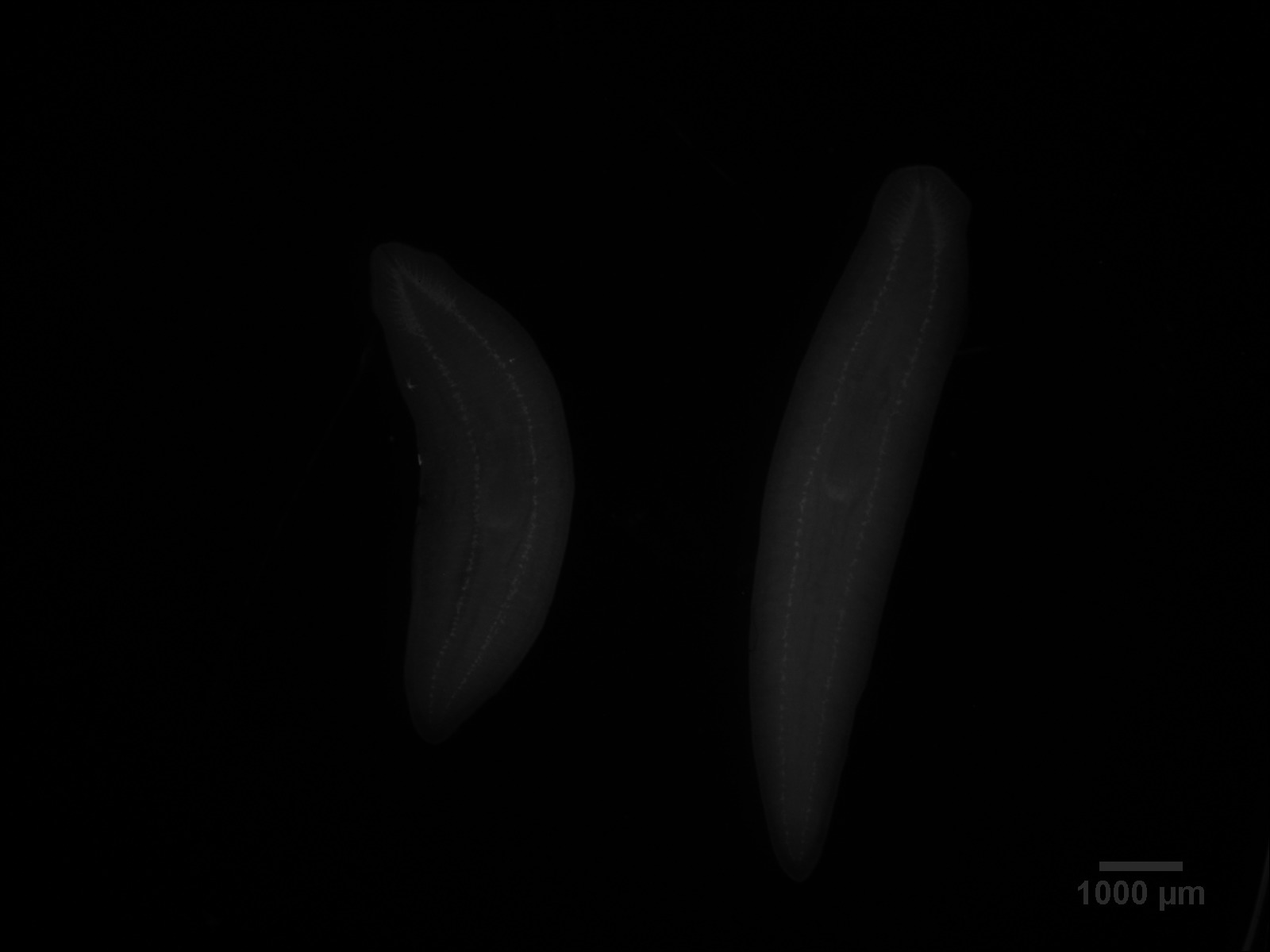

Supplement: S2 Dataset — This dataset contains raw-images of synapsin stains of uncut one- and two- headed worms, synapsin stains and brightfield images of the upwards and inverted L-cut scenarios, and synapsin stains and brightfield images showing the effects of the dynein inhibitor Ciliobrevin D on planaria regeneration. A Word document contained in the zip folder provides detailed description of the different cases. (ZIP) [file pcbi.1006904.s017.zip › DatasetS9i/1synapsinstain1H/Sample 1, 2.jpg]

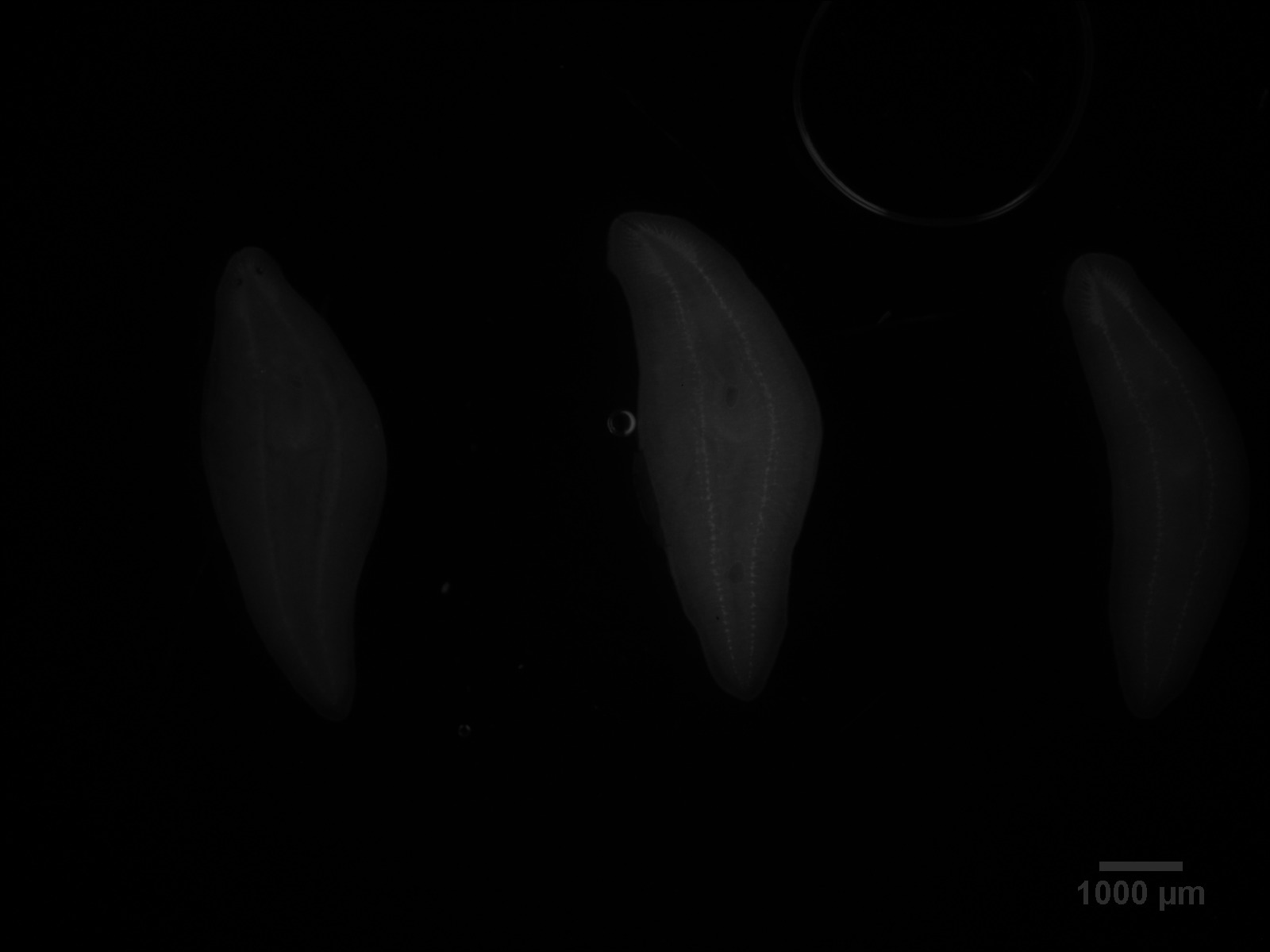

Supplement: S2 Dataset — This dataset contains raw-images of synapsin stains of uncut one- and two- headed worms, synapsin stains and brightfield images of the upwards and inverted L-cut scenarios, and synapsin stains and brightfield images showing the effects of the dynein inhibitor Ciliobrevin D on planaria regeneration. A Word document contained in the zip folder provides detailed description of the different cases. (ZIP) [file pcbi.1006904.s017.zip › DatasetS9i/1synapsinstain1H/Sample 13, 14, 15.jpg]

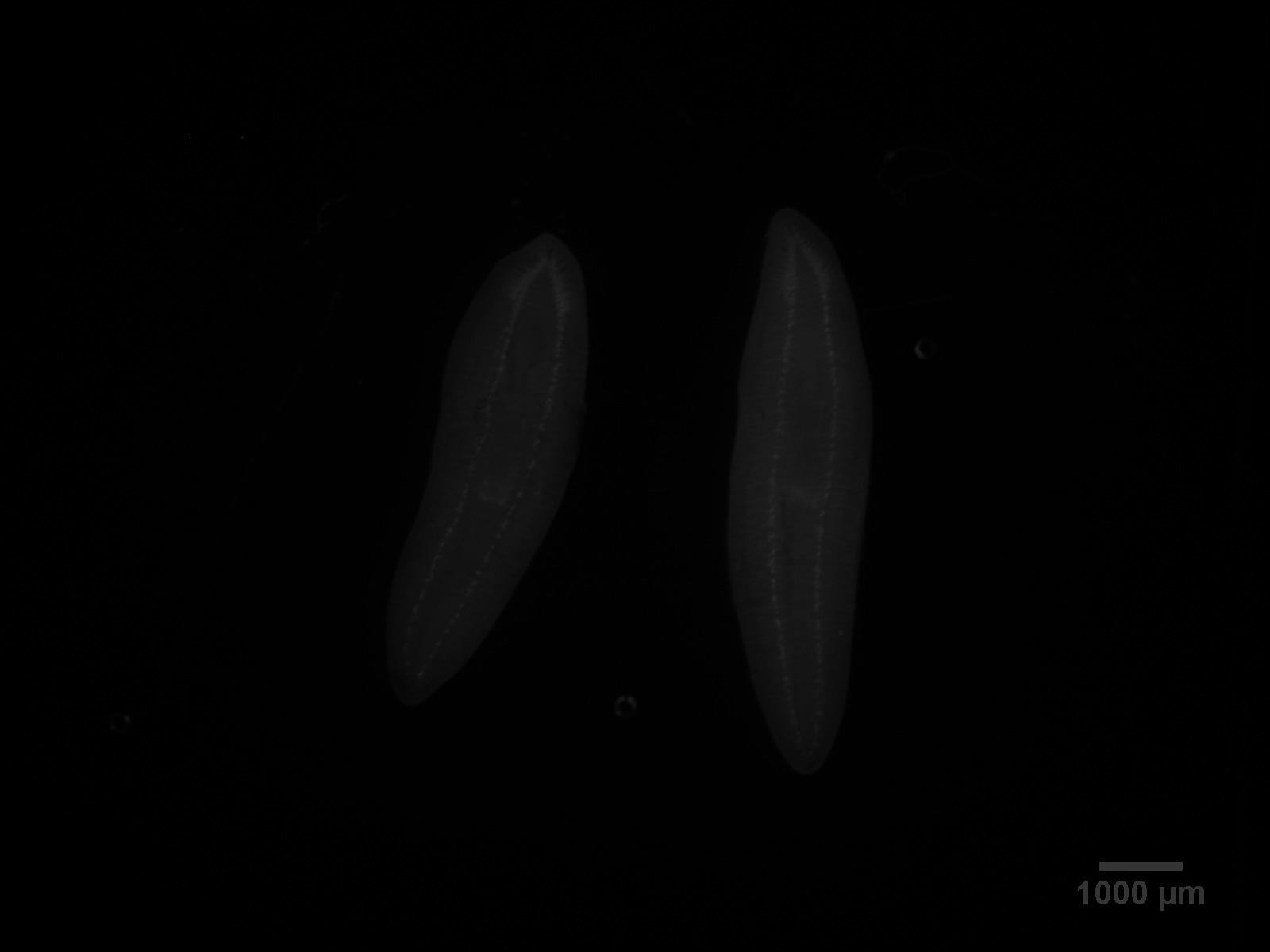

Supplement: S2 Dataset — This dataset contains raw-images of synapsin stains of uncut one- and two- headed worms, synapsin stains and brightfield images of the upwards and inverted L-cut scenarios, and synapsin stains and brightfield images showing the effects of the dynein inhibitor Ciliobrevin D on planaria regeneration. A Word document contained in the zip folder provides detailed description of the different cases. (ZIP) [file pcbi.1006904.s017.zip › DatasetS9i/1synapsinstain1H/Sample 3, 4.jpg]

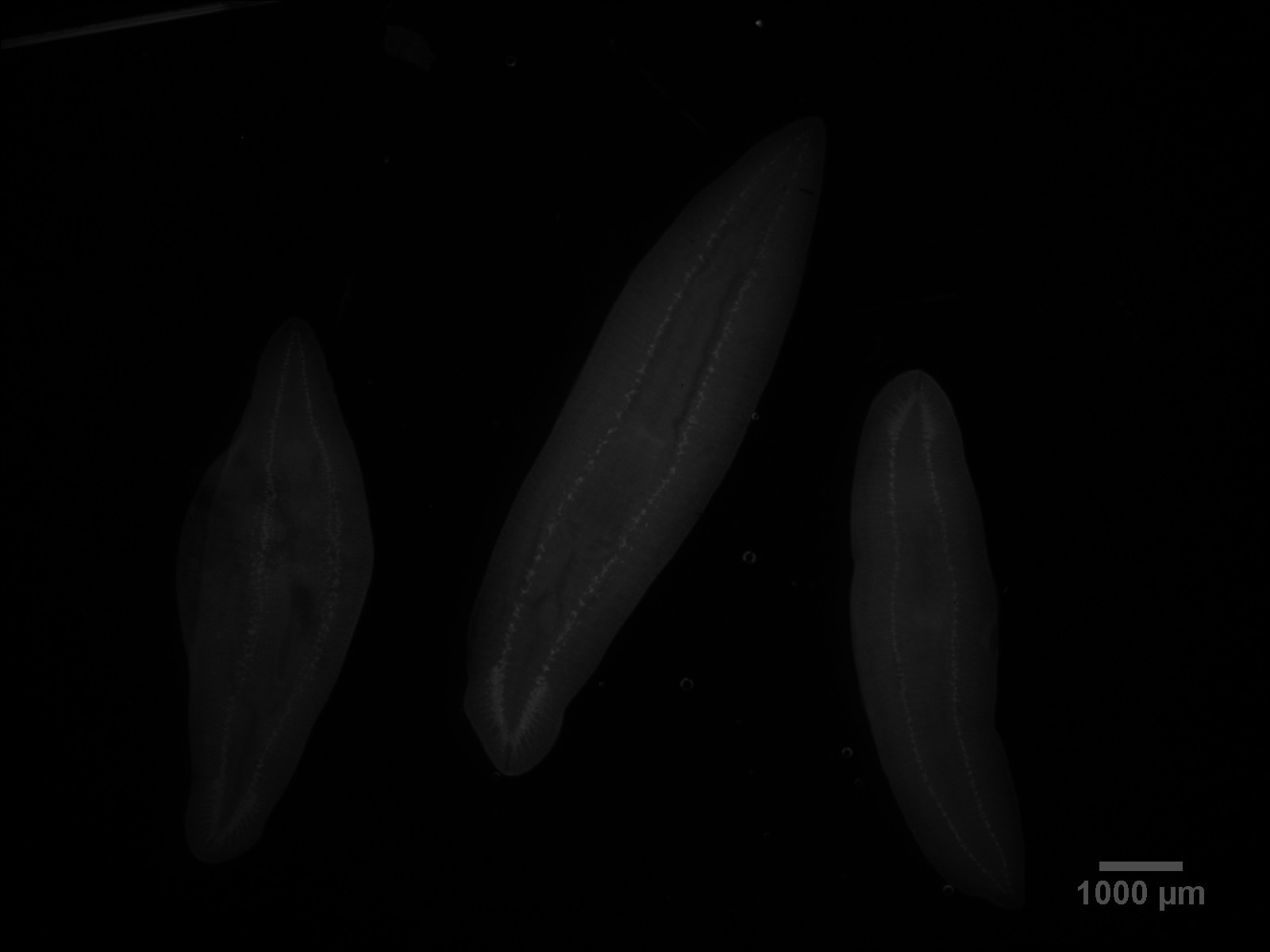

Supplement: S2 Dataset — This dataset contains raw-images of synapsin stains of uncut one- and two- headed worms, synapsin stains and brightfield images of the upwards and inverted L-cut scenarios, and synapsin stains and brightfield images showing the effects of the dynein inhibitor Ciliobrevin D on planaria regeneration. A Word document contained in the zip folder provides detailed description of the different cases. (ZIP) [file pcbi.1006904.s017.zip › DatasetS9i/1synapsinstain1H/Sample 5, 6, 7.jpg]

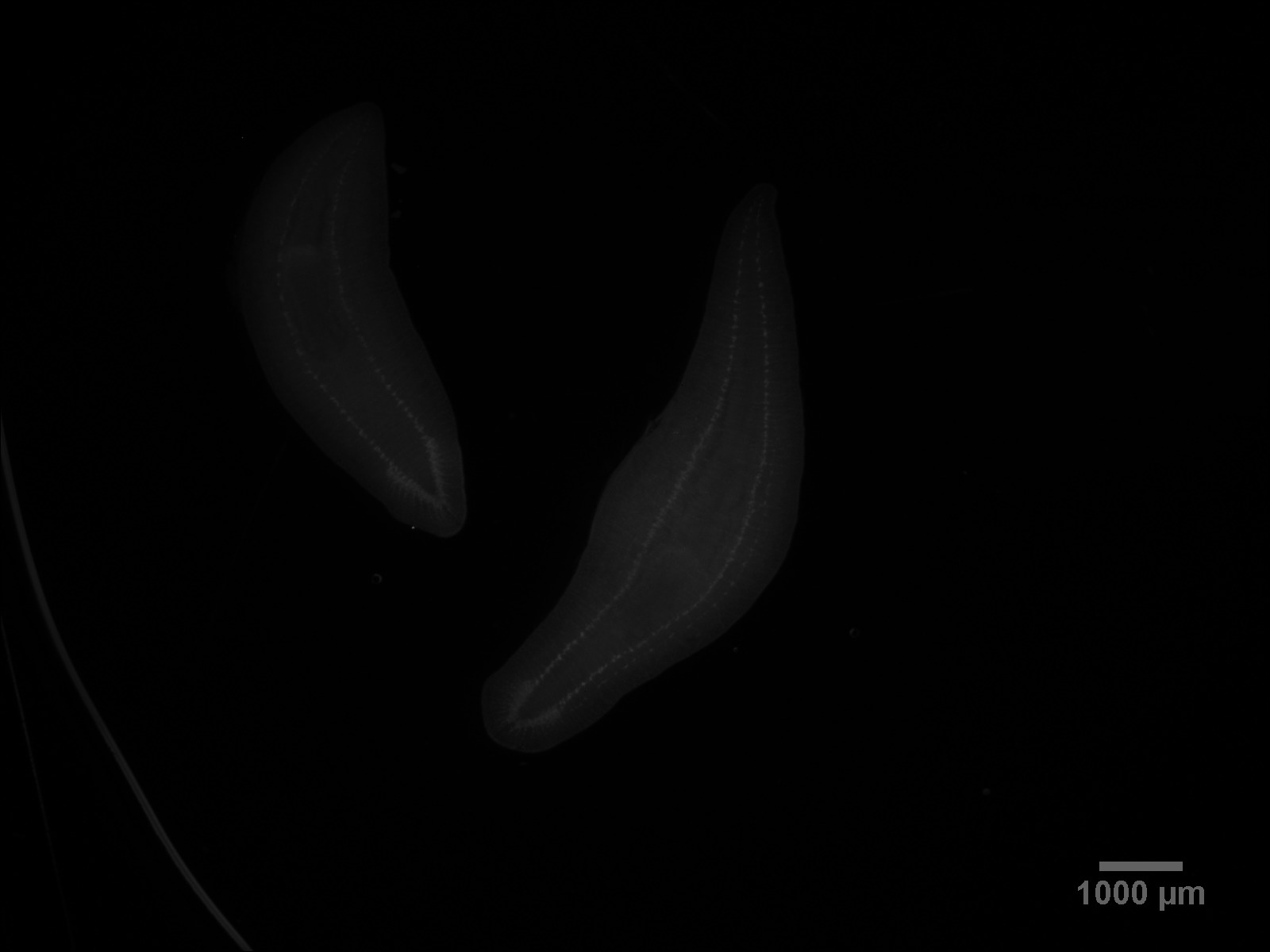

Supplement: S2 Dataset — This dataset contains raw-images of synapsin stains of uncut one- and two- headed worms, synapsin stains and brightfield images of the upwards and inverted L-cut scenarios, and synapsin stains and brightfield images showing the effects of the dynein inhibitor Ciliobrevin D on planaria regeneration. A Word document contained in the zip folder provides detailed description of the different cases. (ZIP) [file pcbi.1006904.s017.zip › DatasetS9i/1synapsinstain1H/Sample 8, 9.jpg]

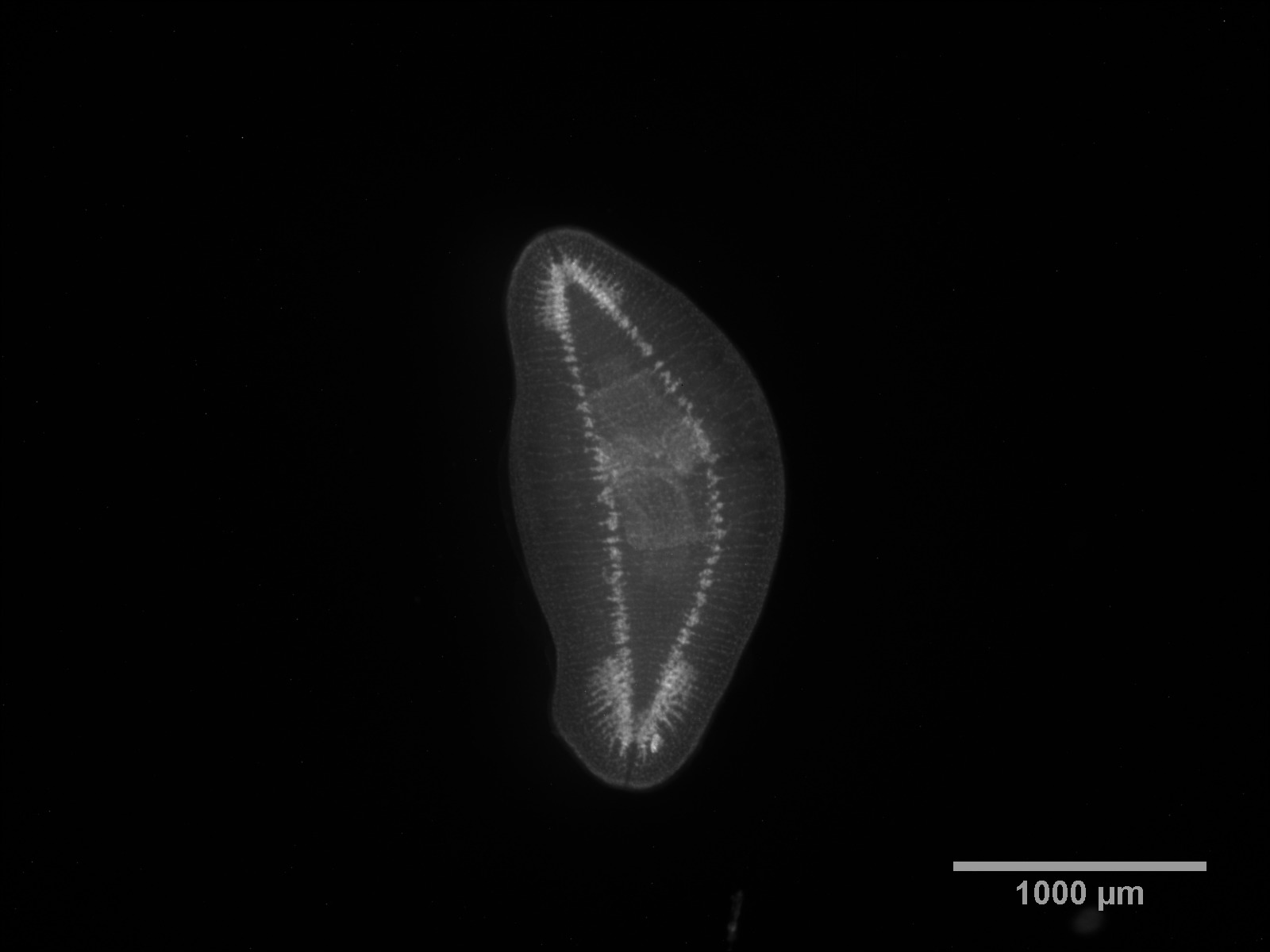

Supplement: S2 Dataset — This dataset contains raw-images of synapsin stains of uncut one- and two- headed worms, synapsin stains and brightfield images of the upwards and inverted L-cut scenarios, and synapsin stains and brightfield images showing the effects of the dynein inhibitor Ciliobrevin D on planaria regeneration. A Word document contained in the zip folder provides detailed description of the different cases. (ZIP) [file pcbi.1006904.s017.zip › DatasetS9i/2synapsinstain2H/Sample 10.jpg]

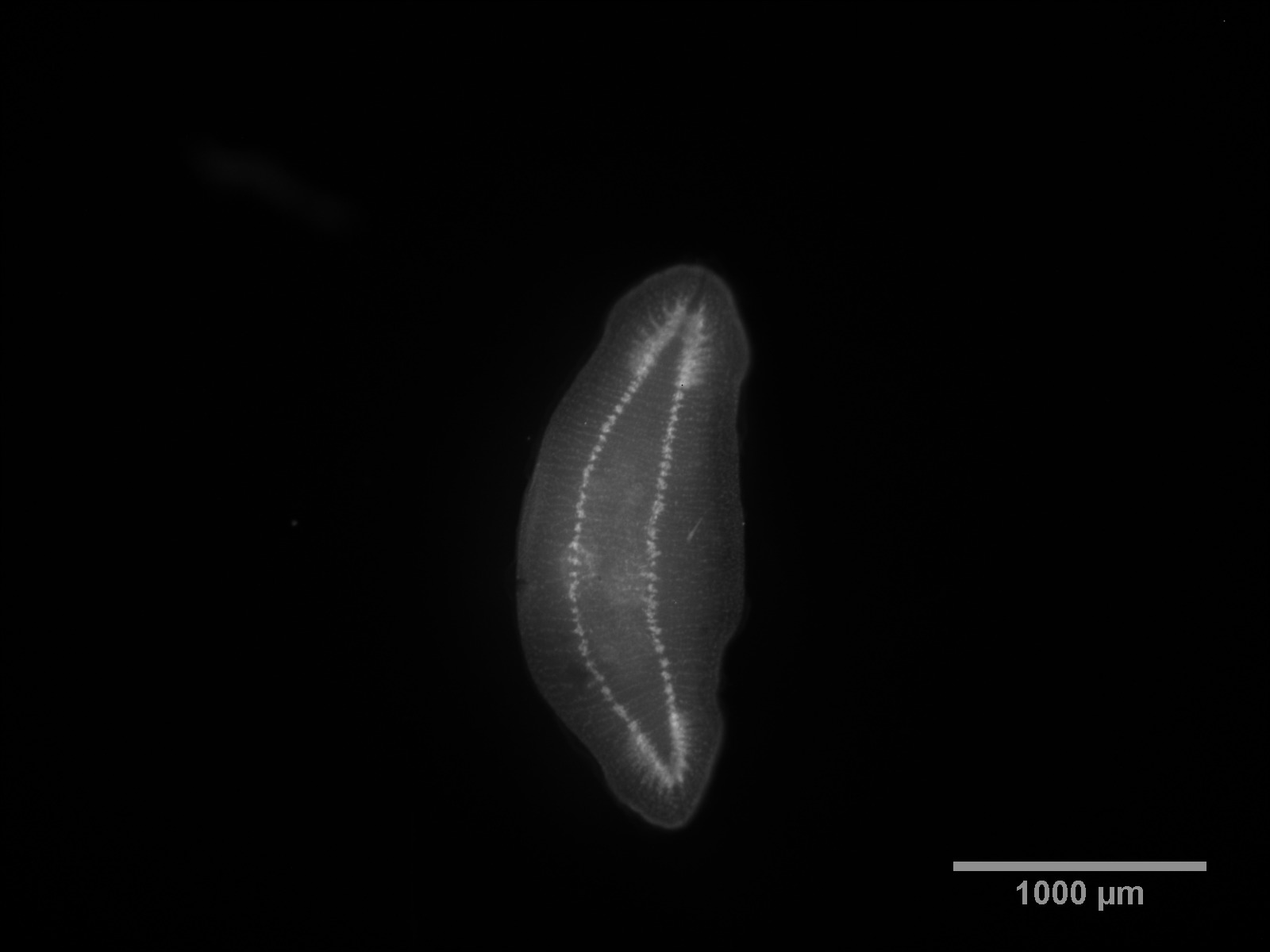

Supplement: S2 Dataset — This dataset contains raw-images of synapsin stains of uncut one- and two- headed worms, synapsin stains and brightfield images of the upwards and inverted L-cut scenarios, and synapsin stains and brightfield images showing the effects of the dynein inhibitor Ciliobrevin D on planaria regeneration. A Word document contained in the zip folder provides detailed description of the different cases. (ZIP) [file pcbi.1006904.s017.zip › DatasetS9i/2synapsinstain2H/Sample 11.jpg]

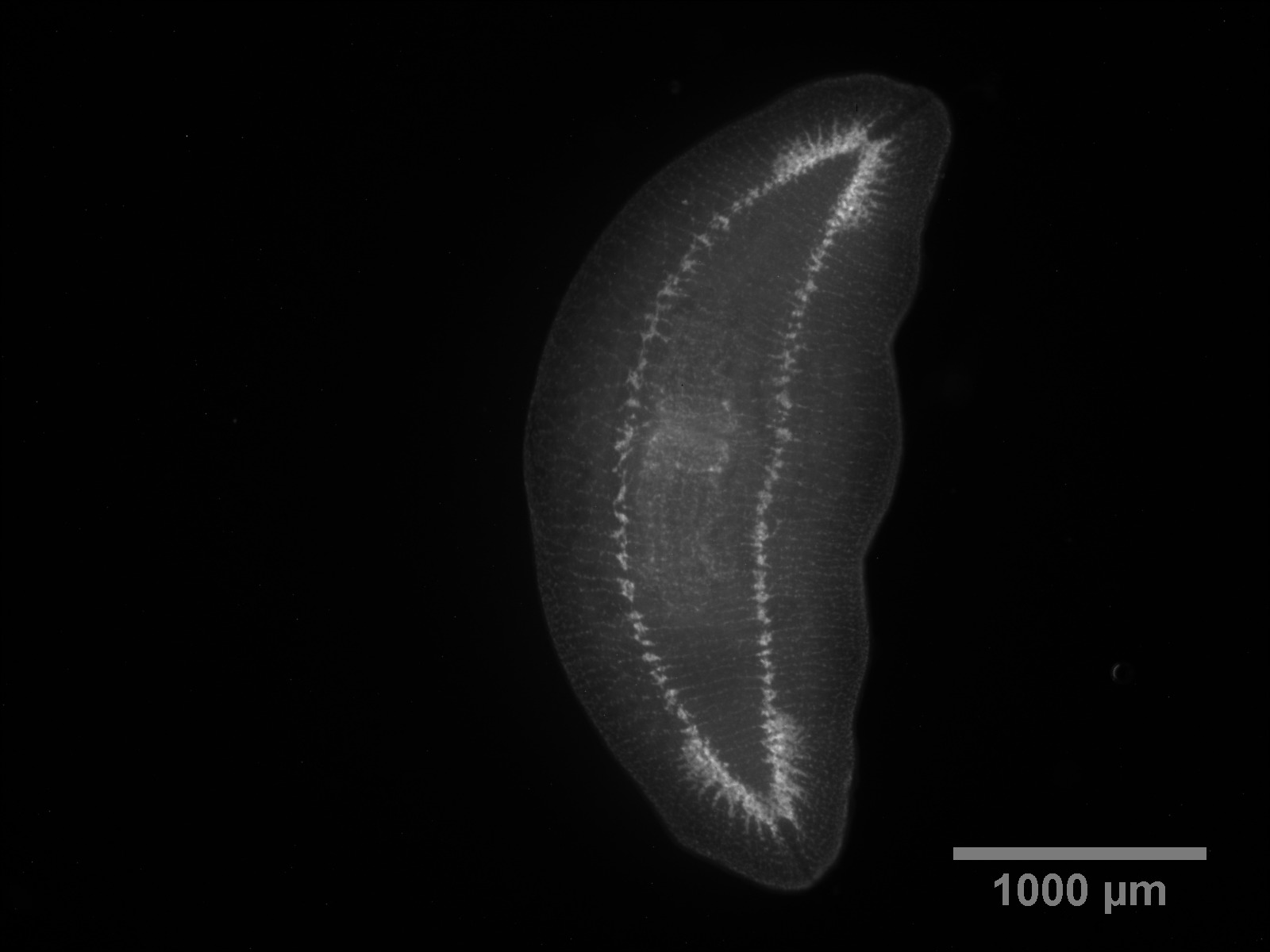

Supplement: S2 Dataset — This dataset contains raw-images of synapsin stains of uncut one- and two- headed worms, synapsin stains and brightfield images of the upwards and inverted L-cut scenarios, and synapsin stains and brightfield images showing the effects of the dynein inhibitor Ciliobrevin D on planaria regeneration. A Word document contained in the zip folder provides detailed description of the different cases. (ZIP) [file pcbi.1006904.s017.zip › DatasetS9i/2synapsinstain2H/Sample 12.jpg]

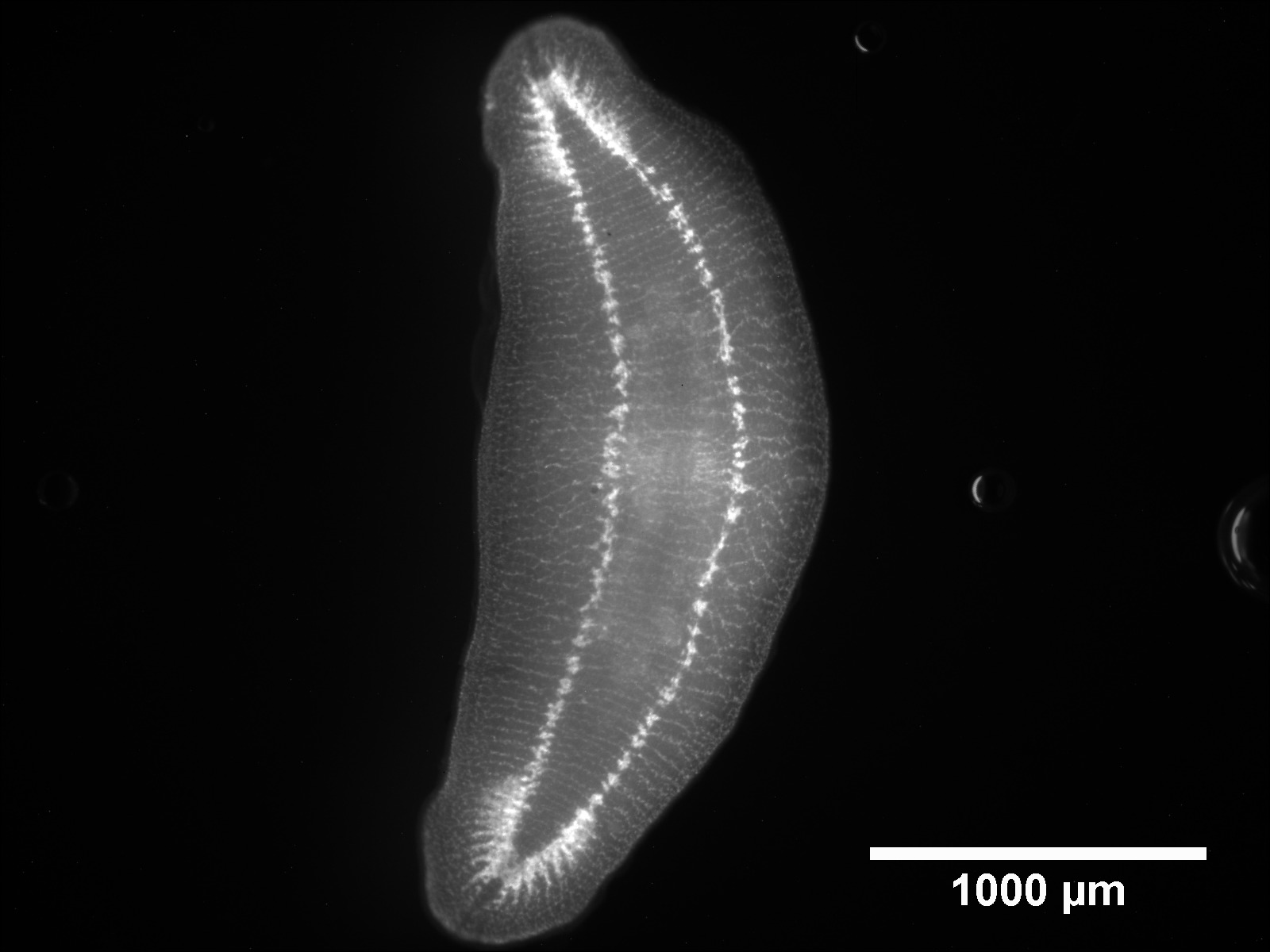

Supplement: S2 Dataset — This dataset contains raw-images of synapsin stains of uncut one- and two- headed worms, synapsin stains and brightfield images of the upwards and inverted L-cut scenarios, and synapsin stains and brightfield images showing the effects of the dynein inhibitor Ciliobrevin D on planaria regeneration. A Word document contained in the zip folder provides detailed description of the different cases. (ZIP) [file pcbi.1006904.s017.zip › DatasetS9i/2synapsinstain2H/Sample 13.jpg]

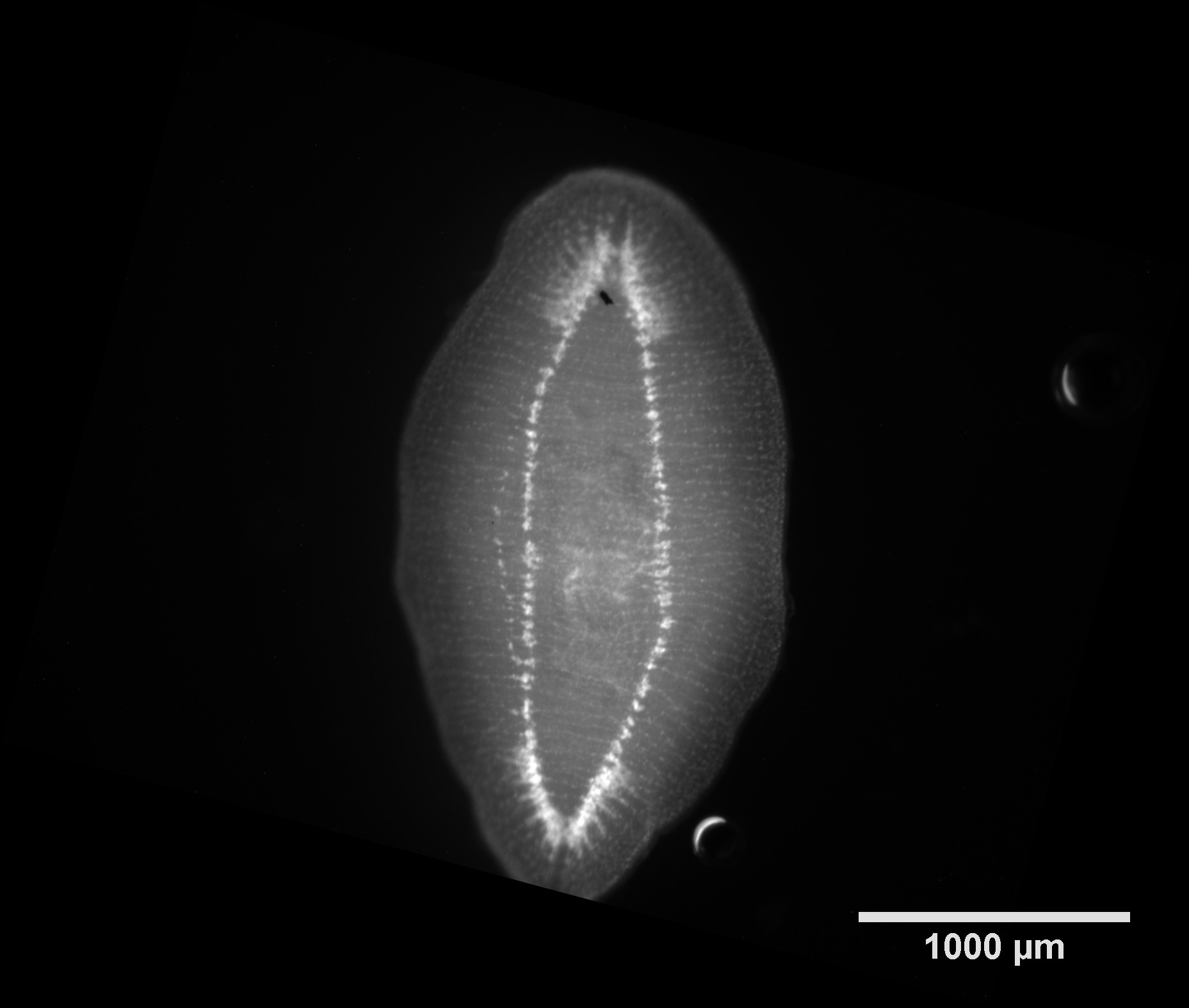

Supplement: S2 Dataset — This dataset contains raw-images of synapsin stains of uncut one- and two- headed worms, synapsin stains and brightfield images of the upwards and inverted L-cut scenarios, and synapsin stains and brightfield images showing the effects of the dynein inhibitor Ciliobrevin D on planaria regeneration. A Word document contained in the zip folder provides detailed description of the different cases. (ZIP) [file pcbi.1006904.s017.zip › DatasetS9i/2synapsinstain2H/Sample 14.jpg]

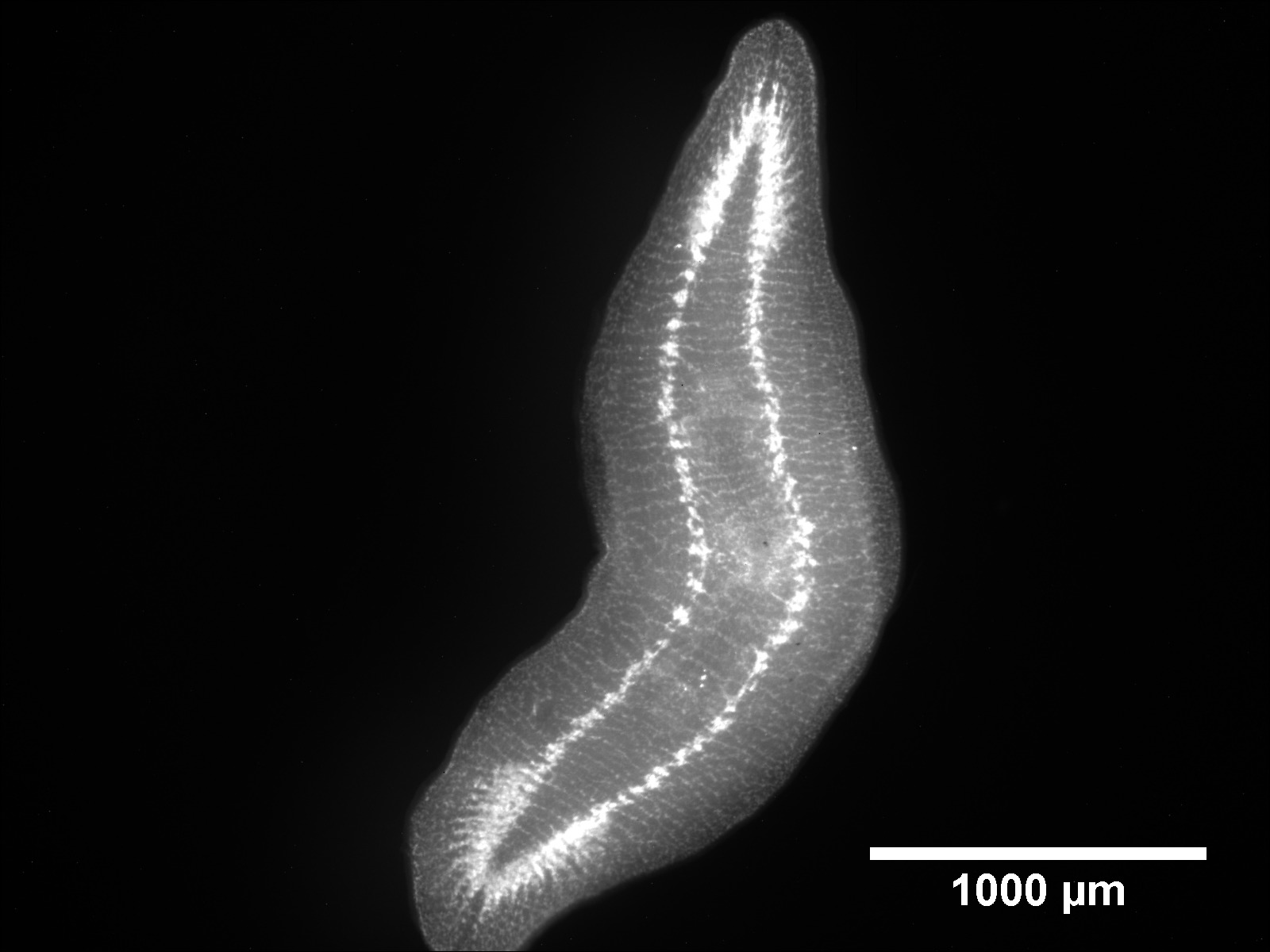

Supplement: S2 Dataset — This dataset contains raw-images of synapsin stains of uncut one- and two- headed worms, synapsin stains and brightfield images of the upwards and inverted L-cut scenarios, and synapsin stains and brightfield images showing the effects of the dynein inhibitor Ciliobrevin D on planaria regeneration. A Word document contained in the zip folder provides detailed description of the different cases. (ZIP) [file pcbi.1006904.s017.zip › DatasetS9i/2synapsinstain2H/Sample 15.jpg]

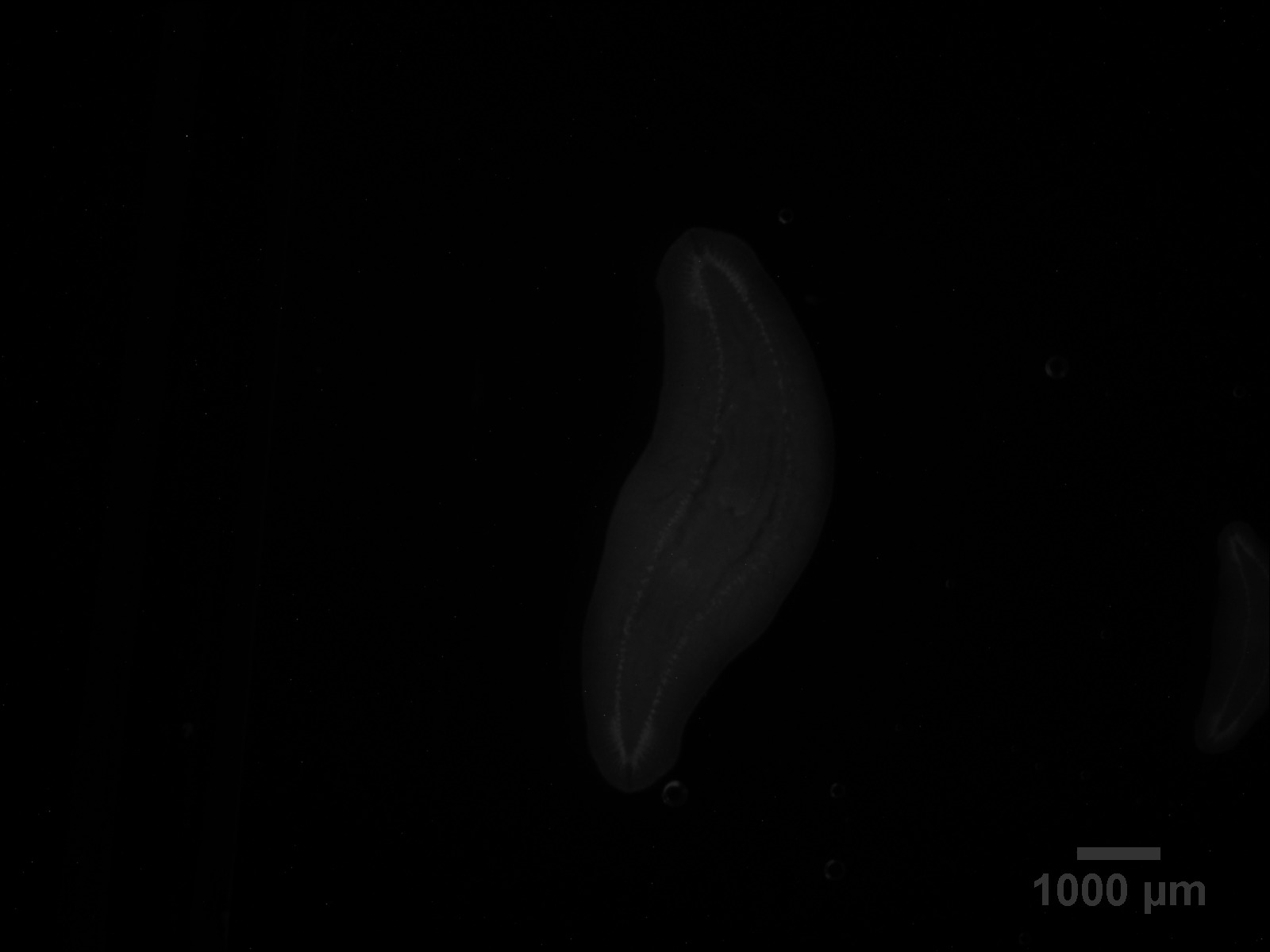

Supplement: S2 Dataset — This dataset contains raw-images of synapsin stains of uncut one- and two- headed worms, synapsin stains and brightfield images of the upwards and inverted L-cut scenarios, and synapsin stains and brightfield images showing the effects of the dynein inhibitor Ciliobrevin D on planaria regeneration. A Word document contained in the zip folder provides detailed description of the different cases. (ZIP) [file pcbi.1006904.s017.zip › DatasetS9i/2synapsinstain2H/Sample 1.jpg]

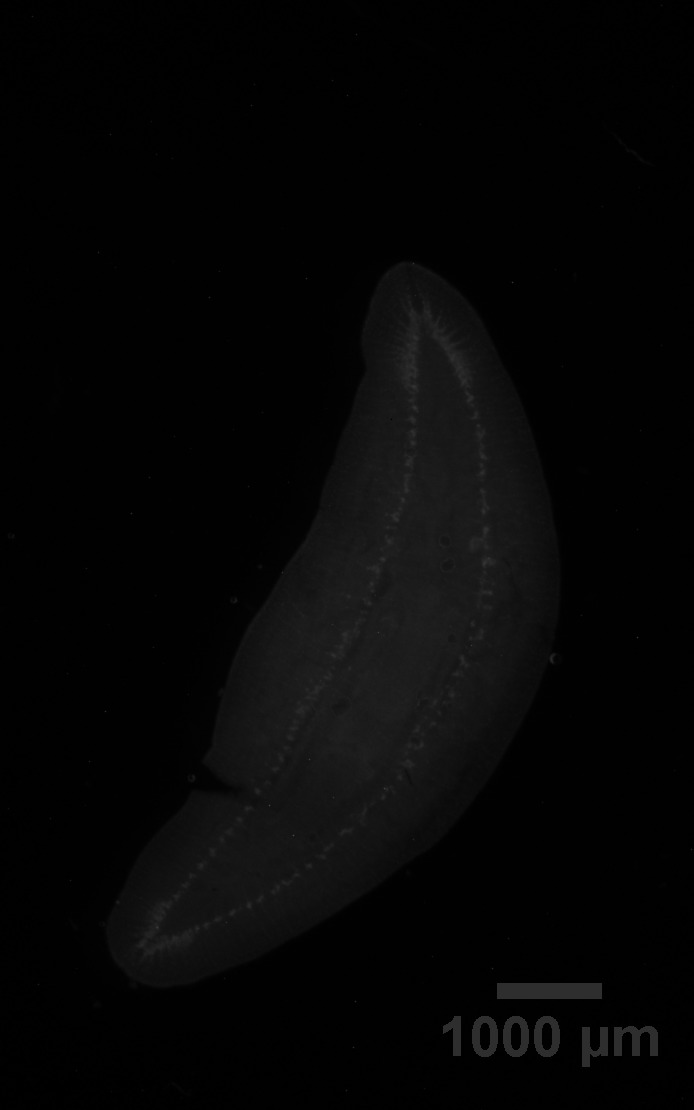

Supplement: S2 Dataset — This dataset contains raw-images of synapsin stains of uncut one- and two- headed worms, synapsin stains and brightfield images of the upwards and inverted L-cut scenarios, and synapsin stains and brightfield images showing the effects of the dynein inhibitor Ciliobrevin D on planaria regeneration. A Word document contained in the zip folder provides detailed description of the different cases. (ZIP) [file pcbi.1006904.s017.zip › DatasetS9i/2synapsinstain2H/Sample 2.jpg]

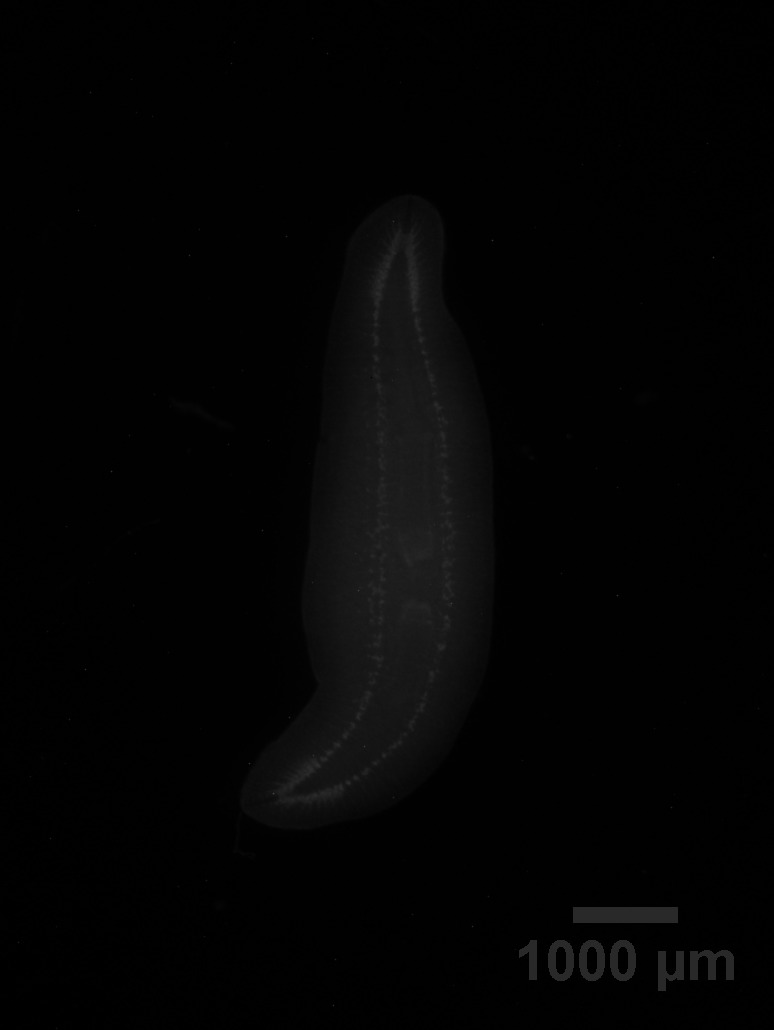

Supplement: S2 Dataset — This dataset contains raw-images of synapsin stains of uncut one- and two- headed worms, synapsin stains and brightfield images of the upwards and inverted L-cut scenarios, and synapsin stains and brightfield images showing the effects of the dynein inhibitor Ciliobrevin D on planaria regeneration. A Word document contained in the zip folder provides detailed description of the different cases. (ZIP) [file pcbi.1006904.s017.zip › DatasetS9i/2synapsinstain2H/Sample 3.jpg]

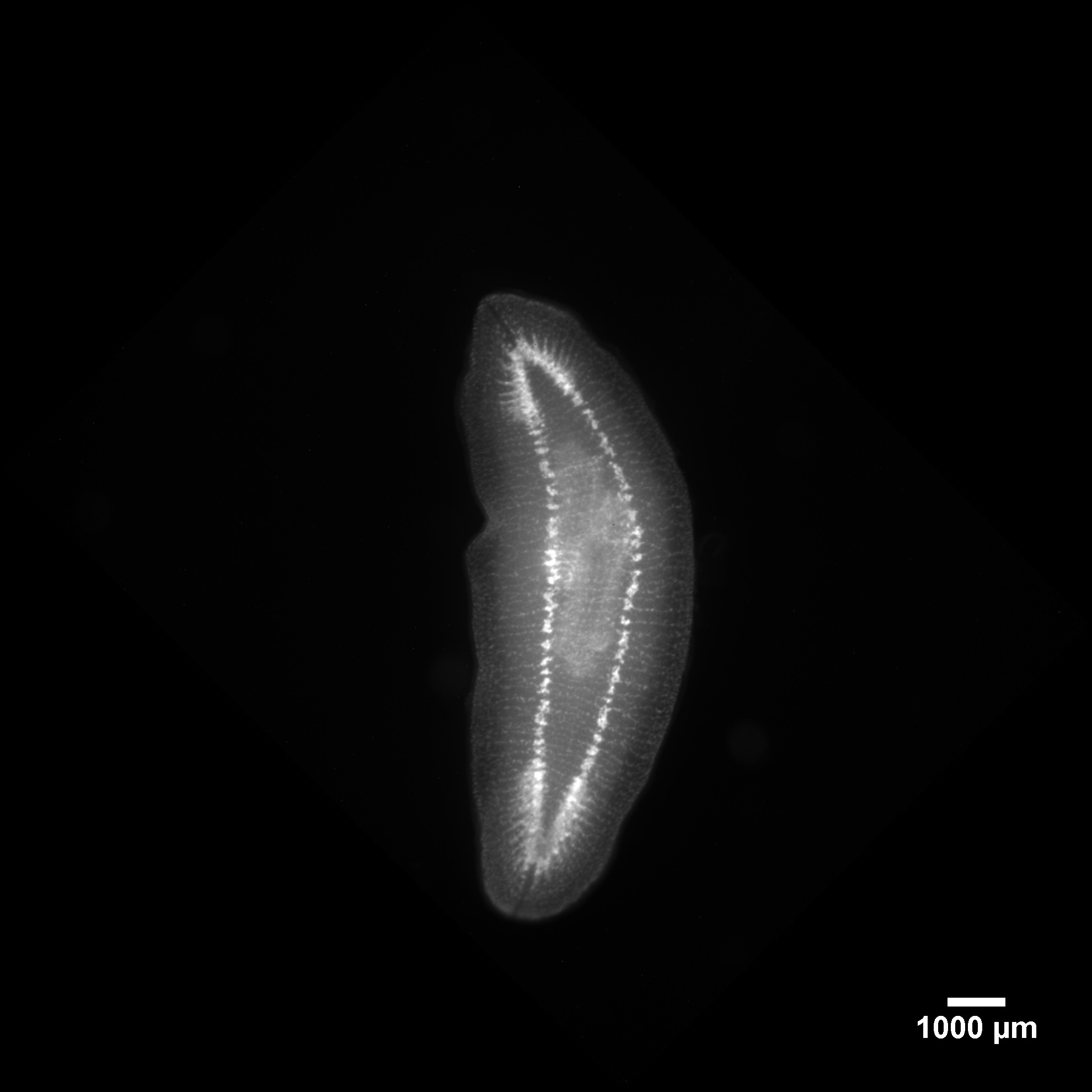

Supplement: S2 Dataset — This dataset contains raw-images of synapsin stains of uncut one- and two- headed worms, synapsin stains and brightfield images of the upwards and inverted L-cut scenarios, and synapsin stains and brightfield images showing the effects of the dynein inhibitor Ciliobrevin D on planaria regeneration. A Word document contained in the zip folder provides detailed description of the different cases. (ZIP) [file pcbi.1006904.s017.zip › DatasetS9i/2synapsinstain2H/Sample 4.jpg]

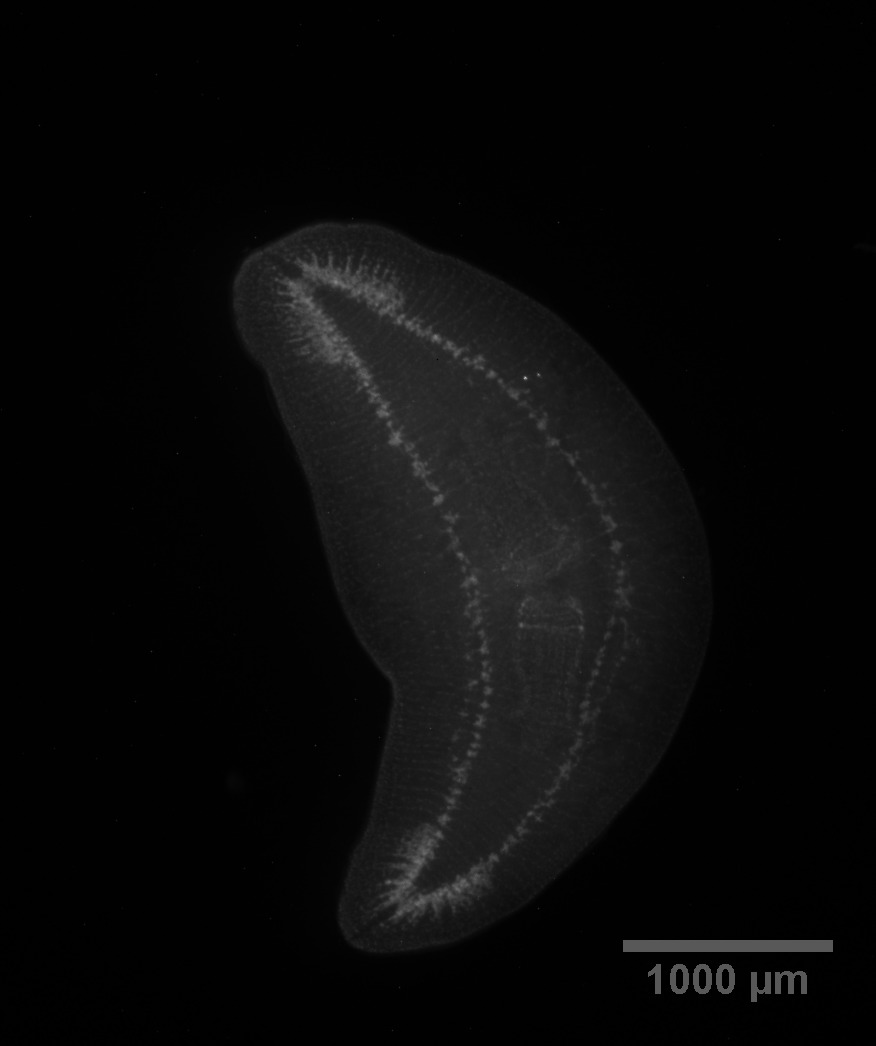

Supplement: S2 Dataset — This dataset contains raw-images of synapsin stains of uncut one- and two- headed worms, synapsin stains and brightfield images of the upwards and inverted L-cut scenarios, and synapsin stains and brightfield images showing the effects of the dynein inhibitor Ciliobrevin D on planaria regeneration. A Word document contained in the zip folder provides detailed description of the different cases. (ZIP) [file pcbi.1006904.s017.zip › DatasetS9i/2synapsinstain2H/Sample 5.jpg]

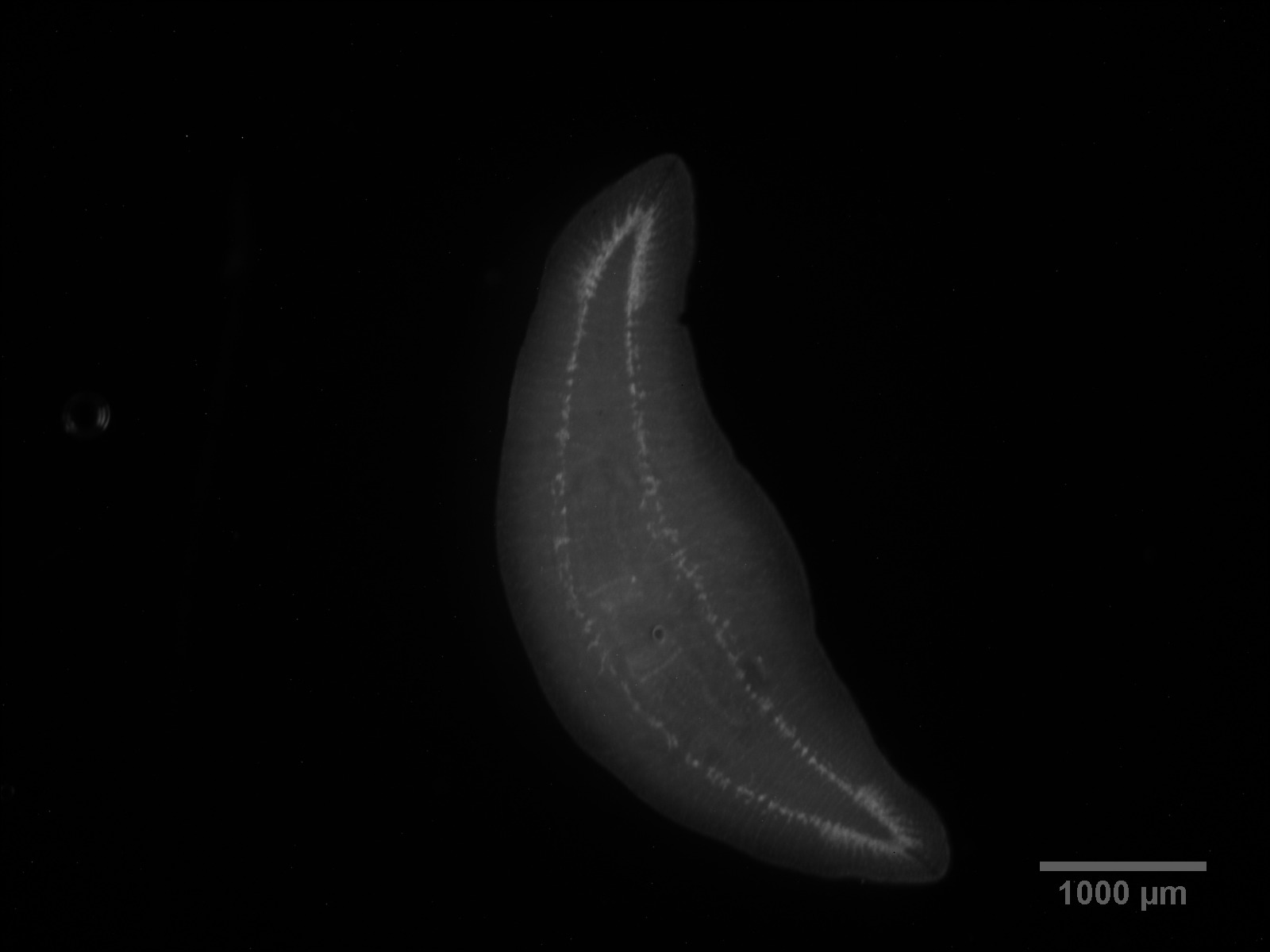

Supplement: S2 Dataset — This dataset contains raw-images of synapsin stains of uncut one- and two- headed worms, synapsin stains and brightfield images of the upwards and inverted L-cut scenarios, and synapsin stains and brightfield images showing the effects of the dynein inhibitor Ciliobrevin D on planaria regeneration. A Word document contained in the zip folder provides detailed description of the different cases. (ZIP) [file pcbi.1006904.s017.zip › DatasetS9i/2synapsinstain2H/Sample 6.jpg]

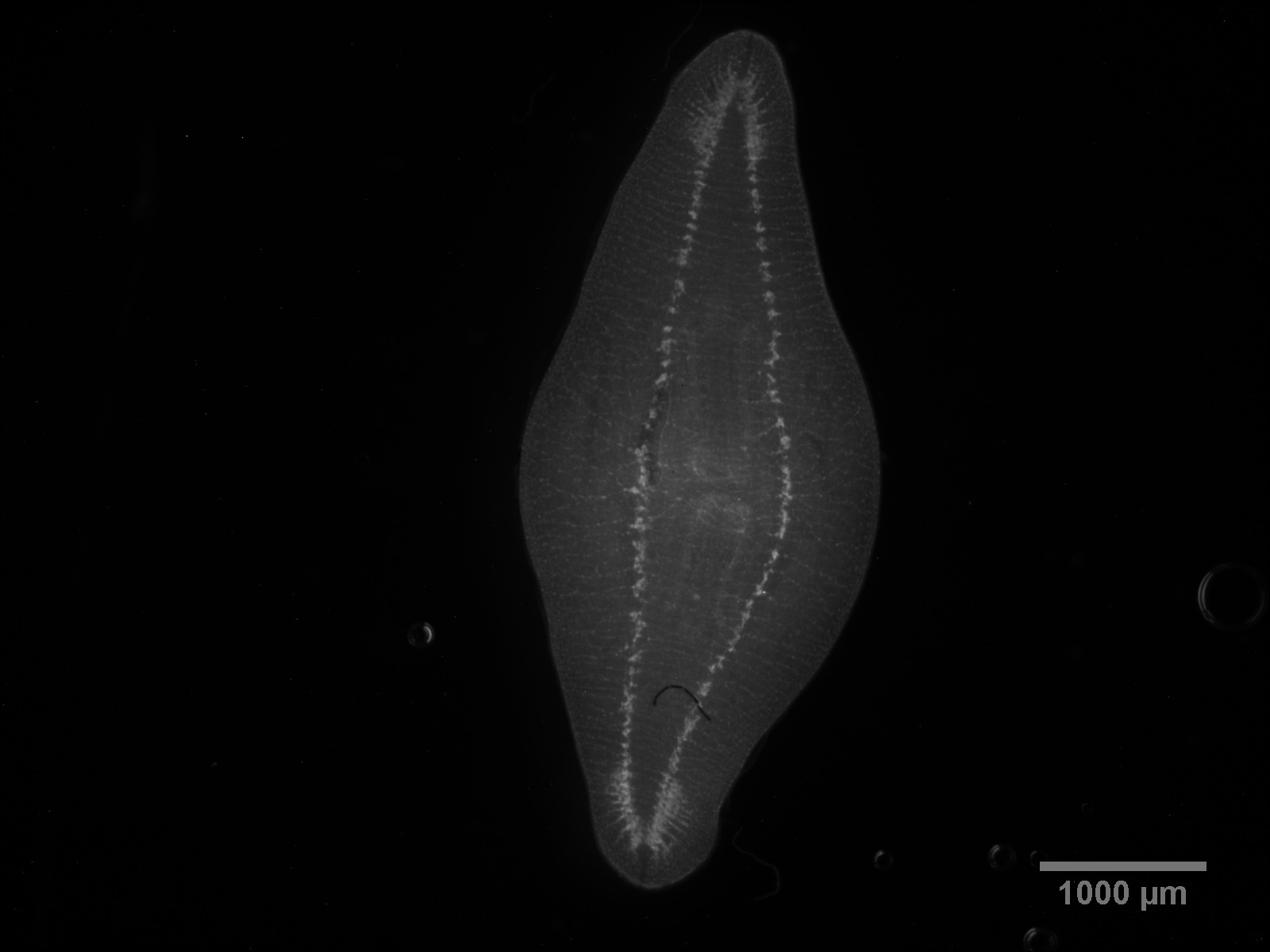

Supplement: S2 Dataset — This dataset contains raw-images of synapsin stains of uncut one- and two- headed worms, synapsin stains and brightfield images of the upwards and inverted L-cut scenarios, and synapsin stains and brightfield images showing the effects of the dynein inhibitor Ciliobrevin D on planaria regeneration. A Word document contained in the zip folder provides detailed description of the different cases. (ZIP) [file pcbi.1006904.s017.zip › DatasetS9i/2synapsinstain2H/Sample 7.jpg]

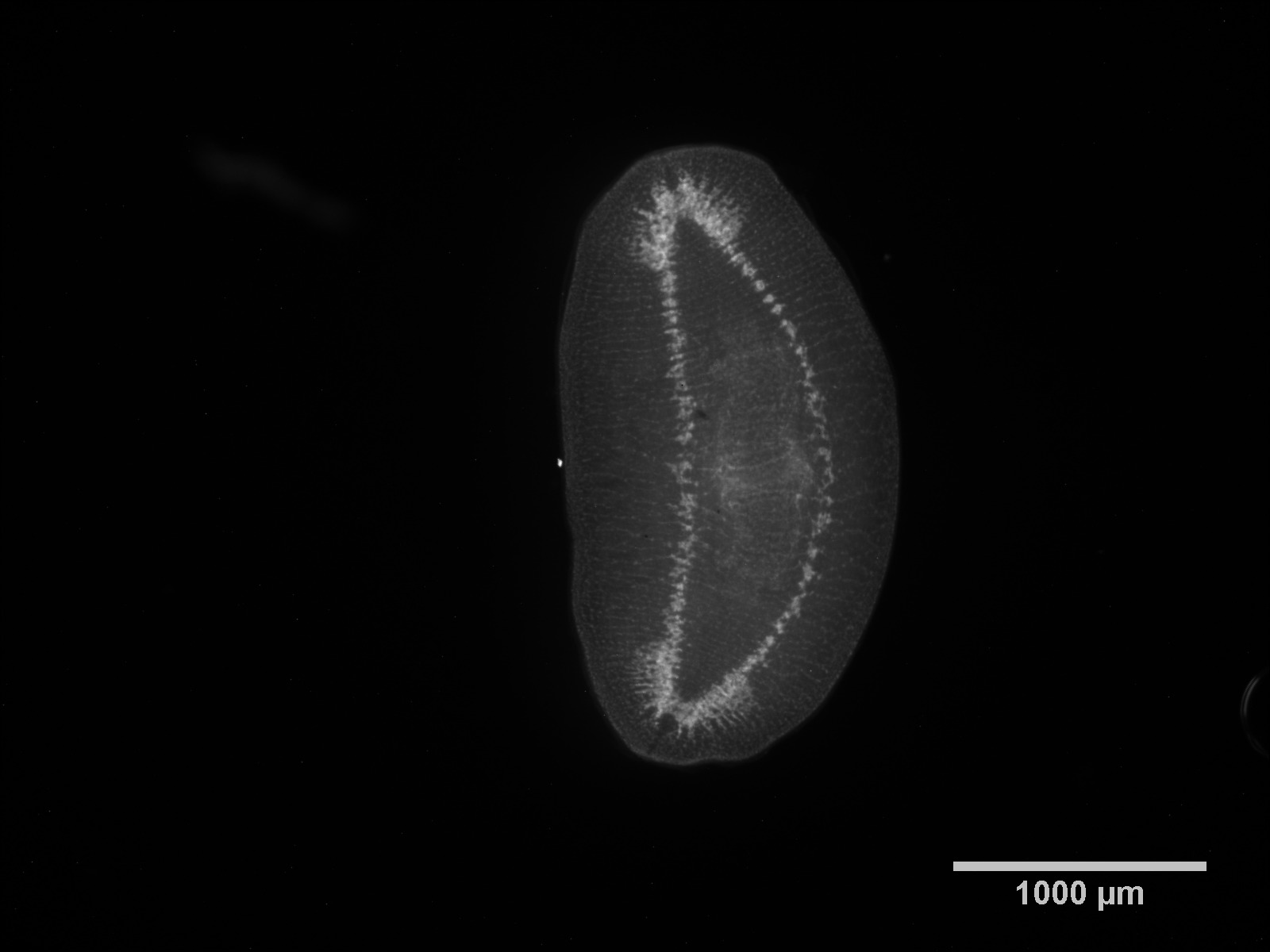

Supplement: S2 Dataset — This dataset contains raw-images of synapsin stains of uncut one- and two- headed worms, synapsin stains and brightfield images of the upwards and inverted L-cut scenarios, and synapsin stains and brightfield images showing the effects of the dynein inhibitor Ciliobrevin D on planaria regeneration. A Word document contained in the zip folder provides detailed description of the different cases. (ZIP) [file pcbi.1006904.s017.zip › DatasetS9i/2synapsinstain2H/Sample 8.jpg]

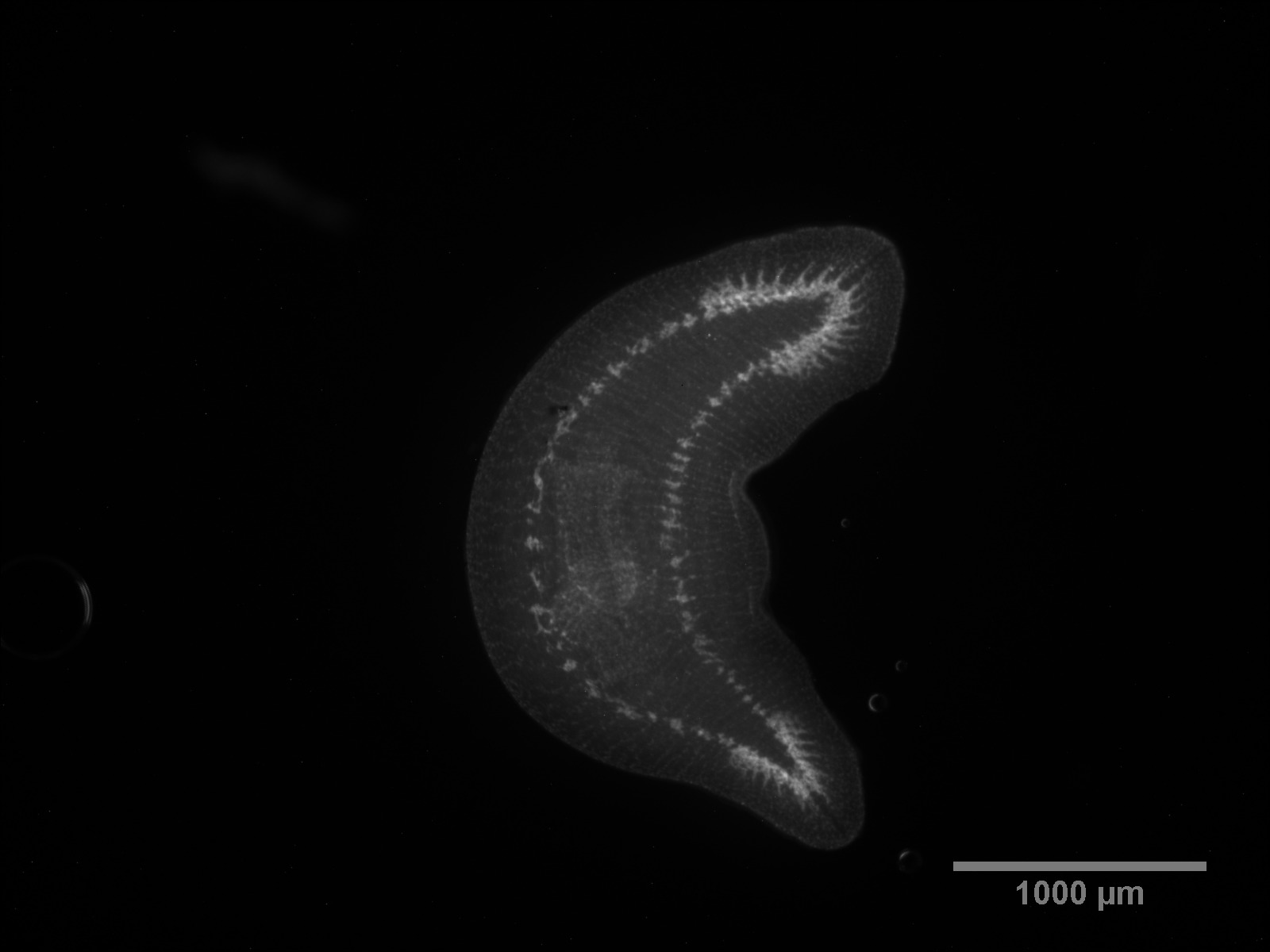

Supplement: S2 Dataset — This dataset contains raw-images of synapsin stains of uncut one- and two- headed worms, synapsin stains and brightfield images of the upwards and inverted L-cut scenarios, and synapsin stains and brightfield images showing the effects of the dynein inhibitor Ciliobrevin D on planaria regeneration. A Word document contained in the zip folder provides detailed description of the different cases. (ZIP) [file pcbi.1006904.s017.zip › DatasetS9i/2synapsinstain2H/Sample 9.jpg]

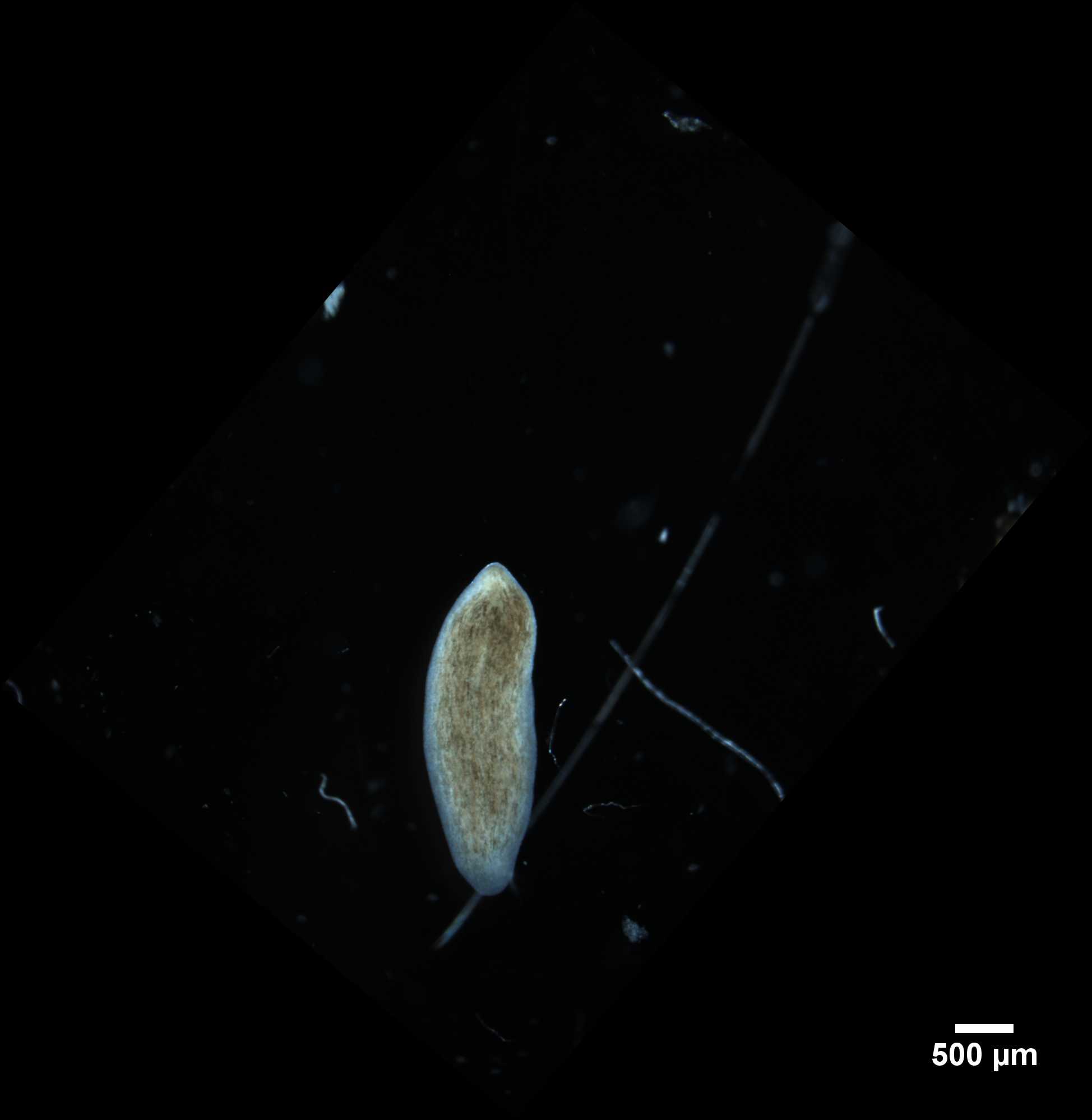

Supplement: S2 Dataset — This dataset contains raw-images of synapsin stains of uncut one- and two- headed worms, synapsin stains and brightfield images of the upwards and inverted L-cut scenarios, and synapsin stains and brightfield images showing the effects of the dynein inhibitor Ciliobrevin D on planaria regeneration. A Word document contained in the zip folder provides detailed description of the different cases. (ZIP) [file pcbi.1006904.s017.zip › DatasetS9i/Dynein_inhibition/Brightfield/Sample 10.jpg]

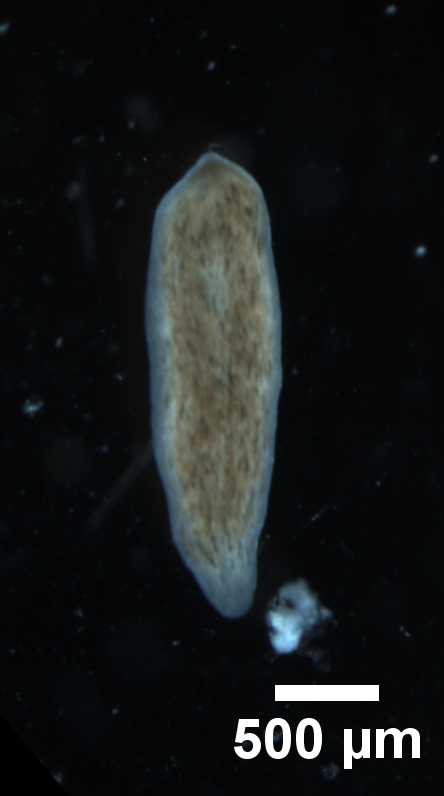

Supplement: S2 Dataset — This dataset contains raw-images of synapsin stains of uncut one- and two- headed worms, synapsin stains and brightfield images of the upwards and inverted L-cut scenarios, and synapsin stains and brightfield images showing the effects of the dynein inhibitor Ciliobrevin D on planaria regeneration. A Word document contained in the zip folder provides detailed description of the different cases. (ZIP) [file pcbi.1006904.s017.zip › DatasetS9i/Dynein_inhibition/Brightfield/Sample 1.jpg]

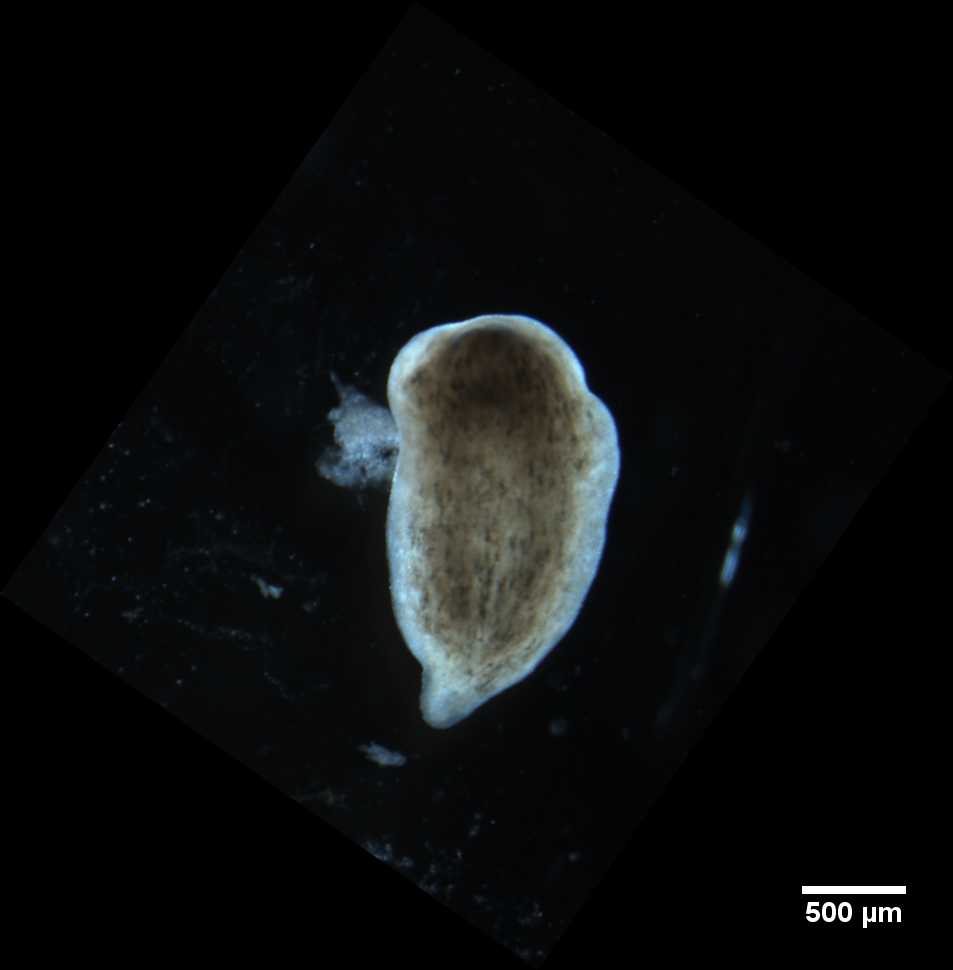

Supplement: S2 Dataset — This dataset contains raw-images of synapsin stains of uncut one- and two- headed worms, synapsin stains and brightfield images of the upwards and inverted L-cut scenarios, and synapsin stains and brightfield images showing the effects of the dynein inhibitor Ciliobrevin D on planaria regeneration. A Word document contained in the zip folder provides detailed description of the different cases. (ZIP) [file pcbi.1006904.s017.zip › DatasetS9i/Dynein_inhibition/Brightfield/Sample 2.jpg]

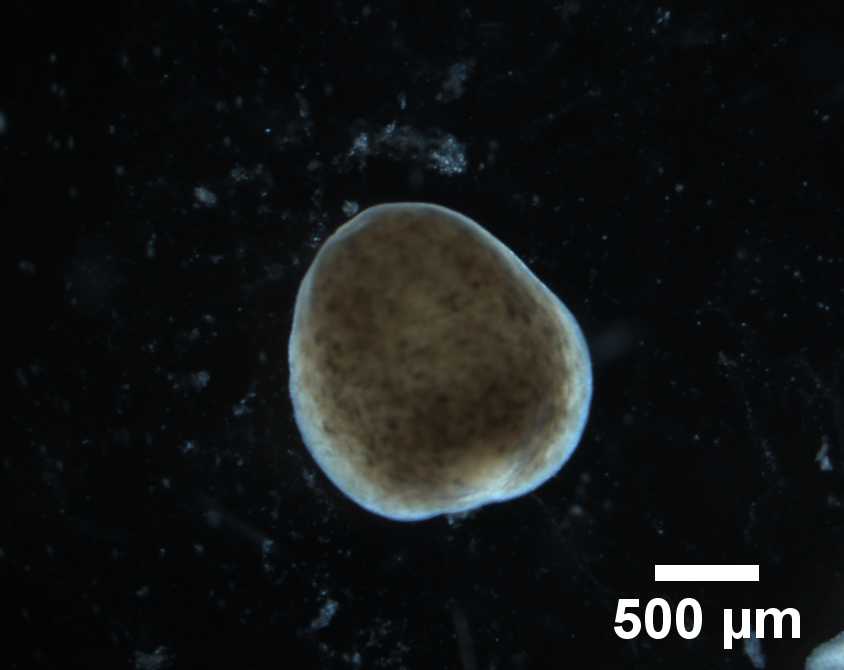

Supplement: S2 Dataset — This dataset contains raw-images of synapsin stains of uncut one- and two- headed worms, synapsin stains and brightfield images of the upwards and inverted L-cut scenarios, and synapsin stains and brightfield images showing the effects of the dynein inhibitor Ciliobrevin D on planaria regeneration. A Word document contained in the zip folder provides detailed description of the different cases. (ZIP) [file pcbi.1006904.s017.zip › DatasetS9i/Dynein_inhibition/Brightfield/Sample 3.jpg]

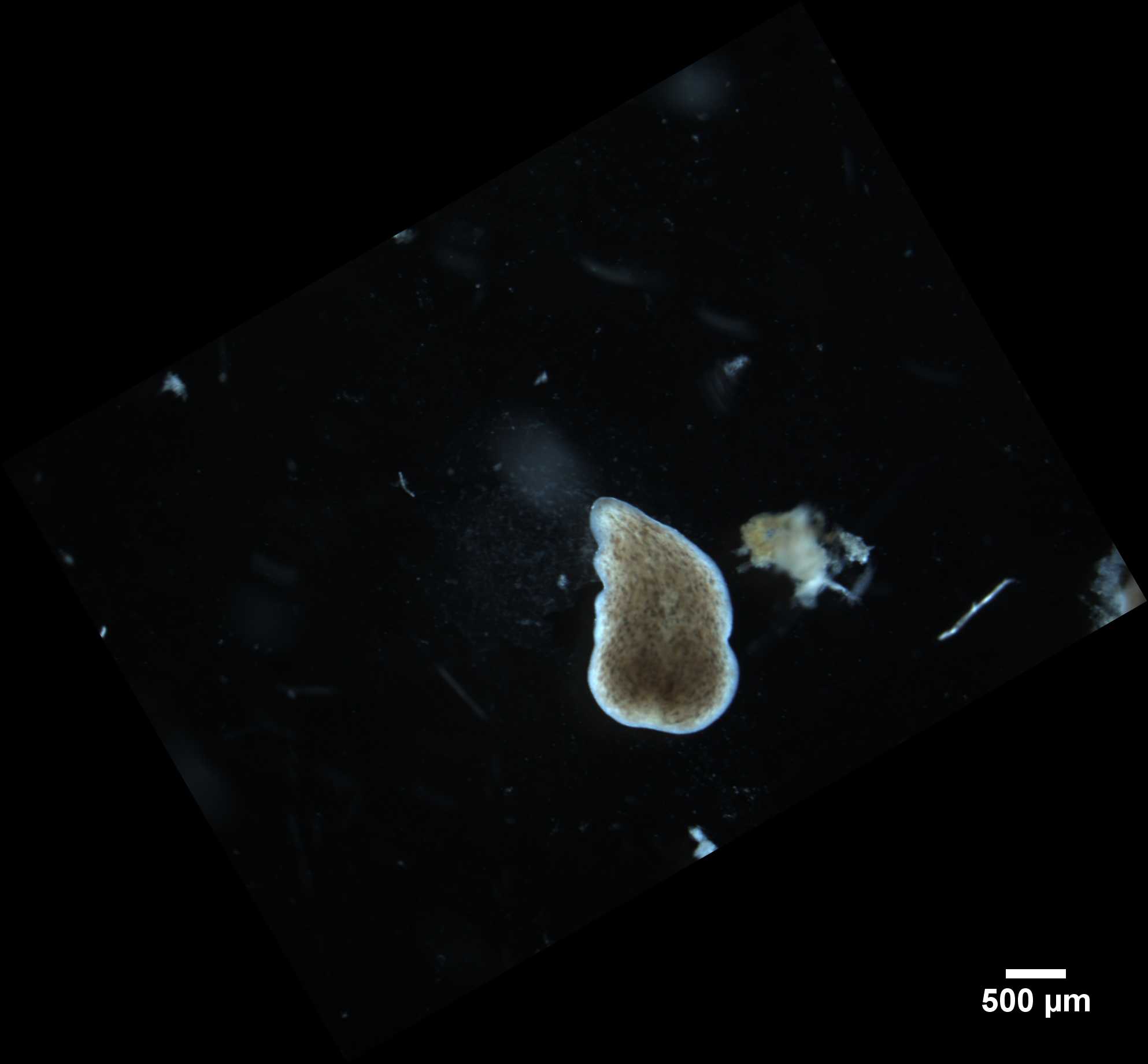

Supplement: S2 Dataset — This dataset contains raw-images of synapsin stains of uncut one- and two- headed worms, synapsin stains and brightfield images of the upwards and inverted L-cut scenarios, and synapsin stains and brightfield images showing the effects of the dynein inhibitor Ciliobrevin D on planaria regeneration. A Word document contained in the zip folder provides detailed description of the different cases. (ZIP) [file pcbi.1006904.s017.zip › DatasetS9i/Dynein_inhibition/Brightfield/Sample 4.jpg]

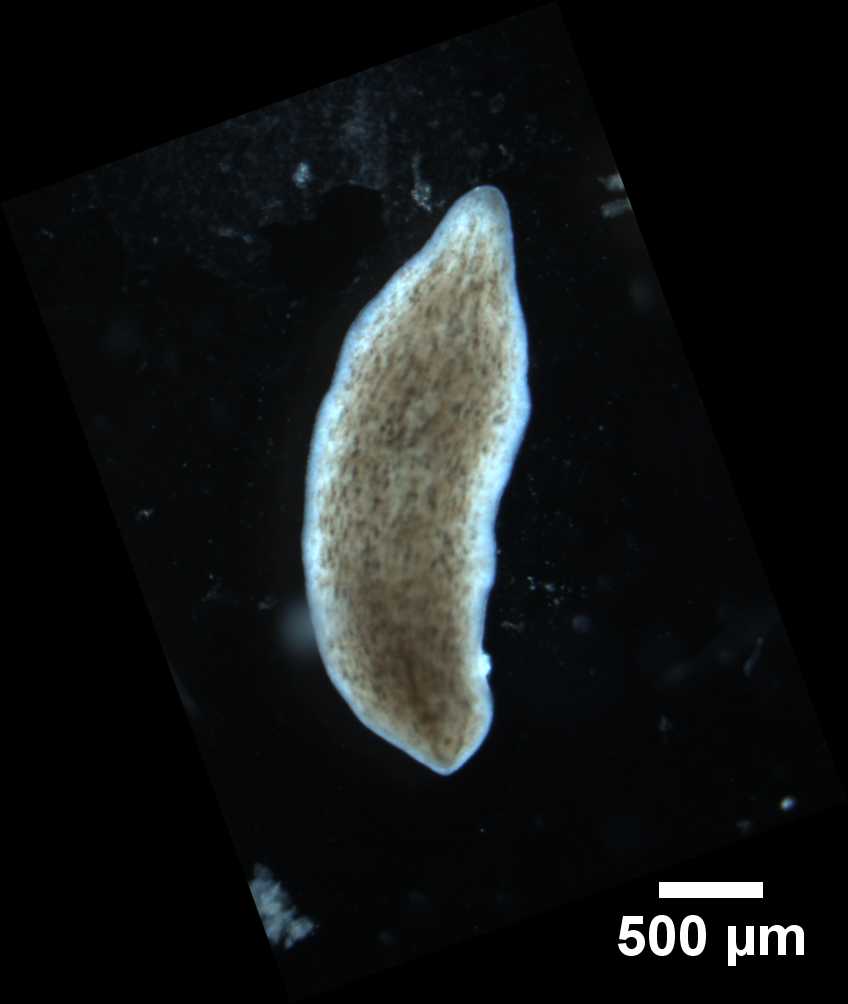

Supplement: S2 Dataset — This dataset contains raw-images of synapsin stains of uncut one- and two- headed worms, synapsin stains and brightfield images of the upwards and inverted L-cut scenarios, and synapsin stains and brightfield images showing the effects of the dynein inhibitor Ciliobrevin D on planaria regeneration. A Word document contained in the zip folder provides detailed description of the different cases. (ZIP) [file pcbi.1006904.s017.zip › DatasetS9i/Dynein_inhibition/Brightfield/Sample 5.jpg]

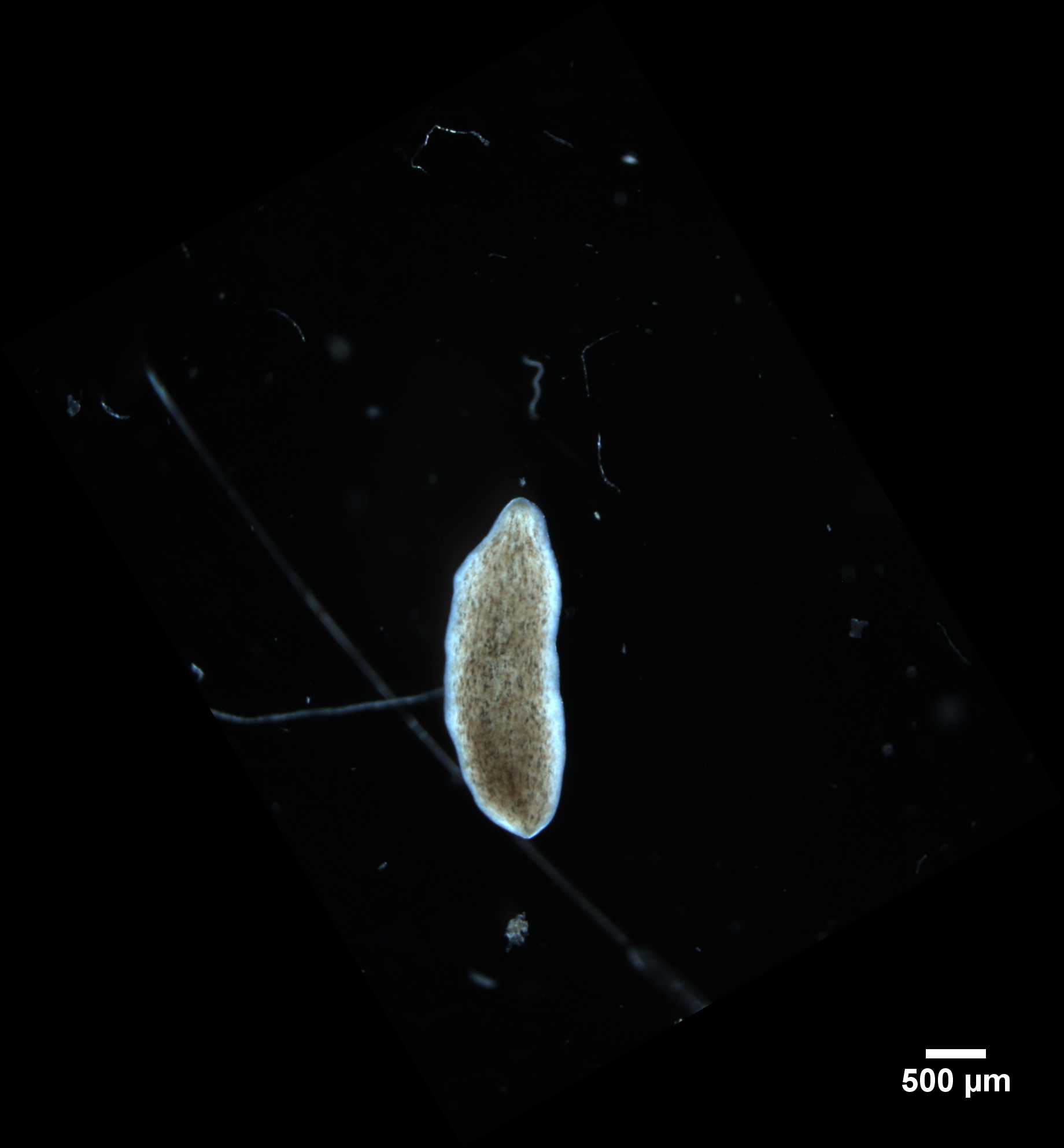

Supplement: S2 Dataset — This dataset contains raw-images of synapsin stains of uncut one- and two- headed worms, synapsin stains and brightfield images of the upwards and inverted L-cut scenarios, and synapsin stains and brightfield images showing the effects of the dynein inhibitor Ciliobrevin D on planaria regeneration. A Word document contained in the zip folder provides detailed description of the different cases. (ZIP) [file pcbi.1006904.s017.zip › DatasetS9i/Dynein_inhibition/Brightfield/Sample 6.jpg]

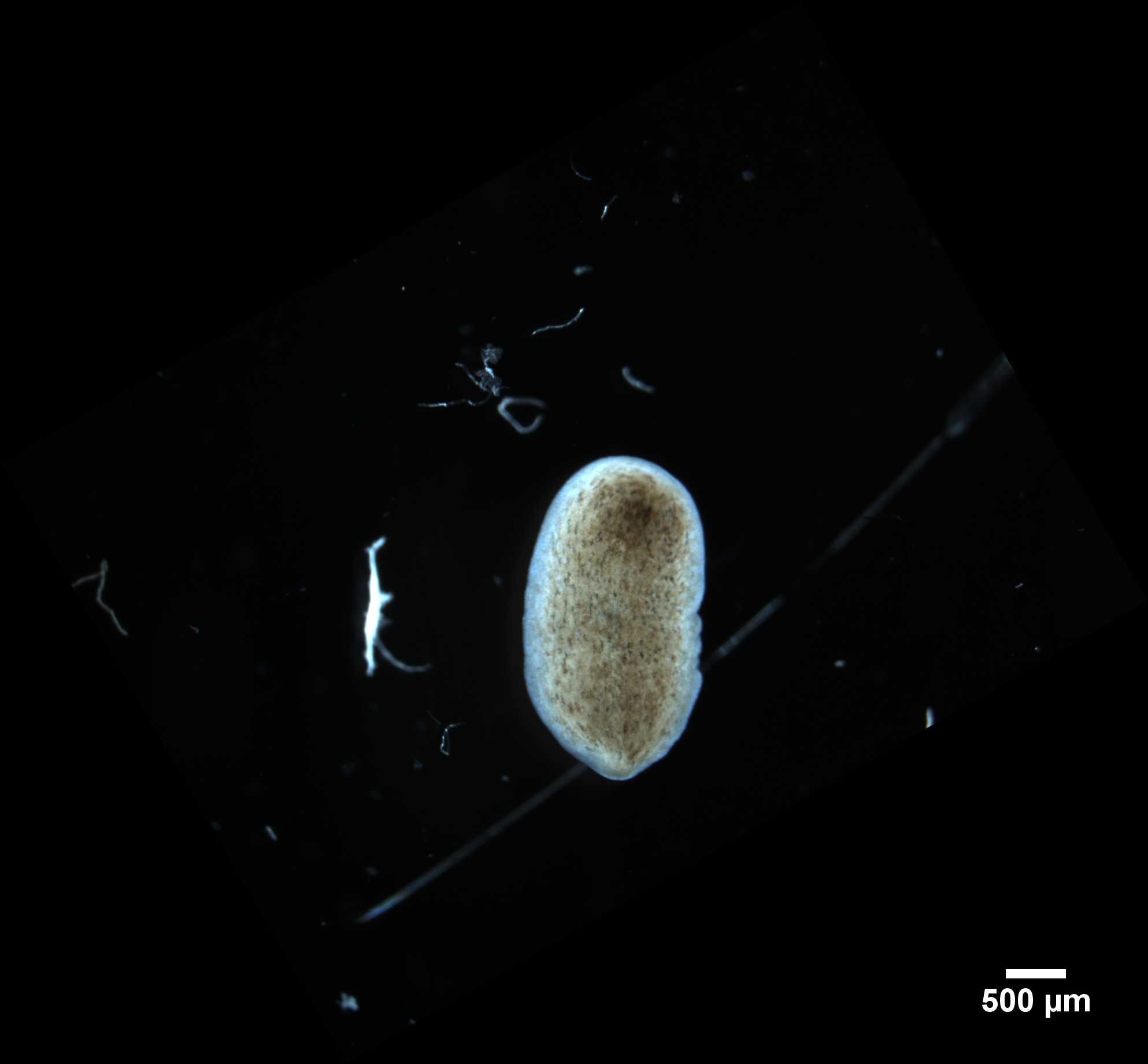

Supplement: S2 Dataset — This dataset contains raw-images of synapsin stains of uncut one- and two- headed worms, synapsin stains and brightfield images of the upwards and inverted L-cut scenarios, and synapsin stains and brightfield images showing the effects of the dynein inhibitor Ciliobrevin D on planaria regeneration. A Word document contained in the zip folder provides detailed description of the different cases. (ZIP) [file pcbi.1006904.s017.zip › DatasetS9i/Dynein_inhibition/Brightfield/Sample 7.jpg]

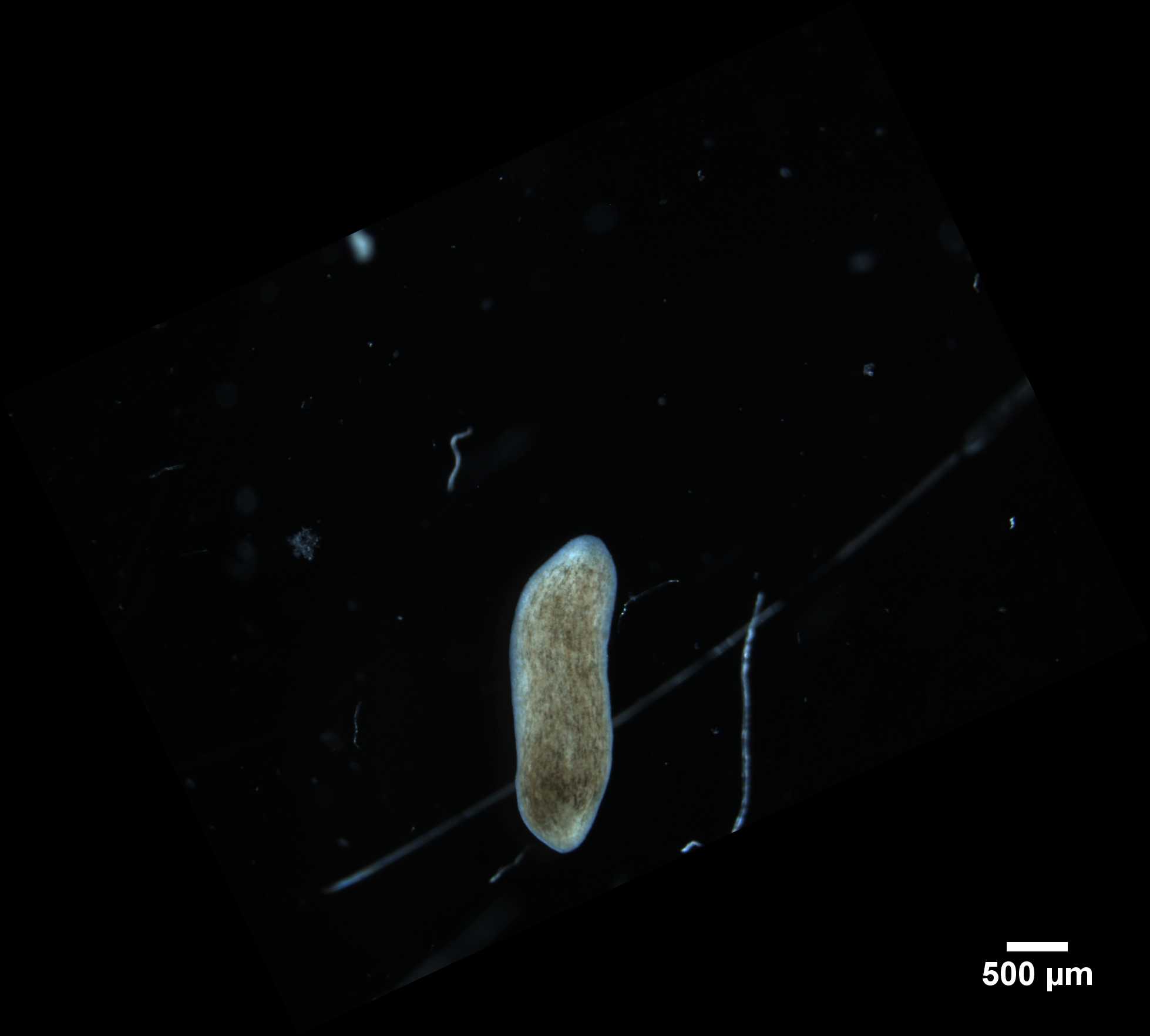

Supplement: S2 Dataset — This dataset contains raw-images of synapsin stains of uncut one- and two- headed worms, synapsin stains and brightfield images of the upwards and inverted L-cut scenarios, and synapsin stains and brightfield images showing the effects of the dynein inhibitor Ciliobrevin D on planaria regeneration. A Word document contained in the zip folder provides detailed description of the different cases. (ZIP) [file pcbi.1006904.s017.zip › DatasetS9i/Dynein_inhibition/Brightfield/Sample 8.jpg]

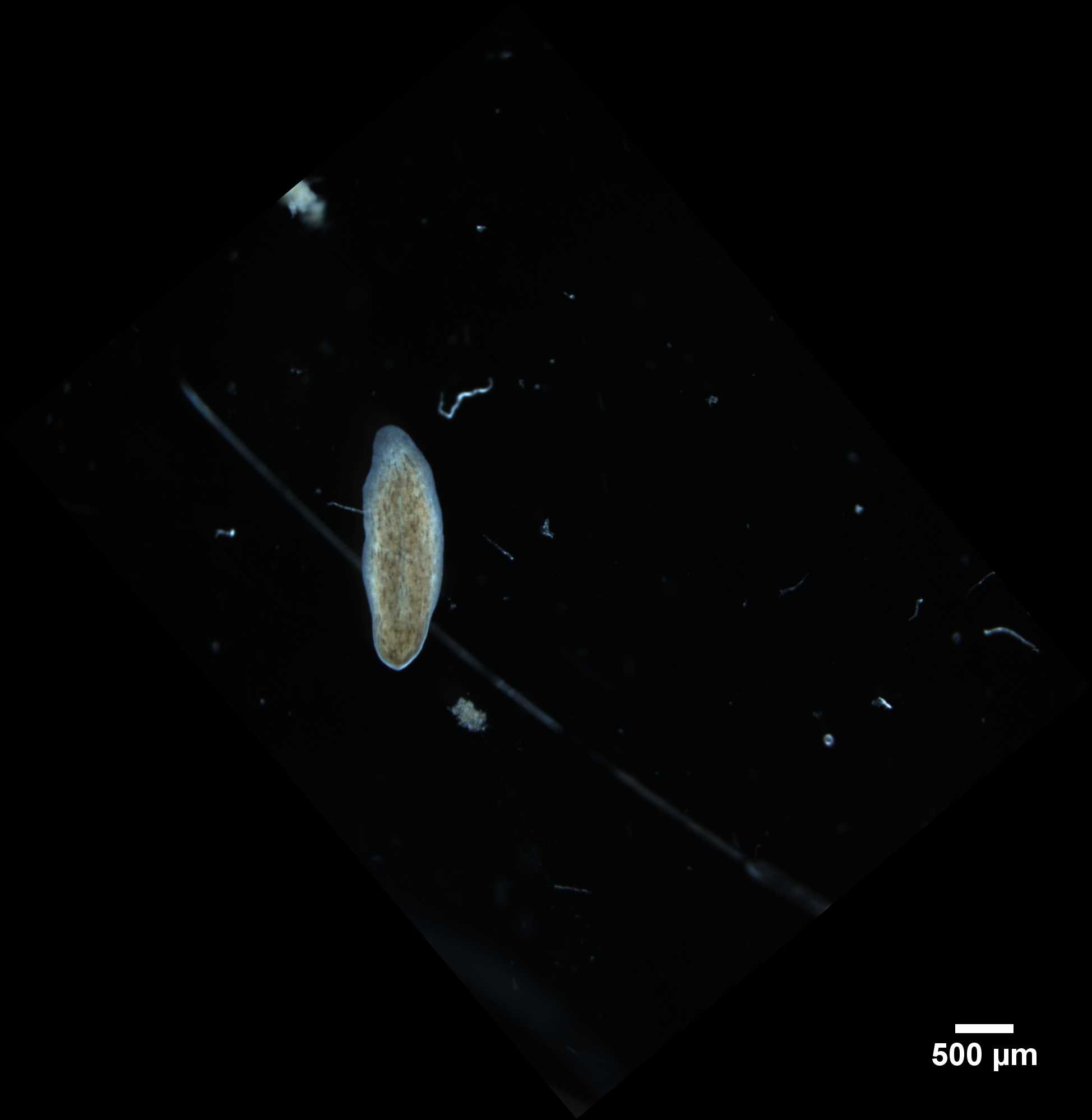

Supplement: S2 Dataset — This dataset contains raw-images of synapsin stains of uncut one- and two- headed worms, synapsin stains and brightfield images of the upwards and inverted L-cut scenarios, and synapsin stains and brightfield images showing the effects of the dynein inhibitor Ciliobrevin D on planaria regeneration. A Word document contained in the zip folder provides detailed description of the different cases. (ZIP) [file pcbi.1006904.s017.zip › DatasetS9i/Dynein_inhibition/Brightfield/Sample 9.jpg]

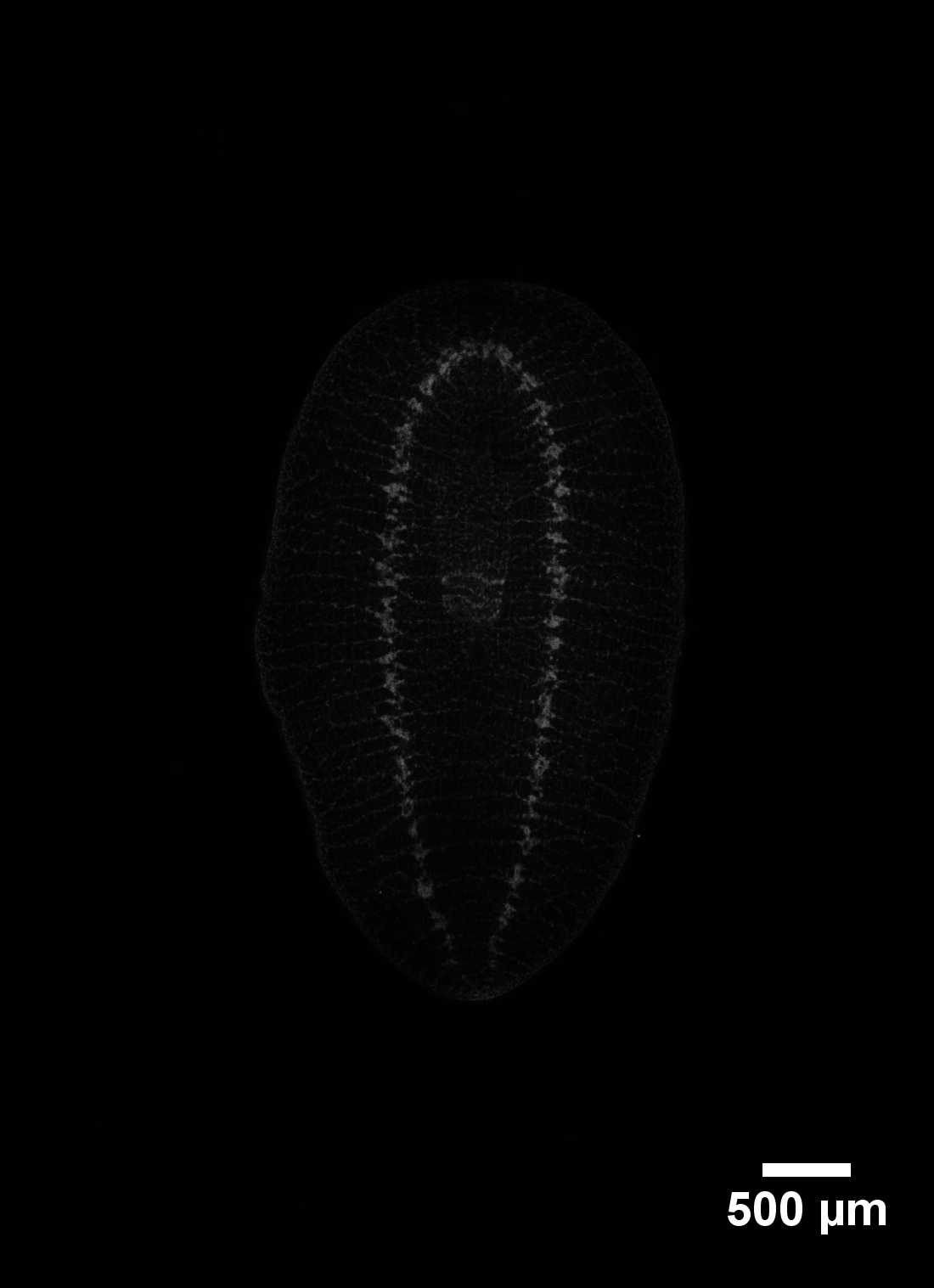

Supplement: S2 Dataset — This dataset contains raw-images of synapsin stains of uncut one- and two- headed worms, synapsin stains and brightfield images of the upwards and inverted L-cut scenarios, and synapsin stains and brightfield images showing the effects of the dynein inhibitor Ciliobrevin D on planaria regeneration. A Word document contained in the zip folder provides detailed description of the different cases. (ZIP) [file pcbi.1006904.s017.zip › DatasetS9i/Dynein_inhibition/synapsin stain/Sample 1-0.jpg]

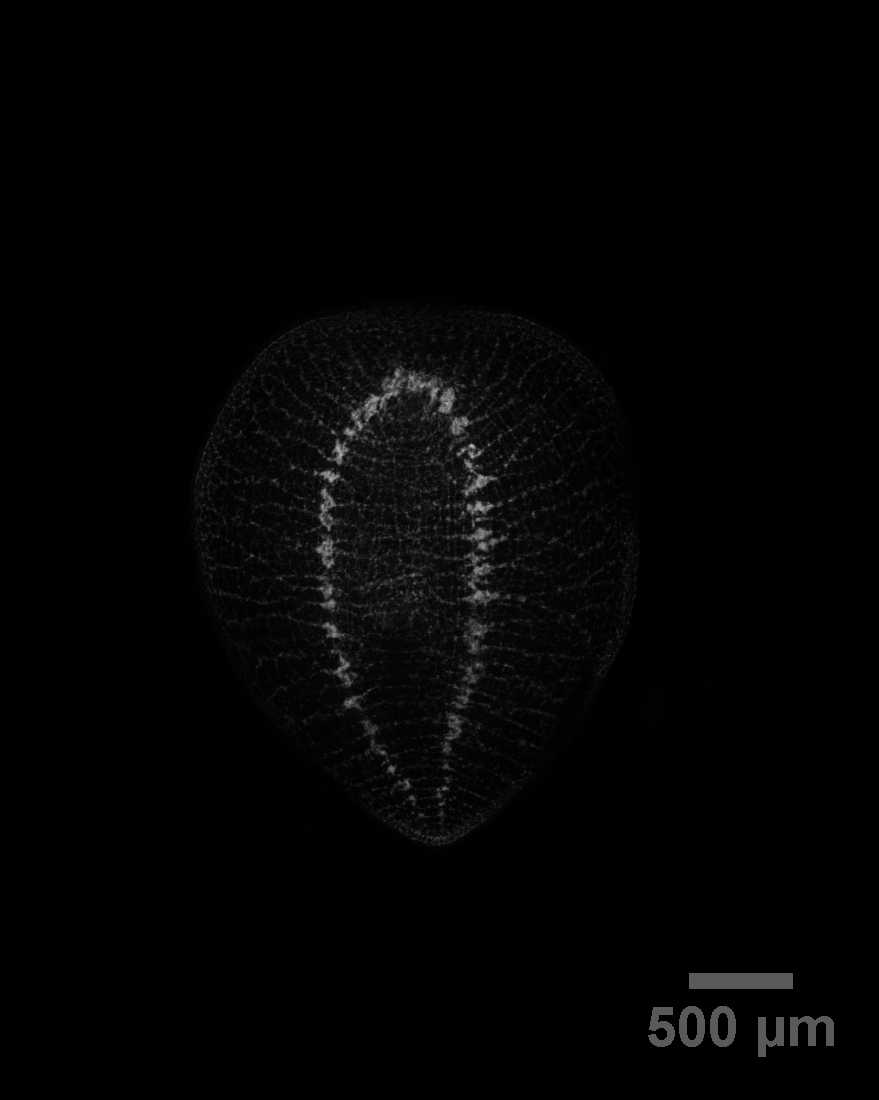

Supplement: S2 Dataset — This dataset contains raw-images of synapsin stains of uncut one- and two- headed worms, synapsin stains and brightfield images of the upwards and inverted L-cut scenarios, and synapsin stains and brightfield images showing the effects of the dynein inhibitor Ciliobrevin D on planaria regeneration. A Word document contained in the zip folder provides detailed description of the different cases. (ZIP) [file pcbi.1006904.s017.zip › DatasetS9i/Dynein_inhibition/synapsin stain/Sample 2-0.jpg]

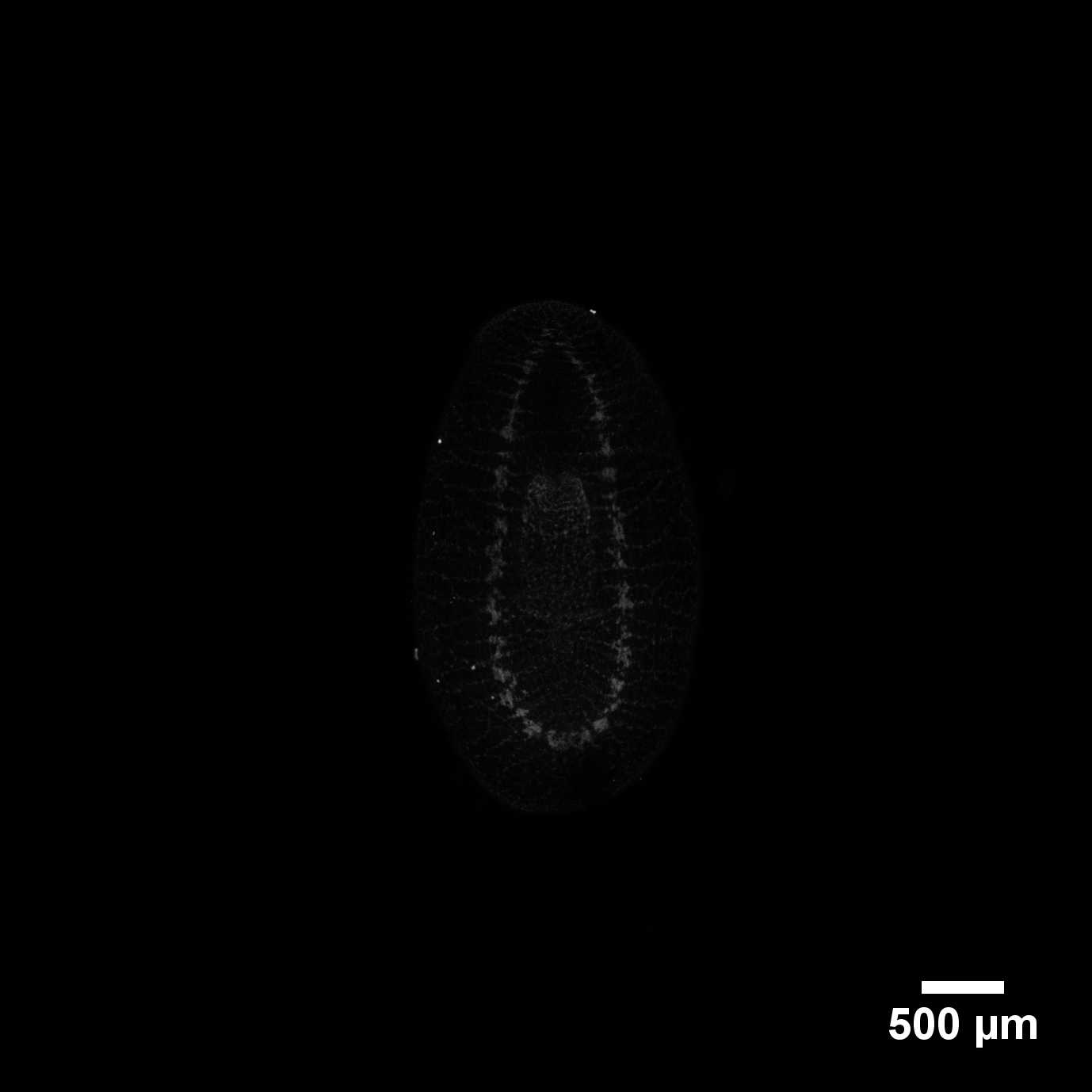

Supplement: S2 Dataset — This dataset contains raw-images of synapsin stains of uncut one- and two- headed worms, synapsin stains and brightfield images of the upwards and inverted L-cut scenarios, and synapsin stains and brightfield images showing the effects of the dynein inhibitor Ciliobrevin D on planaria regeneration. A Word document contained in the zip folder provides detailed description of the different cases. (ZIP) [file pcbi.1006904.s017.zip › DatasetS9i/Dynein_inhibition/synapsin stain/Sample 3-0.jpg]

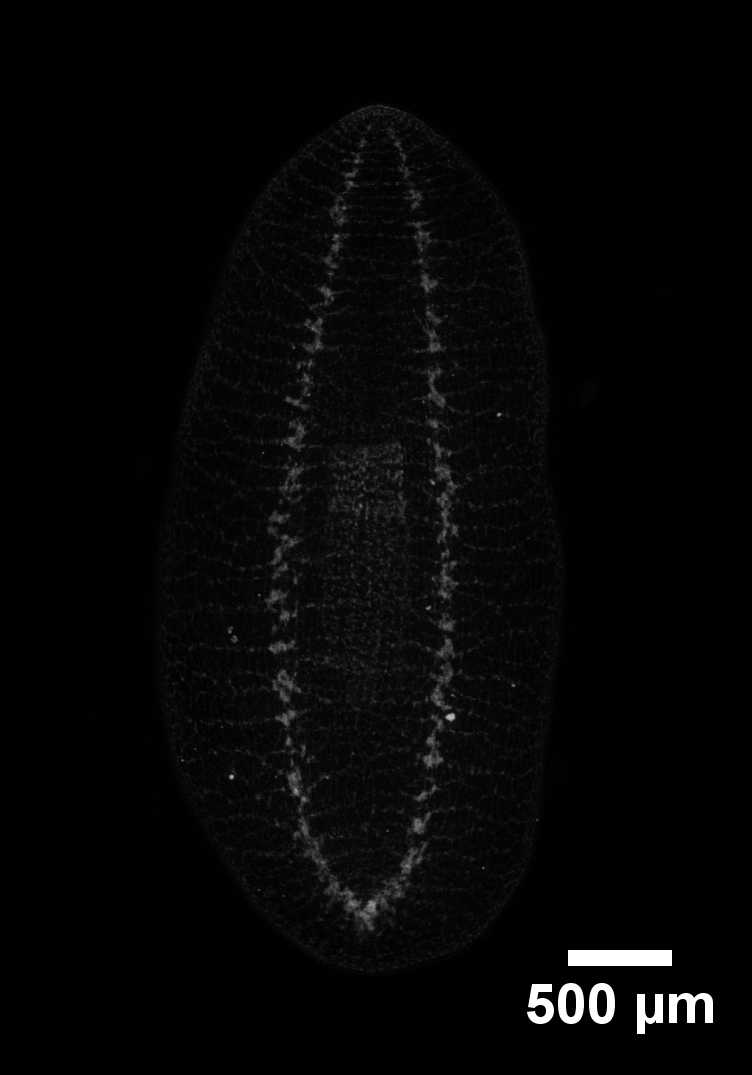

Supplement: S2 Dataset — This dataset contains raw-images of synapsin stains of uncut one- and two- headed worms, synapsin stains and brightfield images of the upwards and inverted L-cut scenarios, and synapsin stains and brightfield images showing the effects of the dynein inhibitor Ciliobrevin D on planaria regeneration. A Word document contained in the zip folder provides detailed description of the different cases. (ZIP) [file pcbi.1006904.s017.zip › DatasetS9i/Dynein_inhibition/synapsin stain/Sample 4-0.jpg]

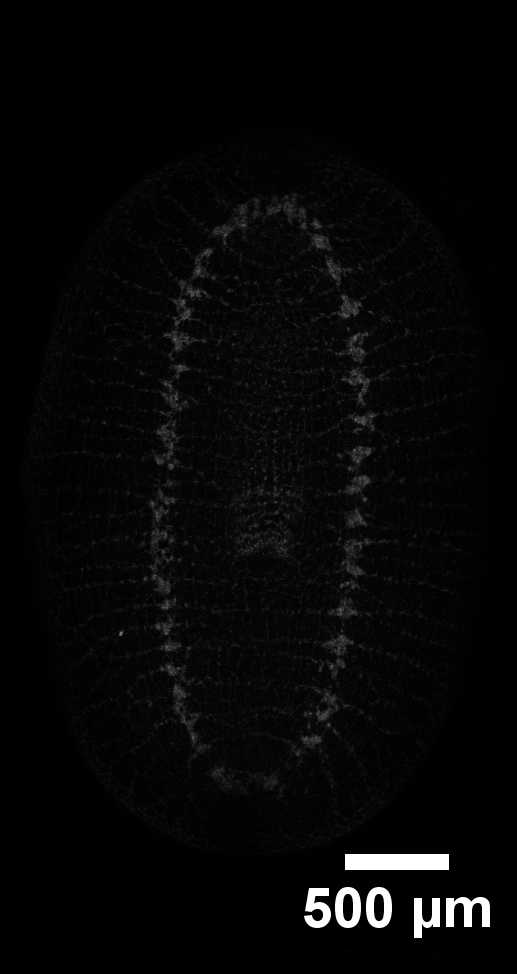

Supplement: S2 Dataset — This dataset contains raw-images of synapsin stains of uncut one- and two- headed worms, synapsin stains and brightfield images of the upwards and inverted L-cut scenarios, and synapsin stains and brightfield images showing the effects of the dynein inhibitor Ciliobrevin D on planaria regeneration. A Word document contained in the zip folder provides detailed description of the different cases. (ZIP) [file pcbi.1006904.s017.zip › DatasetS9i/Dynein_inhibition/synapsin stain/Sample 5-0.jpg]

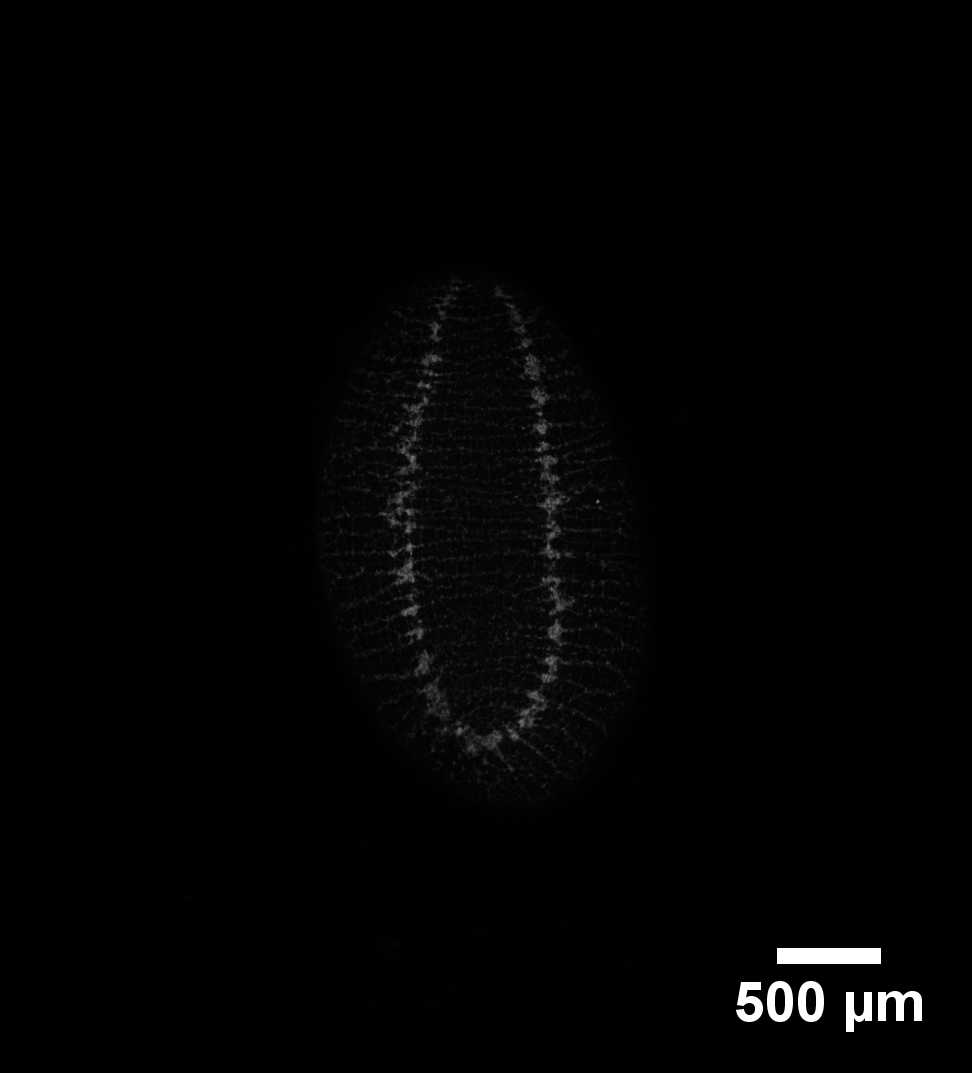

Supplement: S2 Dataset — This dataset contains raw-images of synapsin stains of uncut one- and two- headed worms, synapsin stains and brightfield images of the upwards and inverted L-cut scenarios, and synapsin stains and brightfield images showing the effects of the dynein inhibitor Ciliobrevin D on planaria regeneration. A Word document contained in the zip folder provides detailed description of the different cases. (ZIP) [file pcbi.1006904.s017.zip › DatasetS9i/Dynein_inhibition/synapsin stain/Sample 6-0.jpg]

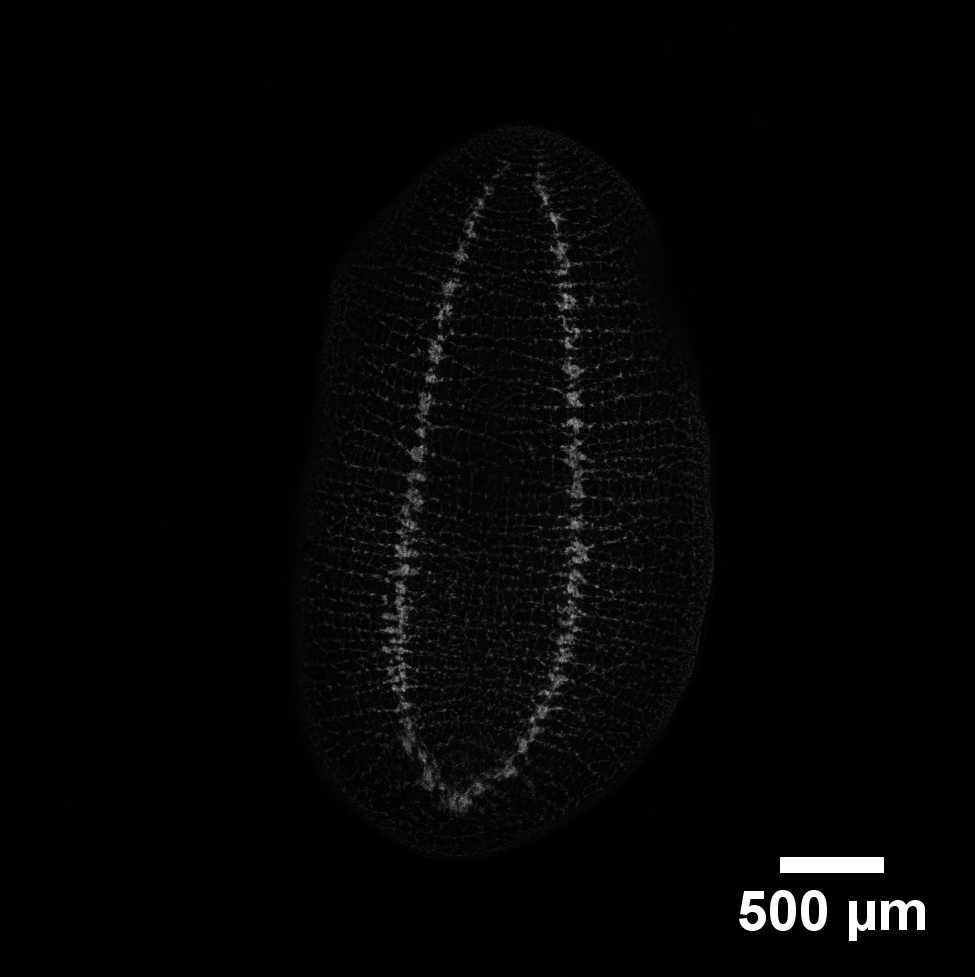

Supplement: S2 Dataset — This dataset contains raw-images of synapsin stains of uncut one- and two- headed worms, synapsin stains and brightfield images of the upwards and inverted L-cut scenarios, and synapsin stains and brightfield images showing the effects of the dynein inhibitor Ciliobrevin D on planaria regeneration. A Word document contained in the zip folder provides detailed description of the different cases. (ZIP) [file pcbi.1006904.s017.zip › DatasetS9i/Dynein_inhibition/synapsin stain/Sample 7-0.jpg]

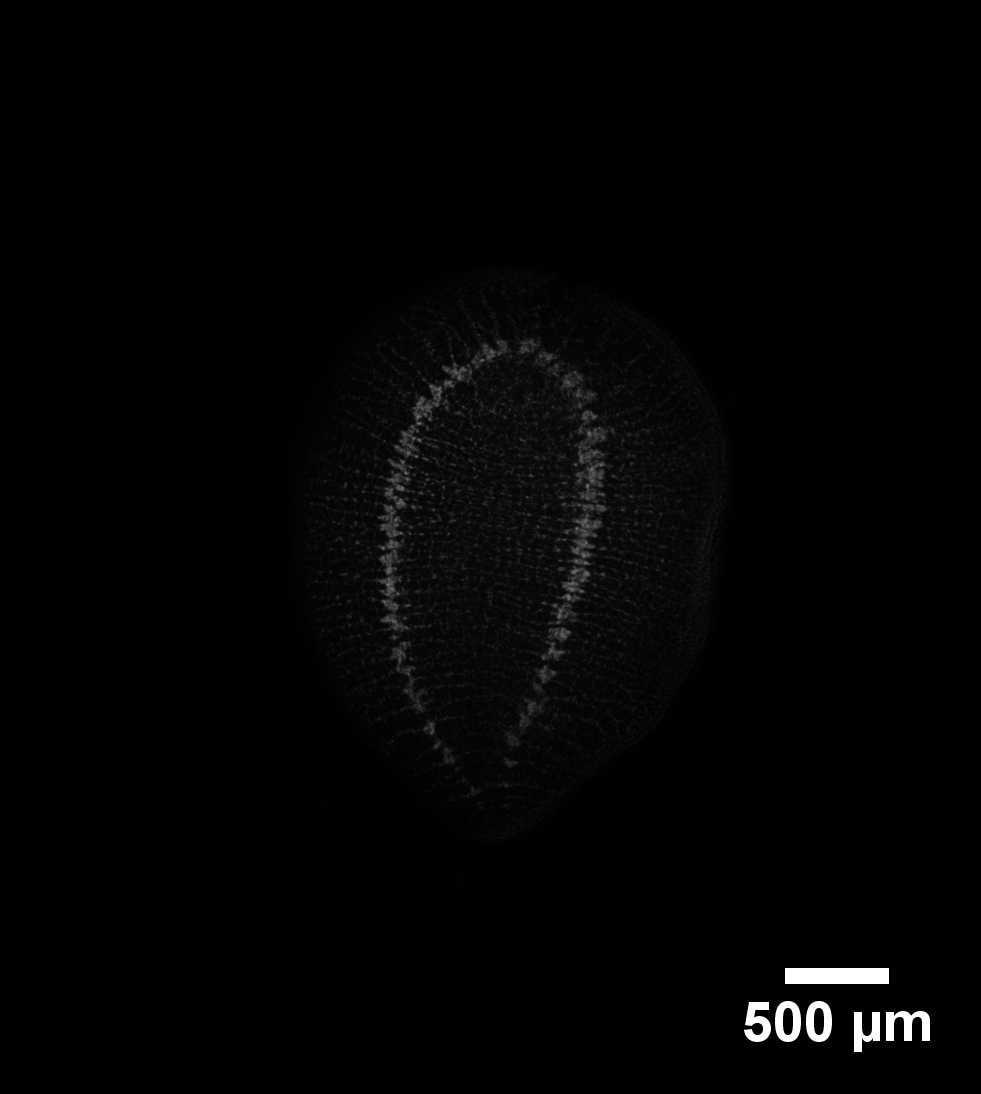

Supplement: S2 Dataset — This dataset contains raw-images of synapsin stains of uncut one- and two- headed worms, synapsin stains and brightfield images of the upwards and inverted L-cut scenarios, and synapsin stains and brightfield images showing the effects of the dynein inhibitor Ciliobrevin D on planaria regeneration. A Word document contained in the zip folder provides detailed description of the different cases. (ZIP) [file pcbi.1006904.s017.zip › DatasetS9i/Dynein_inhibition/synapsin stain/Sample 8-0.jpg]

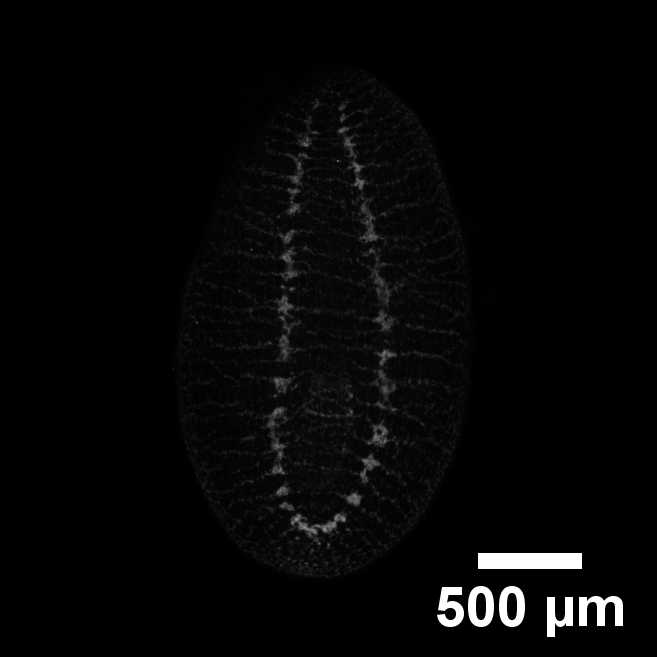

Supplement: S2 Dataset — This dataset contains raw-images of synapsin stains of uncut one- and two- headed worms, synapsin stains and brightfield images of the upwards and inverted L-cut scenarios, and synapsin stains and brightfield images showing the effects of the dynein inhibitor Ciliobrevin D on planaria regeneration. A Word document contained in the zip folder provides detailed description of the different cases. (ZIP) [file pcbi.1006904.s017.zip › DatasetS9i/Dynein_inhibition/synapsin stain/Sample 9-0.jpg]

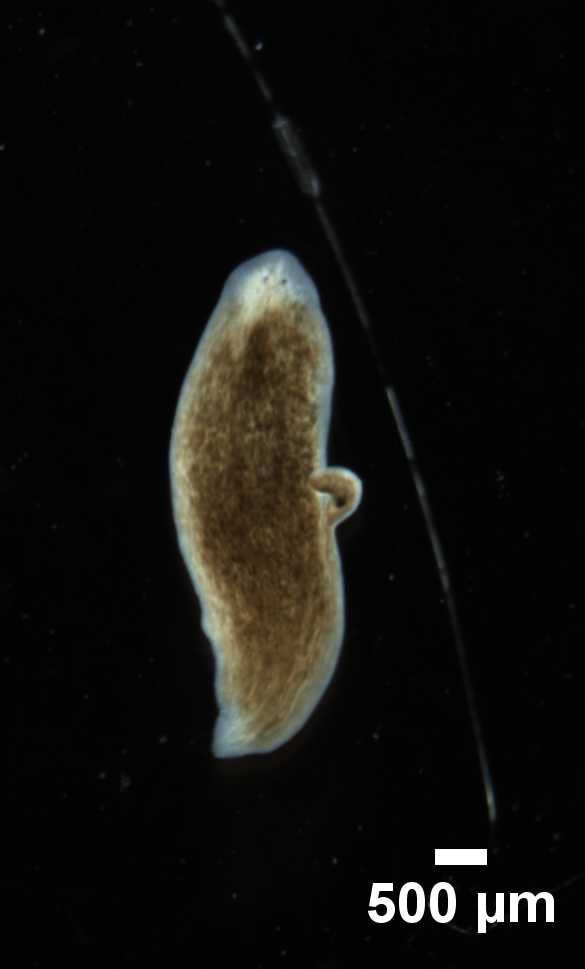

Supplement: S2 Dataset — This dataset contains raw-images of synapsin stains of uncut one- and two- headed worms, synapsin stains and brightfield images of the upwards and inverted L-cut scenarios, and synapsin stains and brightfield images showing the effects of the dynein inhibitor Ciliobrevin D on planaria regeneration. A Word document contained in the zip folder provides detailed description of the different cases. (ZIP) [file pcbi.1006904.s017.zip › DatasetS9i/L_cuts/a) control L-cut/brightfield pictures/Sample 10.jpg]

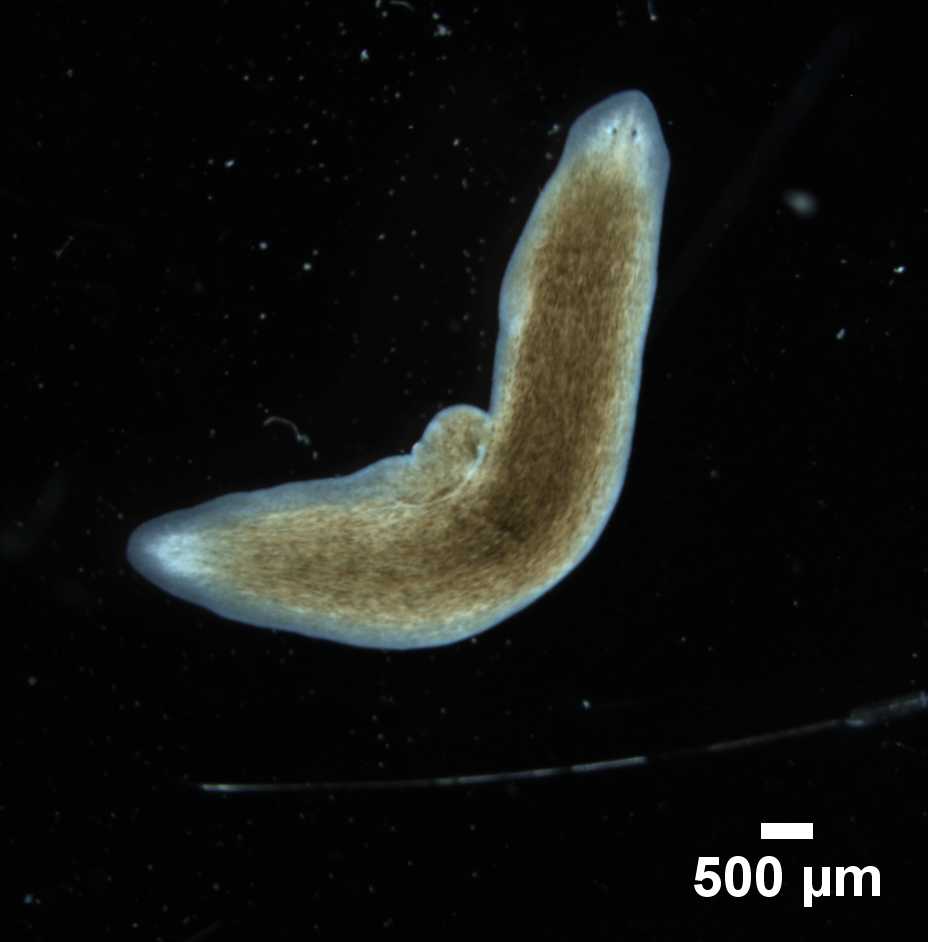

Supplement: S2 Dataset — This dataset contains raw-images of synapsin stains of uncut one- and two- headed worms, synapsin stains and brightfield images of the upwards and inverted L-cut scenarios, and synapsin stains and brightfield images showing the effects of the dynein inhibitor Ciliobrevin D on planaria regeneration. A Word document contained in the zip folder provides detailed description of the different cases. (ZIP) [file pcbi.1006904.s017.zip › DatasetS9i/L_cuts/a) control L-cut/brightfield pictures/Sample 11.jpg]

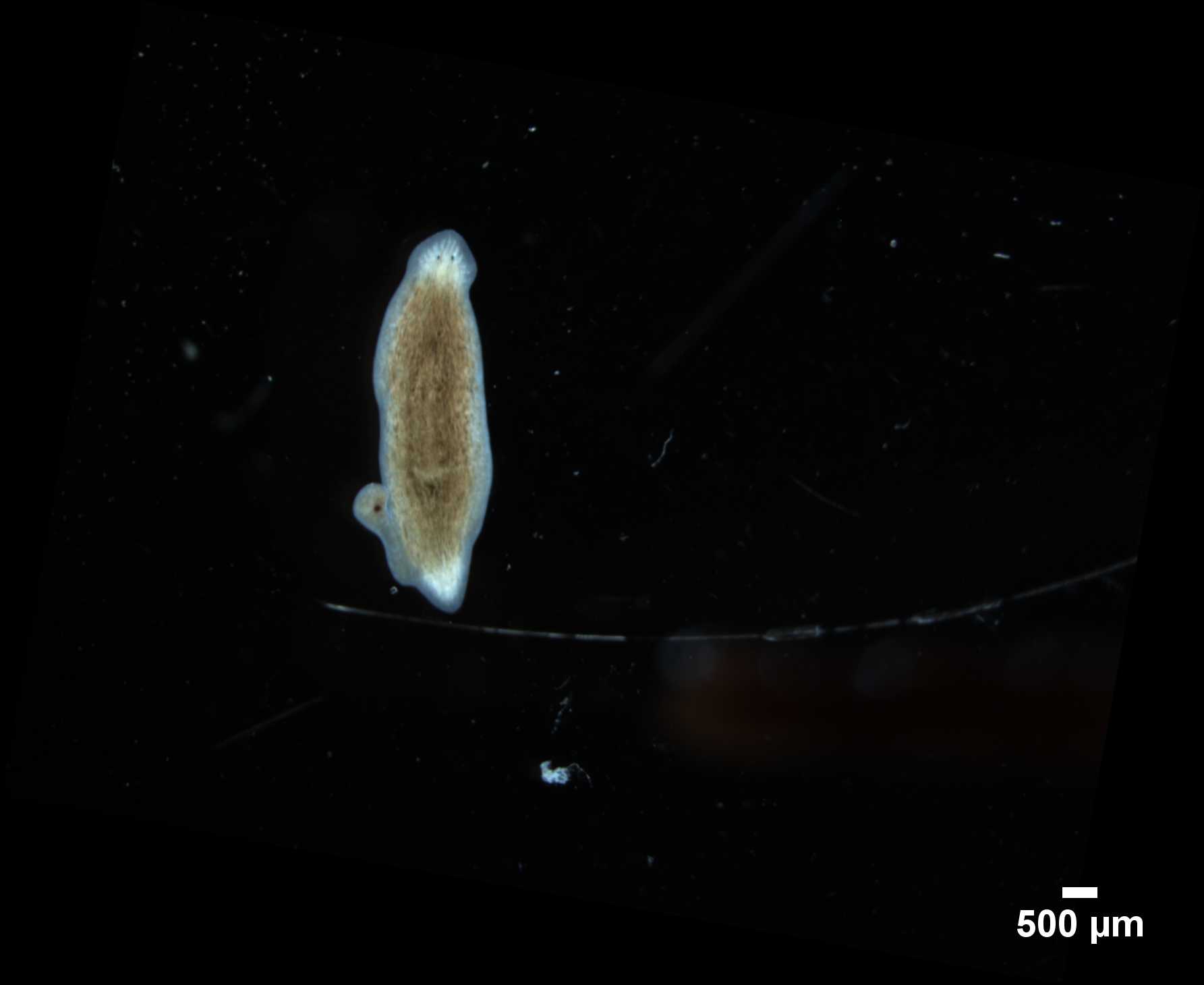

Supplement: S2 Dataset — This dataset contains raw-images of synapsin stains of uncut one- and two- headed worms, synapsin stains and brightfield images of the upwards and inverted L-cut scenarios, and synapsin stains and brightfield images showing the effects of the dynein inhibitor Ciliobrevin D on planaria regeneration. A Word document contained in the zip folder provides detailed description of the different cases. (ZIP) [file pcbi.1006904.s017.zip › DatasetS9i/L_cuts/a) control L-cut/brightfield pictures/Sample 1.jpg]

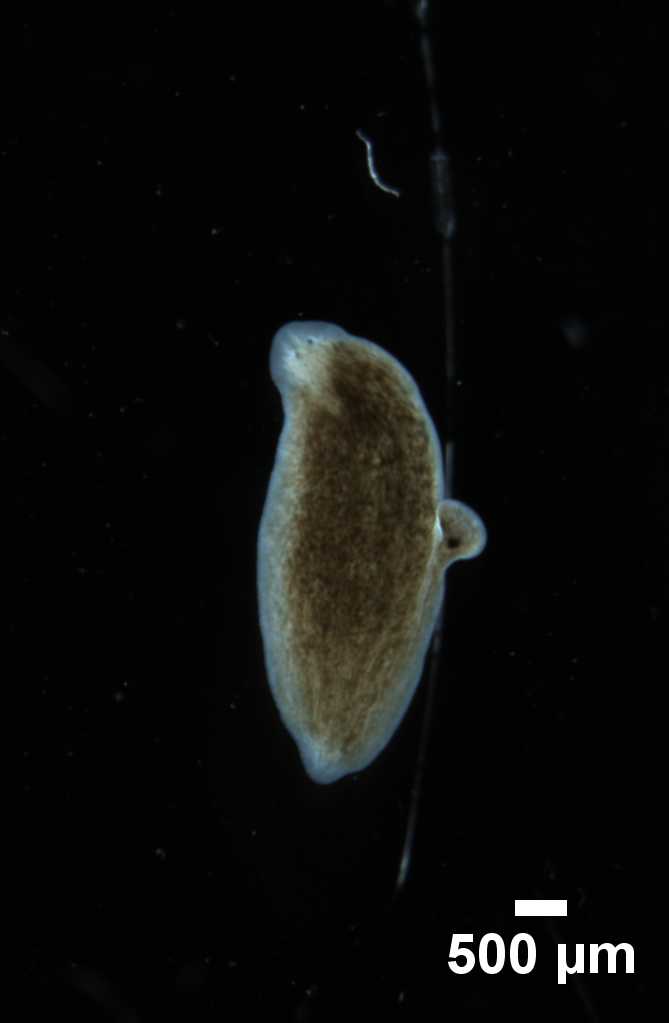

Supplement: S2 Dataset — This dataset contains raw-images of synapsin stains of uncut one- and two- headed worms, synapsin stains and brightfield images of the upwards and inverted L-cut scenarios, and synapsin stains and brightfield images showing the effects of the dynein inhibitor Ciliobrevin D on planaria regeneration. A Word document contained in the zip folder provides detailed description of the different cases. (ZIP) [file pcbi.1006904.s017.zip › DatasetS9i/L_cuts/a) control L-cut/brightfield pictures/Sample 2.jpg]

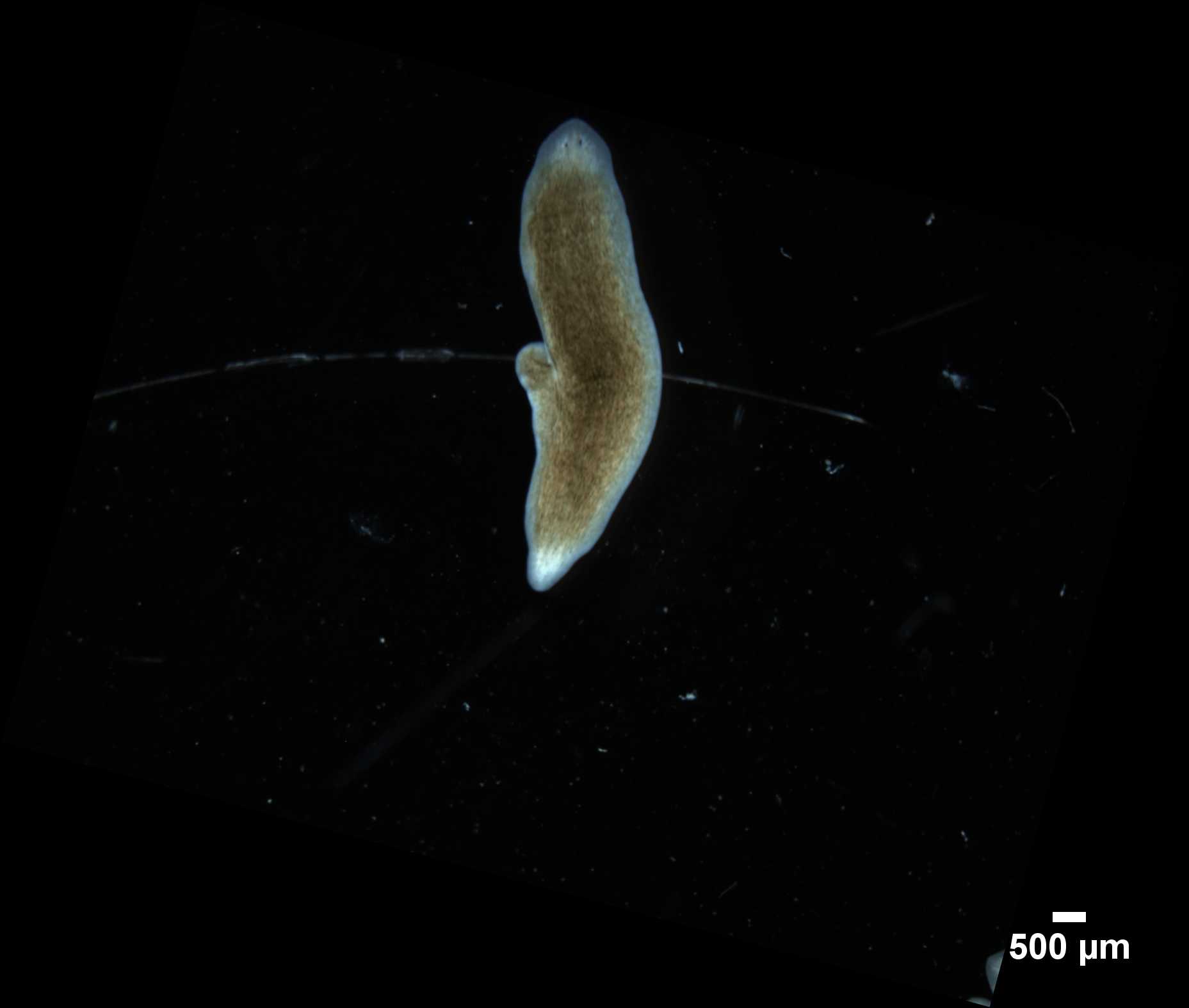

Supplement: S2 Dataset — This dataset contains raw-images of synapsin stains of uncut one- and two- headed worms, synapsin stains and brightfield images of the upwards and inverted L-cut scenarios, and synapsin stains and brightfield images showing the effects of the dynein inhibitor Ciliobrevin D on planaria regeneration. A Word document contained in the zip folder provides detailed description of the different cases. (ZIP) [file pcbi.1006904.s017.zip › DatasetS9i/L_cuts/a) control L-cut/brightfield pictures/Sample 3.jpg]

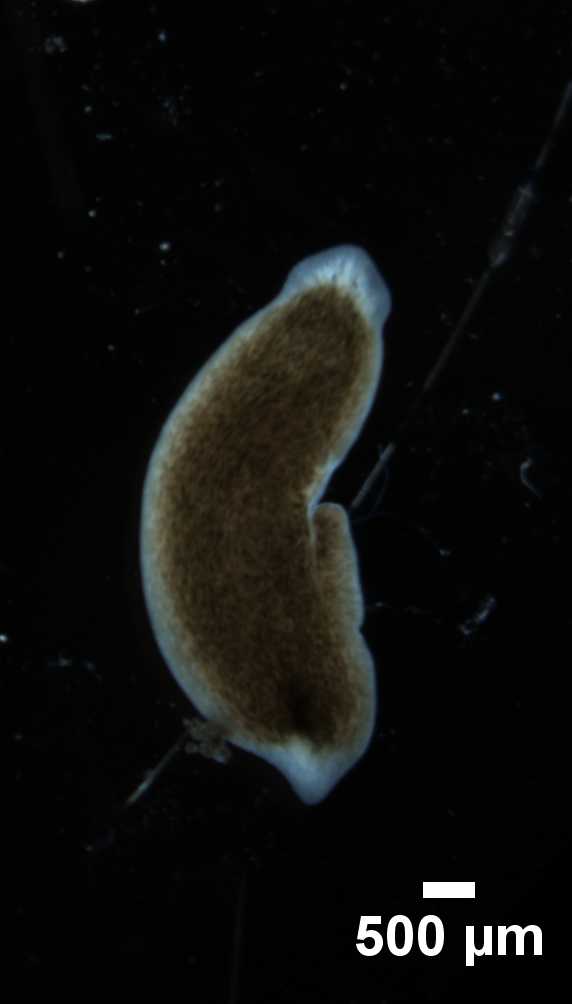

Supplement: S2 Dataset — This dataset contains raw-images of synapsin stains of uncut one- and two- headed worms, synapsin stains and brightfield images of the upwards and inverted L-cut scenarios, and synapsin stains and brightfield images showing the effects of the dynein inhibitor Ciliobrevin D on planaria regeneration. A Word document contained in the zip folder provides detailed description of the different cases. (ZIP) [file pcbi.1006904.s017.zip › DatasetS9i/L_cuts/a) control L-cut/brightfield pictures/Sample 4.jpg]

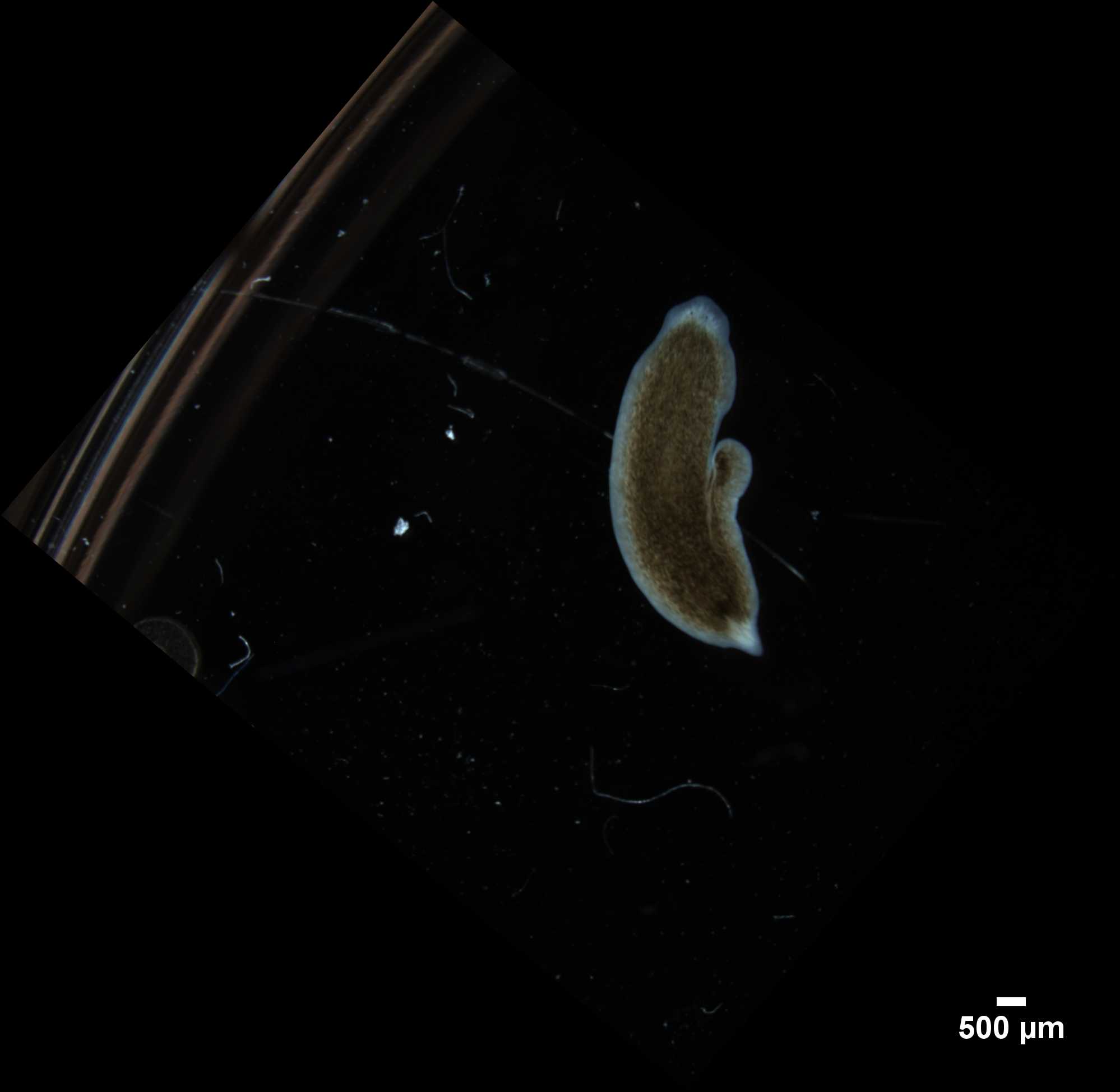

Supplement: S2 Dataset — This dataset contains raw-images of synapsin stains of uncut one- and two- headed worms, synapsin stains and brightfield images of the upwards and inverted L-cut scenarios, and synapsin stains and brightfield images showing the effects of the dynein inhibitor Ciliobrevin D on planaria regeneration. A Word document contained in the zip folder provides detailed description of the different cases. (ZIP) [file pcbi.1006904.s017.zip › DatasetS9i/L_cuts/a) control L-cut/brightfield pictures/Sample 5.jpg]

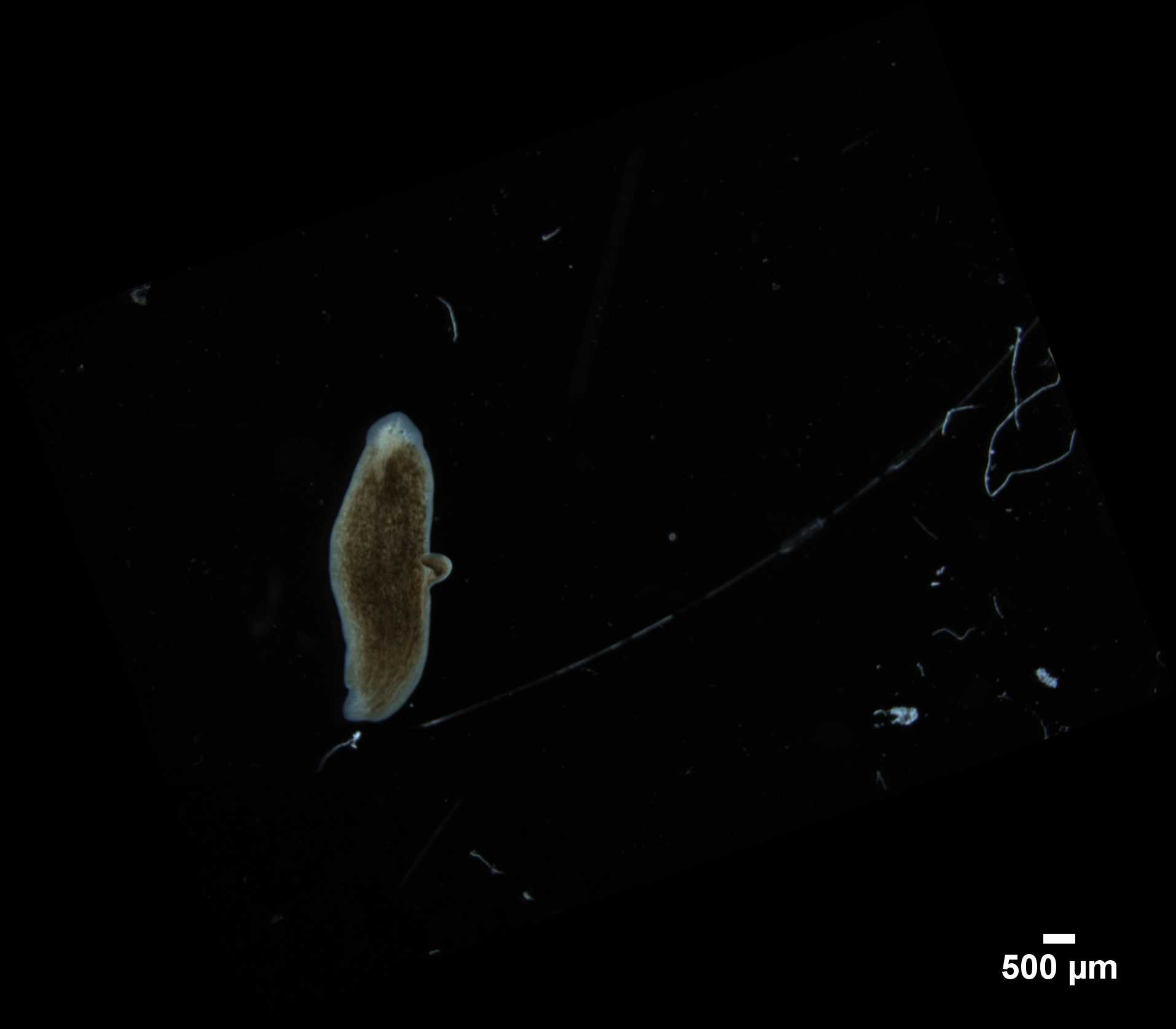

Supplement: S2 Dataset — This dataset contains raw-images of synapsin stains of uncut one- and two- headed worms, synapsin stains and brightfield images of the upwards and inverted L-cut scenarios, and synapsin stains and brightfield images showing the effects of the dynein inhibitor Ciliobrevin D on planaria regeneration. A Word document contained in the zip folder provides detailed description of the different cases. (ZIP) [file pcbi.1006904.s017.zip › DatasetS9i/L_cuts/a) control L-cut/brightfield pictures/Sample 6.jpg]

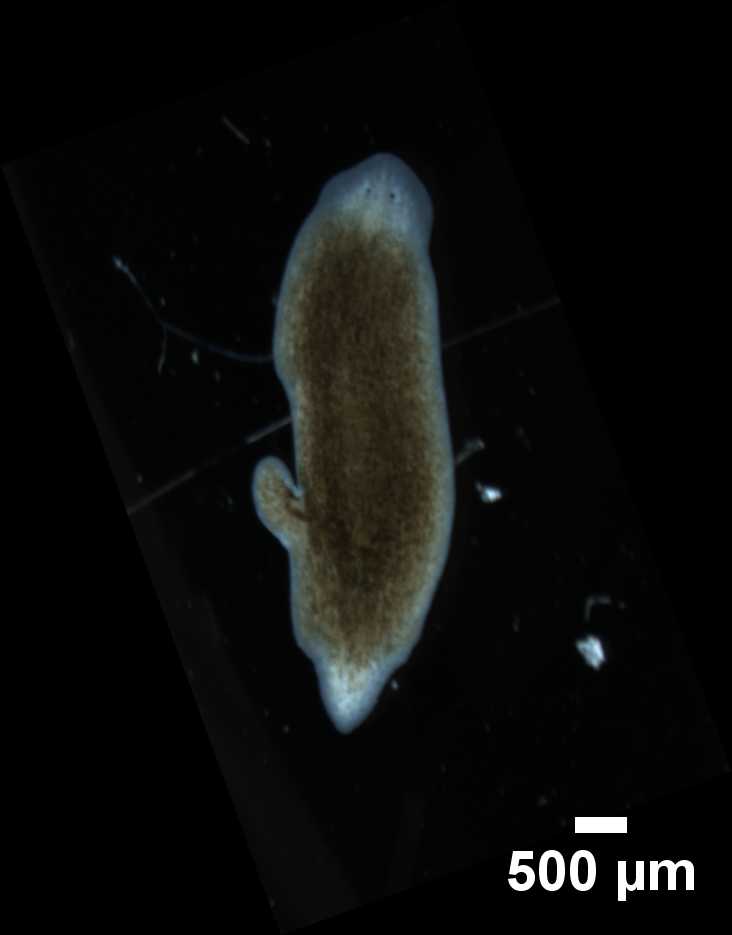

Supplement: S2 Dataset — This dataset contains raw-images of synapsin stains of uncut one- and two- headed worms, synapsin stains and brightfield images of the upwards and inverted L-cut scenarios, and synapsin stains and brightfield images showing the effects of the dynein inhibitor Ciliobrevin D on planaria regeneration. A Word document contained in the zip folder provides detailed description of the different cases. (ZIP) [file pcbi.1006904.s017.zip › DatasetS9i/L_cuts/a) control L-cut/brightfield pictures/Sample 7.jpg]

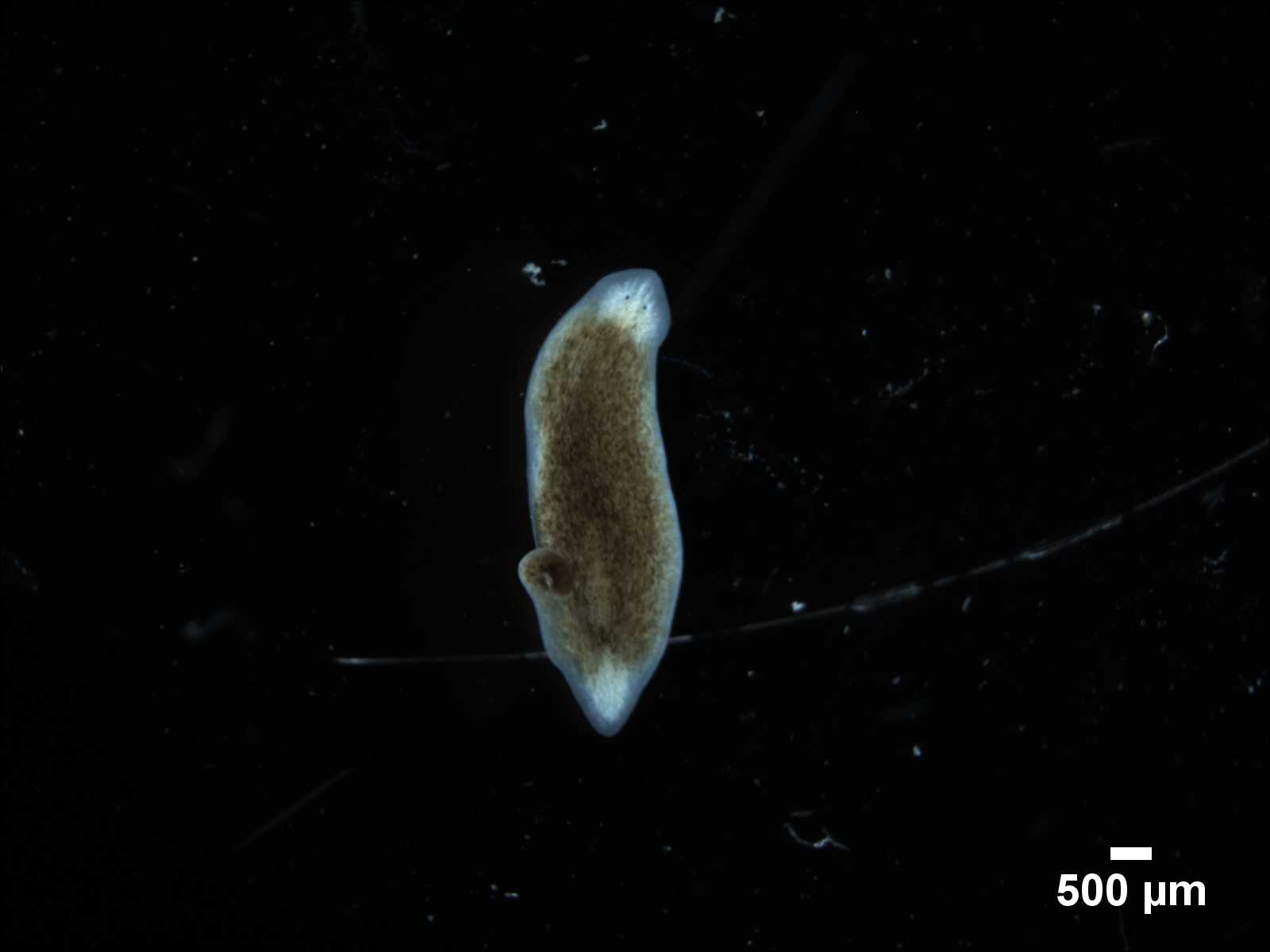

Supplement: S2 Dataset — This dataset contains raw-images of synapsin stains of uncut one- and two- headed worms, synapsin stains and brightfield images of the upwards and inverted L-cut scenarios, and synapsin stains and brightfield images showing the effects of the dynein inhibitor Ciliobrevin D on planaria regeneration. A Word document contained in the zip folder provides detailed description of the different cases. (ZIP) [file pcbi.1006904.s017.zip › DatasetS9i/L_cuts/a) control L-cut/brightfield pictures/Sample 8.jpg]

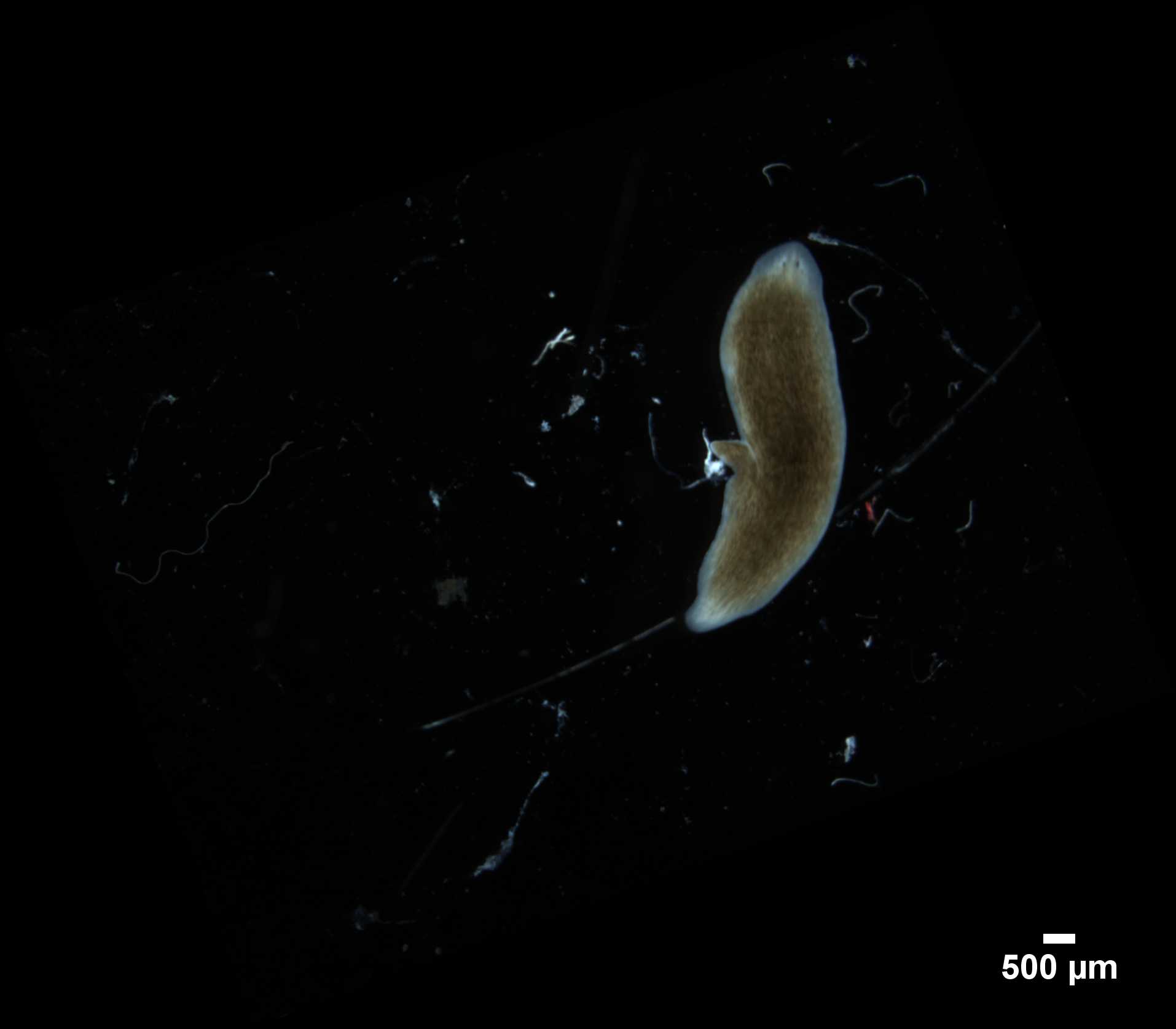

Supplement: S2 Dataset — This dataset contains raw-images of synapsin stains of uncut one- and two- headed worms, synapsin stains and brightfield images of the upwards and inverted L-cut scenarios, and synapsin stains and brightfield images showing the effects of the dynein inhibitor Ciliobrevin D on planaria regeneration. A Word document contained in the zip folder provides detailed description of the different cases. (ZIP) [file pcbi.1006904.s017.zip › DatasetS9i/L_cuts/a) control L-cut/brightfield pictures/Sample 9.jpg]

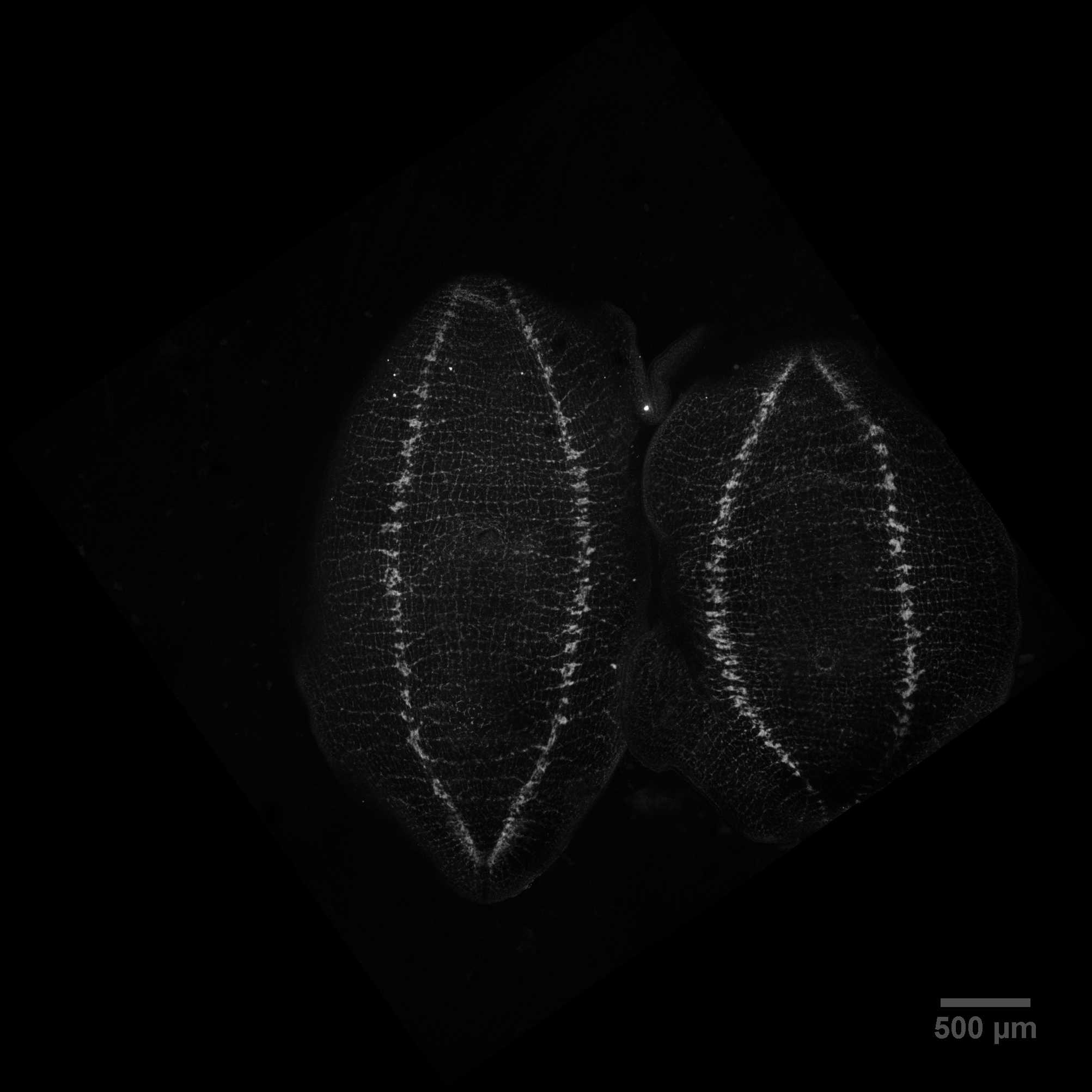

Supplement: S2 Dataset — This dataset contains raw-images of synapsin stains of uncut one- and two- headed worms, synapsin stains and brightfield images of the upwards and inverted L-cut scenarios, and synapsin stains and brightfield images showing the effects of the dynein inhibitor Ciliobrevin D on planaria regeneration. A Word document contained in the zip folder provides detailed description of the different cases. (ZIP) [file pcbi.1006904.s017.zip › DatasetS9i/L_cuts/a) control L-cut/synapsin stain/Sample 1, 2.jpg]

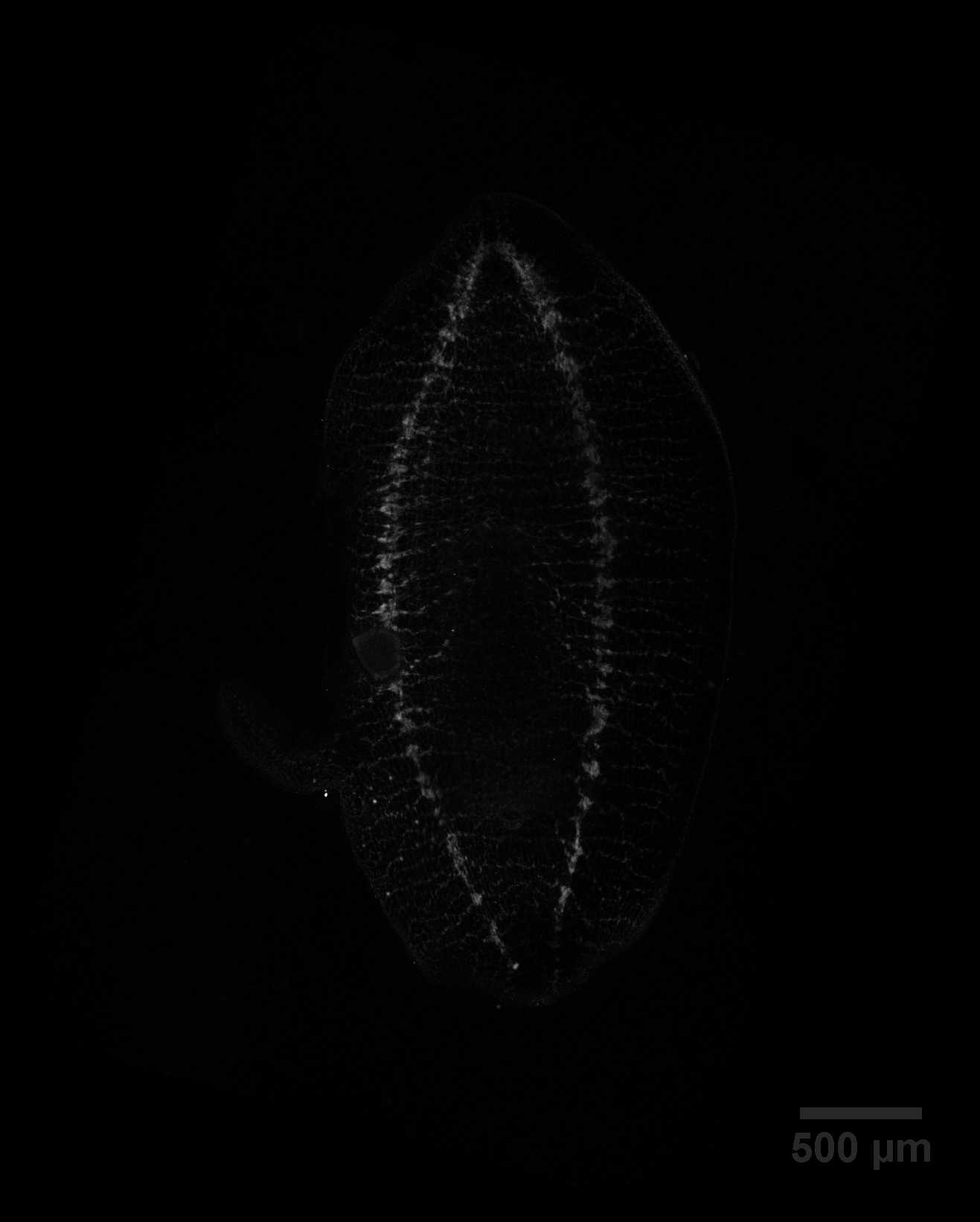

Supplement: S2 Dataset — This dataset contains raw-images of synapsin stains of uncut one- and two- headed worms, synapsin stains and brightfield images of the upwards and inverted L-cut scenarios, and synapsin stains and brightfield images showing the effects of the dynein inhibitor Ciliobrevin D on planaria regeneration. A Word document contained in the zip folder provides detailed description of the different cases. (ZIP) [file pcbi.1006904.s017.zip › DatasetS9i/L_cuts/a) control L-cut/synapsin stain/Sample 3.jpg]

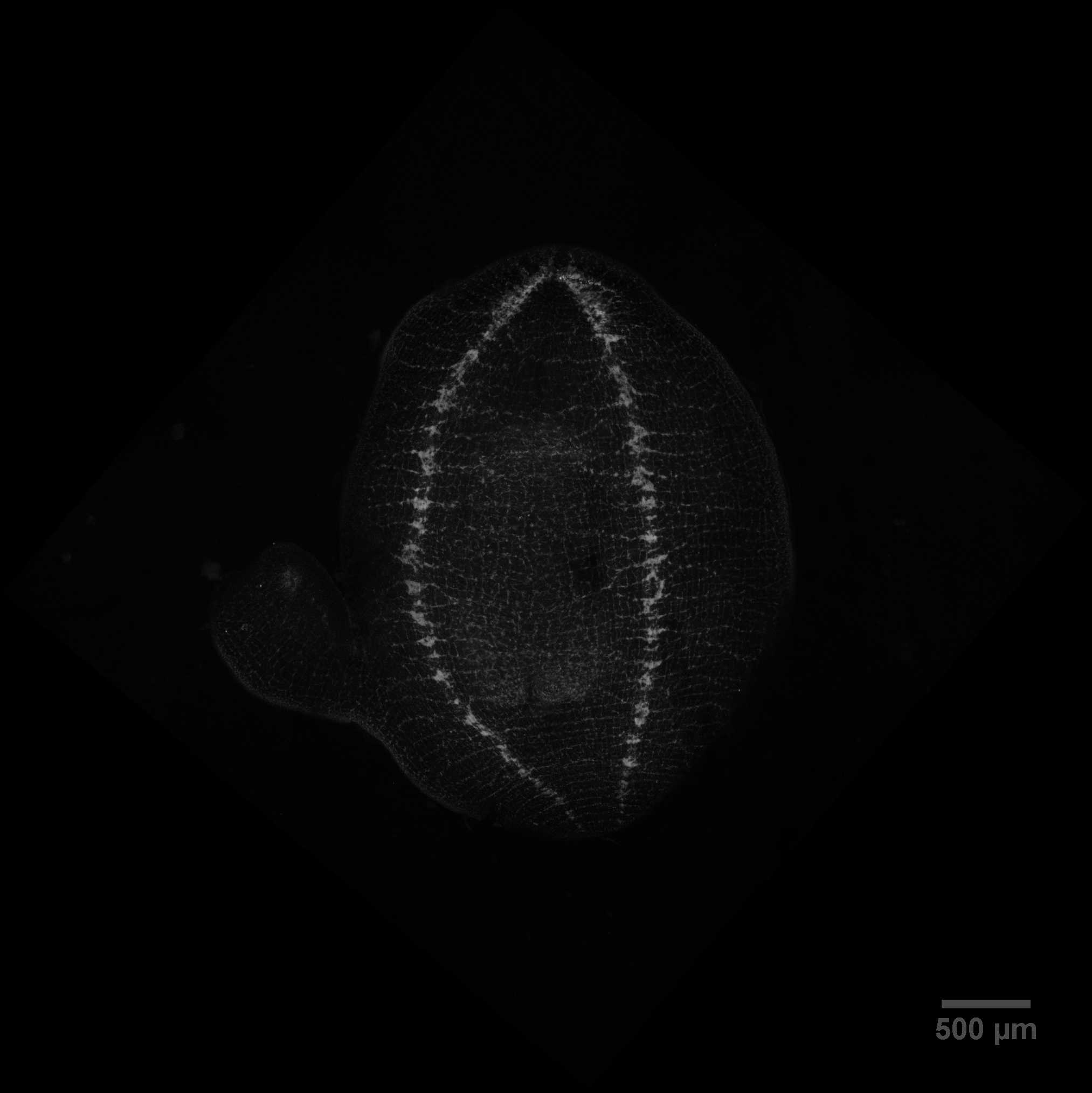

Supplement: S2 Dataset — This dataset contains raw-images of synapsin stains of uncut one- and two- headed worms, synapsin stains and brightfield images of the upwards and inverted L-cut scenarios, and synapsin stains and brightfield images showing the effects of the dynein inhibitor Ciliobrevin D on planaria regeneration. A Word document contained in the zip folder provides detailed description of the different cases. (ZIP) [file pcbi.1006904.s017.zip › DatasetS9i/L_cuts/a) control L-cut/synapsin stain/Sample 4.jpg]

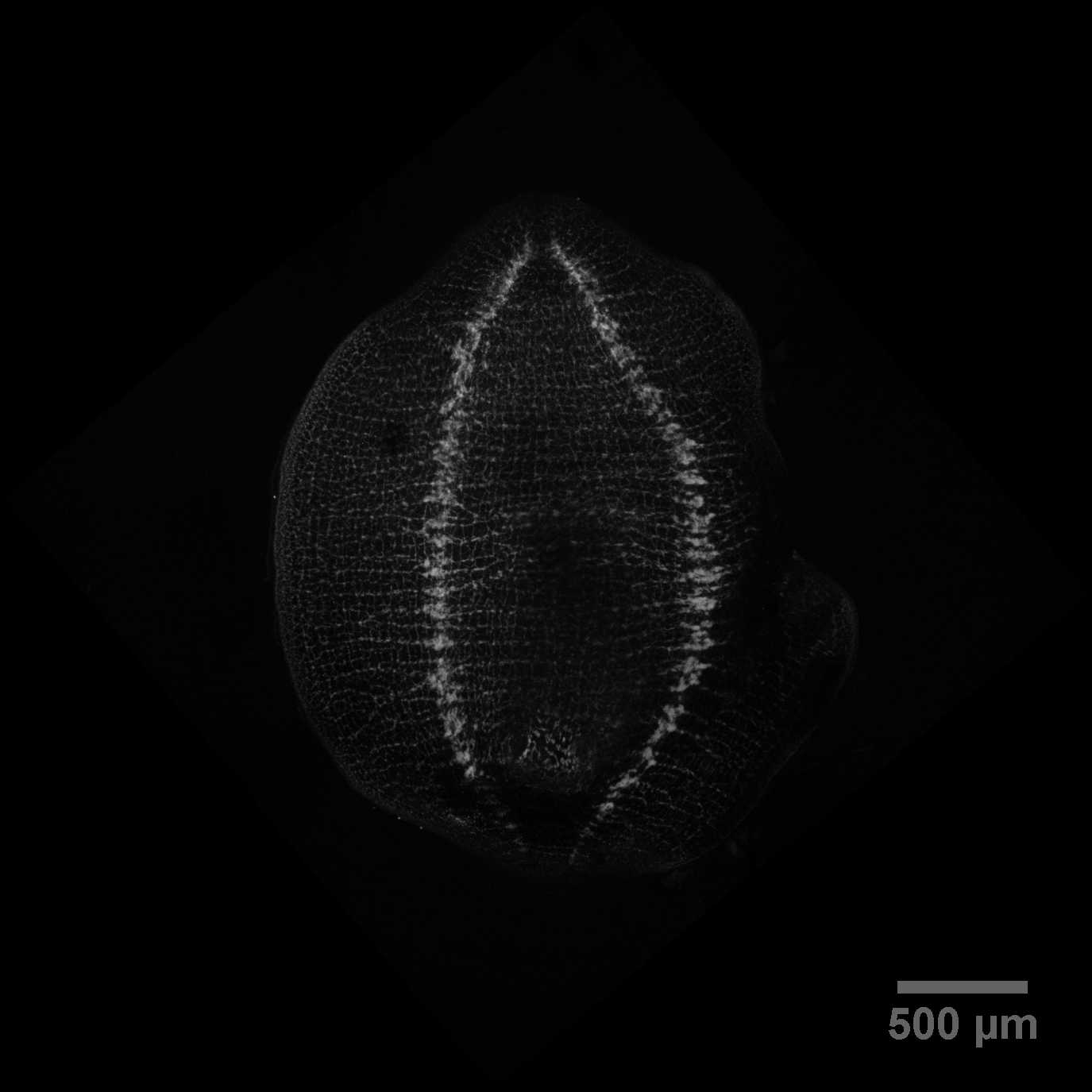

Supplement: S2 Dataset — This dataset contains raw-images of synapsin stains of uncut one- and two- headed worms, synapsin stains and brightfield images of the upwards and inverted L-cut scenarios, and synapsin stains and brightfield images showing the effects of the dynein inhibitor Ciliobrevin D on planaria regeneration. A Word document contained in the zip folder provides detailed description of the different cases. (ZIP) [file pcbi.1006904.s017.zip › DatasetS9i/L_cuts/a) control L-cut/synapsin stain/Sample 5.jpg]

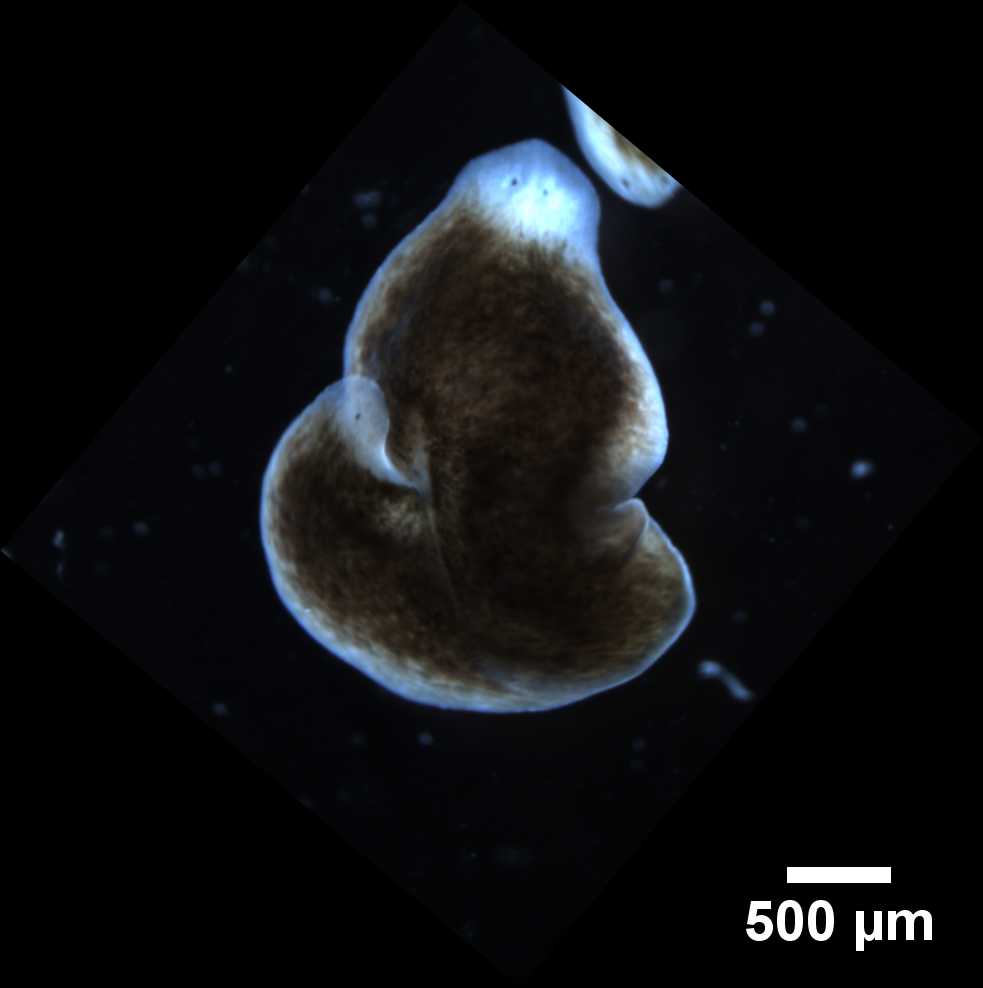

Supplement: S2 Dataset — This dataset contains raw-images of synapsin stains of uncut one- and two- headed worms, synapsin stains and brightfield images of the upwards and inverted L-cut scenarios, and synapsin stains and brightfield images showing the effects of the dynein inhibitor Ciliobrevin D on planaria regeneration. A Word document contained in the zip folder provides detailed description of the different cases. (ZIP) [file pcbi.1006904.s017.zip › DatasetS9i/L_cuts/b) downwards L-cut/brightfield pictures/Sample 10.jpg]

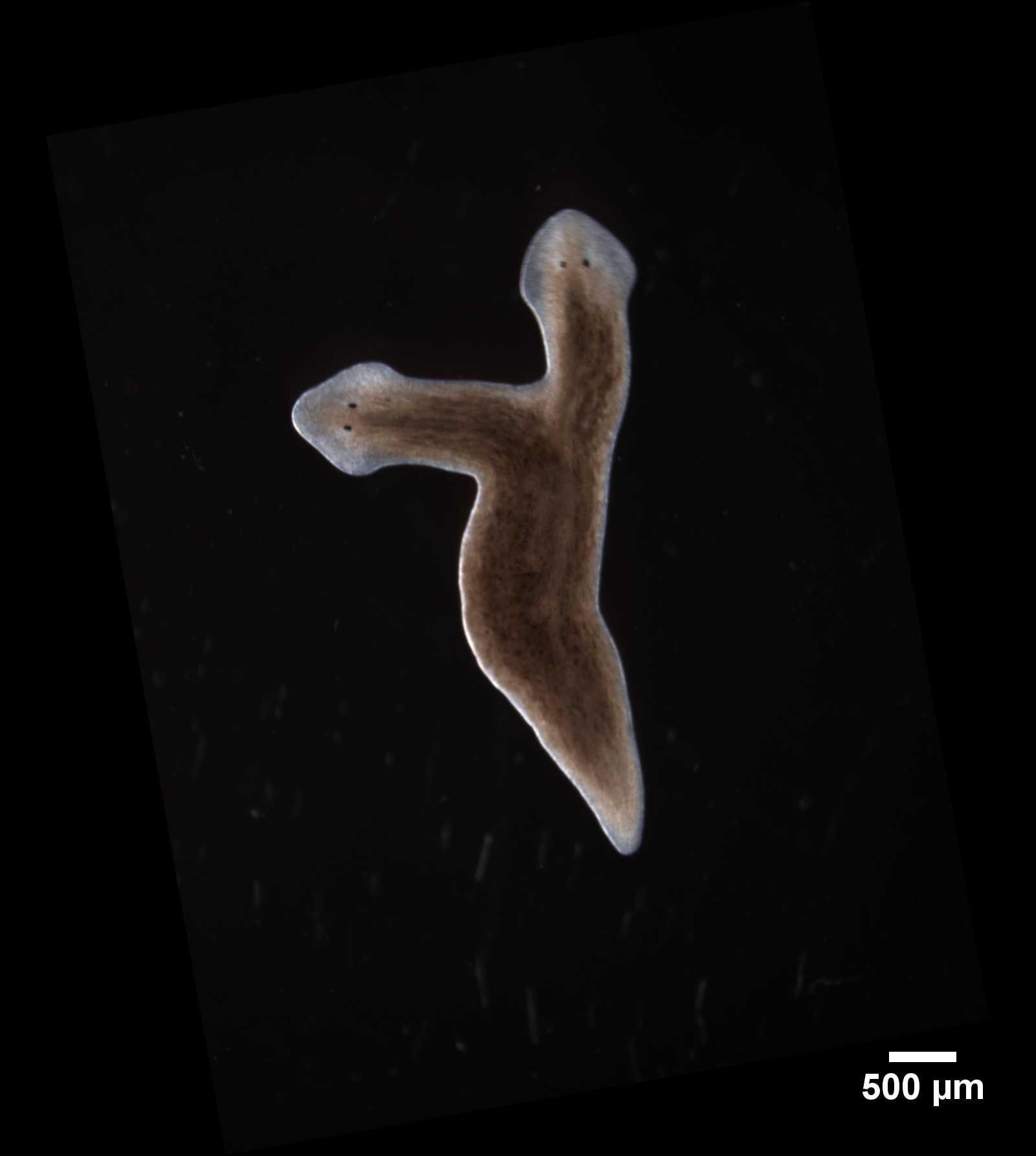

Supplement: S2 Dataset — This dataset contains raw-images of synapsin stains of uncut one- and two- headed worms, synapsin stains and brightfield images of the upwards and inverted L-cut scenarios, and synapsin stains and brightfield images showing the effects of the dynein inhibitor Ciliobrevin D on planaria regeneration. A Word document contained in the zip folder provides detailed description of the different cases. (ZIP) [file pcbi.1006904.s017.zip › DatasetS9i/L_cuts/b) downwards L-cut/brightfield pictures/Sample 11.jpg]

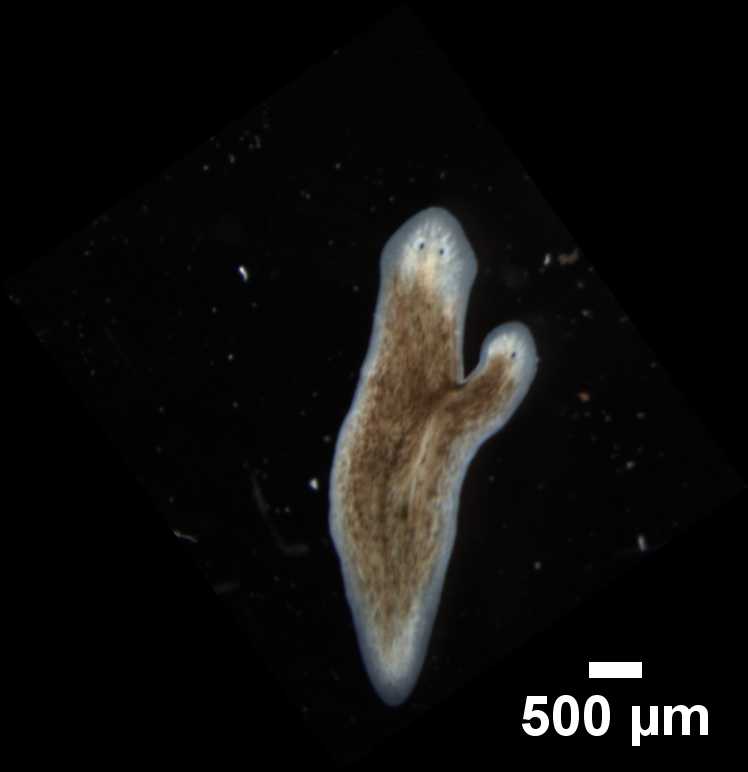

Supplement: S2 Dataset — This dataset contains raw-images of synapsin stains of uncut one- and two- headed worms, synapsin stains and brightfield images of the upwards and inverted L-cut scenarios, and synapsin stains and brightfield images showing the effects of the dynein inhibitor Ciliobrevin D on planaria regeneration. A Word document contained in the zip folder provides detailed description of the different cases. (ZIP) [file pcbi.1006904.s017.zip › DatasetS9i/L_cuts/b) downwards L-cut/brightfield pictures/Sample 12.jpg]

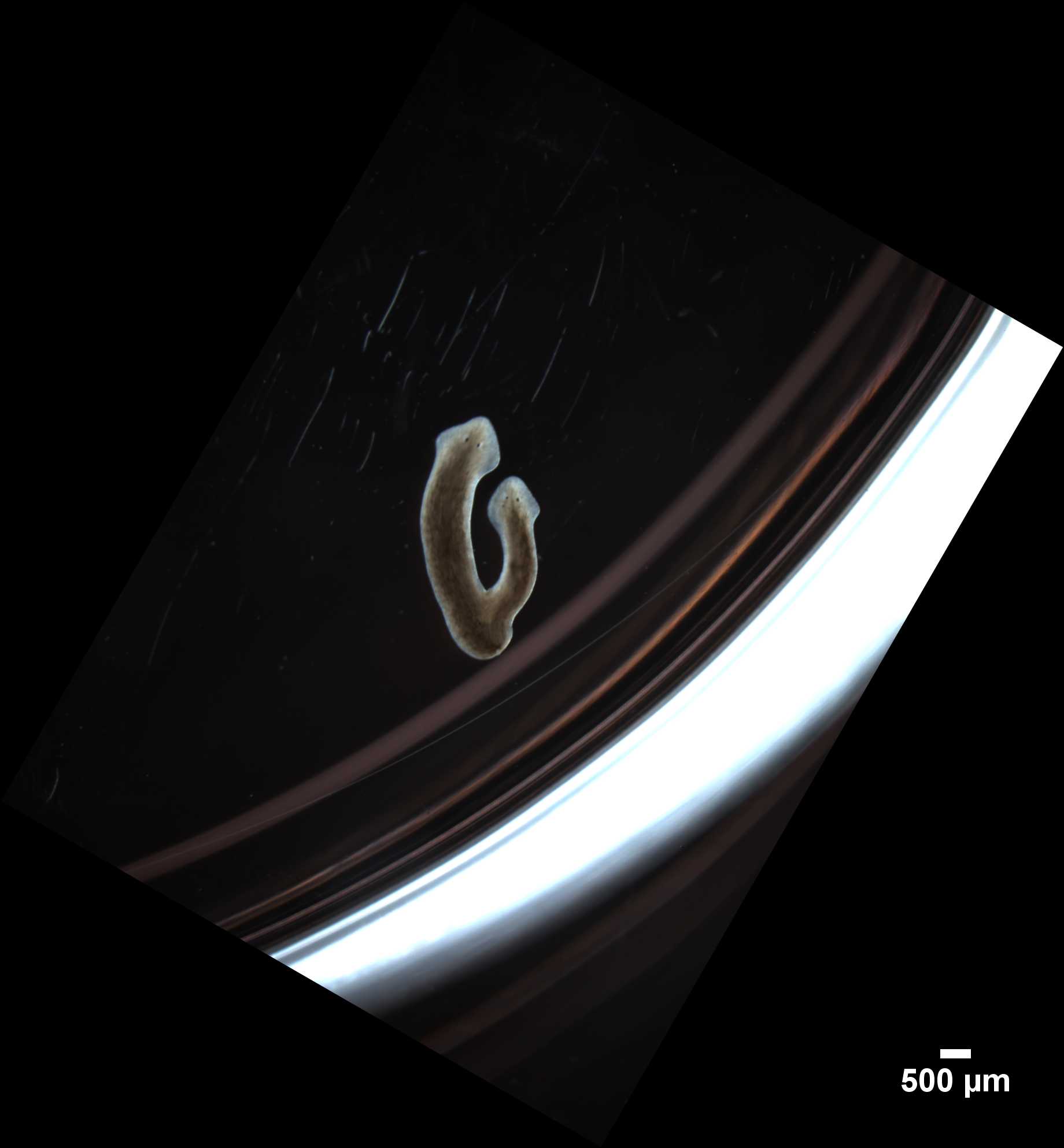

Supplement: S2 Dataset — This dataset contains raw-images of synapsin stains of uncut one- and two- headed worms, synapsin stains and brightfield images of the upwards and inverted L-cut scenarios, and synapsin stains and brightfield images showing the effects of the dynein inhibitor Ciliobrevin D on planaria regeneration. A Word document contained in the zip folder provides detailed description of the different cases. (ZIP) [file pcbi.1006904.s017.zip › DatasetS9i/L_cuts/b) downwards L-cut/brightfield pictures/Sample 13.jpg]

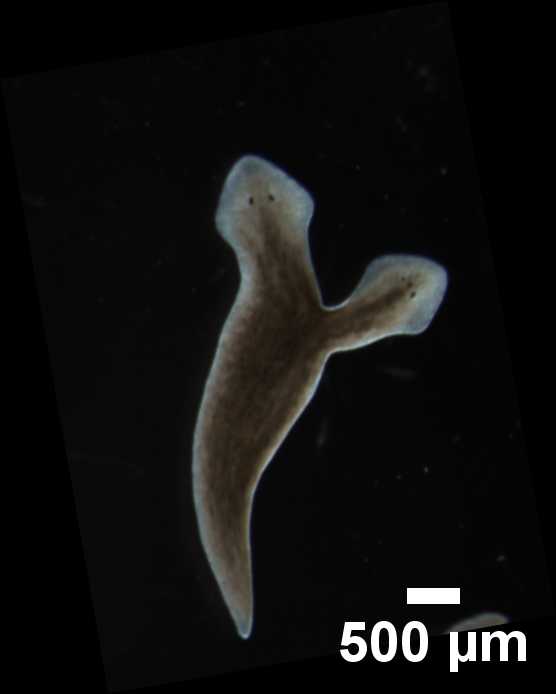

Supplement: S2 Dataset — This dataset contains raw-images of synapsin stains of uncut one- and two- headed worms, synapsin stains and brightfield images of the upwards and inverted L-cut scenarios, and synapsin stains and brightfield images showing the effects of the dynein inhibitor Ciliobrevin D on planaria regeneration. A Word document contained in the zip folder provides detailed description of the different cases. (ZIP) [file pcbi.1006904.s017.zip › DatasetS9i/L_cuts/b) downwards L-cut/brightfield pictures/Sample 14.jpg]

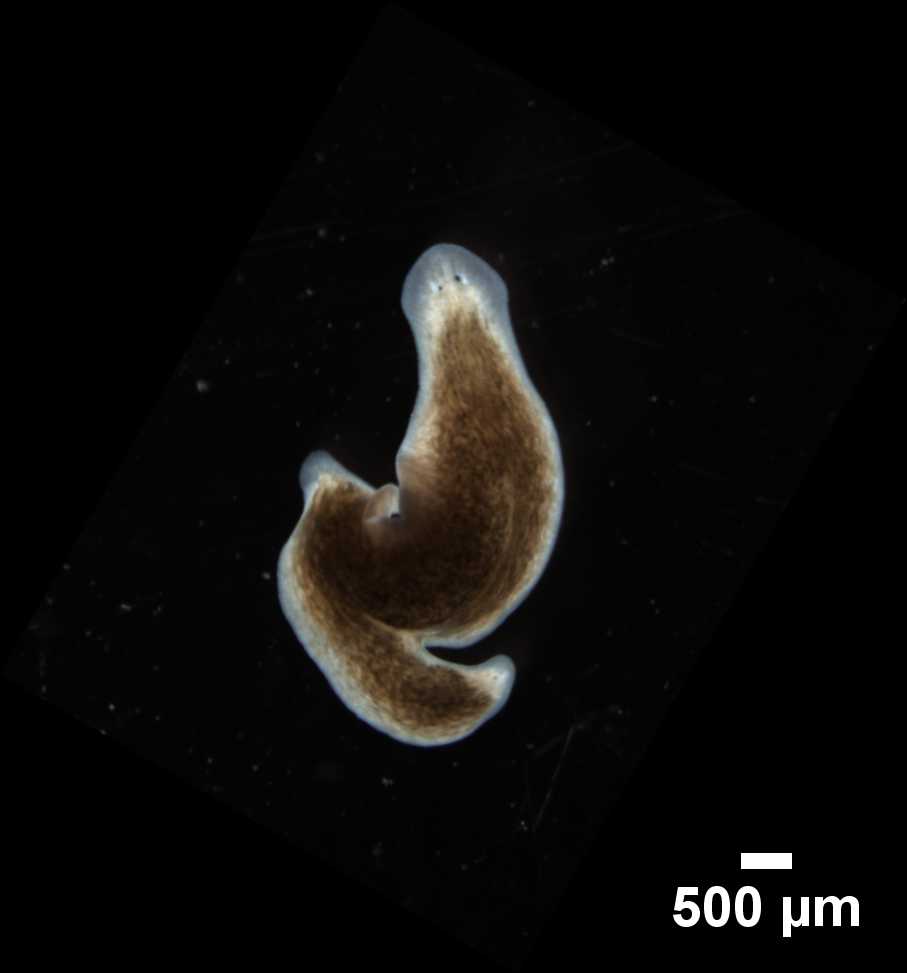

Supplement: S2 Dataset — This dataset contains raw-images of synapsin stains of uncut one- and two- headed worms, synapsin stains and brightfield images of the upwards and inverted L-cut scenarios, and synapsin stains and brightfield images showing the effects of the dynein inhibitor Ciliobrevin D on planaria regeneration. A Word document contained in the zip folder provides detailed description of the different cases. (ZIP) [file pcbi.1006904.s017.zip › DatasetS9i/L_cuts/b) downwards L-cut/brightfield pictures/Sample 1.jpg]

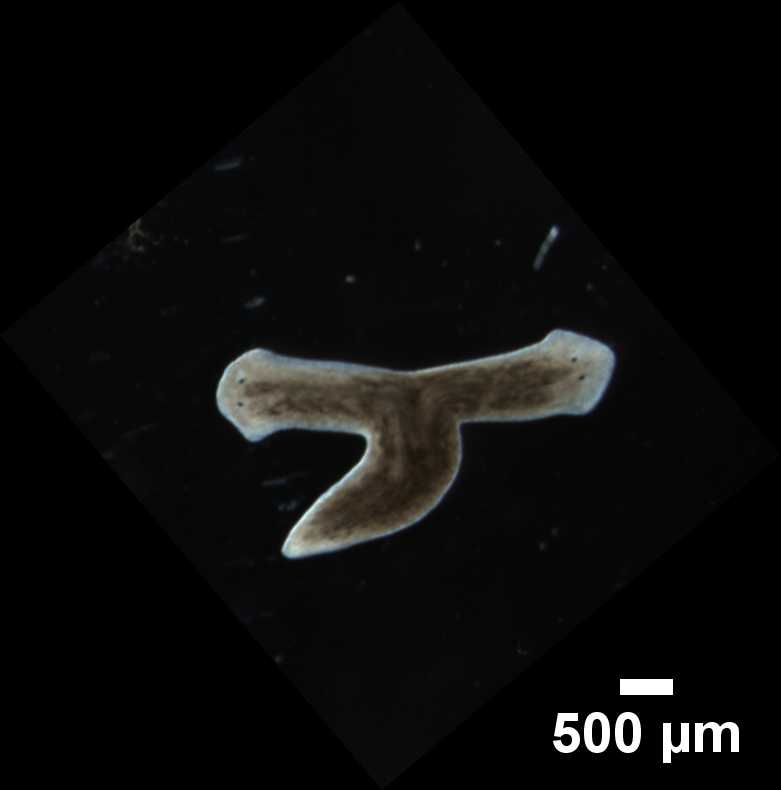

Supplement: S2 Dataset — This dataset contains raw-images of synapsin stains of uncut one- and two- headed worms, synapsin stains and brightfield images of the upwards and inverted L-cut scenarios, and synapsin stains and brightfield images showing the effects of the dynein inhibitor Ciliobrevin D on planaria regeneration. A Word document contained in the zip folder provides detailed description of the different cases. (ZIP) [file pcbi.1006904.s017.zip › DatasetS9i/L_cuts/b) downwards L-cut/brightfield pictures/Sample 2.jpg]

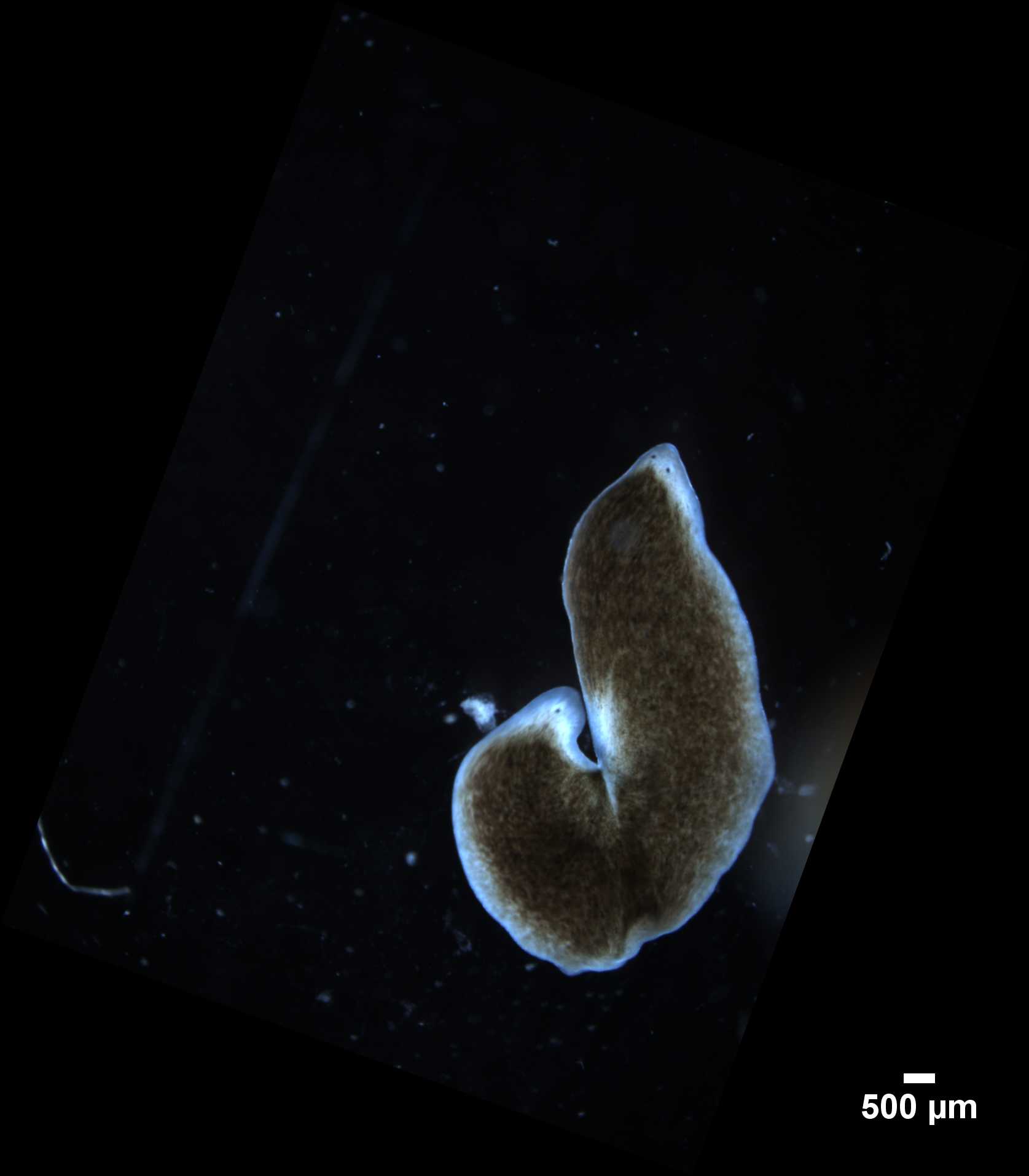

Supplement: S2 Dataset — This dataset contains raw-images of synapsin stains of uncut one- and two- headed worms, synapsin stains and brightfield images of the upwards and inverted L-cut scenarios, and synapsin stains and brightfield images showing the effects of the dynein inhibitor Ciliobrevin D on planaria regeneration. A Word document contained in the zip folder provides detailed description of the different cases. (ZIP) [file pcbi.1006904.s017.zip › DatasetS9i/L_cuts/b) downwards L-cut/brightfield pictures/Sample 3.jpg]

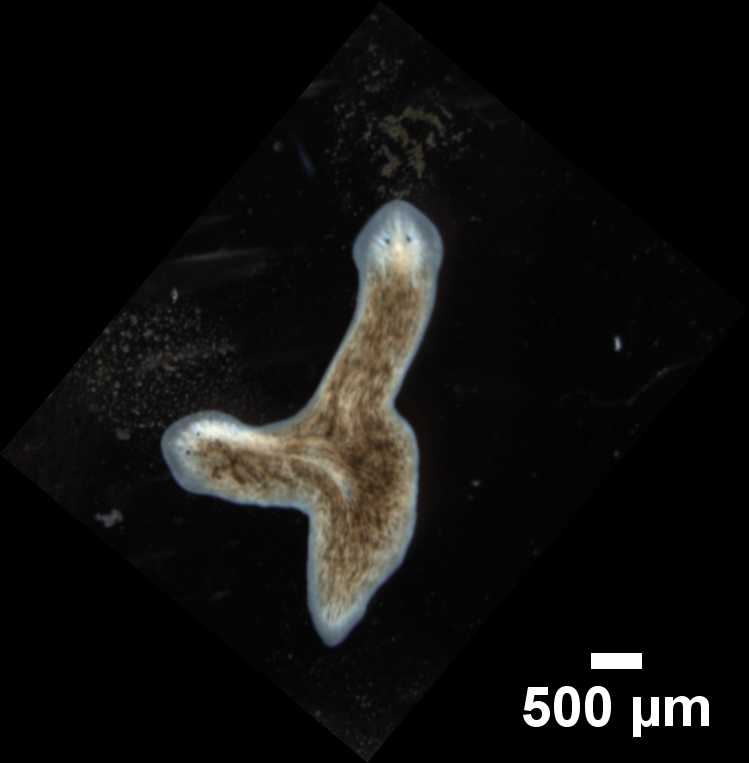

Supplement: S2 Dataset — This dataset contains raw-images of synapsin stains of uncut one- and two- headed worms, synapsin stains and brightfield images of the upwards and inverted L-cut scenarios, and synapsin stains and brightfield images showing the effects of the dynein inhibitor Ciliobrevin D on planaria regeneration. A Word document contained in the zip folder provides detailed description of the different cases. (ZIP) [file pcbi.1006904.s017.zip › DatasetS9i/L_cuts/b) downwards L-cut/brightfield pictures/Sample 4.jpg]

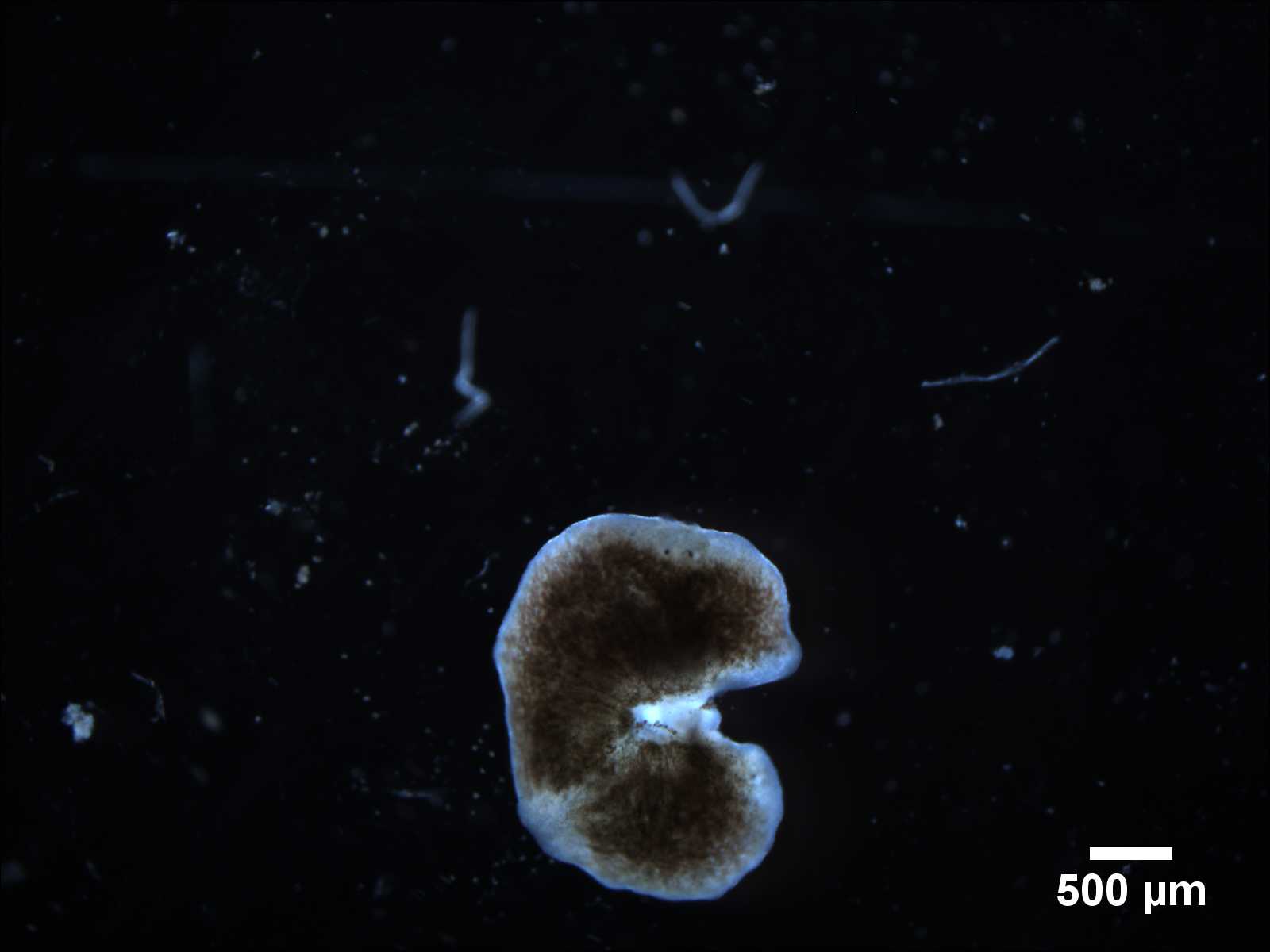

Supplement: S2 Dataset — This dataset contains raw-images of synapsin stains of uncut one- and two- headed worms, synapsin stains and brightfield images of the upwards and inverted L-cut scenarios, and synapsin stains and brightfield images showing the effects of the dynein inhibitor Ciliobrevin D on planaria regeneration. A Word document contained in the zip folder provides detailed description of the different cases. (ZIP) [file pcbi.1006904.s017.zip › DatasetS9i/L_cuts/b) downwards L-cut/brightfield pictures/Sample 5.jpg]

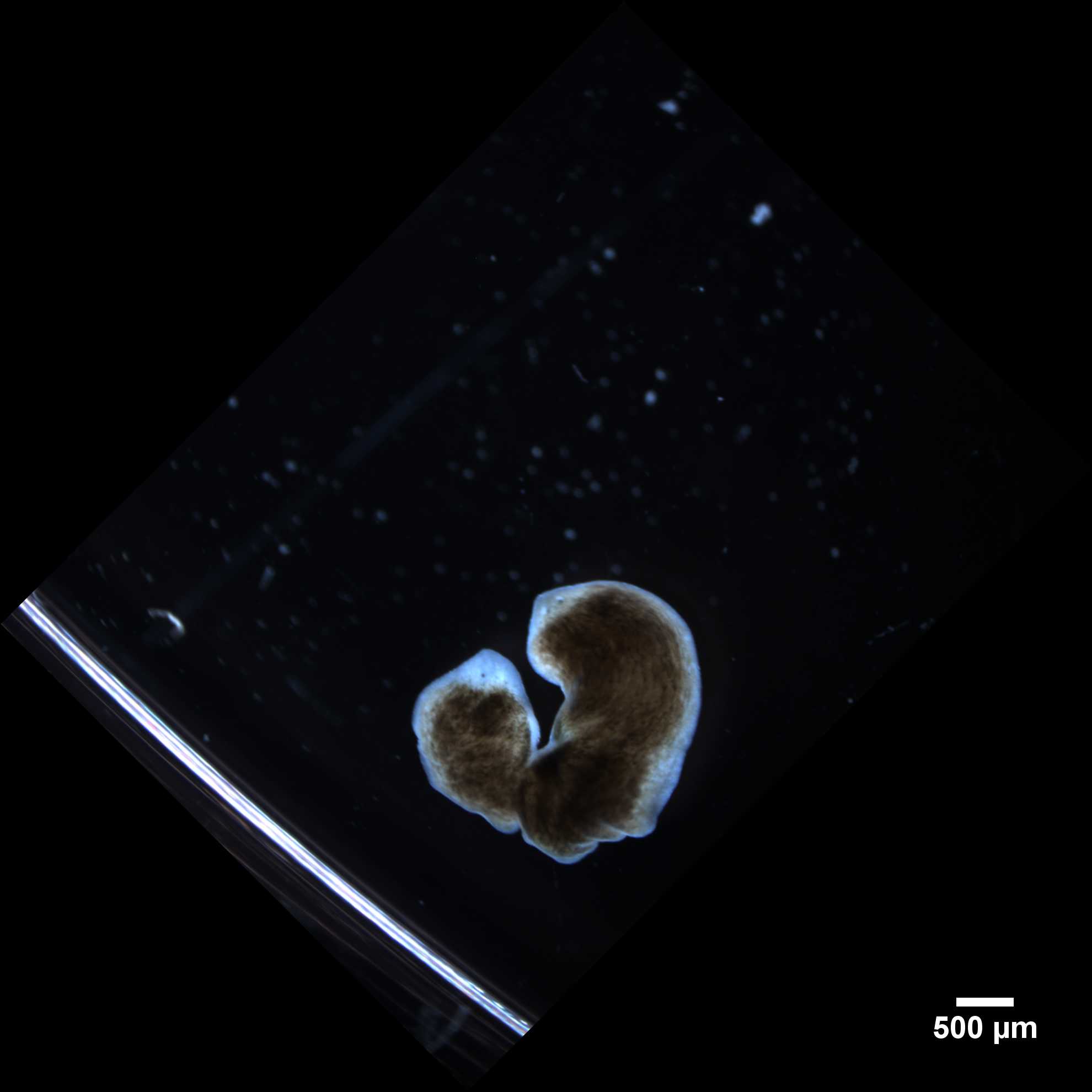

Supplement: S2 Dataset — This dataset contains raw-images of synapsin stains of uncut one- and two- headed worms, synapsin stains and brightfield images of the upwards and inverted L-cut scenarios, and synapsin stains and brightfield images showing the effects of the dynein inhibitor Ciliobrevin D on planaria regeneration. A Word document contained in the zip folder provides detailed description of the different cases. (ZIP) [file pcbi.1006904.s017.zip › DatasetS9i/L_cuts/b) downwards L-cut/brightfield pictures/Sample 6.jpg]

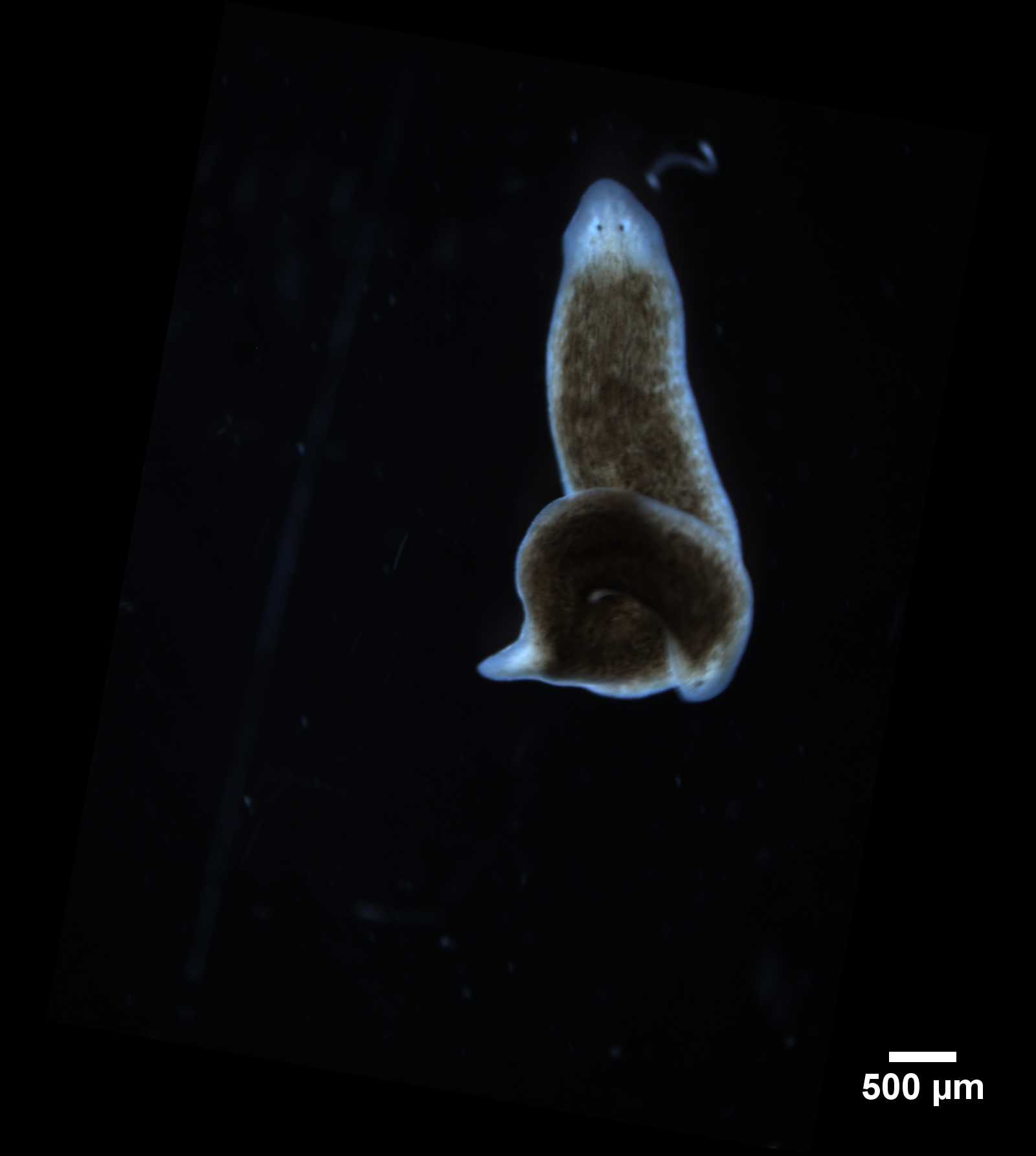

Supplement: S2 Dataset — This dataset contains raw-images of synapsin stains of uncut one- and two- headed worms, synapsin stains and brightfield images of the upwards and inverted L-cut scenarios, and synapsin stains and brightfield images showing the effects of the dynein inhibitor Ciliobrevin D on planaria regeneration. A Word document contained in the zip folder provides detailed description of the different cases. (ZIP) [file pcbi.1006904.s017.zip › DatasetS9i/L_cuts/b) downwards L-cut/brightfield pictures/Sample 7.jpg]

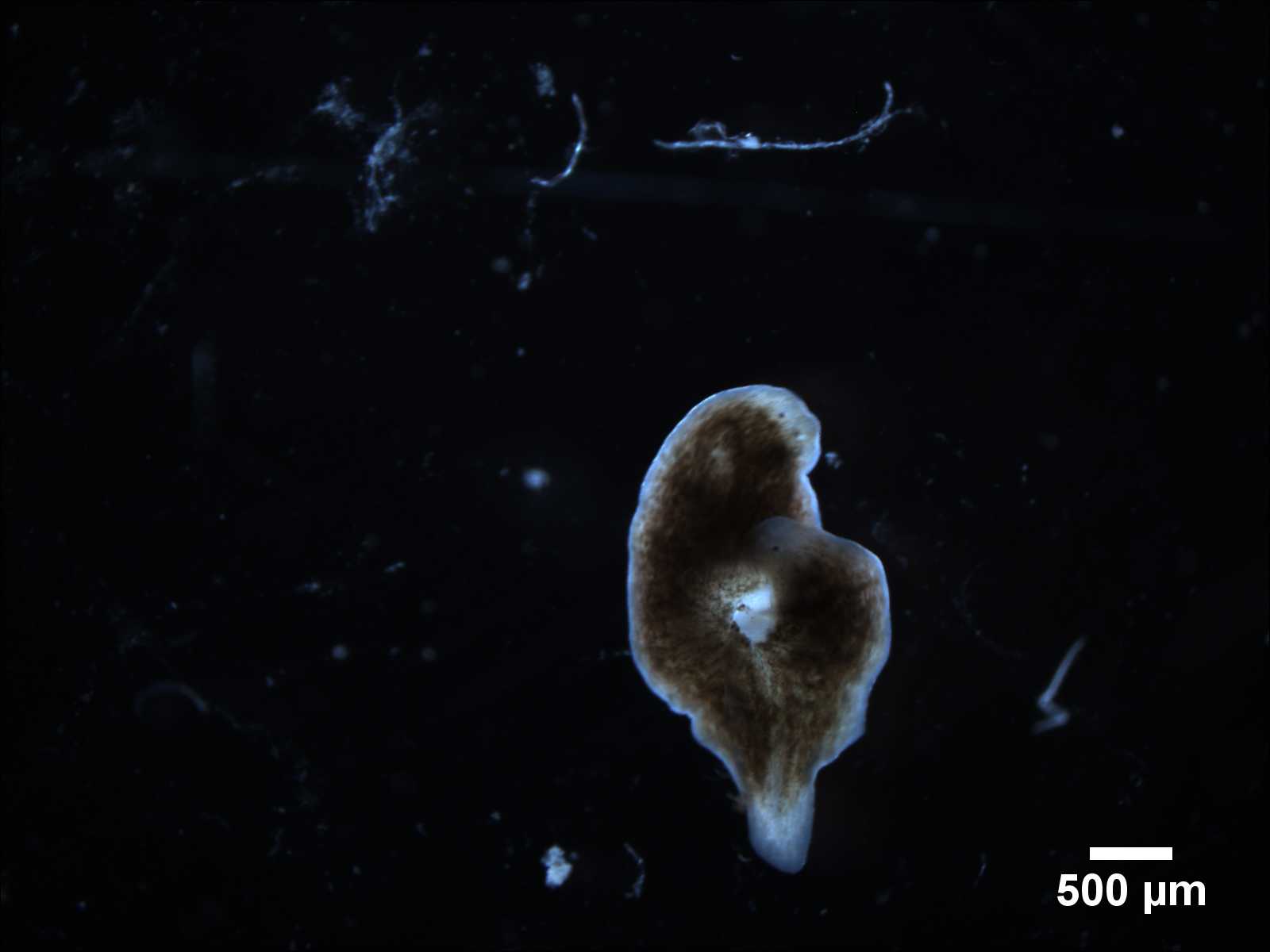

Supplement: S2 Dataset — This dataset contains raw-images of synapsin stains of uncut one- and two- headed worms, synapsin stains and brightfield images of the upwards and inverted L-cut scenarios, and synapsin stains and brightfield images showing the effects of the dynein inhibitor Ciliobrevin D on planaria regeneration. A Word document contained in the zip folder provides detailed description of the different cases. (ZIP) [file pcbi.1006904.s017.zip › DatasetS9i/L_cuts/b) downwards L-cut/brightfield pictures/Sample 8.jpg]

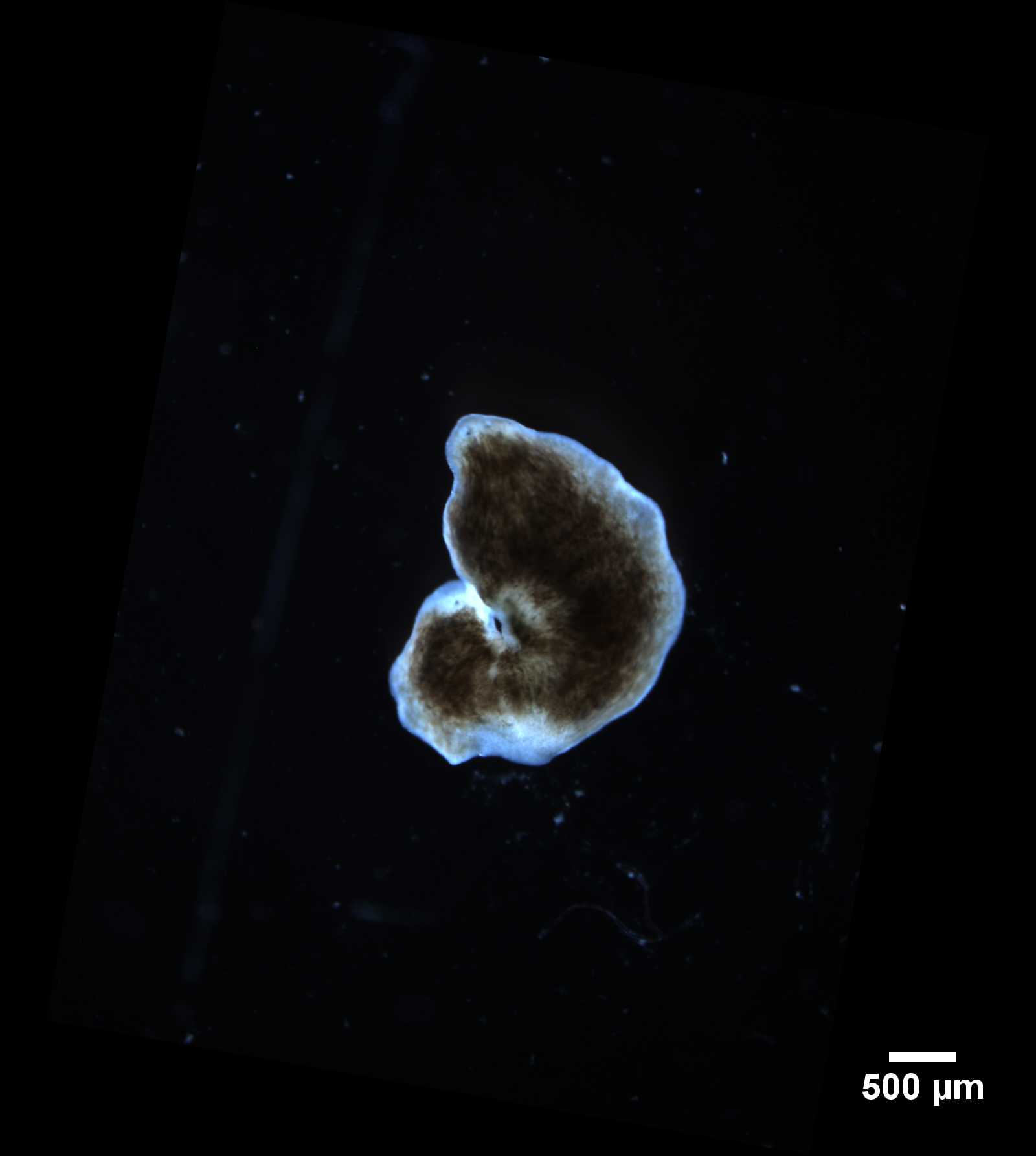

Supplement: S2 Dataset — This dataset contains raw-images of synapsin stains of uncut one- and two- headed worms, synapsin stains and brightfield images of the upwards and inverted L-cut scenarios, and synapsin stains and brightfield images showing the effects of the dynein inhibitor Ciliobrevin D on planaria regeneration. A Word document contained in the zip folder provides detailed description of the different cases. (ZIP) [file pcbi.1006904.s017.zip › DatasetS9i/L_cuts/b) downwards L-cut/brightfield pictures/Sample 9.jpg]

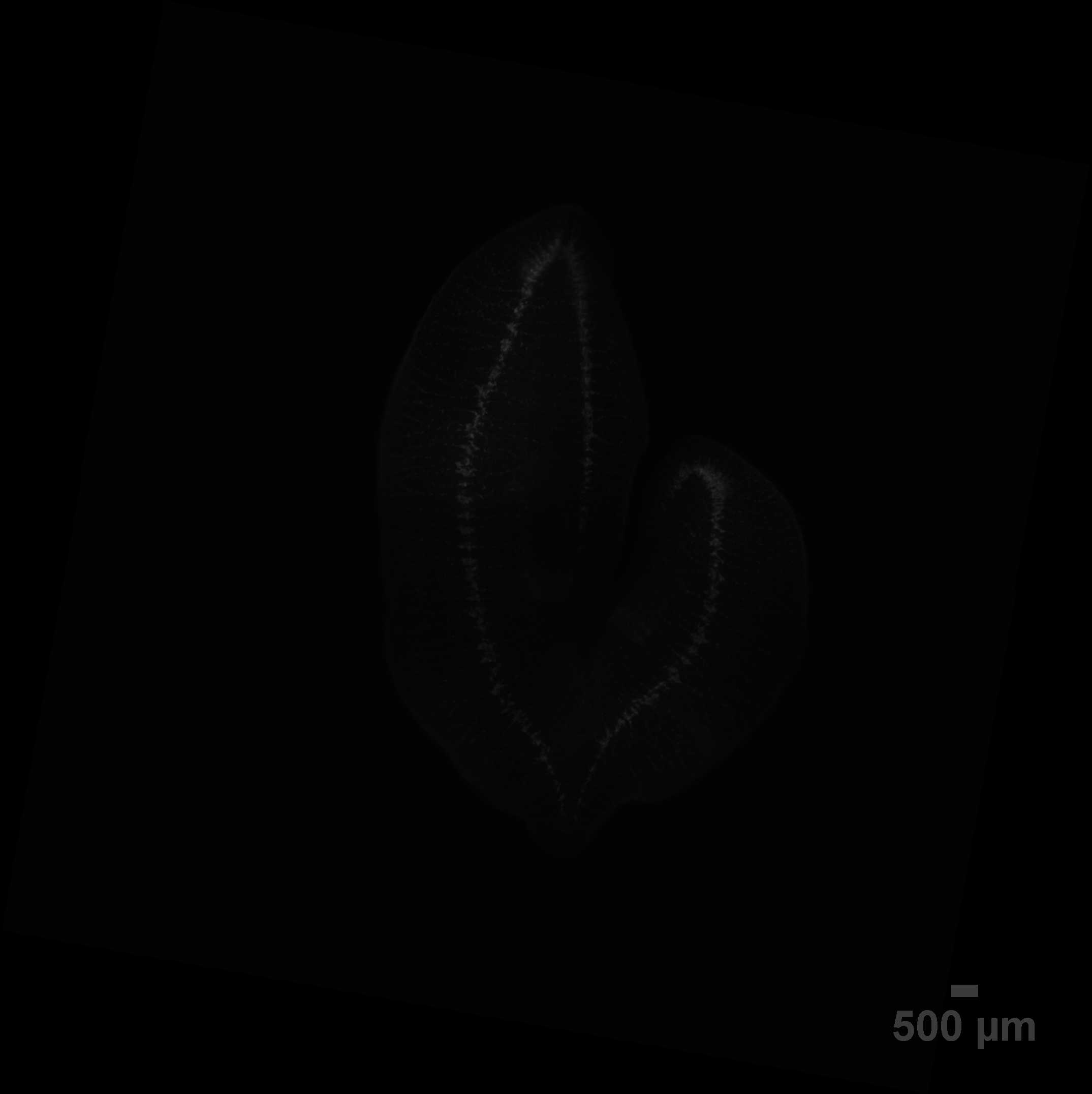

Supplement: S2 Dataset — This dataset contains raw-images of synapsin stains of uncut one- and two- headed worms, synapsin stains and brightfield images of the upwards and inverted L-cut scenarios, and synapsin stains and brightfield images showing the effects of the dynein inhibitor Ciliobrevin D on planaria regeneration. A Word document contained in the zip folder provides detailed description of the different cases. (ZIP) [file pcbi.1006904.s017.zip › DatasetS9i/L_cuts/b) downwards L-cut/synapsin stain/11 dpc_Sample 10.jpg]

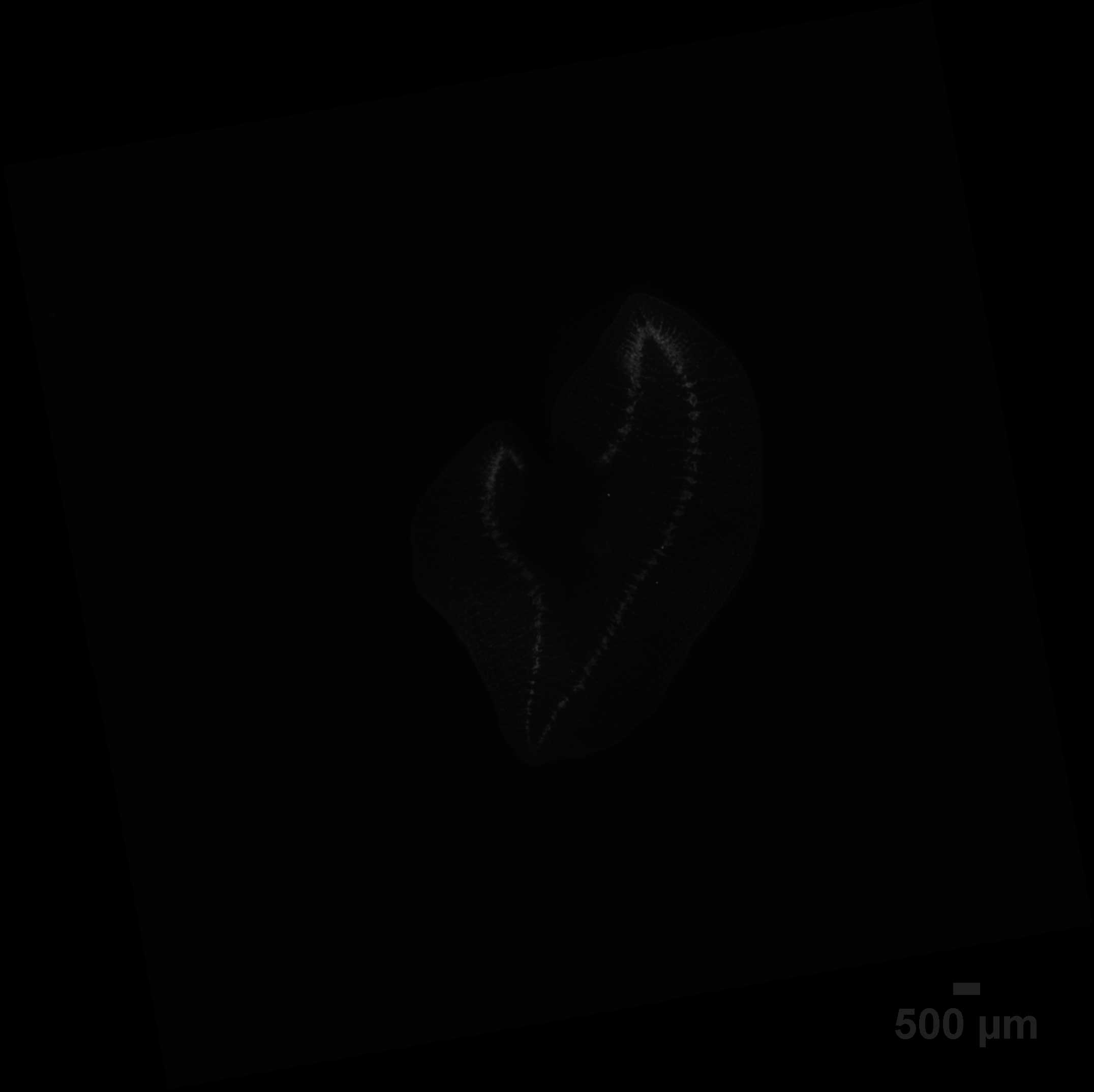

Supplement: S2 Dataset — This dataset contains raw-images of synapsin stains of uncut one- and two- headed worms, synapsin stains and brightfield images of the upwards and inverted L-cut scenarios, and synapsin stains and brightfield images showing the effects of the dynein inhibitor Ciliobrevin D on planaria regeneration. A Word document contained in the zip folder provides detailed description of the different cases. (ZIP) [file pcbi.1006904.s017.zip › DatasetS9i/L_cuts/b) downwards L-cut/synapsin stain/11 dpc_Sample 1.jpg]

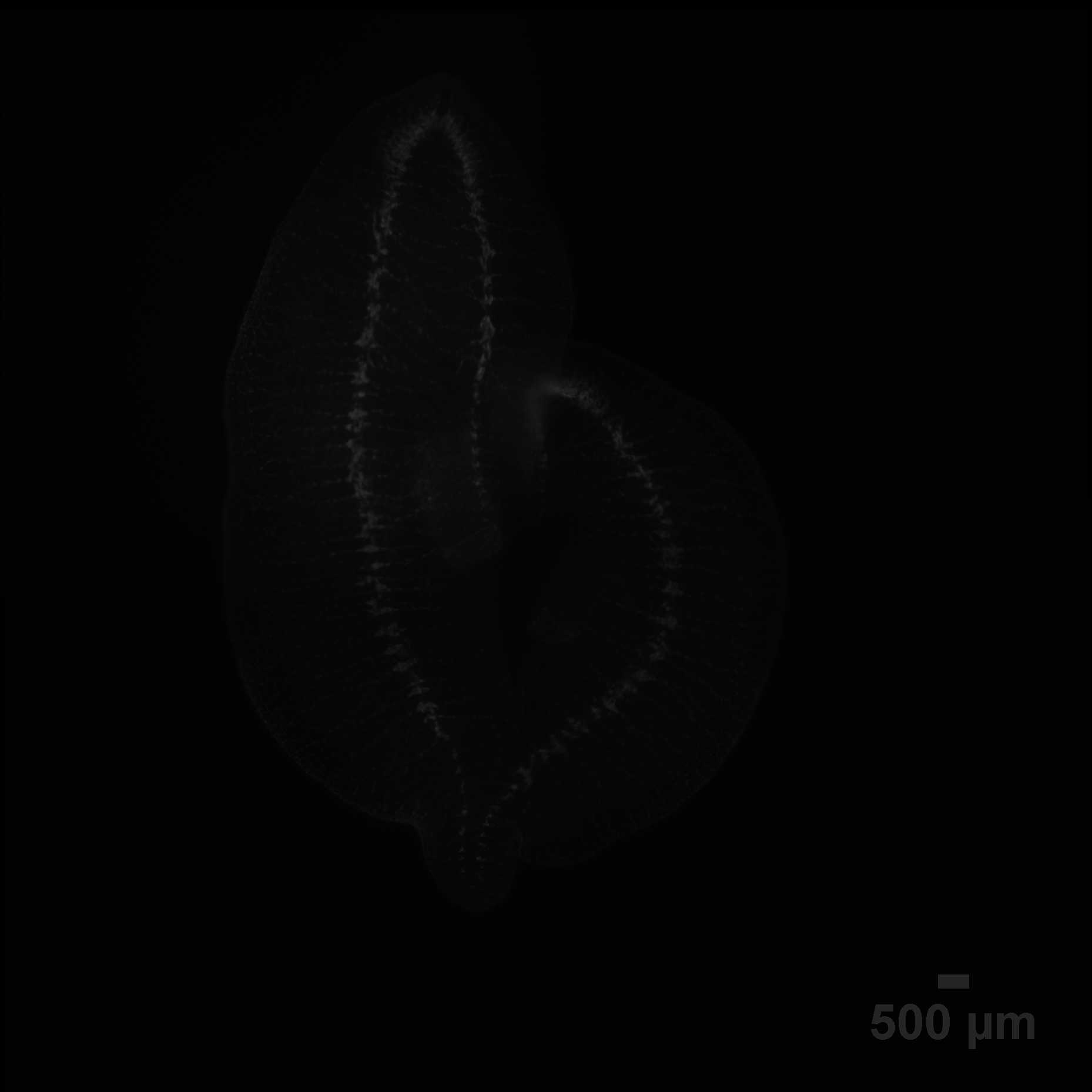

Supplement: S2 Dataset — This dataset contains raw-images of synapsin stains of uncut one- and two- headed worms, synapsin stains and brightfield images of the upwards and inverted L-cut scenarios, and synapsin stains and brightfield images showing the effects of the dynein inhibitor Ciliobrevin D on planaria regeneration. A Word document contained in the zip folder provides detailed description of the different cases. (ZIP) [file pcbi.1006904.s017.zip › DatasetS9i/L_cuts/b) downwards L-cut/synapsin stain/11 dpc_Sample 2.jpg]

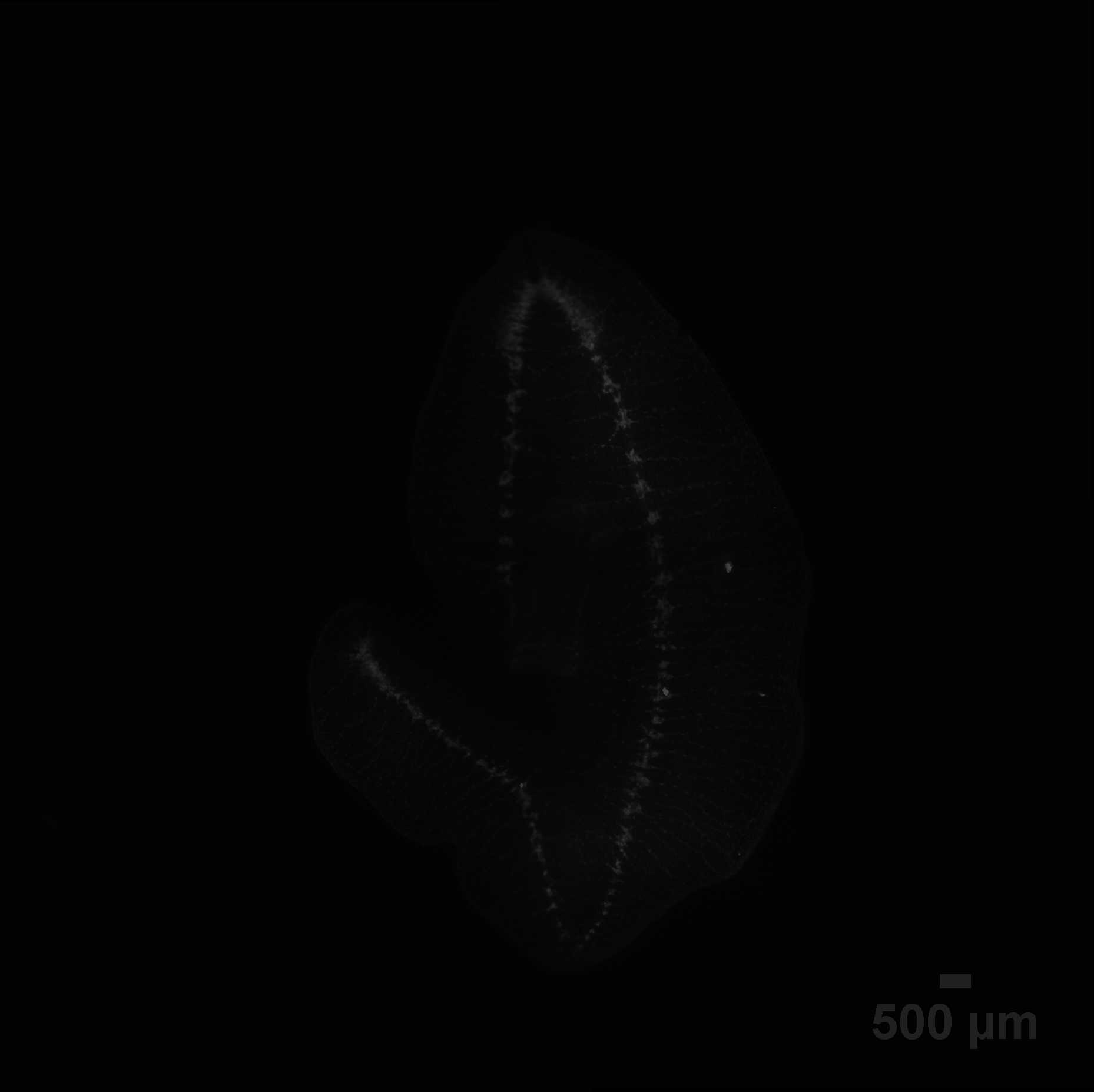

Supplement: S2 Dataset — This dataset contains raw-images of synapsin stains of uncut one- and two- headed worms, synapsin stains and brightfield images of the upwards and inverted L-cut scenarios, and synapsin stains and brightfield images showing the effects of the dynein inhibitor Ciliobrevin D on planaria regeneration. A Word document contained in the zip folder provides detailed description of the different cases. (ZIP) [file pcbi.1006904.s017.zip › DatasetS9i/L_cuts/b) downwards L-cut/synapsin stain/11 dpc_Sample 3.jpg]

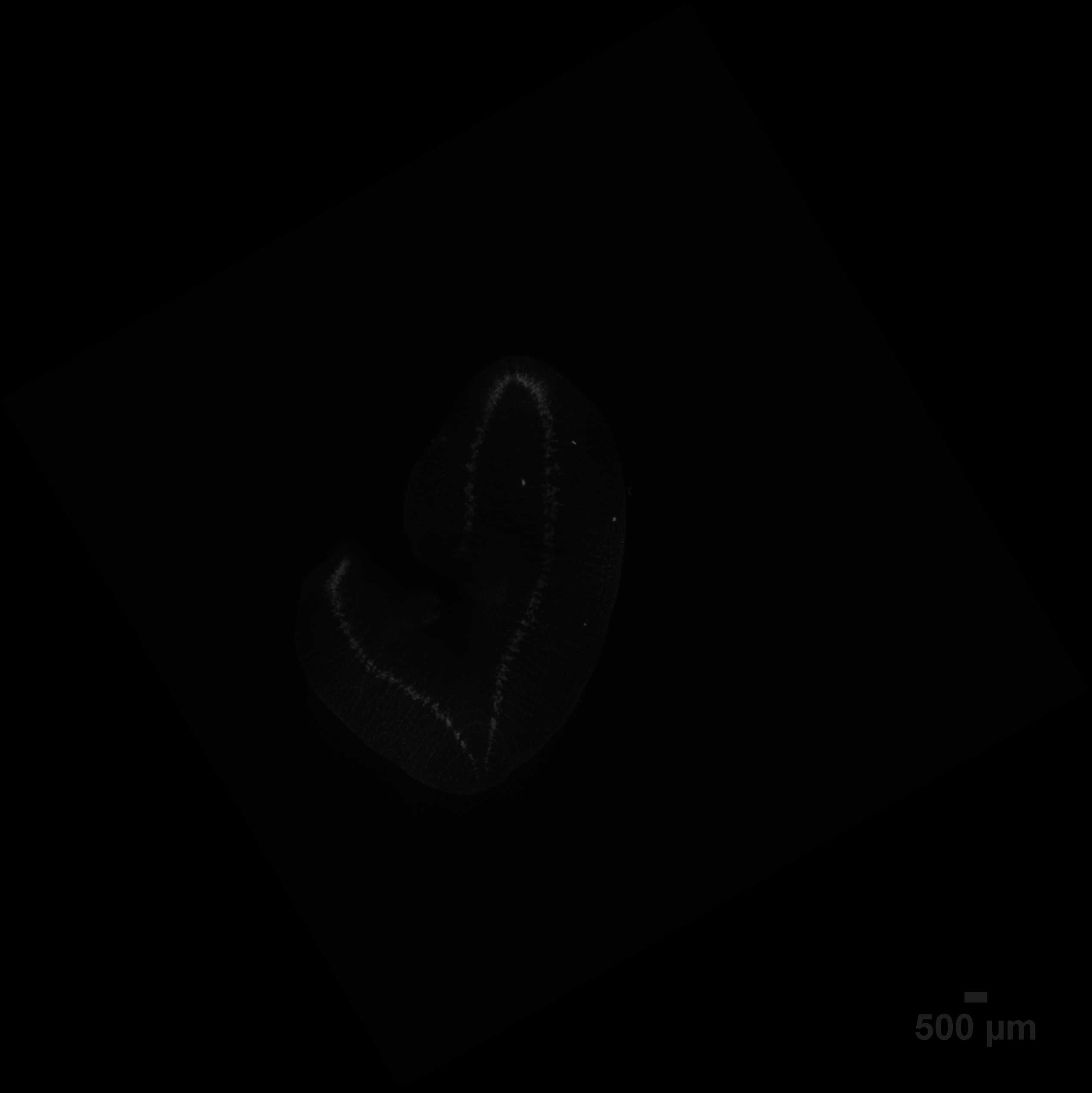

Supplement: S2 Dataset — This dataset contains raw-images of synapsin stains of uncut one- and two- headed worms, synapsin stains and brightfield images of the upwards and inverted L-cut scenarios, and synapsin stains and brightfield images showing the effects of the dynein inhibitor Ciliobrevin D on planaria regeneration. A Word document contained in the zip folder provides detailed description of the different cases. (ZIP) [file pcbi.1006904.s017.zip › DatasetS9i/L_cuts/b) downwards L-cut/synapsin stain/11 dpc_Sample 4.jpg]

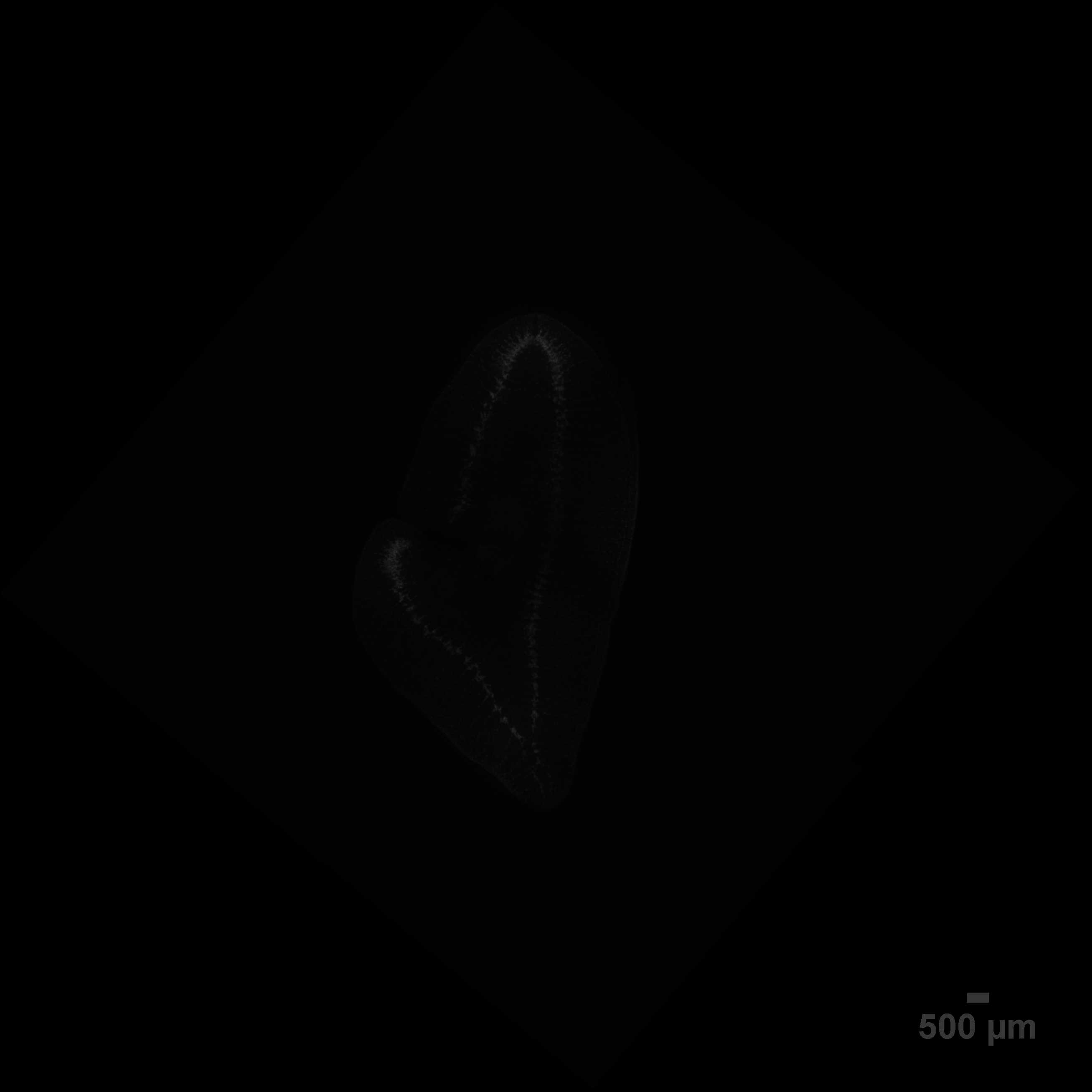

Supplement: S2 Dataset — This dataset contains raw-images of synapsin stains of uncut one- and two- headed worms, synapsin stains and brightfield images of the upwards and inverted L-cut scenarios, and synapsin stains and brightfield images showing the effects of the dynein inhibitor Ciliobrevin D on planaria regeneration. A Word document contained in the zip folder provides detailed description of the different cases. (ZIP) [file pcbi.1006904.s017.zip › DatasetS9i/L_cuts/b) downwards L-cut/synapsin stain/11 dpc_Sample 5.jpg]

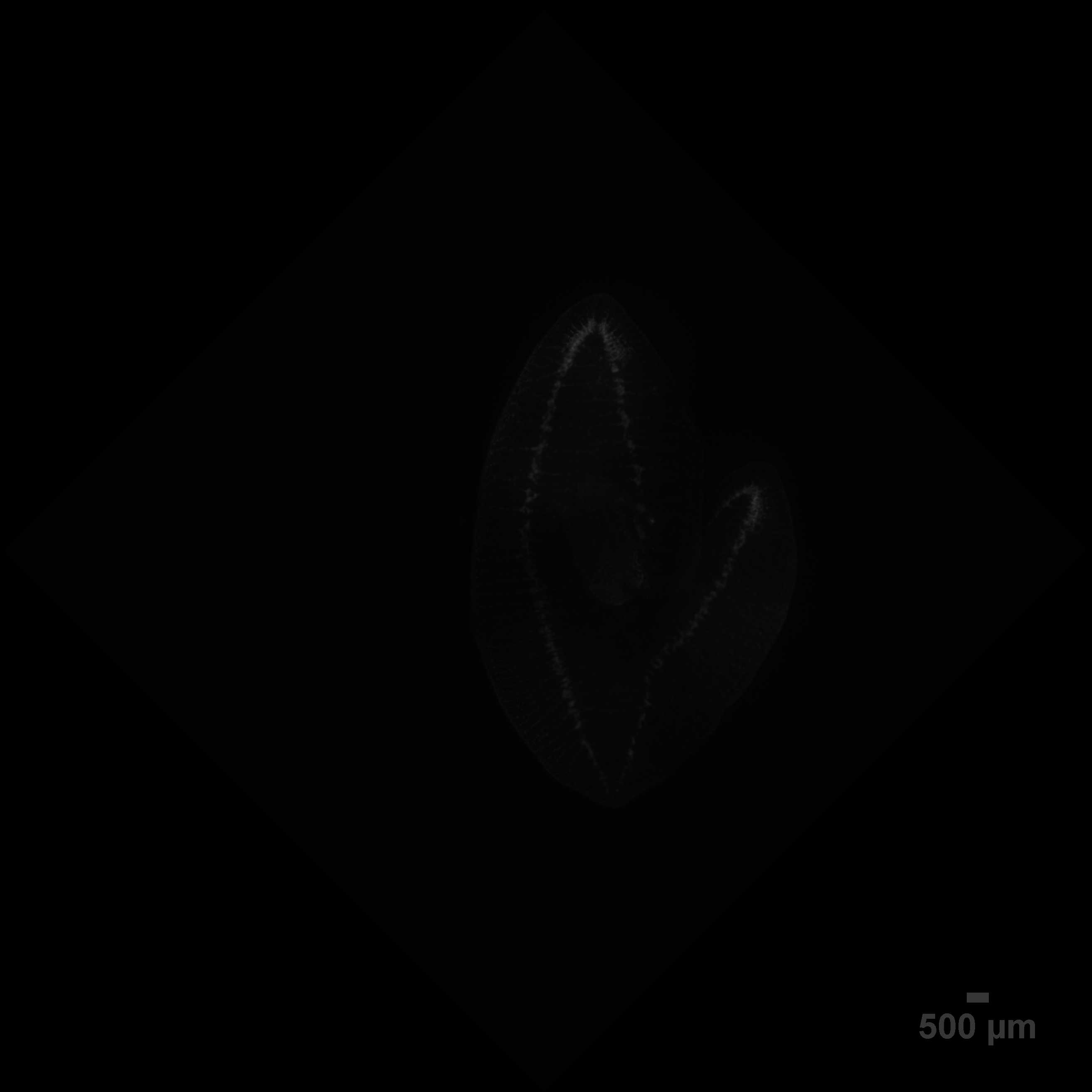

Supplement: S2 Dataset — This dataset contains raw-images of synapsin stains of uncut one- and two- headed worms, synapsin stains and brightfield images of the upwards and inverted L-cut scenarios, and synapsin stains and brightfield images showing the effects of the dynein inhibitor Ciliobrevin D on planaria regeneration. A Word document contained in the zip folder provides detailed description of the different cases. (ZIP) [file pcbi.1006904.s017.zip › DatasetS9i/L_cuts/b) downwards L-cut/synapsin stain/11 dpc_Sample 6.jpg]

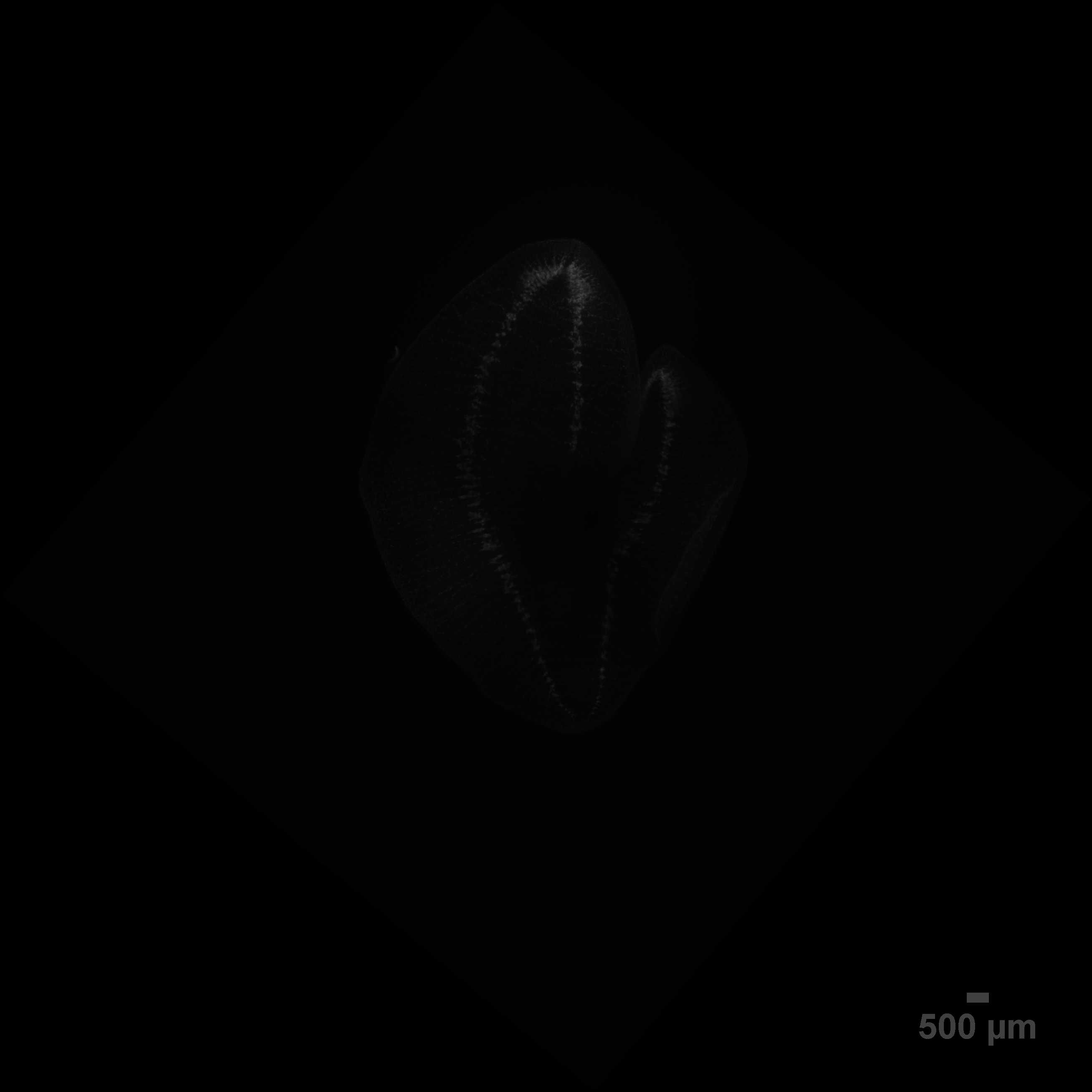

Supplement: S2 Dataset — This dataset contains raw-images of synapsin stains of uncut one- and two- headed worms, synapsin stains and brightfield images of the upwards and inverted L-cut scenarios, and synapsin stains and brightfield images showing the effects of the dynein inhibitor Ciliobrevin D on planaria regeneration. A Word document contained in the zip folder provides detailed description of the different cases. (ZIP) [file pcbi.1006904.s017.zip › DatasetS9i/L_cuts/b) downwards L-cut/synapsin stain/11 dpc_Sample 7.jpg]

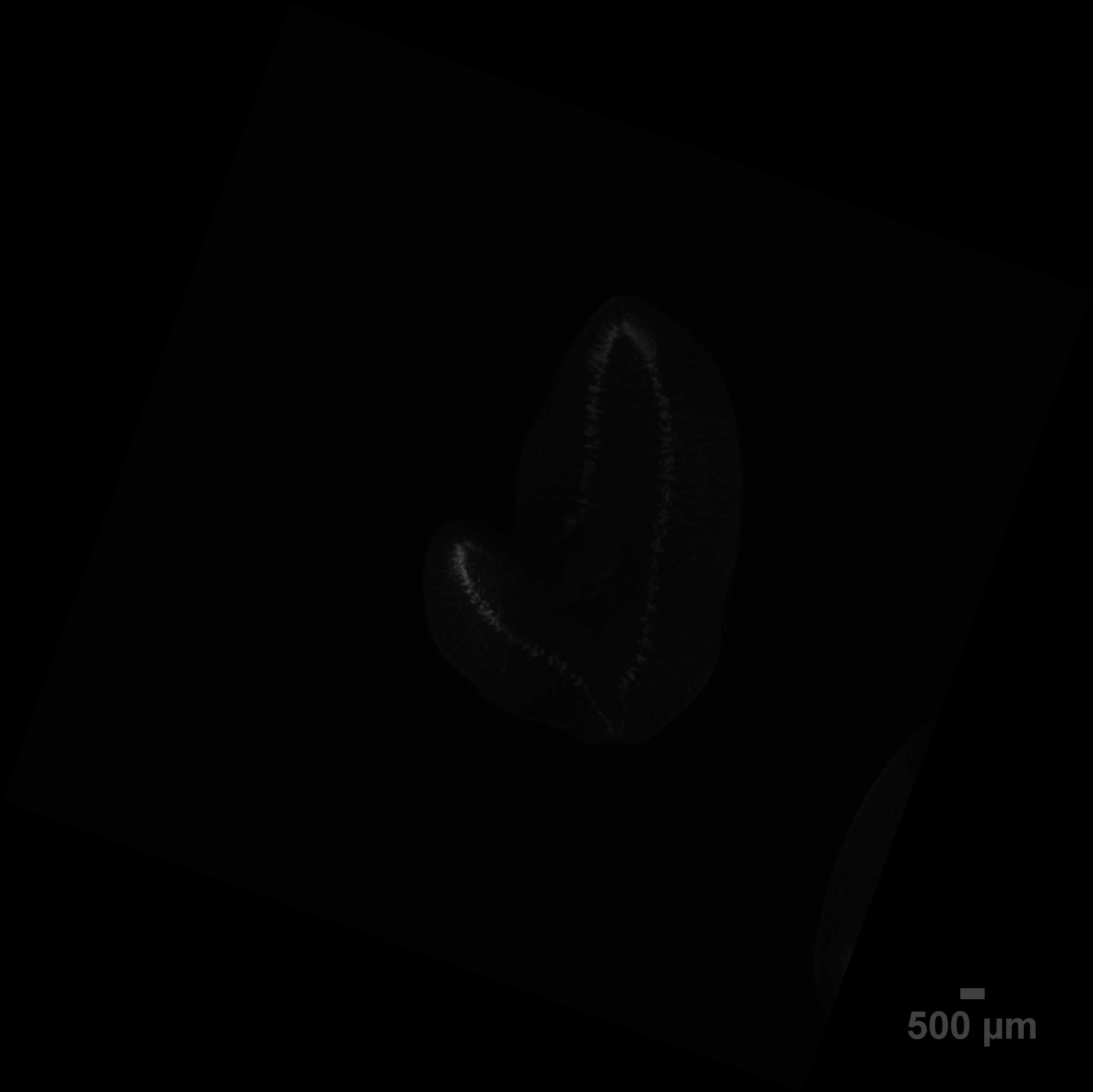

Supplement: S2 Dataset — This dataset contains raw-images of synapsin stains of uncut one- and two- headed worms, synapsin stains and brightfield images of the upwards and inverted L-cut scenarios, and synapsin stains and brightfield images showing the effects of the dynein inhibitor Ciliobrevin D on planaria regeneration. A Word document contained in the zip folder provides detailed description of the different cases. (ZIP) [file pcbi.1006904.s017.zip › DatasetS9i/L_cuts/b) downwards L-cut/synapsin stain/11 dpc_Sample 8.jpg]

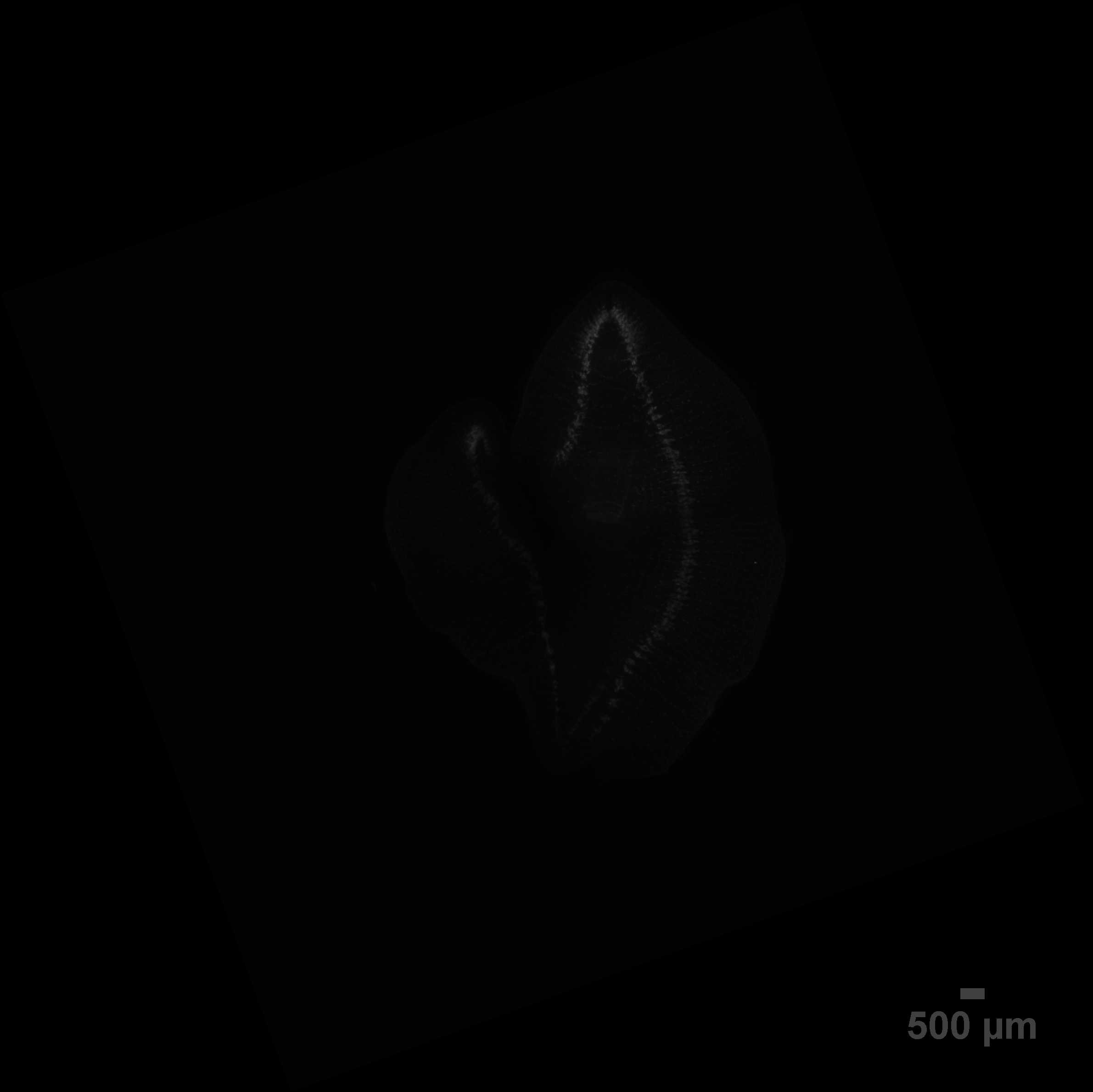

Supplement: S2 Dataset — This dataset contains raw-images of synapsin stains of uncut one- and two- headed worms, synapsin stains and brightfield images of the upwards and inverted L-cut scenarios, and synapsin stains and brightfield images showing the effects of the dynein inhibitor Ciliobrevin D on planaria regeneration. A Word document contained in the zip folder provides detailed description of the different cases. (ZIP) [file pcbi.1006904.s017.zip › DatasetS9i/L_cuts/b) downwards L-cut/synapsin stain/11 dpc_Sample 9.jpg]

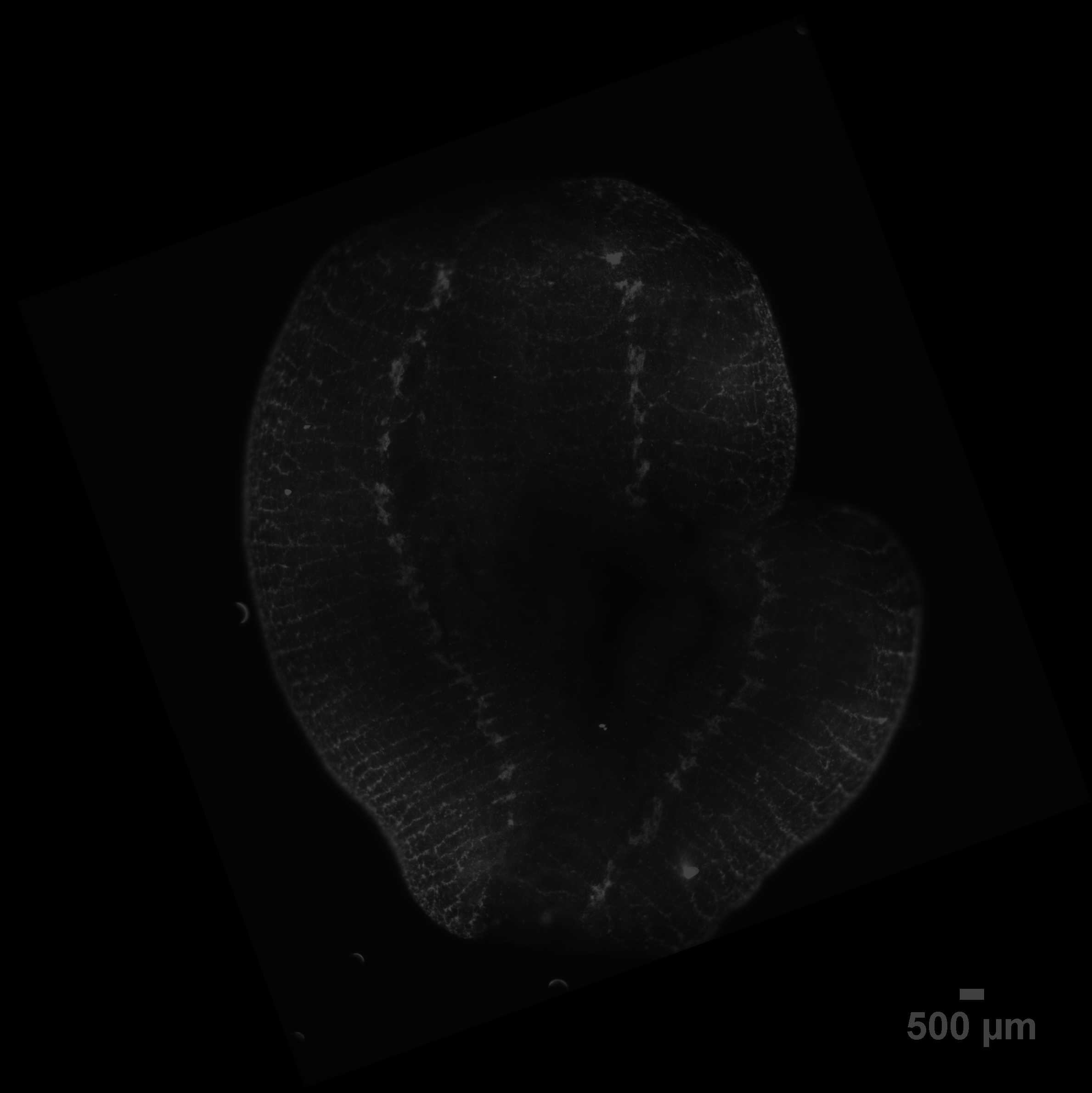

Supplement: S2 Dataset — This dataset contains raw-images of synapsin stains of uncut one- and two- headed worms, synapsin stains and brightfield images of the upwards and inverted L-cut scenarios, and synapsin stains and brightfield images showing the effects of the dynein inhibitor Ciliobrevin D on planaria regeneration. A Word document contained in the zip folder provides detailed description of the different cases. (ZIP) [file pcbi.1006904.s017.zip › DatasetS9i/L_cuts/b) downwards L-cut/synapsin stain/3 dpc_Sample 1.jpg]

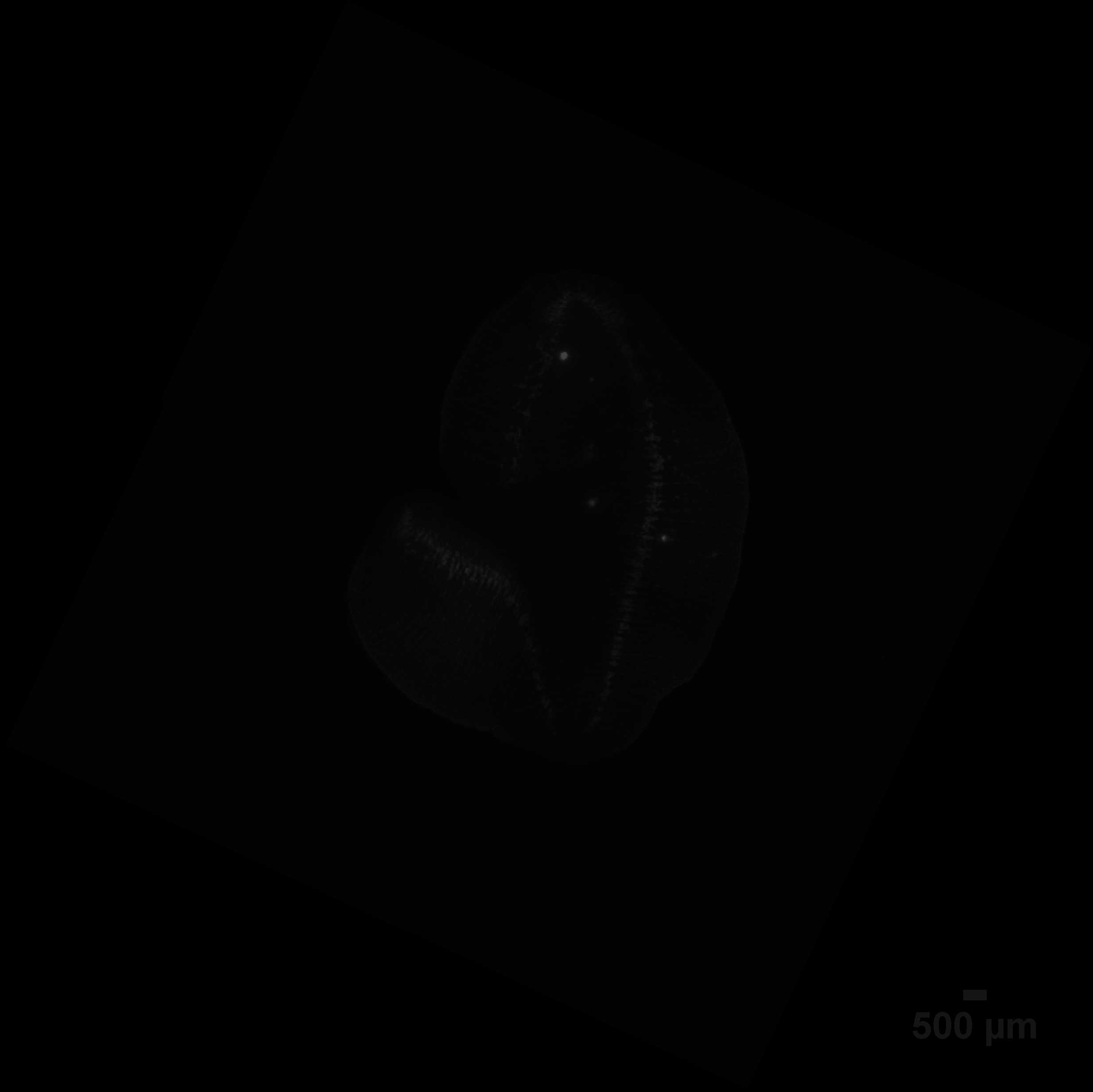

Supplement: S2 Dataset — This dataset contains raw-images of synapsin stains of uncut one- and two- headed worms, synapsin stains and brightfield images of the upwards and inverted L-cut scenarios, and synapsin stains and brightfield images showing the effects of the dynein inhibitor Ciliobrevin D on planaria regeneration. A Word document contained in the zip folder provides detailed description of the different cases. (ZIP) [file pcbi.1006904.s017.zip › DatasetS9i/L_cuts/b) downwards L-cut/synapsin stain/5 dpc_Sample 1.jpg]

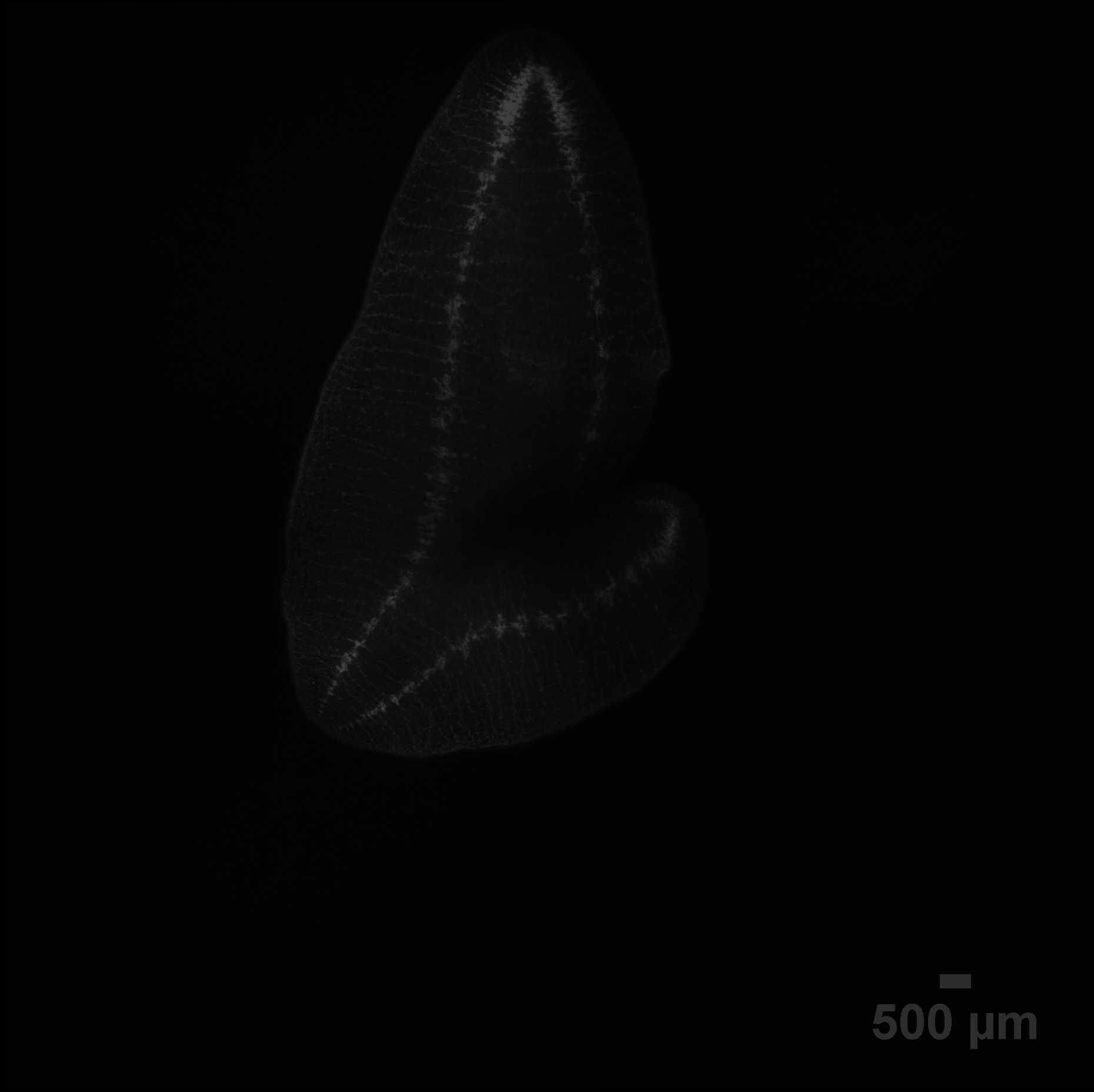

Supplement: S2 Dataset — This dataset contains raw-images of synapsin stains of uncut one- and two- headed worms, synapsin stains and brightfield images of the upwards and inverted L-cut scenarios, and synapsin stains and brightfield images showing the effects of the dynein inhibitor Ciliobrevin D on planaria regeneration. A Word document contained in the zip folder provides detailed description of the different cases. (ZIP) [file pcbi.1006904.s017.zip › DatasetS9i/L_cuts/b) downwards L-cut/synapsin stain/7 dpc_Sample 1.jpg]

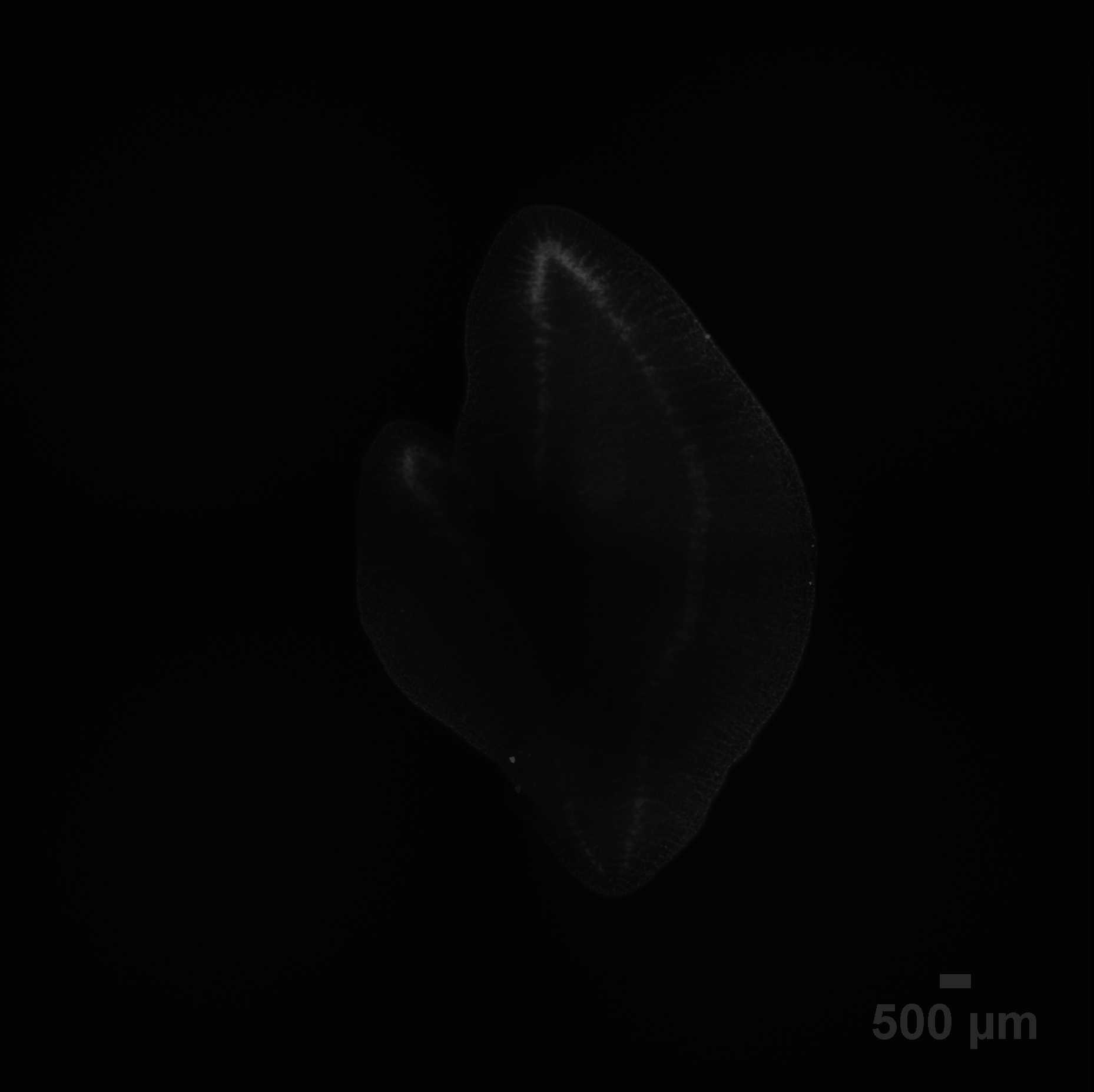

Supplement: S2 Dataset — This dataset contains raw-images of synapsin stains of uncut one- and two- headed worms, synapsin stains and brightfield images of the upwards and inverted L-cut scenarios, and synapsin stains and brightfield images showing the effects of the dynein inhibitor Ciliobrevin D on planaria regeneration. A Word document contained in the zip folder provides detailed description of the different cases. (ZIP) [file pcbi.1006904.s017.zip › DatasetS9i/L_cuts/b) downwards L-cut/synapsin stain/7 dpc_Sample 2.jpg]

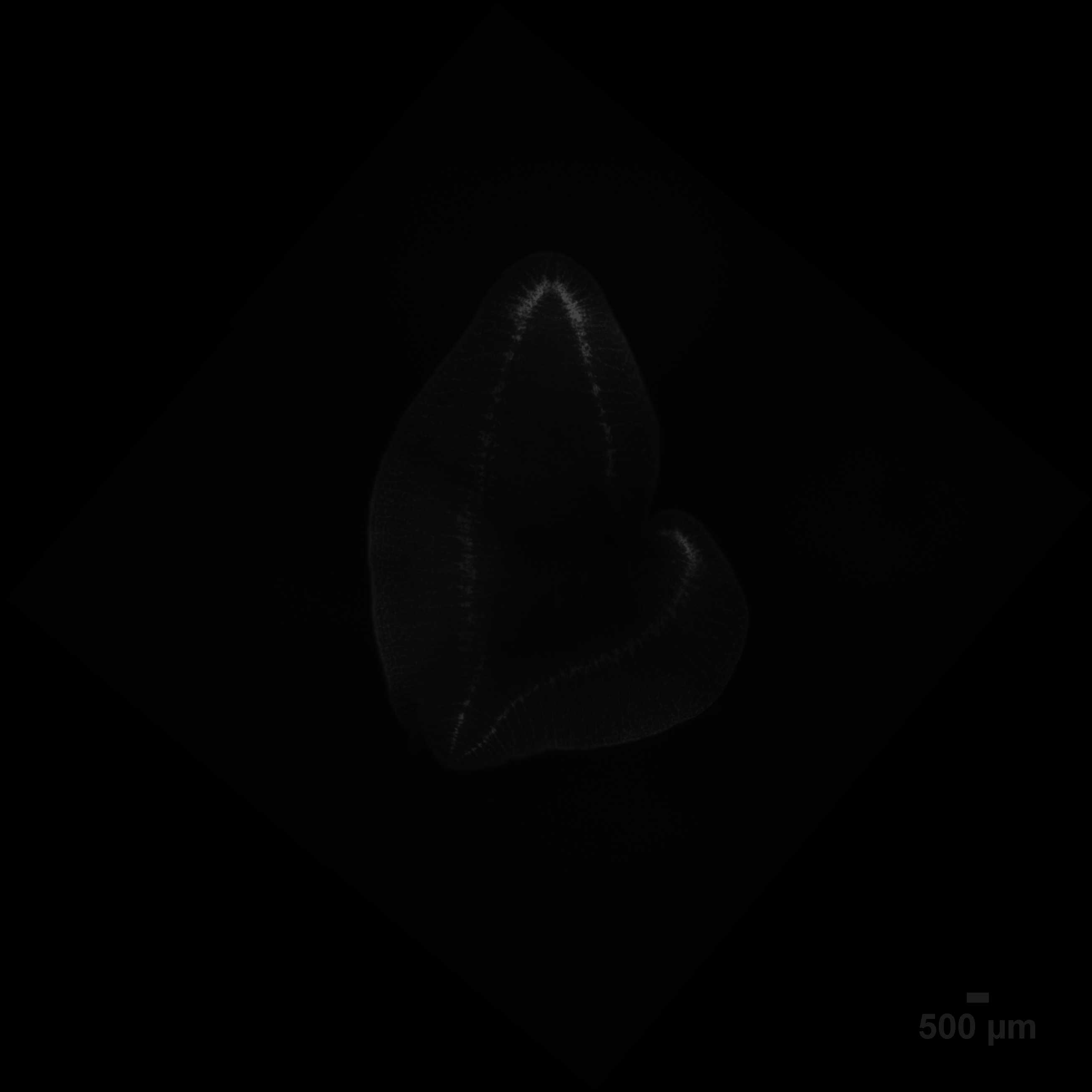

Supplement: S2 Dataset — This dataset contains raw-images of synapsin stains of uncut one- and two- headed worms, synapsin stains and brightfield images of the upwards and inverted L-cut scenarios, and synapsin stains and brightfield images showing the effects of the dynein inhibitor Ciliobrevin D on planaria regeneration. A Word document contained in the zip folder provides detailed description of the different cases. (ZIP) [file pcbi.1006904.s017.zip › DatasetS9i/L_cuts/b) downwards L-cut/synapsin stain/7 dpc_Sample 3.jpg]

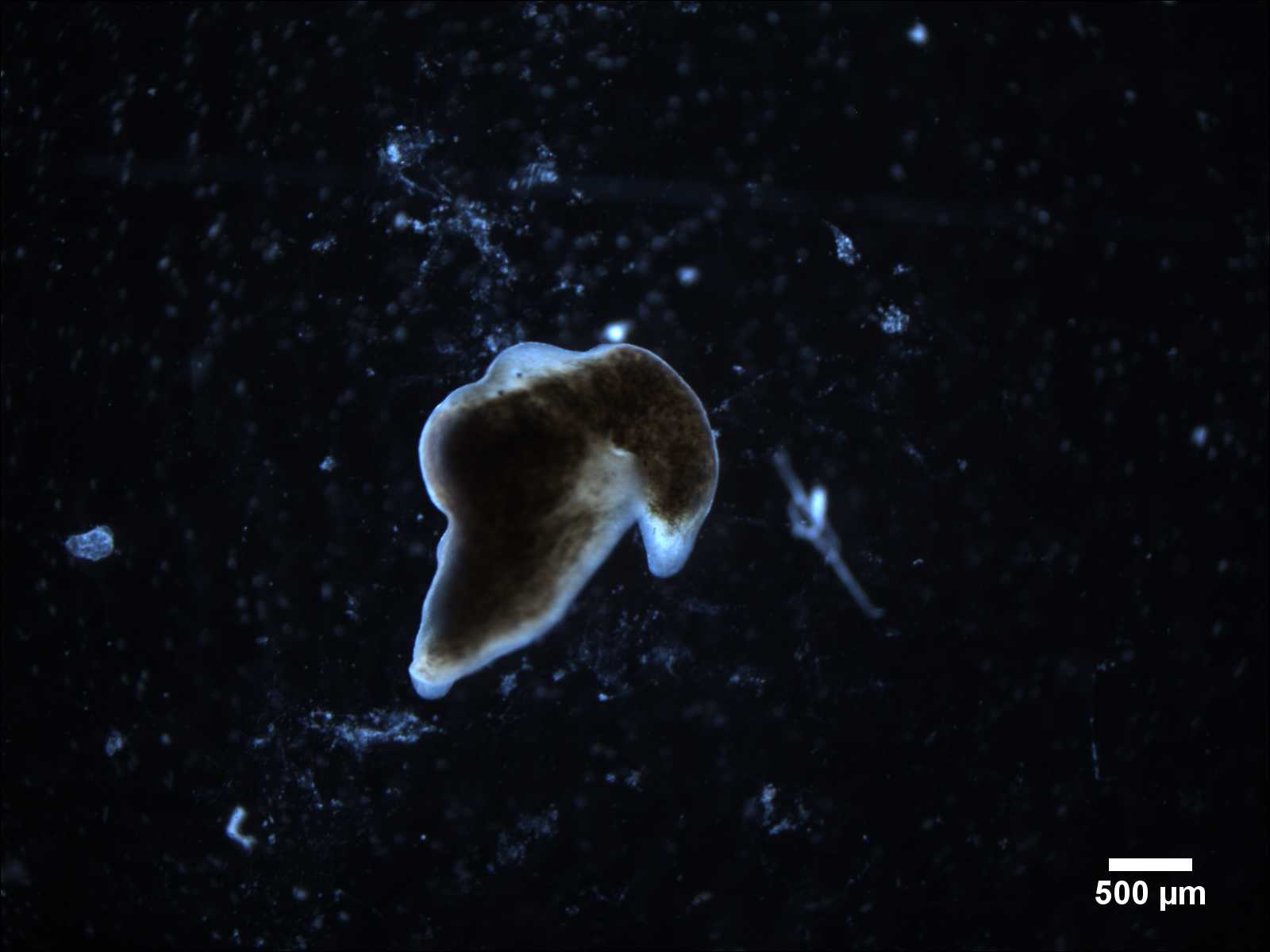

Supplement: S2 Dataset — This dataset contains raw-images of synapsin stains of uncut one- and two- headed worms, synapsin stains and brightfield images of the upwards and inverted L-cut scenarios, and synapsin stains and brightfield images showing the effects of the dynein inhibitor Ciliobrevin D on planaria regeneration. A Word document contained in the zip folder provides detailed description of the different cases. (ZIP) [file pcbi.1006904.s017.zip › DatasetS9i/L_cuts/c) upwards L-cut/brightfield pictures/Sample 10.jpg]

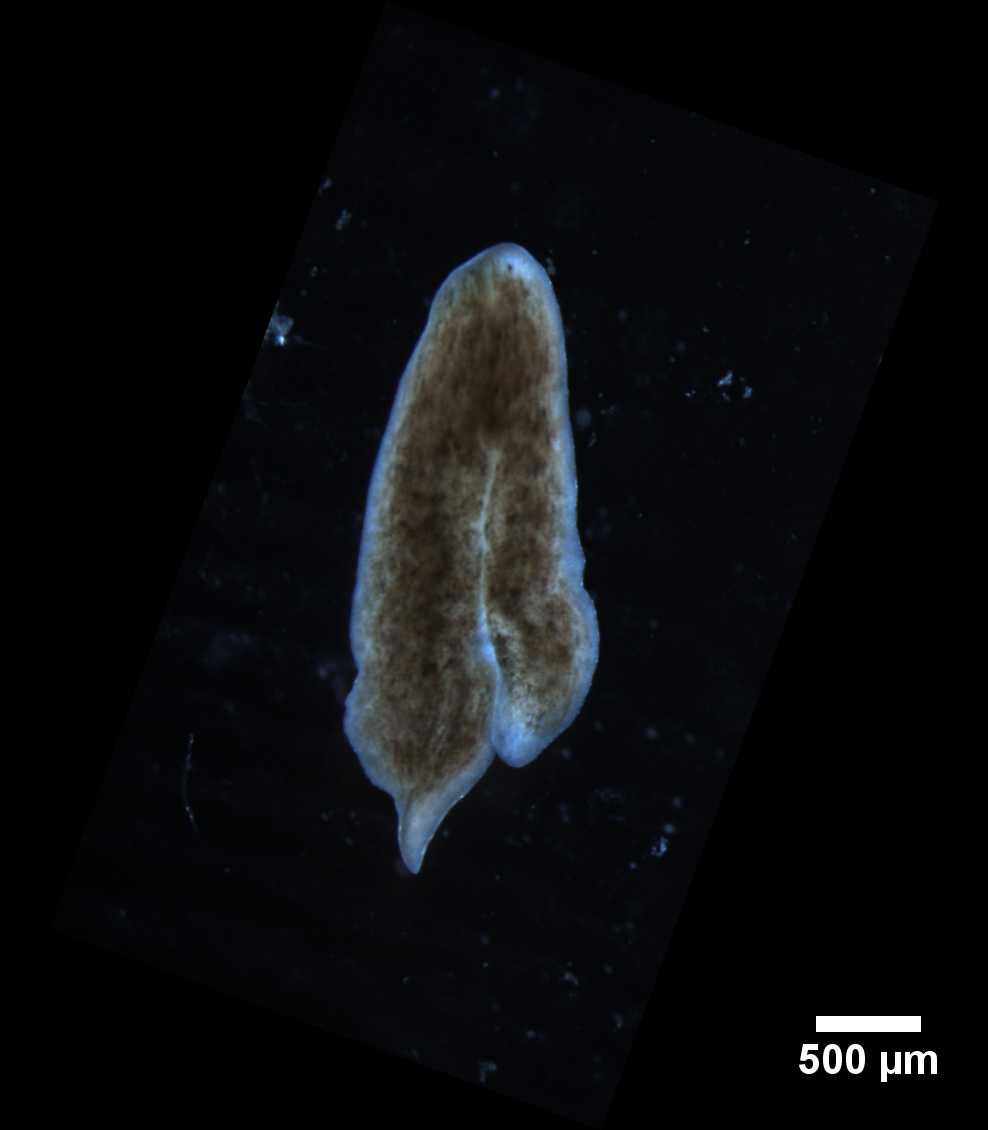

Supplement: S2 Dataset — This dataset contains raw-images of synapsin stains of uncut one- and two- headed worms, synapsin stains and brightfield images of the upwards and inverted L-cut scenarios, and synapsin stains and brightfield images showing the effects of the dynein inhibitor Ciliobrevin D on planaria regeneration. A Word document contained in the zip folder provides detailed description of the different cases. (ZIP) [file pcbi.1006904.s017.zip › DatasetS9i/L_cuts/c) upwards L-cut/brightfield pictures/Sample 11.jpg]

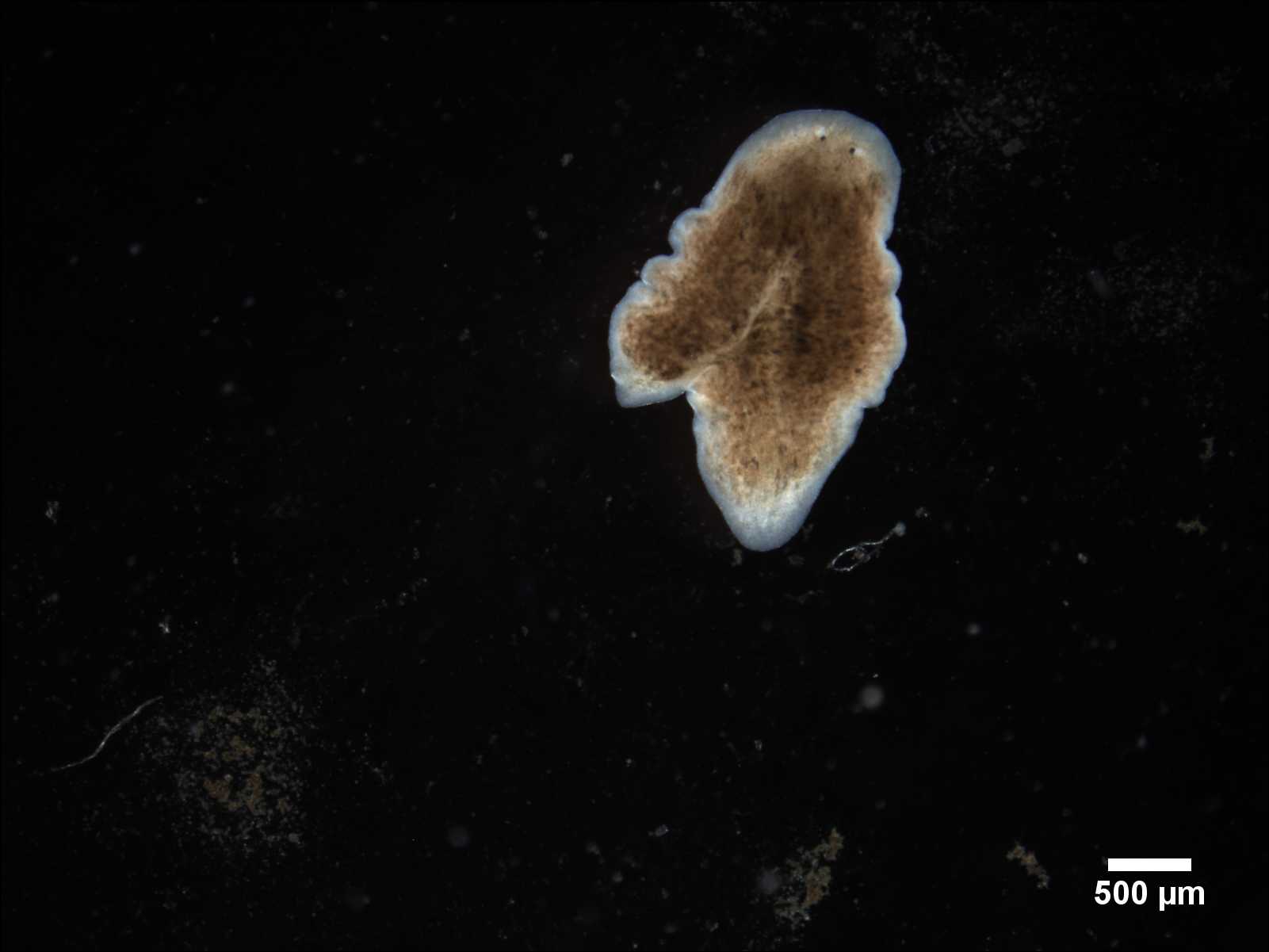

Supplement: S2 Dataset — This dataset contains raw-images of synapsin stains of uncut one- and two- headed worms, synapsin stains and brightfield images of the upwards and inverted L-cut scenarios, and synapsin stains and brightfield images showing the effects of the dynein inhibitor Ciliobrevin D on planaria regeneration. A Word document contained in the zip folder provides detailed description of the different cases. (ZIP) [file pcbi.1006904.s017.zip › DatasetS9i/L_cuts/c) upwards L-cut/brightfield pictures/Sample 12.jpg]

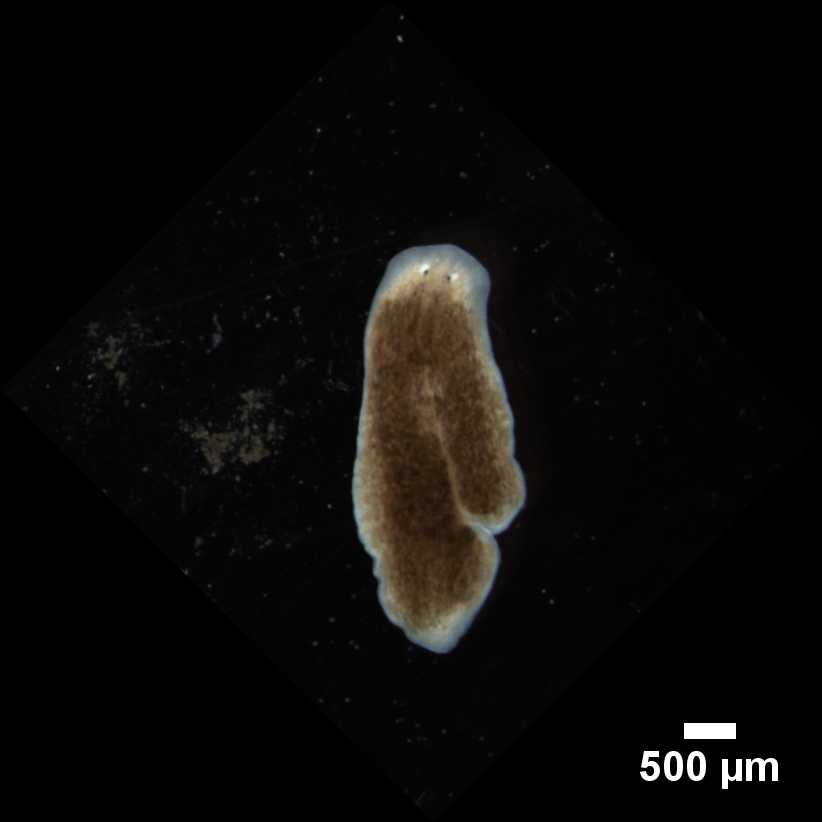

Supplement: S2 Dataset — This dataset contains raw-images of synapsin stains of uncut one- and two- headed worms, synapsin stains and brightfield images of the upwards and inverted L-cut scenarios, and synapsin stains and brightfield images showing the effects of the dynein inhibitor Ciliobrevin D on planaria regeneration. A Word document contained in the zip folder provides detailed description of the different cases. (ZIP) [file pcbi.1006904.s017.zip › DatasetS9i/L_cuts/c) upwards L-cut/brightfield pictures/Sample 1.jpg]

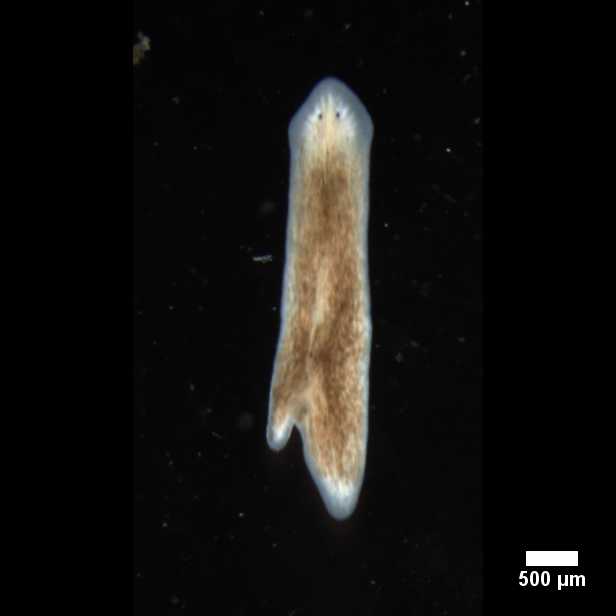

Supplement: S2 Dataset — This dataset contains raw-images of synapsin stains of uncut one- and two- headed worms, synapsin stains and brightfield images of the upwards and inverted L-cut scenarios, and synapsin stains and brightfield images showing the effects of the dynein inhibitor Ciliobrevin D on planaria regeneration. A Word document contained in the zip folder provides detailed description of the different cases. (ZIP) [file pcbi.1006904.s017.zip › DatasetS9i/L_cuts/c) upwards L-cut/brightfield pictures/Sample 2.jpg]

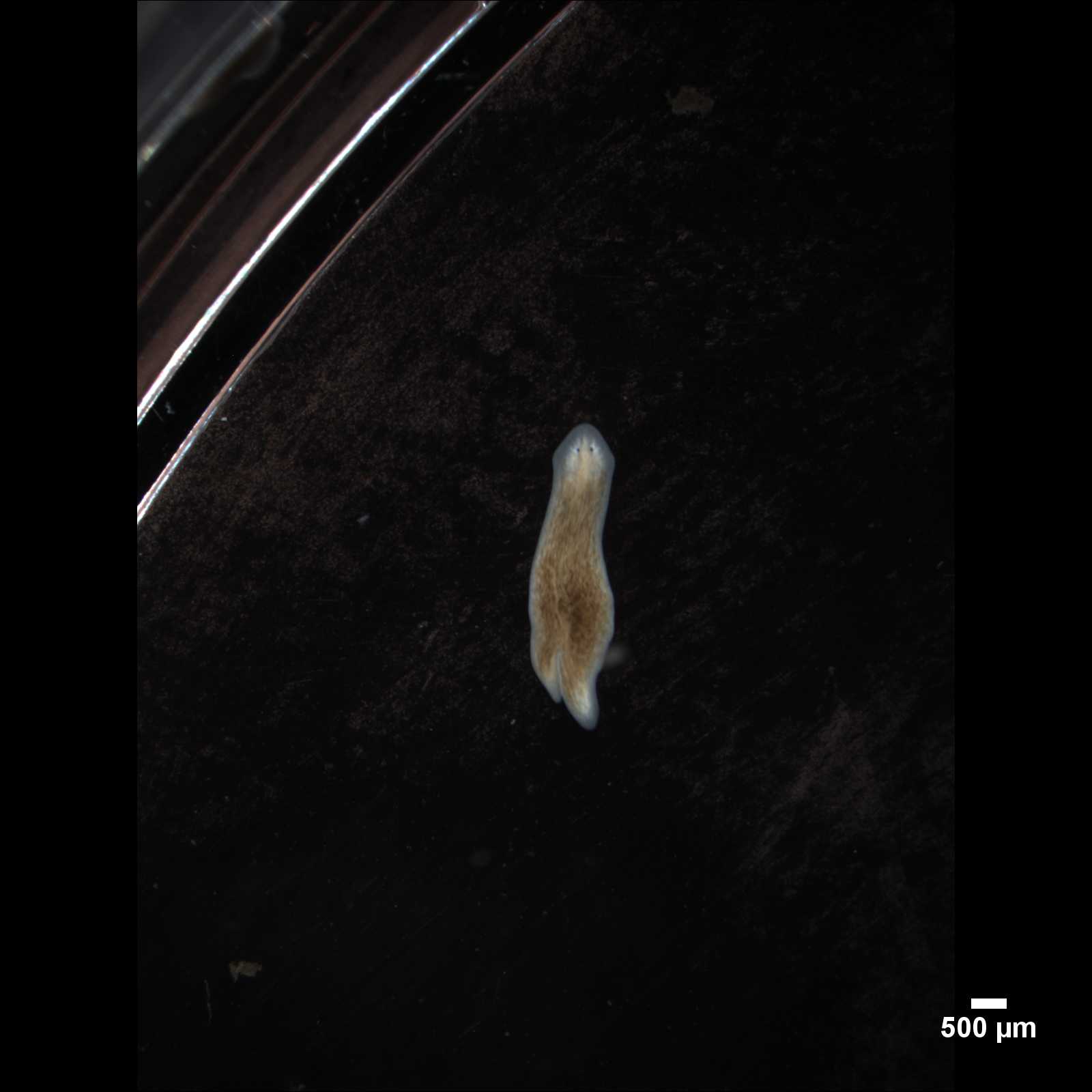

Supplement: S2 Dataset — This dataset contains raw-images of synapsin stains of uncut one- and two- headed worms, synapsin stains and brightfield images of the upwards and inverted L-cut scenarios, and synapsin stains and brightfield images showing the effects of the dynein inhibitor Ciliobrevin D on planaria regeneration. A Word document contained in the zip folder provides detailed description of the different cases. (ZIP) [file pcbi.1006904.s017.zip › DatasetS9i/L_cuts/c) upwards L-cut/brightfield pictures/Sample 3.jpg]

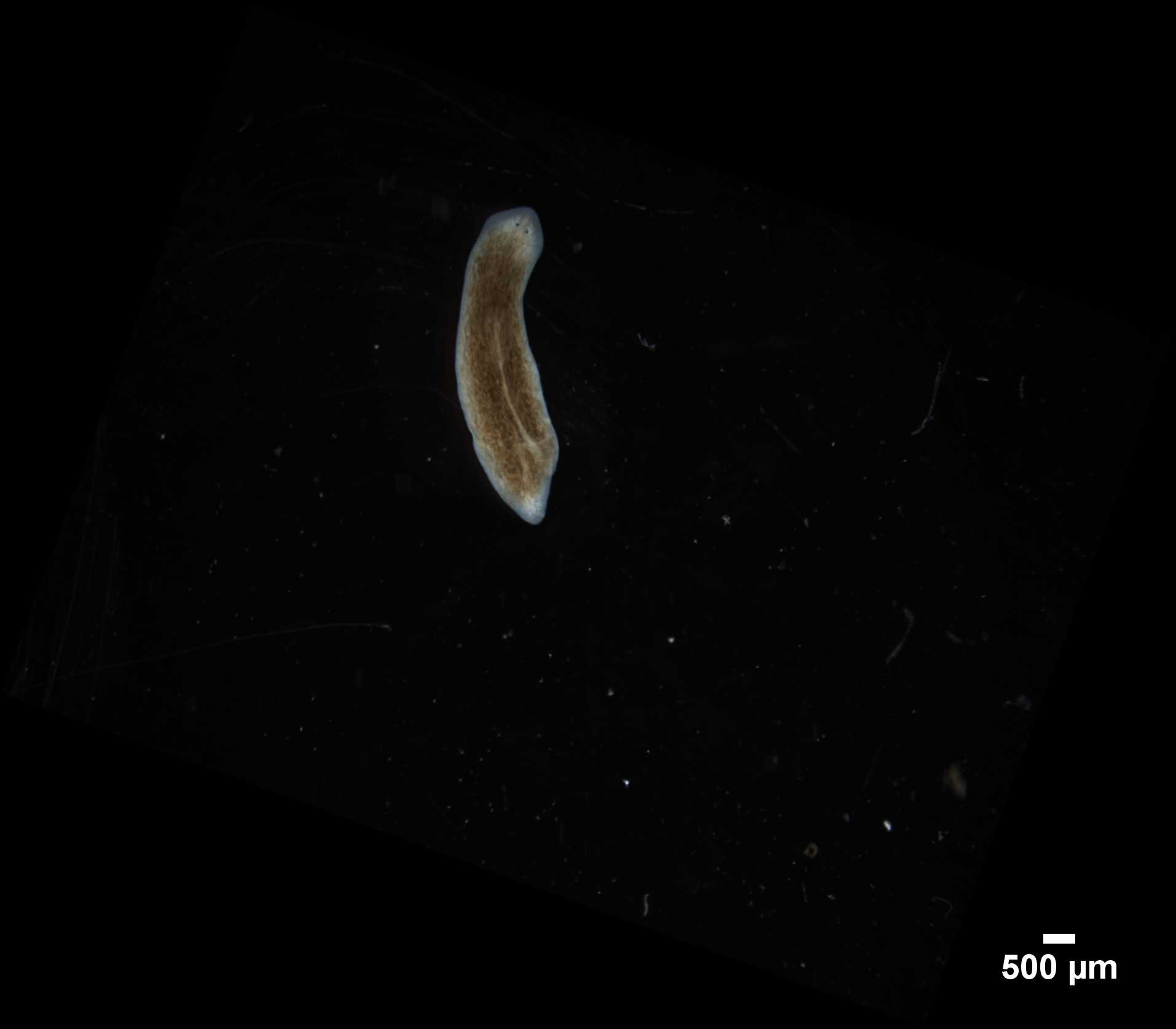

Supplement: S2 Dataset — This dataset contains raw-images of synapsin stains of uncut one- and two- headed worms, synapsin stains and brightfield images of the upwards and inverted L-cut scenarios, and synapsin stains and brightfield images showing the effects of the dynein inhibitor Ciliobrevin D on planaria regeneration. A Word document contained in the zip folder provides detailed description of the different cases. (ZIP) [file pcbi.1006904.s017.zip › DatasetS9i/L_cuts/c) upwards L-cut/brightfield pictures/Sample 4.jpg]

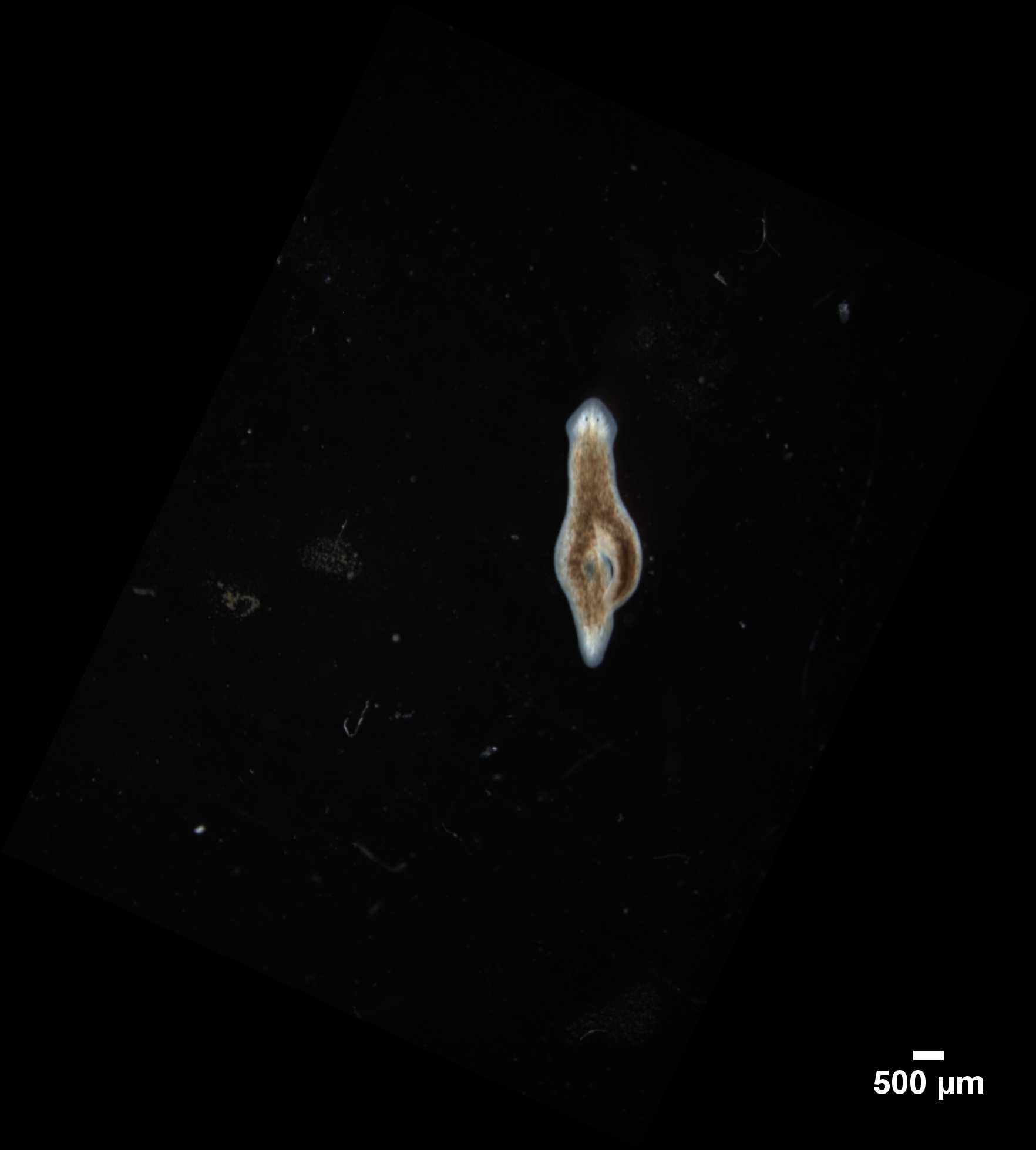

Supplement: S2 Dataset — This dataset contains raw-images of synapsin stains of uncut one- and two- headed worms, synapsin stains and brightfield images of the upwards and inverted L-cut scenarios, and synapsin stains and brightfield images showing the effects of the dynein inhibitor Ciliobrevin D on planaria regeneration. A Word document contained in the zip folder provides detailed description of the different cases. (ZIP) [file pcbi.1006904.s017.zip › DatasetS9i/L_cuts/c) upwards L-cut/brightfield pictures/Sample 5.jpg]

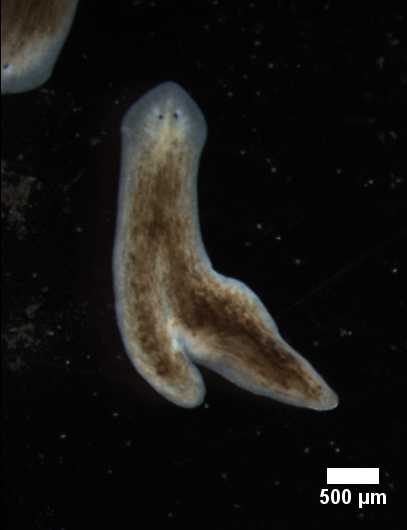

Supplement: S2 Dataset — This dataset contains raw-images of synapsin stains of uncut one- and two- headed worms, synapsin stains and brightfield images of the upwards and inverted L-cut scenarios, and synapsin stains and brightfield images showing the effects of the dynein inhibitor Ciliobrevin D on planaria regeneration. A Word document contained in the zip folder provides detailed description of the different cases. (ZIP) [file pcbi.1006904.s017.zip › DatasetS9i/L_cuts/c) upwards L-cut/brightfield pictures/Sample 6.jpg]

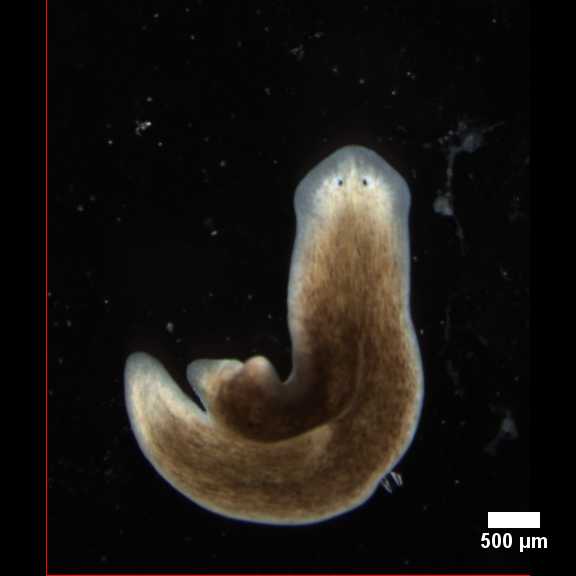

Supplement: S2 Dataset — This dataset contains raw-images of synapsin stains of uncut one- and two- headed worms, synapsin stains and brightfield images of the upwards and inverted L-cut scenarios, and synapsin stains and brightfield images showing the effects of the dynein inhibitor Ciliobrevin D on planaria regeneration. A Word document contained in the zip folder provides detailed description of the different cases. (ZIP) [file pcbi.1006904.s017.zip › DatasetS9i/L_cuts/c) upwards L-cut/brightfield pictures/Sample 7.jpg]

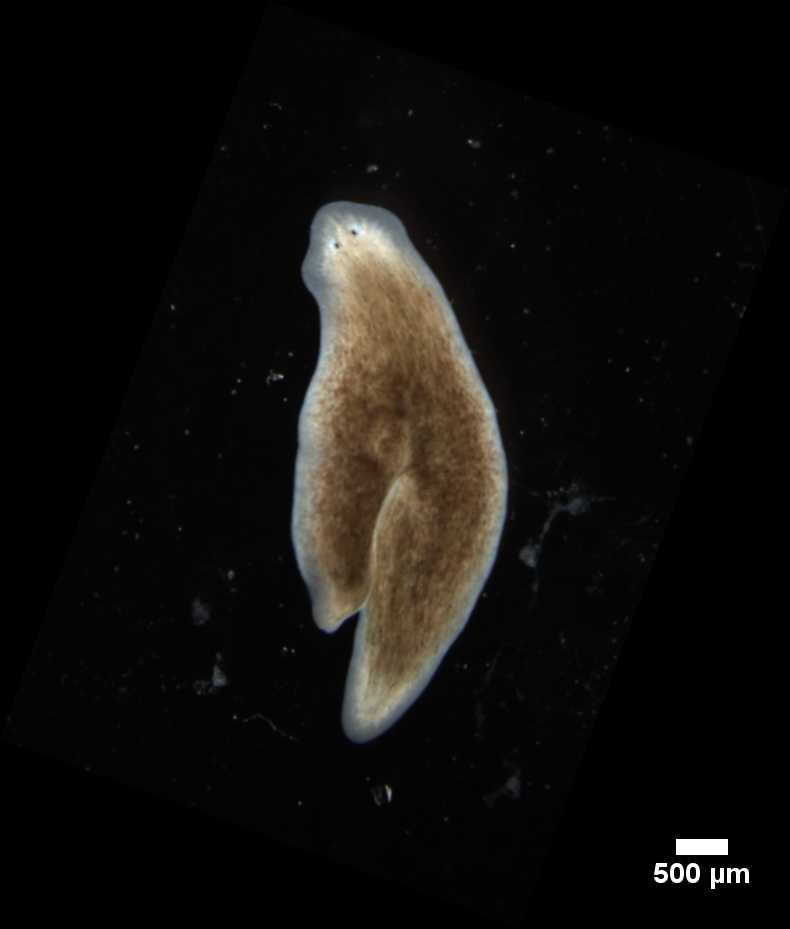

Supplement: S2 Dataset — This dataset contains raw-images of synapsin stains of uncut one- and two- headed worms, synapsin stains and brightfield images of the upwards and inverted L-cut scenarios, and synapsin stains and brightfield images showing the effects of the dynein inhibitor Ciliobrevin D on planaria regeneration. A Word document contained in the zip folder provides detailed description of the different cases. (ZIP) [file pcbi.1006904.s017.zip › DatasetS9i/L_cuts/c) upwards L-cut/brightfield pictures/Sample 8.jpg]

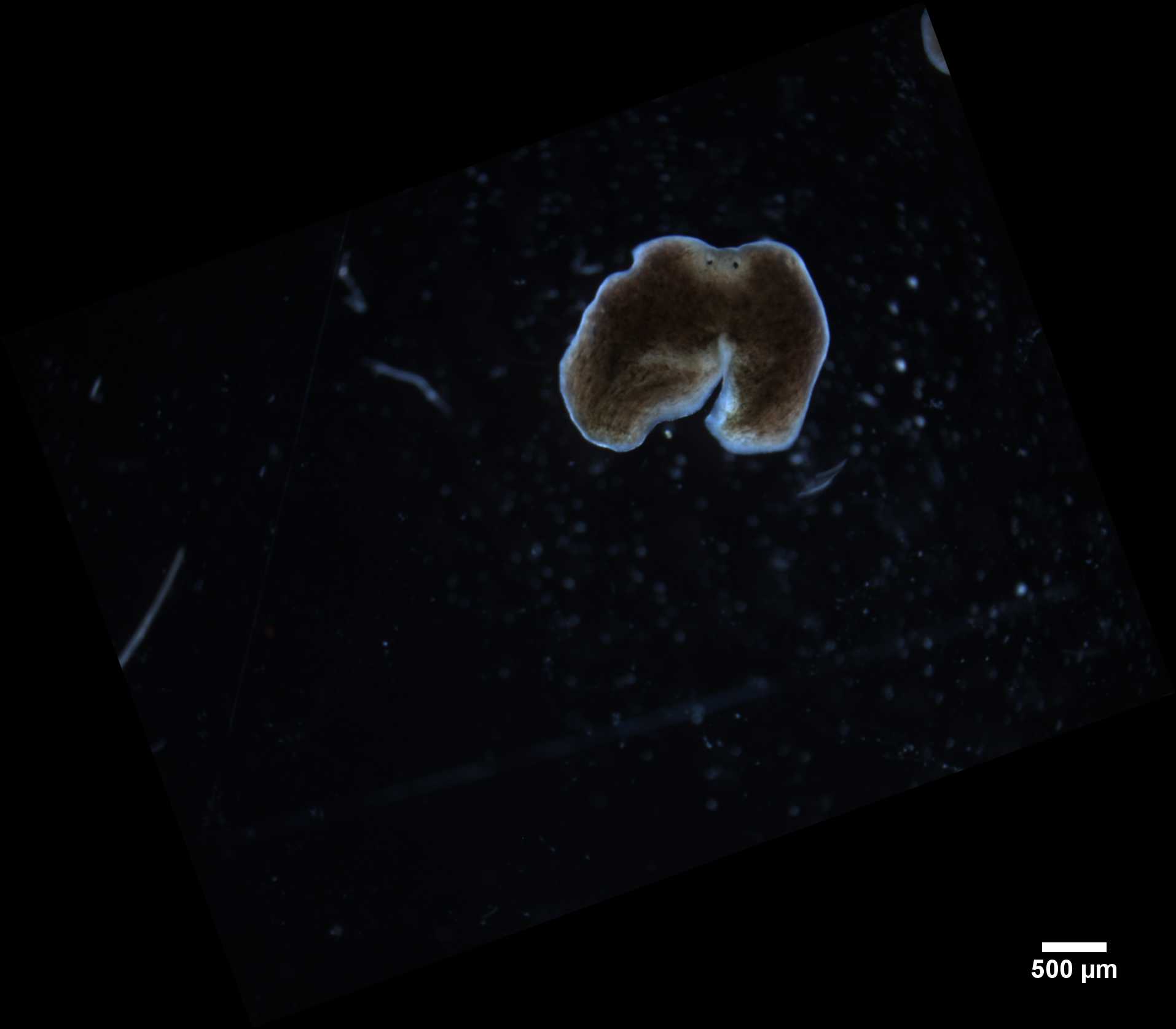

Supplement: S2 Dataset — This dataset contains raw-images of synapsin stains of uncut one- and two- headed worms, synapsin stains and brightfield images of the upwards and inverted L-cut scenarios, and synapsin stains and brightfield images showing the effects of the dynein inhibitor Ciliobrevin D on planaria regeneration. A Word document contained in the zip folder provides detailed description of the different cases. (ZIP) [file pcbi.1006904.s017.zip › DatasetS9i/L_cuts/c) upwards L-cut/brightfield pictures/Sample 9.jpg]

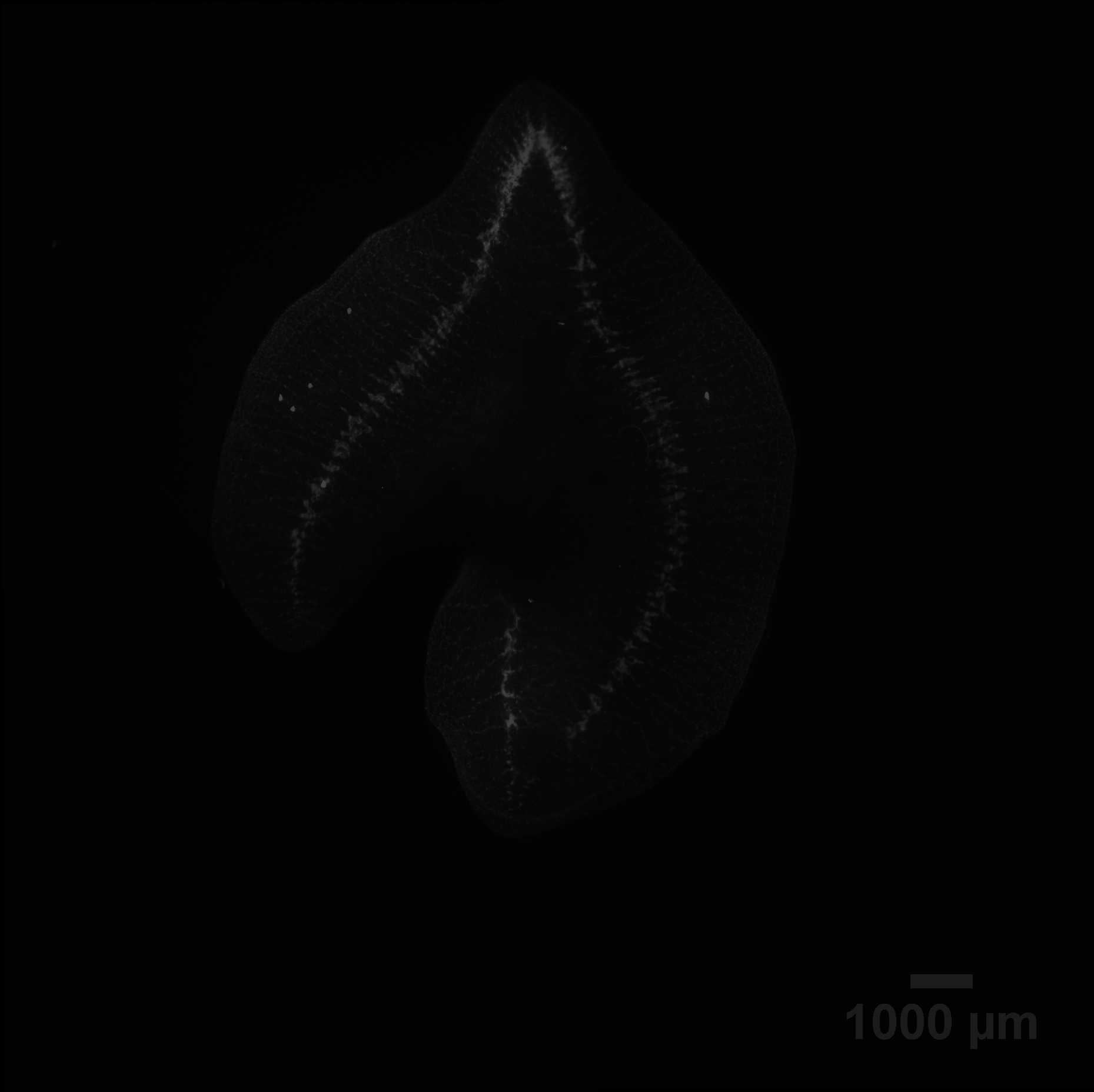

Supplement: S2 Dataset — This dataset contains raw-images of synapsin stains of uncut one- and two- headed worms, synapsin stains and brightfield images of the upwards and inverted L-cut scenarios, and synapsin stains and brightfield images showing the effects of the dynein inhibitor Ciliobrevin D on planaria regeneration. A Word document contained in the zip folder provides detailed description of the different cases. (ZIP) [file pcbi.1006904.s017.zip › DatasetS9i/L_cuts/c) upwards L-cut/synapsin stain/11 dpc_Sample 1.jpg]

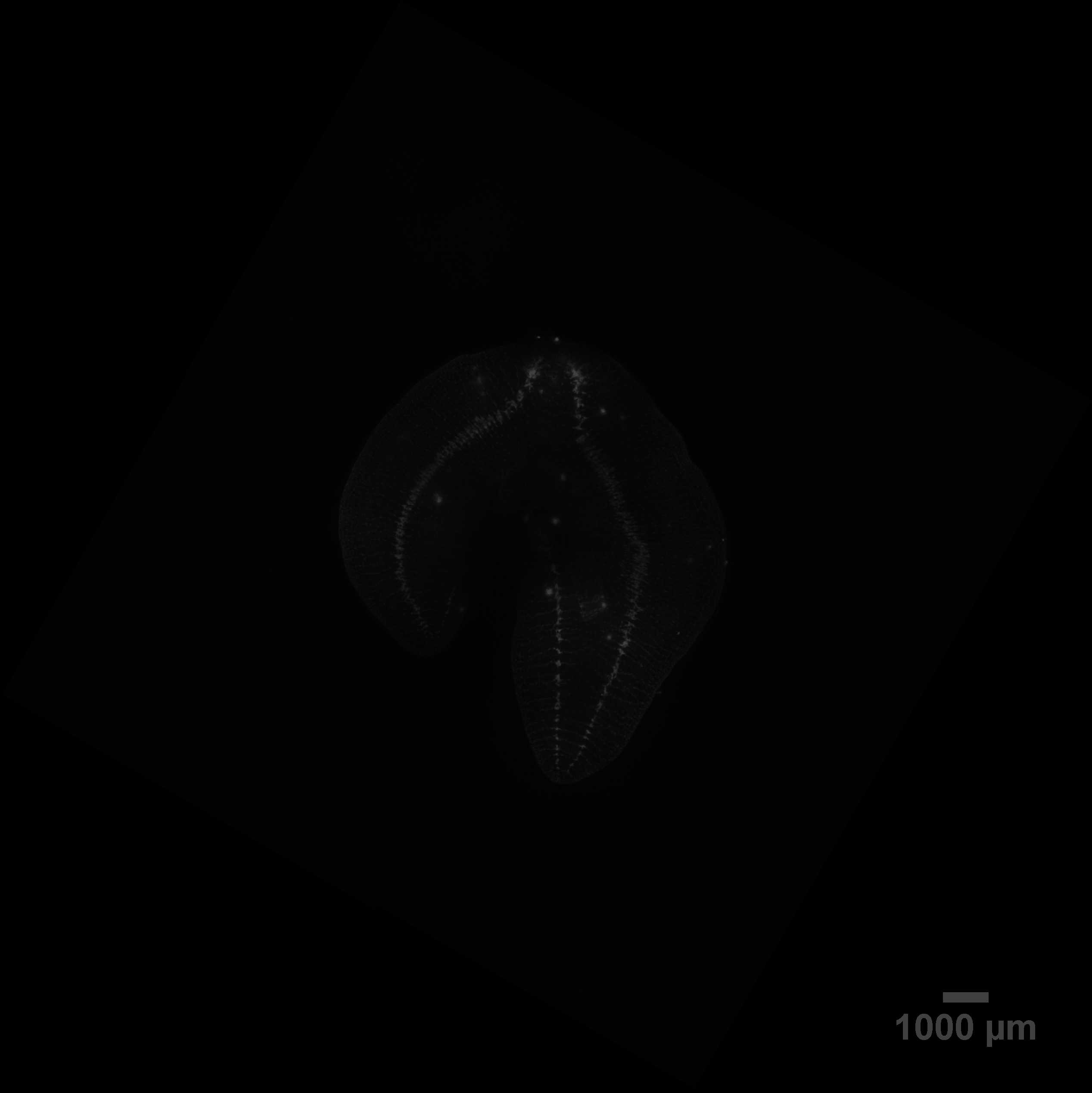

Supplement: S2 Dataset — This dataset contains raw-images of synapsin stains of uncut one- and two- headed worms, synapsin stains and brightfield images of the upwards and inverted L-cut scenarios, and synapsin stains and brightfield images showing the effects of the dynein inhibitor Ciliobrevin D on planaria regeneration. A Word document contained in the zip folder provides detailed description of the different cases. (ZIP) [file pcbi.1006904.s017.zip › DatasetS9i/L_cuts/c) upwards L-cut/synapsin stain/11 dpc_Sample 2.jpg]

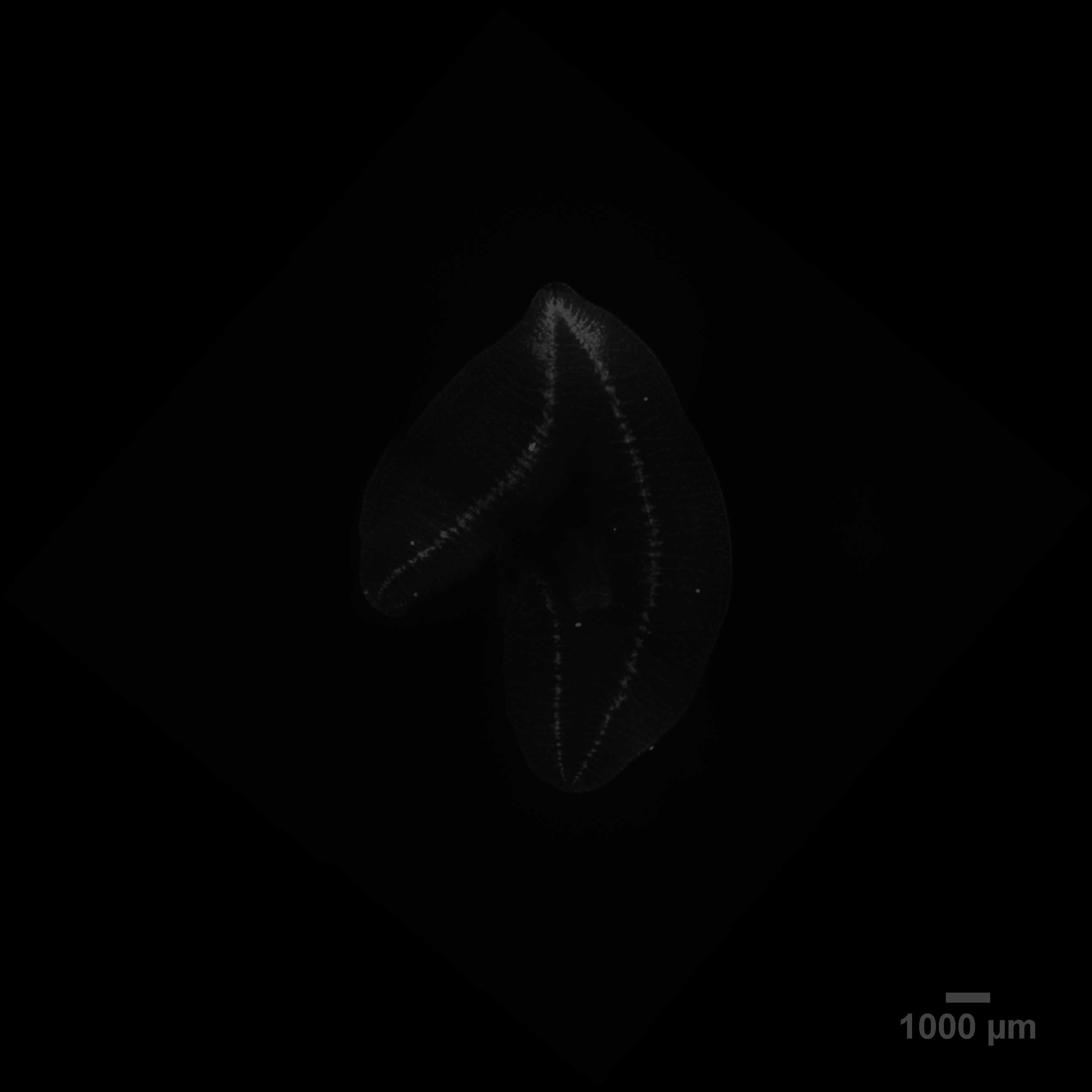

Supplement: S2 Dataset — This dataset contains raw-images of synapsin stains of uncut one- and two- headed worms, synapsin stains and brightfield images of the upwards and inverted L-cut scenarios, and synapsin stains and brightfield images showing the effects of the dynein inhibitor Ciliobrevin D on planaria regeneration. A Word document contained in the zip folder provides detailed description of the different cases. (ZIP) [file pcbi.1006904.s017.zip › DatasetS9i/L_cuts/c) upwards L-cut/synapsin stain/11 dpc_Sample 3.jpg]
