# Supplementary figures and images for: Neural control of body-plan axis in regenerating planaria (part 2 of 4)
Source: PLoS Comput Biol. 2019 Apr 16;15(4):e1006904. doi: 10.1371/journal.pcbi.1006904 (PMC6485777; doi:10.1371/journal.pcbi.1006904)

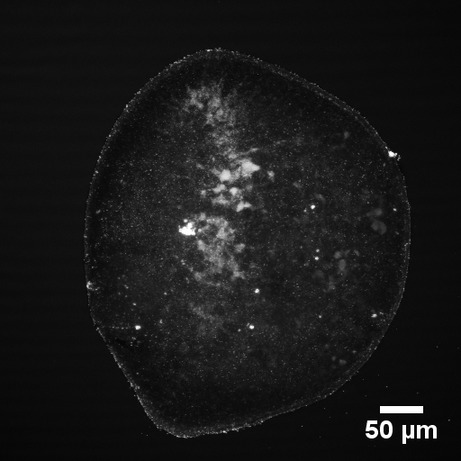

Supplement: S1 Dataset — This dataset contains brightfield image and corresponding synapsin stains for the VNC-free and VNC-containing small fragment cutting scenarios shown in Fig 6. Each image is labeled in the format “x_dpc_Sample_y_tn.jpg”, where “x” represents the number of days post cutting and “y” the replicate number. (ZIP) [file pcbi.1006904.s016.zip › smallfragments/VNC-containing/synapsin_stains/6 dpc_Sample 1_tn.jpg]

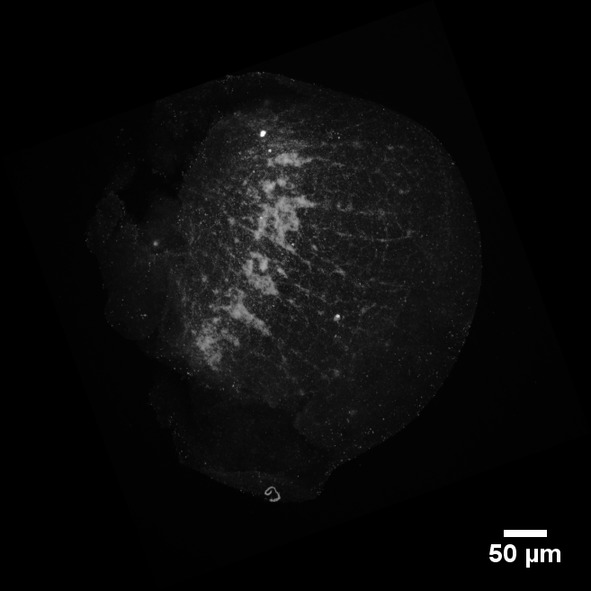

Supplement: S1 Dataset — This dataset contains brightfield image and corresponding synapsin stains for the VNC-free and VNC-containing small fragment cutting scenarios shown in Fig 6. Each image is labeled in the format “x_dpc_Sample_y_tn.jpg”, where “x” represents the number of days post cutting and “y” the replicate number. (ZIP) [file pcbi.1006904.s016.zip › smallfragments/VNC-containing/synapsin_stains/6 dpc_Sample 2_tn.jpg]

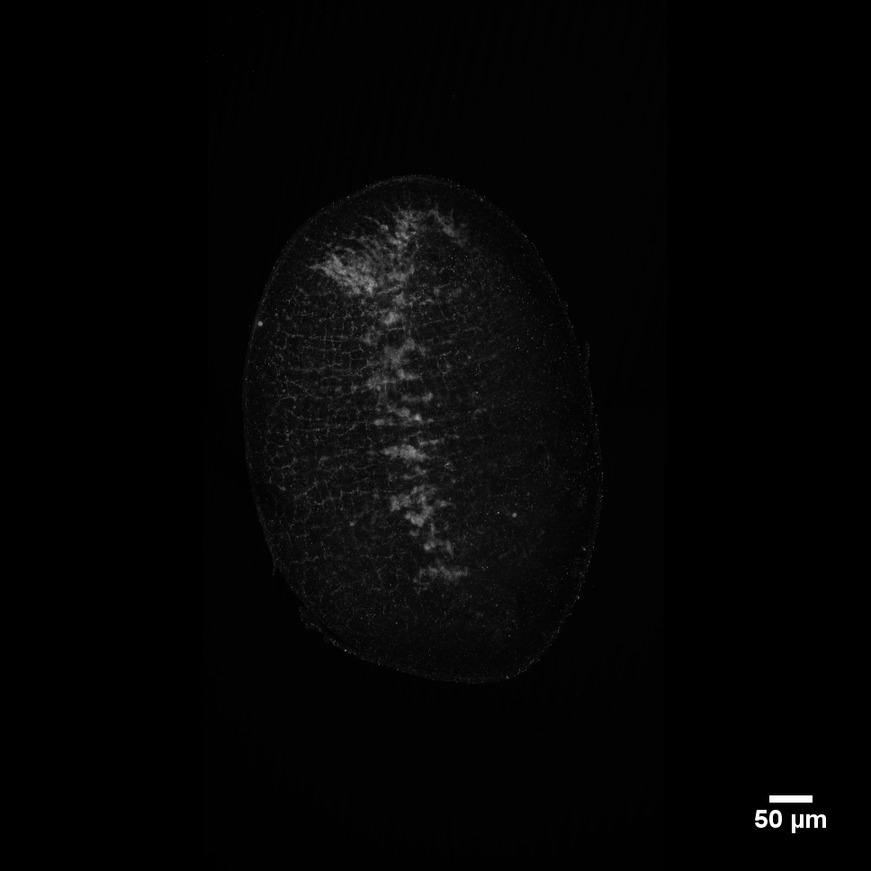

Supplement: S1 Dataset — This dataset contains brightfield image and corresponding synapsin stains for the VNC-free and VNC-containing small fragment cutting scenarios shown in Fig 6. Each image is labeled in the format “x_dpc_Sample_y_tn.jpg”, where “x” represents the number of days post cutting and “y” the replicate number. (ZIP) [file pcbi.1006904.s016.zip › smallfragments/VNC-containing/synapsin_stains/6 dpc_Sample 3_tn.jpg]

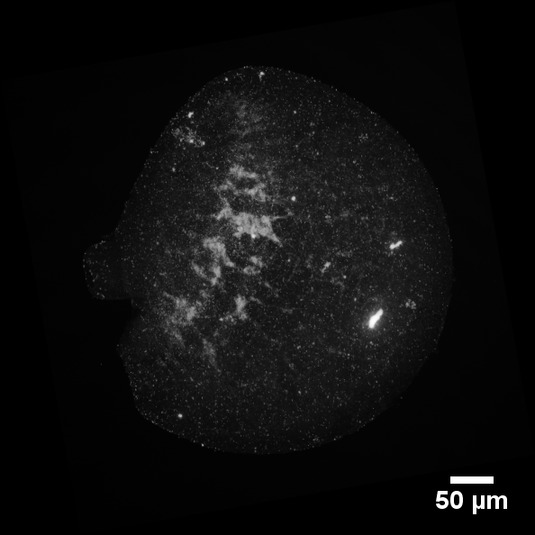

Supplement: S1 Dataset — This dataset contains brightfield image and corresponding synapsin stains for the VNC-free and VNC-containing small fragment cutting scenarios shown in Fig 6. Each image is labeled in the format “x_dpc_Sample_y_tn.jpg”, where “x” represents the number of days post cutting and “y” the replicate number. (ZIP) [file pcbi.1006904.s016.zip › smallfragments/VNC-containing/synapsin_stains/6 dpc_Sample 4_tn.jpg]

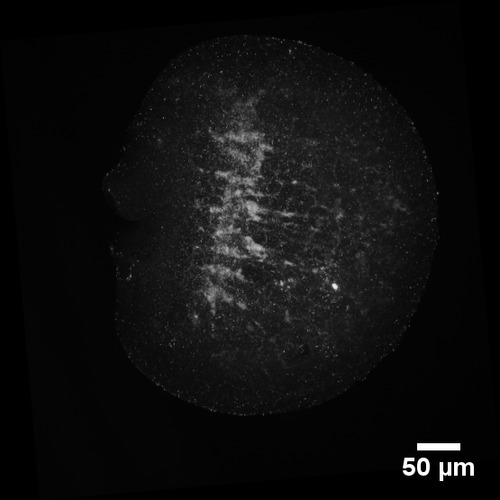

Supplement: S1 Dataset — This dataset contains brightfield image and corresponding synapsin stains for the VNC-free and VNC-containing small fragment cutting scenarios shown in Fig 6. Each image is labeled in the format “x_dpc_Sample_y_tn.jpg”, where “x” represents the number of days post cutting and “y” the replicate number. (ZIP) [file pcbi.1006904.s016.zip › smallfragments/VNC-containing/synapsin_stains/6 dpc_Sample 5_tn.jpg]

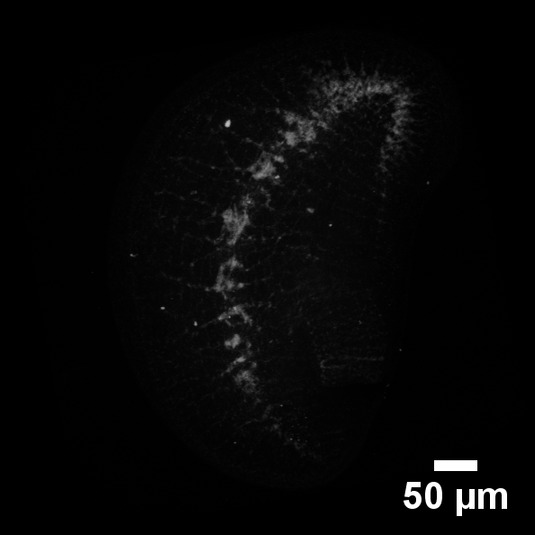

Supplement: S1 Dataset — This dataset contains brightfield image and corresponding synapsin stains for the VNC-free and VNC-containing small fragment cutting scenarios shown in Fig 6. Each image is labeled in the format “x_dpc_Sample_y_tn.jpg”, where “x” represents the number of days post cutting and “y” the replicate number. (ZIP) [file pcbi.1006904.s016.zip › smallfragments/VNC-containing/synapsin_stains/7 dpc_Sample 1_tn.jpg]

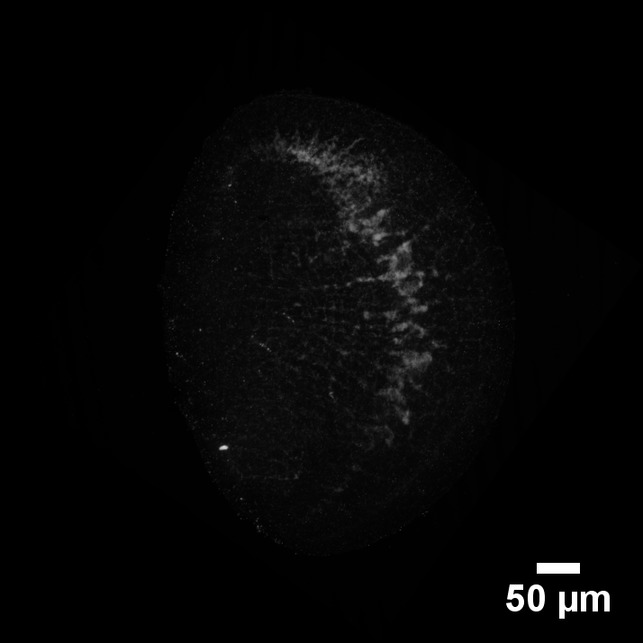

Supplement: S1 Dataset — This dataset contains brightfield image and corresponding synapsin stains for the VNC-free and VNC-containing small fragment cutting scenarios shown in Fig 6. Each image is labeled in the format “x_dpc_Sample_y_tn.jpg”, where “x” represents the number of days post cutting and “y” the replicate number. (ZIP) [file pcbi.1006904.s016.zip › smallfragments/VNC-containing/synapsin_stains/7 dpc_Sample 2_tn.jpg]

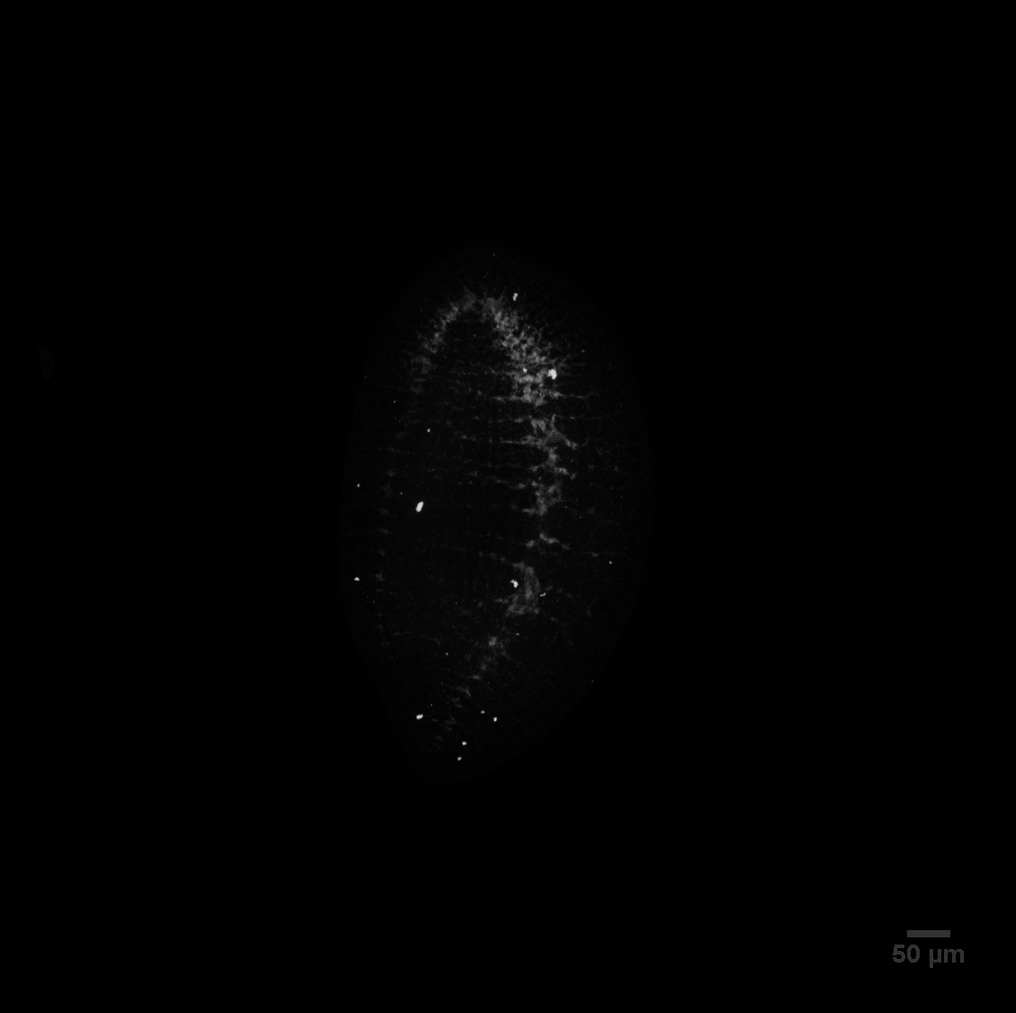

Supplement: S1 Dataset — This dataset contains brightfield image and corresponding synapsin stains for the VNC-free and VNC-containing small fragment cutting scenarios shown in Fig 6. Each image is labeled in the format “x_dpc_Sample_y_tn.jpg”, where “x” represents the number of days post cutting and “y” the replicate number. (ZIP) [file pcbi.1006904.s016.zip › smallfragments/VNC-containing/synapsin_stains/7 dpc_Sample 3_tn.jpg]

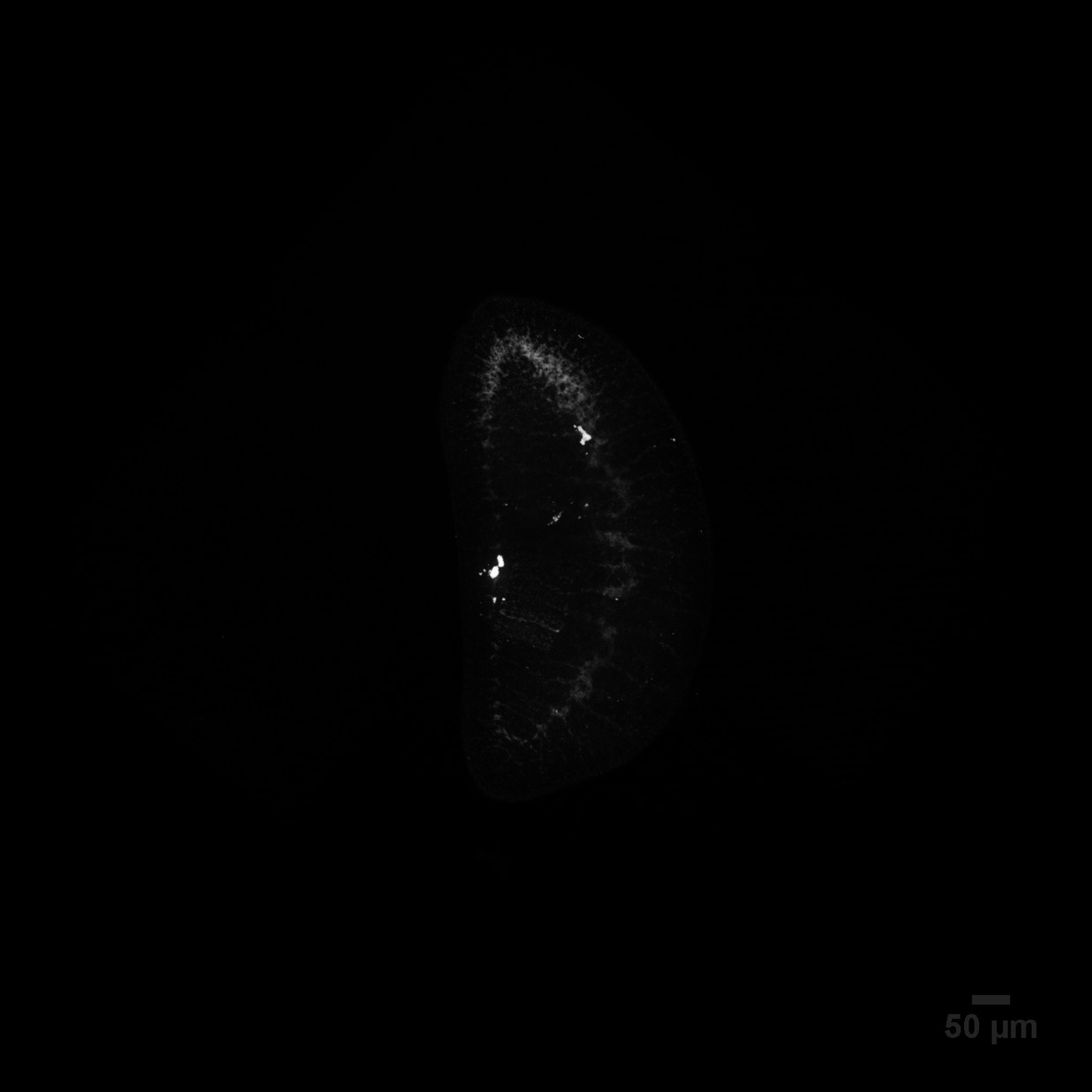

Supplement: S1 Dataset — This dataset contains brightfield image and corresponding synapsin stains for the VNC-free and VNC-containing small fragment cutting scenarios shown in Fig 6. Each image is labeled in the format “x_dpc_Sample_y_tn.jpg”, where “x” represents the number of days post cutting and “y” the replicate number. (ZIP) [file pcbi.1006904.s016.zip › smallfragments/VNC-containing/synapsin_stains/7 dpc_Sample 4_tn.jpg]

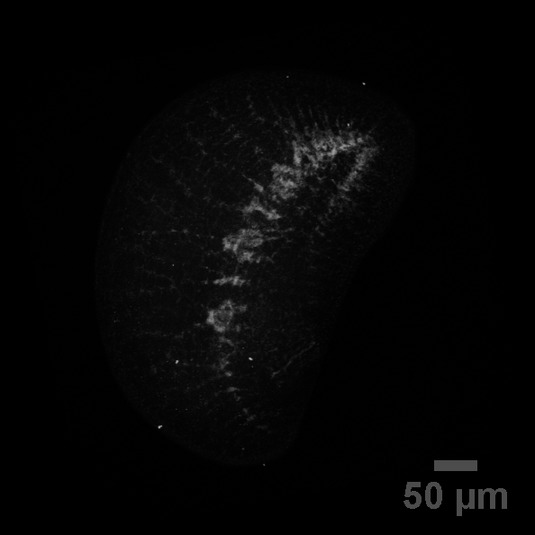

Supplement: S1 Dataset — This dataset contains brightfield image and corresponding synapsin stains for the VNC-free and VNC-containing small fragment cutting scenarios shown in Fig 6. Each image is labeled in the format “x_dpc_Sample_y_tn.jpg”, where “x” represents the number of days post cutting and “y” the replicate number. (ZIP) [file pcbi.1006904.s016.zip › smallfragments/VNC-containing/synapsin_stains/7 dpc_Sample 5_tn.jpg]

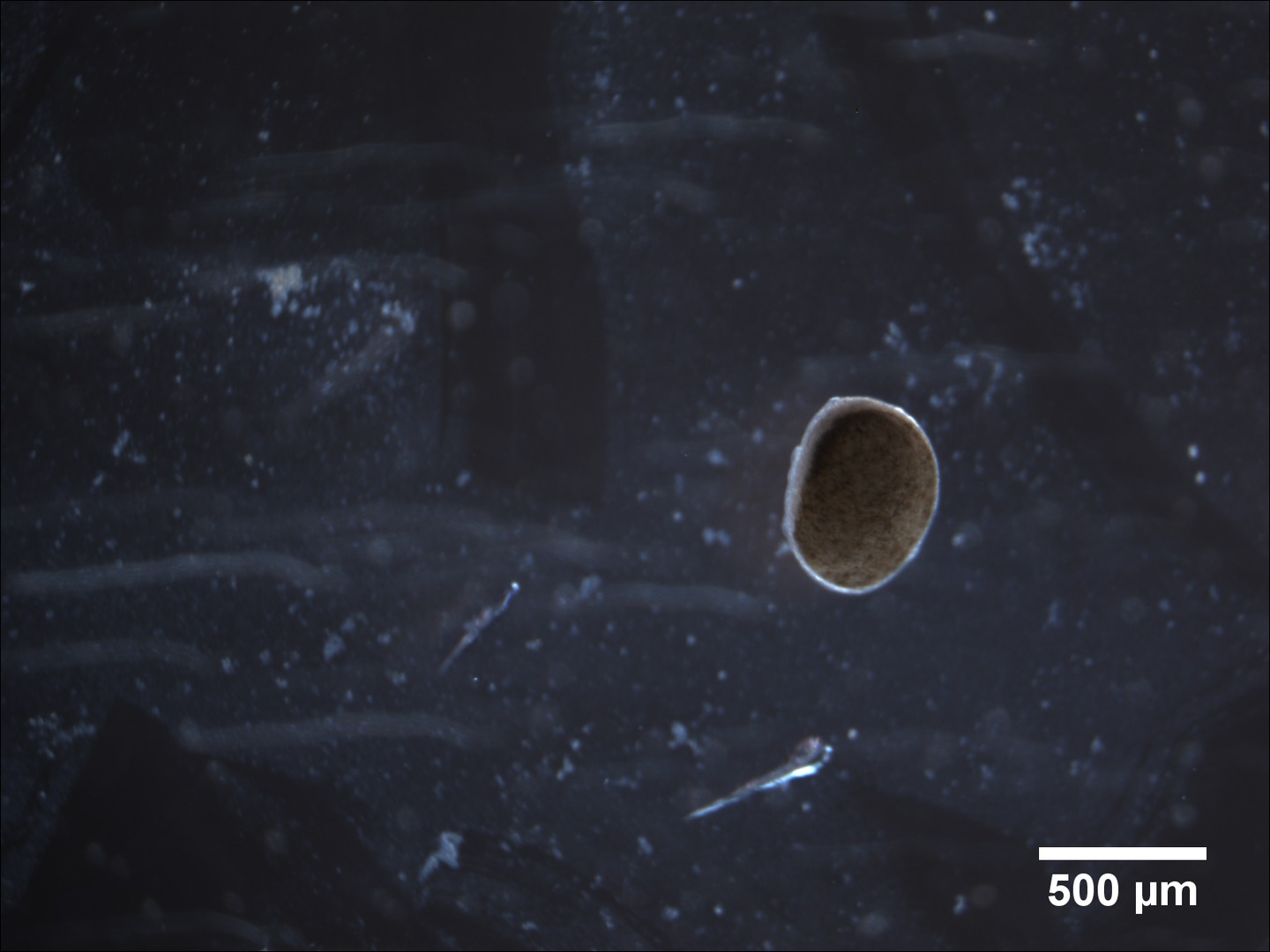

Supplement: S1 Dataset — This dataset contains brightfield image and corresponding synapsin stains for the VNC-free and VNC-containing small fragment cutting scenarios shown in Fig 6. Each image is labeled in the format “x_dpc_Sample_y_tn.jpg”, where “x” represents the number of days post cutting and “y” the replicate number. (ZIP) [file pcbi.1006904.s016.zip › smallfragments/VNC-free/Brigthfield_images/1 dpc_Sample 1_tn.jpg]

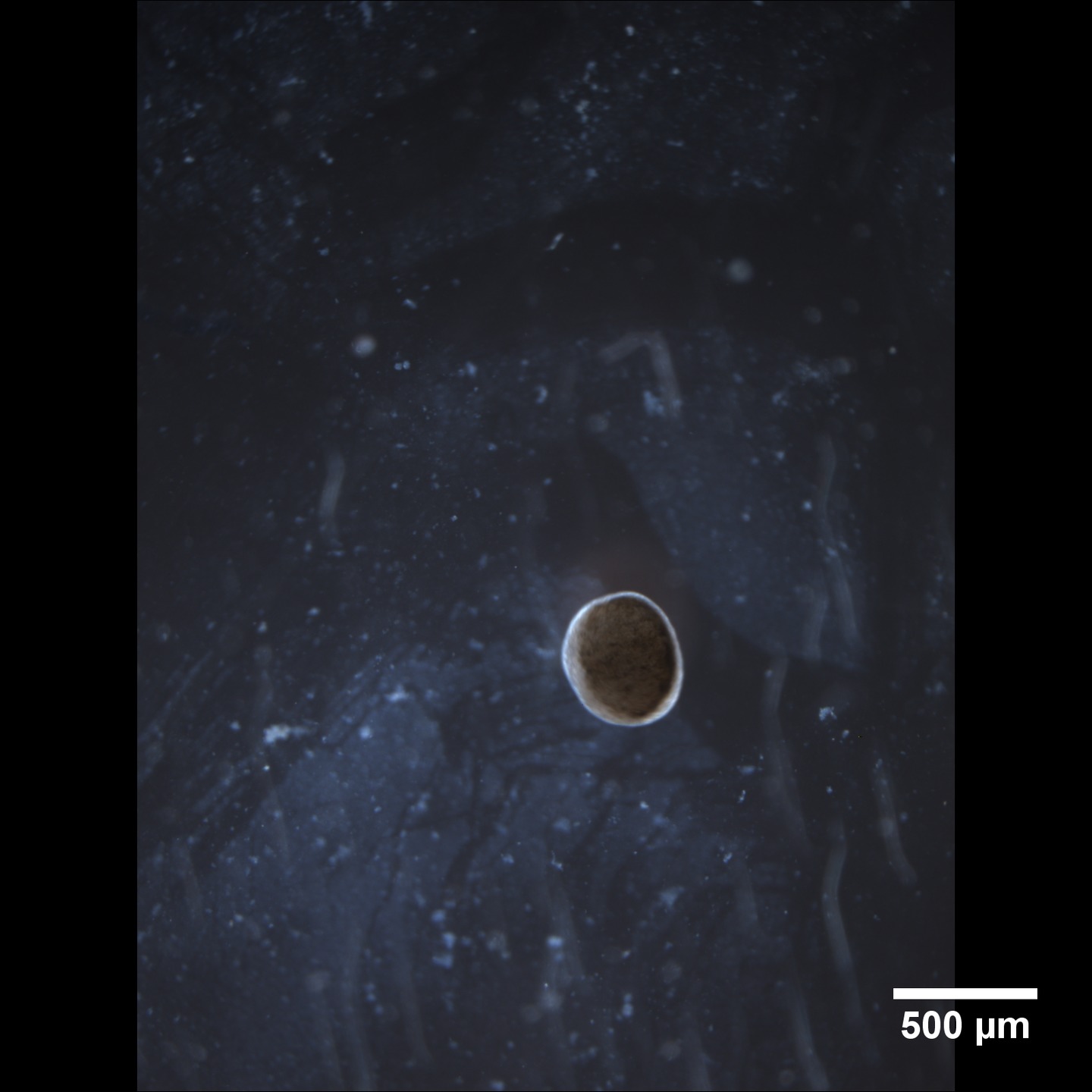

Supplement: S1 Dataset — This dataset contains brightfield image and corresponding synapsin stains for the VNC-free and VNC-containing small fragment cutting scenarios shown in Fig 6. Each image is labeled in the format “x_dpc_Sample_y_tn.jpg”, where “x” represents the number of days post cutting and “y” the replicate number. (ZIP) [file pcbi.1006904.s016.zip › smallfragments/VNC-free/Brigthfield_images/1 dpc_Sample 2_tn.jpg]

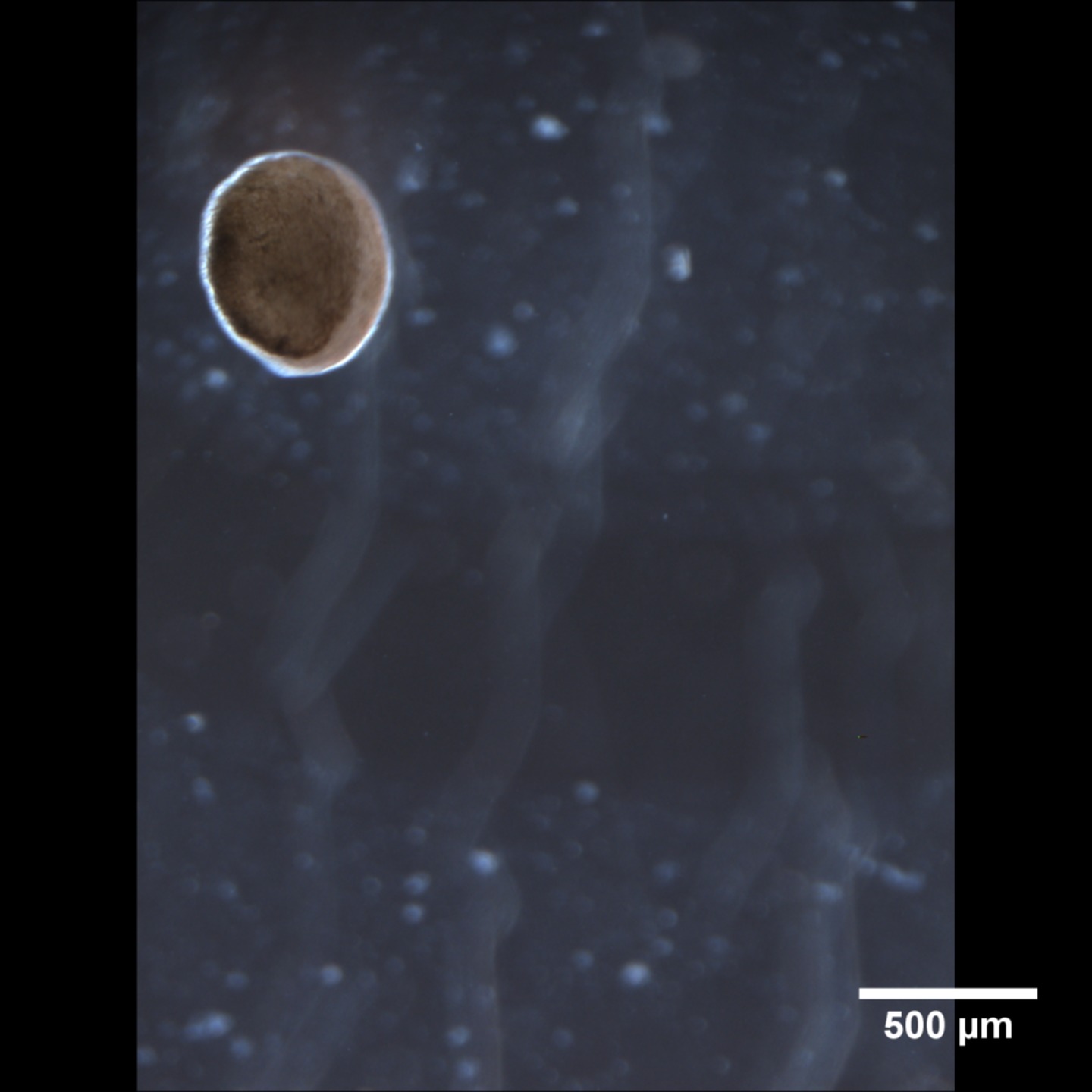

Supplement: S1 Dataset — This dataset contains brightfield image and corresponding synapsin stains for the VNC-free and VNC-containing small fragment cutting scenarios shown in Fig 6. Each image is labeled in the format “x_dpc_Sample_y_tn.jpg”, where “x” represents the number of days post cutting and “y” the replicate number. (ZIP) [file pcbi.1006904.s016.zip › smallfragments/VNC-free/Brigthfield_images/1 dpc_Sample 3_tn.jpg]

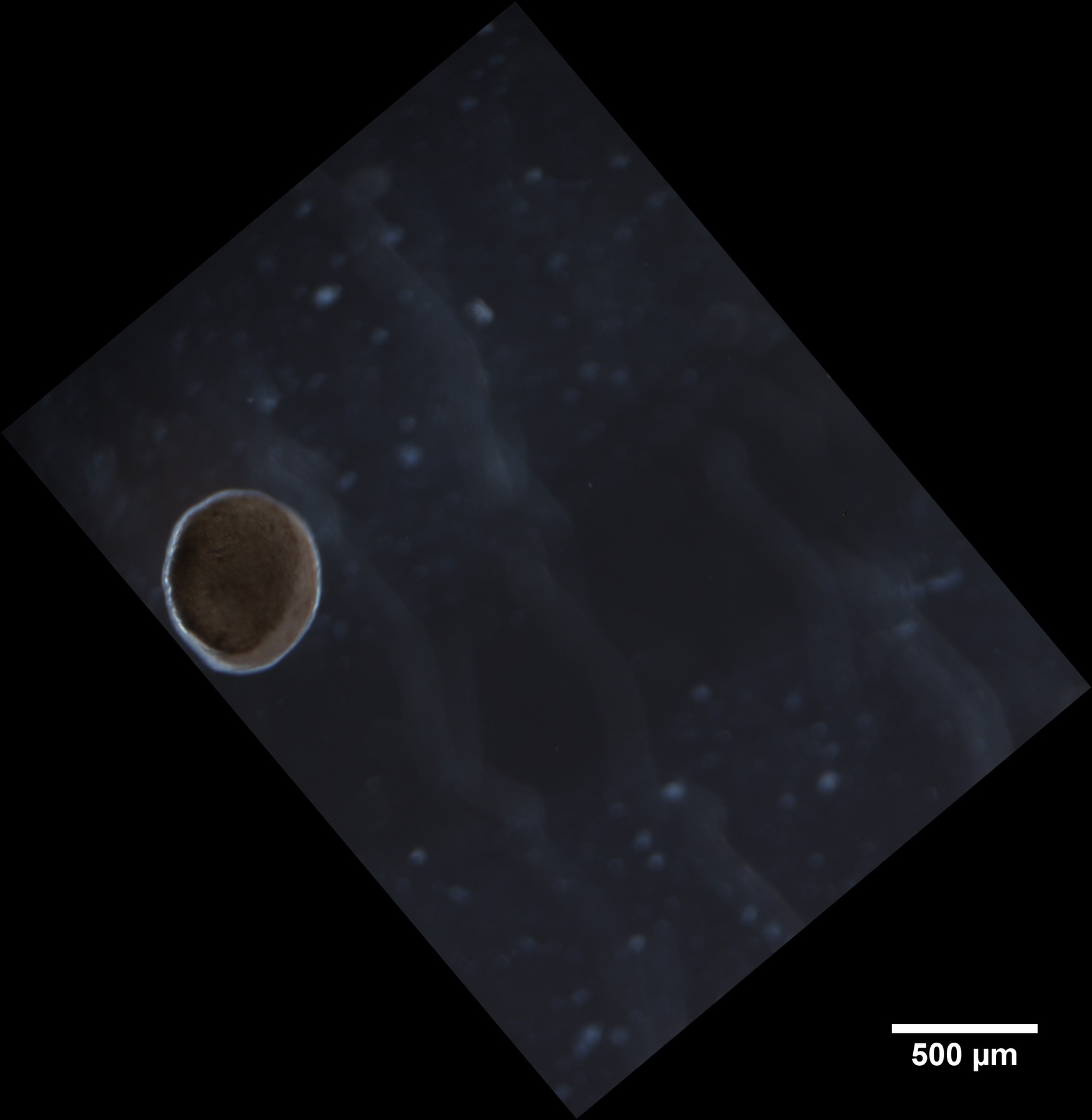

Supplement: S1 Dataset — This dataset contains brightfield image and corresponding synapsin stains for the VNC-free and VNC-containing small fragment cutting scenarios shown in Fig 6. Each image is labeled in the format “x_dpc_Sample_y_tn.jpg”, where “x” represents the number of days post cutting and “y” the replicate number. (ZIP) [file pcbi.1006904.s016.zip › smallfragments/VNC-free/Brigthfield_images/1 dpc_Sample 4_tn.jpg]

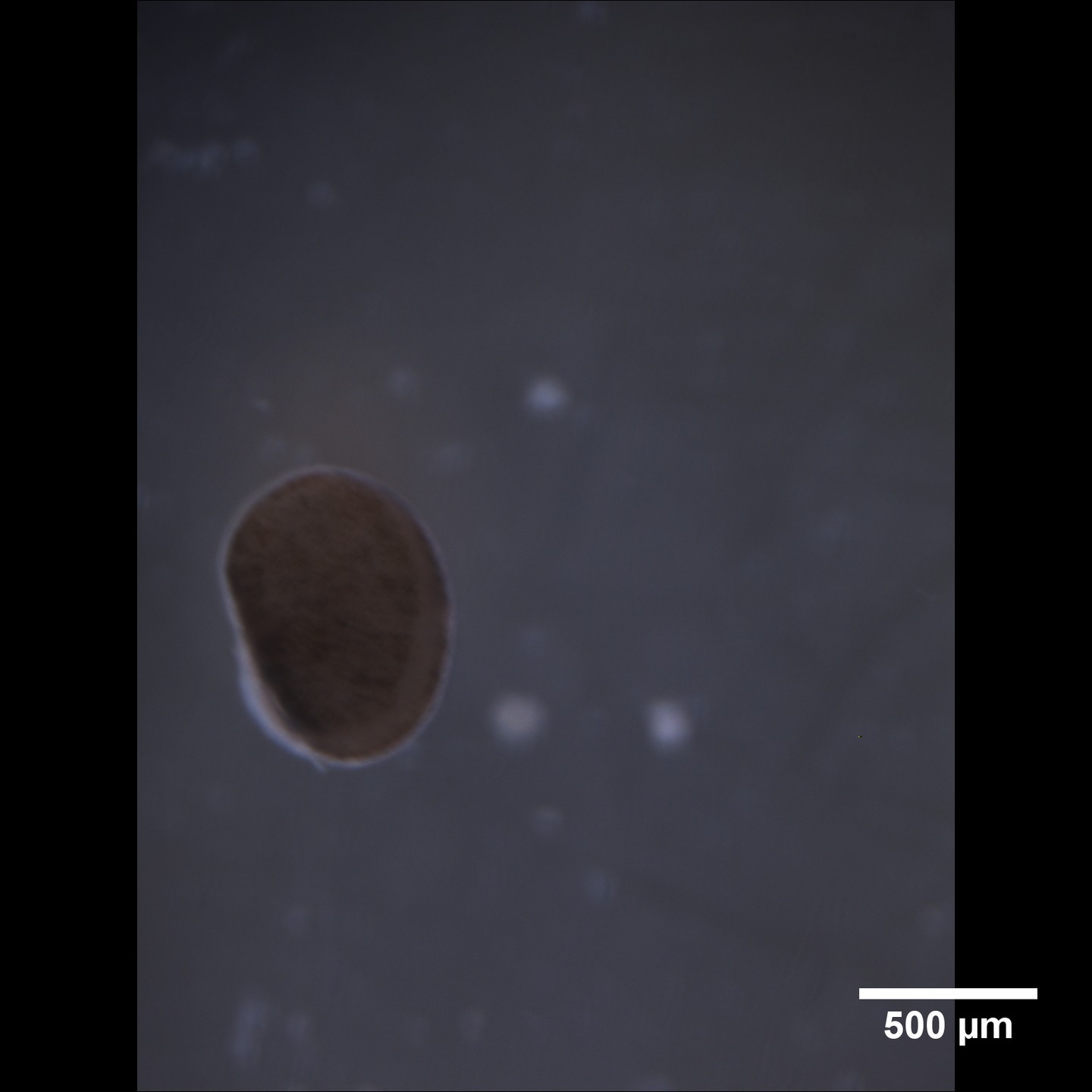

Supplement: S1 Dataset — This dataset contains brightfield image and corresponding synapsin stains for the VNC-free and VNC-containing small fragment cutting scenarios shown in Fig 6. Each image is labeled in the format “x_dpc_Sample_y_tn.jpg”, where “x” represents the number of days post cutting and “y” the replicate number. (ZIP) [file pcbi.1006904.s016.zip › smallfragments/VNC-free/Brigthfield_images/1 dpc_Sample 5_tn.jpg]

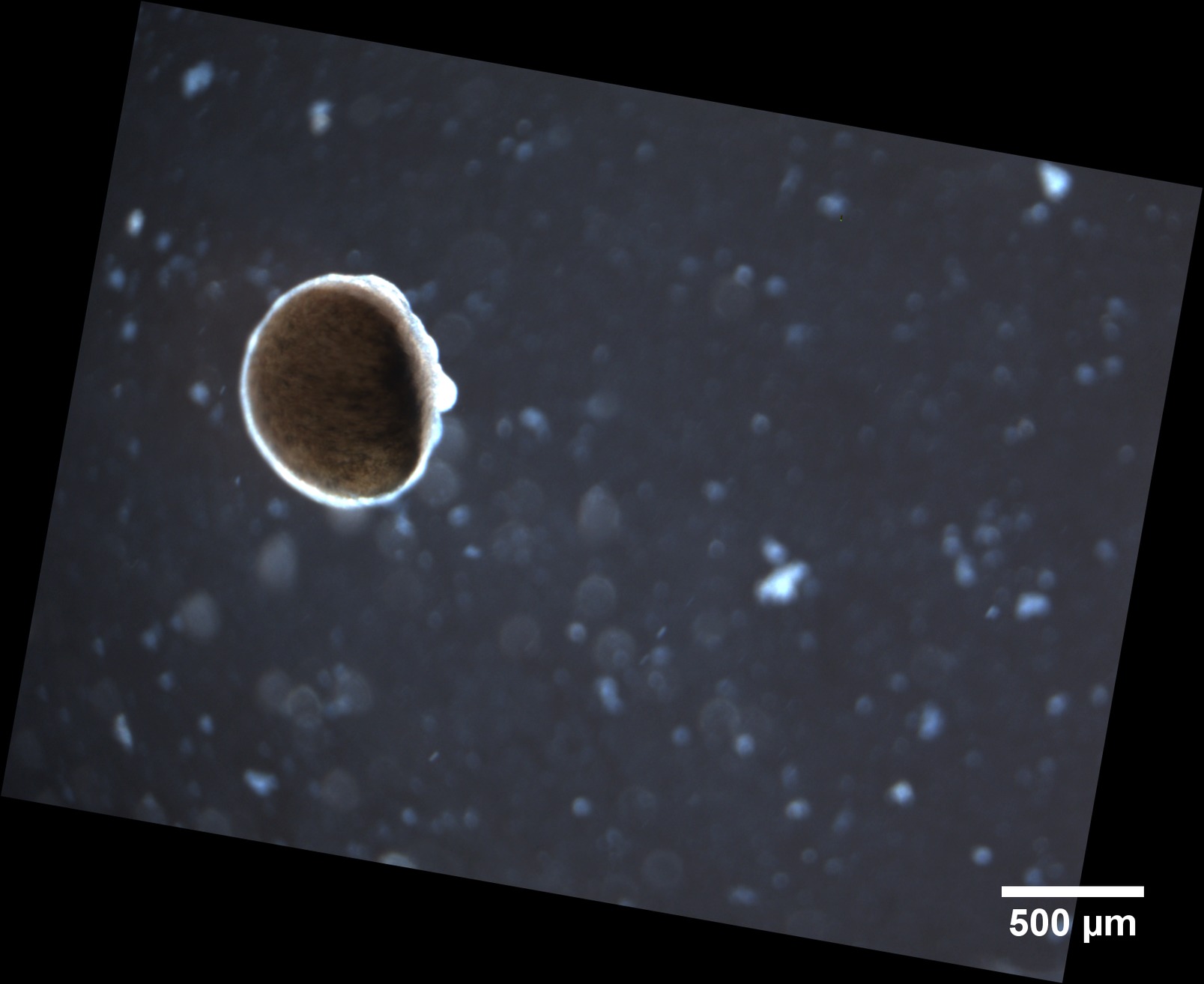

Supplement: S1 Dataset — This dataset contains brightfield image and corresponding synapsin stains for the VNC-free and VNC-containing small fragment cutting scenarios shown in Fig 6. Each image is labeled in the format “x_dpc_Sample_y_tn.jpg”, where “x” represents the number of days post cutting and “y” the replicate number. (ZIP) [file pcbi.1006904.s016.zip › smallfragments/VNC-free/Brigthfield_images/1 dpc_Sample 6_tn.jpg]

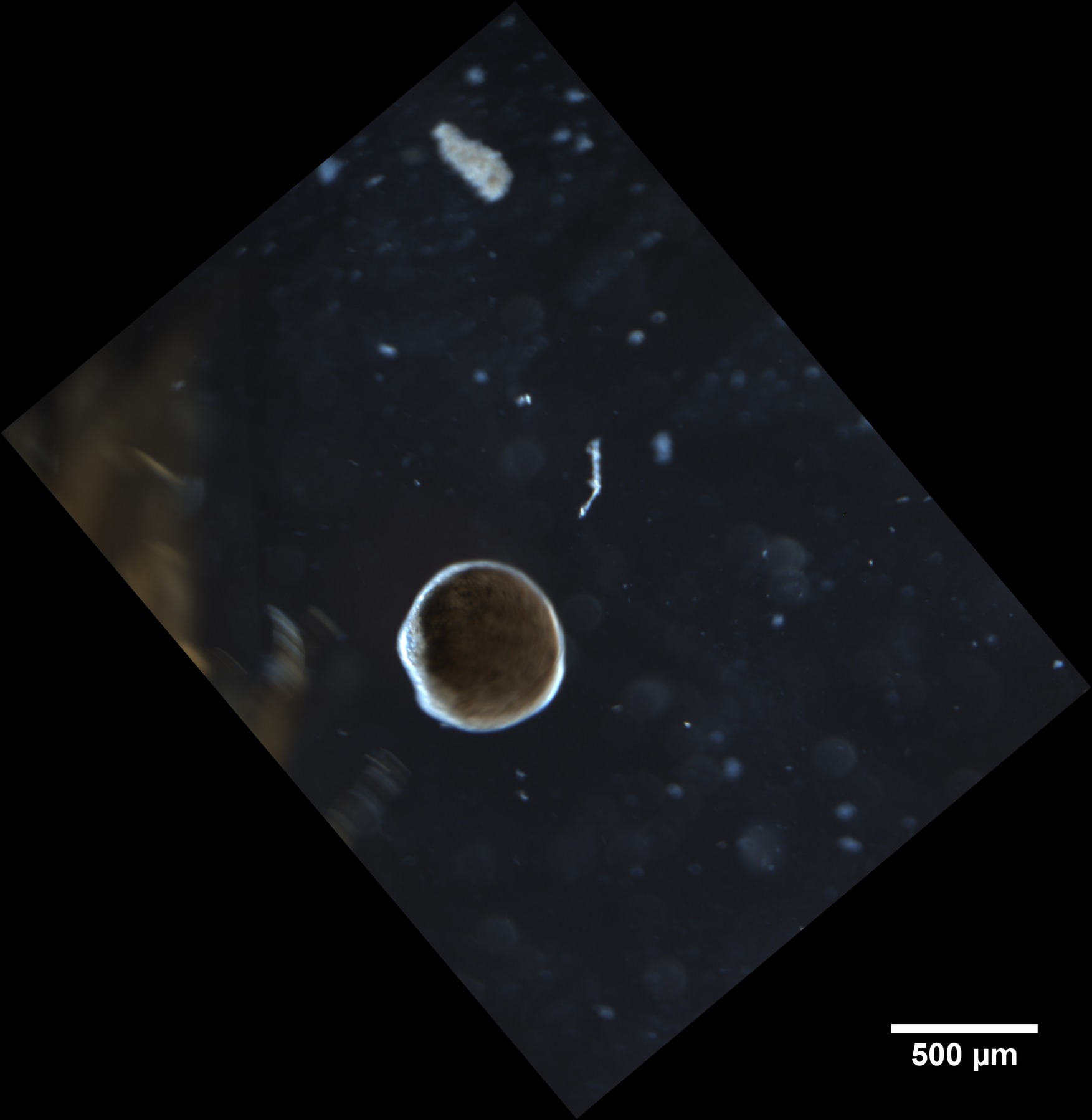

Supplement: S1 Dataset — This dataset contains brightfield image and corresponding synapsin stains for the VNC-free and VNC-containing small fragment cutting scenarios shown in Fig 6. Each image is labeled in the format “x_dpc_Sample_y_tn.jpg”, where “x” represents the number of days post cutting and “y” the replicate number. (ZIP) [file pcbi.1006904.s016.zip › smallfragments/VNC-free/Brigthfield_images/1 dpc_Sample 7_tn.jpg]

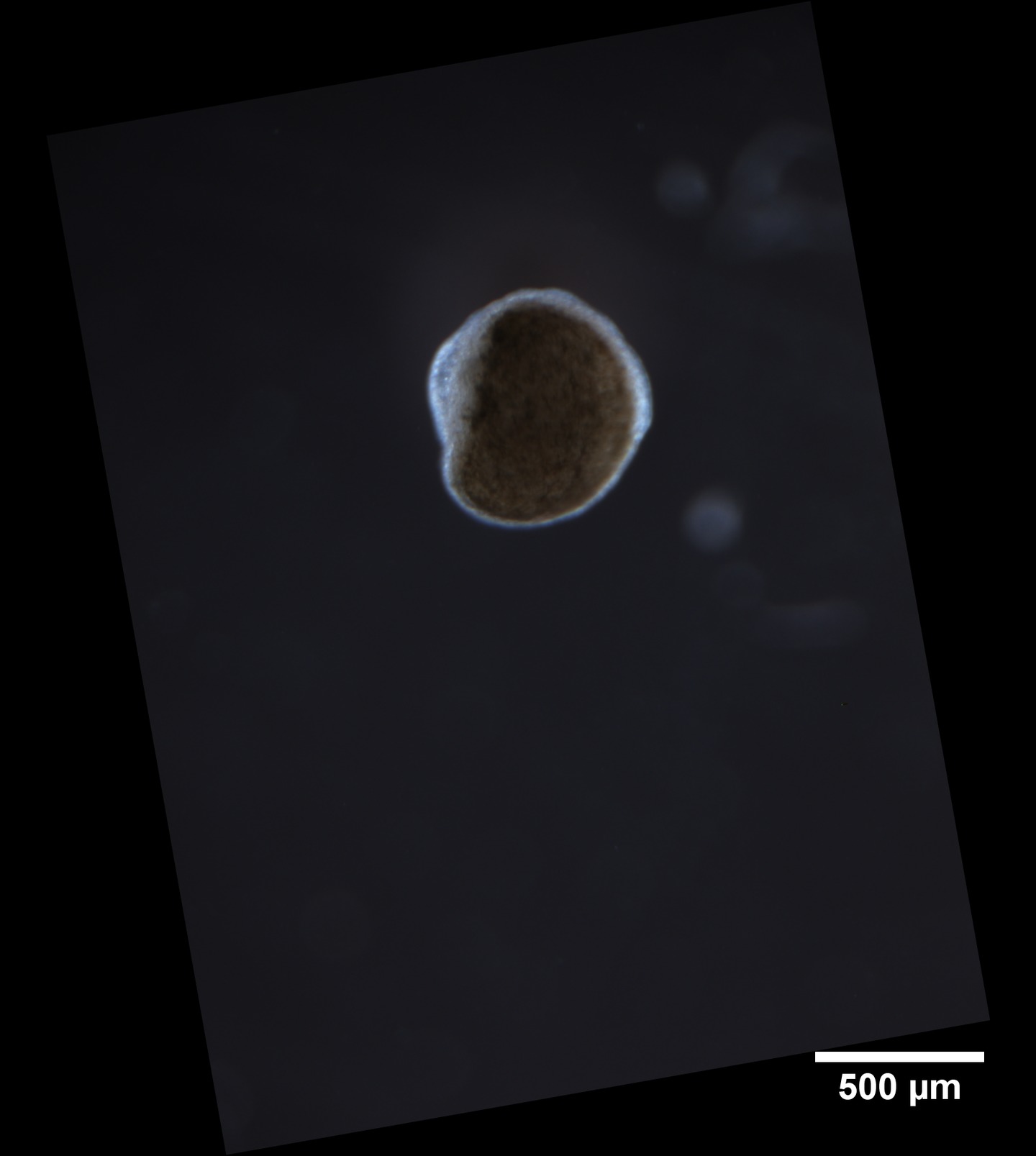

Supplement: S1 Dataset — This dataset contains brightfield image and corresponding synapsin stains for the VNC-free and VNC-containing small fragment cutting scenarios shown in Fig 6. Each image is labeled in the format “x_dpc_Sample_y_tn.jpg”, where “x” represents the number of days post cutting and “y” the replicate number. (ZIP) [file pcbi.1006904.s016.zip › smallfragments/VNC-free/Brigthfield_images/2 dpc_Sample 1_tn.jpg]

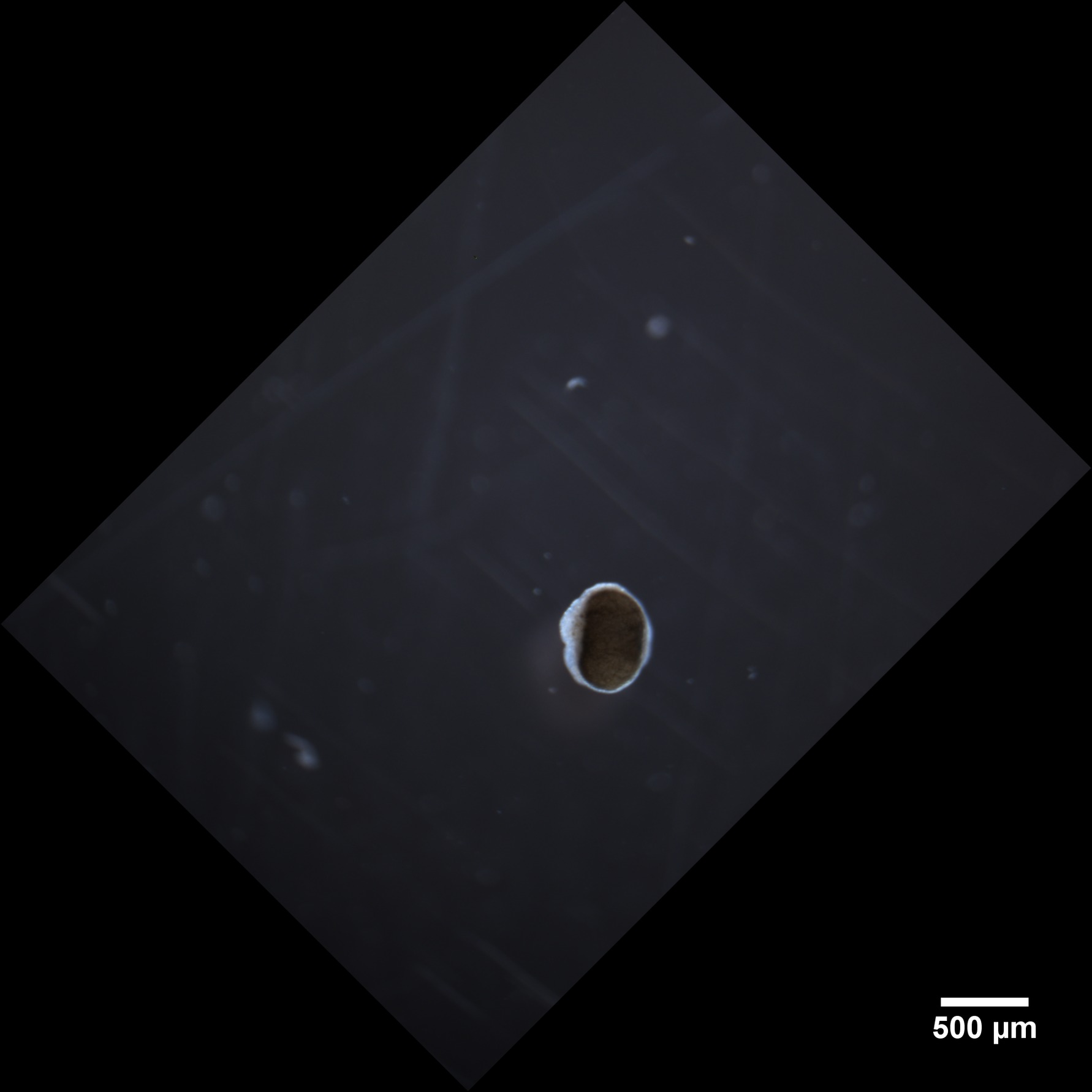

Supplement: S1 Dataset — This dataset contains brightfield image and corresponding synapsin stains for the VNC-free and VNC-containing small fragment cutting scenarios shown in Fig 6. Each image is labeled in the format “x_dpc_Sample_y_tn.jpg”, where “x” represents the number of days post cutting and “y” the replicate number. (ZIP) [file pcbi.1006904.s016.zip › smallfragments/VNC-free/Brigthfield_images/2 dpc_Sample 2_tn.jpg]

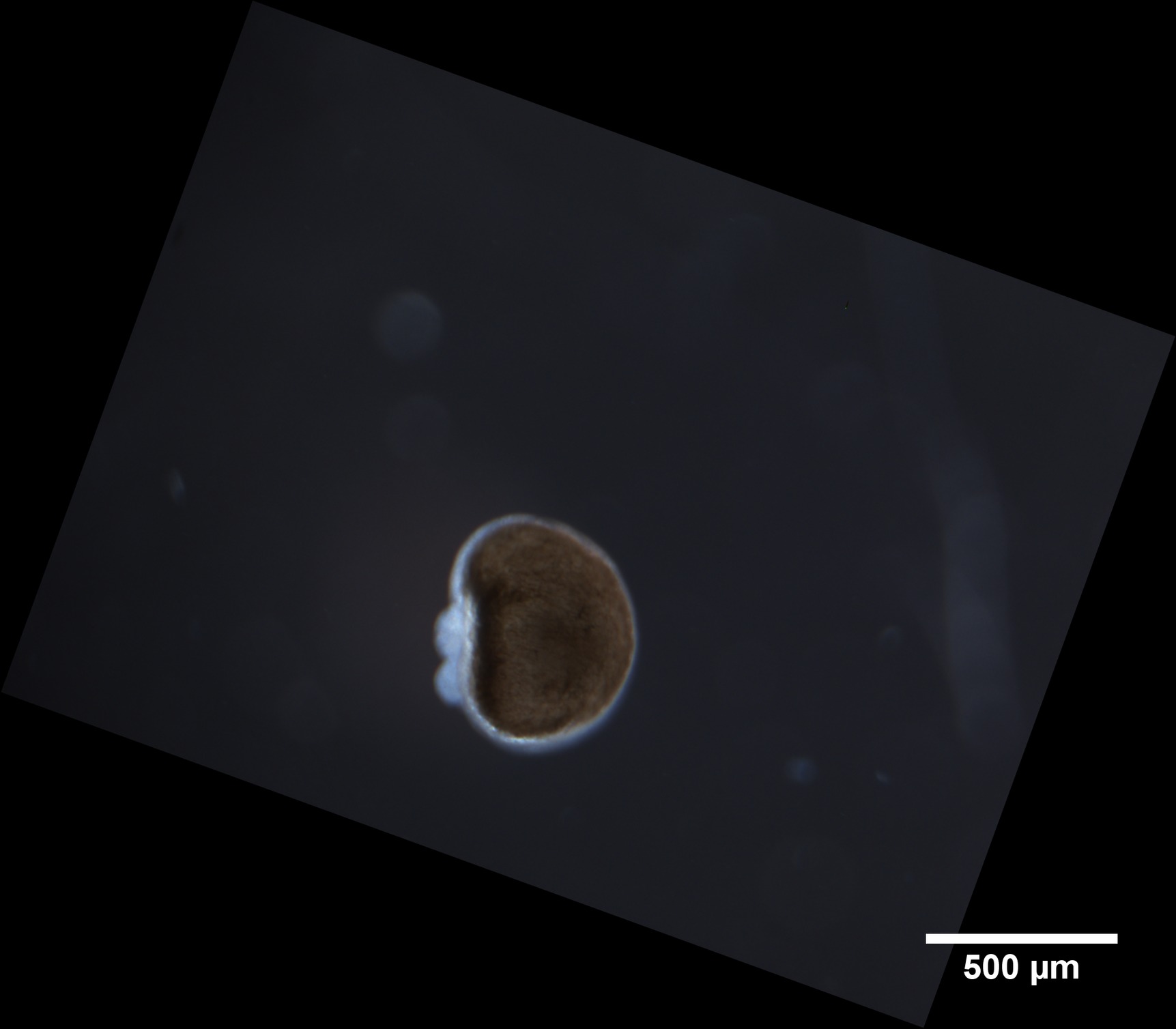

Supplement: S1 Dataset — This dataset contains brightfield image and corresponding synapsin stains for the VNC-free and VNC-containing small fragment cutting scenarios shown in Fig 6. Each image is labeled in the format “x_dpc_Sample_y_tn.jpg”, where “x” represents the number of days post cutting and “y” the replicate number. (ZIP) [file pcbi.1006904.s016.zip › smallfragments/VNC-free/Brigthfield_images/2 dpc_Sample 3_tn.jpg]

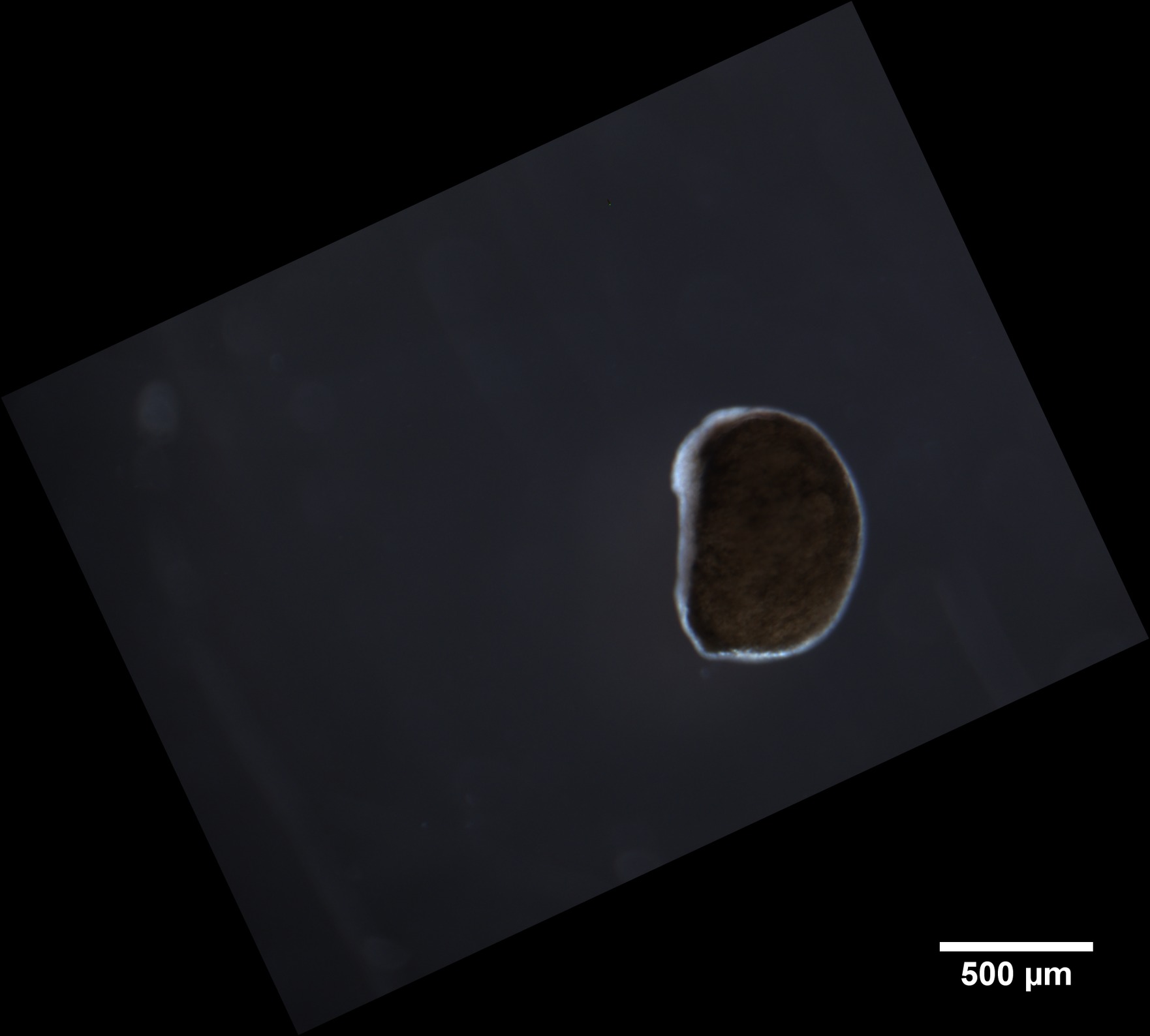

Supplement: S1 Dataset — This dataset contains brightfield image and corresponding synapsin stains for the VNC-free and VNC-containing small fragment cutting scenarios shown in Fig 6. Each image is labeled in the format “x_dpc_Sample_y_tn.jpg”, where “x” represents the number of days post cutting and “y” the replicate number. (ZIP) [file pcbi.1006904.s016.zip › smallfragments/VNC-free/Brigthfield_images/2 dpc_Sample 4_tn.jpg]

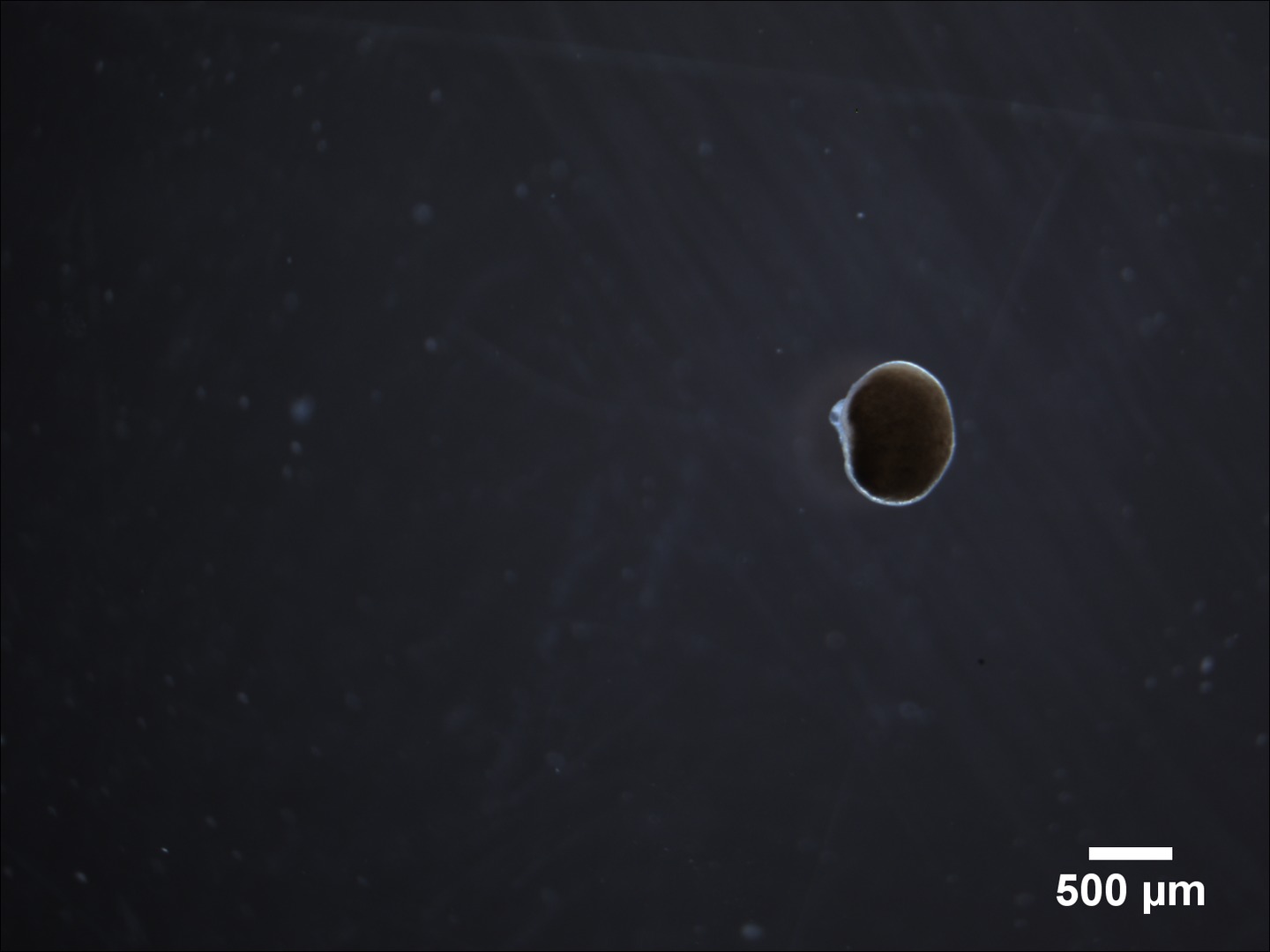

Supplement: S1 Dataset — This dataset contains brightfield image and corresponding synapsin stains for the VNC-free and VNC-containing small fragment cutting scenarios shown in Fig 6. Each image is labeled in the format “x_dpc_Sample_y_tn.jpg”, where “x” represents the number of days post cutting and “y” the replicate number. (ZIP) [file pcbi.1006904.s016.zip › smallfragments/VNC-free/Brigthfield_images/2 dpc_Sample 5_tn.jpg]

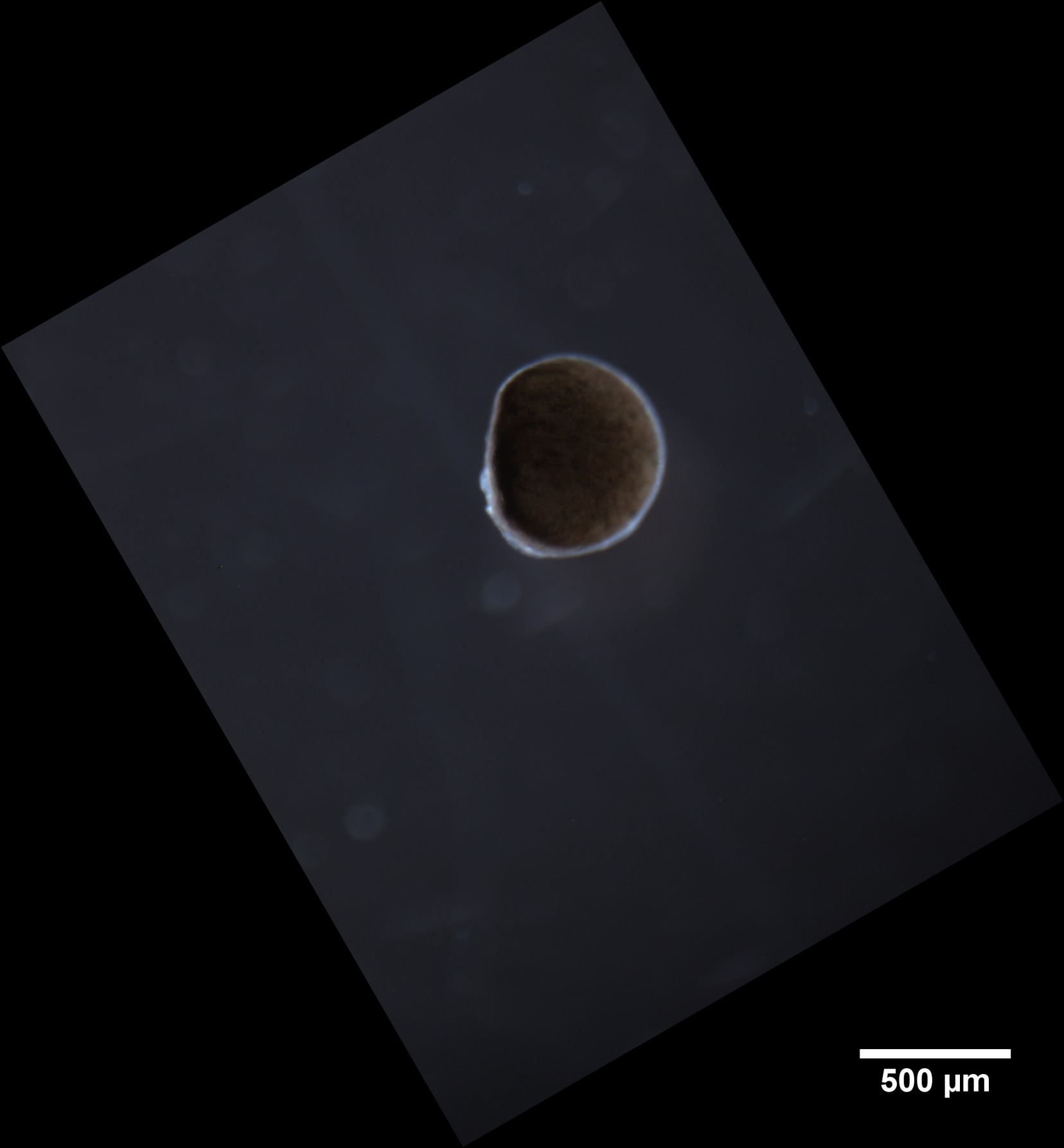

Supplement: S1 Dataset — This dataset contains brightfield image and corresponding synapsin stains for the VNC-free and VNC-containing small fragment cutting scenarios shown in Fig 6. Each image is labeled in the format “x_dpc_Sample_y_tn.jpg”, where “x” represents the number of days post cutting and “y” the replicate number. (ZIP) [file pcbi.1006904.s016.zip › smallfragments/VNC-free/Brigthfield_images/2 dpc_Sample 6_tn.jpg]

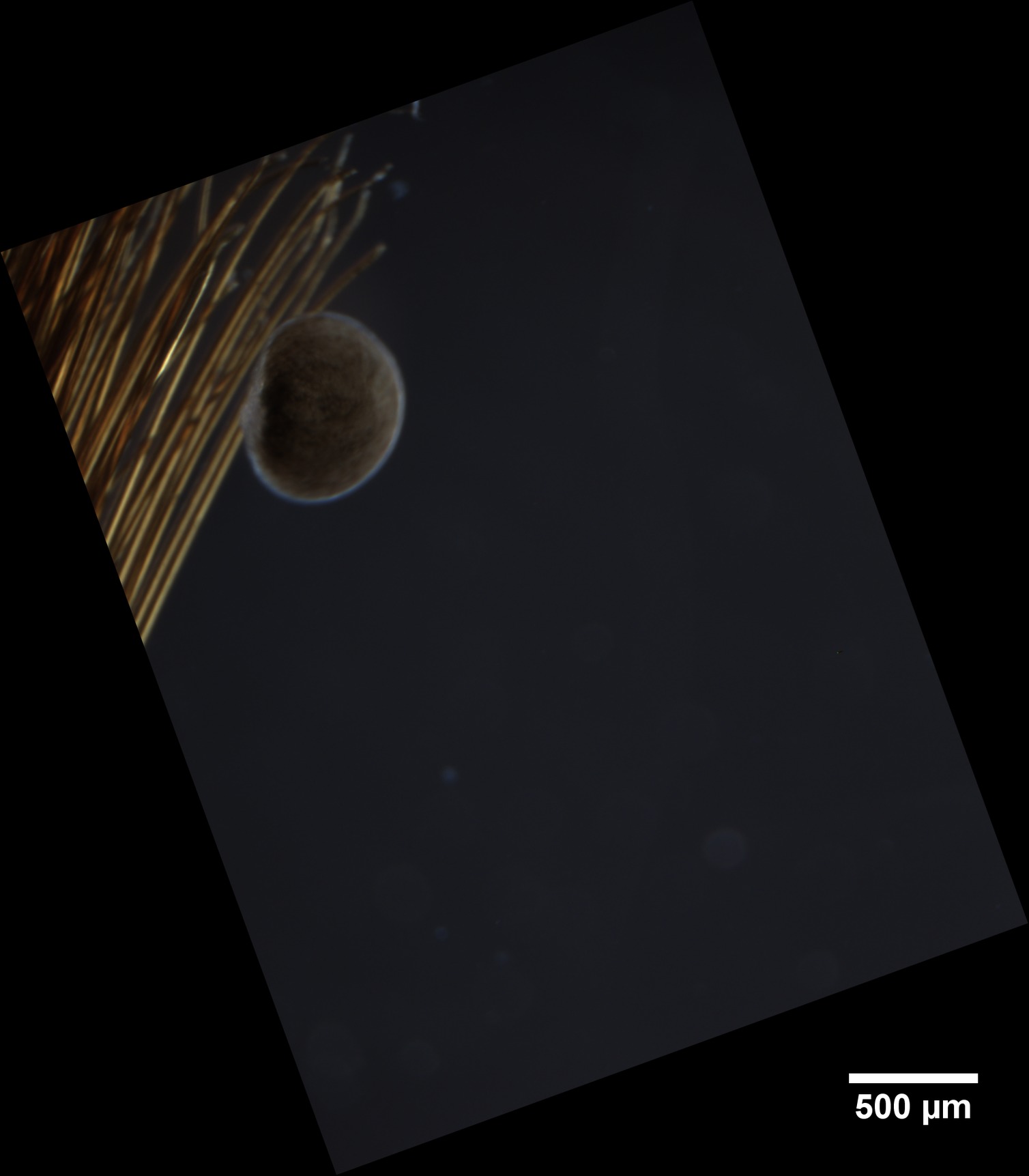

Supplement: S1 Dataset — This dataset contains brightfield image and corresponding synapsin stains for the VNC-free and VNC-containing small fragment cutting scenarios shown in Fig 6. Each image is labeled in the format “x_dpc_Sample_y_tn.jpg”, where “x” represents the number of days post cutting and “y” the replicate number. (ZIP) [file pcbi.1006904.s016.zip › smallfragments/VNC-free/Brigthfield_images/2 dpc_Sample 7_tn.jpg]

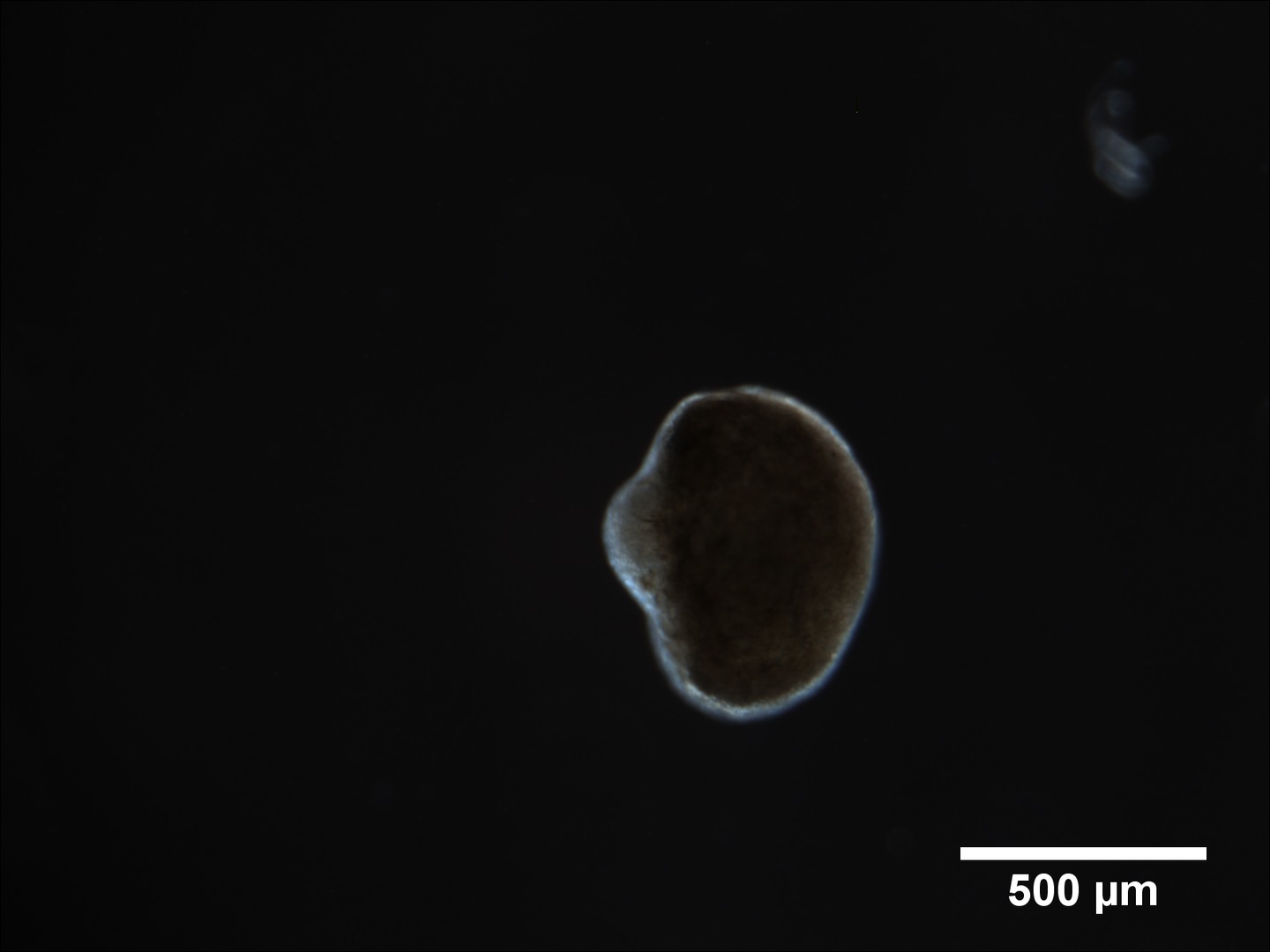

Supplement: S1 Dataset — This dataset contains brightfield image and corresponding synapsin stains for the VNC-free and VNC-containing small fragment cutting scenarios shown in Fig 6. Each image is labeled in the format “x_dpc_Sample_y_tn.jpg”, where “x” represents the number of days post cutting and “y” the replicate number. (ZIP) [file pcbi.1006904.s016.zip › smallfragments/VNC-free/Brigthfield_images/3 dpc_Sample 1_tn.jpg]

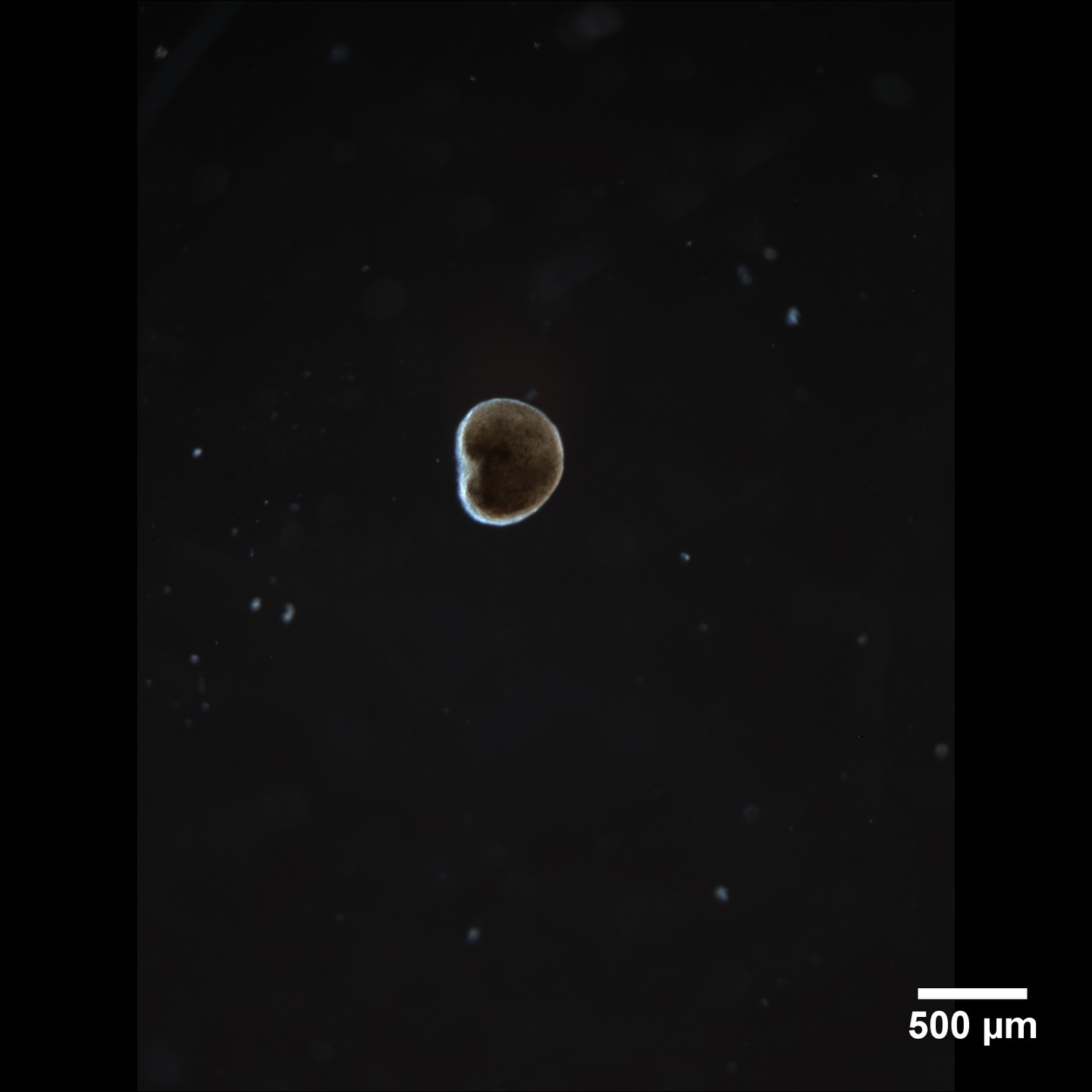

Supplement: S1 Dataset — This dataset contains brightfield image and corresponding synapsin stains for the VNC-free and VNC-containing small fragment cutting scenarios shown in Fig 6. Each image is labeled in the format “x_dpc_Sample_y_tn.jpg”, where “x” represents the number of days post cutting and “y” the replicate number. (ZIP) [file pcbi.1006904.s016.zip › smallfragments/VNC-free/Brigthfield_images/3 dpc_Sample 3_tn.jpg]

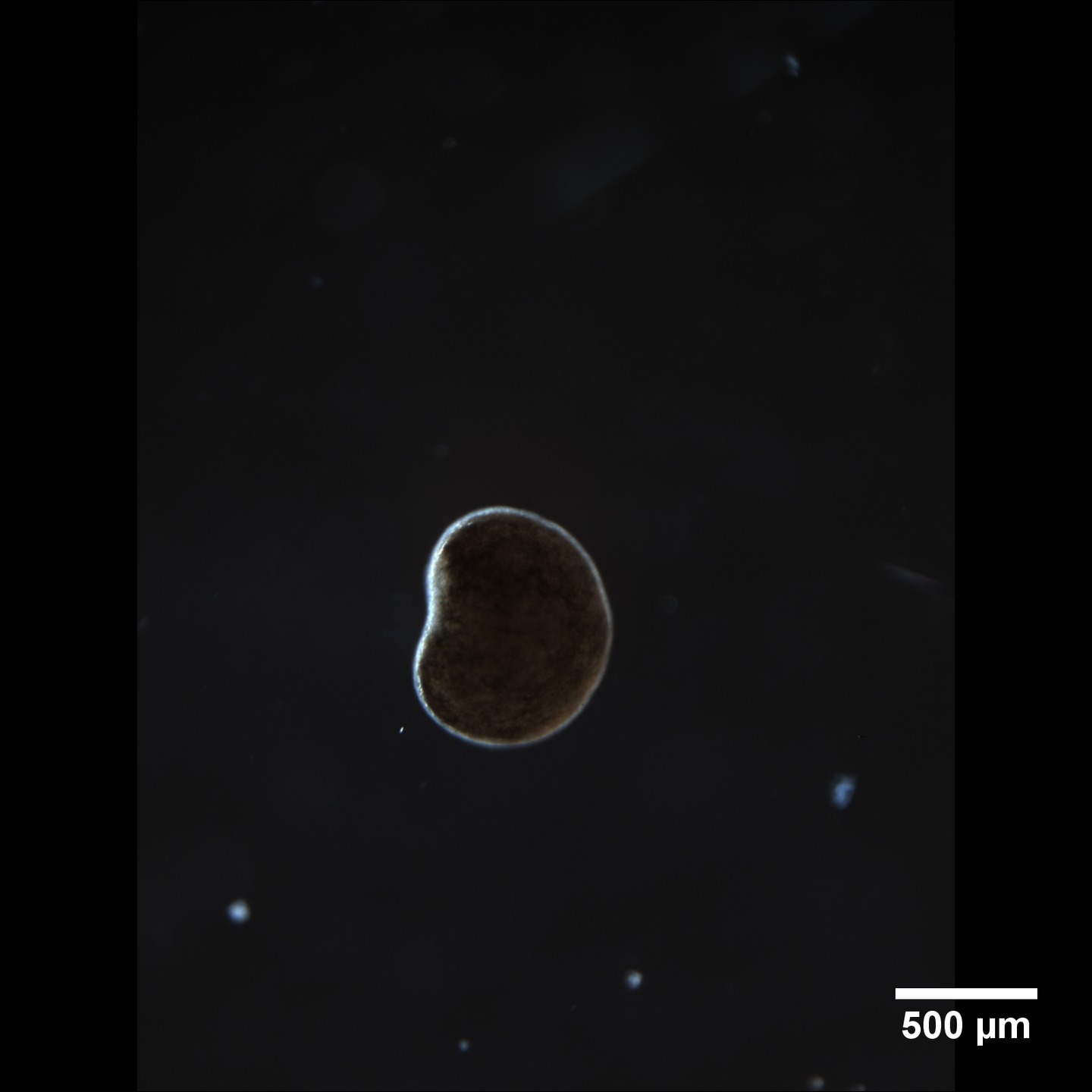

Supplement: S1 Dataset — This dataset contains brightfield image and corresponding synapsin stains for the VNC-free and VNC-containing small fragment cutting scenarios shown in Fig 6. Each image is labeled in the format “x_dpc_Sample_y_tn.jpg”, where “x” represents the number of days post cutting and “y” the replicate number. (ZIP) [file pcbi.1006904.s016.zip › smallfragments/VNC-free/Brigthfield_images/3 dpc_Sample 4_tn.jpg]

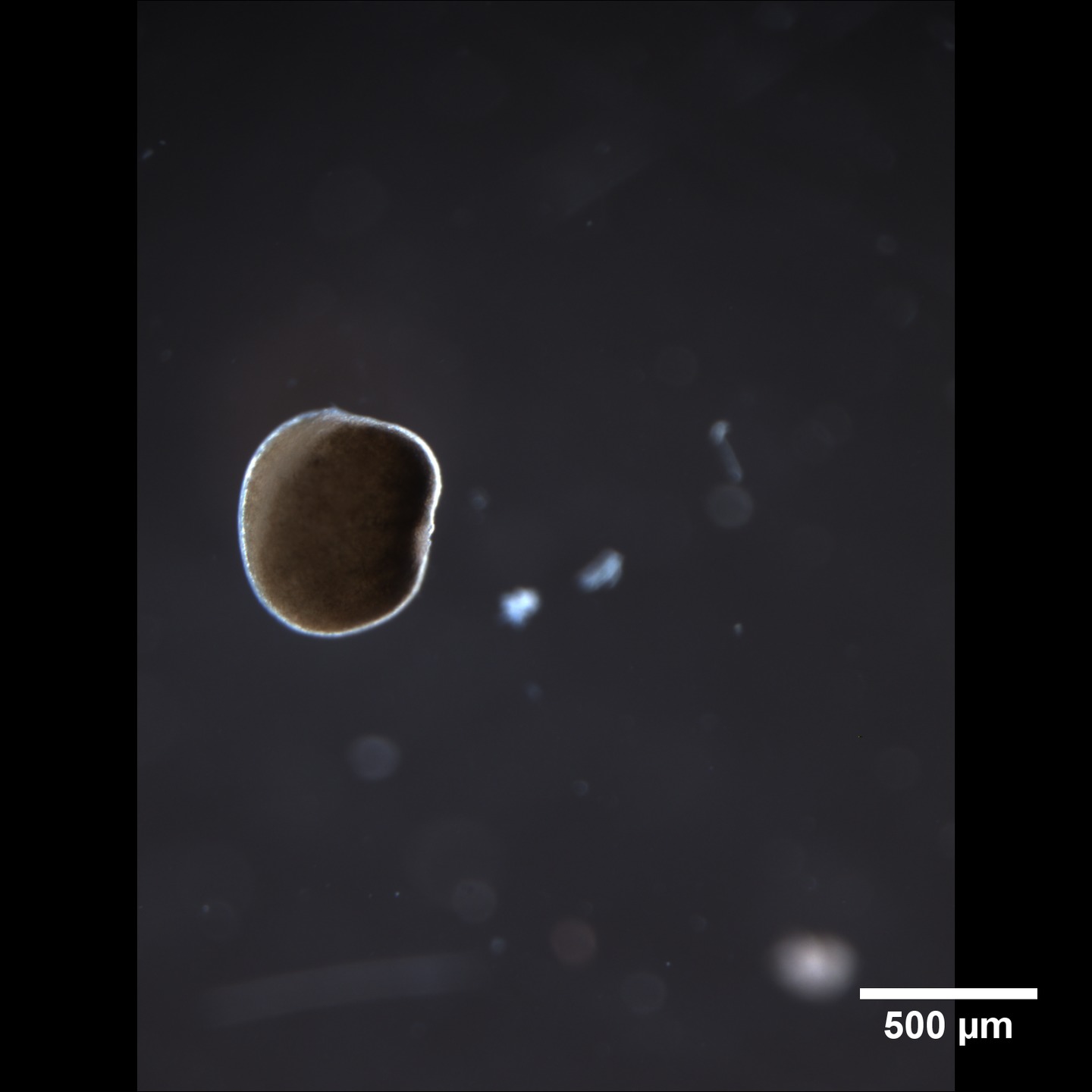

Supplement: S1 Dataset — This dataset contains brightfield image and corresponding synapsin stains for the VNC-free and VNC-containing small fragment cutting scenarios shown in Fig 6. Each image is labeled in the format “x_dpc_Sample_y_tn.jpg”, where “x” represents the number of days post cutting and “y” the replicate number. (ZIP) [file pcbi.1006904.s016.zip › smallfragments/VNC-free/Brigthfield_images/3 dpc_Sample 5_tn.jpg]

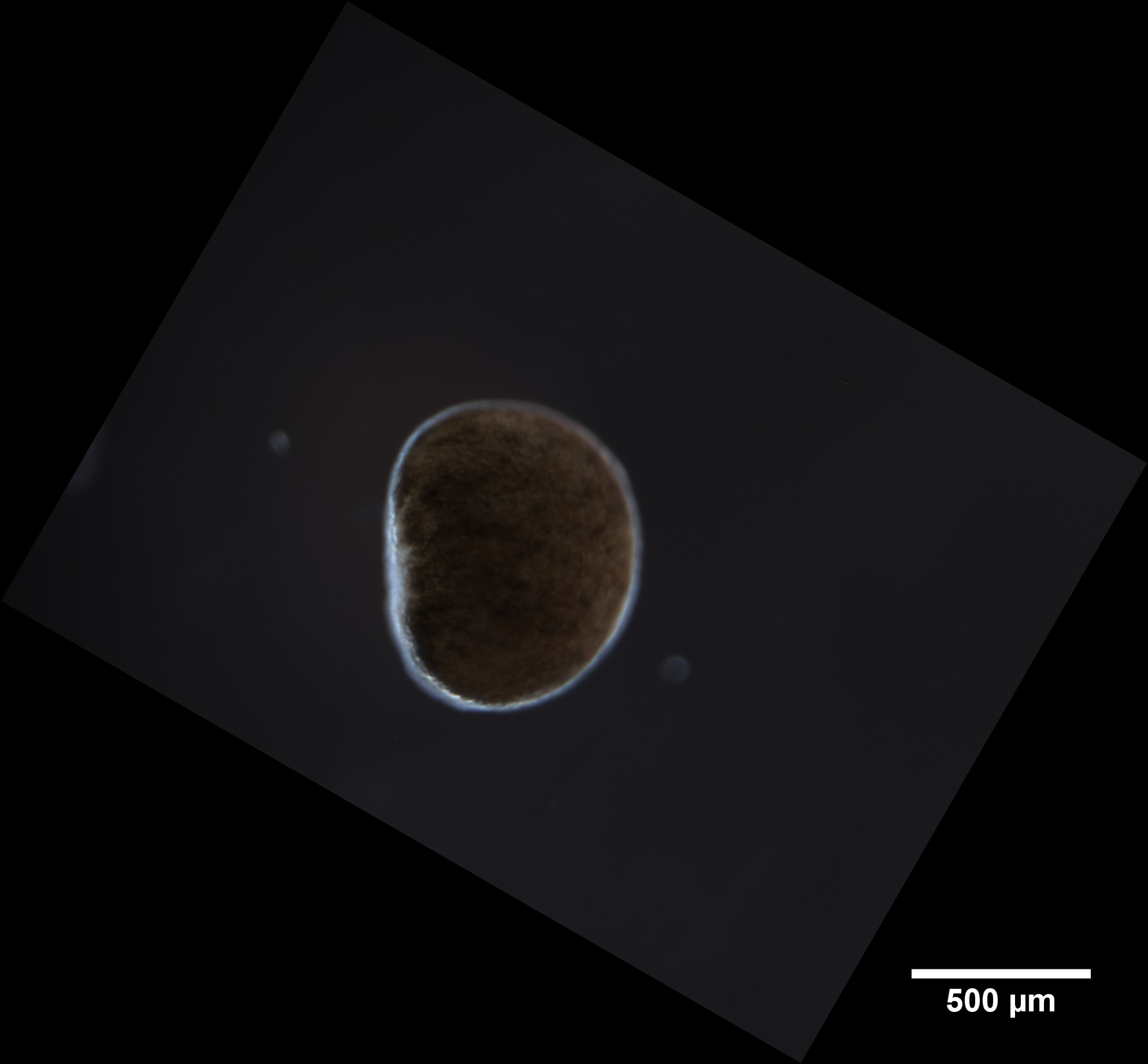

Supplement: S1 Dataset — This dataset contains brightfield image and corresponding synapsin stains for the VNC-free and VNC-containing small fragment cutting scenarios shown in Fig 6. Each image is labeled in the format “x_dpc_Sample_y_tn.jpg”, where “x” represents the number of days post cutting and “y” the replicate number. (ZIP) [file pcbi.1006904.s016.zip › smallfragments/VNC-free/Brigthfield_images/3 dpc_Sample 6_tn.jpg]

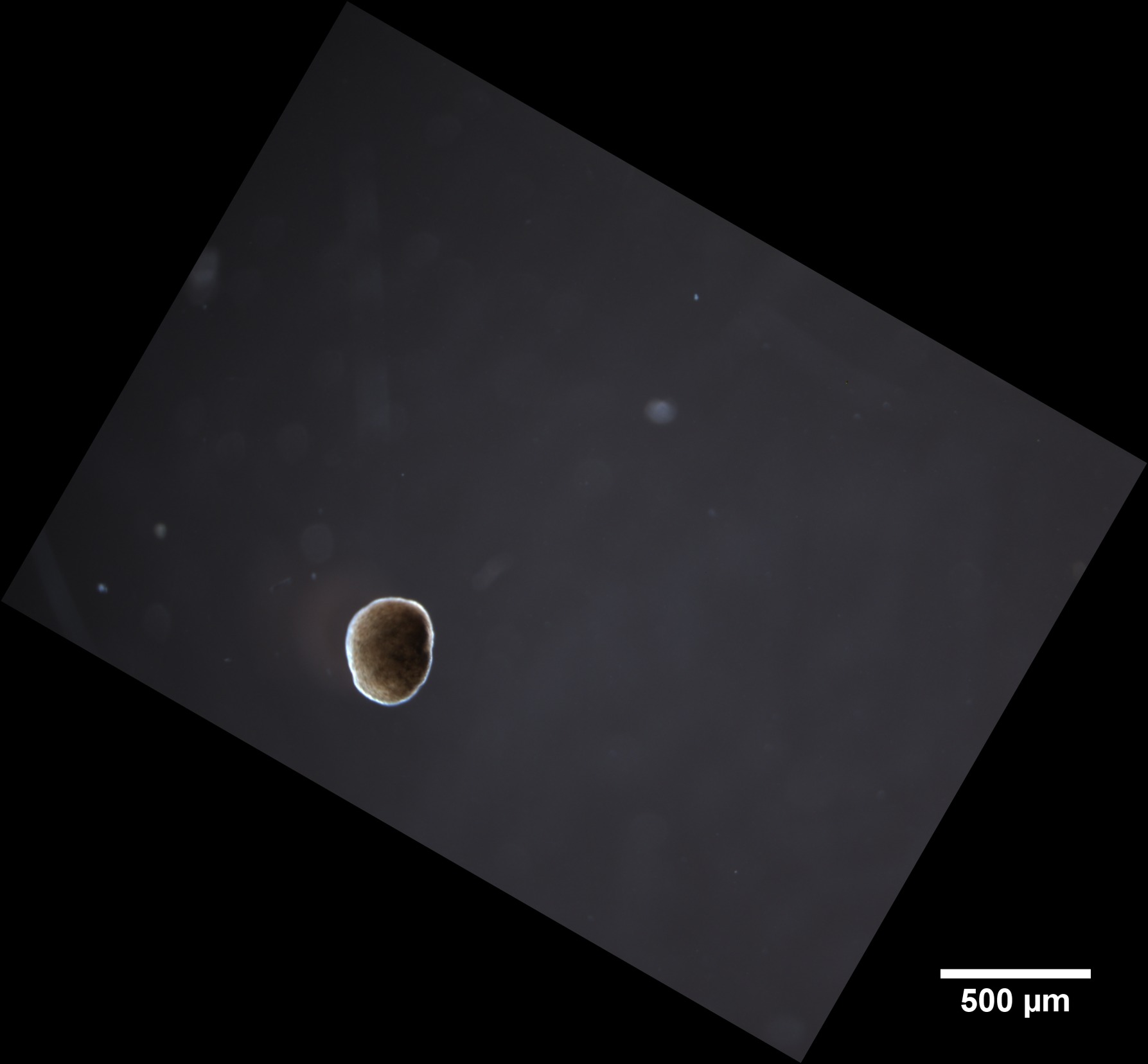

Supplement: S1 Dataset — This dataset contains brightfield image and corresponding synapsin stains for the VNC-free and VNC-containing small fragment cutting scenarios shown in Fig 6. Each image is labeled in the format “x_dpc_Sample_y_tn.jpg”, where “x” represents the number of days post cutting and “y” the replicate number. (ZIP) [file pcbi.1006904.s016.zip › smallfragments/VNC-free/Brigthfield_images/3 dpc_Sample 7_tn.jpg]

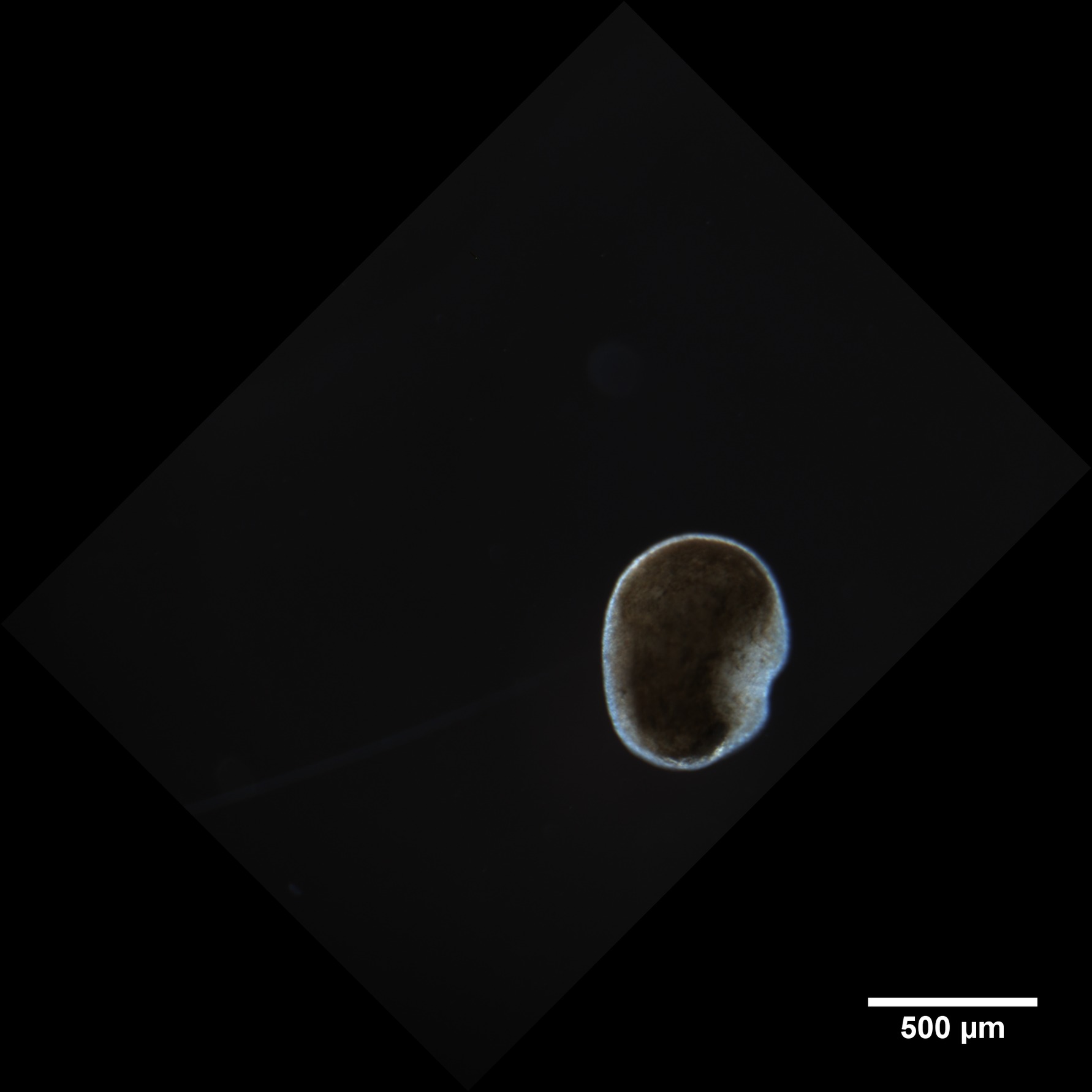

Supplement: S1 Dataset — This dataset contains brightfield image and corresponding synapsin stains for the VNC-free and VNC-containing small fragment cutting scenarios shown in Fig 6. Each image is labeled in the format “x_dpc_Sample_y_tn.jpg”, where “x” represents the number of days post cutting and “y” the replicate number. (ZIP) [file pcbi.1006904.s016.zip › smallfragments/VNC-free/Brigthfield_images/4 dpc_Sample 1_tn.jpg]

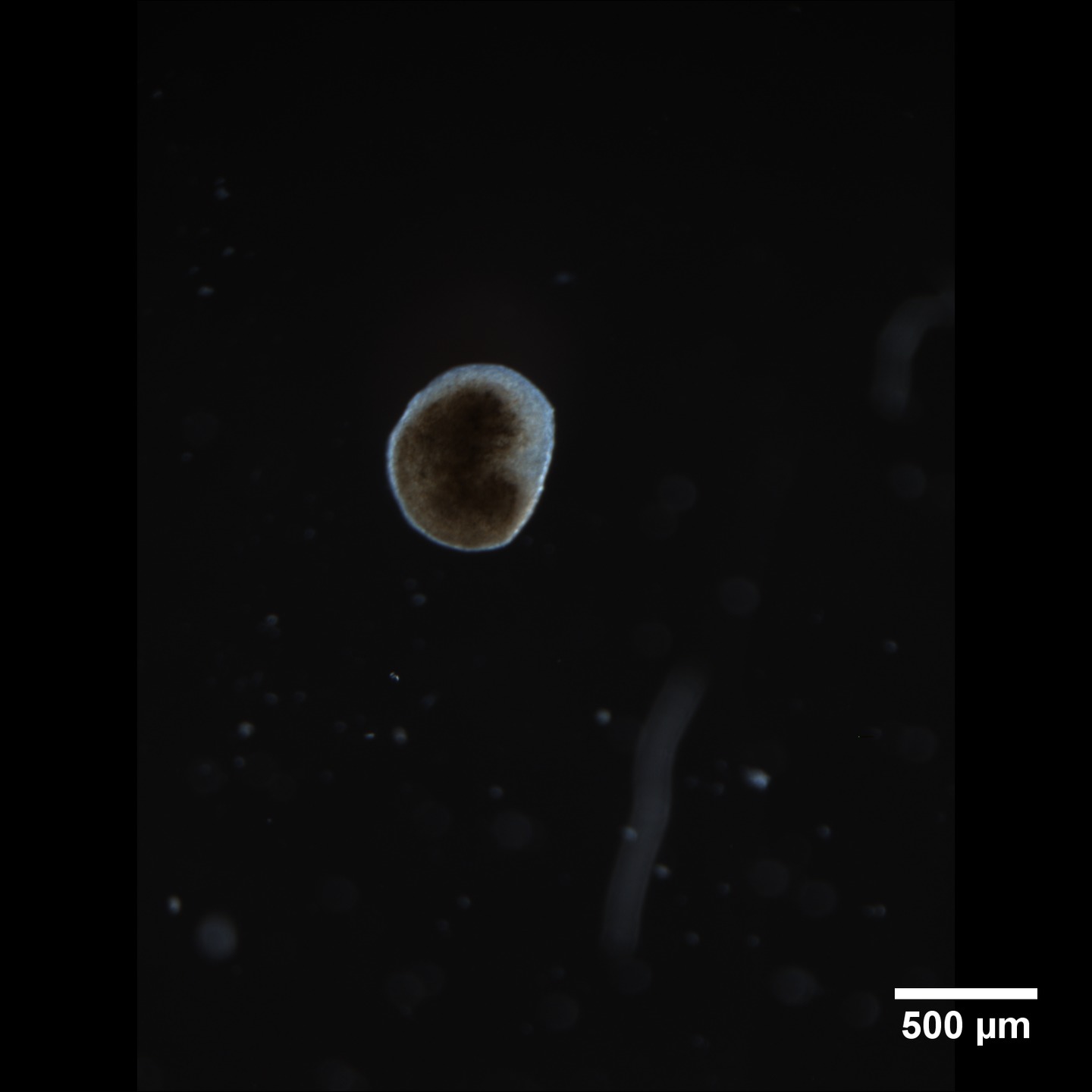

Supplement: S1 Dataset — This dataset contains brightfield image and corresponding synapsin stains for the VNC-free and VNC-containing small fragment cutting scenarios shown in Fig 6. Each image is labeled in the format “x_dpc_Sample_y_tn.jpg”, where “x” represents the number of days post cutting and “y” the replicate number. (ZIP) [file pcbi.1006904.s016.zip › smallfragments/VNC-free/Brigthfield_images/4 dpc_Sample 3_tn.jpg]

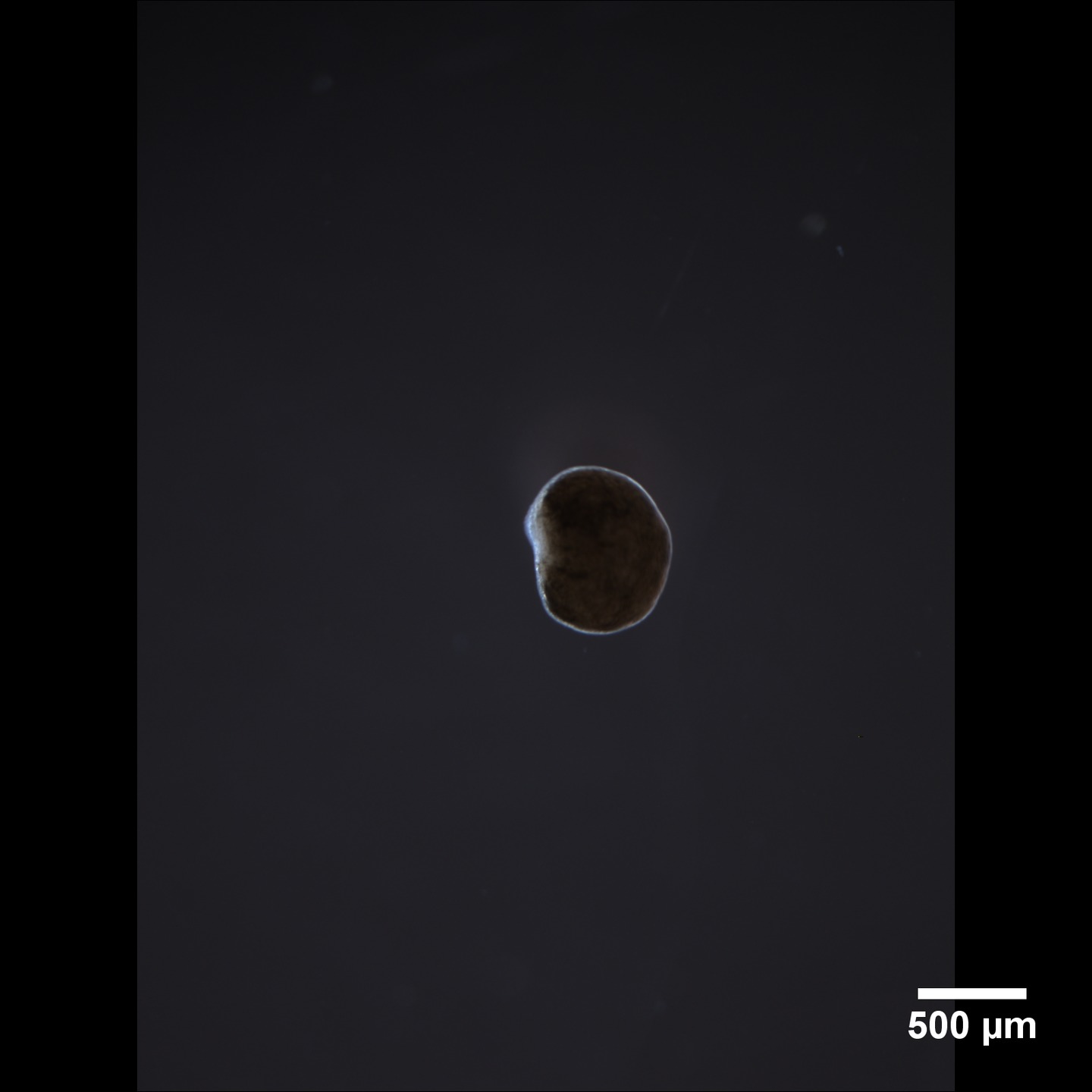

Supplement: S1 Dataset — This dataset contains brightfield image and corresponding synapsin stains for the VNC-free and VNC-containing small fragment cutting scenarios shown in Fig 6. Each image is labeled in the format “x_dpc_Sample_y_tn.jpg”, where “x” represents the number of days post cutting and “y” the replicate number. (ZIP) [file pcbi.1006904.s016.zip › smallfragments/VNC-free/Brigthfield_images/4 dpc_Sample 4_tn.jpg]

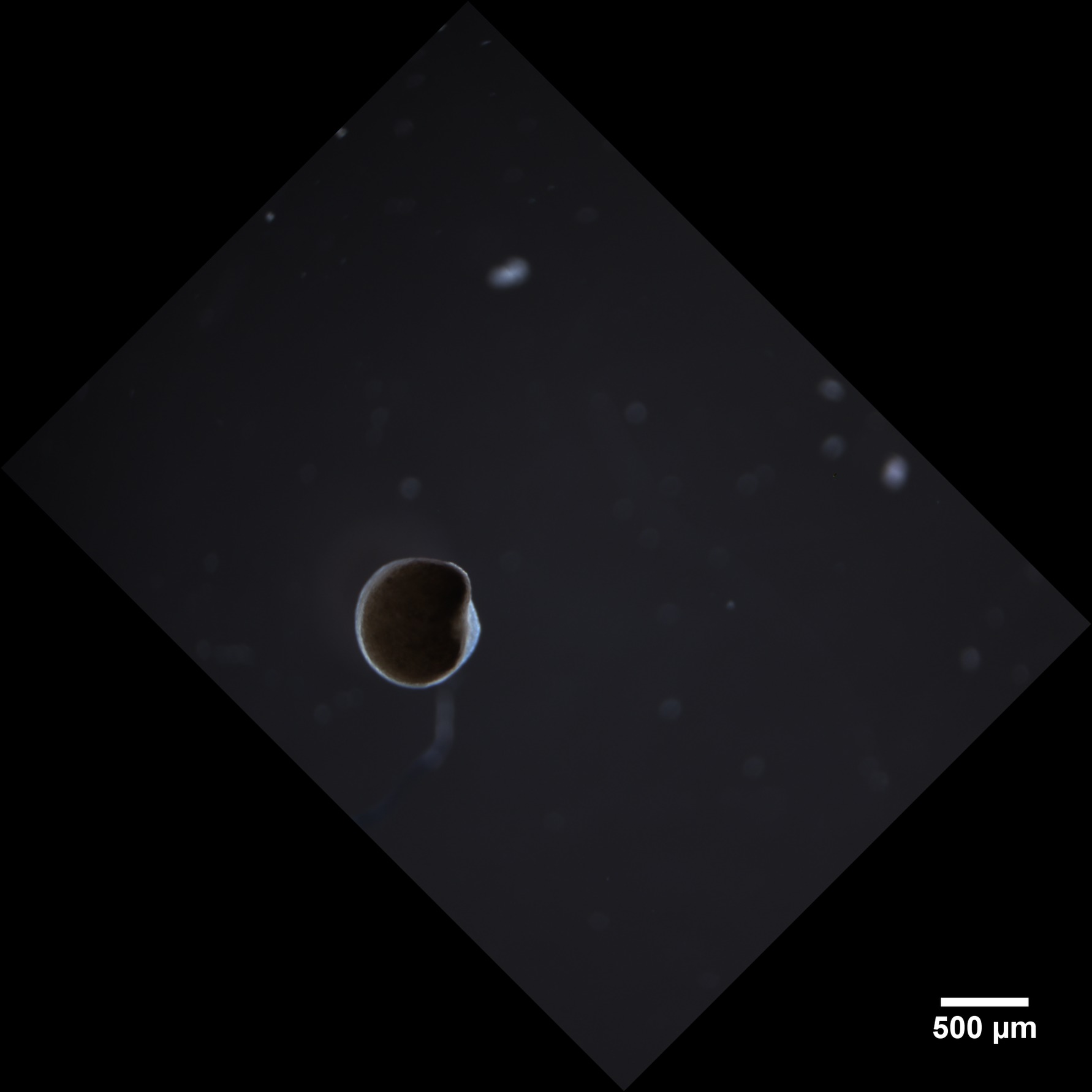

Supplement: S1 Dataset — This dataset contains brightfield image and corresponding synapsin stains for the VNC-free and VNC-containing small fragment cutting scenarios shown in Fig 6. Each image is labeled in the format “x_dpc_Sample_y_tn.jpg”, where “x” represents the number of days post cutting and “y” the replicate number. (ZIP) [file pcbi.1006904.s016.zip › smallfragments/VNC-free/Brigthfield_images/4 dpc_Sample 5_tn.jpg]

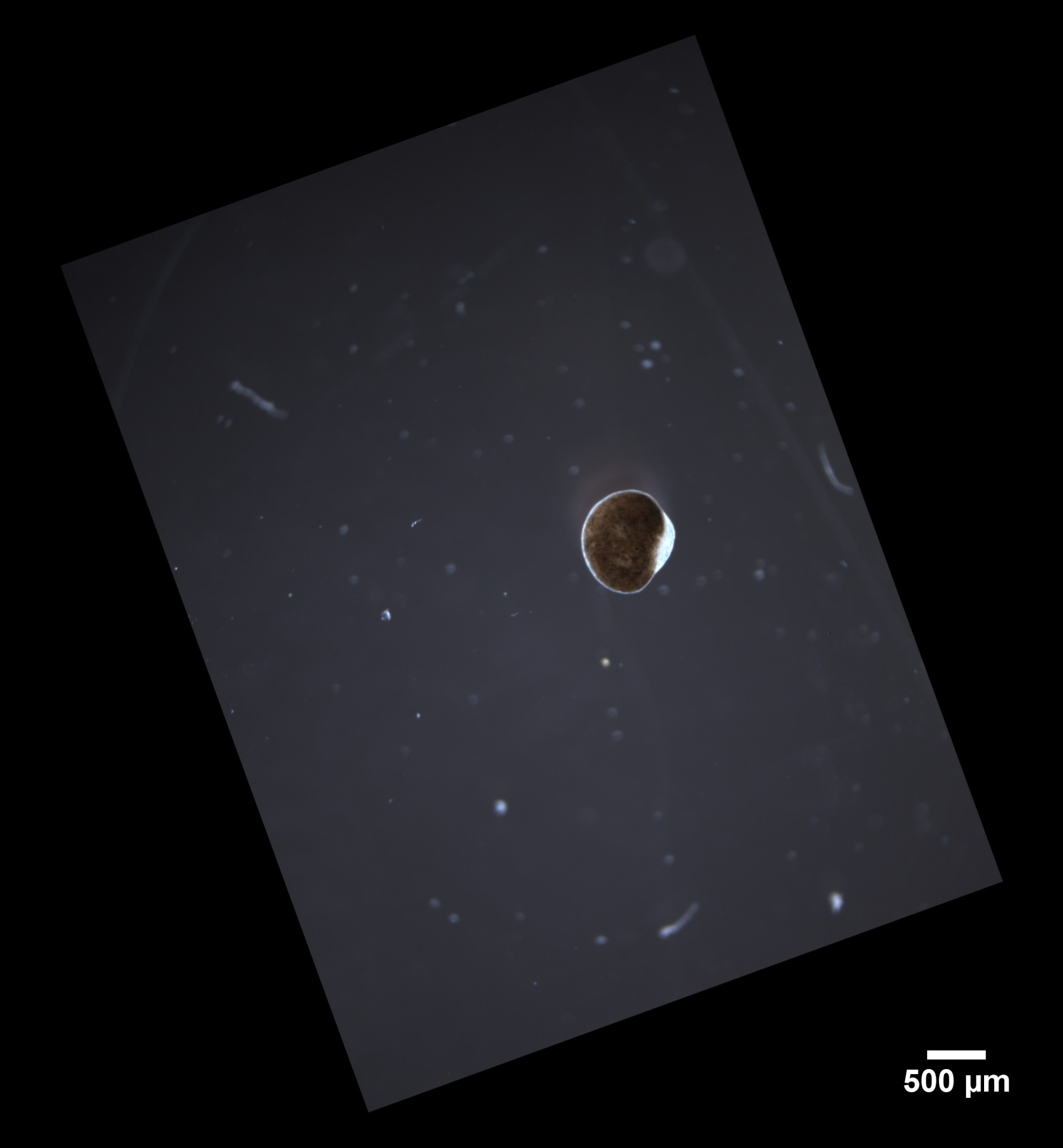

Supplement: S1 Dataset — This dataset contains brightfield image and corresponding synapsin stains for the VNC-free and VNC-containing small fragment cutting scenarios shown in Fig 6. Each image is labeled in the format “x_dpc_Sample_y_tn.jpg”, where “x” represents the number of days post cutting and “y” the replicate number. (ZIP) [file pcbi.1006904.s016.zip › smallfragments/VNC-free/Brigthfield_images/4 dpc_Sample 6_tn.jpg]

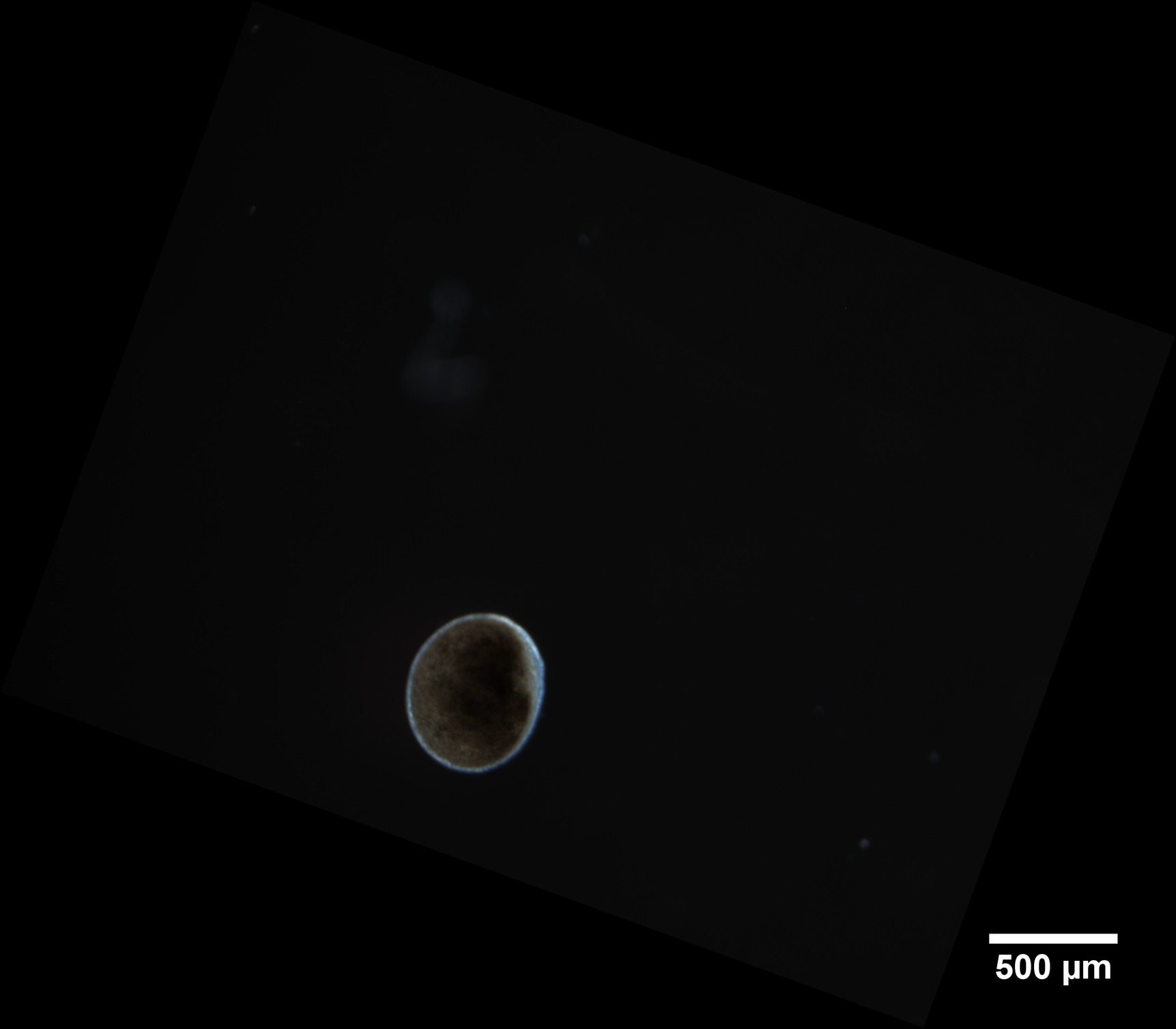

Supplement: S1 Dataset — This dataset contains brightfield image and corresponding synapsin stains for the VNC-free and VNC-containing small fragment cutting scenarios shown in Fig 6. Each image is labeled in the format “x_dpc_Sample_y_tn.jpg”, where “x” represents the number of days post cutting and “y” the replicate number. (ZIP) [file pcbi.1006904.s016.zip › smallfragments/VNC-free/Brigthfield_images/4 dpc_Sample 7_tn.jpg]

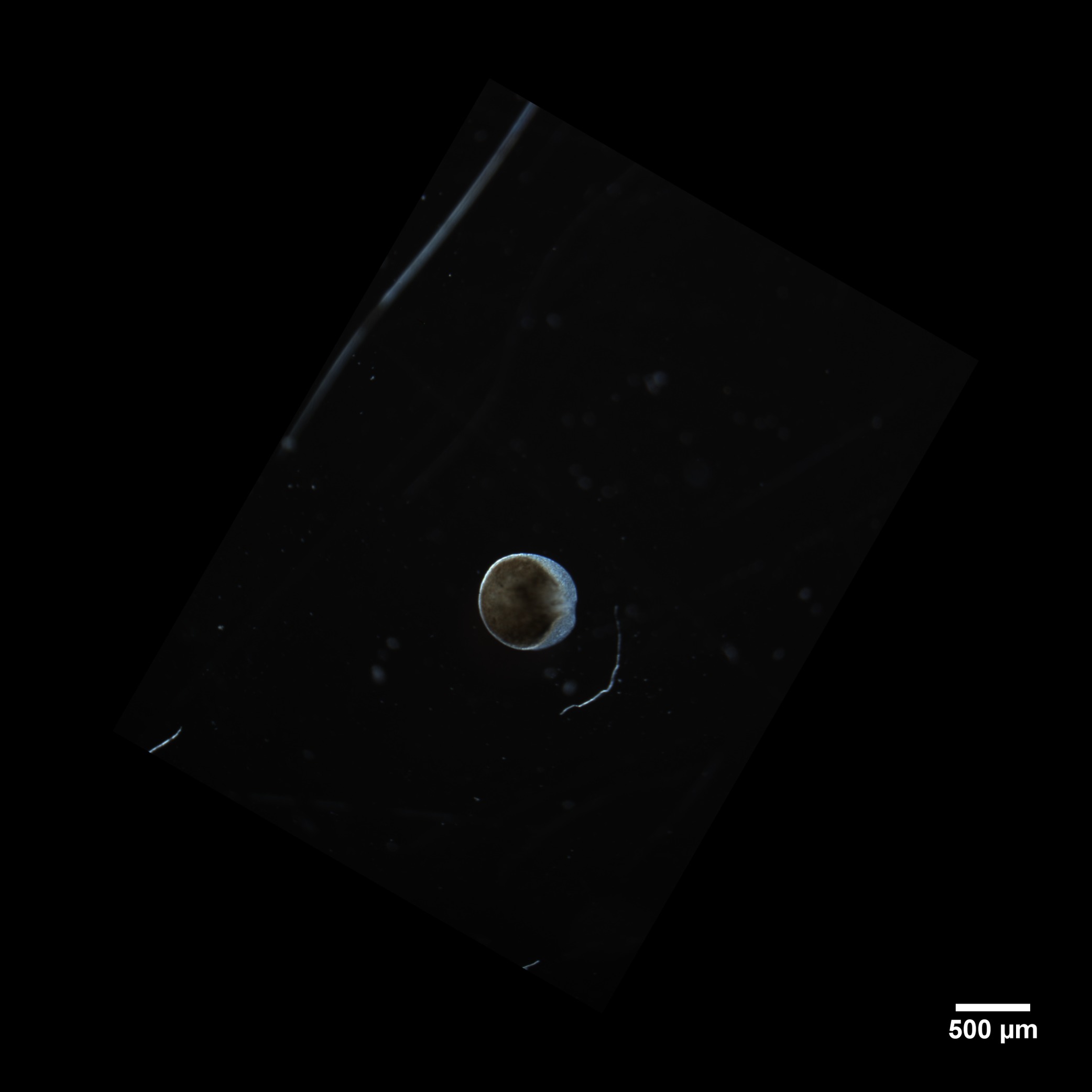

Supplement: S1 Dataset — This dataset contains brightfield image and corresponding synapsin stains for the VNC-free and VNC-containing small fragment cutting scenarios shown in Fig 6. Each image is labeled in the format “x_dpc_Sample_y_tn.jpg”, where “x” represents the number of days post cutting and “y” the replicate number. (ZIP) [file pcbi.1006904.s016.zip › smallfragments/VNC-free/Brigthfield_images/5 dpc_Sample 1_tn.jpg]

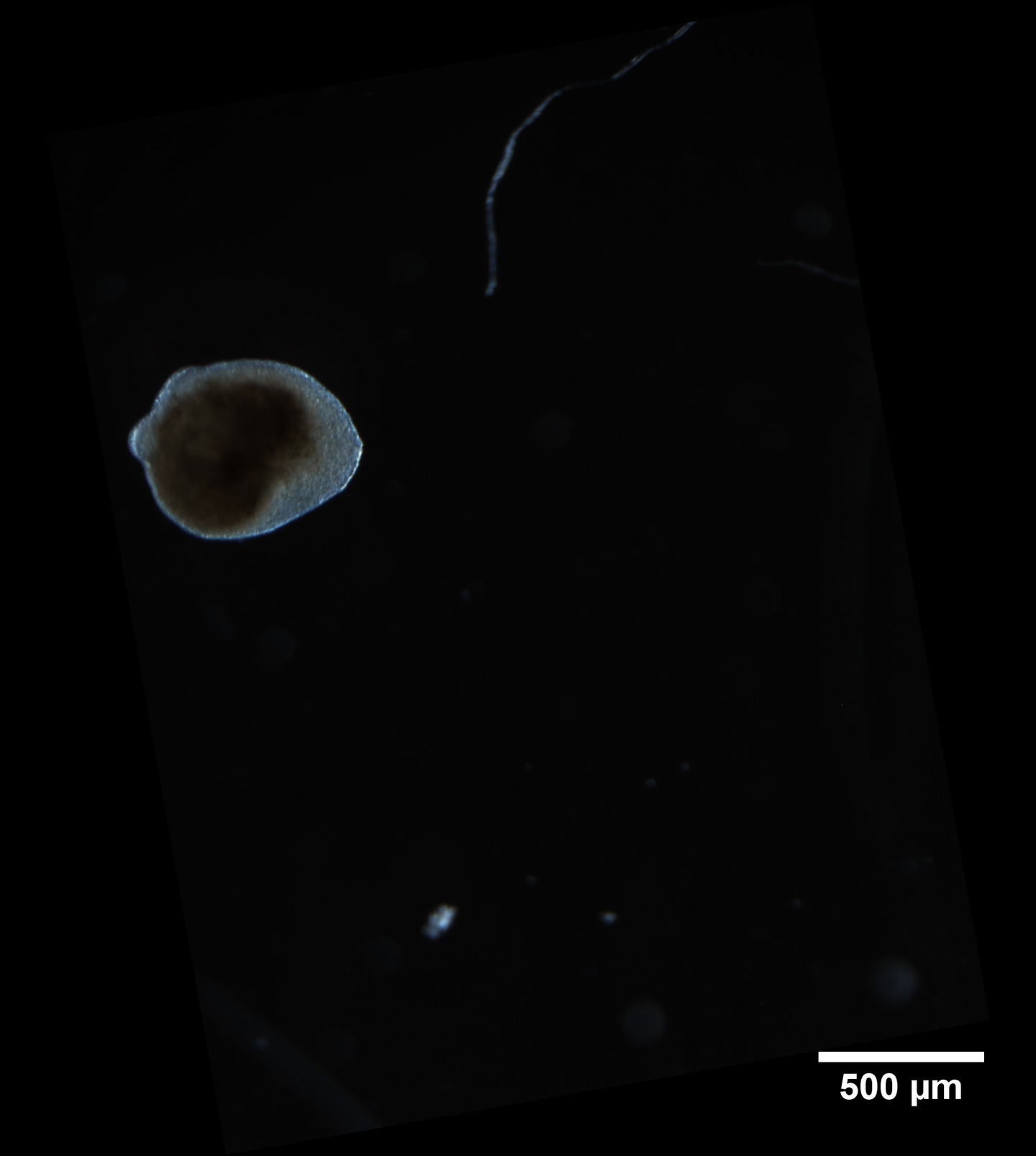

Supplement: S1 Dataset — This dataset contains brightfield image and corresponding synapsin stains for the VNC-free and VNC-containing small fragment cutting scenarios shown in Fig 6. Each image is labeled in the format “x_dpc_Sample_y_tn.jpg”, where “x” represents the number of days post cutting and “y” the replicate number. (ZIP) [file pcbi.1006904.s016.zip › smallfragments/VNC-free/Brigthfield_images/5 dpc_Sample 3_tn.jpg]

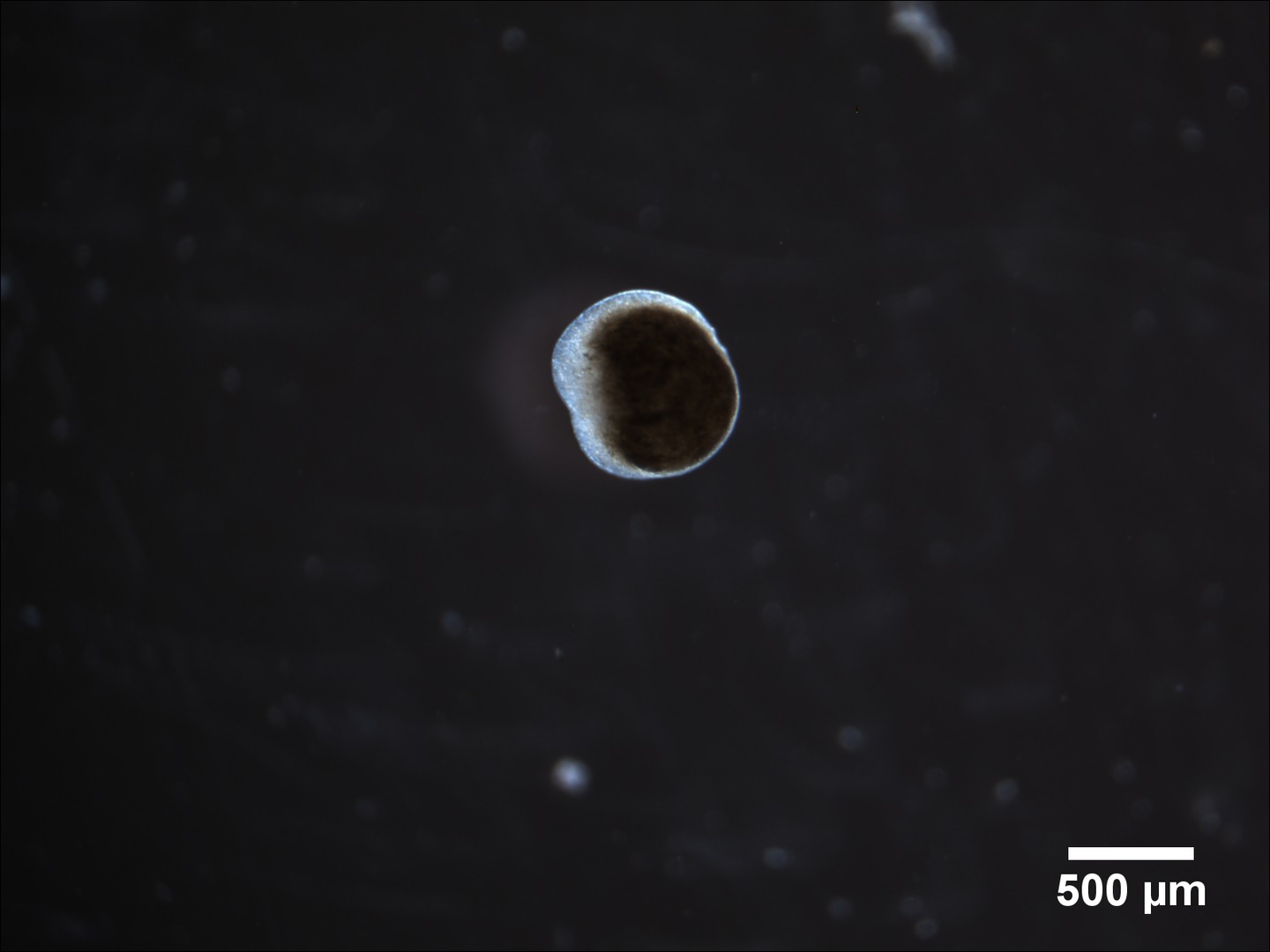

Supplement: S1 Dataset — This dataset contains brightfield image and corresponding synapsin stains for the VNC-free and VNC-containing small fragment cutting scenarios shown in Fig 6. Each image is labeled in the format “x_dpc_Sample_y_tn.jpg”, where “x” represents the number of days post cutting and “y” the replicate number. (ZIP) [file pcbi.1006904.s016.zip › smallfragments/VNC-free/Brigthfield_images/5 dpc_Sample 4_tn.jpg]

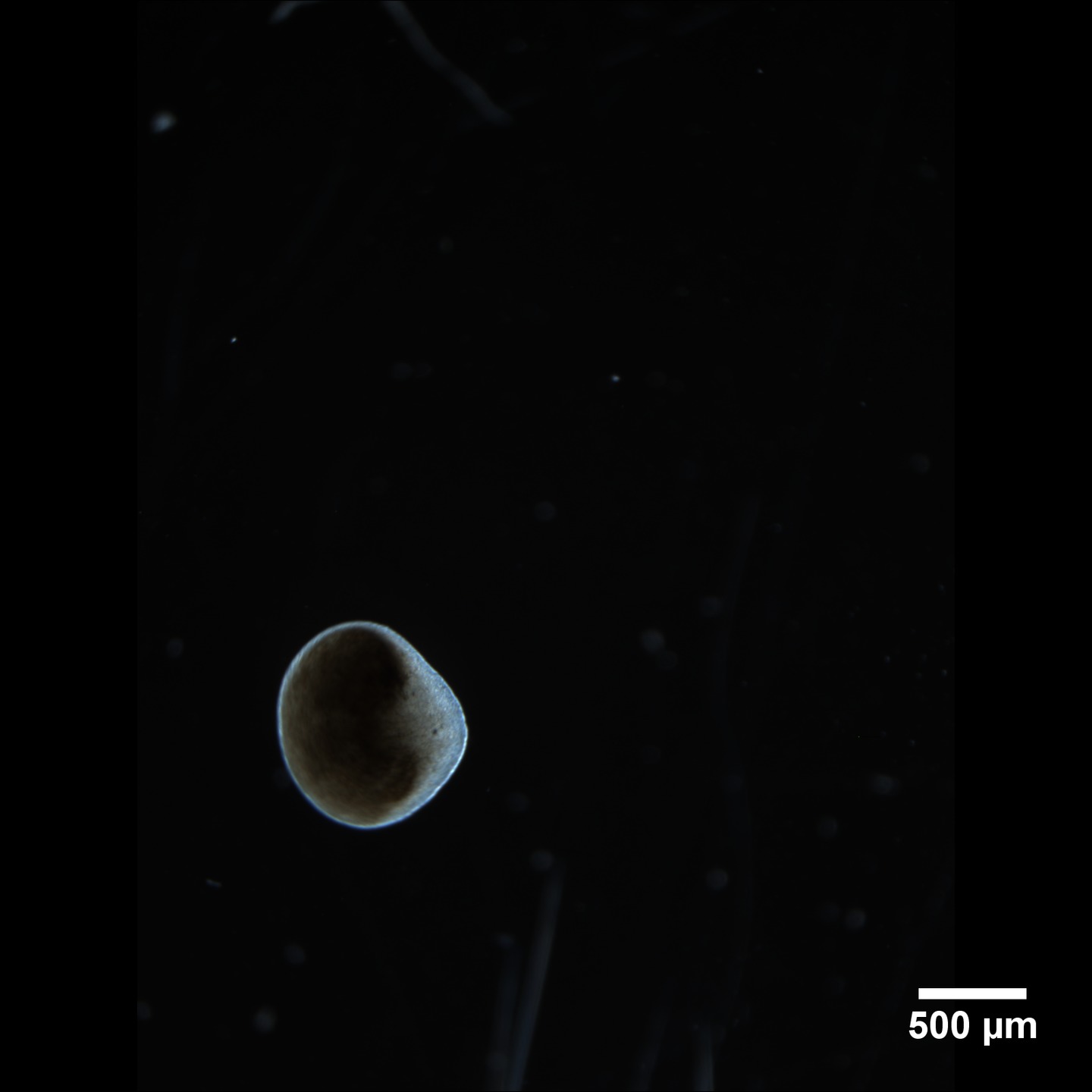

Supplement: S1 Dataset — This dataset contains brightfield image and corresponding synapsin stains for the VNC-free and VNC-containing small fragment cutting scenarios shown in Fig 6. Each image is labeled in the format “x_dpc_Sample_y_tn.jpg”, where “x” represents the number of days post cutting and “y” the replicate number. (ZIP) [file pcbi.1006904.s016.zip › smallfragments/VNC-free/Brigthfield_images/5 dpc_Sample 5_tn.jpg]

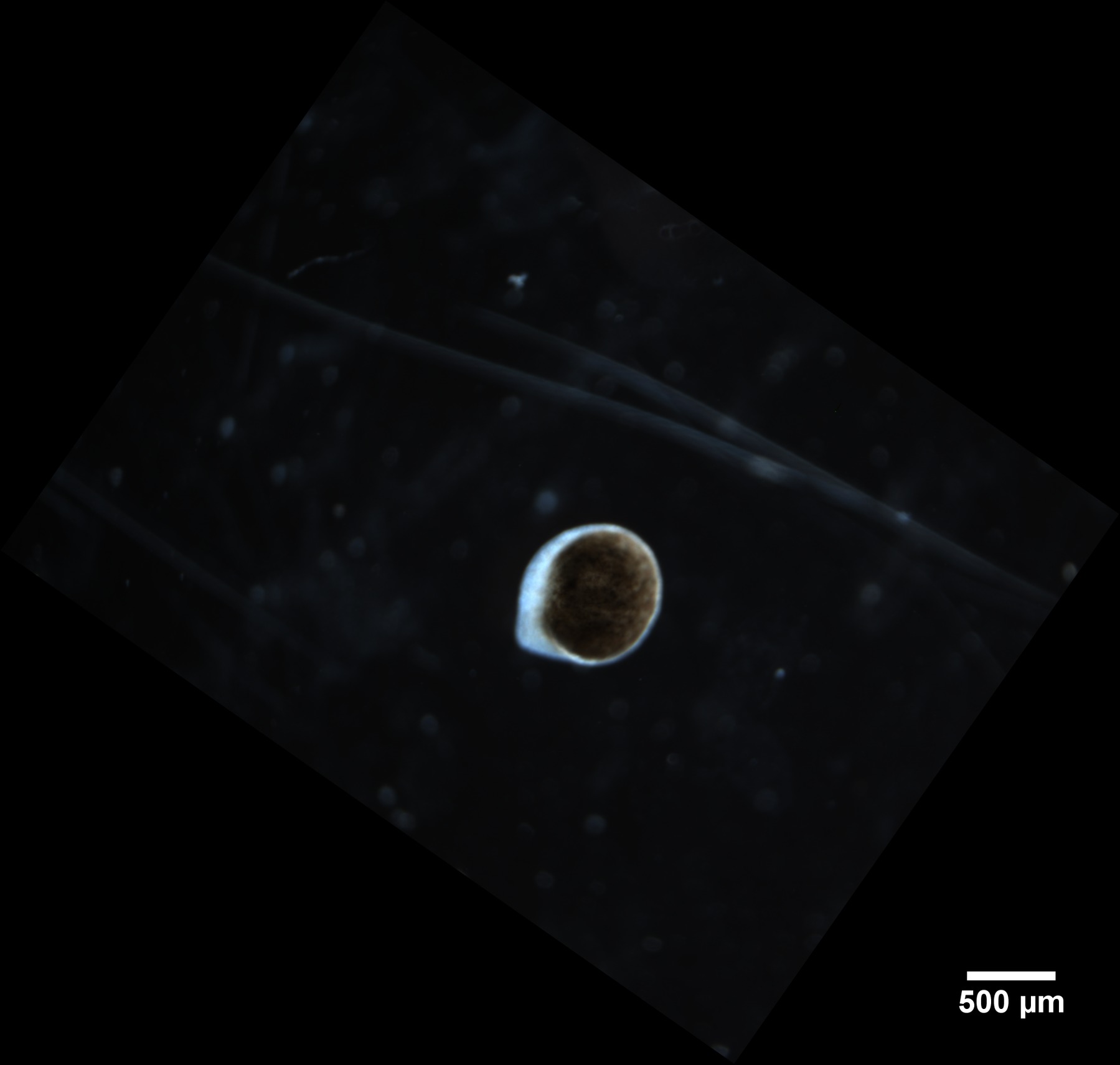

Supplement: S1 Dataset — This dataset contains brightfield image and corresponding synapsin stains for the VNC-free and VNC-containing small fragment cutting scenarios shown in Fig 6. Each image is labeled in the format “x_dpc_Sample_y_tn.jpg”, where “x” represents the number of days post cutting and “y” the replicate number. (ZIP) [file pcbi.1006904.s016.zip › smallfragments/VNC-free/Brigthfield_images/5 dpc_Sample 6_tn.jpg]

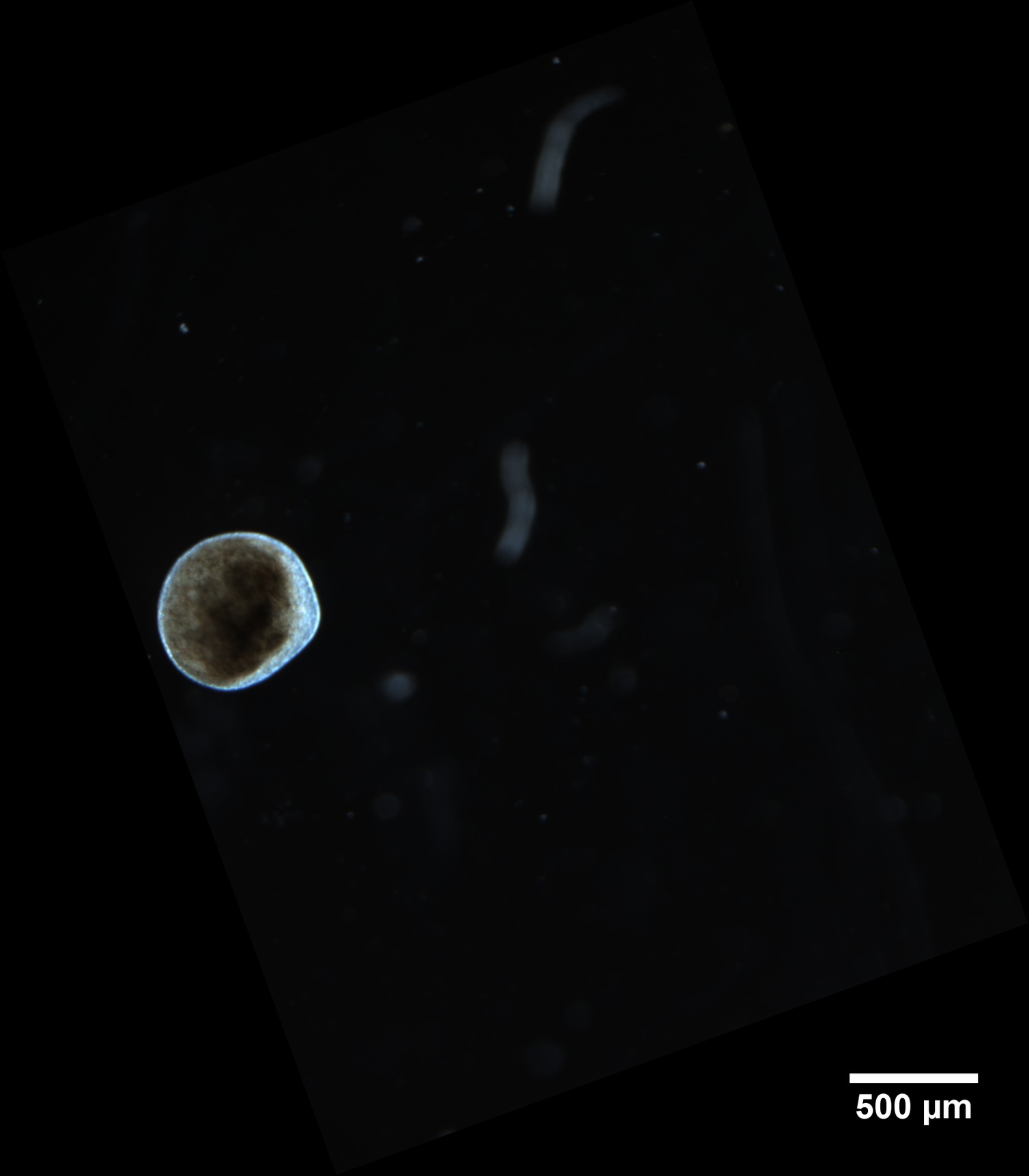

Supplement: S1 Dataset — This dataset contains brightfield image and corresponding synapsin stains for the VNC-free and VNC-containing small fragment cutting scenarios shown in Fig 6. Each image is labeled in the format “x_dpc_Sample_y_tn.jpg”, where “x” represents the number of days post cutting and “y” the replicate number. (ZIP) [file pcbi.1006904.s016.zip › smallfragments/VNC-free/Brigthfield_images/5 dpc_Sample 7_tn.jpg]

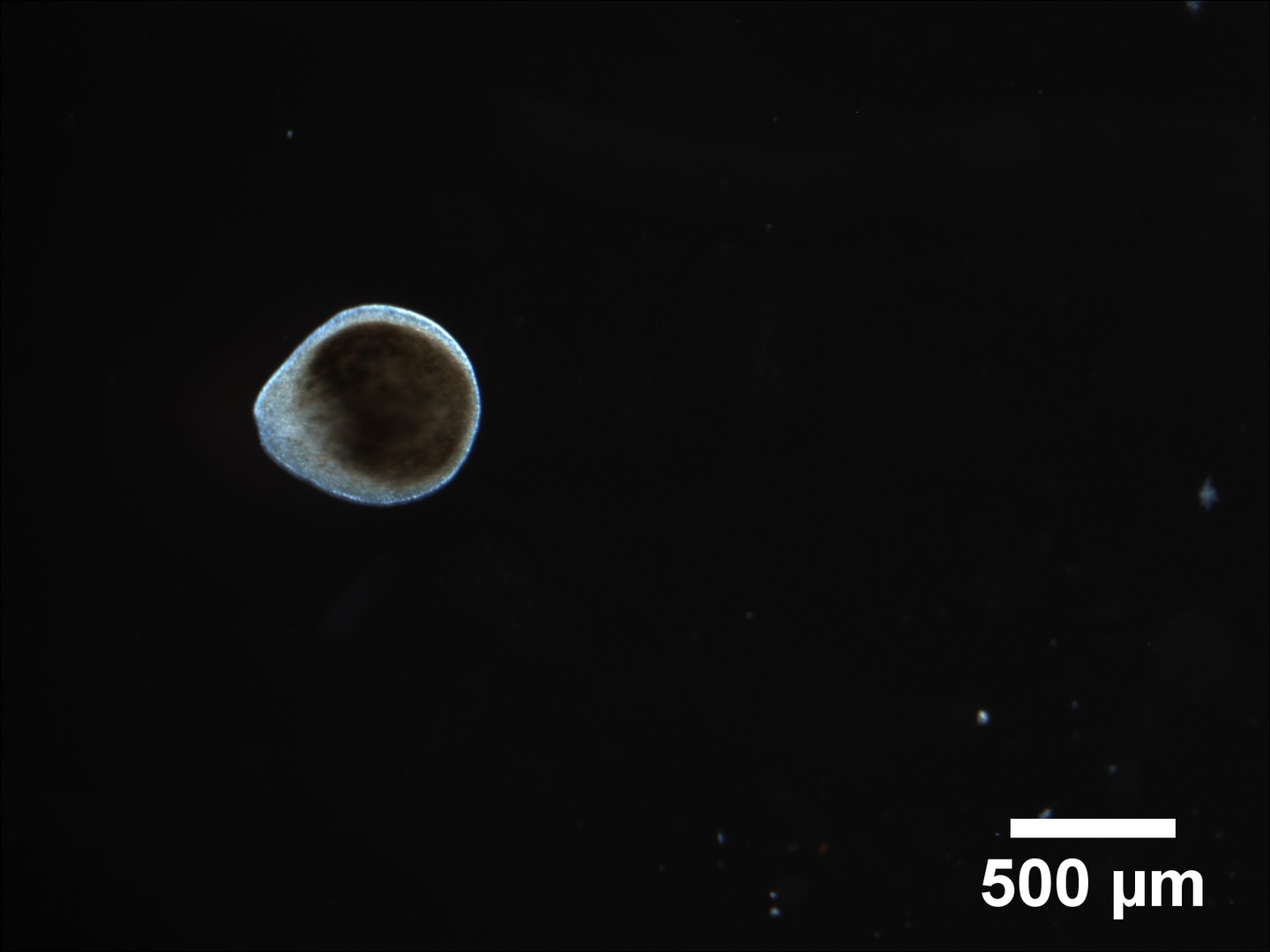

Supplement: S1 Dataset — This dataset contains brightfield image and corresponding synapsin stains for the VNC-free and VNC-containing small fragment cutting scenarios shown in Fig 6. Each image is labeled in the format “x_dpc_Sample_y_tn.jpg”, where “x” represents the number of days post cutting and “y” the replicate number. (ZIP) [file pcbi.1006904.s016.zip › smallfragments/VNC-free/Brigthfield_images/6 dpc_Sample 1_tn.jpg]

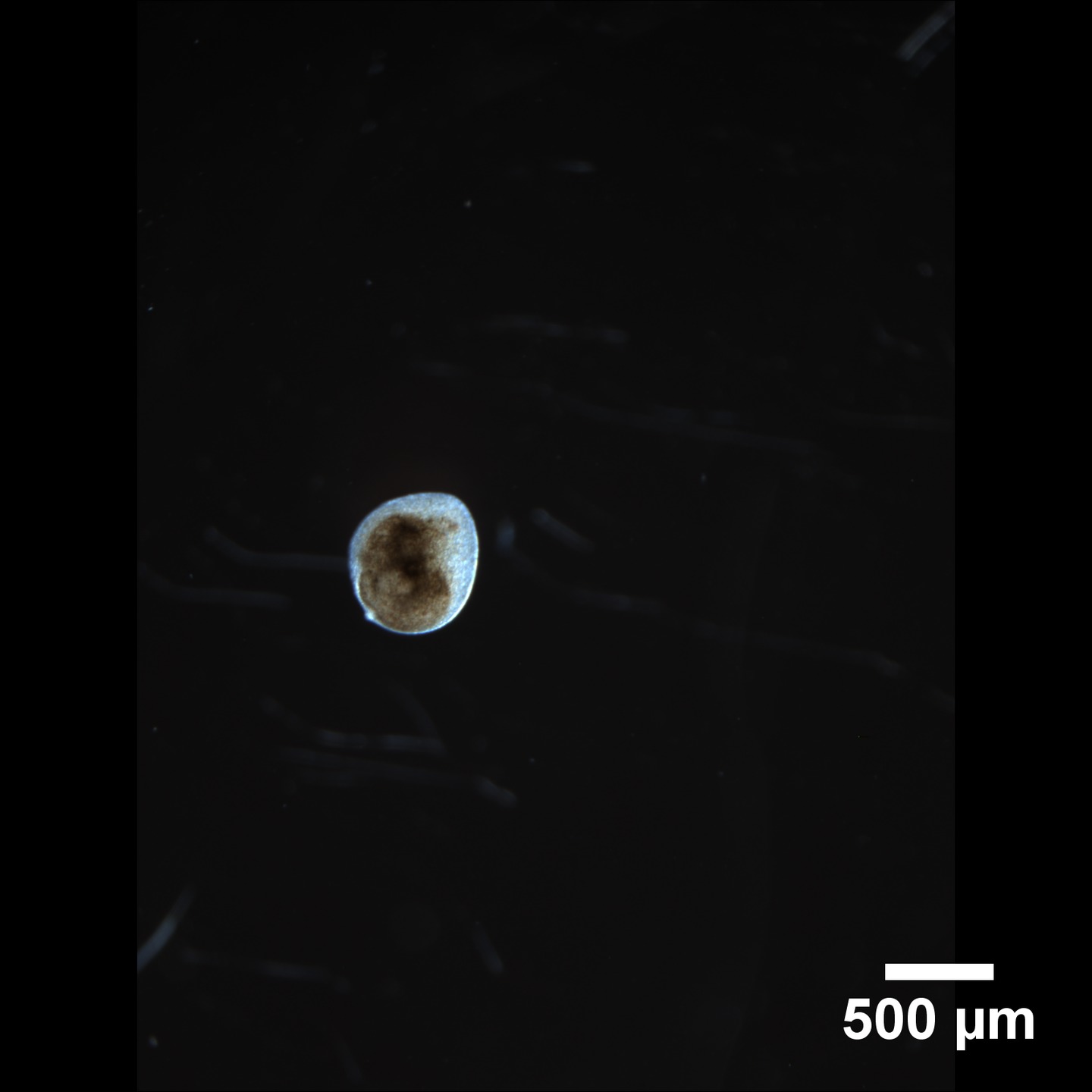

Supplement: S1 Dataset — This dataset contains brightfield image and corresponding synapsin stains for the VNC-free and VNC-containing small fragment cutting scenarios shown in Fig 6. Each image is labeled in the format “x_dpc_Sample_y_tn.jpg”, where “x” represents the number of days post cutting and “y” the replicate number. (ZIP) [file pcbi.1006904.s016.zip › smallfragments/VNC-free/Brigthfield_images/6 dpc_Sample 3_tn.jpg]

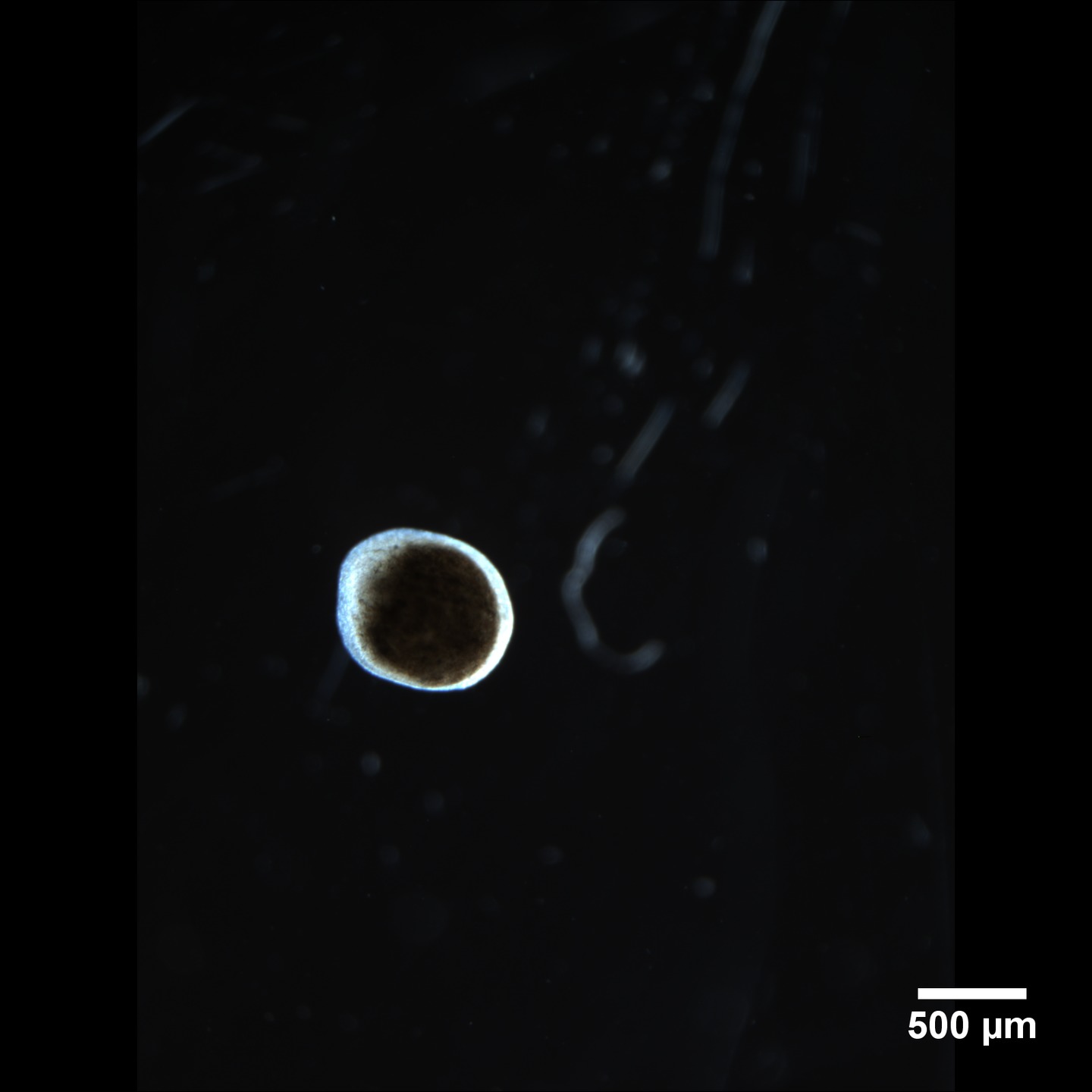

Supplement: S1 Dataset — This dataset contains brightfield image and corresponding synapsin stains for the VNC-free and VNC-containing small fragment cutting scenarios shown in Fig 6. Each image is labeled in the format “x_dpc_Sample_y_tn.jpg”, where “x” represents the number of days post cutting and “y” the replicate number. (ZIP) [file pcbi.1006904.s016.zip › smallfragments/VNC-free/Brigthfield_images/6 dpc_Sample 4_tn.jpg]

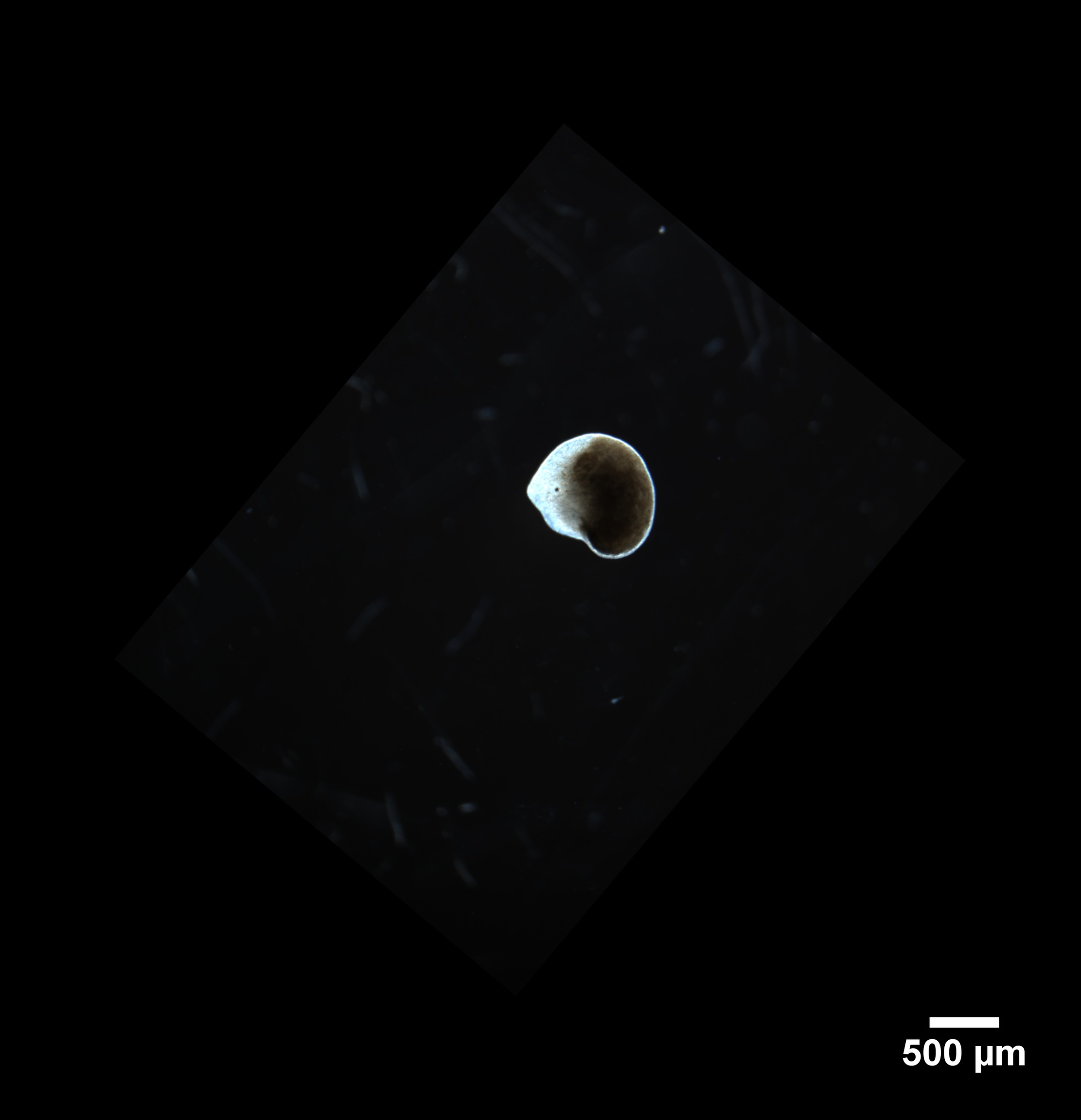

Supplement: S1 Dataset — This dataset contains brightfield image and corresponding synapsin stains for the VNC-free and VNC-containing small fragment cutting scenarios shown in Fig 6. Each image is labeled in the format “x_dpc_Sample_y_tn.jpg”, where “x” represents the number of days post cutting and “y” the replicate number. (ZIP) [file pcbi.1006904.s016.zip › smallfragments/VNC-free/Brigthfield_images/6 dpc_Sample 5_tn.jpg]

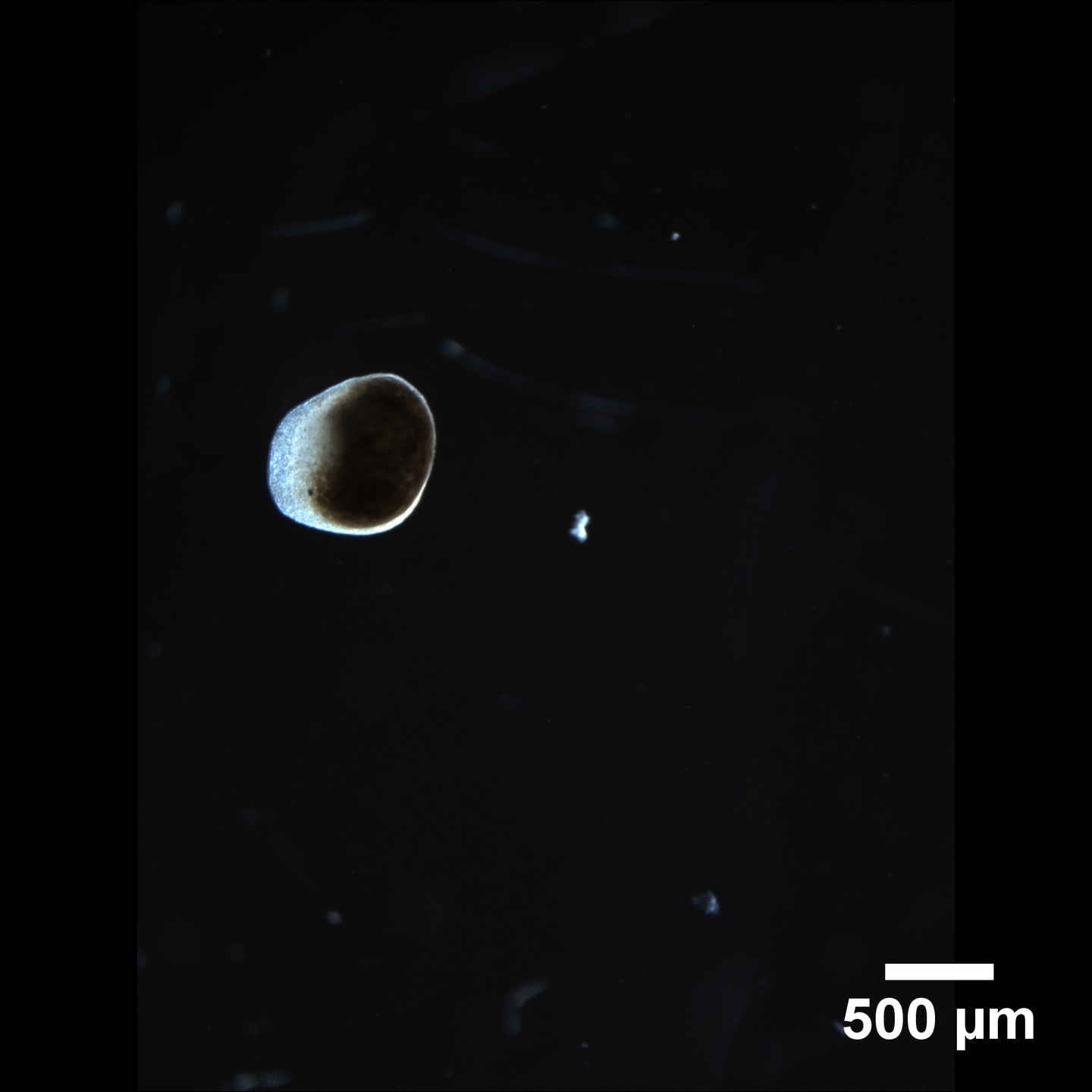

Supplement: S1 Dataset — This dataset contains brightfield image and corresponding synapsin stains for the VNC-free and VNC-containing small fragment cutting scenarios shown in Fig 6. Each image is labeled in the format “x_dpc_Sample_y_tn.jpg”, where “x” represents the number of days post cutting and “y” the replicate number. (ZIP) [file pcbi.1006904.s016.zip › smallfragments/VNC-free/Brigthfield_images/6 dpc_Sample 6_tn.jpg]

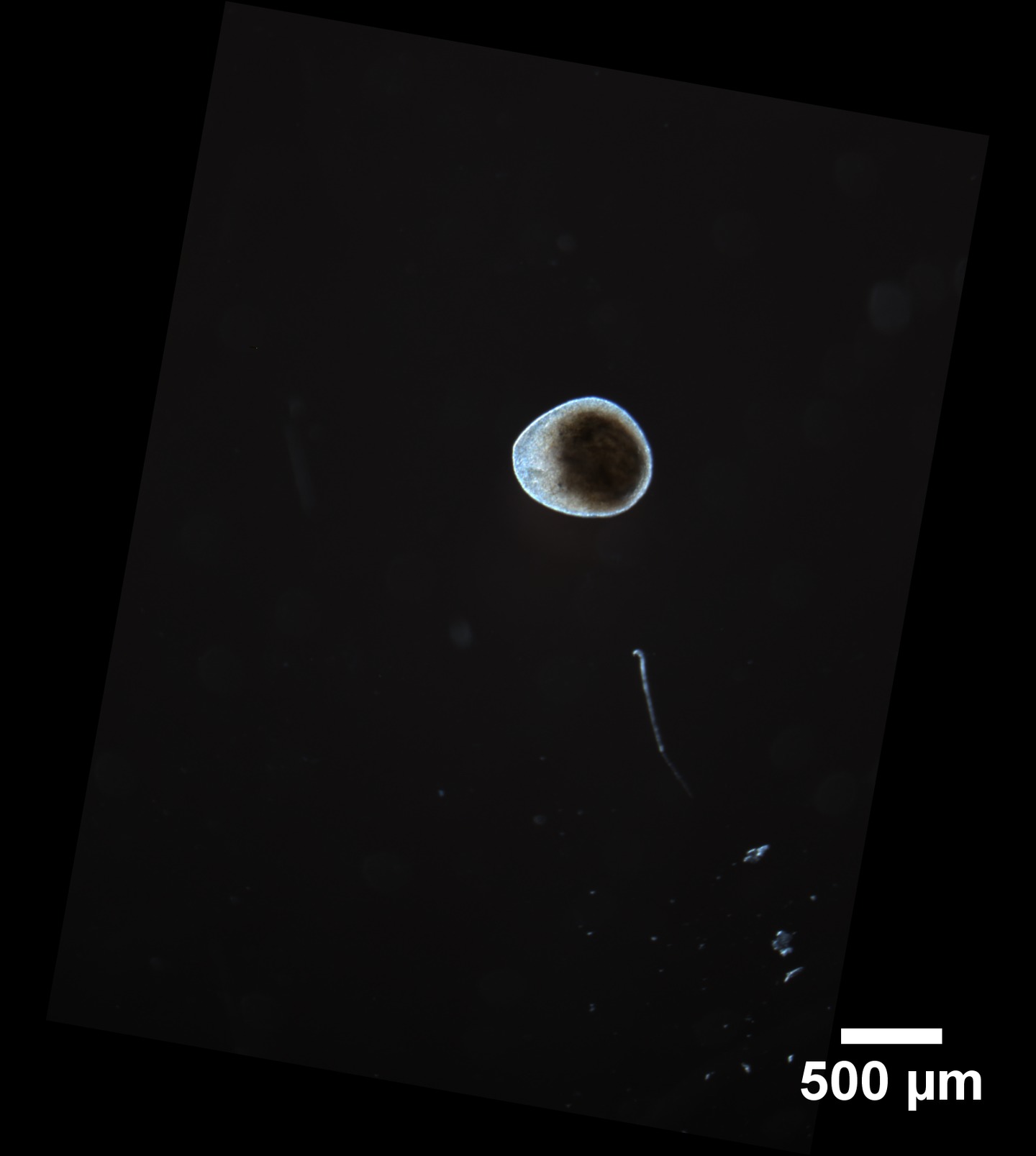

Supplement: S1 Dataset — This dataset contains brightfield image and corresponding synapsin stains for the VNC-free and VNC-containing small fragment cutting scenarios shown in Fig 6. Each image is labeled in the format “x_dpc_Sample_y_tn.jpg”, where “x” represents the number of days post cutting and “y” the replicate number. (ZIP) [file pcbi.1006904.s016.zip › smallfragments/VNC-free/Brigthfield_images/7 dpc_Sample 1_tn.jpg]

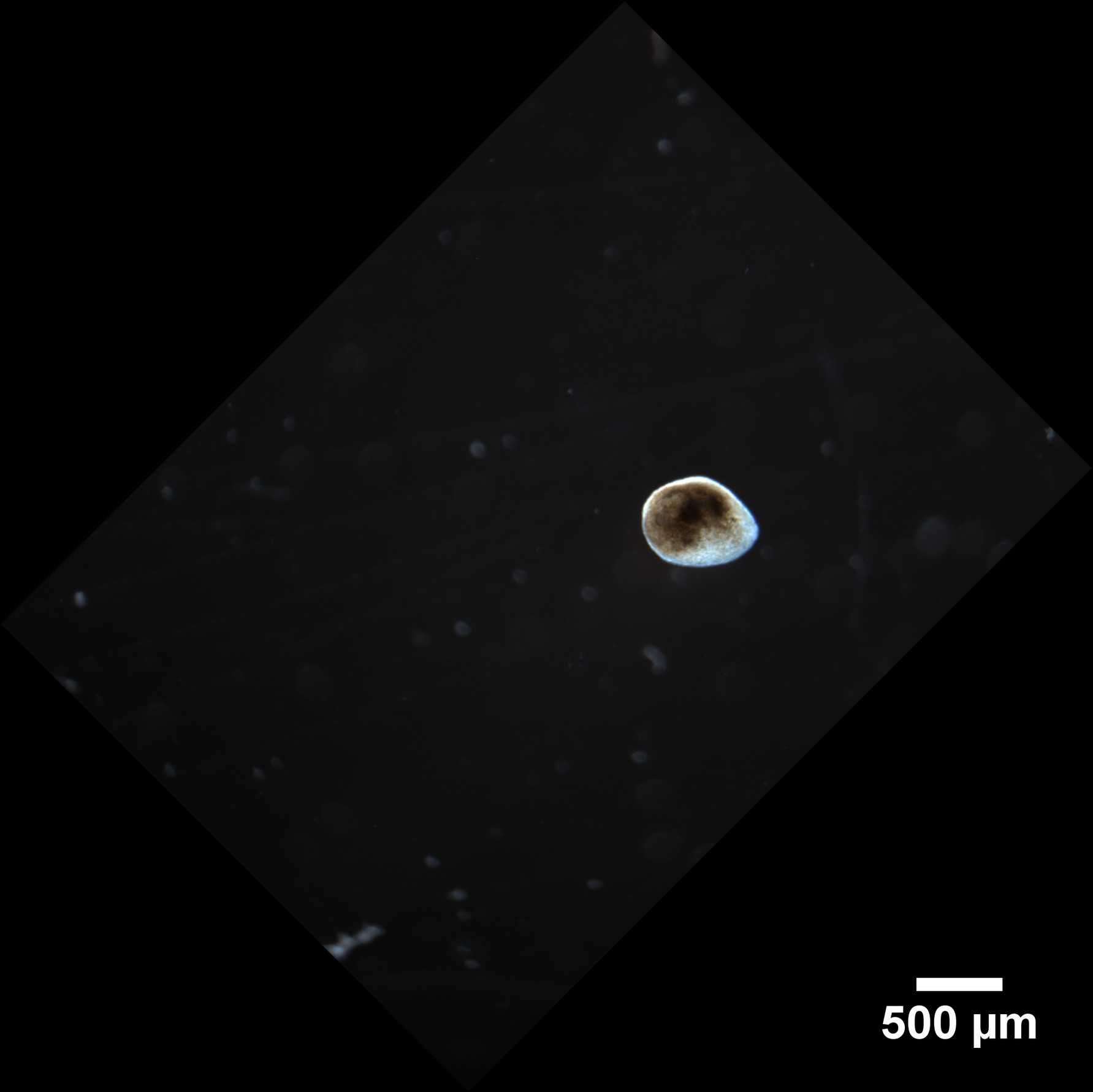

Supplement: S1 Dataset — This dataset contains brightfield image and corresponding synapsin stains for the VNC-free and VNC-containing small fragment cutting scenarios shown in Fig 6. Each image is labeled in the format “x_dpc_Sample_y_tn.jpg”, where “x” represents the number of days post cutting and “y” the replicate number. (ZIP) [file pcbi.1006904.s016.zip › smallfragments/VNC-free/Brigthfield_images/7 dpc_Sample 3_tn.jpg]

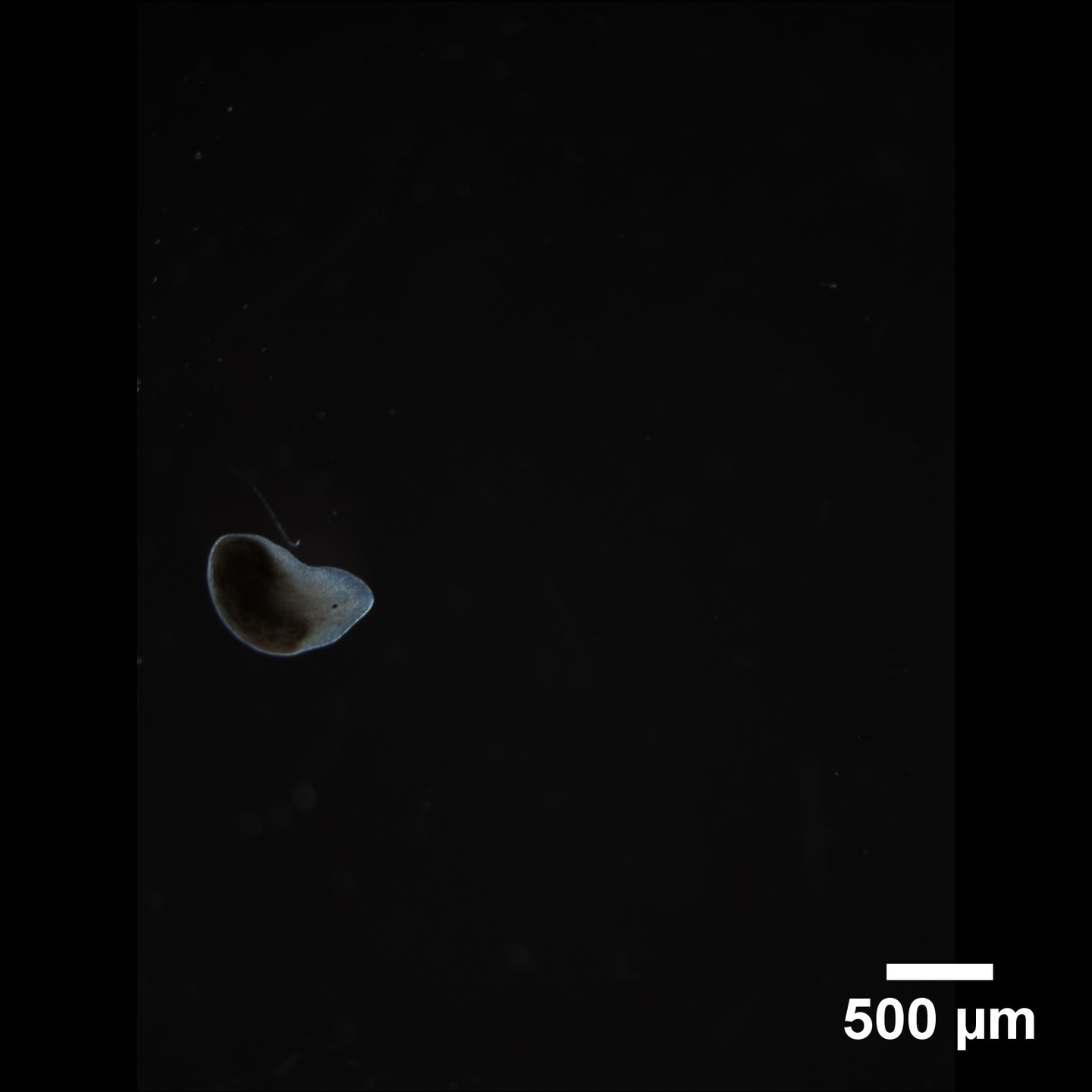

Supplement: S1 Dataset — This dataset contains brightfield image and corresponding synapsin stains for the VNC-free and VNC-containing small fragment cutting scenarios shown in Fig 6. Each image is labeled in the format “x_dpc_Sample_y_tn.jpg”, where “x” represents the number of days post cutting and “y” the replicate number. (ZIP) [file pcbi.1006904.s016.zip › smallfragments/VNC-free/Brigthfield_images/7 dpc_Sample 5_tn.jpg]

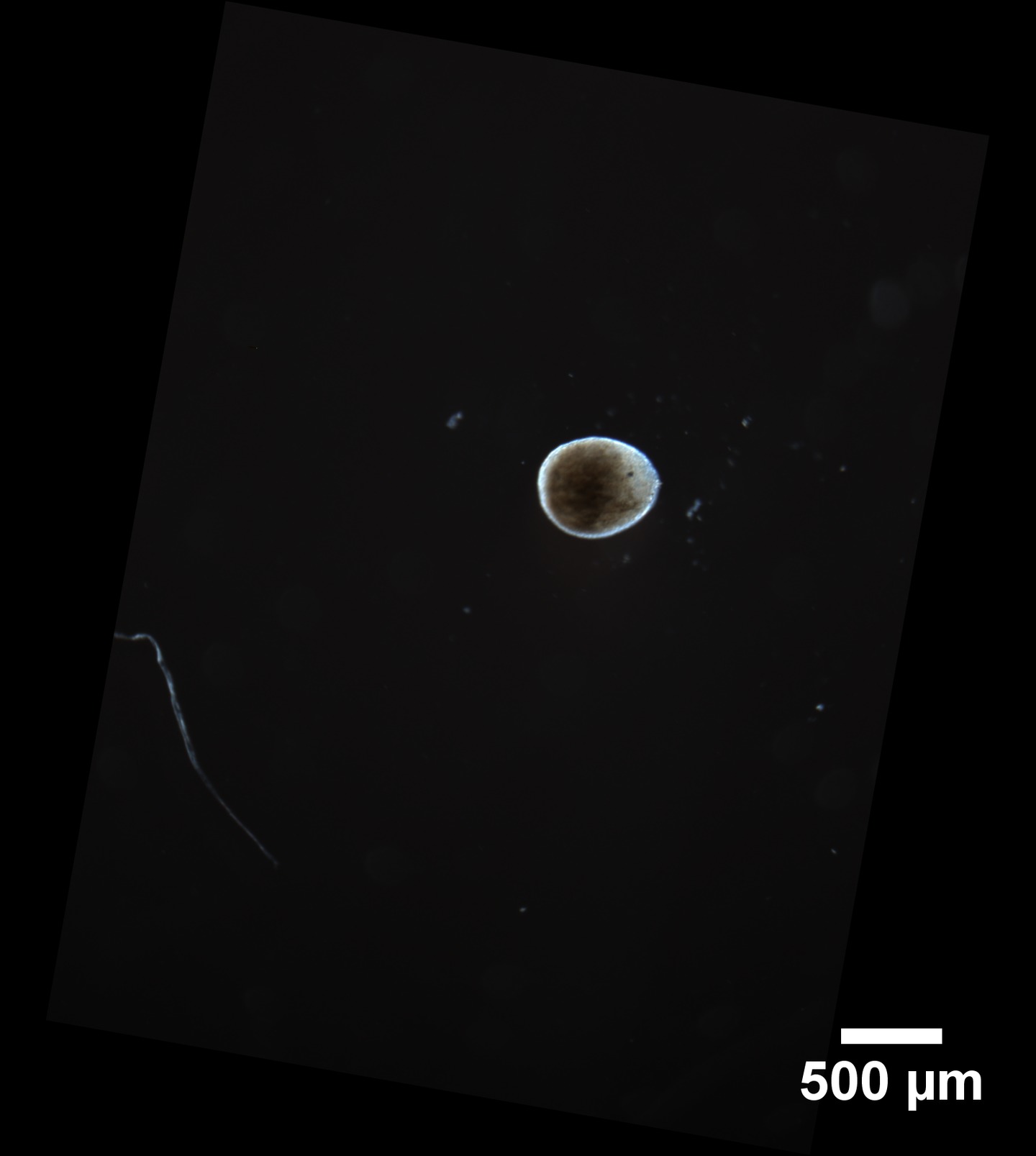

Supplement: S1 Dataset — This dataset contains brightfield image and corresponding synapsin stains for the VNC-free and VNC-containing small fragment cutting scenarios shown in Fig 6. Each image is labeled in the format “x_dpc_Sample_y_tn.jpg”, where “x” represents the number of days post cutting and “y” the replicate number. (ZIP) [file pcbi.1006904.s016.zip › smallfragments/VNC-free/Brigthfield_images/7 dpc_Sample 6_tn.jpg]

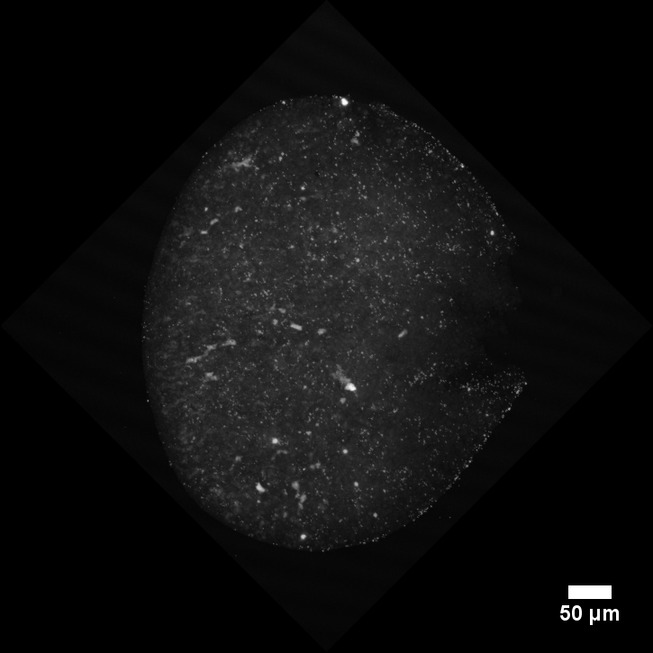

Supplement: S1 Dataset — This dataset contains brightfield image and corresponding synapsin stains for the VNC-free and VNC-containing small fragment cutting scenarios shown in Fig 6. Each image is labeled in the format “x_dpc_Sample_y_tn.jpg”, where “x” represents the number of days post cutting and “y” the replicate number. (ZIP) [file pcbi.1006904.s016.zip › smallfragments/VNC-free/synapsin_stains/1 dpc_Sample 10_tn.jpg]

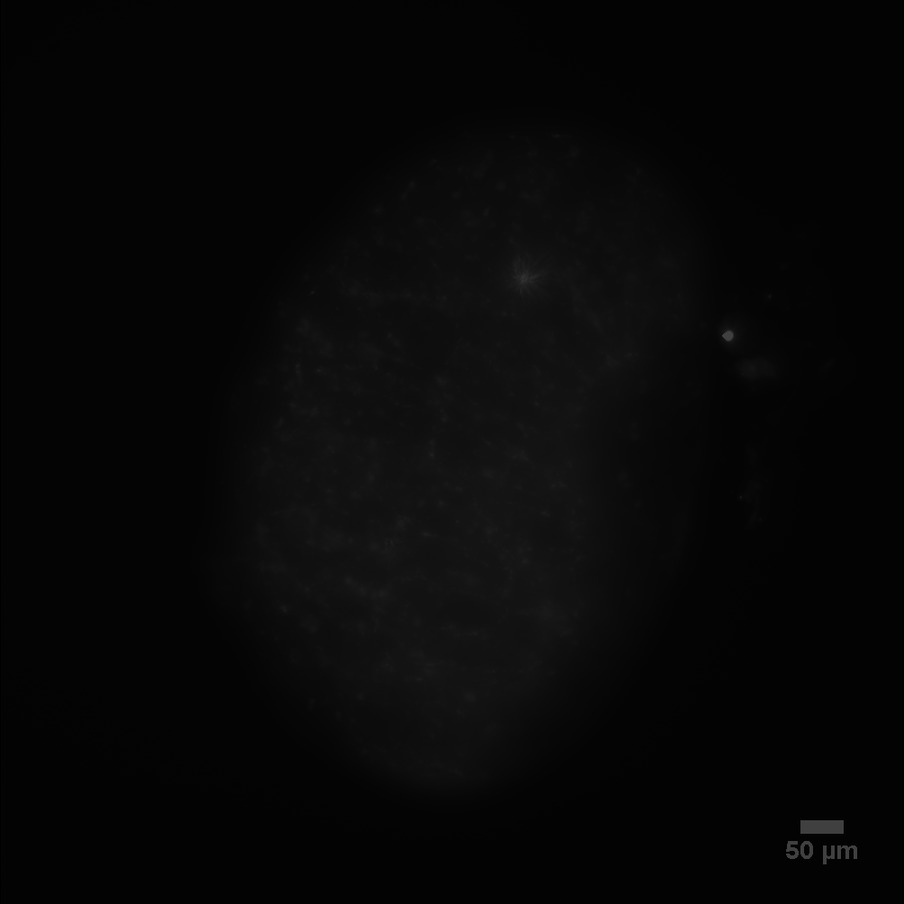

Supplement: S1 Dataset — This dataset contains brightfield image and corresponding synapsin stains for the VNC-free and VNC-containing small fragment cutting scenarios shown in Fig 6. Each image is labeled in the format “x_dpc_Sample_y_tn.jpg”, where “x” represents the number of days post cutting and “y” the replicate number. (ZIP) [file pcbi.1006904.s016.zip › smallfragments/VNC-free/synapsin_stains/1 dpc_Sample 1_tn.jpg]

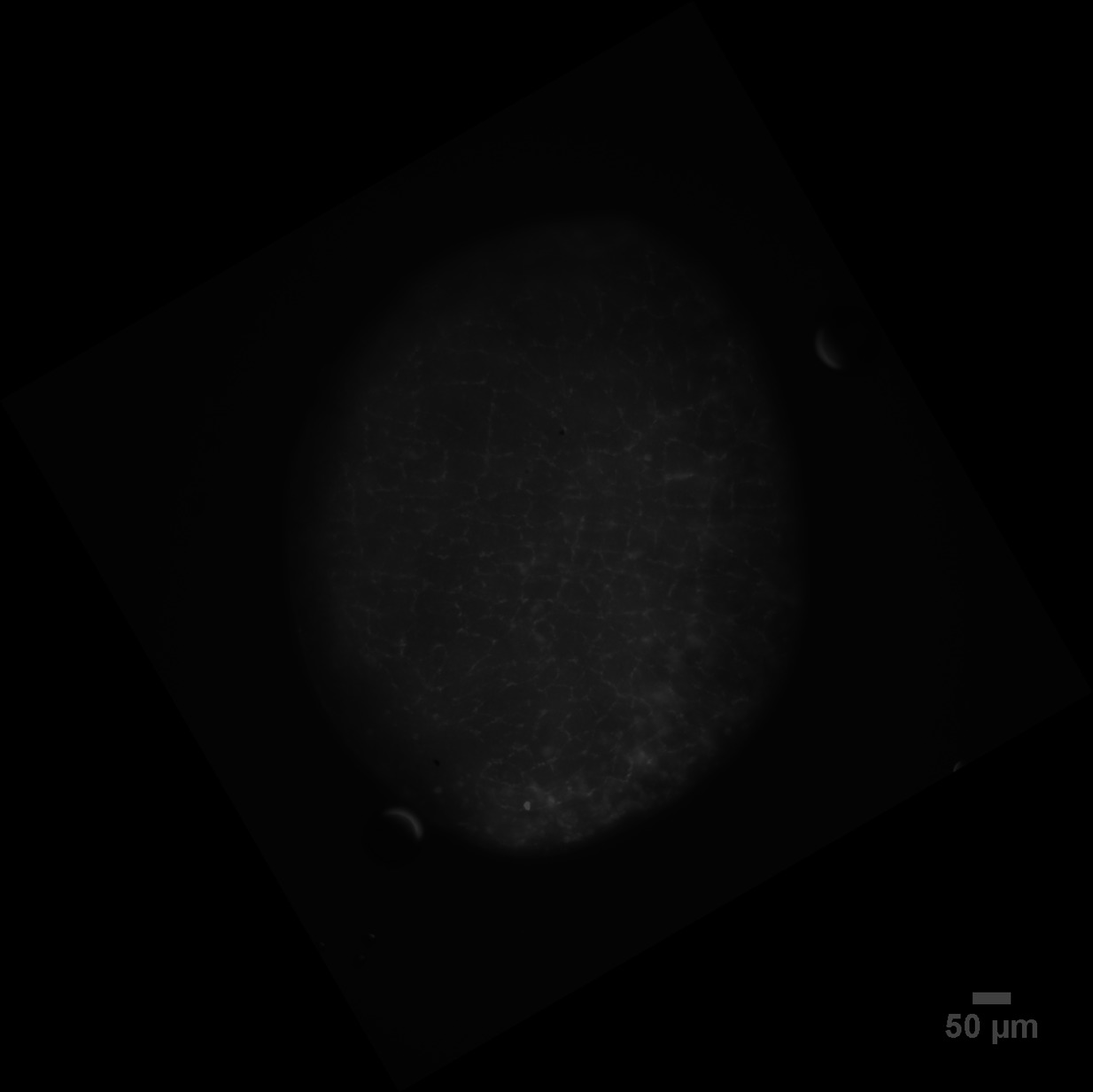

Supplement: S1 Dataset — This dataset contains brightfield image and corresponding synapsin stains for the VNC-free and VNC-containing small fragment cutting scenarios shown in Fig 6. Each image is labeled in the format “x_dpc_Sample_y_tn.jpg”, where “x” represents the number of days post cutting and “y” the replicate number. (ZIP) [file pcbi.1006904.s016.zip › smallfragments/VNC-free/synapsin_stains/1 dpc_Sample 2_tn.jpg]

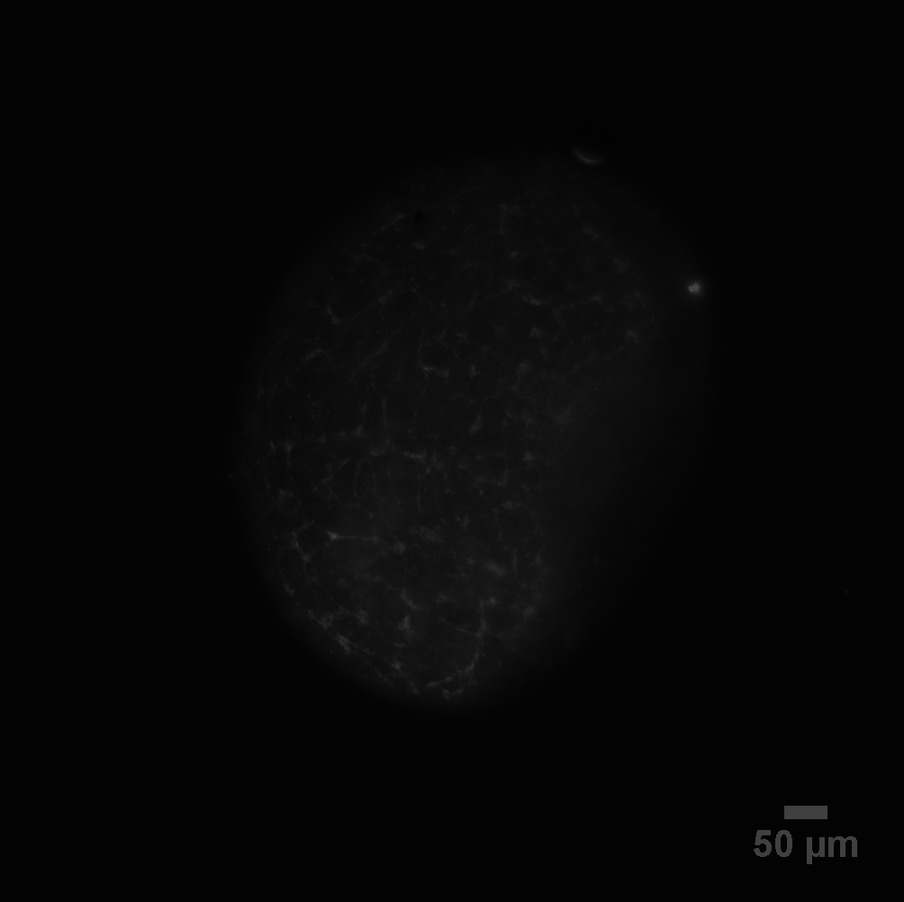

Supplement: S1 Dataset — This dataset contains brightfield image and corresponding synapsin stains for the VNC-free and VNC-containing small fragment cutting scenarios shown in Fig 6. Each image is labeled in the format “x_dpc_Sample_y_tn.jpg”, where “x” represents the number of days post cutting and “y” the replicate number. (ZIP) [file pcbi.1006904.s016.zip › smallfragments/VNC-free/synapsin_stains/1 dpc_Sample 3_tn.jpg]

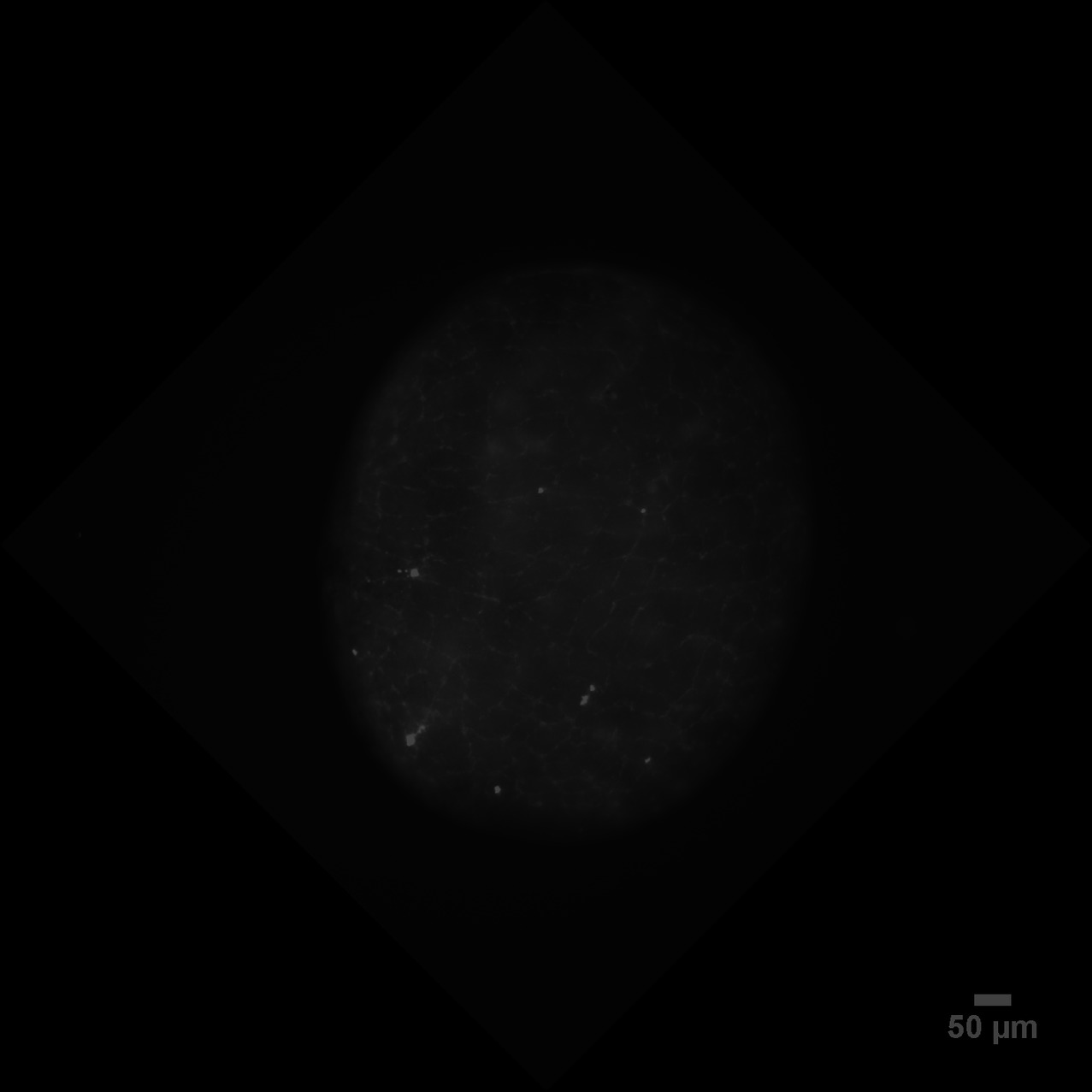

Supplement: S1 Dataset — This dataset contains brightfield image and corresponding synapsin stains for the VNC-free and VNC-containing small fragment cutting scenarios shown in Fig 6. Each image is labeled in the format “x_dpc_Sample_y_tn.jpg”, where “x” represents the number of days post cutting and “y” the replicate number. (ZIP) [file pcbi.1006904.s016.zip › smallfragments/VNC-free/synapsin_stains/1 dpc_Sample 4_tn.jpg]

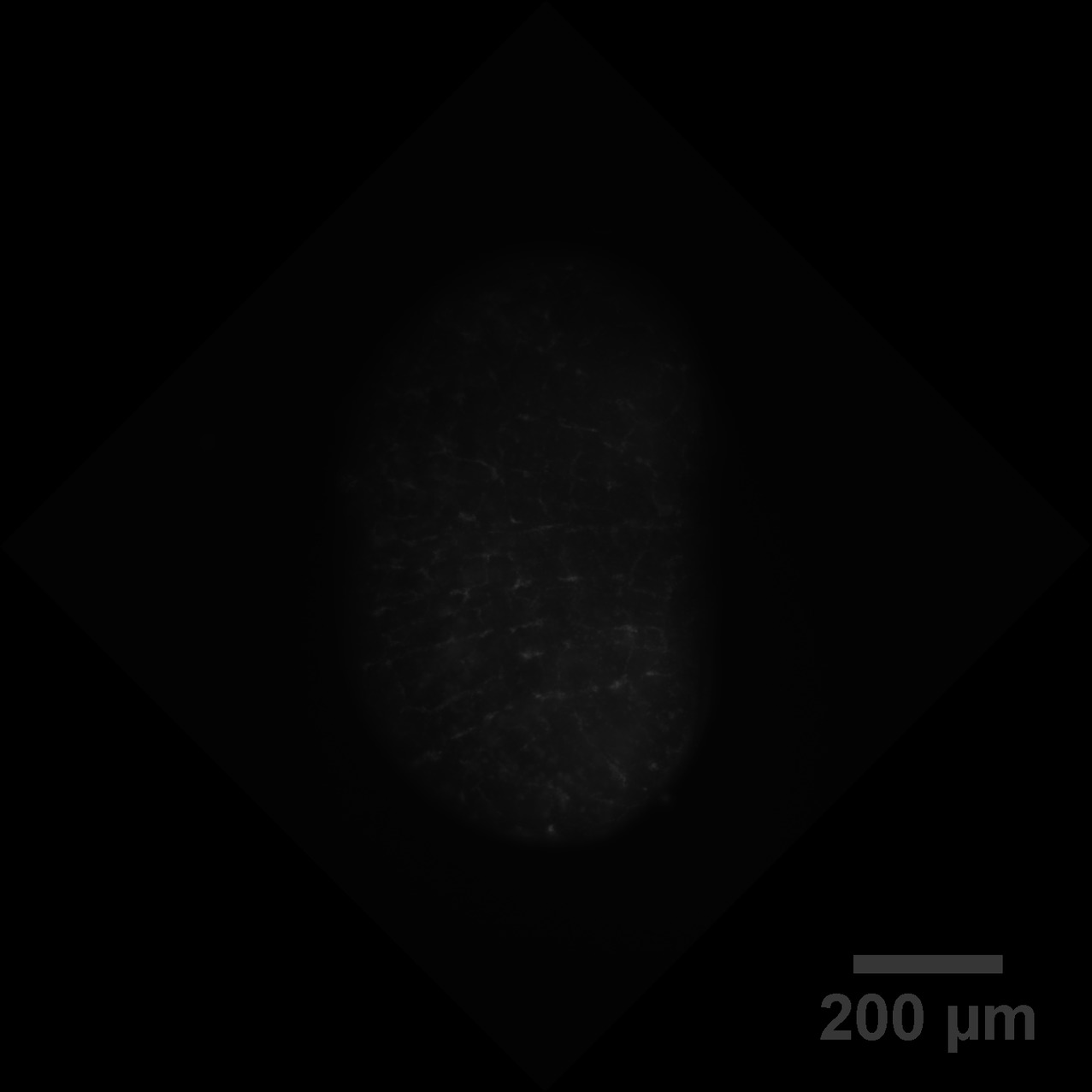

Supplement: S1 Dataset — This dataset contains brightfield image and corresponding synapsin stains for the VNC-free and VNC-containing small fragment cutting scenarios shown in Fig 6. Each image is labeled in the format “x_dpc_Sample_y_tn.jpg”, where “x” represents the number of days post cutting and “y” the replicate number. (ZIP) [file pcbi.1006904.s016.zip › smallfragments/VNC-free/synapsin_stains/1 dpc_Sample 5_tn.jpg]

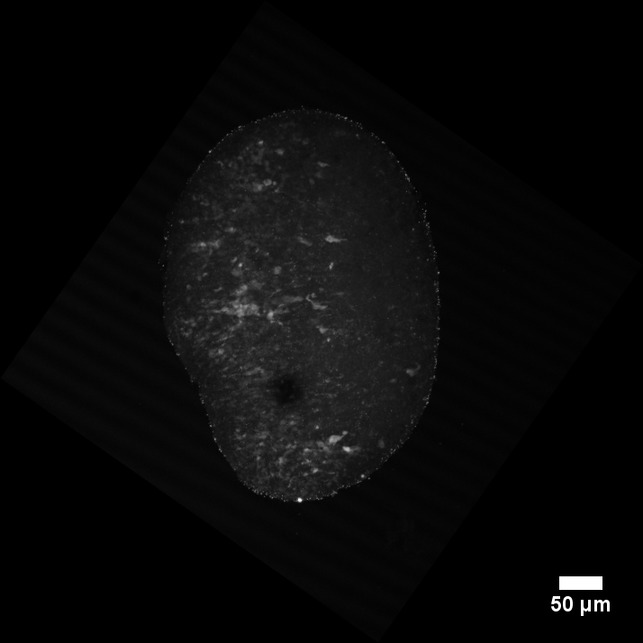

Supplement: S1 Dataset — This dataset contains brightfield image and corresponding synapsin stains for the VNC-free and VNC-containing small fragment cutting scenarios shown in Fig 6. Each image is labeled in the format “x_dpc_Sample_y_tn.jpg”, where “x” represents the number of days post cutting and “y” the replicate number. (ZIP) [file pcbi.1006904.s016.zip › smallfragments/VNC-free/synapsin_stains/1 dpc_Sample 6_tn.jpg]

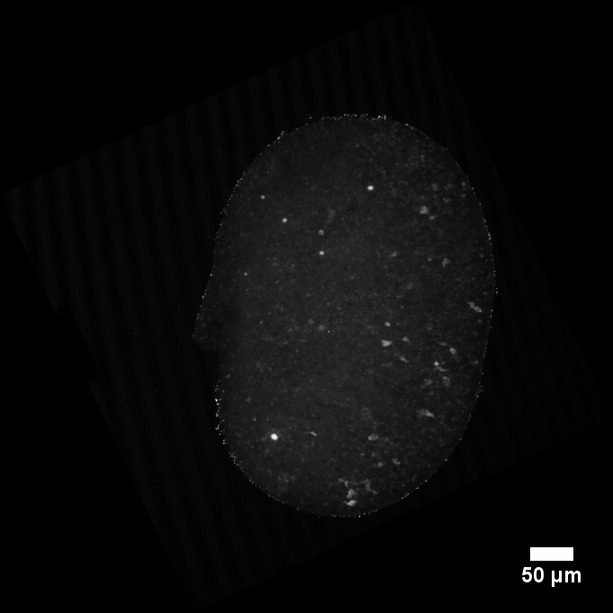

Supplement: S1 Dataset — This dataset contains brightfield image and corresponding synapsin stains for the VNC-free and VNC-containing small fragment cutting scenarios shown in Fig 6. Each image is labeled in the format “x_dpc_Sample_y_tn.jpg”, where “x” represents the number of days post cutting and “y” the replicate number. (ZIP) [file pcbi.1006904.s016.zip › smallfragments/VNC-free/synapsin_stains/1 dpc_Sample 7_tn.jpg]

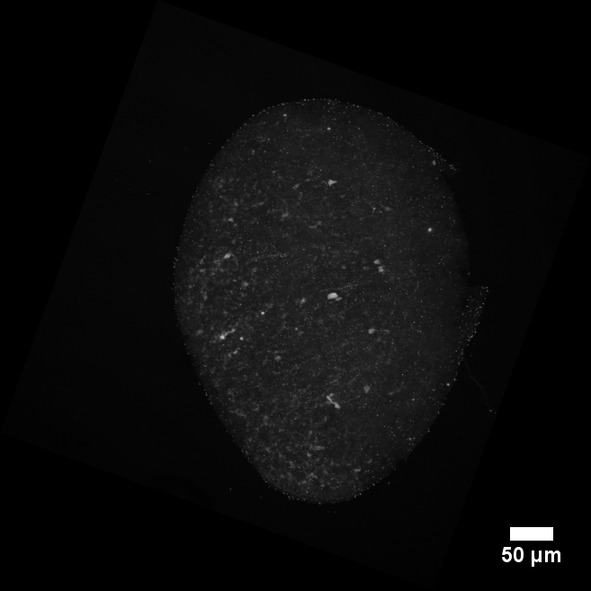

Supplement: S1 Dataset — This dataset contains brightfield image and corresponding synapsin stains for the VNC-free and VNC-containing small fragment cutting scenarios shown in Fig 6. Each image is labeled in the format “x_dpc_Sample_y_tn.jpg”, where “x” represents the number of days post cutting and “y” the replicate number. (ZIP) [file pcbi.1006904.s016.zip › smallfragments/VNC-free/synapsin_stains/1 dpc_Sample 8_tn.jpg]

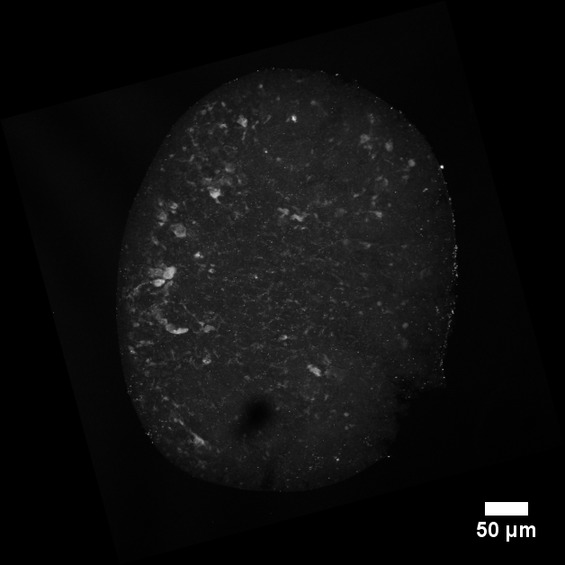

Supplement: S1 Dataset — This dataset contains brightfield image and corresponding synapsin stains for the VNC-free and VNC-containing small fragment cutting scenarios shown in Fig 6. Each image is labeled in the format “x_dpc_Sample_y_tn.jpg”, where “x” represents the number of days post cutting and “y” the replicate number. (ZIP) [file pcbi.1006904.s016.zip › smallfragments/VNC-free/synapsin_stains/1 dpc_Sample 9_tn.jpg]

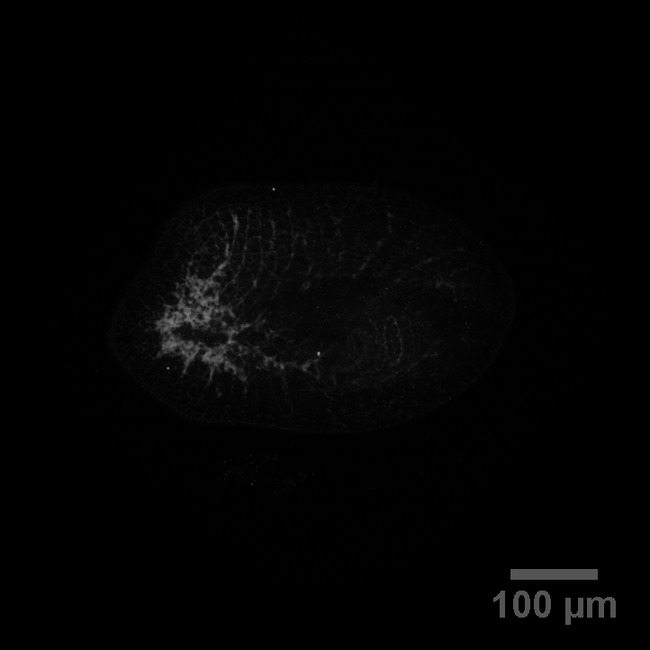

Supplement: S1 Dataset — This dataset contains brightfield image and corresponding synapsin stains for the VNC-free and VNC-containing small fragment cutting scenarios shown in Fig 6. Each image is labeled in the format “x_dpc_Sample_y_tn.jpg”, where “x” represents the number of days post cutting and “y” the replicate number. (ZIP) [file pcbi.1006904.s016.zip › smallfragments/VNC-free/synapsin_stains/10 dpc_Sample 1_tn.jpg]

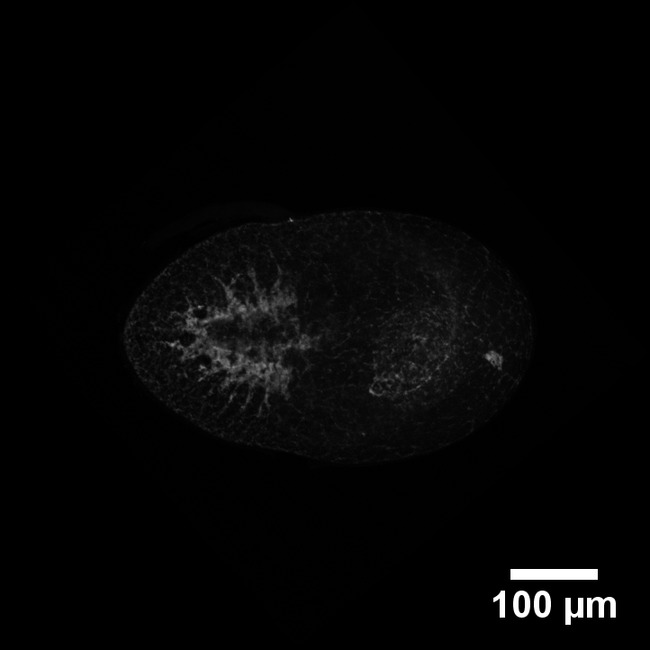

Supplement: S1 Dataset — This dataset contains brightfield image and corresponding synapsin stains for the VNC-free and VNC-containing small fragment cutting scenarios shown in Fig 6. Each image is labeled in the format “x_dpc_Sample_y_tn.jpg”, where “x” represents the number of days post cutting and “y” the replicate number. (ZIP) [file pcbi.1006904.s016.zip › smallfragments/VNC-free/synapsin_stains/14 dpc_Sample 1_tn.jpg]

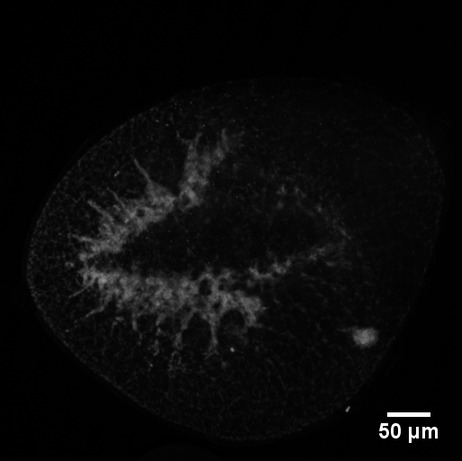

Supplement: S1 Dataset — This dataset contains brightfield image and corresponding synapsin stains for the VNC-free and VNC-containing small fragment cutting scenarios shown in Fig 6. Each image is labeled in the format “x_dpc_Sample_y_tn.jpg”, where “x” represents the number of days post cutting and “y” the replicate number. (ZIP) [file pcbi.1006904.s016.zip › smallfragments/VNC-free/synapsin_stains/14 dpc_Sample 2_tn.jpg]

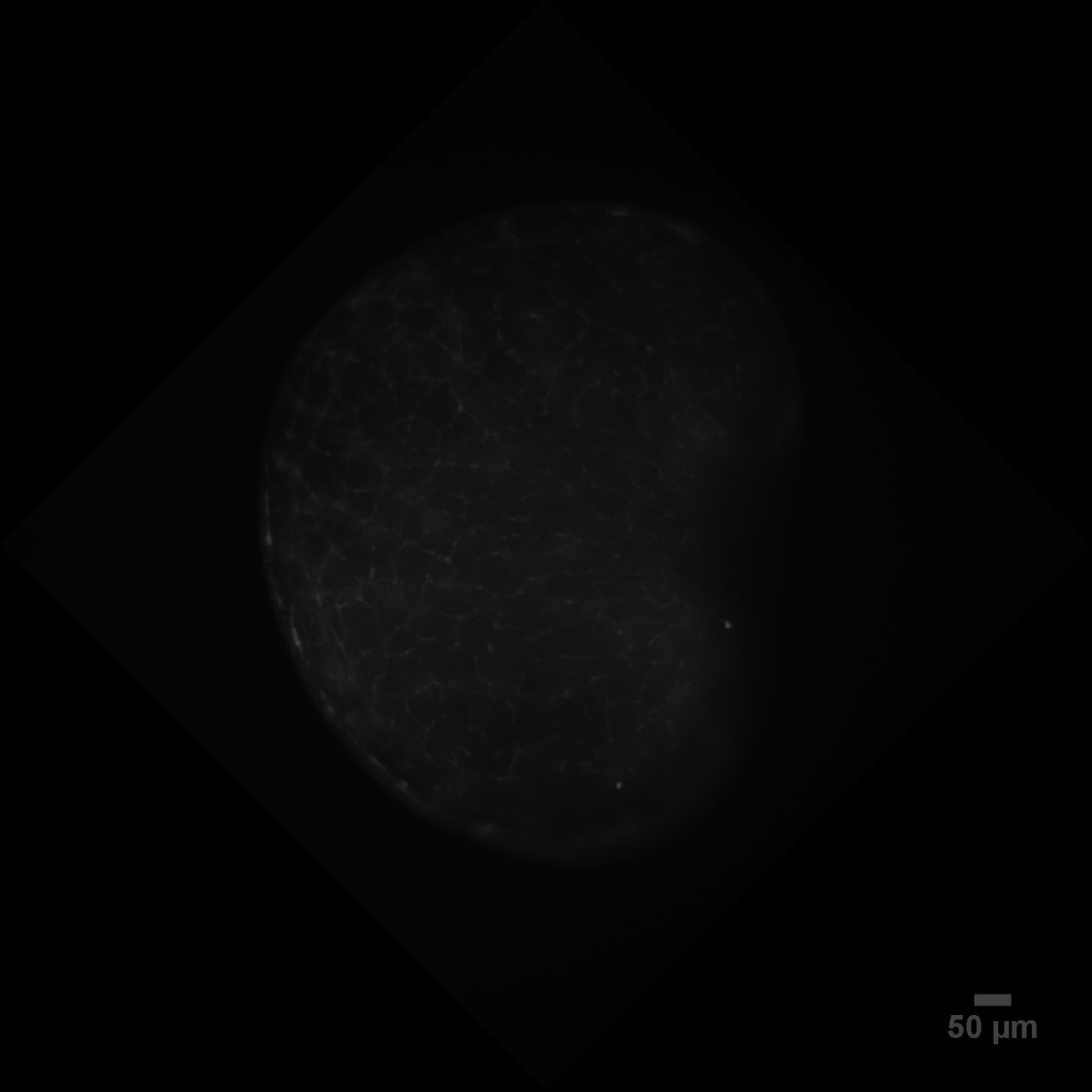

Supplement: S1 Dataset — This dataset contains brightfield image and corresponding synapsin stains for the VNC-free and VNC-containing small fragment cutting scenarios shown in Fig 6. Each image is labeled in the format “x_dpc_Sample_y_tn.jpg”, where “x” represents the number of days post cutting and “y” the replicate number. (ZIP) [file pcbi.1006904.s016.zip › smallfragments/VNC-free/synapsin_stains/2 dpc_Sample 1_tn.jpg]

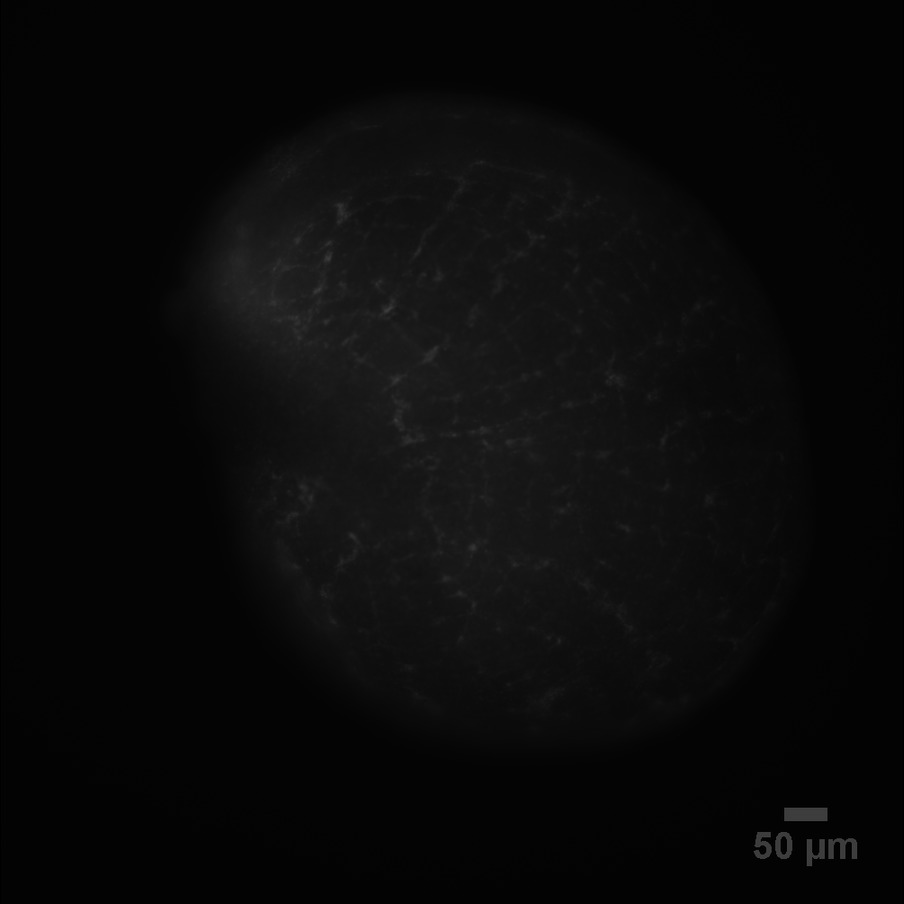

Supplement: S1 Dataset — This dataset contains brightfield image and corresponding synapsin stains for the VNC-free and VNC-containing small fragment cutting scenarios shown in Fig 6. Each image is labeled in the format “x_dpc_Sample_y_tn.jpg”, where “x” represents the number of days post cutting and “y” the replicate number. (ZIP) [file pcbi.1006904.s016.zip › smallfragments/VNC-free/synapsin_stains/2 dpc_Sample 2_tn.jpg]

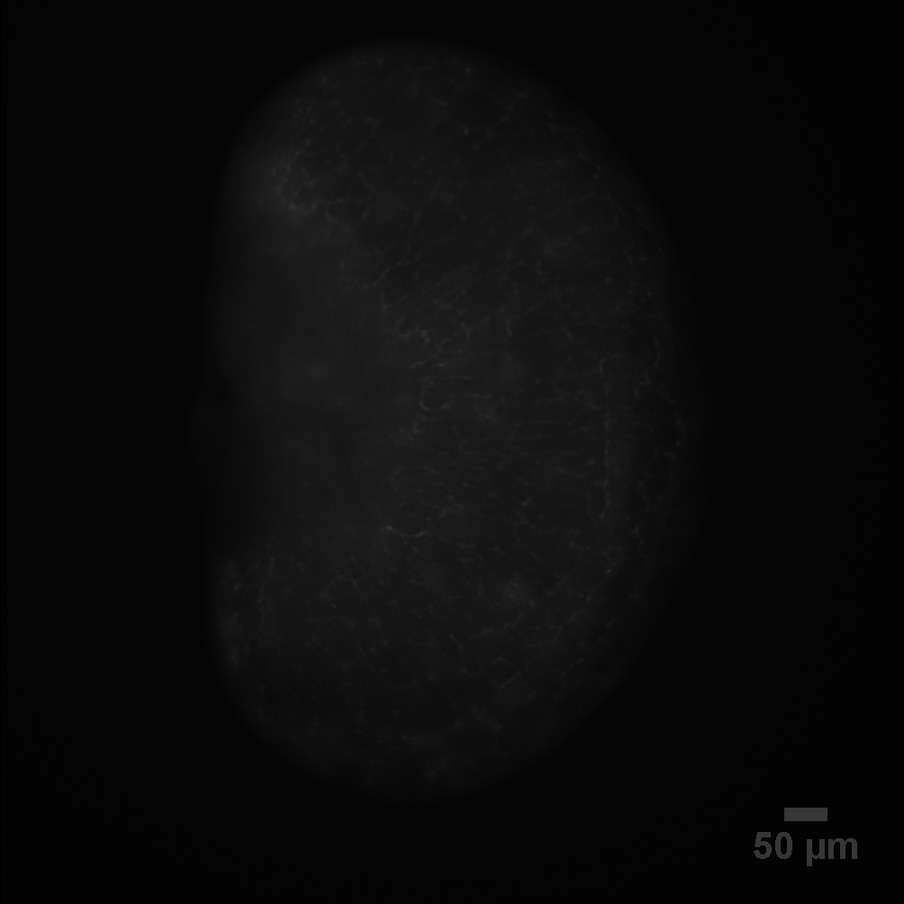

Supplement: S1 Dataset — This dataset contains brightfield image and corresponding synapsin stains for the VNC-free and VNC-containing small fragment cutting scenarios shown in Fig 6. Each image is labeled in the format “x_dpc_Sample_y_tn.jpg”, where “x” represents the number of days post cutting and “y” the replicate number. (ZIP) [file pcbi.1006904.s016.zip › smallfragments/VNC-free/synapsin_stains/2 dpc_Sample 3_tn.jpg]

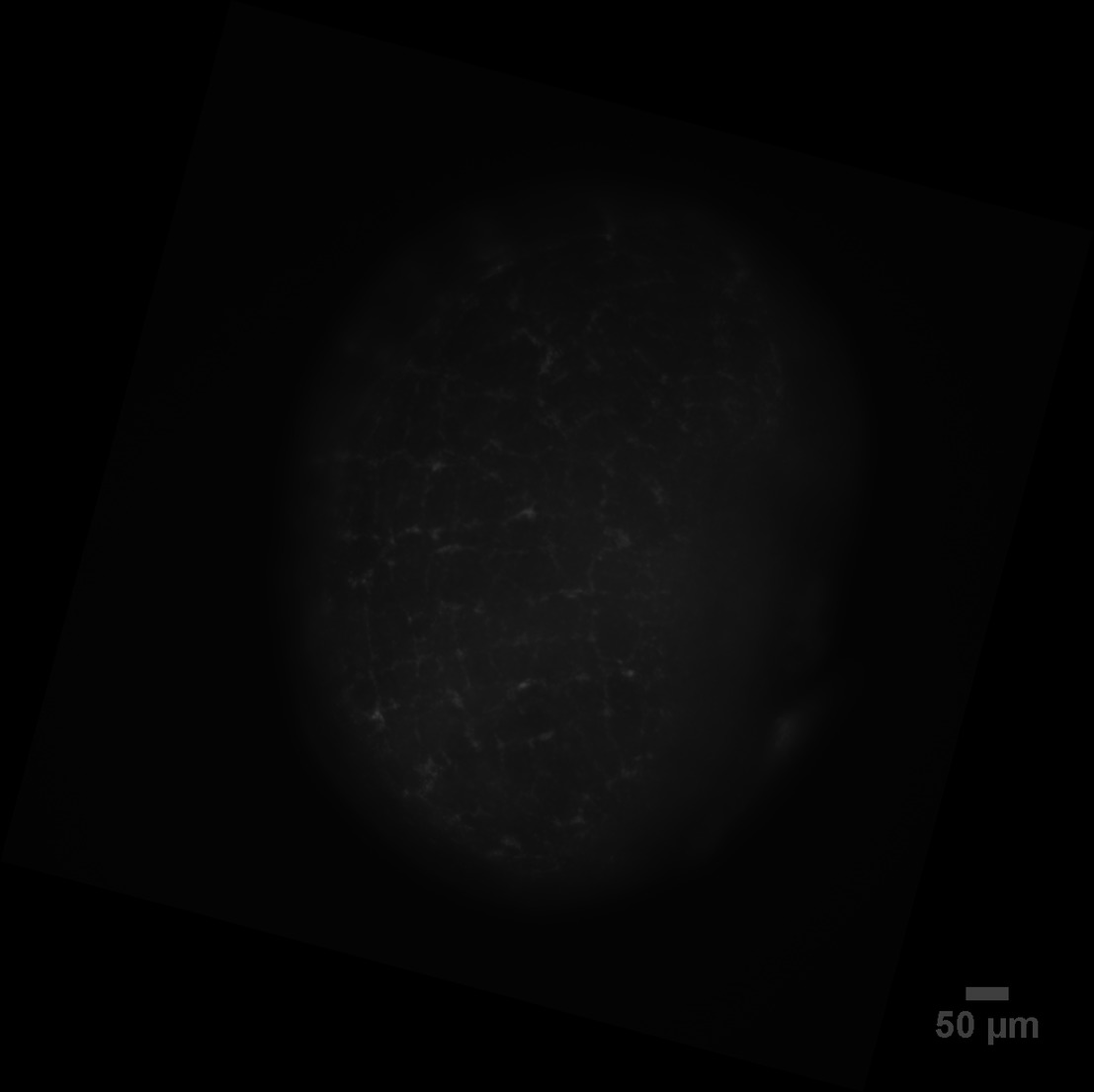

Supplement: S1 Dataset — This dataset contains brightfield image and corresponding synapsin stains for the VNC-free and VNC-containing small fragment cutting scenarios shown in Fig 6. Each image is labeled in the format “x_dpc_Sample_y_tn.jpg”, where “x” represents the number of days post cutting and “y” the replicate number. (ZIP) [file pcbi.1006904.s016.zip › smallfragments/VNC-free/synapsin_stains/2 dpc_Sample 4_tn.jpg]

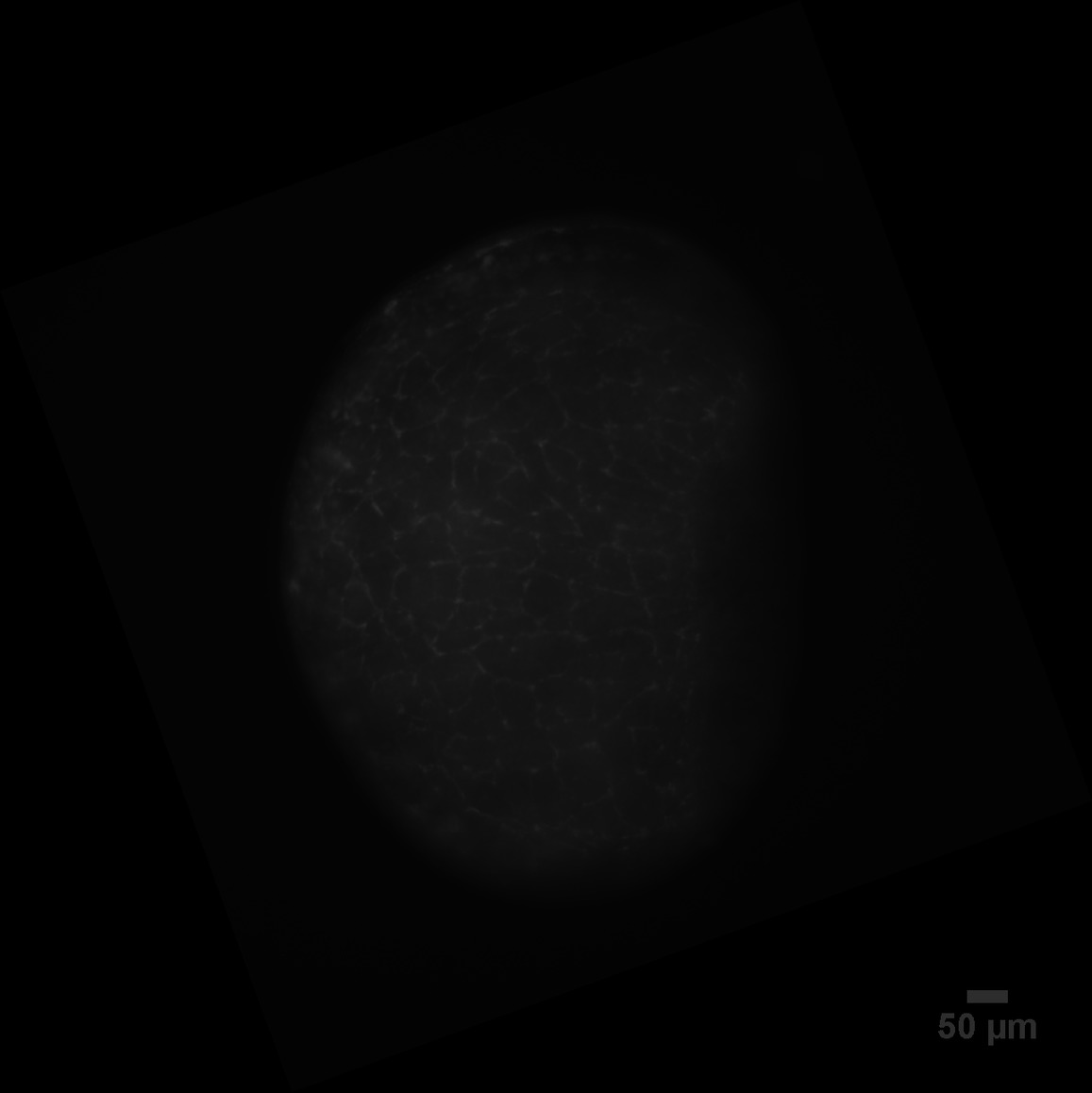

Supplement: S1 Dataset — This dataset contains brightfield image and corresponding synapsin stains for the VNC-free and VNC-containing small fragment cutting scenarios shown in Fig 6. Each image is labeled in the format “x_dpc_Sample_y_tn.jpg”, where “x” represents the number of days post cutting and “y” the replicate number. (ZIP) [file pcbi.1006904.s016.zip › smallfragments/VNC-free/synapsin_stains/2 dpc_Sample 5_tn.jpg]

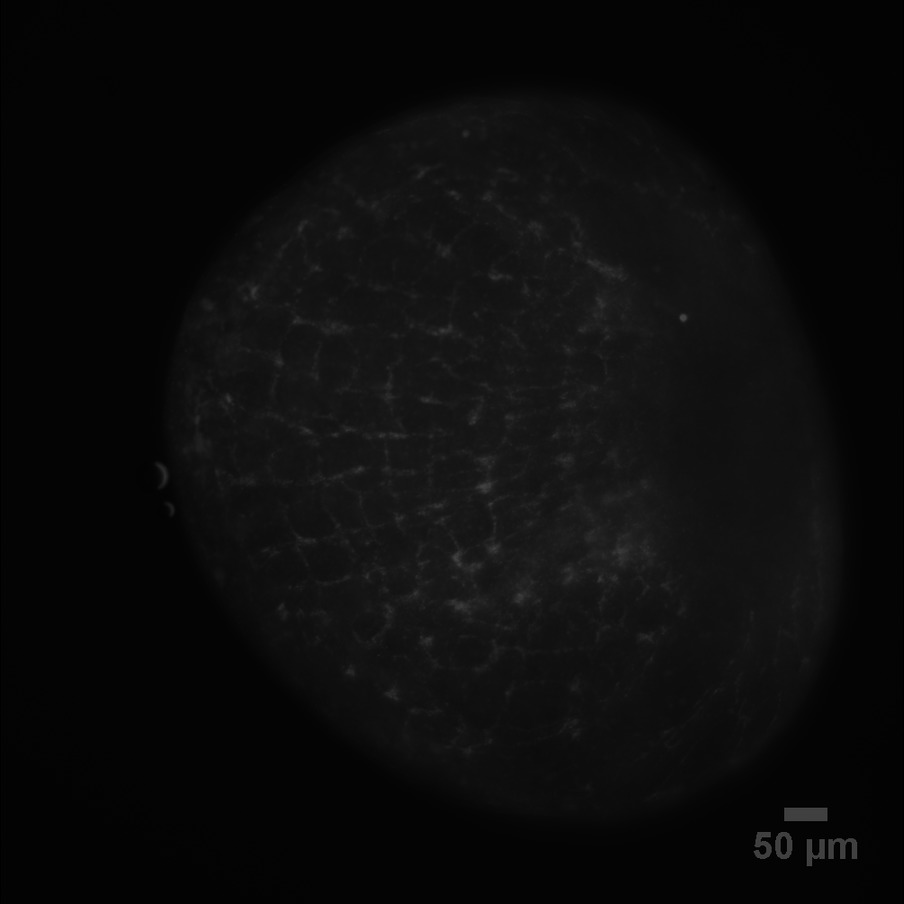

Supplement: S1 Dataset — This dataset contains brightfield image and corresponding synapsin stains for the VNC-free and VNC-containing small fragment cutting scenarios shown in Fig 6. Each image is labeled in the format “x_dpc_Sample_y_tn.jpg”, where “x” represents the number of days post cutting and “y” the replicate number. (ZIP) [file pcbi.1006904.s016.zip › smallfragments/VNC-free/synapsin_stains/2 dpc_Sample 6_tn.jpg]

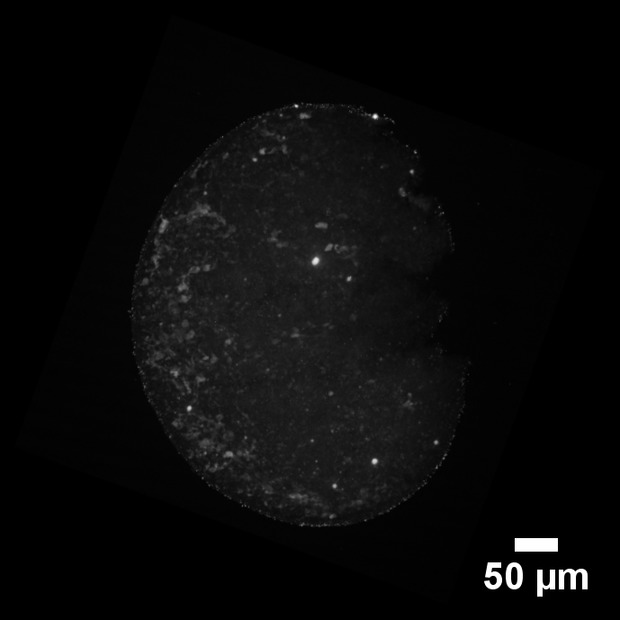

Supplement: S1 Dataset — This dataset contains brightfield image and corresponding synapsin stains for the VNC-free and VNC-containing small fragment cutting scenarios shown in Fig 6. Each image is labeled in the format “x_dpc_Sample_y_tn.jpg”, where “x” represents the number of days post cutting and “y” the replicate number. (ZIP) [file pcbi.1006904.s016.zip › smallfragments/VNC-free/synapsin_stains/2 dpc_Sample 7_tn.jpg]

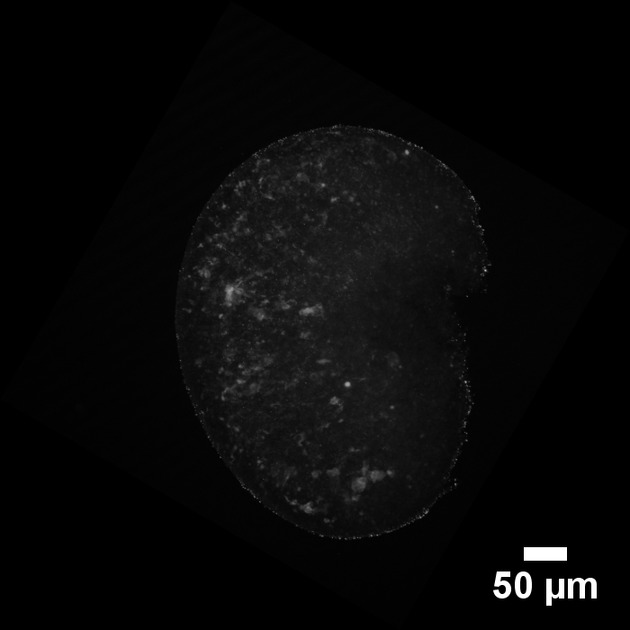

Supplement: S1 Dataset — This dataset contains brightfield image and corresponding synapsin stains for the VNC-free and VNC-containing small fragment cutting scenarios shown in Fig 6. Each image is labeled in the format “x_dpc_Sample_y_tn.jpg”, where “x” represents the number of days post cutting and “y” the replicate number. (ZIP) [file pcbi.1006904.s016.zip › smallfragments/VNC-free/synapsin_stains/2 dpc_Sample 8_tn.jpg]

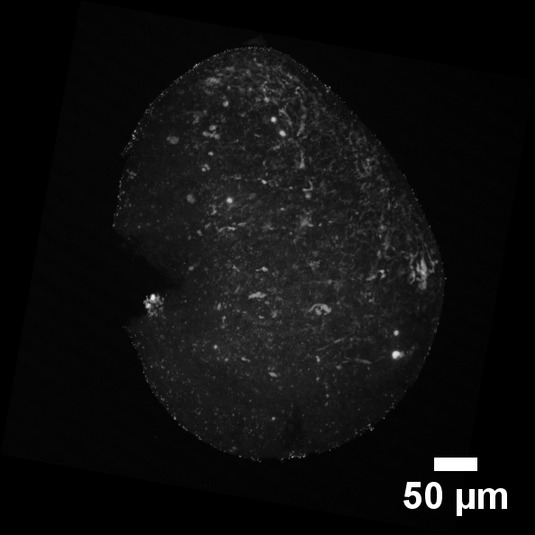

Supplement: S1 Dataset — This dataset contains brightfield image and corresponding synapsin stains for the VNC-free and VNC-containing small fragment cutting scenarios shown in Fig 6. Each image is labeled in the format “x_dpc_Sample_y_tn.jpg”, where “x” represents the number of days post cutting and “y” the replicate number. (ZIP) [file pcbi.1006904.s016.zip › smallfragments/VNC-free/synapsin_stains/2 dpc_Sample 9_tn.jpg]

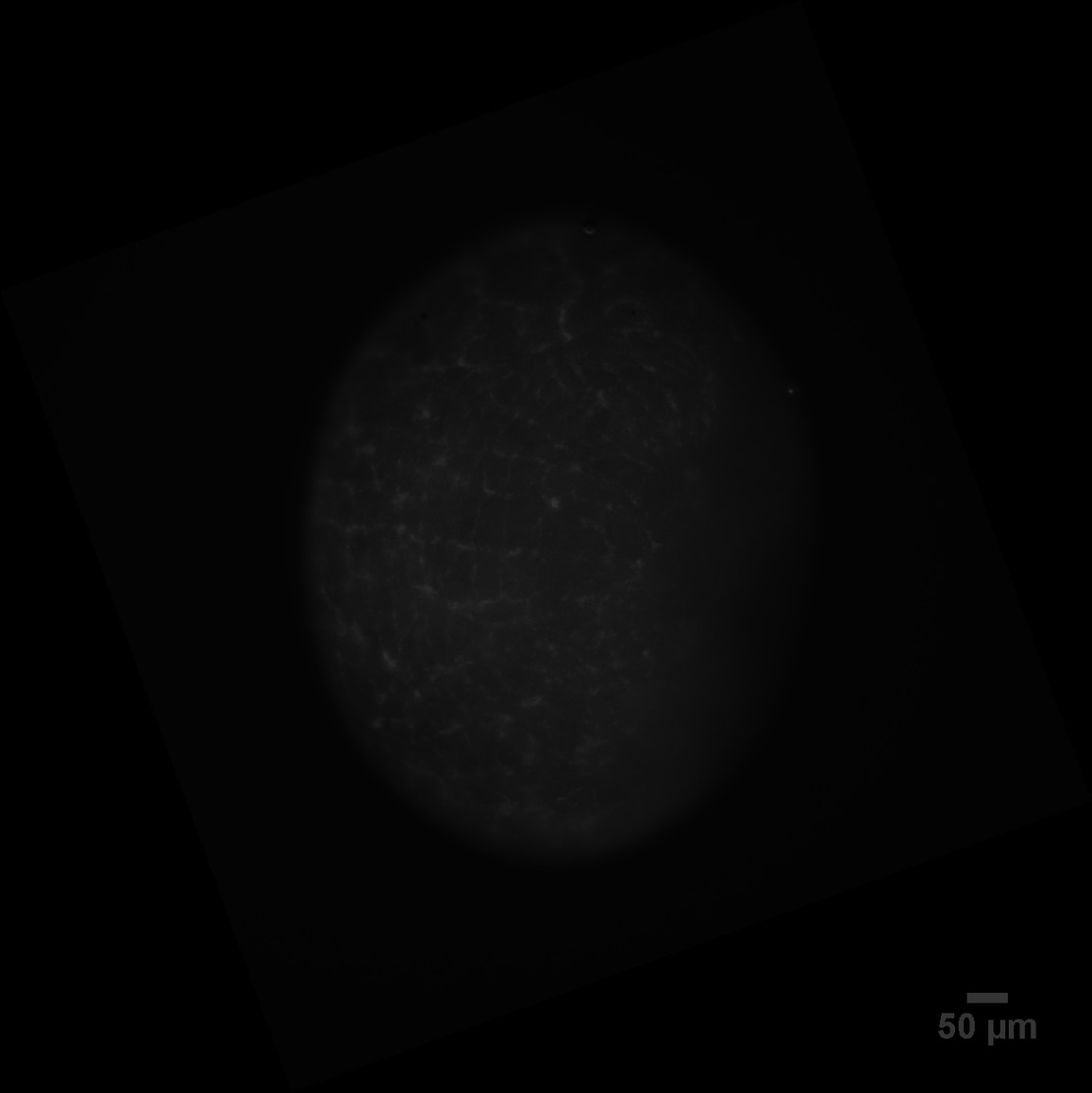

Supplement: S1 Dataset — This dataset contains brightfield image and corresponding synapsin stains for the VNC-free and VNC-containing small fragment cutting scenarios shown in Fig 6. Each image is labeled in the format “x_dpc_Sample_y_tn.jpg”, where “x” represents the number of days post cutting and “y” the replicate number. (ZIP) [file pcbi.1006904.s016.zip › smallfragments/VNC-free/synapsin_stains/3 dpc_Sample 1_tn.jpg]

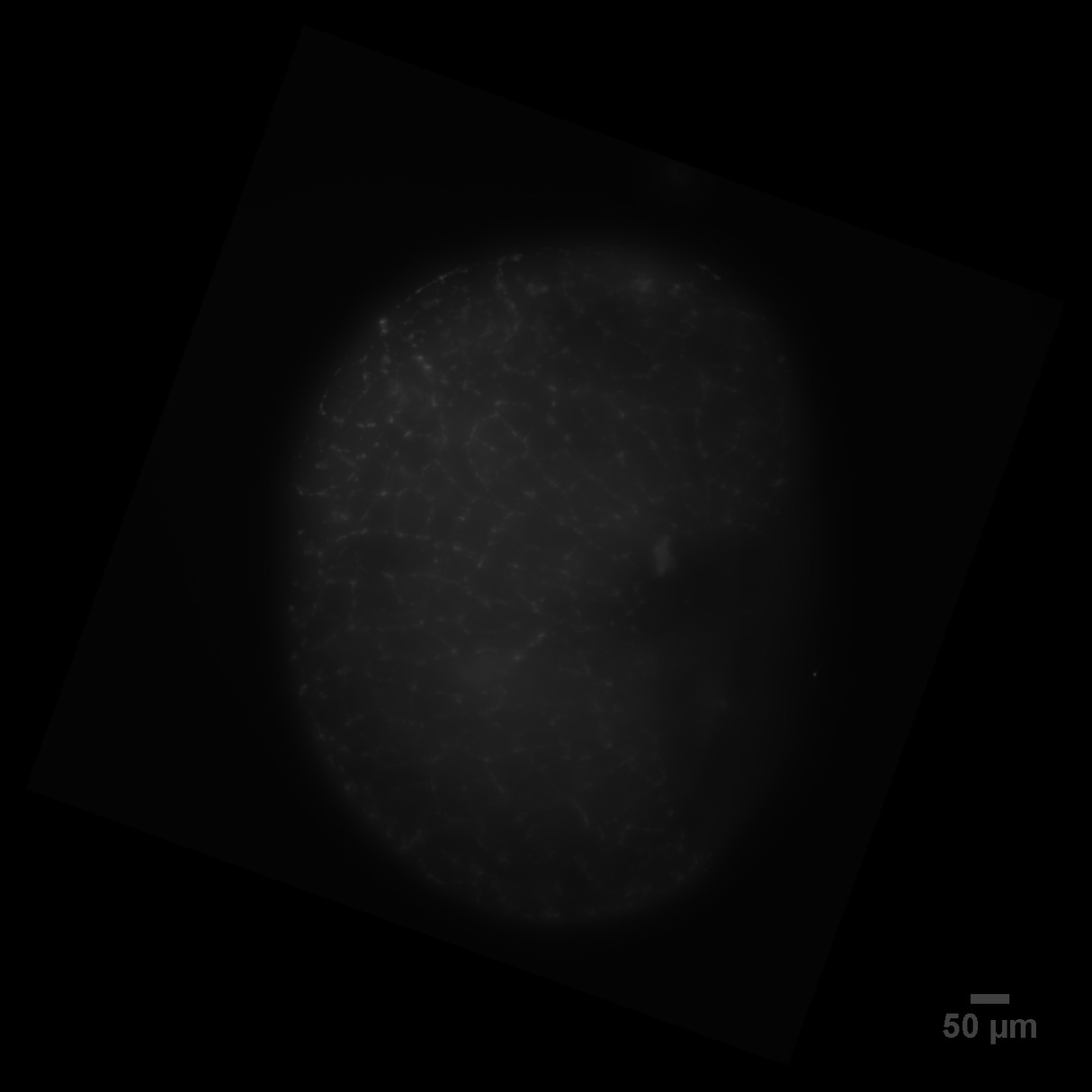

Supplement: S1 Dataset — This dataset contains brightfield image and corresponding synapsin stains for the VNC-free and VNC-containing small fragment cutting scenarios shown in Fig 6. Each image is labeled in the format “x_dpc_Sample_y_tn.jpg”, where “x” represents the number of days post cutting and “y” the replicate number. (ZIP) [file pcbi.1006904.s016.zip › smallfragments/VNC-free/synapsin_stains/3 dpc_Sample 2_tn.jpg]

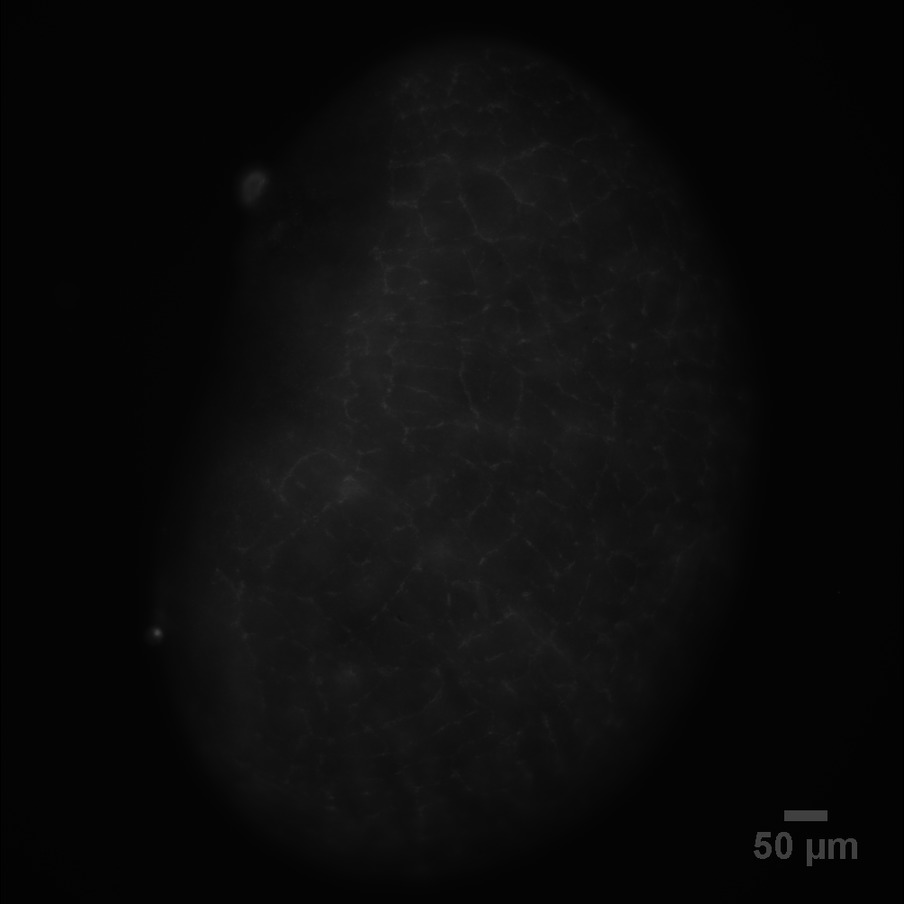

Supplement: S1 Dataset — This dataset contains brightfield image and corresponding synapsin stains for the VNC-free and VNC-containing small fragment cutting scenarios shown in Fig 6. Each image is labeled in the format “x_dpc_Sample_y_tn.jpg”, where “x” represents the number of days post cutting and “y” the replicate number. (ZIP) [file pcbi.1006904.s016.zip › smallfragments/VNC-free/synapsin_stains/3 dpc_Sample 3_tn.jpg]

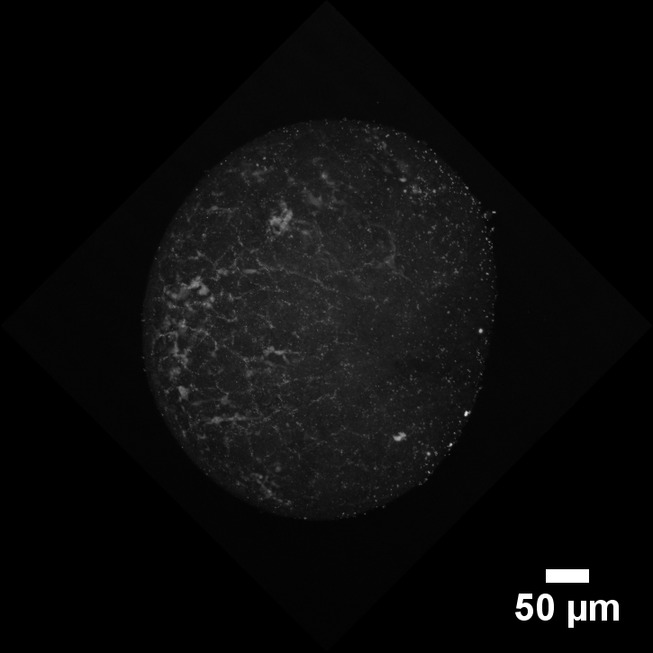

Supplement: S1 Dataset — This dataset contains brightfield image and corresponding synapsin stains for the VNC-free and VNC-containing small fragment cutting scenarios shown in Fig 6. Each image is labeled in the format “x_dpc_Sample_y_tn.jpg”, where “x” represents the number of days post cutting and “y” the replicate number. (ZIP) [file pcbi.1006904.s016.zip › smallfragments/VNC-free/synapsin_stains/3 dpc_Sample 4_tn.jpg]

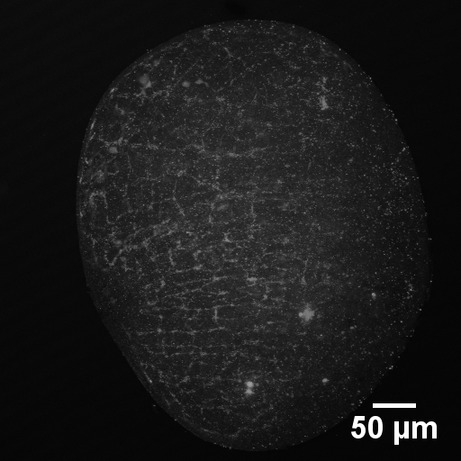

Supplement: S1 Dataset — This dataset contains brightfield image and corresponding synapsin stains for the VNC-free and VNC-containing small fragment cutting scenarios shown in Fig 6. Each image is labeled in the format “x_dpc_Sample_y_tn.jpg”, where “x” represents the number of days post cutting and “y” the replicate number. (ZIP) [file pcbi.1006904.s016.zip › smallfragments/VNC-free/synapsin_stains/3 dpc_Sample 5_tn.jpg]

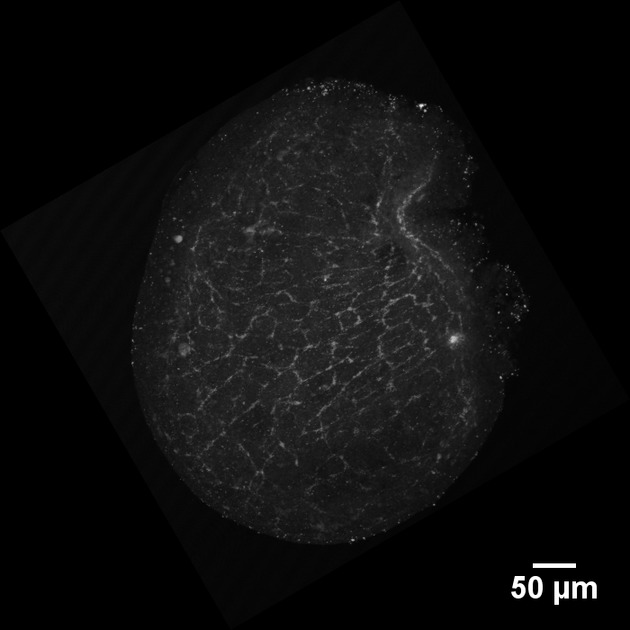

Supplement: S1 Dataset — This dataset contains brightfield image and corresponding synapsin stains for the VNC-free and VNC-containing small fragment cutting scenarios shown in Fig 6. Each image is labeled in the format “x_dpc_Sample_y_tn.jpg”, where “x” represents the number of days post cutting and “y” the replicate number. (ZIP) [file pcbi.1006904.s016.zip › smallfragments/VNC-free/synapsin_stains/3 dpc_Sample 6_tn.jpg]

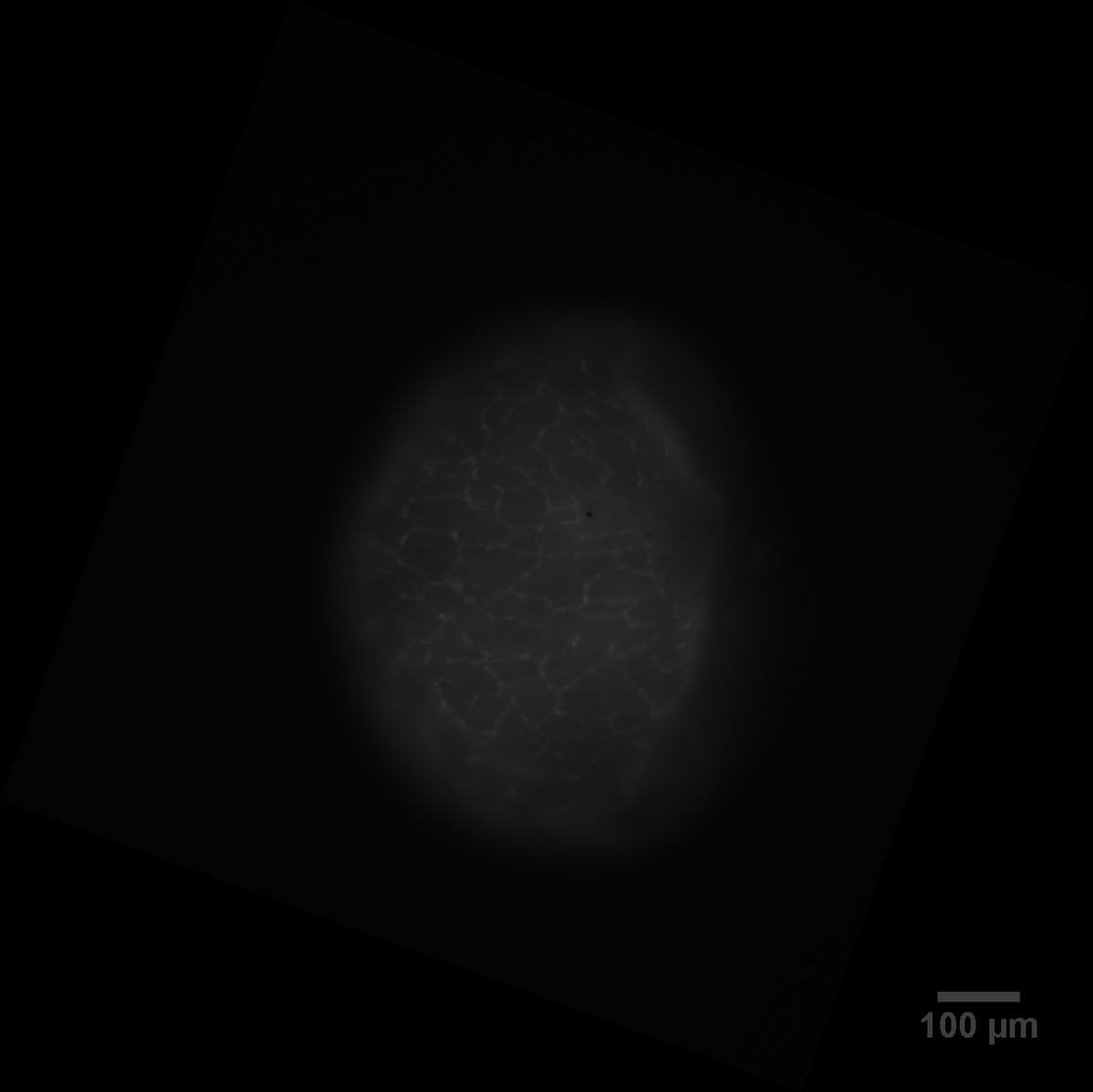

Supplement: S1 Dataset — This dataset contains brightfield image and corresponding synapsin stains for the VNC-free and VNC-containing small fragment cutting scenarios shown in Fig 6. Each image is labeled in the format “x_dpc_Sample_y_tn.jpg”, where “x” represents the number of days post cutting and “y” the replicate number. (ZIP) [file pcbi.1006904.s016.zip › smallfragments/VNC-free/synapsin_stains/4 dpc_Sample 1_tn.jpg]

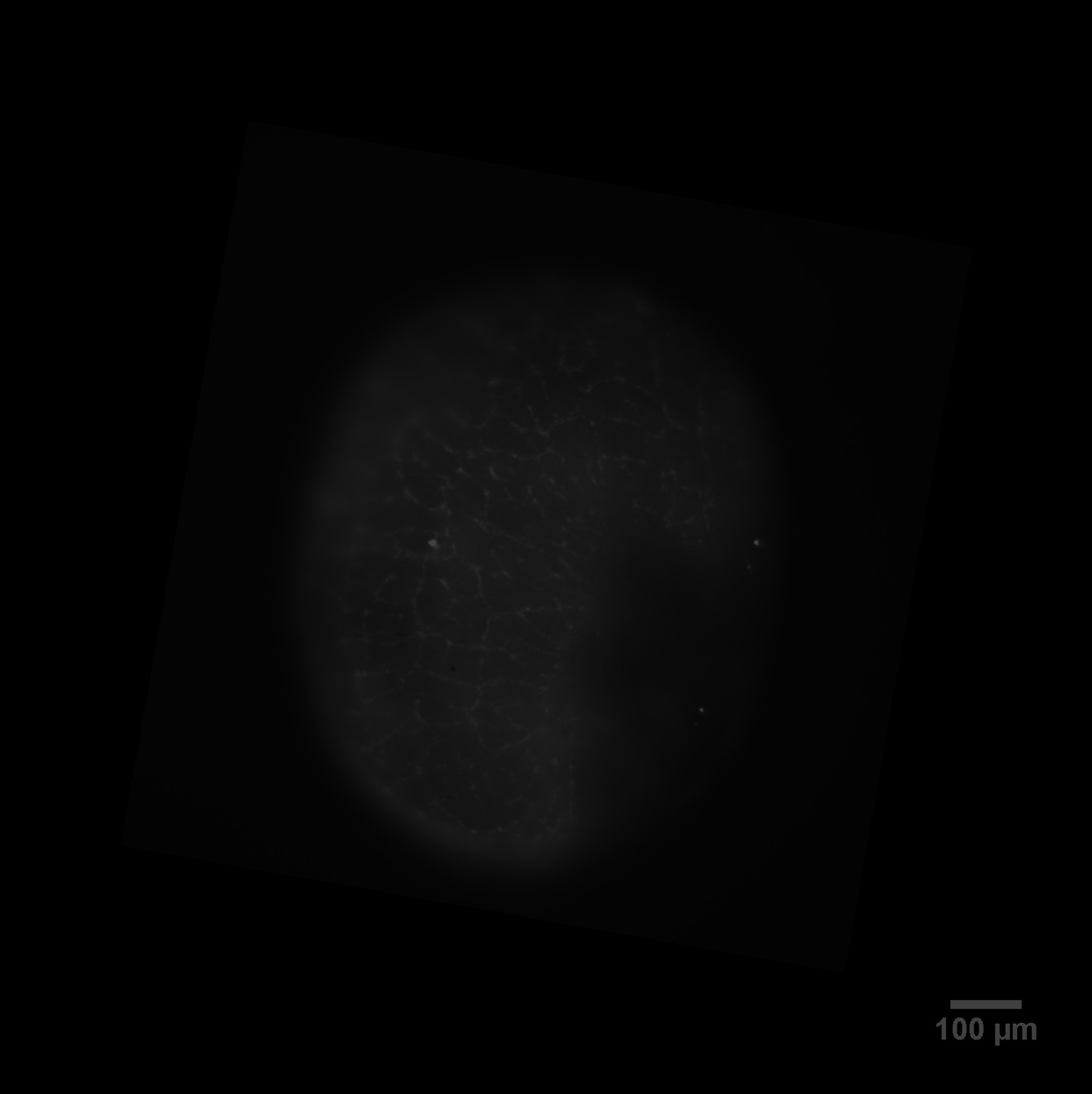

Supplement: S1 Dataset — This dataset contains brightfield image and corresponding synapsin stains for the VNC-free and VNC-containing small fragment cutting scenarios shown in Fig 6. Each image is labeled in the format “x_dpc_Sample_y_tn.jpg”, where “x” represents the number of days post cutting and “y” the replicate number. (ZIP) [file pcbi.1006904.s016.zip › smallfragments/VNC-free/synapsin_stains/4 dpc_Sample 2_tn.jpg]

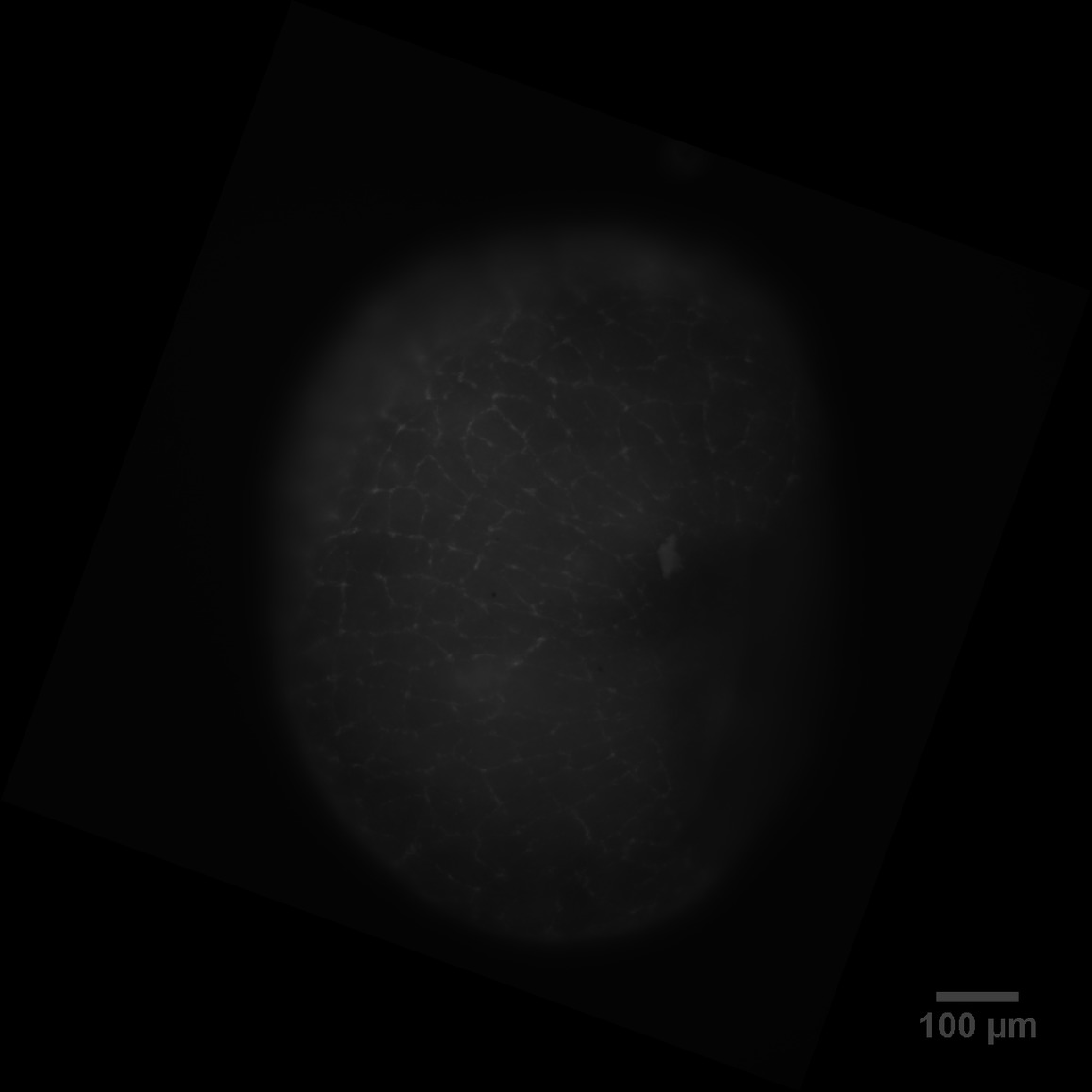

Supplement: S1 Dataset — This dataset contains brightfield image and corresponding synapsin stains for the VNC-free and VNC-containing small fragment cutting scenarios shown in Fig 6. Each image is labeled in the format “x_dpc_Sample_y_tn.jpg”, where “x” represents the number of days post cutting and “y” the replicate number. (ZIP) [file pcbi.1006904.s016.zip › smallfragments/VNC-free/synapsin_stains/4 dpc_Sample 3_tn.jpg]

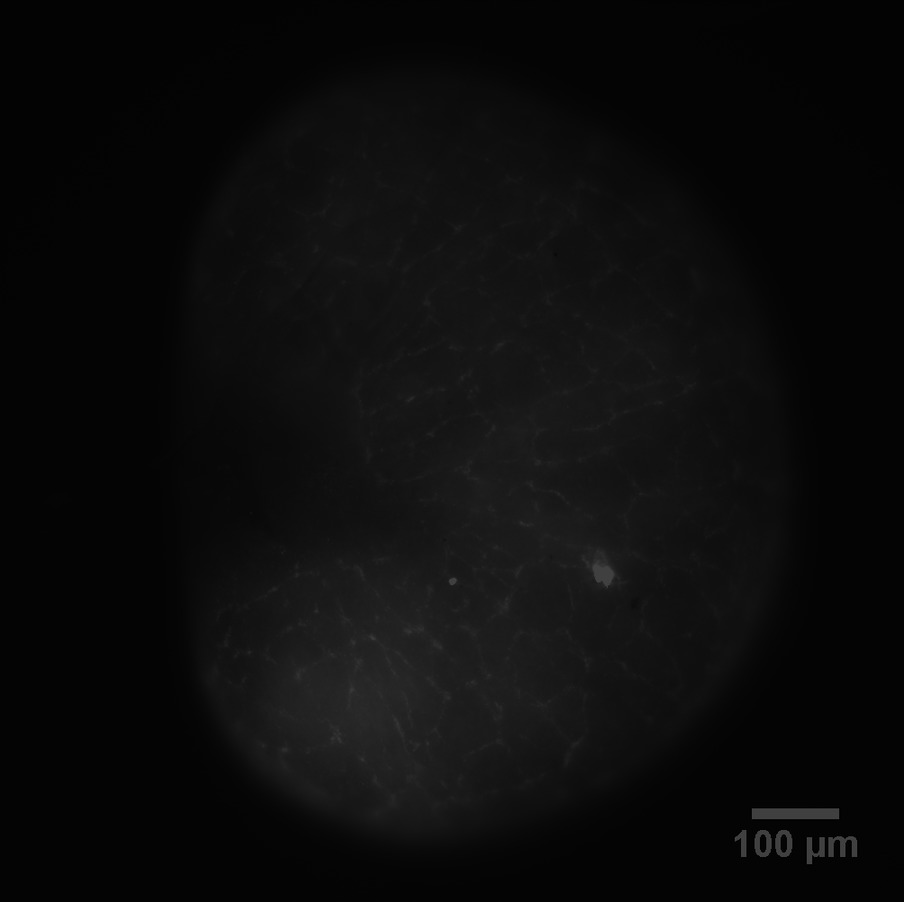

Supplement: S1 Dataset — This dataset contains brightfield image and corresponding synapsin stains for the VNC-free and VNC-containing small fragment cutting scenarios shown in Fig 6. Each image is labeled in the format “x_dpc_Sample_y_tn.jpg”, where “x” represents the number of days post cutting and “y” the replicate number. (ZIP) [file pcbi.1006904.s016.zip › smallfragments/VNC-free/synapsin_stains/4 dpc_Sample 4_tn.jpg]

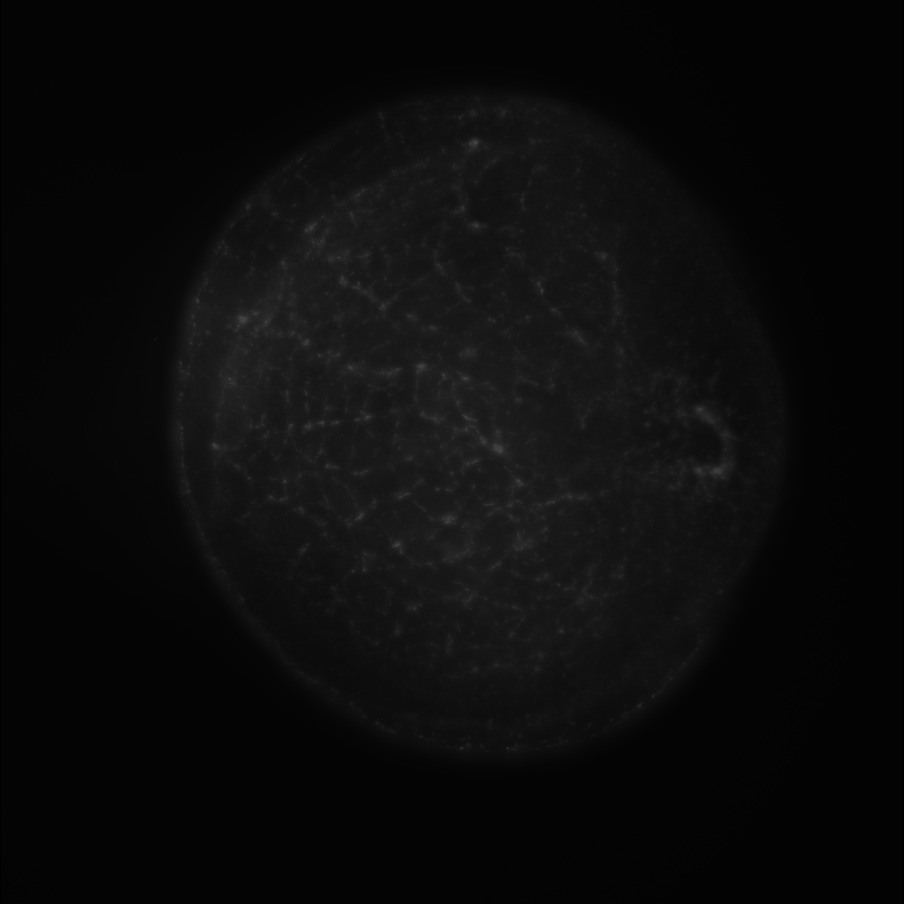

Supplement: S1 Dataset — This dataset contains brightfield image and corresponding synapsin stains for the VNC-free and VNC-containing small fragment cutting scenarios shown in Fig 6. Each image is labeled in the format “x_dpc_Sample_y_tn.jpg”, where “x” represents the number of days post cutting and “y” the replicate number. (ZIP) [file pcbi.1006904.s016.zip › smallfragments/VNC-free/synapsin_stains/4 dpc_Sample 5_tn.jpg]

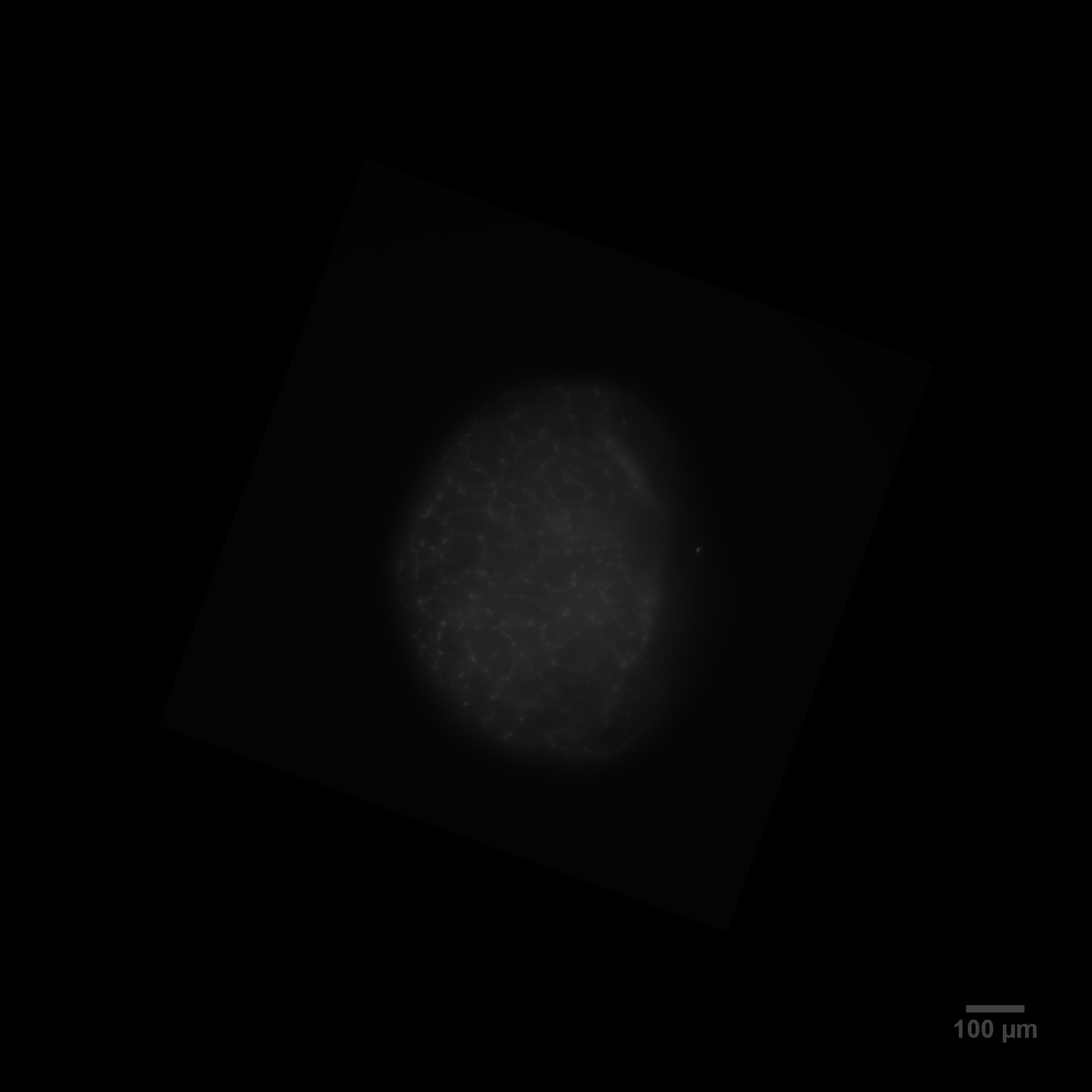

Supplement: S1 Dataset — This dataset contains brightfield image and corresponding synapsin stains for the VNC-free and VNC-containing small fragment cutting scenarios shown in Fig 6. Each image is labeled in the format “x_dpc_Sample_y_tn.jpg”, where “x” represents the number of days post cutting and “y” the replicate number. (ZIP) [file pcbi.1006904.s016.zip › smallfragments/VNC-free/synapsin_stains/4 dpc_Sample 6_tn.jpg]

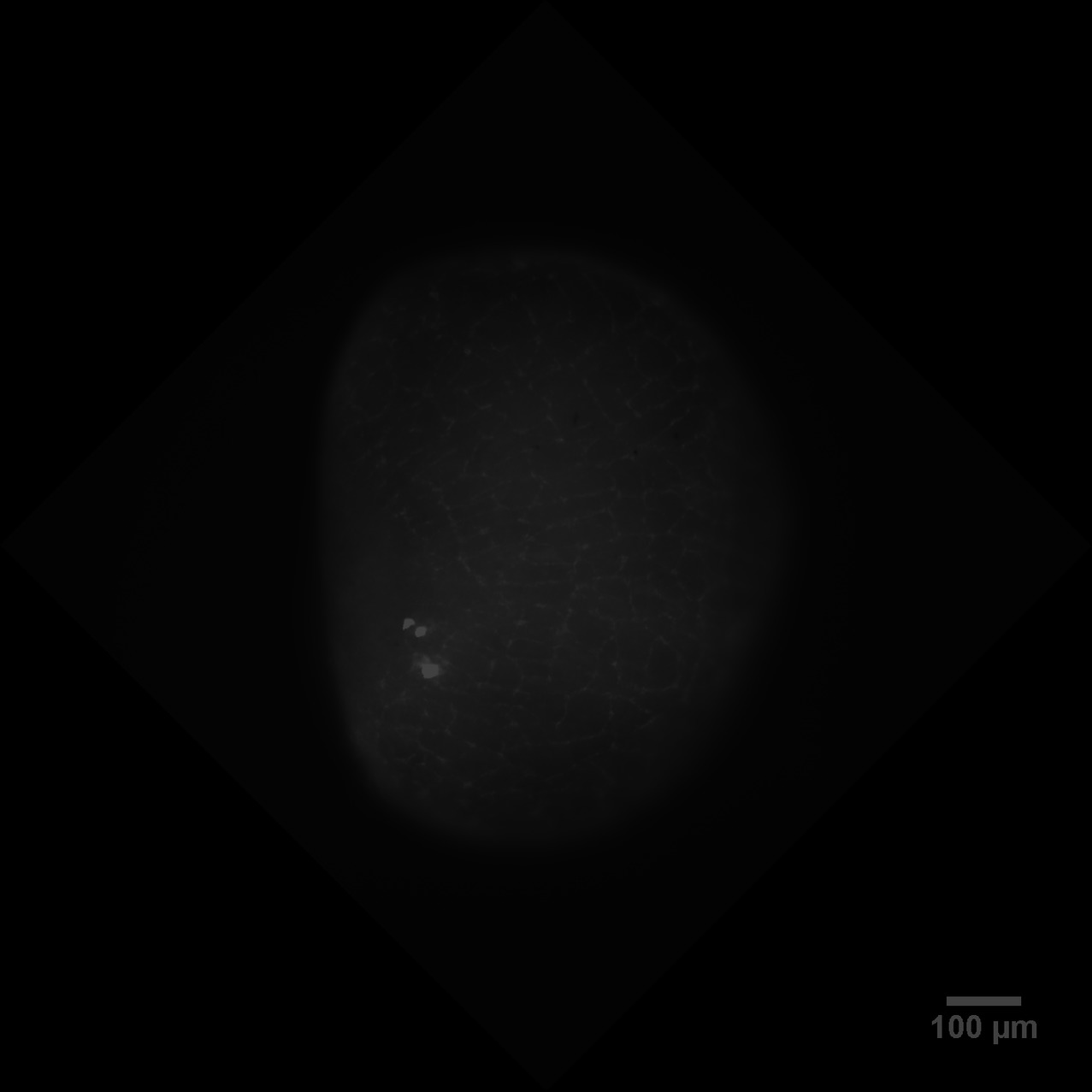

Supplement: S1 Dataset — This dataset contains brightfield image and corresponding synapsin stains for the VNC-free and VNC-containing small fragment cutting scenarios shown in Fig 6. Each image is labeled in the format “x_dpc_Sample_y_tn.jpg”, where “x” represents the number of days post cutting and “y” the replicate number. (ZIP) [file pcbi.1006904.s016.zip › smallfragments/VNC-free/synapsin_stains/4 dpc_Sample 7_tn.jpg]

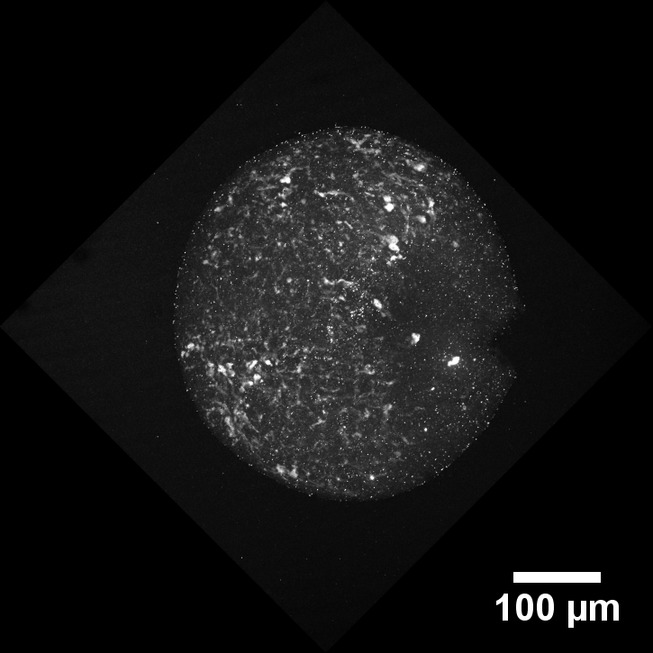

Supplement: S1 Dataset — This dataset contains brightfield image and corresponding synapsin stains for the VNC-free and VNC-containing small fragment cutting scenarios shown in Fig 6. Each image is labeled in the format “x_dpc_Sample_y_tn.jpg”, where “x” represents the number of days post cutting and “y” the replicate number. (ZIP) [file pcbi.1006904.s016.zip › smallfragments/VNC-free/synapsin_stains/4 dpc_Sample 8_tn.jpg]

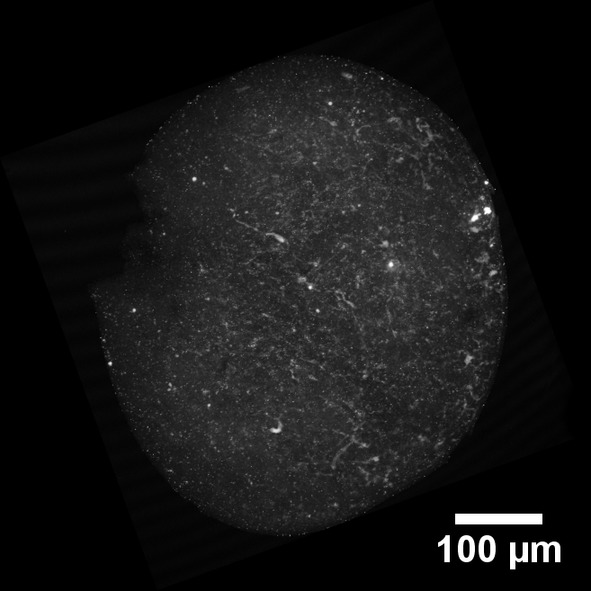

Supplement: S1 Dataset — This dataset contains brightfield image and corresponding synapsin stains for the VNC-free and VNC-containing small fragment cutting scenarios shown in Fig 6. Each image is labeled in the format “x_dpc_Sample_y_tn.jpg”, where “x” represents the number of days post cutting and “y” the replicate number. (ZIP) [file pcbi.1006904.s016.zip › smallfragments/VNC-free/synapsin_stains/4 dpc_Sample 9_tn.jpg]

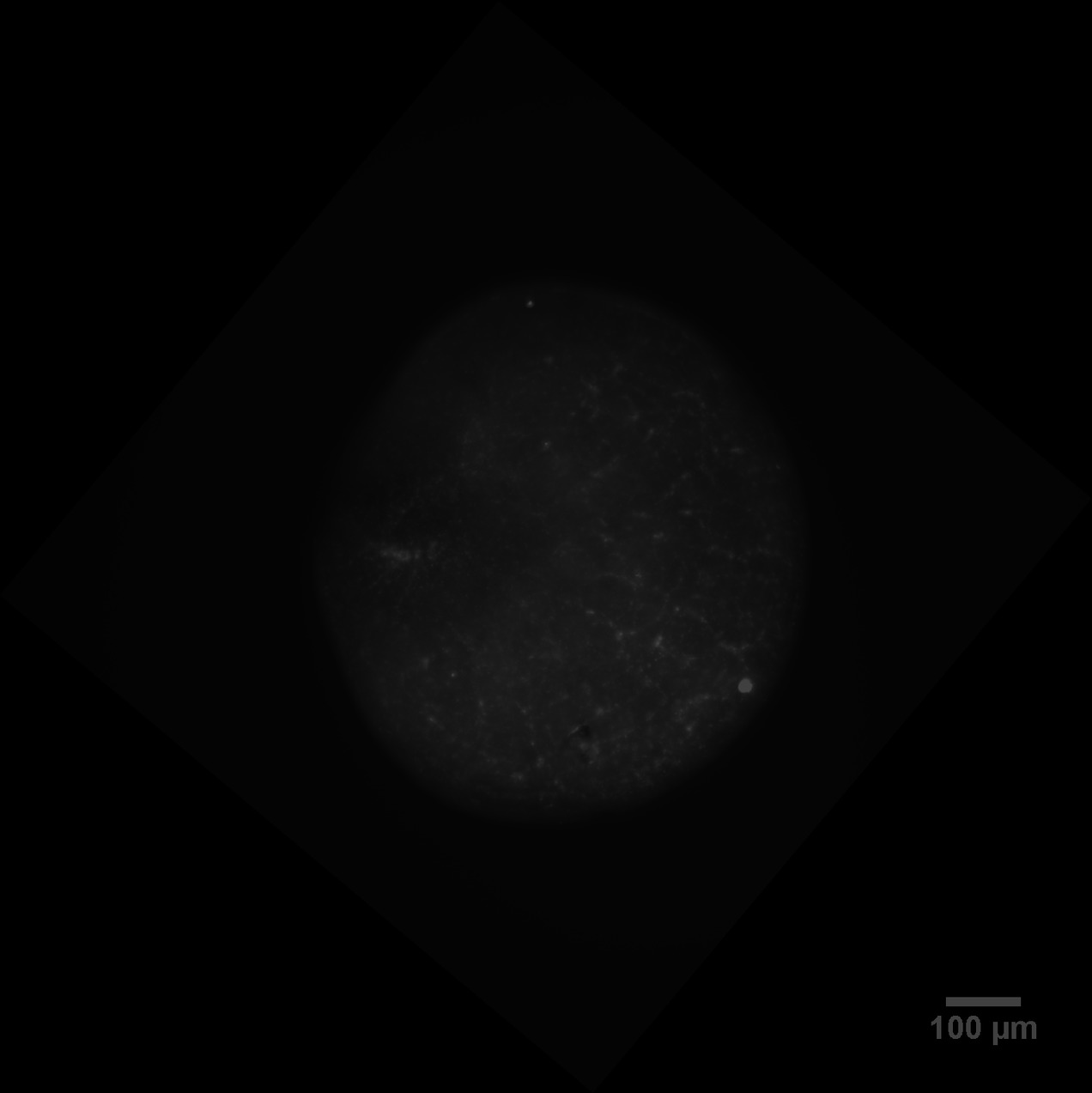

Supplement: S1 Dataset — This dataset contains brightfield image and corresponding synapsin stains for the VNC-free and VNC-containing small fragment cutting scenarios shown in Fig 6. Each image is labeled in the format “x_dpc_Sample_y_tn.jpg”, where “x” represents the number of days post cutting and “y” the replicate number. (ZIP) [file pcbi.1006904.s016.zip › smallfragments/VNC-free/synapsin_stains/5 dpc_Sample 1_tn.jpg]

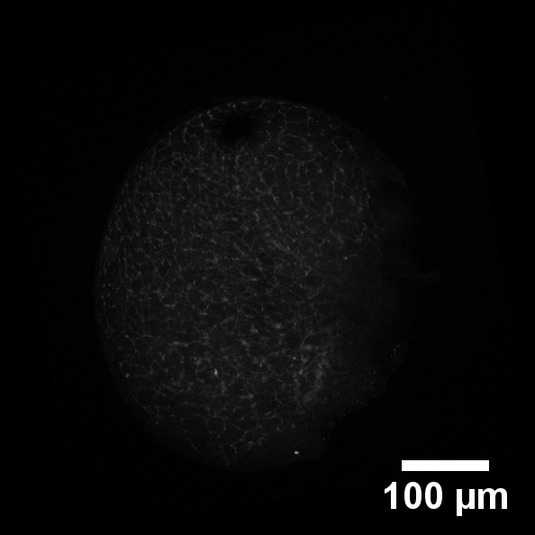

Supplement: S1 Dataset — This dataset contains brightfield image and corresponding synapsin stains for the VNC-free and VNC-containing small fragment cutting scenarios shown in Fig 6. Each image is labeled in the format “x_dpc_Sample_y_tn.jpg”, where “x” represents the number of days post cutting and “y” the replicate number. (ZIP) [file pcbi.1006904.s016.zip › smallfragments/VNC-free/synapsin_stains/5 dpc_Sample 2_tn.jpg]

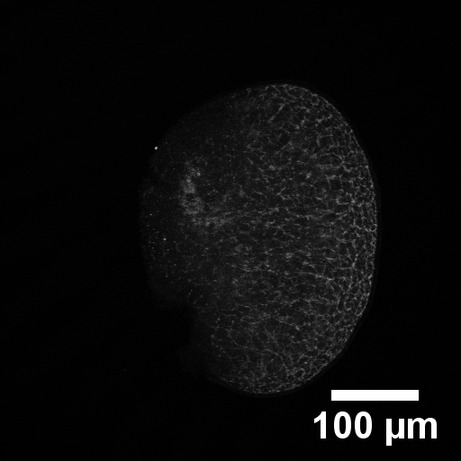

Supplement: S1 Dataset — This dataset contains brightfield image and corresponding synapsin stains for the VNC-free and VNC-containing small fragment cutting scenarios shown in Fig 6. Each image is labeled in the format “x_dpc_Sample_y_tn.jpg”, where “x” represents the number of days post cutting and “y” the replicate number. (ZIP) [file pcbi.1006904.s016.zip › smallfragments/VNC-free/synapsin_stains/5 dpc_Sample 3_tn.jpg]

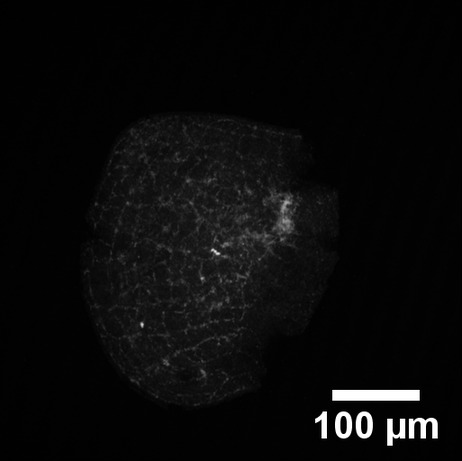

Supplement: S1 Dataset — This dataset contains brightfield image and corresponding synapsin stains for the VNC-free and VNC-containing small fragment cutting scenarios shown in Fig 6. Each image is labeled in the format “x_dpc_Sample_y_tn.jpg”, where “x” represents the number of days post cutting and “y” the replicate number. (ZIP) [file pcbi.1006904.s016.zip › smallfragments/VNC-free/synapsin_stains/5 dpc_Sample 4_tn.jpg]

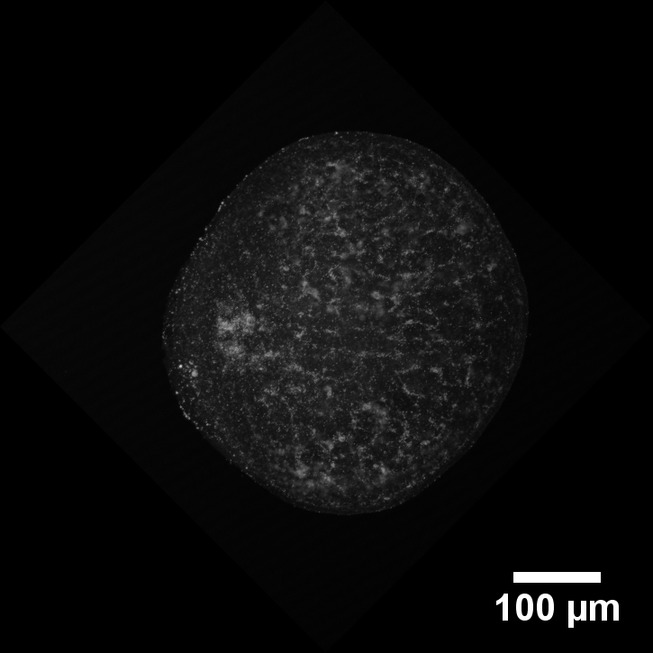

Supplement: S1 Dataset — This dataset contains brightfield image and corresponding synapsin stains for the VNC-free and VNC-containing small fragment cutting scenarios shown in Fig 6. Each image is labeled in the format “x_dpc_Sample_y_tn.jpg”, where “x” represents the number of days post cutting and “y” the replicate number. (ZIP) [file pcbi.1006904.s016.zip › smallfragments/VNC-free/synapsin_stains/6 dpc_Sample 1_tn.jpg]

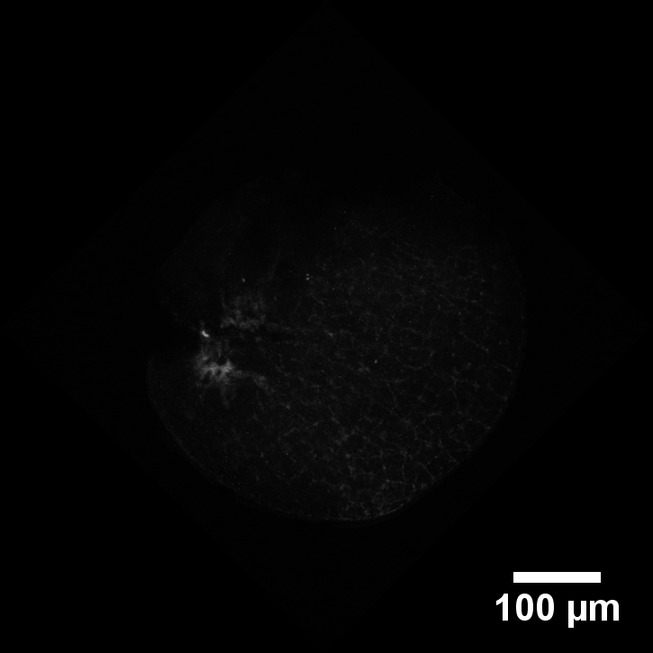

Supplement: S1 Dataset — This dataset contains brightfield image and corresponding synapsin stains for the VNC-free and VNC-containing small fragment cutting scenarios shown in Fig 6. Each image is labeled in the format “x_dpc_Sample_y_tn.jpg”, where “x” represents the number of days post cutting and “y” the replicate number. (ZIP) [file pcbi.1006904.s016.zip › smallfragments/VNC-free/synapsin_stains/6 dpc_Sample 2_tn.jpg]

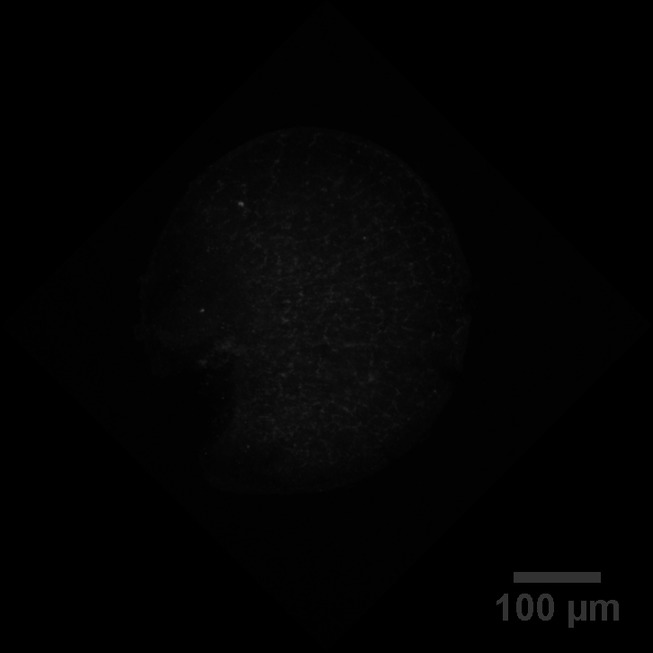

Supplement: S1 Dataset — This dataset contains brightfield image and corresponding synapsin stains for the VNC-free and VNC-containing small fragment cutting scenarios shown in Fig 6. Each image is labeled in the format “x_dpc_Sample_y_tn.jpg”, where “x” represents the number of days post cutting and “y” the replicate number. (ZIP) [file pcbi.1006904.s016.zip › smallfragments/VNC-free/synapsin_stains/6 dpc_Sample 3_tn.jpg]

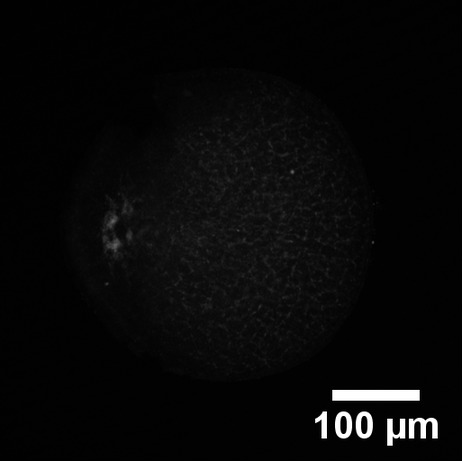

Supplement: S1 Dataset — This dataset contains brightfield image and corresponding synapsin stains for the VNC-free and VNC-containing small fragment cutting scenarios shown in Fig 6. Each image is labeled in the format “x_dpc_Sample_y_tn.jpg”, where “x” represents the number of days post cutting and “y” the replicate number. (ZIP) [file pcbi.1006904.s016.zip › smallfragments/VNC-free/synapsin_stains/6 dpc_Sample 4_tn.jpg]

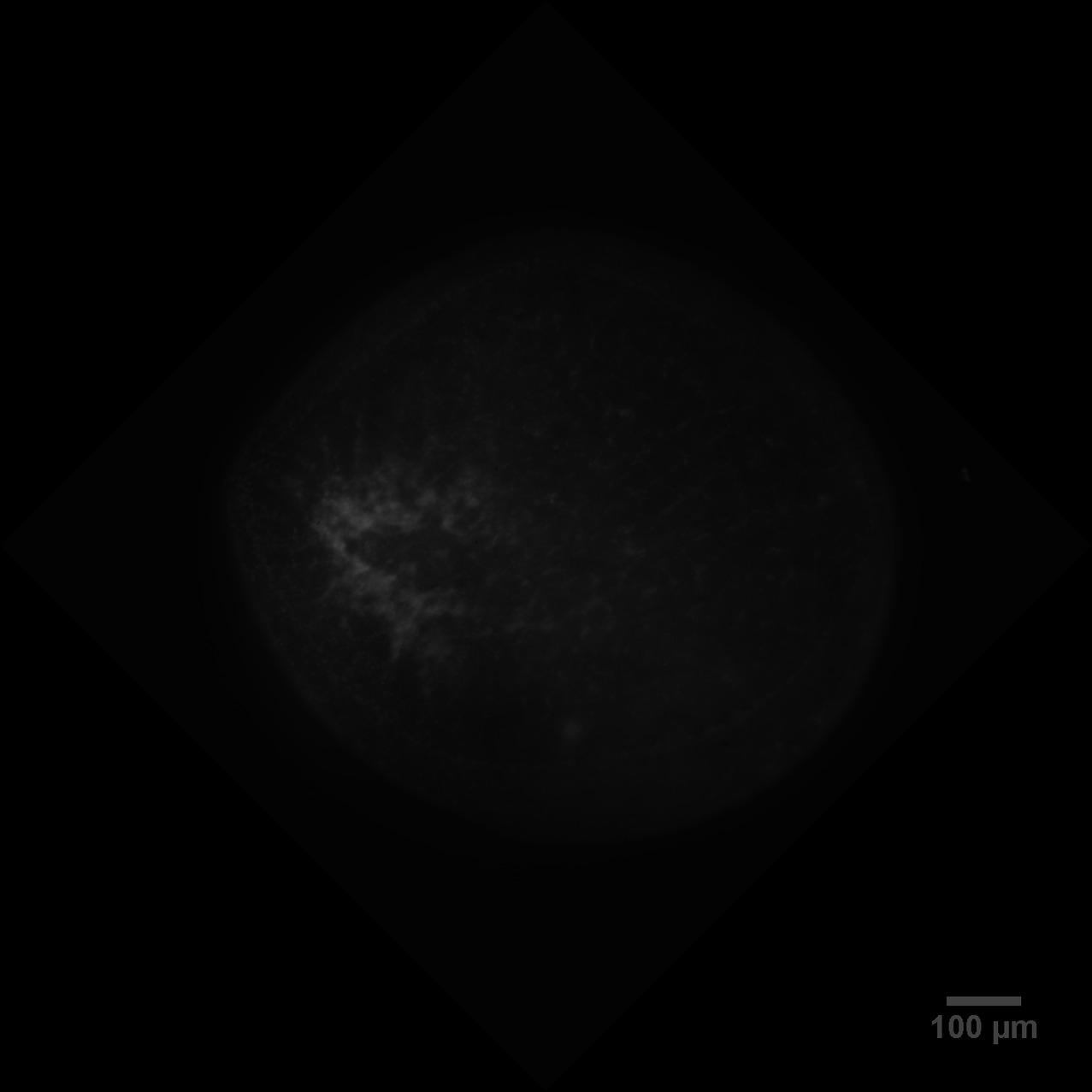

Supplement: S1 Dataset — This dataset contains brightfield image and corresponding synapsin stains for the VNC-free and VNC-containing small fragment cutting scenarios shown in Fig 6. Each image is labeled in the format “x_dpc_Sample_y_tn.jpg”, where “x” represents the number of days post cutting and “y” the replicate number. (ZIP) [file pcbi.1006904.s016.zip › smallfragments/VNC-free/synapsin_stains/7 dpc_Sample 1_tn.jpg]

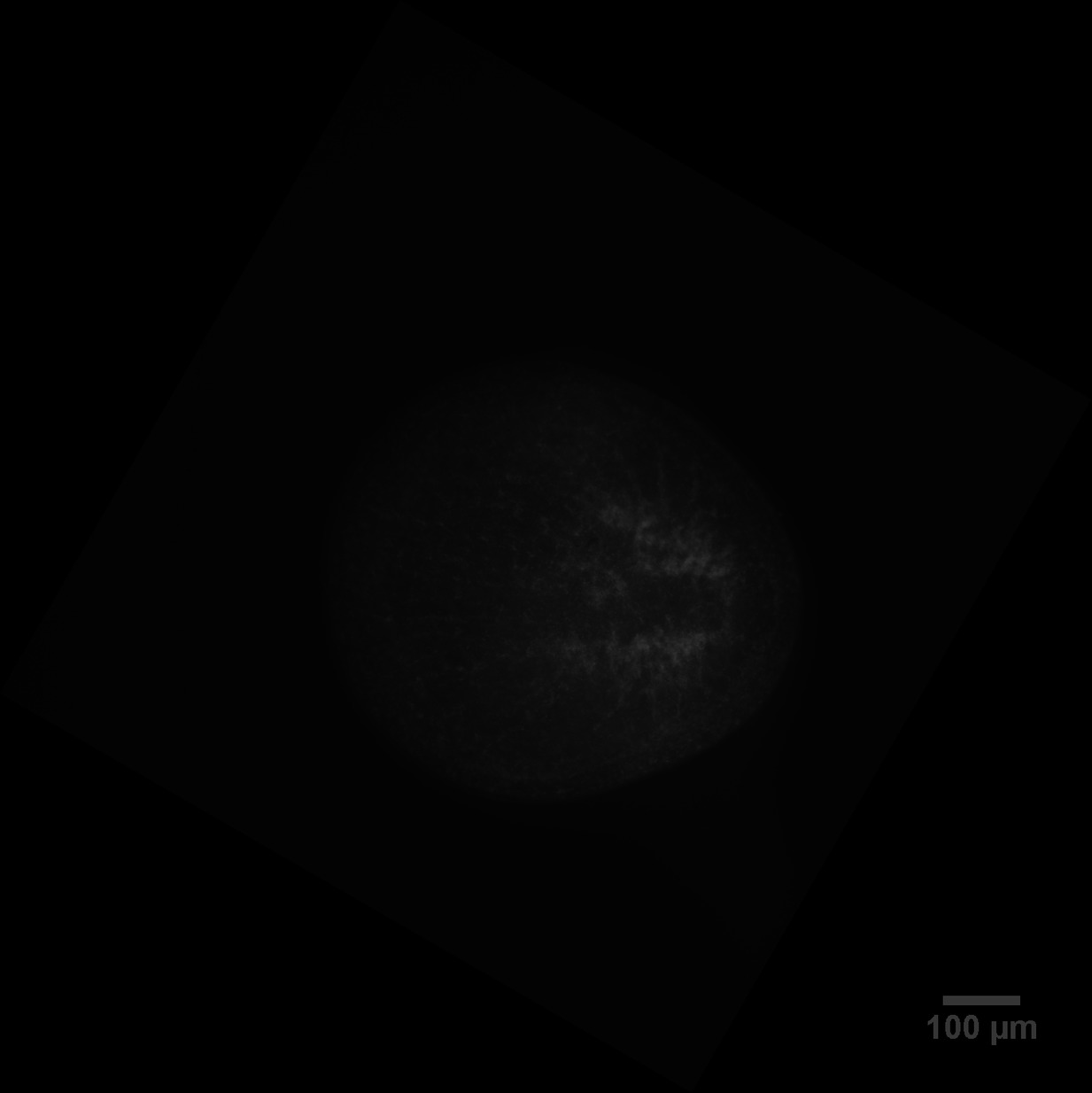

Supplement: S1 Dataset — This dataset contains brightfield image and corresponding synapsin stains for the VNC-free and VNC-containing small fragment cutting scenarios shown in Fig 6. Each image is labeled in the format “x_dpc_Sample_y_tn.jpg”, where “x” represents the number of days post cutting and “y” the replicate number. (ZIP) [file pcbi.1006904.s016.zip › smallfragments/VNC-free/synapsin_stains/7 dpc_Sample 2_tn.jpg]

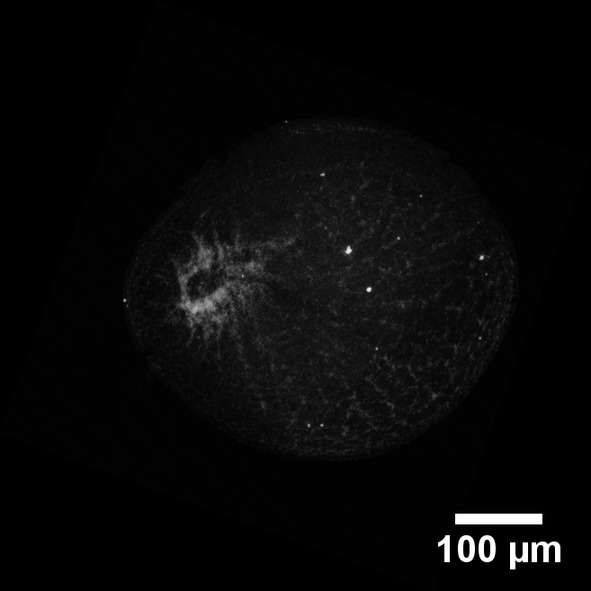

Supplement: S1 Dataset — This dataset contains brightfield image and corresponding synapsin stains for the VNC-free and VNC-containing small fragment cutting scenarios shown in Fig 6. Each image is labeled in the format “x_dpc_Sample_y_tn.jpg”, where “x” represents the number of days post cutting and “y” the replicate number. (ZIP) [file pcbi.1006904.s016.zip › smallfragments/VNC-free/synapsin_stains/7 dpc_Sample 3_tn.jpg]

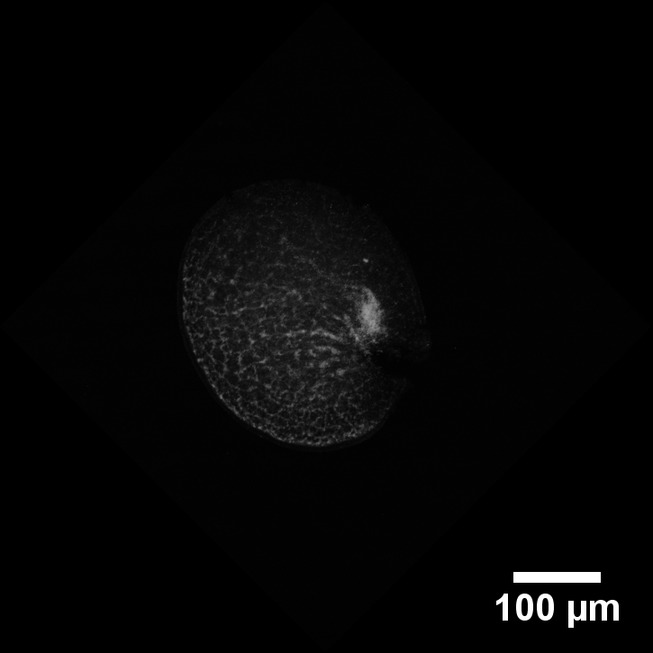

Supplement: S1 Dataset — This dataset contains brightfield image and corresponding synapsin stains for the VNC-free and VNC-containing small fragment cutting scenarios shown in Fig 6. Each image is labeled in the format “x_dpc_Sample_y_tn.jpg”, where “x” represents the number of days post cutting and “y” the replicate number. (ZIP) [file pcbi.1006904.s016.zip › smallfragments/VNC-free/synapsin_stains/7 dpc_Sample 4_tn.jpg]
