# Supplementary material for: Neural control of body-plan axis in regenerating planaria
Source: PLoS Comput Biol. 2019 Apr 16;15(4):e1006904. doi: 10.1371/journal.pcbi.1006904 (PMC6485777; doi:10.1371/journal.pcbi.1006904)
Supplement: S2 Dataset — This dataset contains raw-images of synapsin stains of uncut one- and two- headed worms, synapsin stains and brightfield images of the upwards and inverted L-cut scenarios, and synapsin stains and brightfield images showing the effects of the dynein inhibitor Ciliobrevin D on planaria regeneration. A Word document contained in the zip folder provides detailed description of the different cases. (ZIP) [file pcbi.1006904.s017.zip › DatasetS9i/Supplement Image description.docx]

This supplementary image data package contains additional images of experimental outcomes for the manuscript “Neural Control of Body-plan Axis Specification in Regenerating Planaria”

Data for the following experiments is included here:

[1. Synapsin stain of single-headed wildtype planaria – Figure 1 2](#_Toc525269039)

[2. Synapsin stain of double-headed wildtype planaria – Figure 7 2](#_Toc525269040)

[3. Dynein inhibition – Ciliobrevin D treatment – Figure 10 (or Supp Fig 3?) 2](#_Toc525269041)

[4. L-cuts – Figure 5 2](#_Toc525269042)

Further description of the individual files is provided below.

# Synapsin stain of single-headed wildtype planaria – Figure 1

15 Samples of single-headed planaria stained with the anti-SYNORF synapsin antibody, illustrating the general neuronal architecture of the animals. This data was used as the basis of the neuronal maps used in the model.

# Synapsin stain of double-headed wildtype planaria – Figure 7

15 Samples of double-headed planaria stained with the anti-SYNORF synapsin antibody, illustrating the general neuronal architecture of these animals. This shows the bipolar mirrored structure of the nervous system in the double-headed worms.

# Dynein inhibition – Ciliobrevin D treatment

This folder contains both brightfield images and synapsin stain images of headless worms following regeneration from trunk fragments treated with 3 µM Ciliobrevin D for 3 days. 10 regenerates are shown in the brightfield, illustrating the range of shape outcomes. 9 regenerates are shown stained with the anti-SYNORF synapsin antibody, illustrating absence of the brain and organization of the VNCs in headless animals.

# L-cuts – Figure 5

1. Control L-cuts

Brightfield images of 11 worms with a control L-cut, as well as 9 regenerates stained with the anti-SYNORF synapsin antibody.

1. Downwards L-cuts

Brightfield images of 14 fully regenerated worms with a downwards L-cut deviating the VNC into the side growth leading to regeneration of a side head upon completion of regeneration. Time-course of regeneration of fragments stained with the anti-SYNORF synapsin antibody, from 3 dpc (days post cutting) (1 sample), 5 dpc (1 sample), 7 dpc (3 samples) and final regeneration outcomes at 11 dpc (10 samples).

1. Upwards L-cuts

Brightfield images of 12 fully regenerated worms with an upwards L-cut deviating the VNC into the side growth leading to regeneration of a side tail upon completion of regeneration. Time-course of regeneration of fragments stained with the anti-SYNORF synapsin antibody, from 3 dpc (days post cutting) (1 sample), 5 dpc (3 samples) and final regeneration outcomes at 11 dpc (7 samples).

# 
