# Supplementary figures and images for: Neural control of body-plan axis in regenerating planaria (part 4 of 4)
Source: PLoS Comput Biol. 2019 Apr 16;15(4):e1006904. doi: 10.1371/journal.pcbi.1006904 (PMC6485777; doi:10.1371/journal.pcbi.1006904)

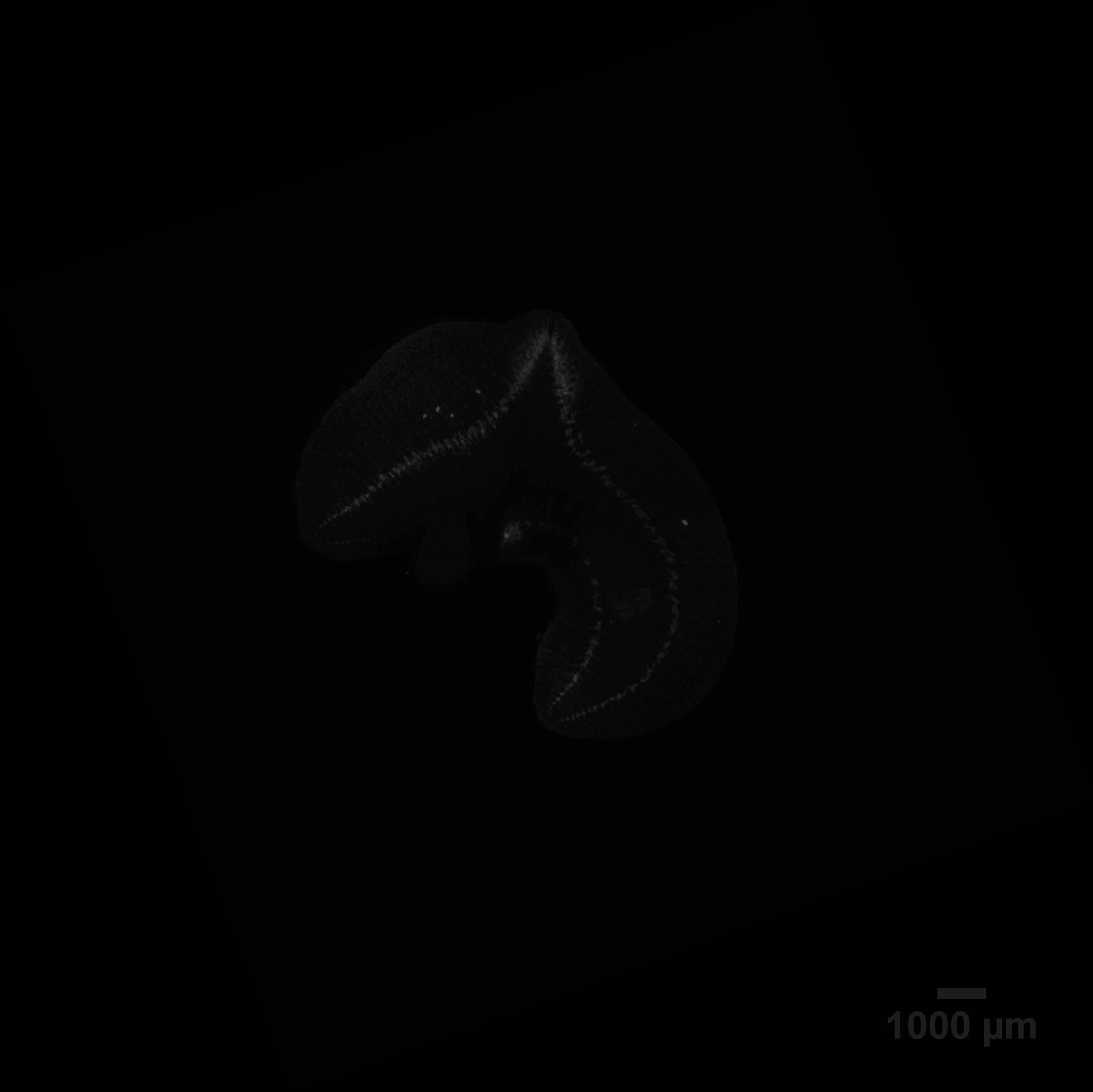

Supplement: S2 Dataset — This dataset contains raw-images of synapsin stains of uncut one- and two- headed worms, synapsin stains and brightfield images of the upwards and inverted L-cut scenarios, and synapsin stains and brightfield images showing the effects of the dynein inhibitor Ciliobrevin D on planaria regeneration. A Word document contained in the zip folder provides detailed description of the different cases. (ZIP) [file pcbi.1006904.s017.zip › DatasetS9i/L_cuts/c) upwards L-cut/synapsin stain/11 dpc_Sample 4.jpg]

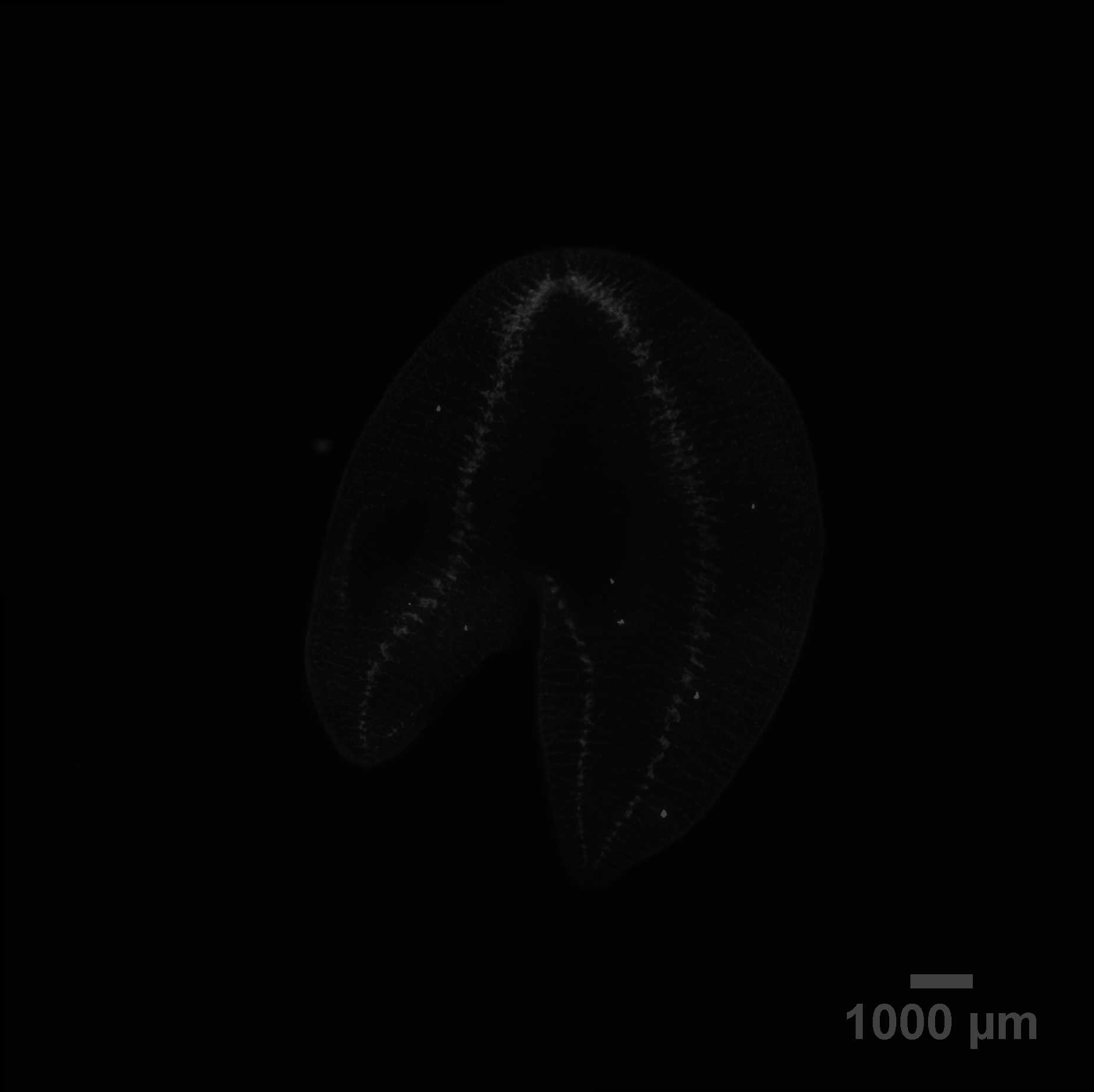

Supplement: S2 Dataset — This dataset contains raw-images of synapsin stains of uncut one- and two- headed worms, synapsin stains and brightfield images of the upwards and inverted L-cut scenarios, and synapsin stains and brightfield images showing the effects of the dynein inhibitor Ciliobrevin D on planaria regeneration. A Word document contained in the zip folder provides detailed description of the different cases. (ZIP) [file pcbi.1006904.s017.zip › DatasetS9i/L_cuts/c) upwards L-cut/synapsin stain/11 dpc_Sample 5.jpg]

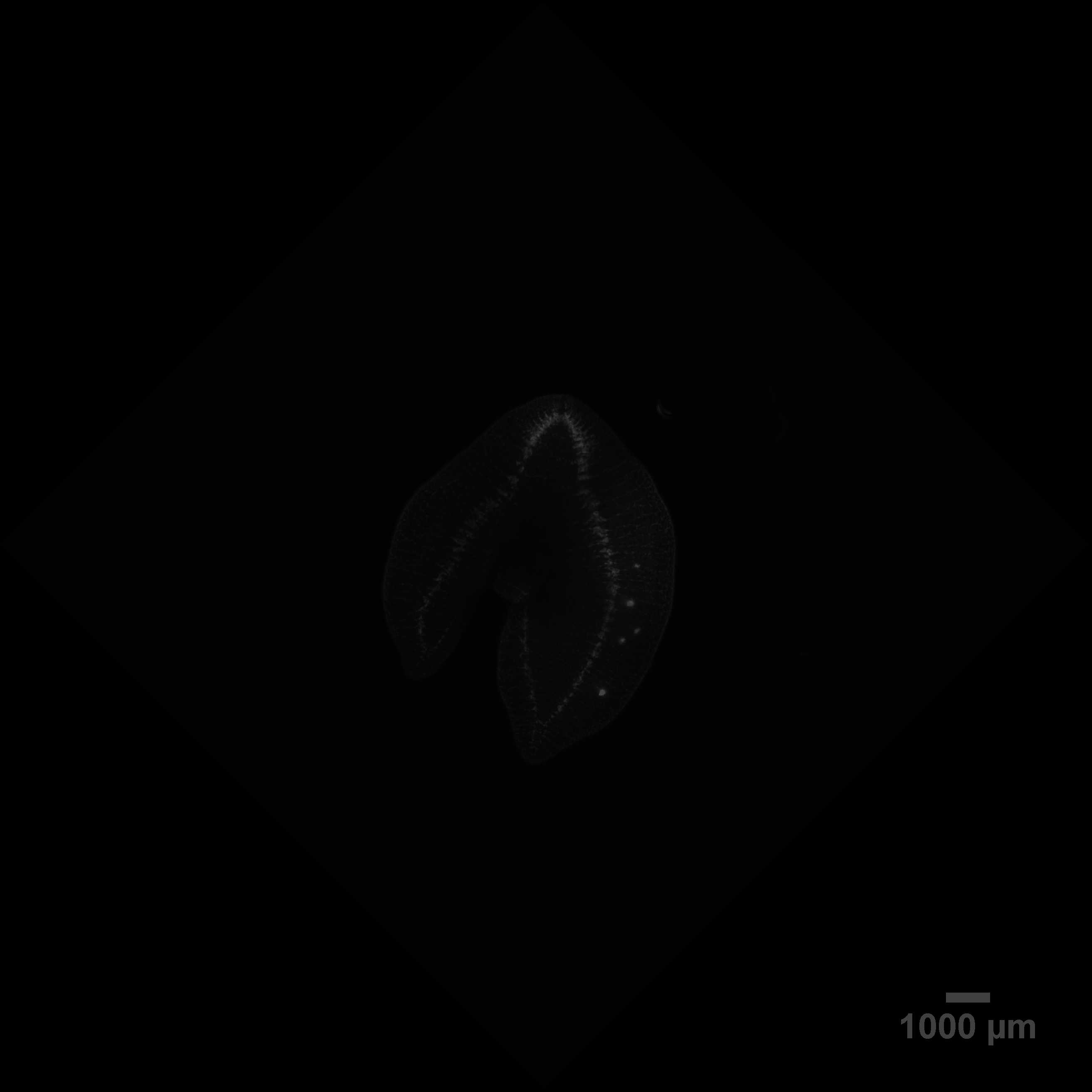

Supplement: S2 Dataset — This dataset contains raw-images of synapsin stains of uncut one- and two- headed worms, synapsin stains and brightfield images of the upwards and inverted L-cut scenarios, and synapsin stains and brightfield images showing the effects of the dynein inhibitor Ciliobrevin D on planaria regeneration. A Word document contained in the zip folder provides detailed description of the different cases. (ZIP) [file pcbi.1006904.s017.zip › DatasetS9i/L_cuts/c) upwards L-cut/synapsin stain/11 dpc_Sample 6.jpg]

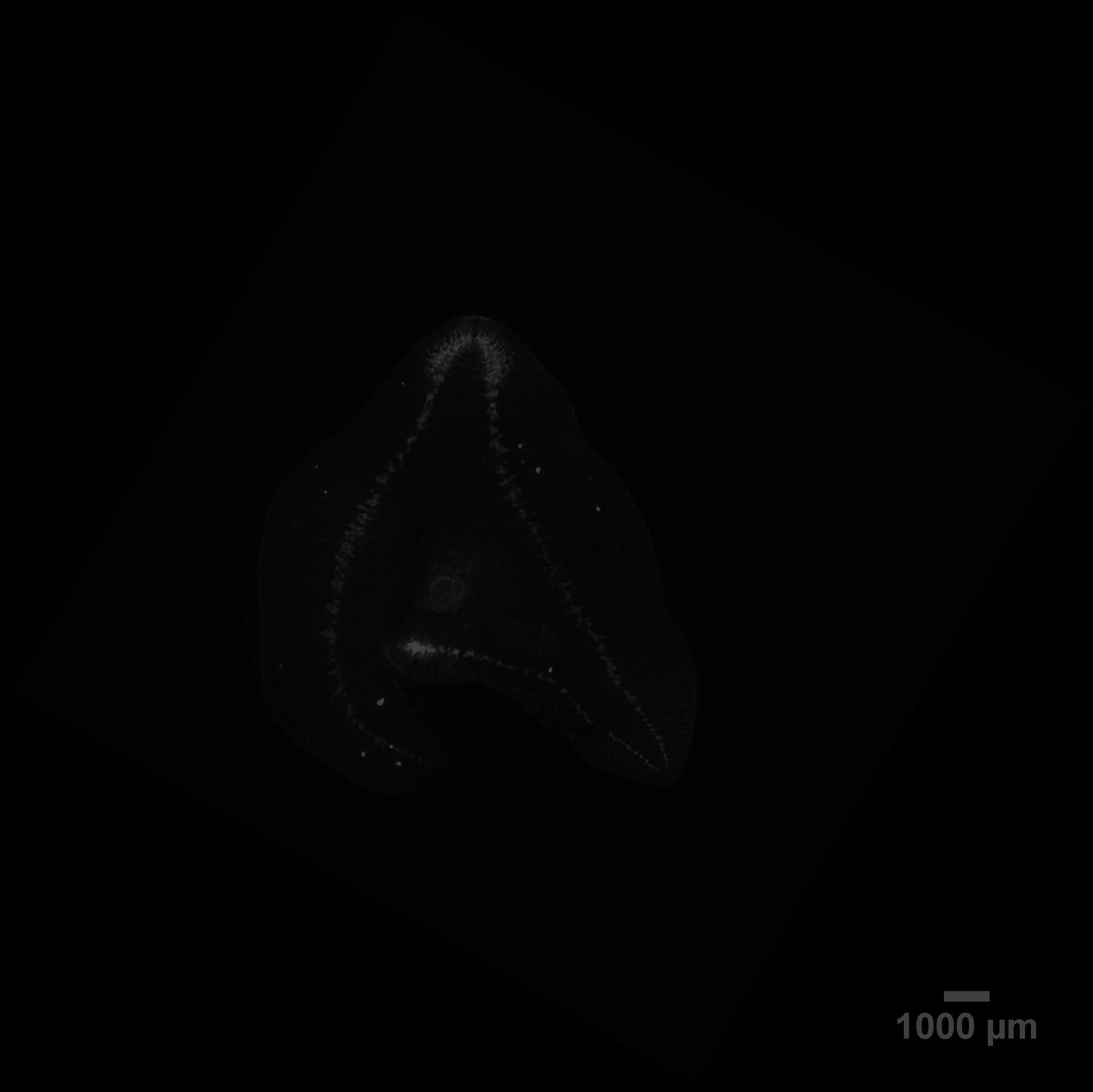

Supplement: S2 Dataset — This dataset contains raw-images of synapsin stains of uncut one- and two- headed worms, synapsin stains and brightfield images of the upwards and inverted L-cut scenarios, and synapsin stains and brightfield images showing the effects of the dynein inhibitor Ciliobrevin D on planaria regeneration. A Word document contained in the zip folder provides detailed description of the different cases. (ZIP) [file pcbi.1006904.s017.zip › DatasetS9i/L_cuts/c) upwards L-cut/synapsin stain/11 dpc_Sample 7.jpg]

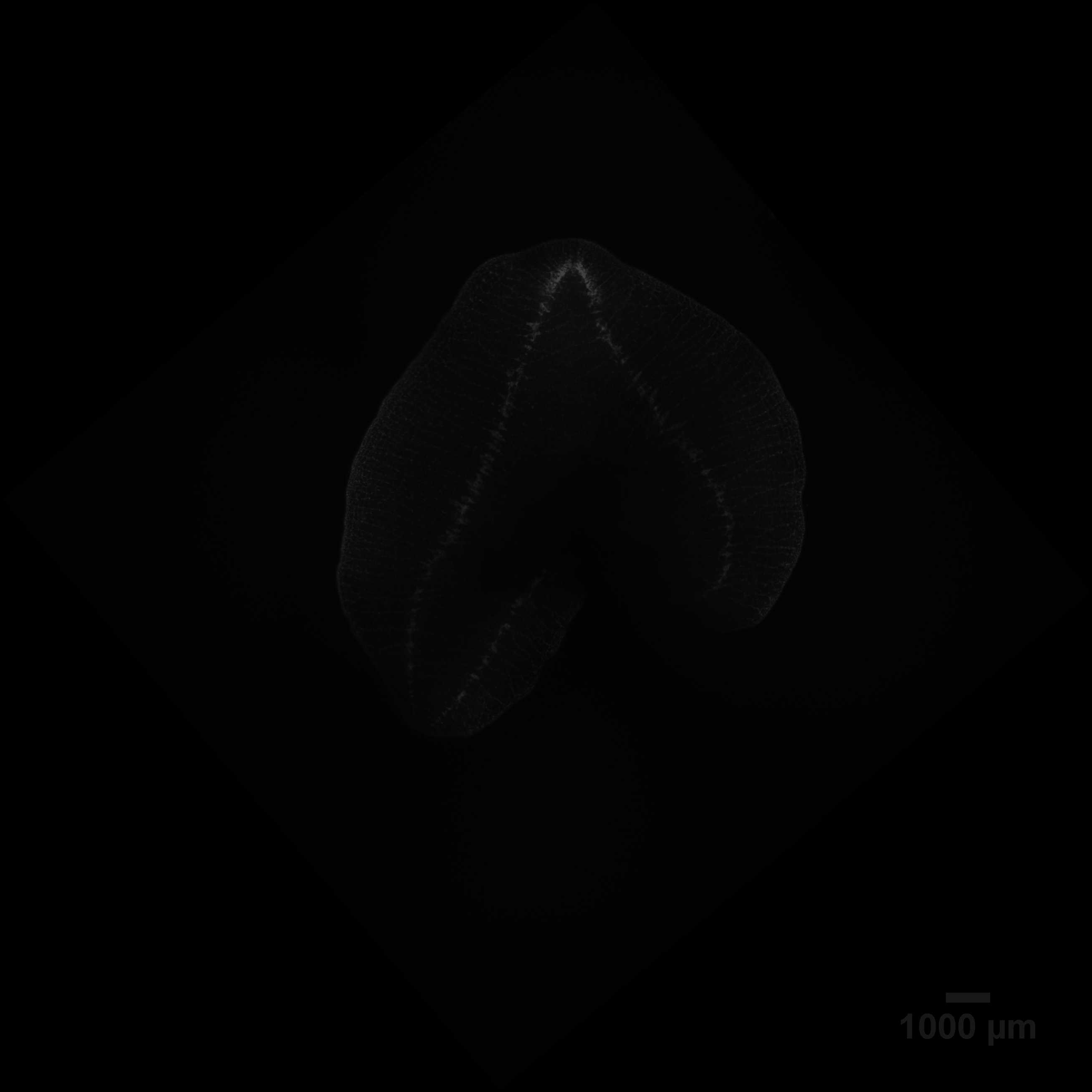

Supplement: S2 Dataset — This dataset contains raw-images of synapsin stains of uncut one- and two- headed worms, synapsin stains and brightfield images of the upwards and inverted L-cut scenarios, and synapsin stains and brightfield images showing the effects of the dynein inhibitor Ciliobrevin D on planaria regeneration. A Word document contained in the zip folder provides detailed description of the different cases. (ZIP) [file pcbi.1006904.s017.zip › DatasetS9i/L_cuts/c) upwards L-cut/synapsin stain/5 dpc_Sample 1.jpg]

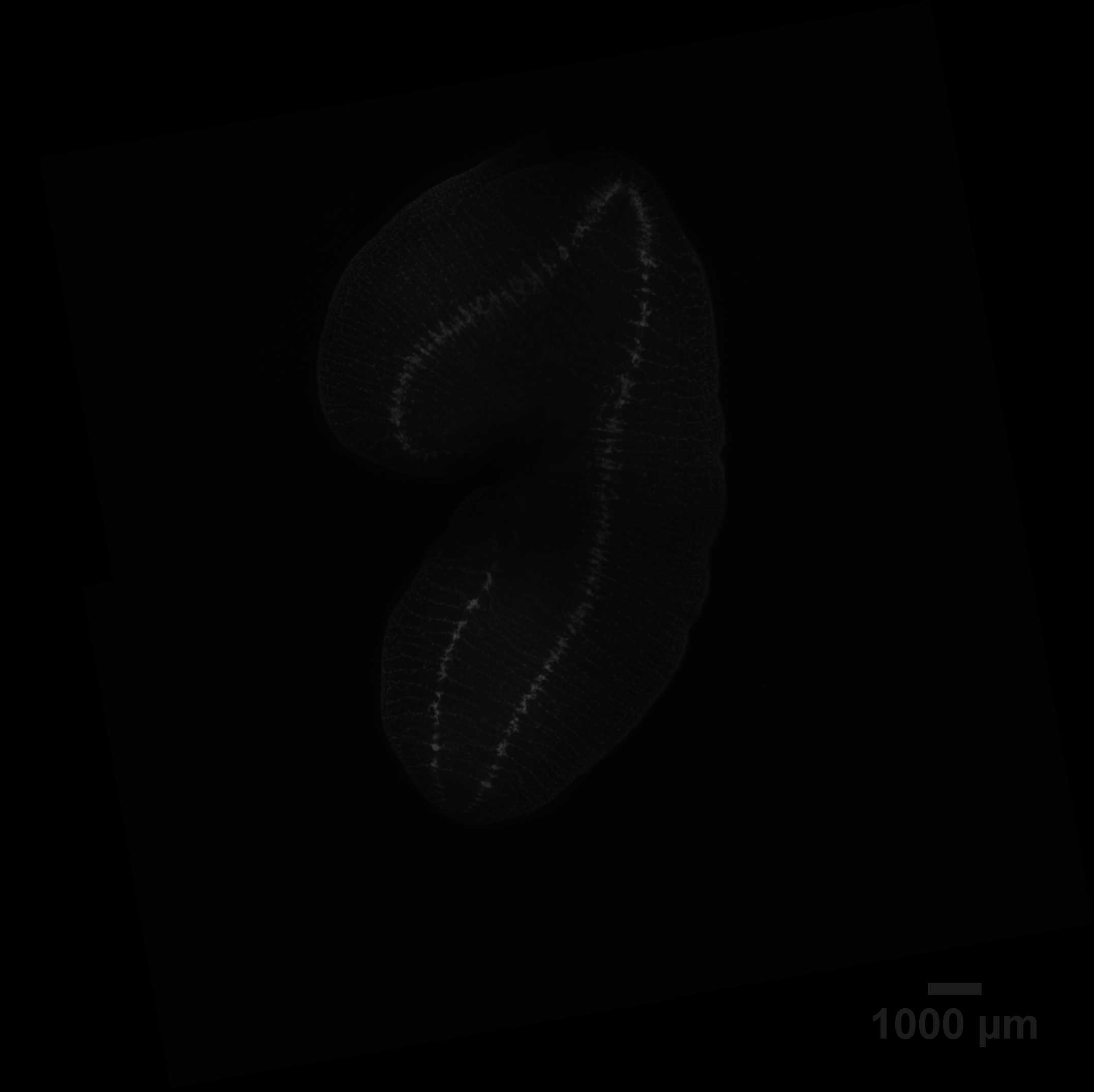

Supplement: S2 Dataset — This dataset contains raw-images of synapsin stains of uncut one- and two- headed worms, synapsin stains and brightfield images of the upwards and inverted L-cut scenarios, and synapsin stains and brightfield images showing the effects of the dynein inhibitor Ciliobrevin D on planaria regeneration. A Word document contained in the zip folder provides detailed description of the different cases. (ZIP) [file pcbi.1006904.s017.zip › DatasetS9i/L_cuts/c) upwards L-cut/synapsin stain/5 dpc_Sample 3.jpg]
